# Supplementary material for: Biosensor-integrated transposon mutagenesis reveals rv0158 as a coordinator of redox homeostasis in Mycobacterium tuberculosis
Source: eLife. 2023 Aug 29;12:e80218. doi: 10.7554/eLife.80218 (PMC10501769; doi:10.7554/eLife.80218)

Batch Analysis Report

Run Date: 2/2/16 3:41 PM Experiment: 02Feb2016 Bact Sorting
User ID: Administrator
Statistics Output: C:\Users\Admin\Desktop\02Feb2016 Bact Sorting-Batch_Analysis_02022016154146.csv
Worksheet PDF Output: C:\Users\Admin\Desktop\02Feb2016 Bact Sorting-Batch_Analysis_02022016154146.pdf

Specimen_001

Tube	Status	Run Time
RV US	OK	2/2/16 3:41 PM
mrx1 rogfp	OK	2/2/16 3:41 PM
mrx1 rogfp_001	OK	2/2/16 3:41 PM
TN40K_001	OK	2/2/16 3:41 PM
TN40K_002	OK	2/2/16 3:41 PM
TN40K_003	OK	2/2/16 3:41 PM
post sort	OK	2/2/16 3:42 PM

BD FACSDiva 8.0.1

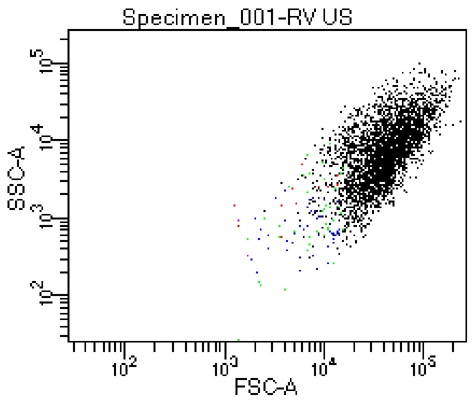

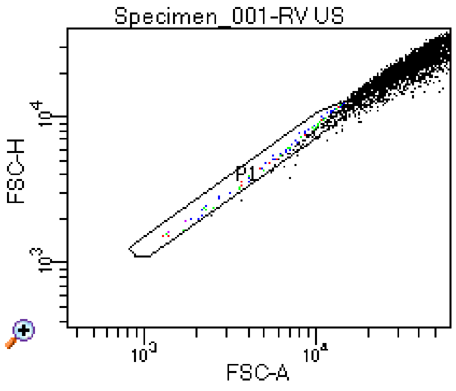

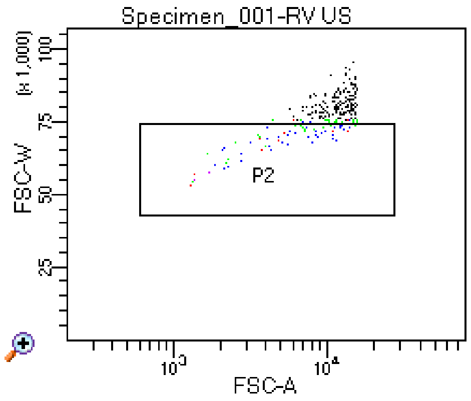

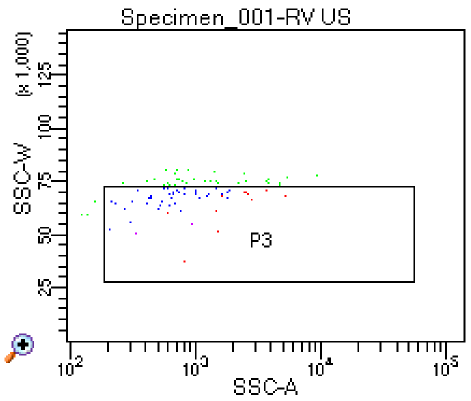

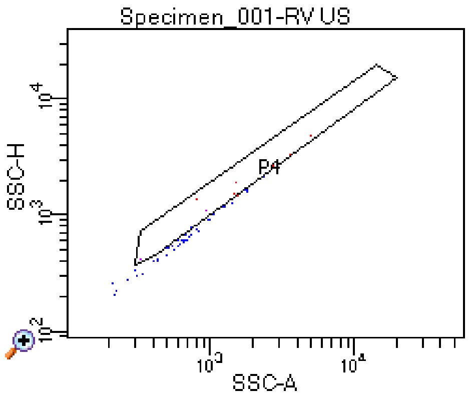

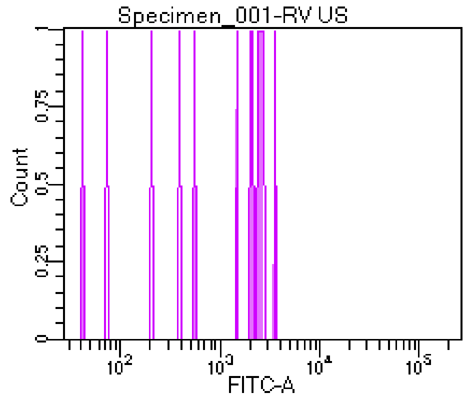

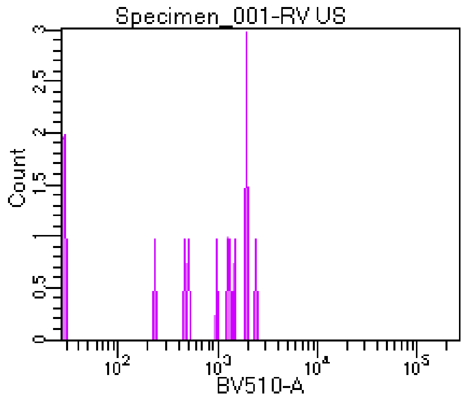

Experiment Name:	02Feb2016 Bact Sorting
Specimen Name:	Specimen_001
Tube Name:	RV US
Record Date:	Feb 2, 2016 2:52:28 PM
$OP:	Administrator
GUID:	76a8b5c1-5df2-4417-83e7-c64...

Population	#Events	%Parent	FITC-A Median	BV510-A Median
All Events	3,500	####	8,128	5,347
P1	249	7.1	2,619	1,856
P2	95	38.2	1,193	777
P3	56	58.9	1,163	742
P4	13	23.2	1,870	1,189
P5	11	84.6	1,942	1,248

Tube: RV US

Population	#Events	%Parent	%Total
All Events	3,500	####	100.0
P1	249	7.1	7.1
P2	95	38.2	2.7
P3	56	58.9	1.6
P4	13	23.2	0.4
P5	11	84.6	0.3

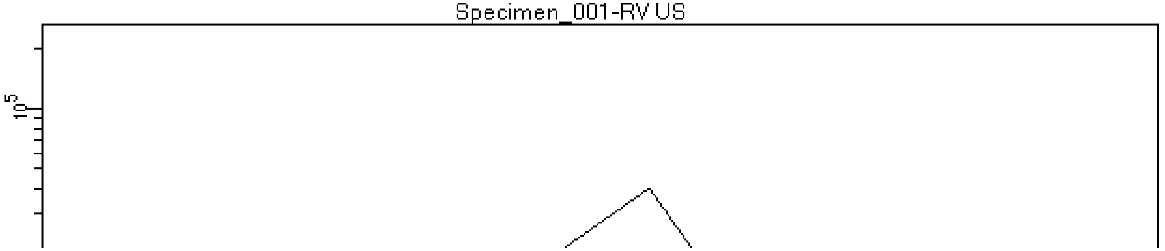

BD FACSDiva 8.0.1

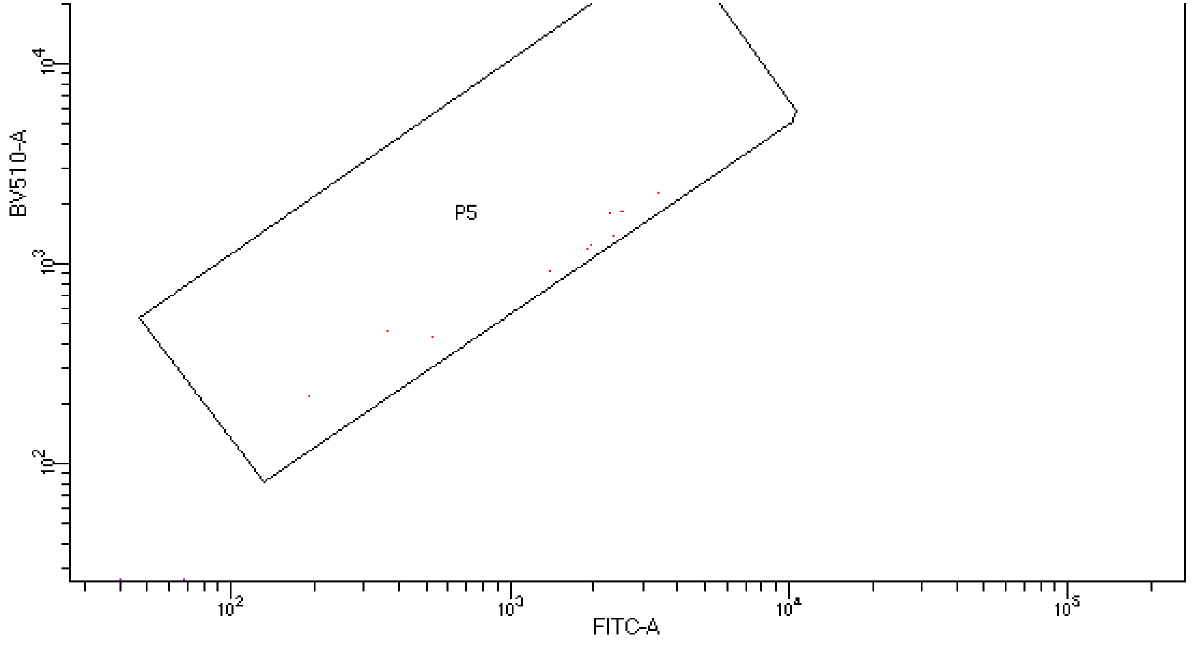

BD FACSDiva 8.0.1

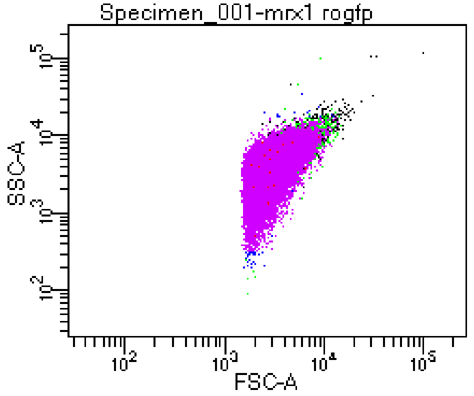

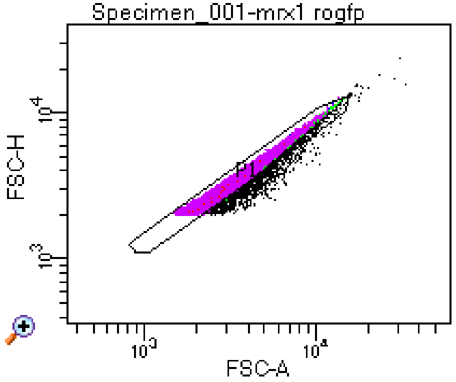

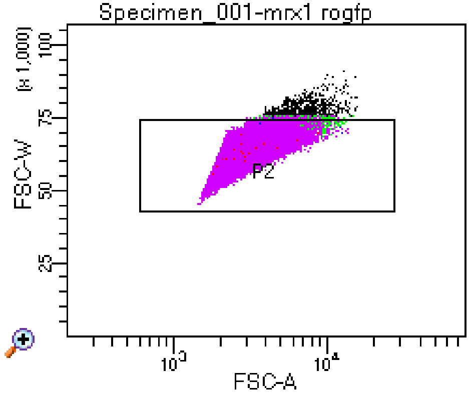

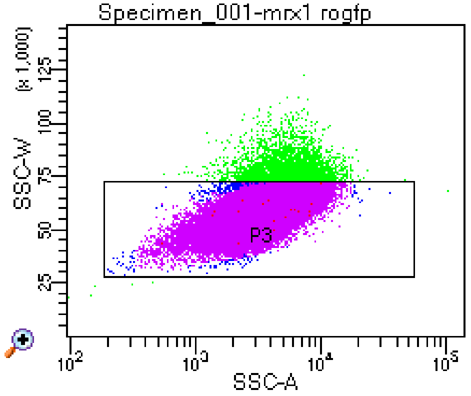

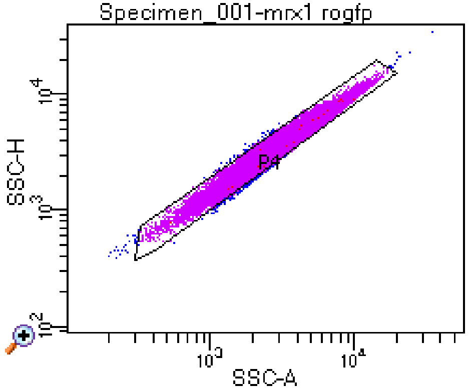

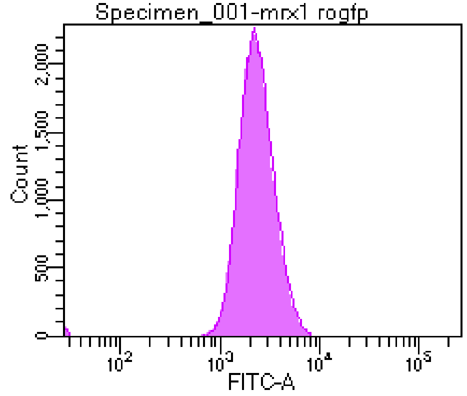

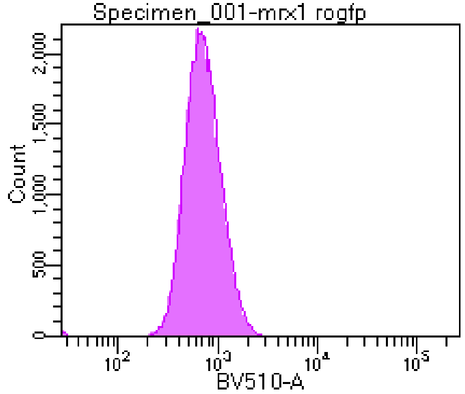

Experiment Name:	02Feb2016 Bact Sorting
Specimen Name:	Specimen_001
Tube Name:	mrx1 rogfp
Record Date:	Feb 2, 2016 2:56:11 PM
$OP:	Administrator
GUID:	7e6bfa69-92af-4b80-b5ab-4aaf...

Population	#Events	%Parent	FITC-A Median	BV510-A Median
All Events	67,381	####	2,230	679
P1	65,644	97.4	2,206	671
P2	64,956	99.0	2,194	668
P3	60,963	93.9	2,138	651
P4	60,475	99.2	2,138	651
P5	18	0.0	604	832

Tube: mrx1 rogfp

Population	#Events	%Parent	%Total
All Events	67,381	####	100.0
P1	65,644	97.4	97.4
P2	64,956	99.0	96.4
P3	60,963	93.9	90.5
P4	60,475	99.2	89.8
P5	18	0.0	0.0

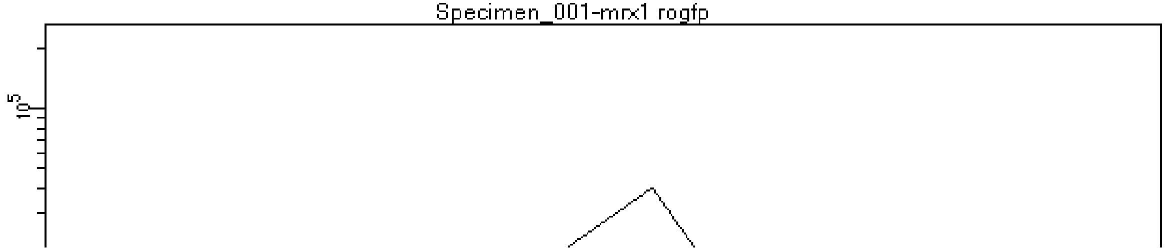

BD FACSDiva 8.0.1

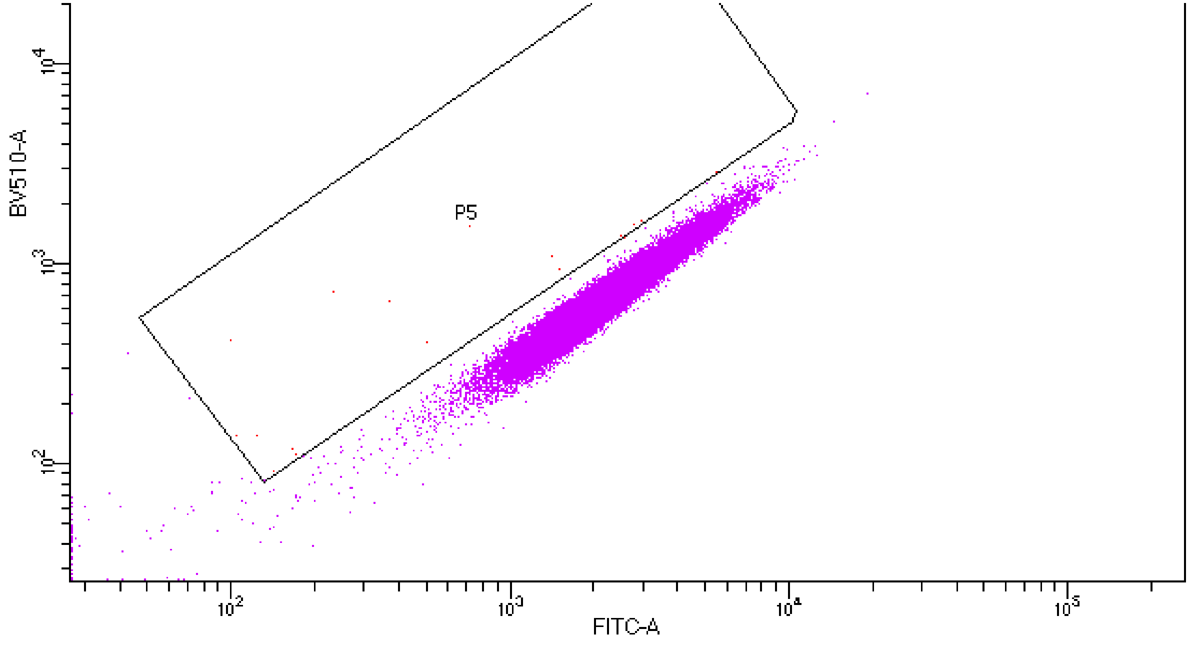

BD FACSDiva 8.0.1

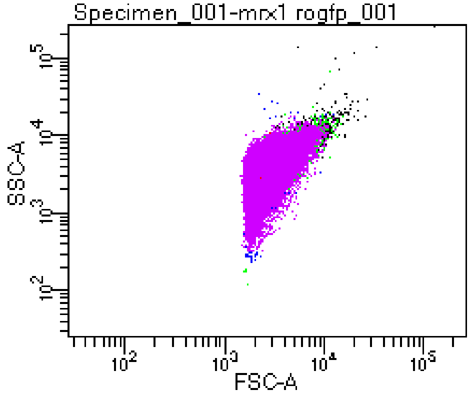

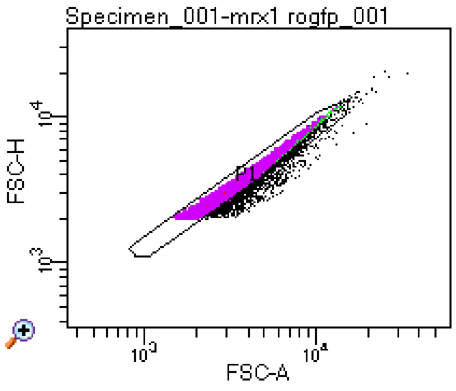

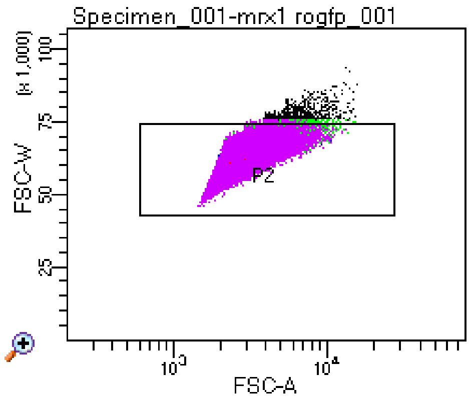

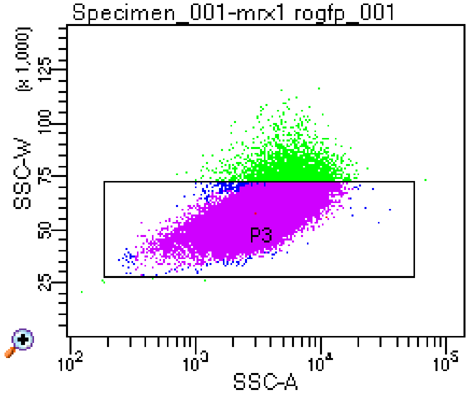

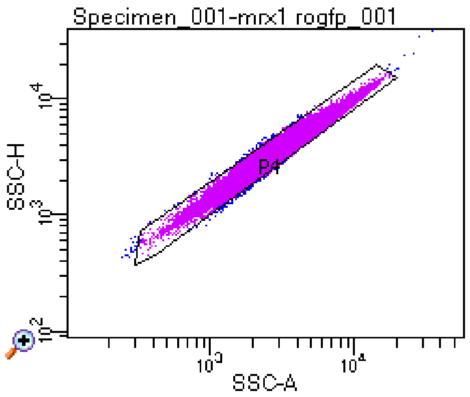

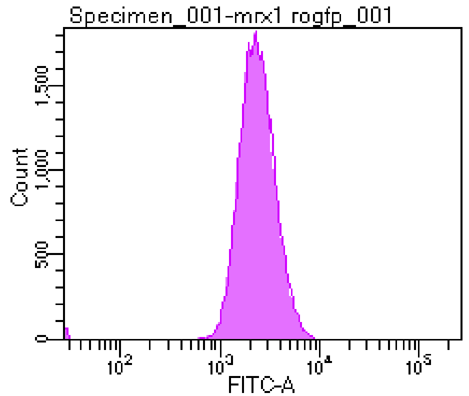

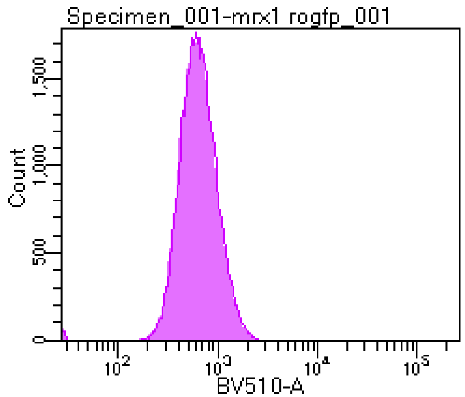

Experiment Name:	02Feb2016 Bact Sorting
Specimen Name:	Specimen_001
Tube Name:	mrx1 rogfp_001
Record Date:	Feb 2, 2016 2:57:41 PM
$OP:	Administrator
GUID:	31646a9c-dfff-4a26-990f-fc8905...

Population	#Events	%Parent	FITC-A Median	BV510-A Median
All Events	56,173	####	2,279	611
P1	54,708	97.4	2,253	605
P2	54,110	98.9	2,240	601
P3	50,718	93.7	2,182	584
P4	50,343	99.3	2,182	584
P5	2	0.0	1,978	1,515

Tube: mrx1 rogfp_001

Population	#Events	%Parent	%Total
All Events	56,173	####	100.0
P1	54,708	97.4	97.4
P2	54,110	98.9	96.3
P3	50,718	93.7	90.3
P4	50,343	99.3	89.6
P5	2	0.0	0.0

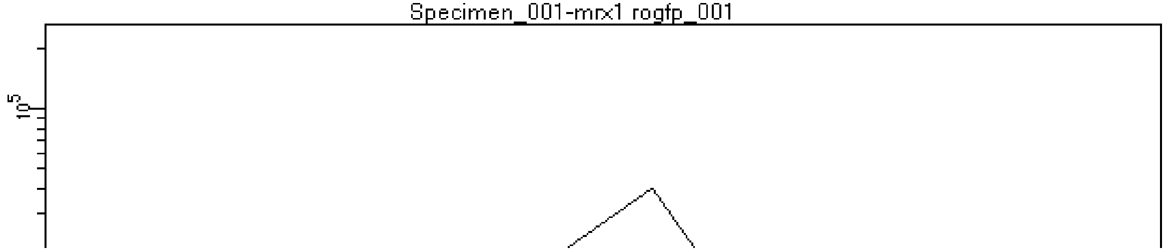

BD FACSDiva 8.0.1

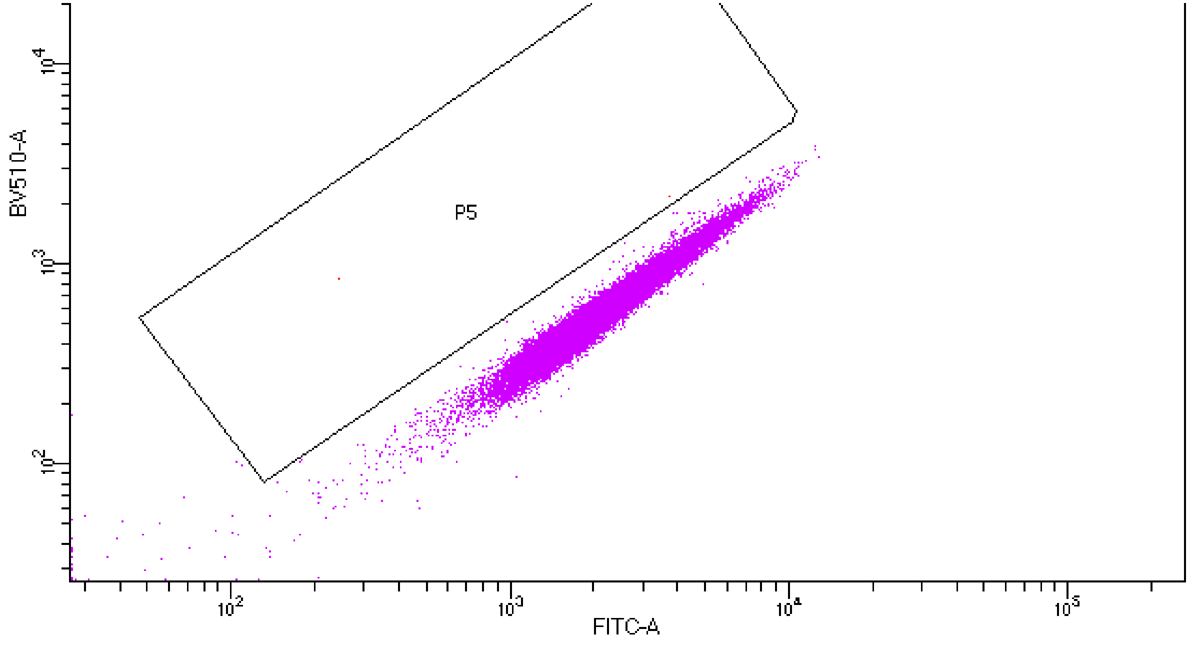

BD FACSDiva 8.0.1

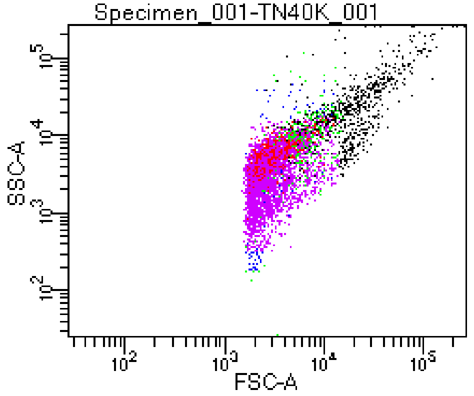

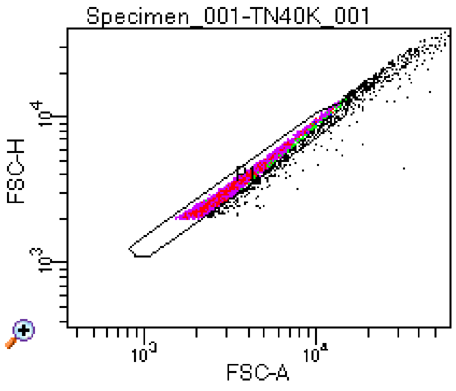

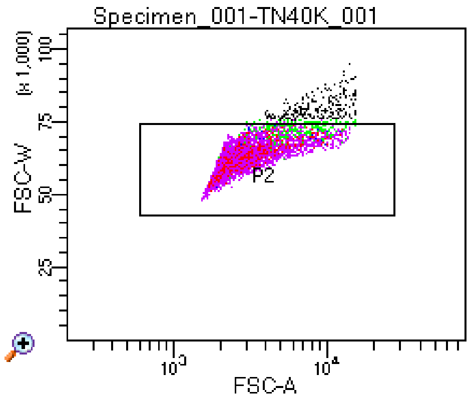

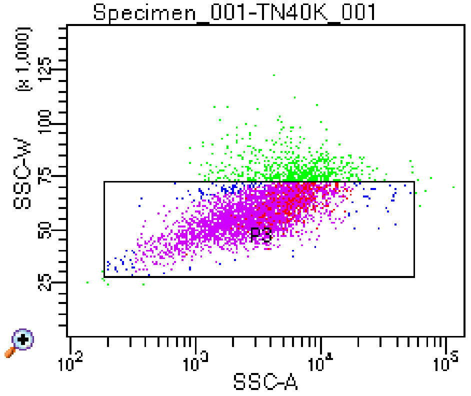

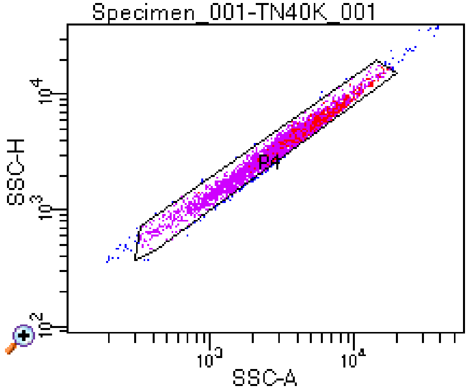

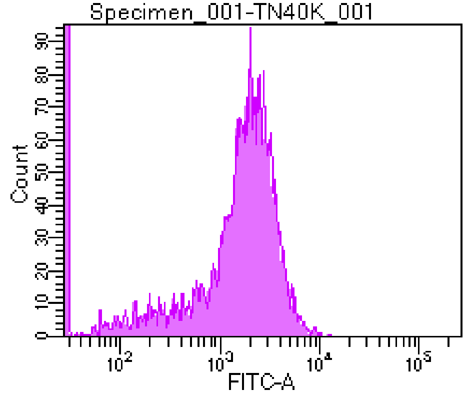

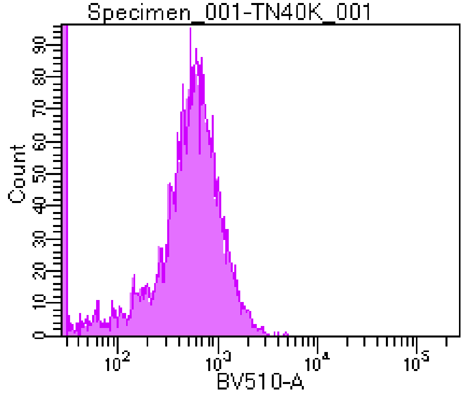

Experiment Name:	02Feb2016 Bact Sorting
Specimen Name:	Specimen_001
Tube Name:	TN40K_001
Record Date:	Feb 2, 2016 3:00:12 PM
$OP:	Administrator
GUID:	311d294c-0d44-4291-a4b9-2db...

Population	#Events	%Parent	FITC-A Median	BV510-A Median
All Events	5,490	####	1,661	529
P1	4,800	87.4	1,596	503
P2	4,560	95.0	1,537	487
P3	3,874	85.0	1,464	458
P4	3,731	96.3	1,470	460
P5	272	7.3	360	322

Tube: TN40K_001

Population	#Events	%Parent	%Total
All Events	5,490	####	100.0
P1	4,800	87.4	87.4
P2	4,560	95.0	83.1
P3	3,874	85.0	70.6
P4	3,731	96.3	68.0
P5	272	7.3	5.0

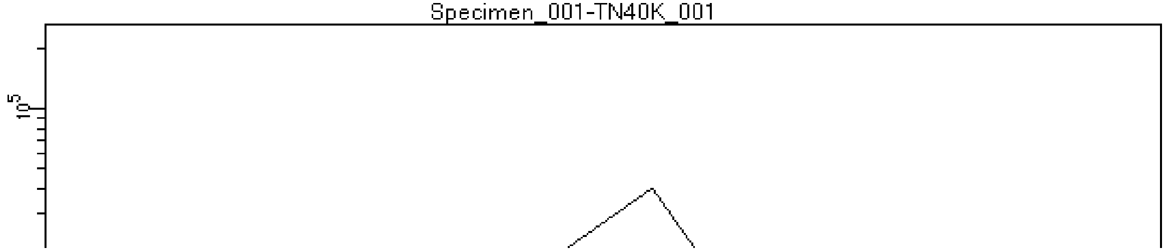

BD FACSDiva 8.0.1

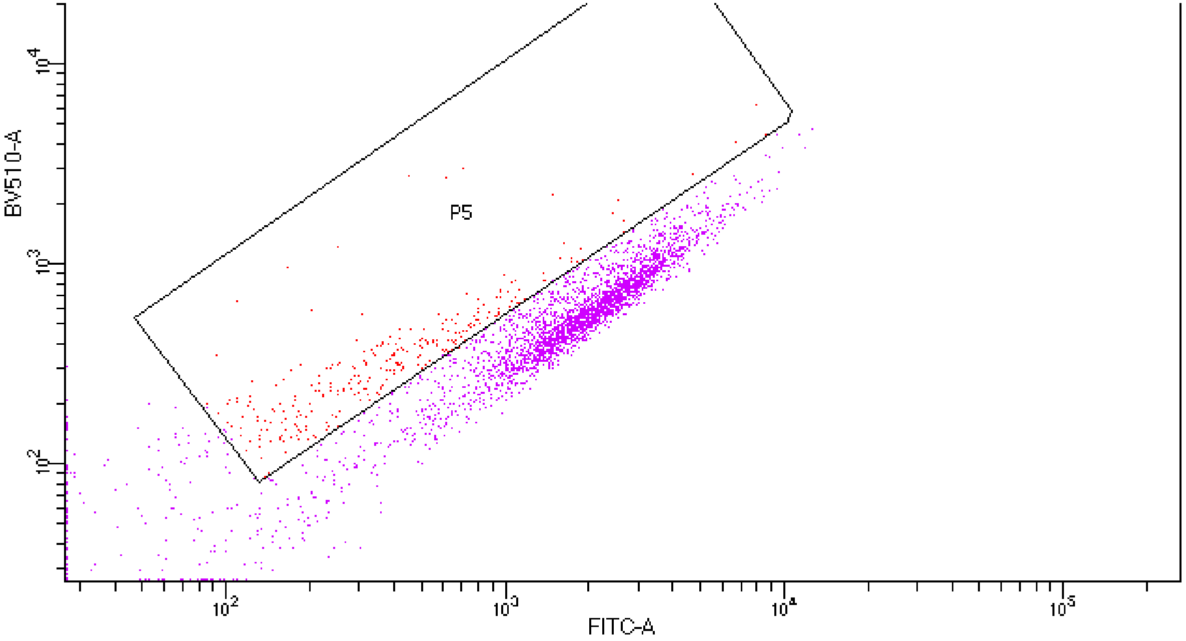

BD FACSDiva 8.0.1

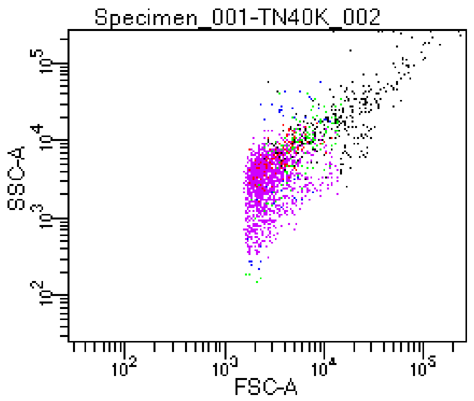

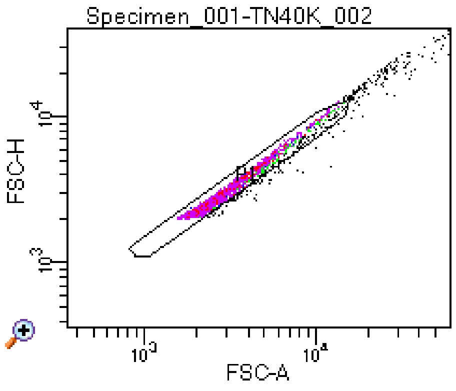

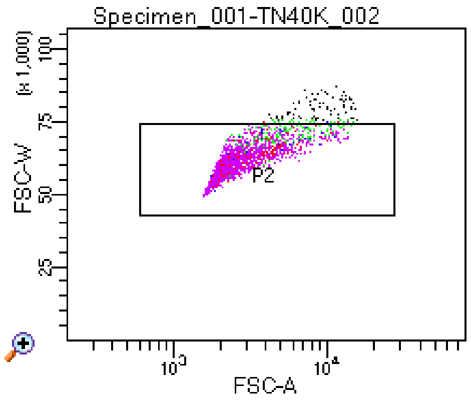

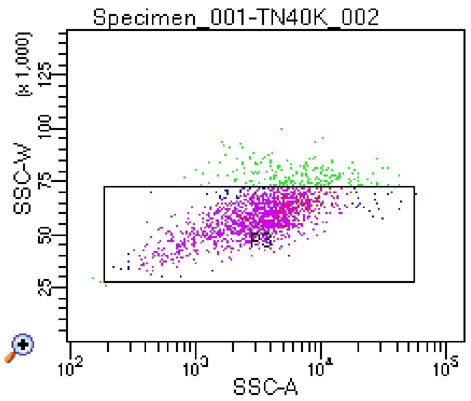

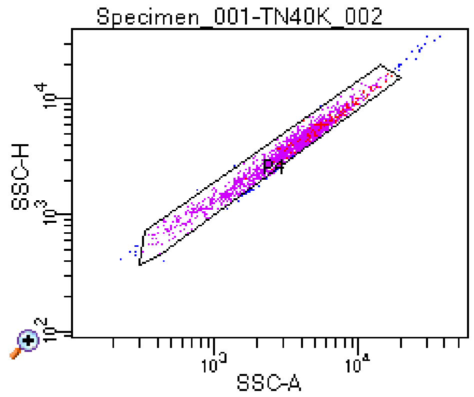

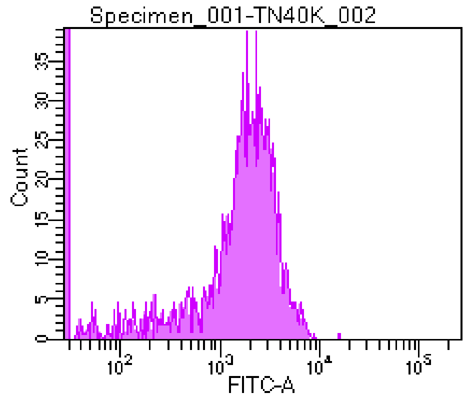

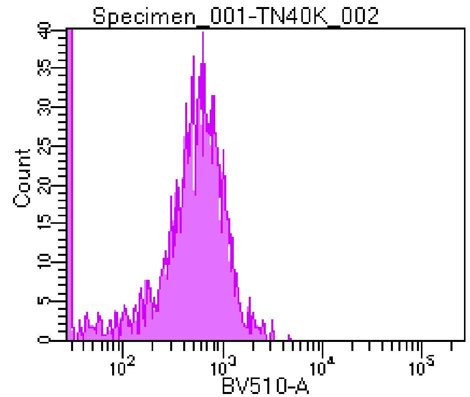

Experiment Name:	02Feb2016 Bact Sorting
Specimen Name:	Specimen_001
Tube Name:	TN40K_002
Record Date:	Feb 2, 2016 3:01:53 PM
$OP:	Administrator
GUID:	e7ed7dbe-4a26-4374-8e8a-da...

Population	#Events	%Parent	FITC-A Median	BV510-A Median
All Events	2,075	####	1,662	529
P1	1,816	87.5	1,592	498
P2	1,739	95.8	1,560	480
P3	1,521	87.5	1,504	450
P4	1,459	95.9	1,531	454
P5	97	6.6	325	318

Tube: TN40K_002

Population	#Events	%Parent	%Total
All Events	2,075	####	100.0
P1	1,816	87.5	87.5
P2	1,739	95.8	83.8
P3	1,521	87.5	73.3
P4	1,459	95.9	70.3
P5	97	6.6	4.7

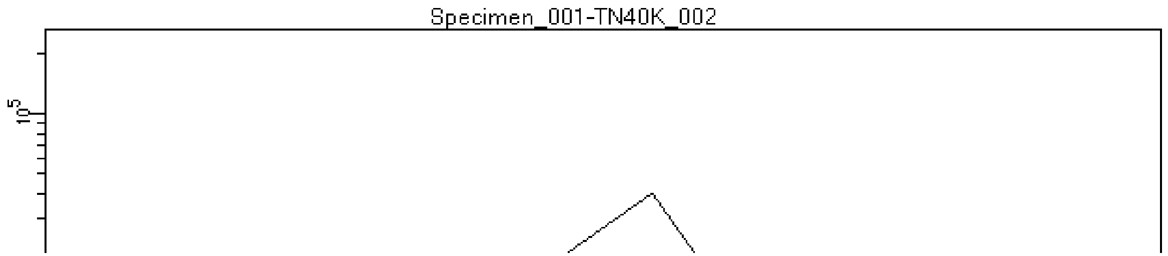

BD FACSDiva 8.0.1

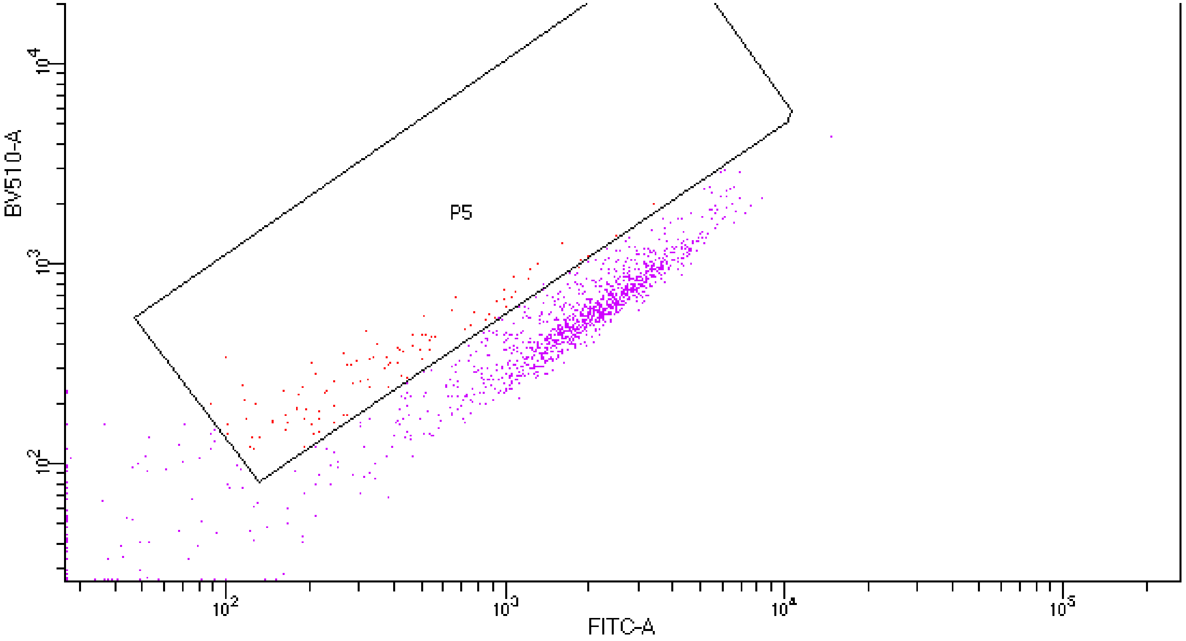

BD FACSDiva 8.0.1

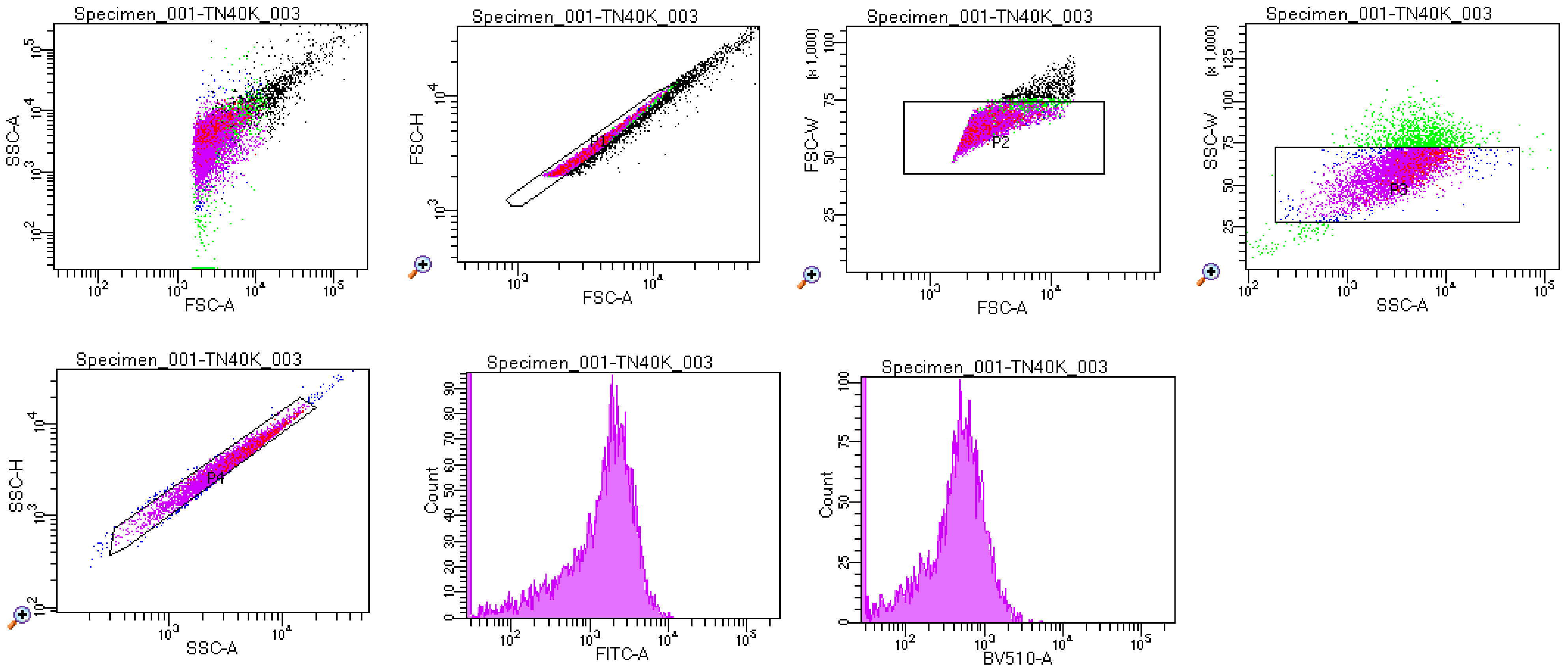

Experiment Name:	02Feb2016 Bact Sorting
Specimen Name:	Specimen_001
Tube Name:	TN40K_003
Record Date:	Feb 2, 2016 3:19:44 PM
$OP:	Administrator
GUID:	645e243a-8fd5-489e-821d-1df1...

Population	#Events	%Parent	FITC-A Median	BV510-A Median
All Events	7,544	####	1,518	464
P1	6,496	86.1	1,393	425
P2	5,990	92.2	1,347	407
P3	4,694	78.4	1,271	380
P4	4,494	95.7	1,274	383
P5	272	6.1	399	322

Tube: TN40K_003

Population	#Events	%Parent	%Total
All Events	7,544	####	100.0
P1	6,496	86.1	86.1
P2	5,990	92.2	79.4
P3	4,694	78.4	62.2
P4	4,494	95.7	59.6
P5	272	6.1	3.6

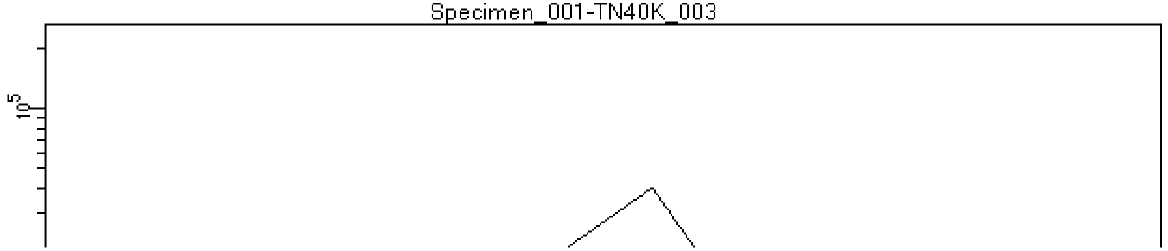

BD FACSDiva 8.0.1

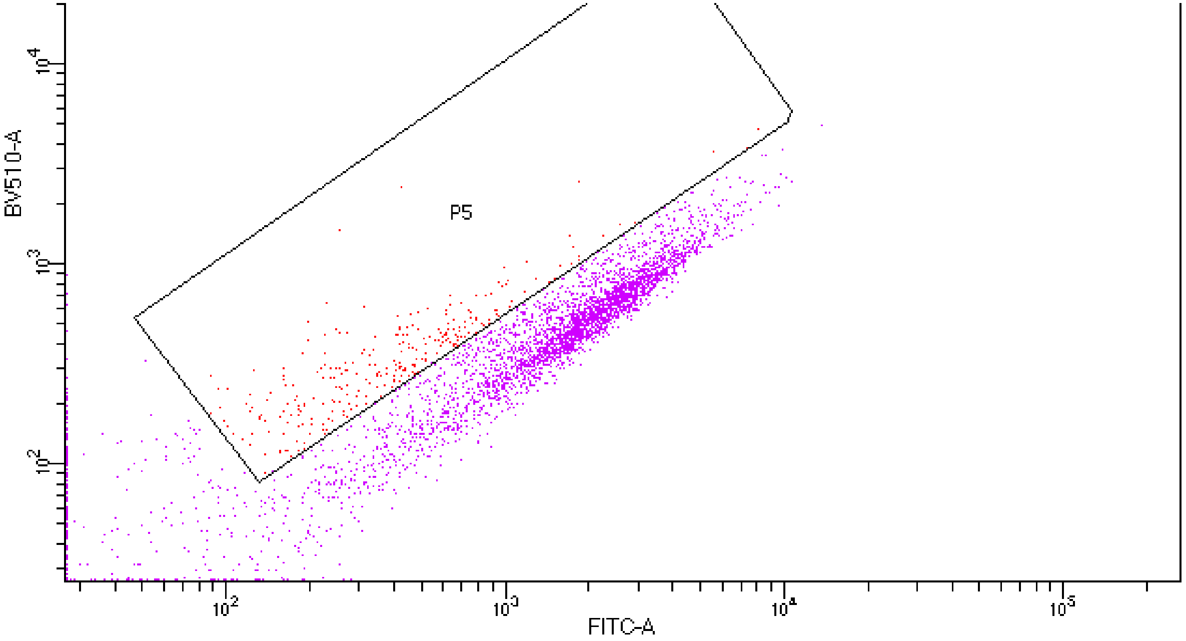

BD FACSDiva 8.0.1

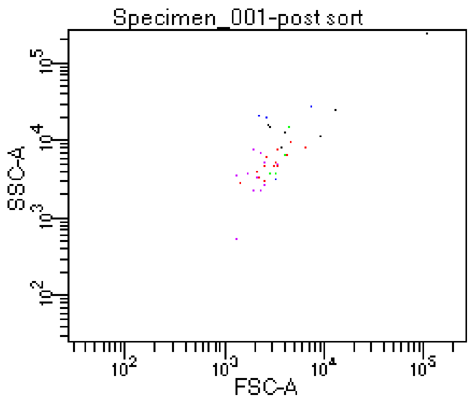

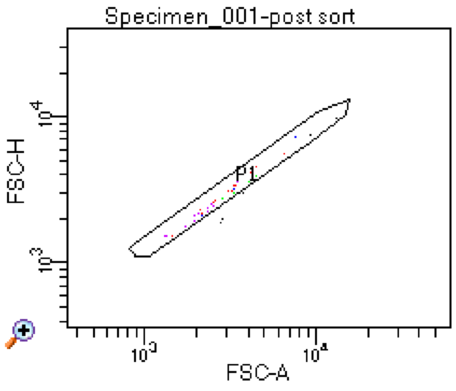

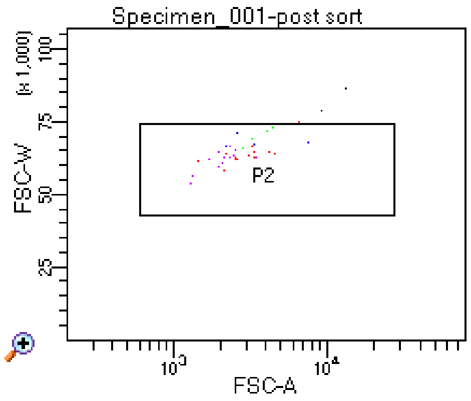

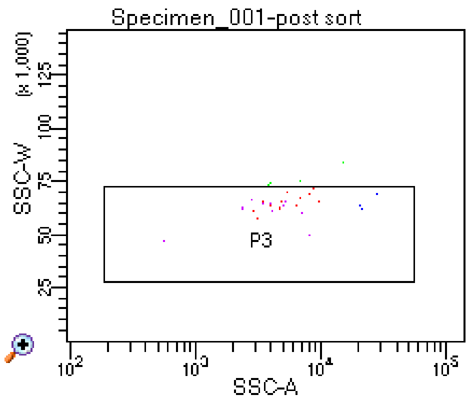

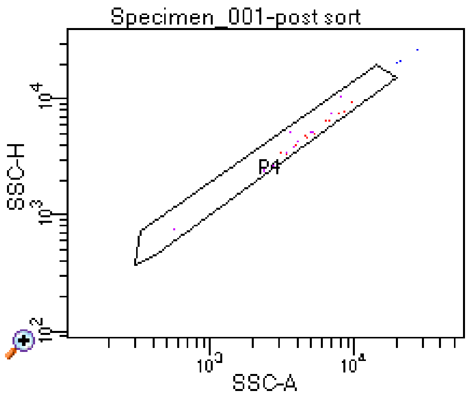

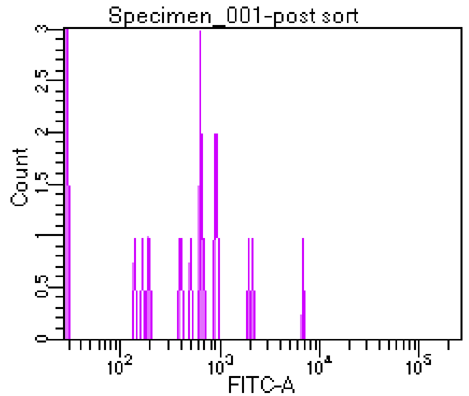

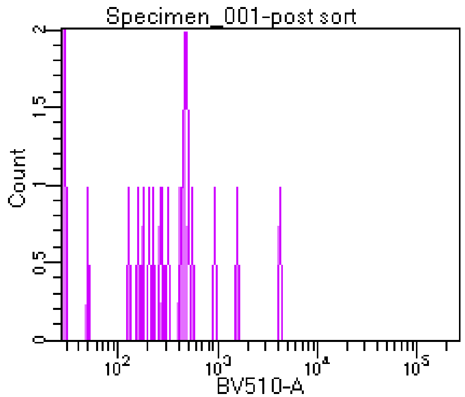

Experiment Name:	02Feb2016 Bact Sorting
Specimen Name:	Specimen_001
Tube Name:	post sort
Record Date:	Feb 2, 2016 3:41:21 PM
$OP:	Administrator
GUID:	d93fe79e-0009-4c05-9bc0-8d6...

Population	#Events	%Parent	FITC-A Median	BV510-A Median
All Events	40	####	417	279
P1	35	87.5	486	290
P2	33	94.3	465	268
P3	29	87.9	465	268
P4	25	86.2	586	290
P5	13	52.0	465	393

Tube: post sort

Population	#Events	%Parent	%Total
All Events	40	####	100.0
P1	35	87.5	87.5
P2	33	94.3	82.5
P3	29	87.9	72.5
P4	25	86.2	62.5
P5	13	52.0	32.5

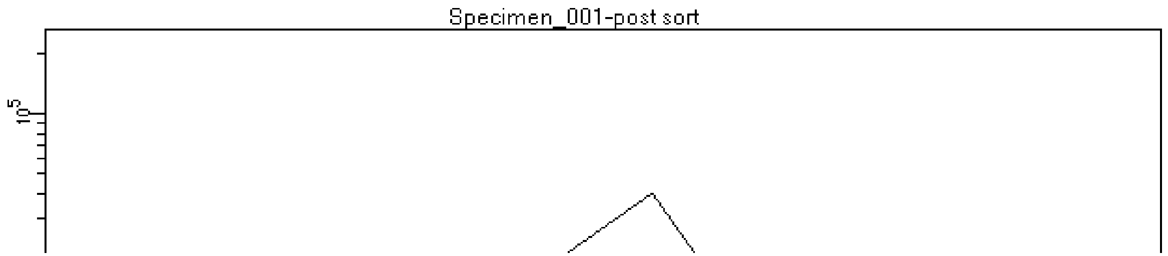

BD FACSDiva 8.0.1

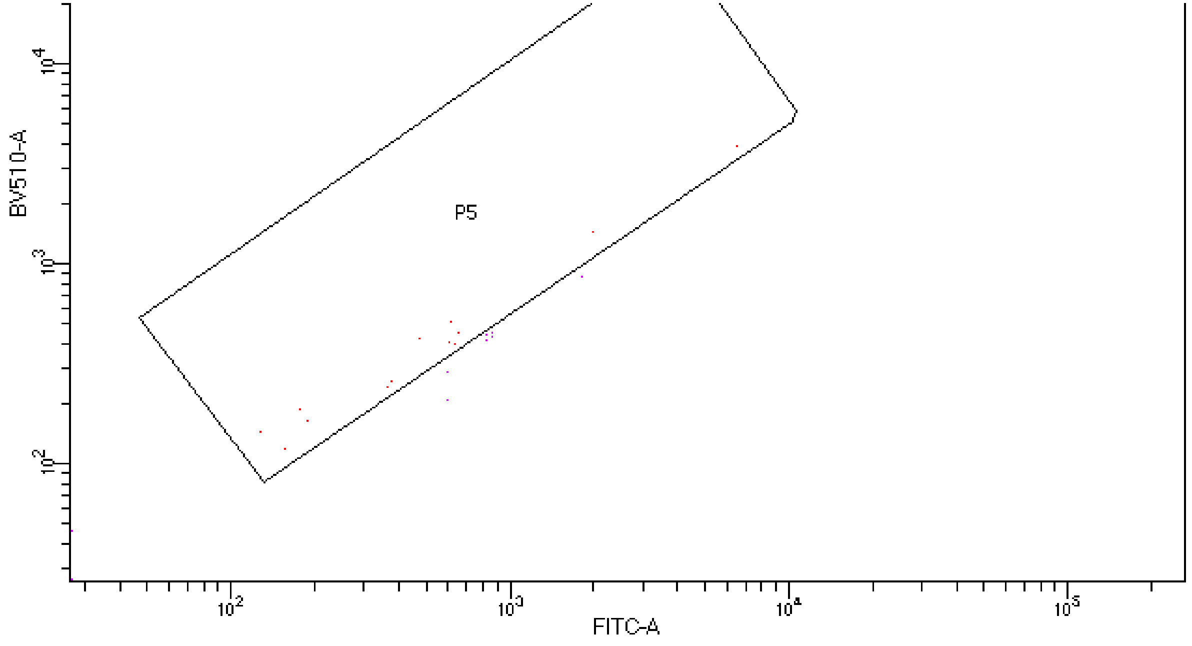

Supplement: Figure 1—source data 1. [file elife-80218-fig1-data1.zip › Round 1 Sorting/02Feb2016 Bact Sorting-Batch_Analysis_02022016154146.pdf]
