# Supplementary material for: Biosensor-integrated transposon mutagenesis reveals rv0158 as a coordinator of redox homeostasis in Mycobacterium tuberculosis
Source: eLife. 2023 Aug 29;12:e80218. doi: 10.7554/eLife.80218 (PMC10501769; doi:10.7554/eLife.80218)
Supplement: Figure 1—source data 1. [file elife-80218-fig1-data1.zip › Round 1 Sorting/03Feb2016 Bact Sorting-Batch_Analysis_03022016171529.pdf]

Batch Analysis Report

Run Date: 2/3/16 5:15 PM Experiment: 03Feb2016 Bact Sorting
User ID: Administrator
Statistics Output: C:\Users\Admin\Desktop\03Feb2016 Bact Sorting-Batch_Analysis_03022016171529.csv
Worksheet PDF Output: C:\Users\Admin\Desktop\03Feb2016 Bact Sorting-Batch_Analysis_03022016171529.pdf

Specimen_001

Tube	Status	Run Time
RV US	OK	2/3/16 5:15 PM
RV Mrx1	OK	2/3/16 5:15 PM
RV Mrx1_001	OK	2/3/16 5:15 PM
RV Mrx1 CHP	OK	2/3/16 5:15 PM
RV Mrx1 CHP_001	OK	2/3/16 5:15 PM
RV Mrx1 DTT	OK	2/3/16 5:15 PM
RV Mrx1 DTT_001	OK	2/3/16 5:15 PM
TN 40K	OK	2/3/16 5:15 PM
TN 40K_001	OK	2/3/16 5:15 PM
post sort	OK	2/3/16 5:15 PM

BD FACSDiva 8.0.1

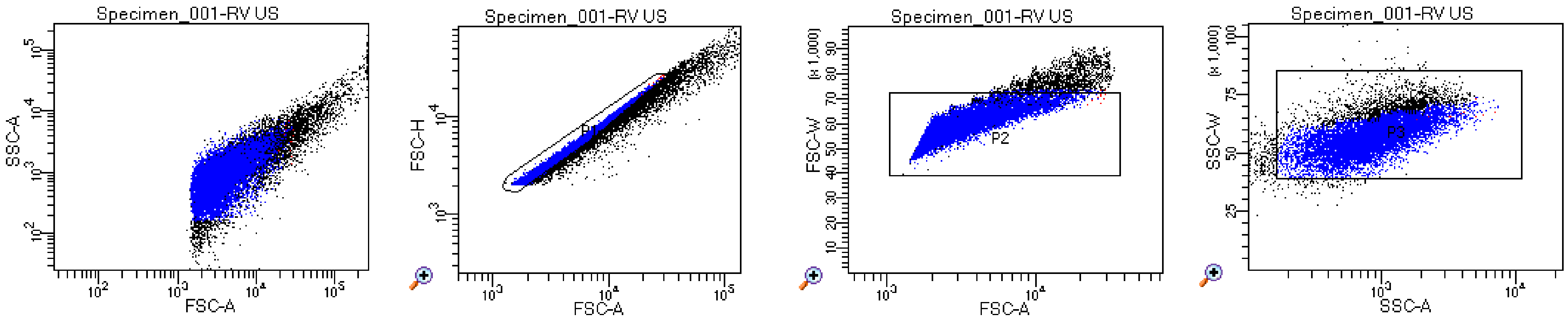

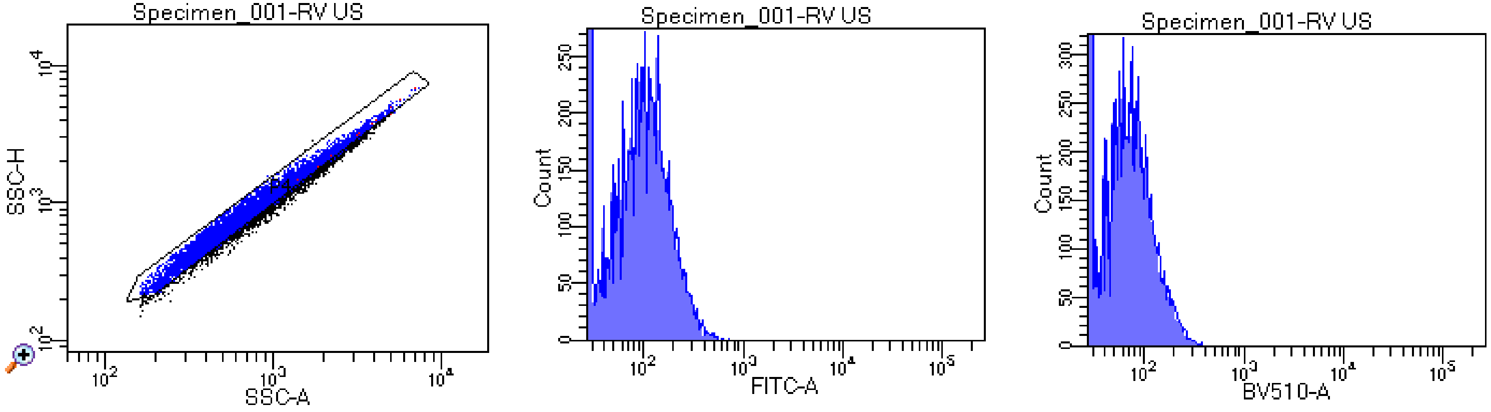

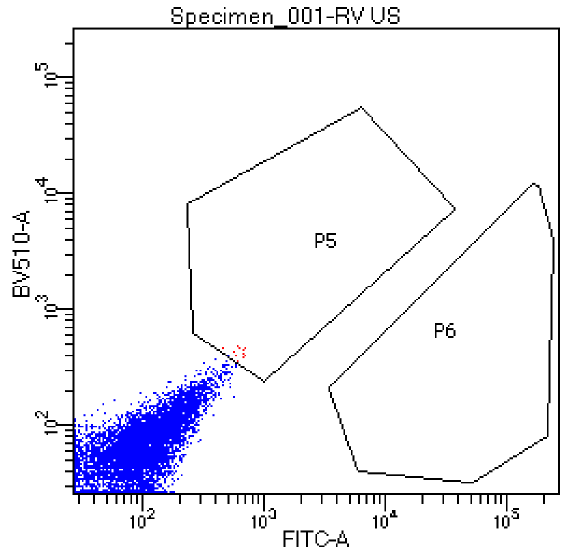

Experiment Name:	03Feb2016 Bact Sorting
Specimen Name:	Specimen_001
Tube Name:	RV US
Record Date:	Feb 3, 2016 3:19:31 PM
$OP:	Administrator
GUID:	5525787f-e853-42cc-836d-07a...

Population	#Events	%Parent	FITC-A Median	BV510-A Median
All Events	16,022	####	117	80
P1	12,827	80.1	102	71
P2	11,478	89.5	93	65
P3	11,003	95.9	94	67
P4	9,432	85.7	89	62
P5	12	0.1	602	420
P6	0	0.0	####	####

Tube: RV US

Population	#Events	%Parent	%Total
All Events	16,022	####	100.0
P1	12,827	80.1	80.1
P2	11,478	89.5	71.6
P3	11,003	95.9	68.7
P4	9,432	85.7	58.9
P5	12	0.1	0.1
P6	0	0.0	0.0

BD FACSDiva 8.0.1

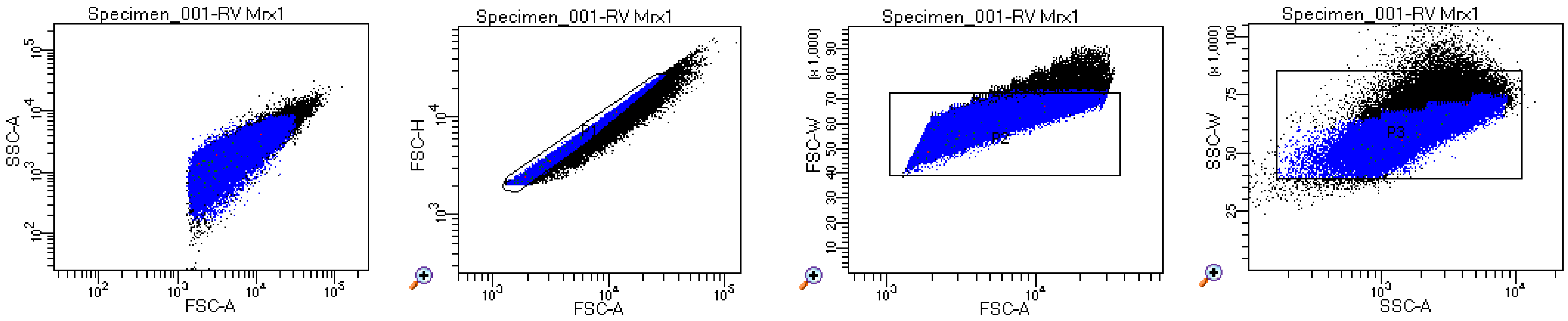

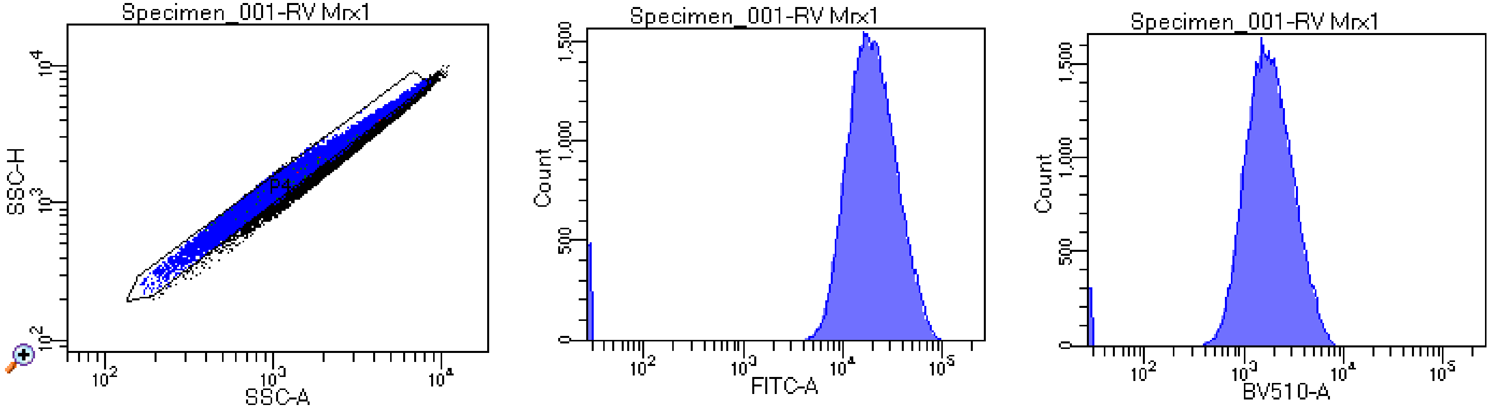

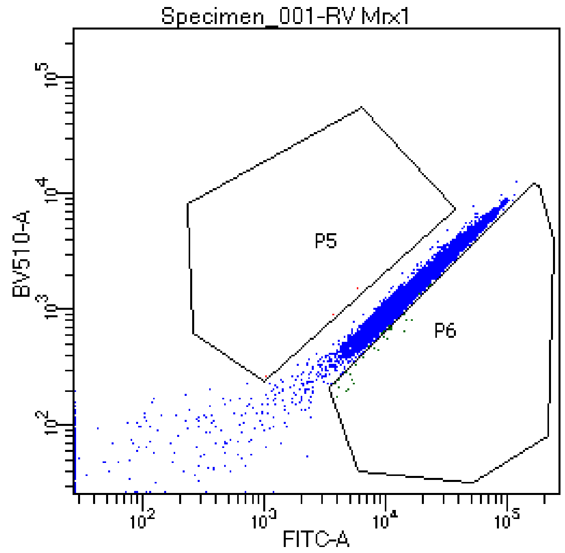

Experiment Name:	03Feb2016 Bact Sorting
Specimen Name:	Specimen_001
Tube Name:	RV Mrx1
Record Date:	Feb 3, 2016 3:20:46 PM
$OP:	Administrator
GUID:	b862cdae-f1c2-437a-b30a-f205...

Population	#Events	%Parent	FITC-A Median	BV510-A Median
All Events	96,347	####	23,308	2,089
P1	88,561	91.9	22,119	1,986
P2	82,624	93.3	20,954	1,885
P3	81,034	98.1	20,874	1,878
P4	60,439	74.6	18,193	1,645
P5	3	0.0	3,654	907
P6	38	0.1	9,589	606

Tube: RV Mrx1

Population	#Events	%Parent	%Total
All Events	96,347	####	100.0
P1	88,561	91.9	91.9
P2	82,624	93.3	85.8
P3	81,034	98.1	84.1
P4	60,439	74.6	62.7
P5	3	0.0	0.0
P6	38	0.1	0.0

BD FACSDiva 8.0.1

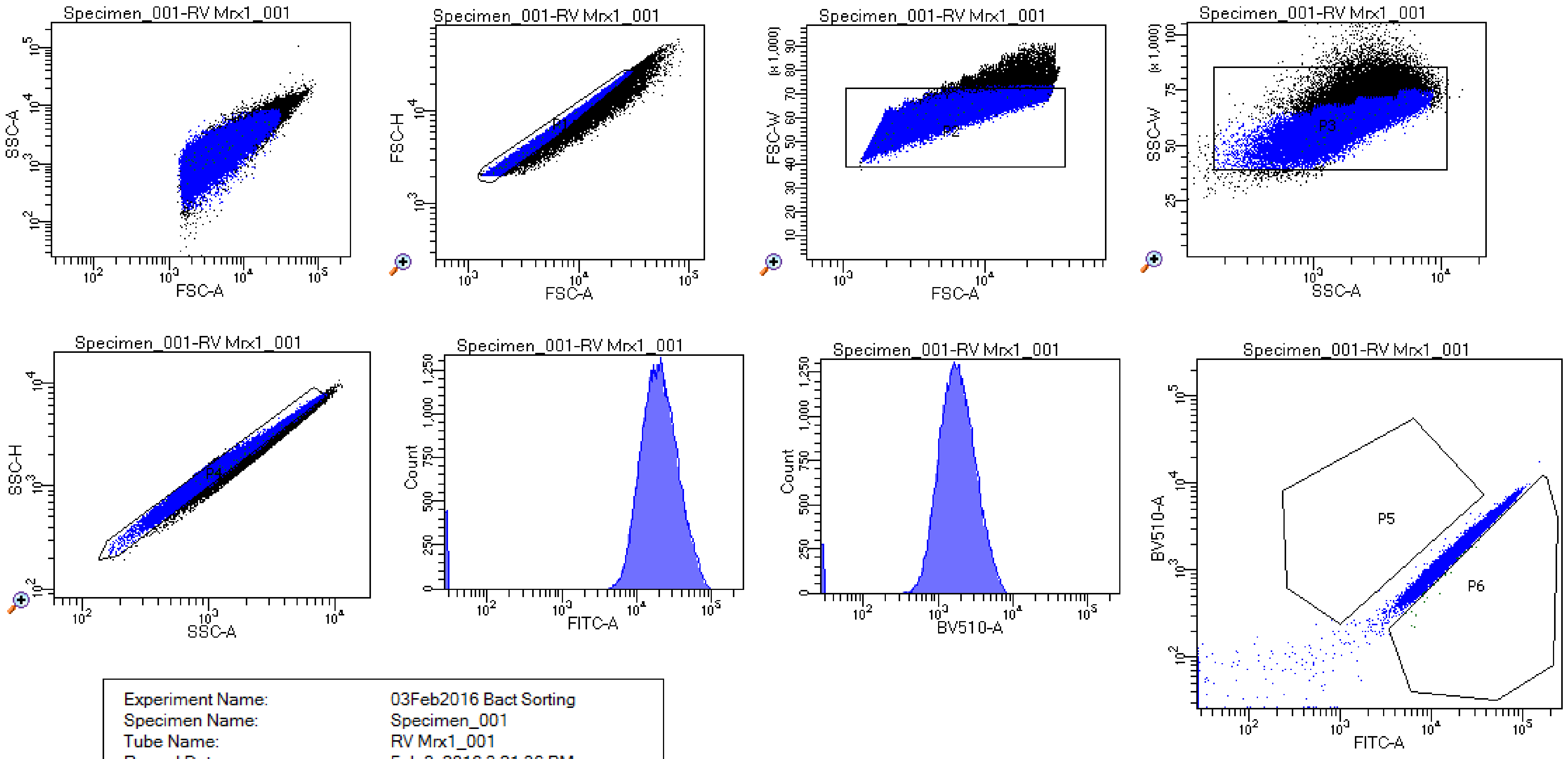

Experiment Name:	03Feb2016 Bact Sorting
Specimen Name:	Specimen_001
Tube Name:	RV Mrx1_001
Record Date:	Feb 3, 2016 3:21:36 PM
$OP:	Administrator
GUID:	f1bcc585-3b64-4776-aea3-f632...

Population	#Events	%Parent	FITC-A Median	BV510-A Median
All Events	82,747	####	24,266	2,140
P1	75,747	91.5	23,019	2,030
P2	70,080	92.5	21,611	1,911
P3	68,839	98.2	21,525	1,904
P4	50,336	73.1	18,656	1,653
P5	0	0.0	####	####
P6	39	0.1	14,244	922

Tube: RV Mrx1_001

Population	#Events	%Parent	%Total
All Events	82,747	####	100.0
P1	75,747	91.5	91.5
P2	70,080	92.5	84.7
P3	68,839	98.2	83.2
P4	50,336	73.1	60.8
P5	0	0.0	0.0
P6	39	0.1	0.0

BD FACSDiva 8.0.1

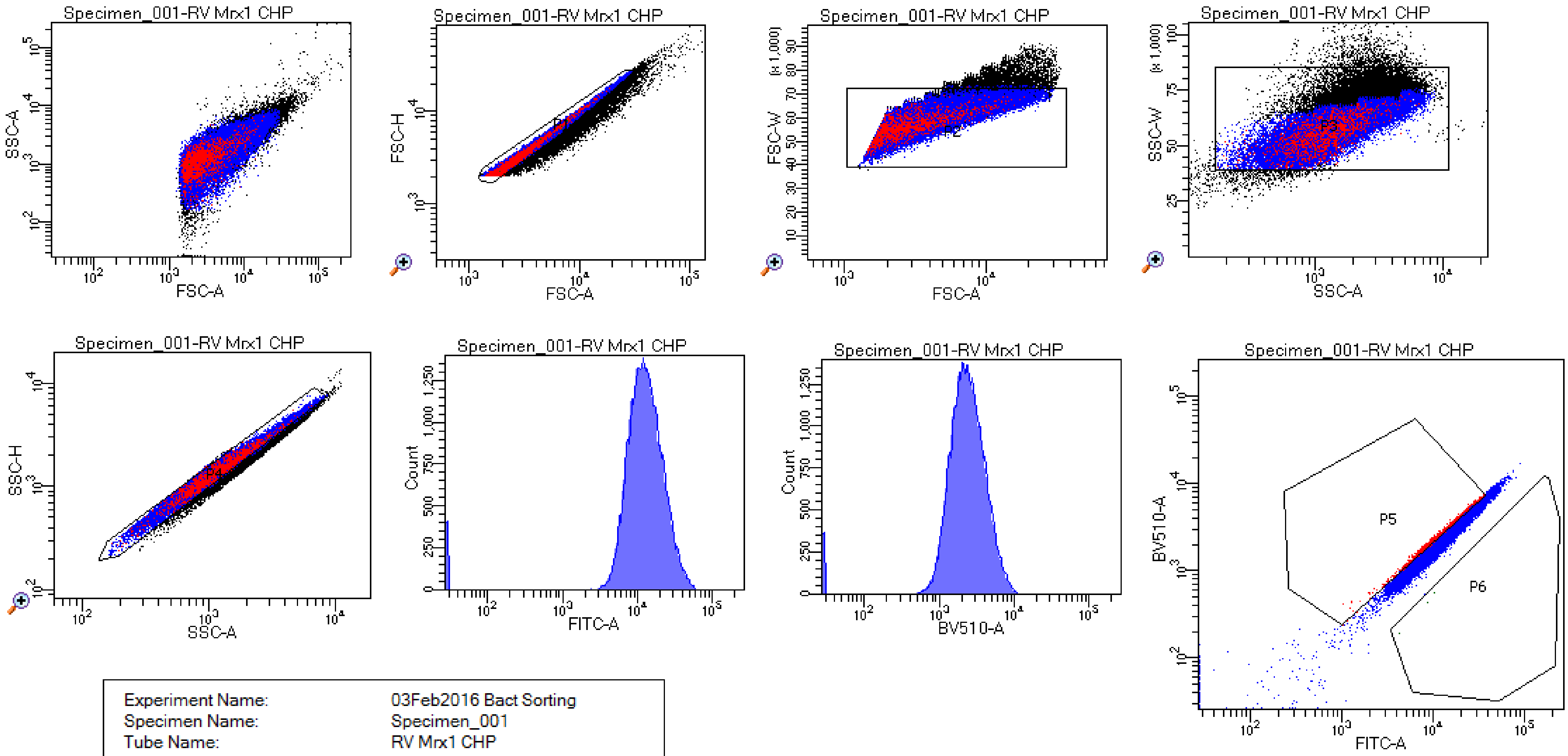

Experiment Name:	03Feb2016 Bact Sorting
Specimen Name:	Specimen_001
Tube Name:	RV Mrx1 CHP
Record Date:	Feb 3, 2016 3:22:42 PM
$OP:	Administrator
GUID:	14cc8366-2b97-4646-b979-0d6...

Population	#Events	%Parent	FITC-A Median	BV510-A Median
All Events	71,472	####	13,991	2,586
P1	66,031	92.4	13,383	2,479
P2	63,100	95.6	12,928	2,395
P3	61,635	97.7	12,950	2,399
P4	49,824	80.8	11,737	2,183
P5	1,416	2.8	10,805	2,366
P6	5	0.0	7,776	437

Tube: RV Mrx1 CHP

Population	#Events	%Parent	%Total
All Events	71,472	####	100.0
P1	66,031	92.4	92.4
P2	63,100	95.6	88.3
P3	61,635	97.7	86.2
P4	49,824	80.8	69.7
P5	1,416	2.8	2.0
P6	5	0.0	0.0

BD FACSDiva 8.0.1

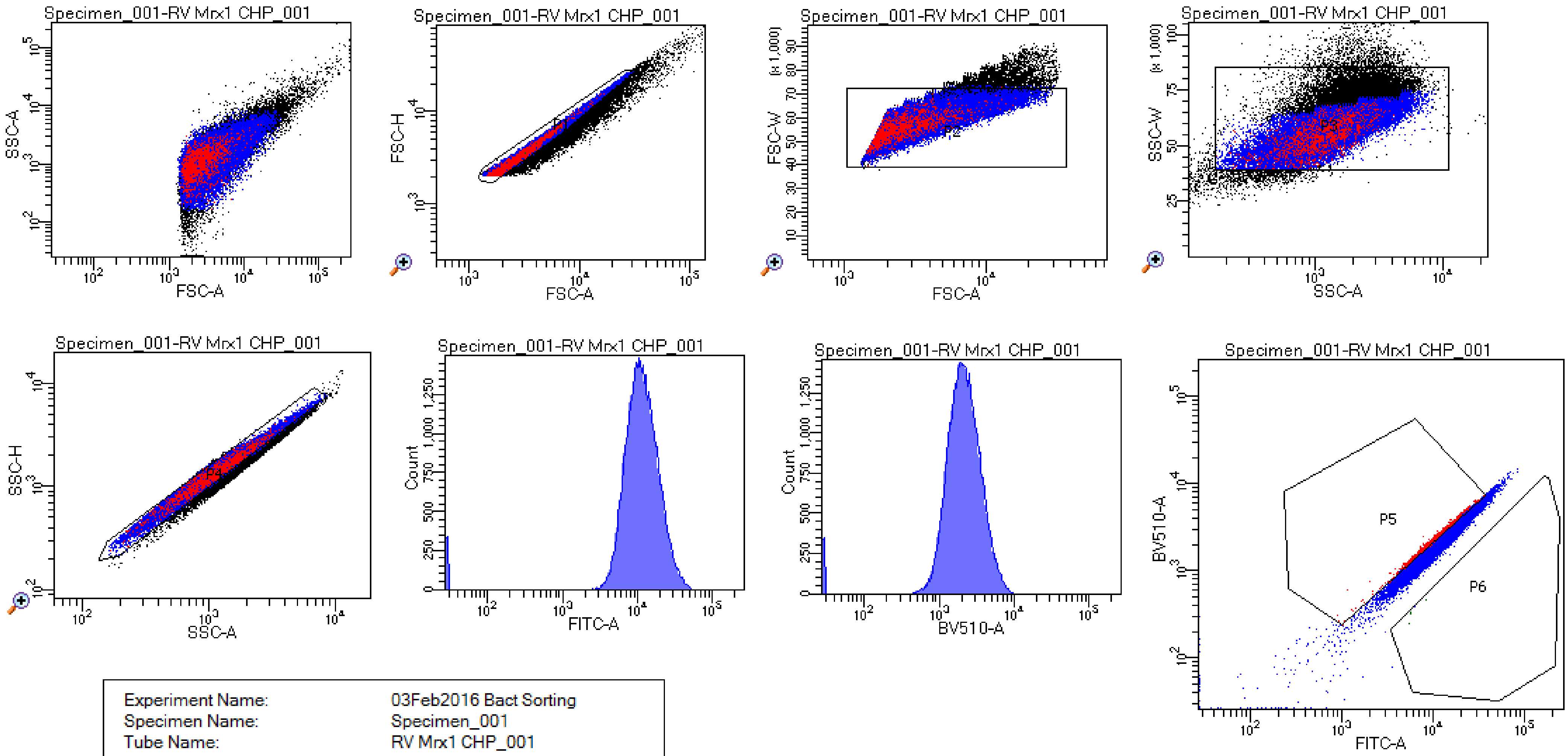

Experiment Name:	03Feb2016 Bact Sorting
Specimen Name:	Specimen_001
Tube Name:	RV Mrx1 CHP_001
Record Date:	Feb 3, 2016 3:23:34 PM
$OP:	Administrator
GUID:	91a05edf-ffbc-4741-b4dd-10f7b...

Population	#Events	%Parent	FITC-A Median	BV510-A Median
All Events	70,694	####	12,270	2,271
P1	64,612	91.4	11,680	2,162
P2	62,245	96.3	11,370	2,108
P3	60,124	96.6	11,447	2,120
P4	49,767	82.8	10,562	1,965
P5	1,399	2.8	10,233	2,261
P6	4	0.0	5,552	323

Tube: RV Mrx1 CHP_001

Population	#Events	%Parent	%Total
All Events	70,694	####	100.0
P1	64,612	91.4	91.4
P2	62,245	96.3	88.0
P3	60,124	96.6	85.0
P4	49,767	82.8	70.4
P5	1,399	2.8	2.0
P6	4	0.0	0.0

BD FACSDiva 8.0.1

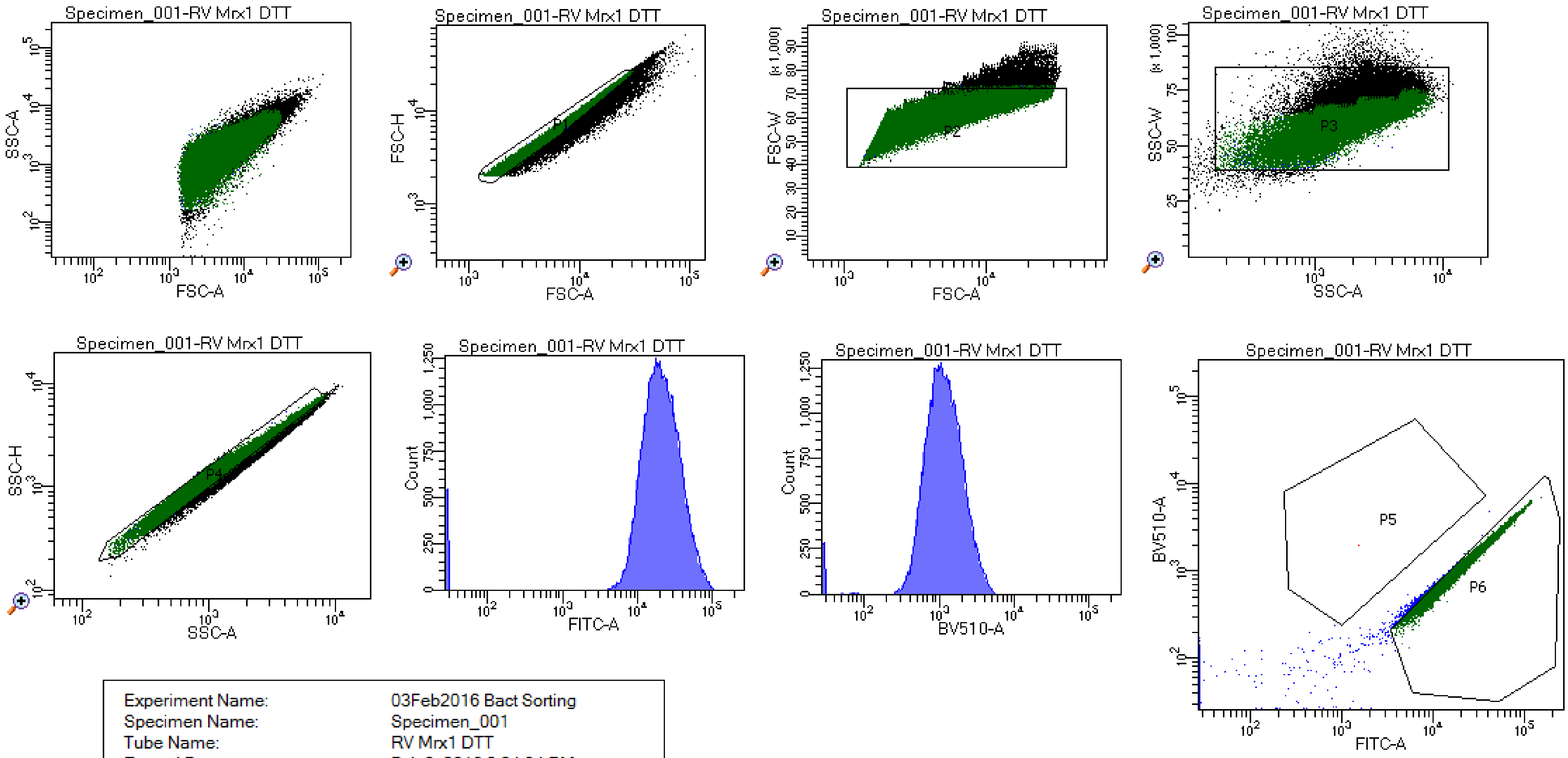

Experiment Name:	03Feb2016 Bact Sorting
Specimen Name:	Specimen_001
Tube Name:	RV Mrx1 DTT
Record Date:	Feb 3, 2016 3:24:34 PM
$OP:	Administrator
GUID:	efefc59e-5606-4206-bd4c-6326...

Population	#Events	%Parent	FITC-A Median	BV510-A Median
All Events	80,498	####	24,715	1,344
P1	73,100	90.8	23,401	1,276
P2	67,922	92.9	22,035	1,202
P3	66,746	98.3	22,033	1,202
P4	50,000	74.9	19,122	1,049
P5	1	0.0	1,470	2,000
P6	48,807	97.6	19,455	1,066

Tube: RV Mrx1 DTT

Population	#Events	%Parent	%Total
All Events	80,498	####	100.0
P1	73,100	90.8	90.8
P2	67,922	92.9	84.4
P3	66,746	98.3	82.9
P4	50,000	74.9	62.1
P5	1	0.0	0.0
P6	48,807	97.6	60.6

BD FACSDiva 8.0.1

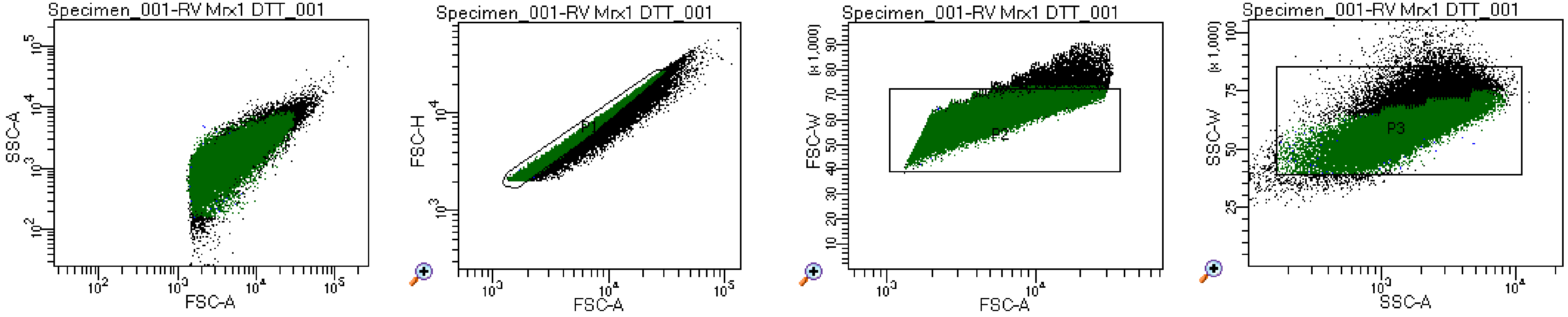

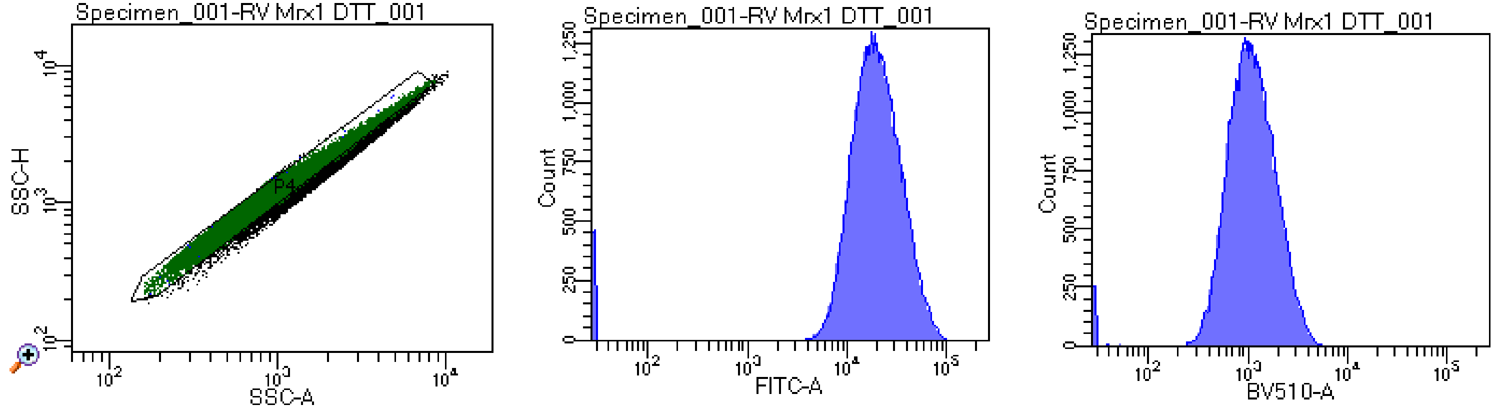

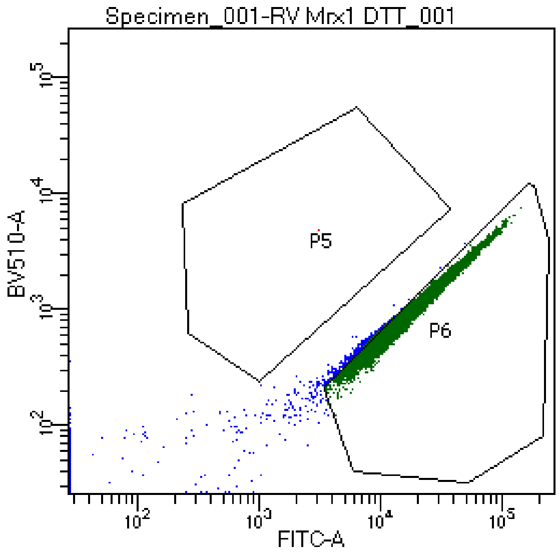

Experiment Name:	03Feb2016 Bact Sorting
Specimen Name:	Specimen_001
Tube Name:	RV Mrx1 DTT_001
Record Date:	Feb 3, 2016 3:25:47 PM
$OP:	Administrator
GUID:	33b9914e-eed8-4b67-9ae4-bf6f...

Population	#Events	%Parent	FITC-A Median	BV510-A Median
All Events	78,431	####	22,966	1,241
P1	71,141	90.7	21,806	1,180
P2	66,735	93.8	20,758	1,123
P3	65,384	98.0	20,767	1,124
P4	50,000	76.5	18,260	992
P5	1	0.0	3,078	4,761
P6	48,870	97.7	18,570	1,008

Tube: RV Mrx1 DTT_001

Population	#Events	%Parent	%Total
All Events	78,431	####	100.0
P1	71,141	90.7	90.7
P2	66,735	93.8	85.1
P3	65,384	98.0	83.4
P4	50,000	76.5	63.8
P5	1	0.0	0.0
P6	48,870	97.7	62.3

BD FACSDiva 8.0.1

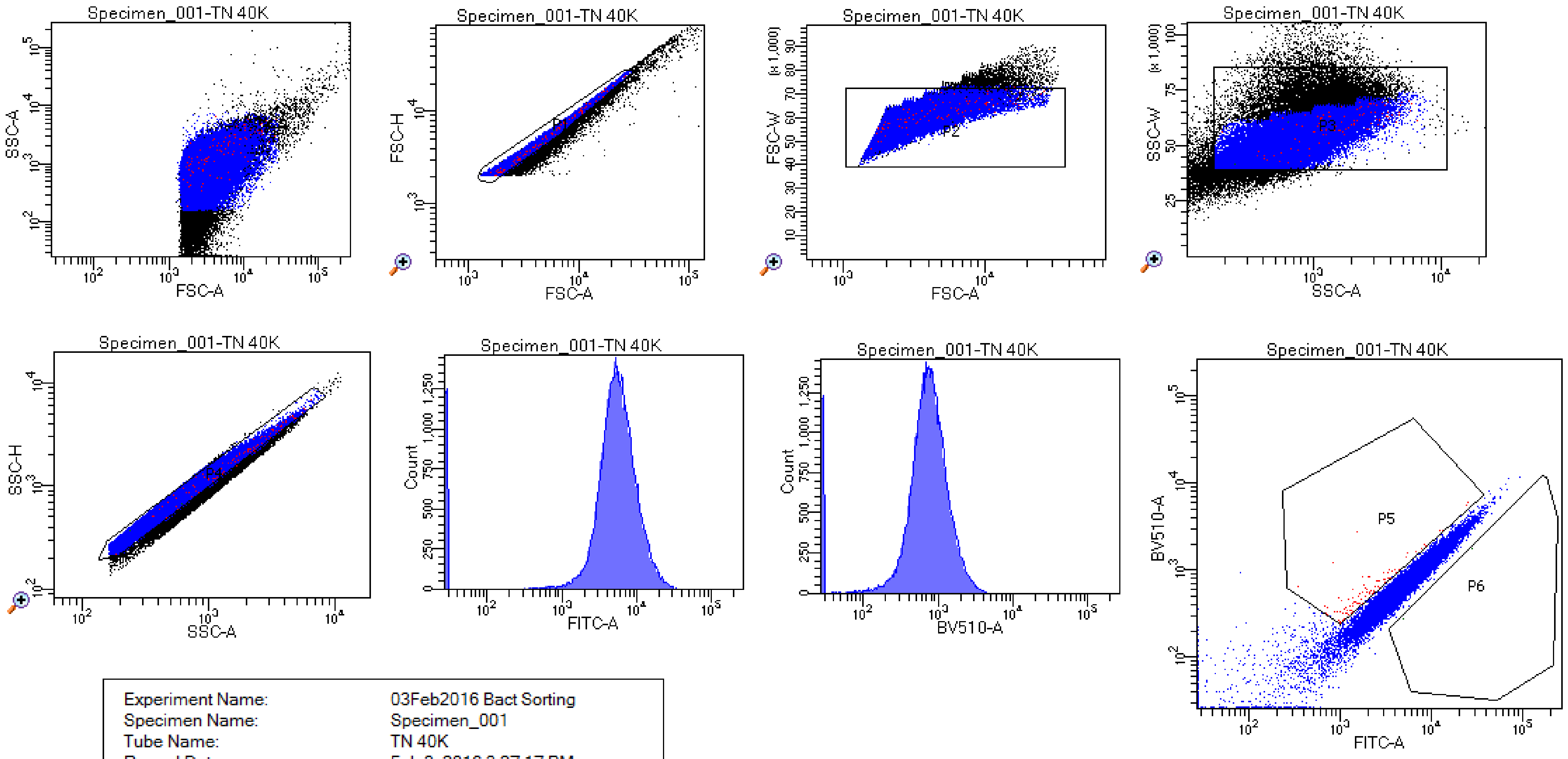

Experiment Name:	03Feb2016 Bact Sorting
Specimen Name:	Specimen_001
Tube Name:	TN 40K
Record Date:	Feb 3, 2016 3:27:17 PM
$OP:	Administrator
GUID:	5afa2684-77fb-42ab-b268-9cf9...

Population	#Events	%Parent	FITC-A Median	BV510-A Median
All Events	77,609	####	5,428	738
P1	70,083	90.3	5,246	713
P2	68,458	97.7	5,167	702
P3	59,505	86.9	5,370	728
P4	50,000	84.0	5,090	692
P5	101	0.2	1,707	548
P6	2	0.0	16,306	1,009

Tube: TN 40K

Population	#Events	%Parent	%Total
All Events	77,609	####	100.0
P1	70,083	90.3	90.3
P2	68,458	97.7	88.2
P3	59,505	86.9	76.7
P4	50,000	84.0	64.4
P5	101	0.2	0.1
P6	2	0.0	0.0

BD FACSDiva 8.0.1

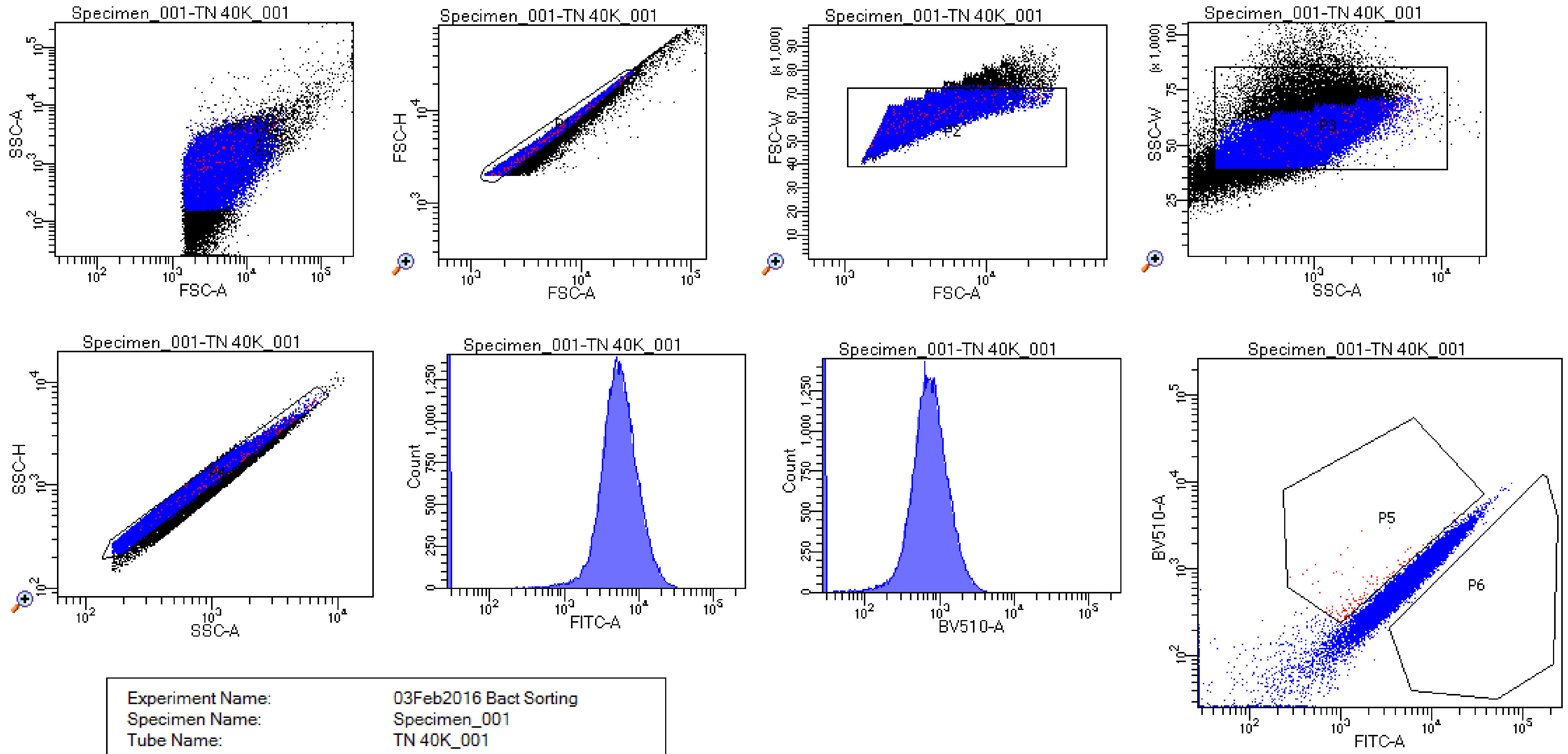

Experiment Name:	03Feb2016 Bact Sorting
Specimen Name:	Specimen_001
Tube Name:	TN 40K_001
Record Date:	Feb 3, 2016 3:30:57 PM
$OP:	Administrator
GUID:	1b423a43-4da6-47ea-9734-9f4...

Population	#Events	%Parent	FITC-A Median	BV510-A Median
All Events	80,849	####	5,326	720
P1	71,890	88.9	5,134	694
P2	70,155	97.6	5,050	682
P3	60,214	85.8	5,260	709
P4	50,000	83.0	4,966	670
P5	135	0.3	1,379	490
P6	0	0.0	####	####

Tube: TN 40K_001

Population	#Events	%Parent	%Total
All Events	80,849	####	100.0
P1	71,890	88.9	88.9
P2	70,155	97.6	86.8
P3	60,214	85.8	74.5
P4	50,000	83.0	61.8
P5	135	0.3	0.2
P6	0	0.0	0.0

BD FACSDiva 8.0.1

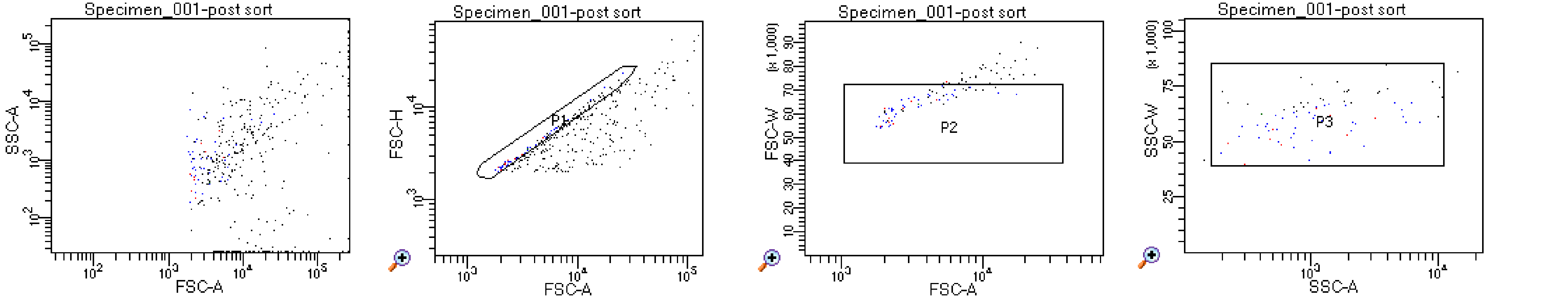

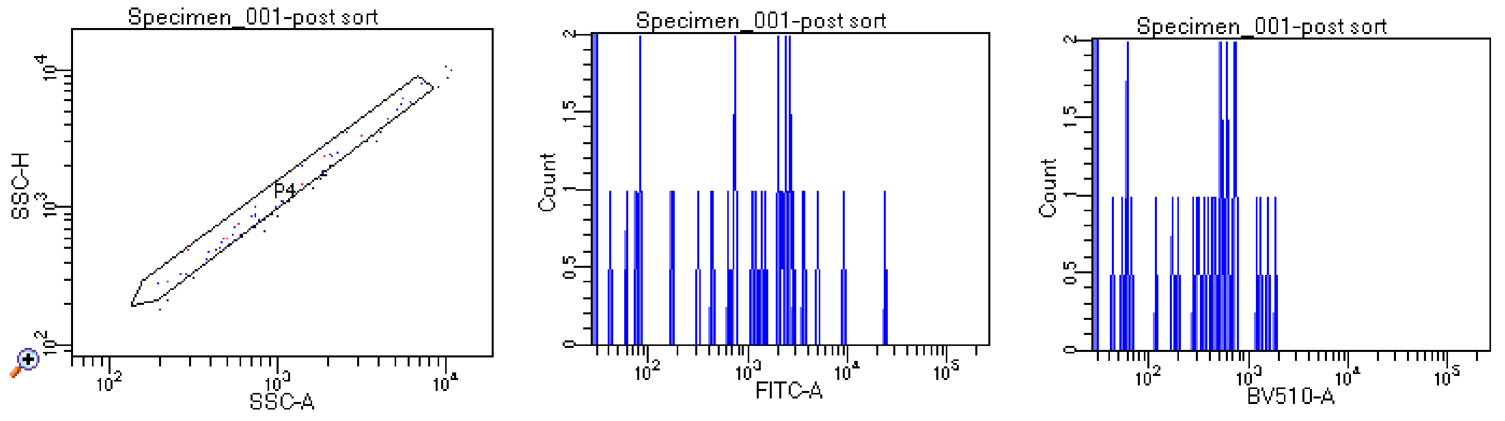

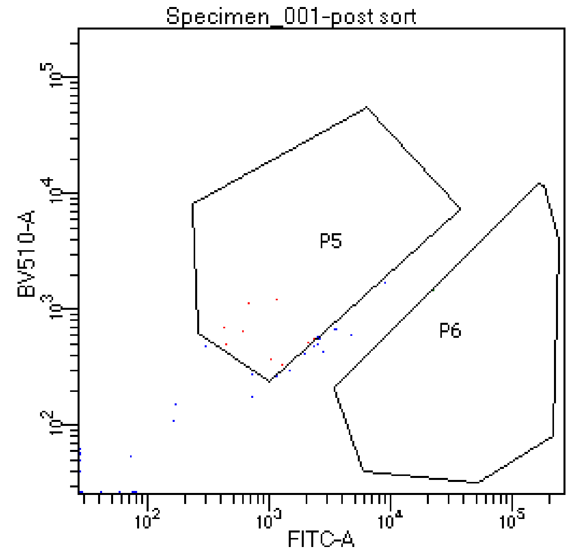

Experiment Name:	03Feb2016 Bact Sorting
Specimen Name:	Specimen_001
Tube Name:	post sort
Record Date:	Feb 3, 2016 5:14:37 PM
$OP:	Administrator
GUID:	62a38b5b-4eb5-4a77-b5e4-42f...

Population	#Events	%Parent	FITC-A Median	BV510-A Median
All Events	366	####	78	66
P1	107	29.2	897	335
P2	80	74.8	696	276
P3	77	96.3	809	286
P4	50	64.9	347	221
P5	9	18.0	1,016	558
P6	1	2.0	22,699	1,465

Tube: post sort

Population	#Events	%Parent	%Total
All Events	366	####	100.0
P1	107	29.2	29.2
P2	80	74.8	21.9
P3	77	96.2	21.0
P4	50	64.9	13.7
P5	9	18.0	2.5
P6	1	2.0	0.3
