# Supplementary material for: Biosensor-integrated transposon mutagenesis reveals rv0158 as a coordinator of redox homeostasis in Mycobacterium tuberculosis
Source: eLife. 2023 Aug 29;12:e80218. doi: 10.7554/eLife.80218 (PMC10501769; doi:10.7554/eLife.80218)
Supplement: Figure 1—source data 1. [file elife-80218-fig1-data1.zip › Round 1 Sorting/16May2016 Bac sorting-Batch_Analysis_16052016113903.pdf]

Batch Analysis Report

Run Date: 5/16/16 11:39 AM
Experiment: 16May2016 Bac sorting
User ID: Administrator
Statistics Output: N/A
Worksheet PDF Output: C:\Users\Admin\Desktop\16May2016 Bac sorting-Batch_Analysis_16052016113903.pdf

Specimen_001

Tube	Status	Run Time
US	OK	5/16/16 11:39 AM
Mrx1	OK	5/16/16 11:39 AM
MRX1 1	OK	5/16/16 11:39 AM
TN40k	OK	5/16/16 11:39 AM

BD FACSDiva 8.0.1

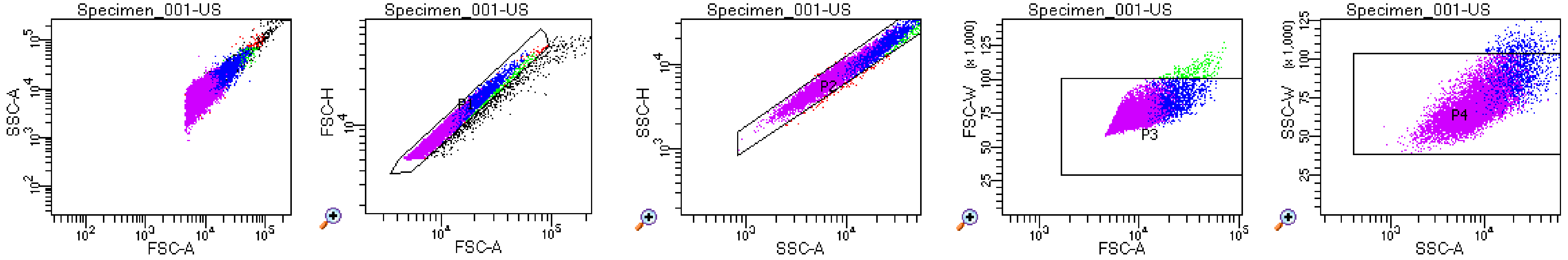

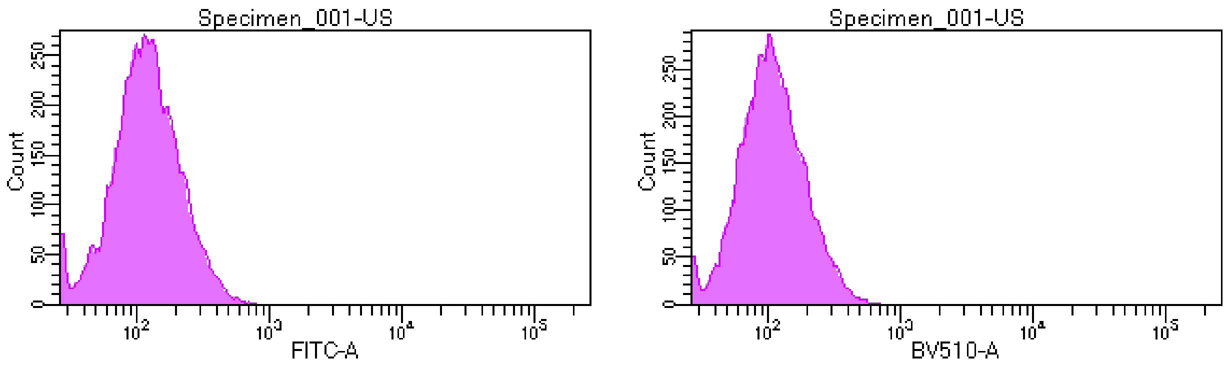

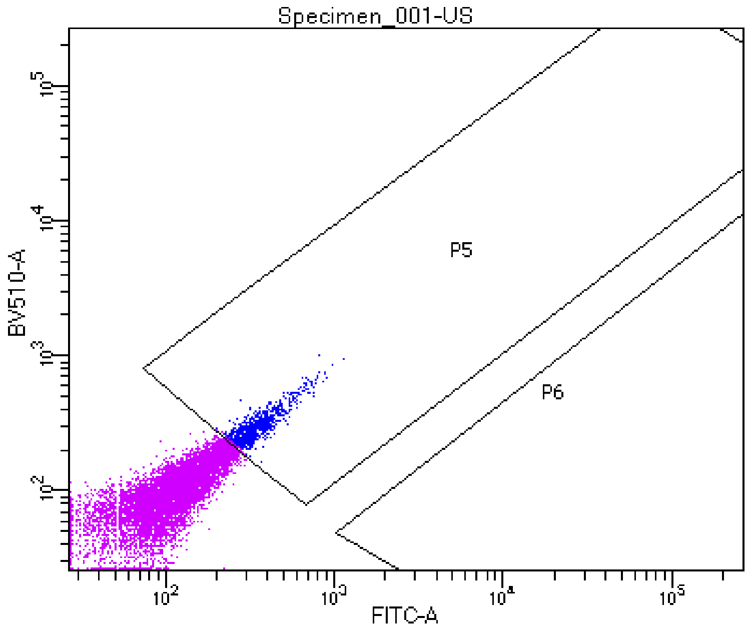

Tube: US

Population	#Events	%Parent	%Total
All Events	11,319	####	100.0
P1	10,720	94.7	94.7
P2	10,567	98.6	93.4
P3	10,362	98.1	91.5
P4	10,084	97.3	89.1
P5	849	8.4	7.5
P6	0	0.0	0.0

Experiment Name:	16May2016 Bac sorting
Specimen Name:	Specimen_001
Tube Name:	US
Record Date:	May 16, 2016 11:34:26 AM
$OP:	Administrator
GUID:	31fa8bf9-36c1-4ab7-9ee3-f1934c6e3670

Population	%Parent	%Grand Parent	%Total	FITC-A Median	BV510-A Median
P4	97.3	95.4	89.1	113	101
P5	8.4	8.2	7.5	319	285
P6	0.0	0.0	0.0	####	####

BD FACSDiva 8.0.1

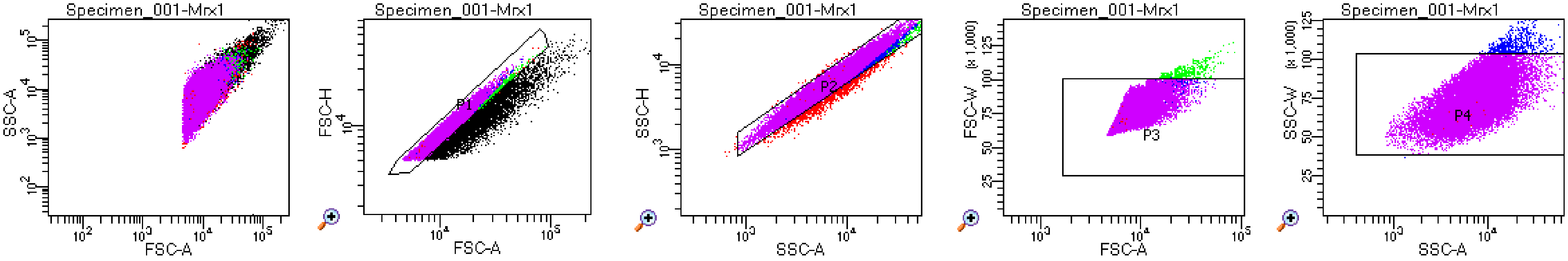

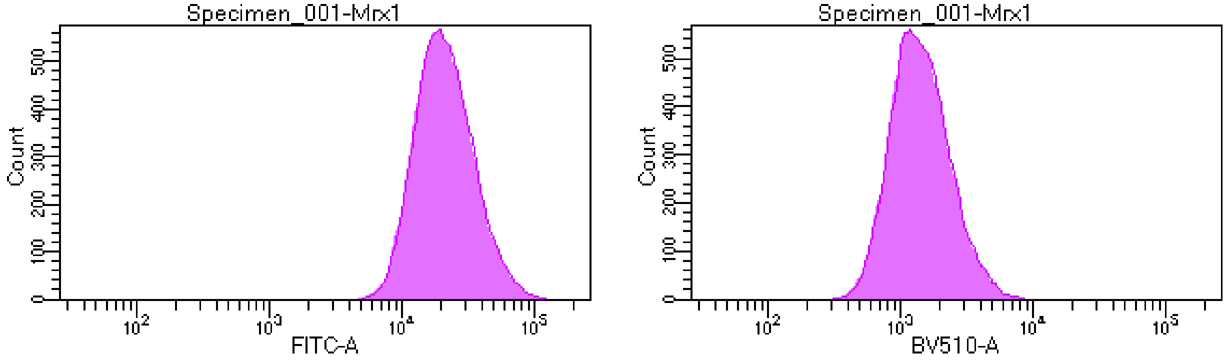

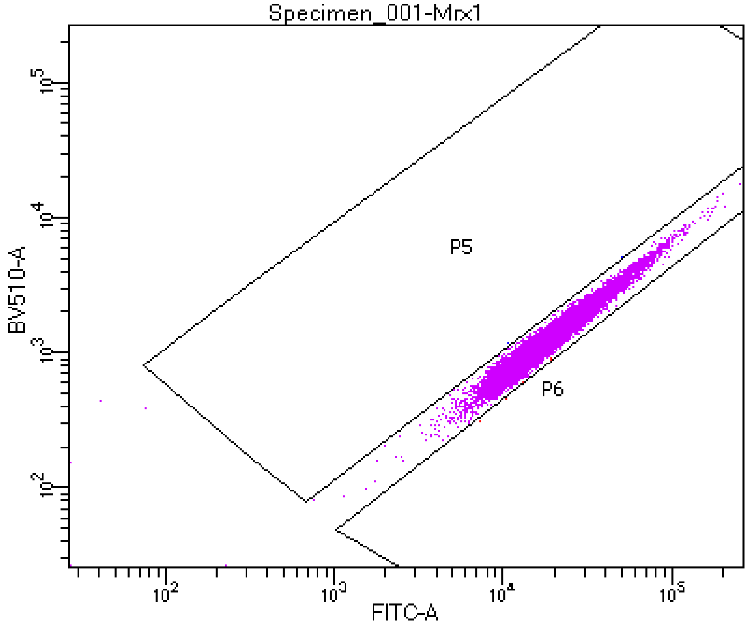

Tube: Mrx1

Population	#Events	%Parent	%Total
All Events	29,979	####	100.0
P1	22,554	75.2	75.2
P2	20,975	93.0	70.0
P3	20,582	98.1	68.7
P4	20,000	97.2	66.7
P5	2	0.0	0.0
P6	10	0.0	0.0

Experiment Name:	16May2016 Bac sorting
Specimen Name:	Specimen_001
Tube Name:	Mrx1
Record Date:	May 16, 2016 11:36:06 AM
$OP:	Administrator
GUID:	c2938a97-2f1b-413c-81aa-fcb2260d303f

Population	%Parent	%Grand Parent	%Total	FITC-A Median	BV510-A Median
P4	97.2	95.4	66.7	19,832	1,318
P5	0.0	0.0	0.0	30,632	3,191
P6	0.1	0.0	0.0	12,749	589

BD FACSDiva 8.0.1

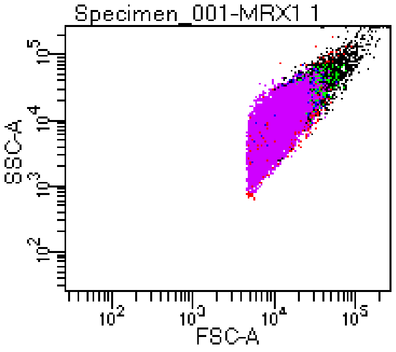

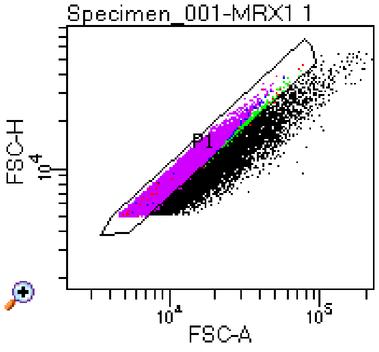

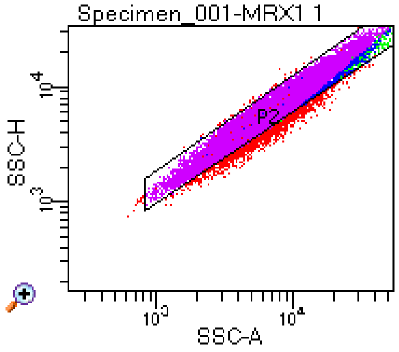

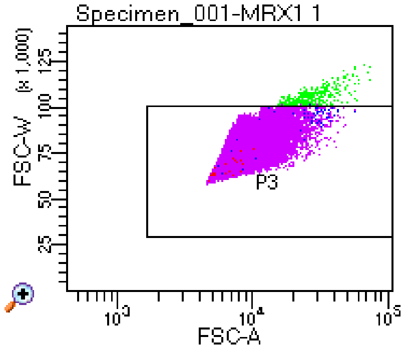

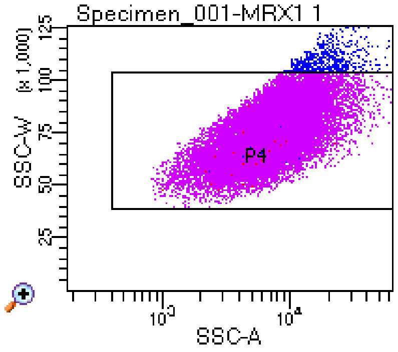

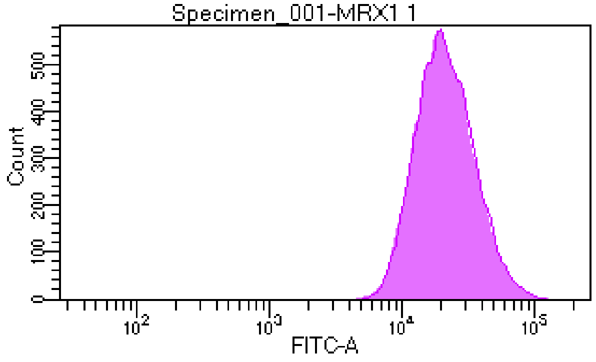

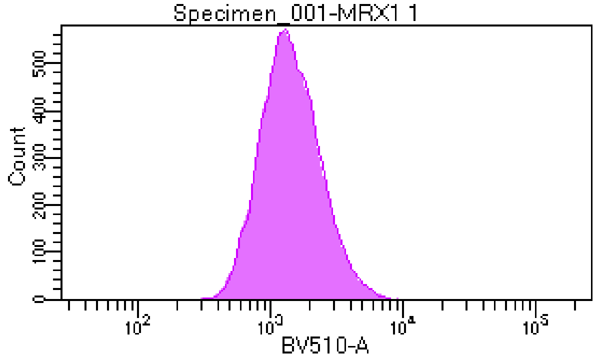

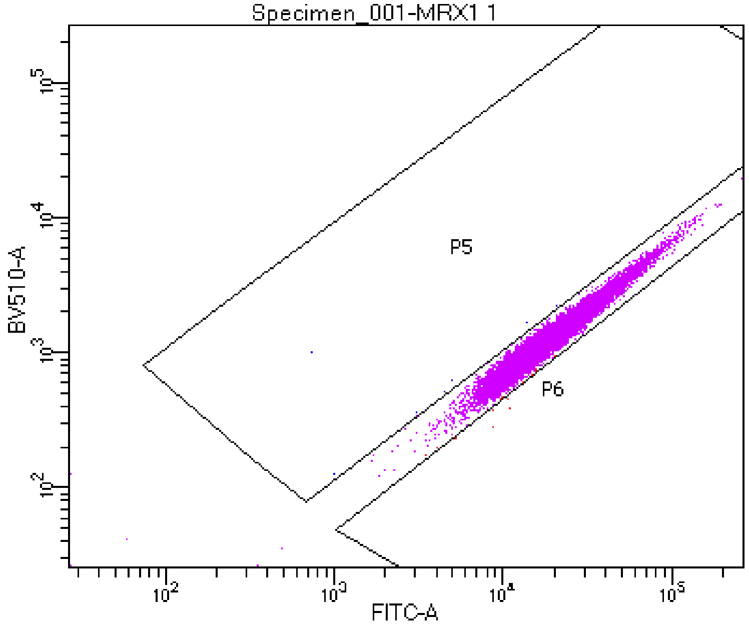

Tube: MRX1 1

Population	#Events	%Parent	%Total
All Events	30,011	####	100.0
P1	22,610	75.3	75.3
P2	20,948	92.6	69.8
P3	20,574	98.2	68.6
P4	20,000	97.2	66.6
P5	7	0.0	0.0
P6	20	0.1	0.1

Experiment Name:	16May2016 Bac sorting
Specimen Name:	Specimen_001
Tube Name:	MRX1 1
Record Date:	May 16, 2016 11:37:13 AM
$OP:	Administrator
GUID:	03e95433-38d1-4b4a-b1c5-1e10be582e34

Population	%Parent	%Grand Parent	%Total	FITC-A Median	BV510-A Median
P4	97.2	95.5	66.6	20,063	1,329
P5	0.0	0.0	0.0	4,406	620
P6	0.1	0.1	0.1	10,737	463

BD FACSDiva 8.0.1

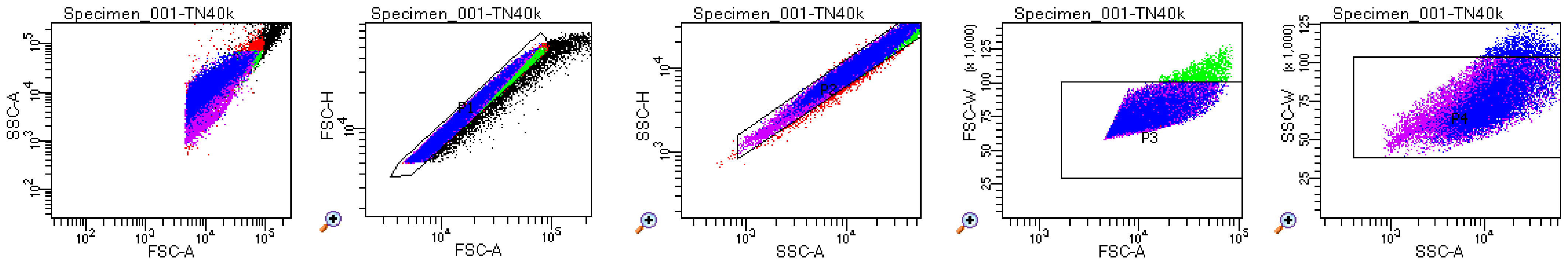

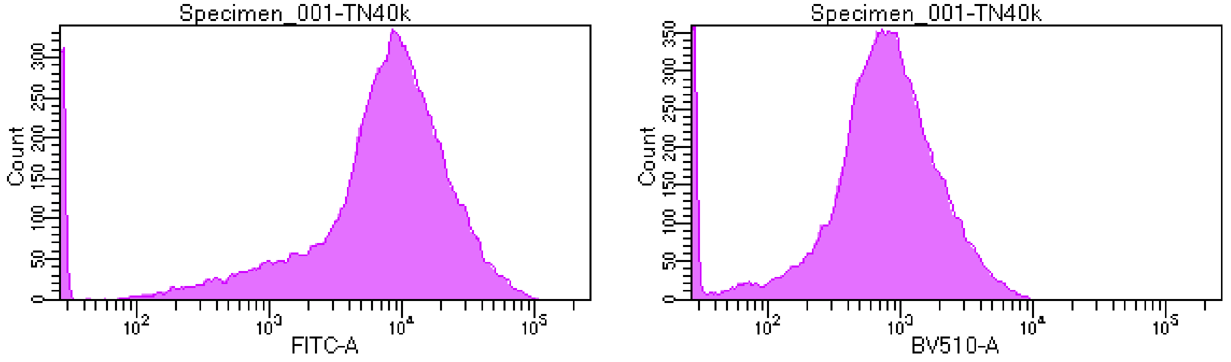

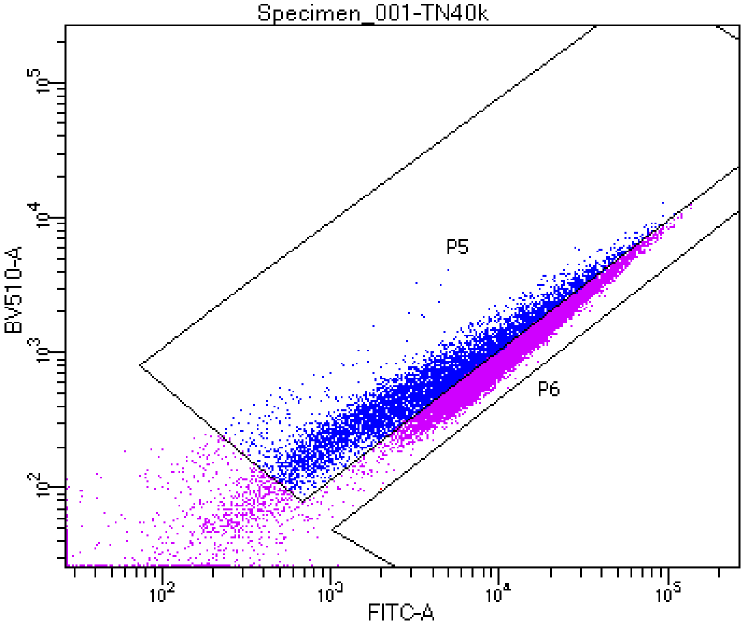

Tube: TN40k

Population	#Events	%Parent	%Total
All Events	25,979	####	100.0
P1	23,001	88.5	88.5
P2	21,984	95.6	84.6
P3	20,961	95.3	80.7
P4	20,000	95.4	77.0
P5	6,089	30.4	23.4
P6	1	0.0	0.0

Experiment Name: 16May2016 Bac sorting
Specimen Name: Specimen_001
Tube Name: TN40k
Record Date: May 16, 2016 11:38:47 AM
$OP: Administrator
GUID: 0ddd6a75-c0ad-41aa-82c7-2522e1159d9c

Population	%Parent	%Grand Parent	%Total	FITC-A Median	BV510-A Median
P4	95.4	91.0	77.0	7,733	726
P5	30.4	29.0	23.4	3,846	587
P6	0.0	0.0	0.0	1,952	97
