# Supplementary material for: Biosensor-integrated transposon mutagenesis reveals rv0158 as a coordinator of redox homeostasis in Mycobacterium tuberculosis
Source: eLife. 2023 Aug 29;12:e80218. doi: 10.7554/eLife.80218 (PMC10501769; doi:10.7554/eLife.80218)
Supplement: Figure 1—source data 1. [file elife-80218-fig1-data1.zip › Round 1 Sorting/21Nov2016 Bac sorting-Batch_Analysis_22112016164451.pdf]

Batch Analysis Report

Run Date: 11/22/16 4:45 PM Experiment: 21Nov2016 Bac sorting
User ID: Administrator
Statistics Output: C:\Users\Admin\Desktop\21Nov2016 Bac sorting-Batch_Analysis_22112016164451.csv
Worksheet PDF Output: C:\Users\Admin\Desktop\21Nov2016 Bac sorting-Batch_Analysis_22112016164451.pdf

21Nov

Tube	Status	Run Time
US	OK	11/22/16 4:45 PM
RV Mrx1	OK	11/22/16 4:45 PM
RV Mrx1_001	OK	11/22/16 4:45 PM
CHP	OK	11/22/16 4:45 PM
CHP_001	OK	11/22/16 4:45 PM
DTT	OK	11/22/16 4:45 PM
DTT_001	OK	11/22/16 4:45 PM
TN lib	OK	11/22/16 4:45 PM
Ox Post Sort	OK	11/22/16 4:45 PM
Red Post Sort	OK	11/22/16 4:45 PM

22Nov

Tube	Status	Run Time
US	OK	11/22/16 4:45 PM
RV Mrx1	OK	11/22/16 4:45 PM
RV Mrx1_001	OK	11/22/16 4:45 PM
CHP	OK	11/22/16 4:45 PM
CHP_001	OK	11/22/16 4:45 PM
DTT	OK	11/22/16 4:45 PM
DTT_001	OK	11/22/16 4:45 PM
TN lib	OK	11/22/16 4:45 PM
Ox Post Sort	OK	11/22/16 4:45 PM
Red Post Sort	OK	11/22/16 4:45 PM

BD FACSDiva 8.0.1

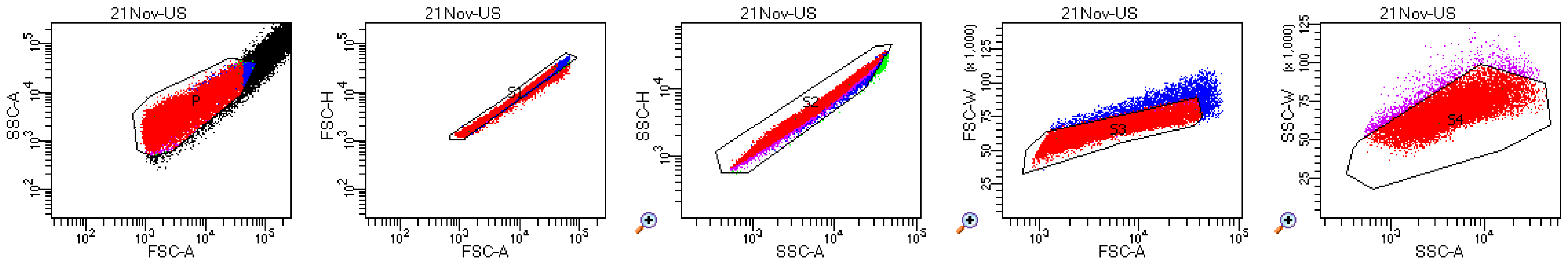

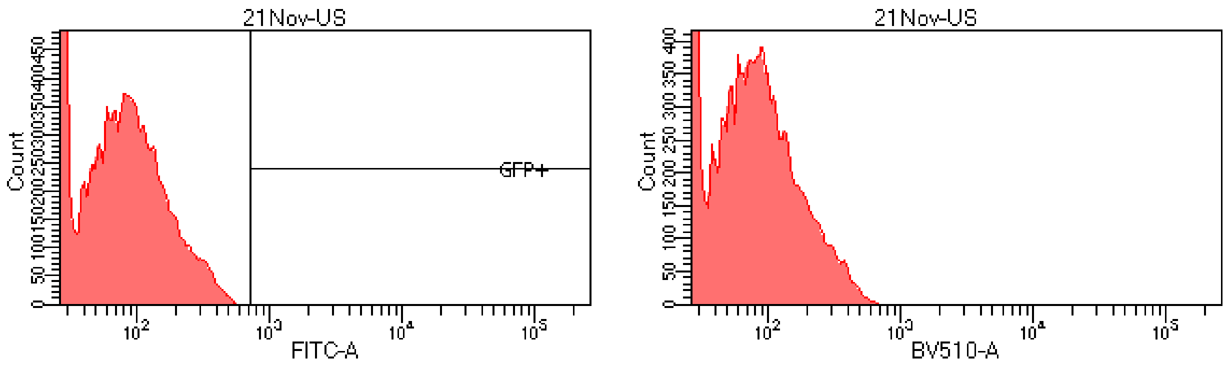

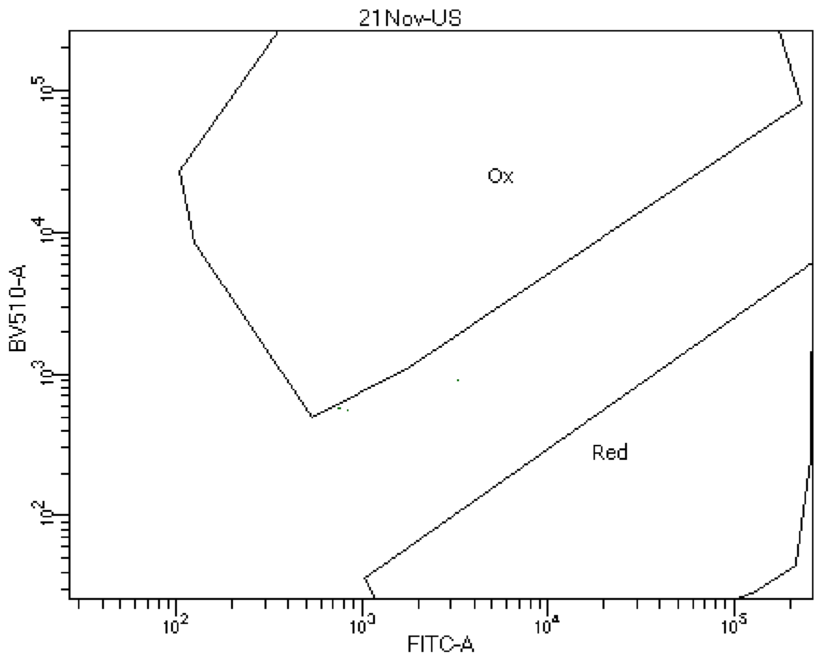

Tube: US

Population	#Events	%Parent	%Total
All Events	37,526	####	100.0
P	26,166	69.7	69.7
S1	25,129	96.0	67.0
S2	24,738	98.4	65.9
S3	22,218	89.8	59.2
S4	21,228	95.5	56.6
GFP+	4	0.0	0.0
Ox	0	0.0	0.0
Red	0	0.0	0.0

Experiment Name: 21Nov2016 Bac sorting
Specimen Name: 21Nov
Tube Name: US
Record Date: Nov 21, 2016 2:26:52 PM
$OP: Administrator
GUID: 2e772c01-6191-4b81-8928-543...

Population	#Events	%Parent	FITC-A Median	BV510-A Median
S4	21,228	95.5	65	67
GFP+	4	0.0	781	572
Ox	0	0.0	####	####
Red	0	0.0	####	####

BD FACSDiva 8.0.1

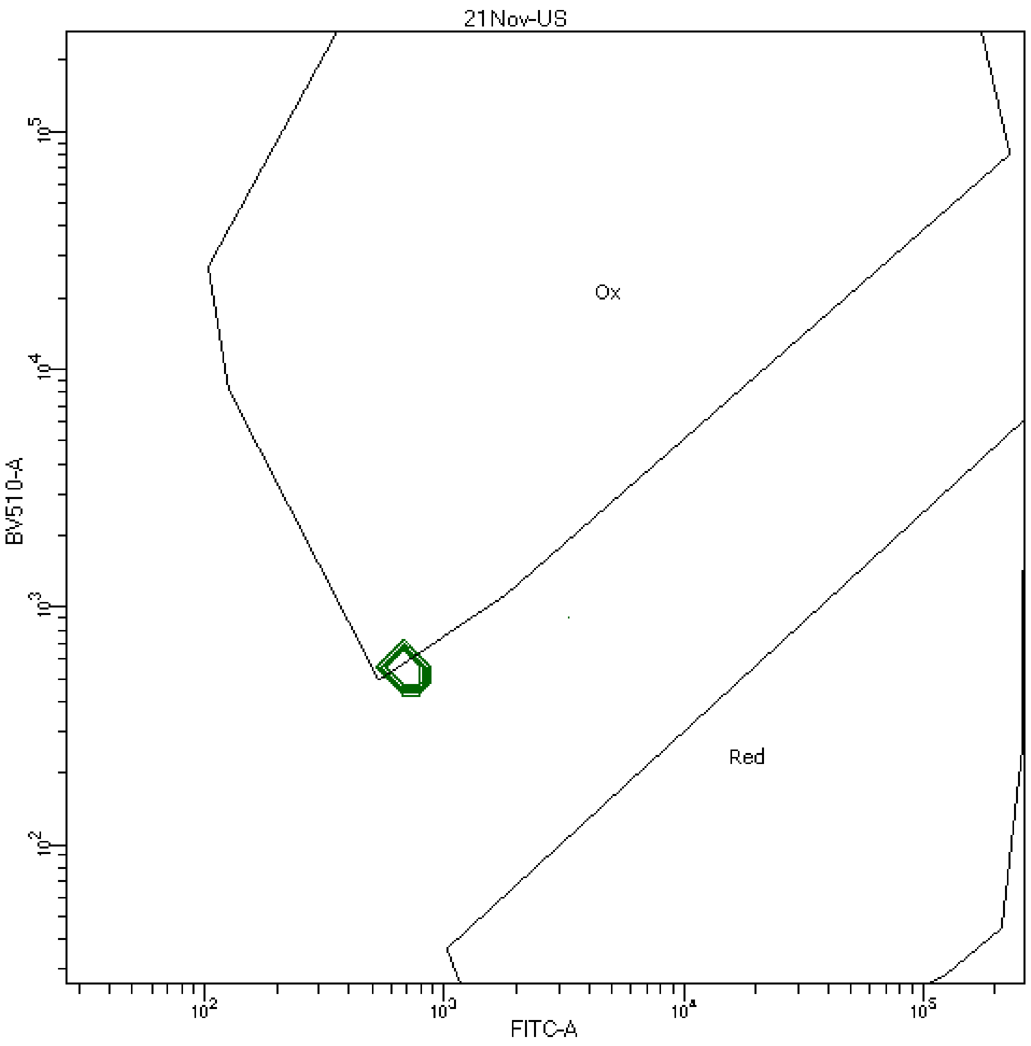

BD FACSDiva 8.0.1

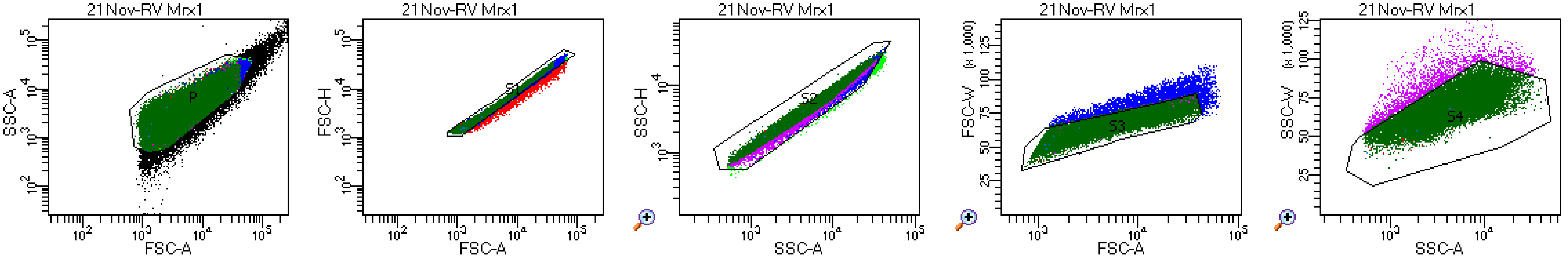

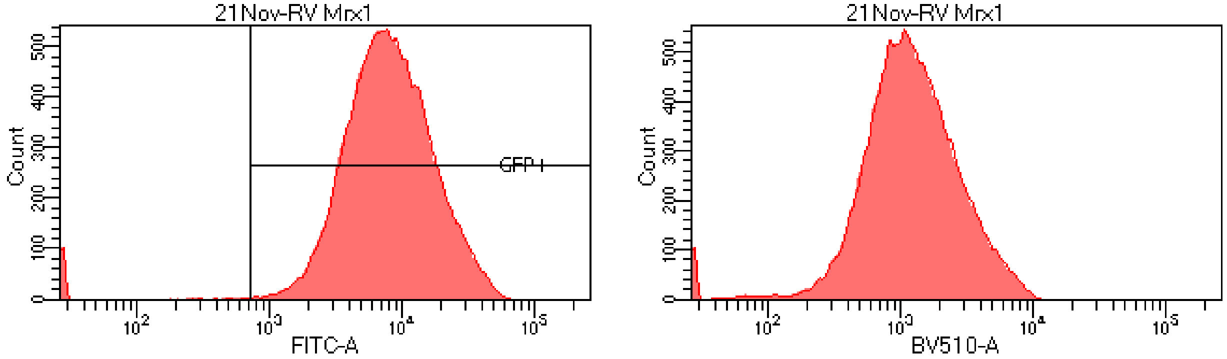

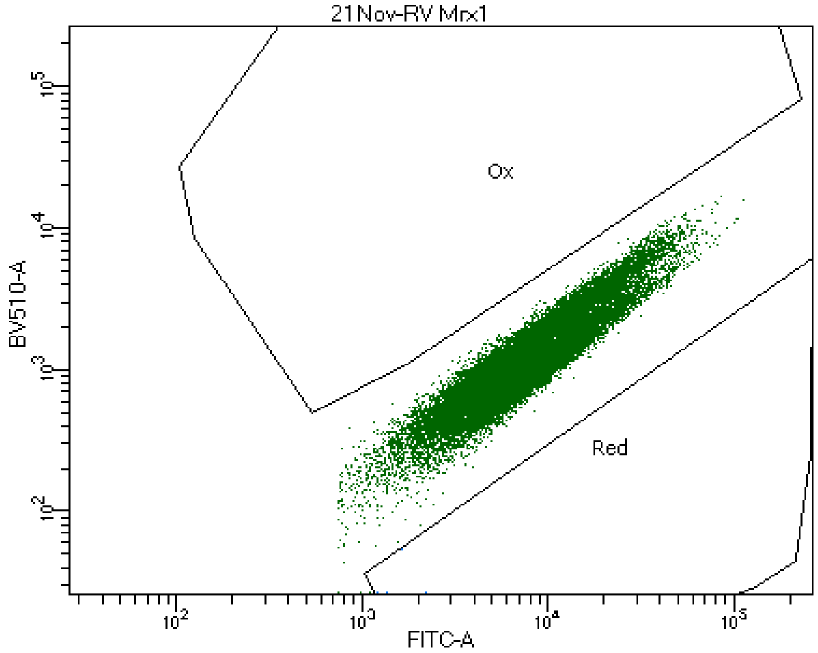

Tube: RV Mrx1

Population	#Events	%Parent	%Total
All Events	40,000	####	100.0
P	35,658	89.1	89.1
S1	33,806	94.8	84.5
S2	33,457	99.0	83.6
S3	30,000	89.7	75.0
S4	28,104	93.7	70.3
GFP+	27,485	97.8	68.7
Ox	0	0.0	0.0
Red	5	0.0	0.0

Experiment Name: 21Nov2016 Bac sorting
Specimen Name: 21Nov
Tube Name: RV Mrx1
Record Date: Nov 21, 2016 2:29:46 PM
$OP: Administrator
GUID: 3639ef2b-e674-43d8-b7d0-9e5...

Population	#Events	%Parent	FITC-A Median	BV510-A Median
S4	28,104	93.7	7,486	1,116
GFP+	27,485	97.8	7,650	1,140
Ox	0	0.0	####	####
Red	5	0.0	1,321	22

BD FACSDiva 8.0.1

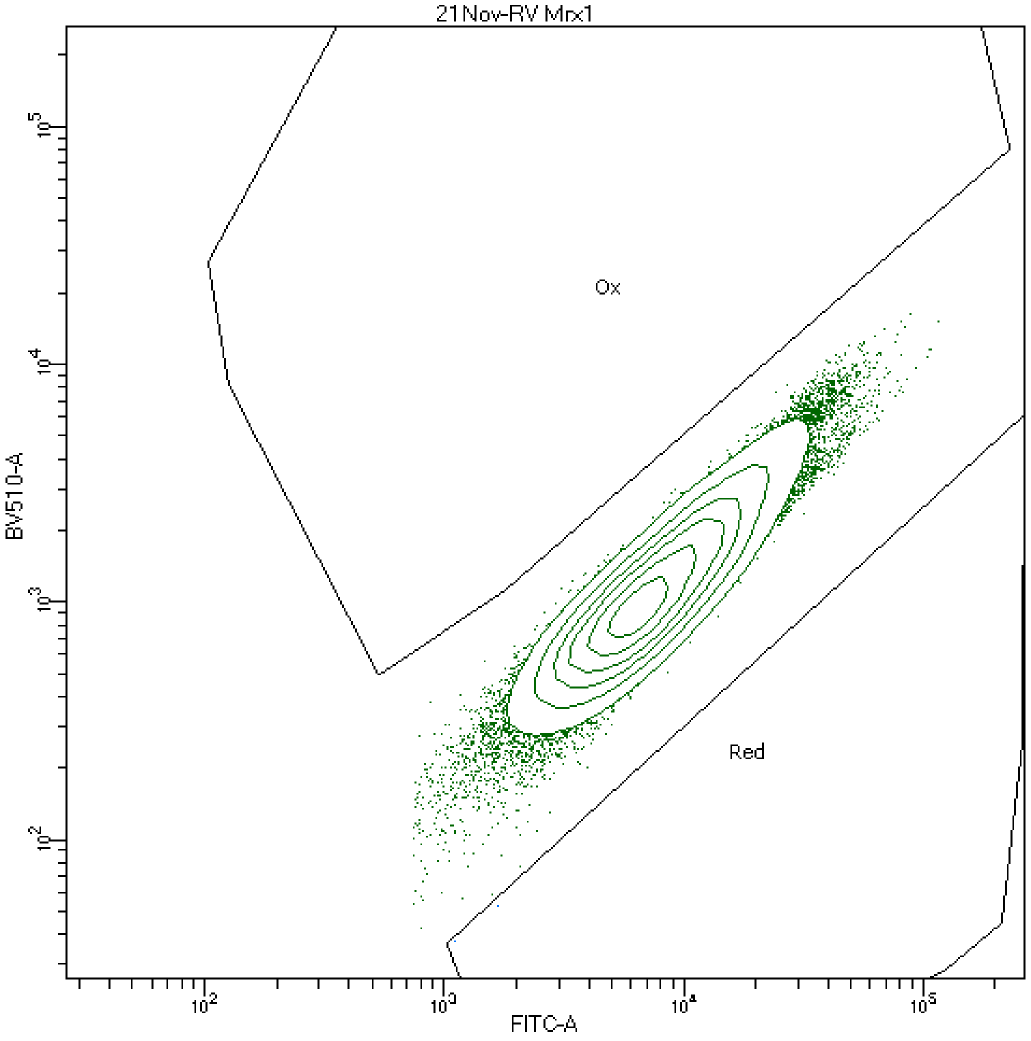

BD FACSDiva 8.0.1

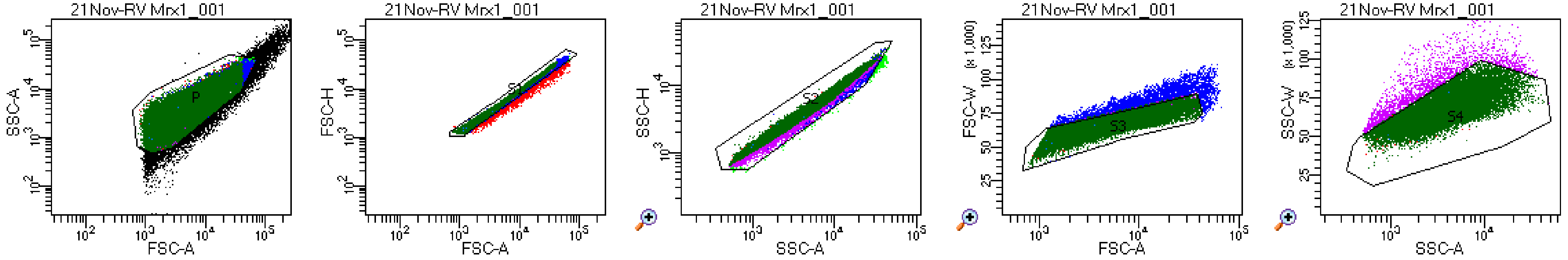

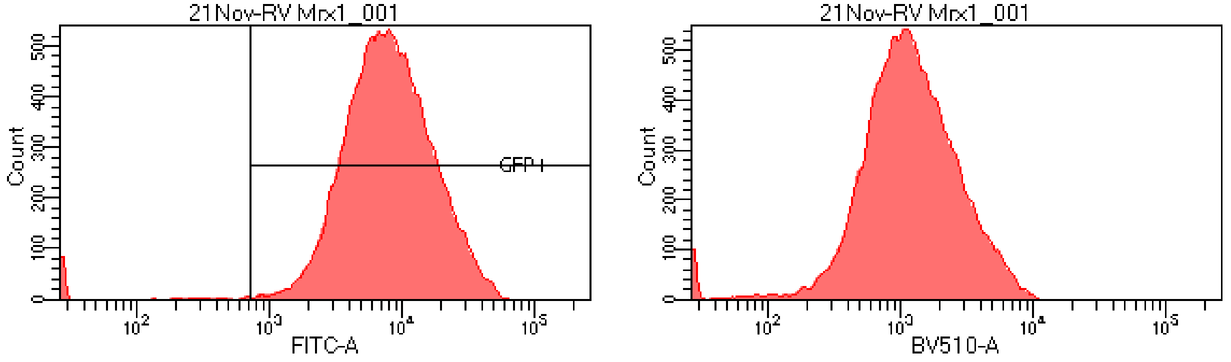

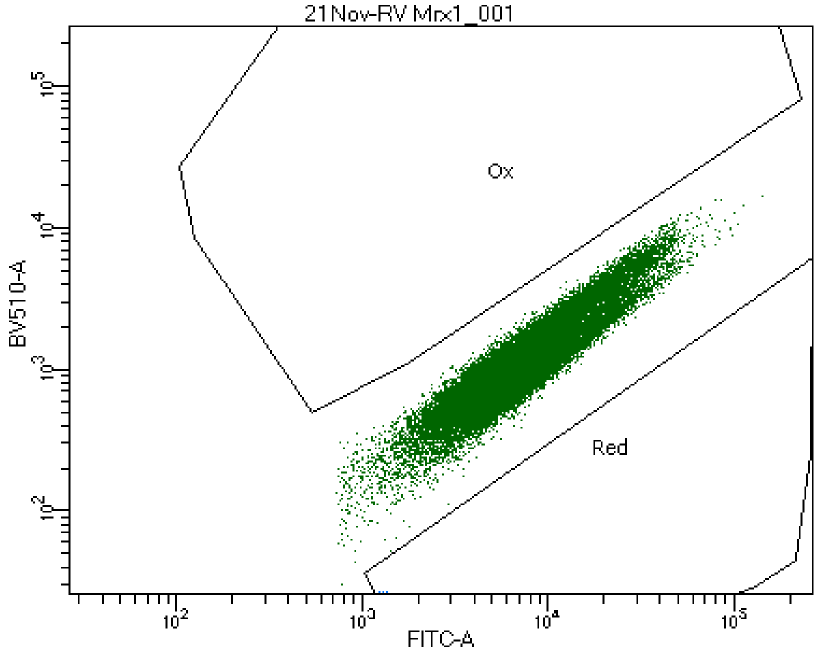

Tube: RV Mrx1_001

Population	#Events	%Parent	%Total
All Events	40,179	####	100.0
P	35,553	88.5	88.5
S1	33,849	95.2	84.2
S2	33,562	99.2	83.5
S3	30,240	90.1	75.3
S4	28,319	93.6	70.5
GFP+	27,665	97.7	68.9
Ox	0	0.0	0.0
Red	4	0.0	0.0

Experiment Name:	21Nov2016 Bac sorting
Specimen Name:	21Nov
Tube Name:	RV Mrx1_001
Record Date:	Nov 21, 2016 2:31:13 PM
$OP:	Administrator
GUID:	3bb8c48d-03bf-4d5e-b0a6-d60...

Population	#Events	%Parent	FITC-A Median	BV510-A Median
S4	28,319	93.6	7,432	1,099
GFP+	27,665	97.7	7,603	1,121
Ox	0	0.0	####	####
Red	4	0.0	1,243	9

BD FACSDiva 8.0.1

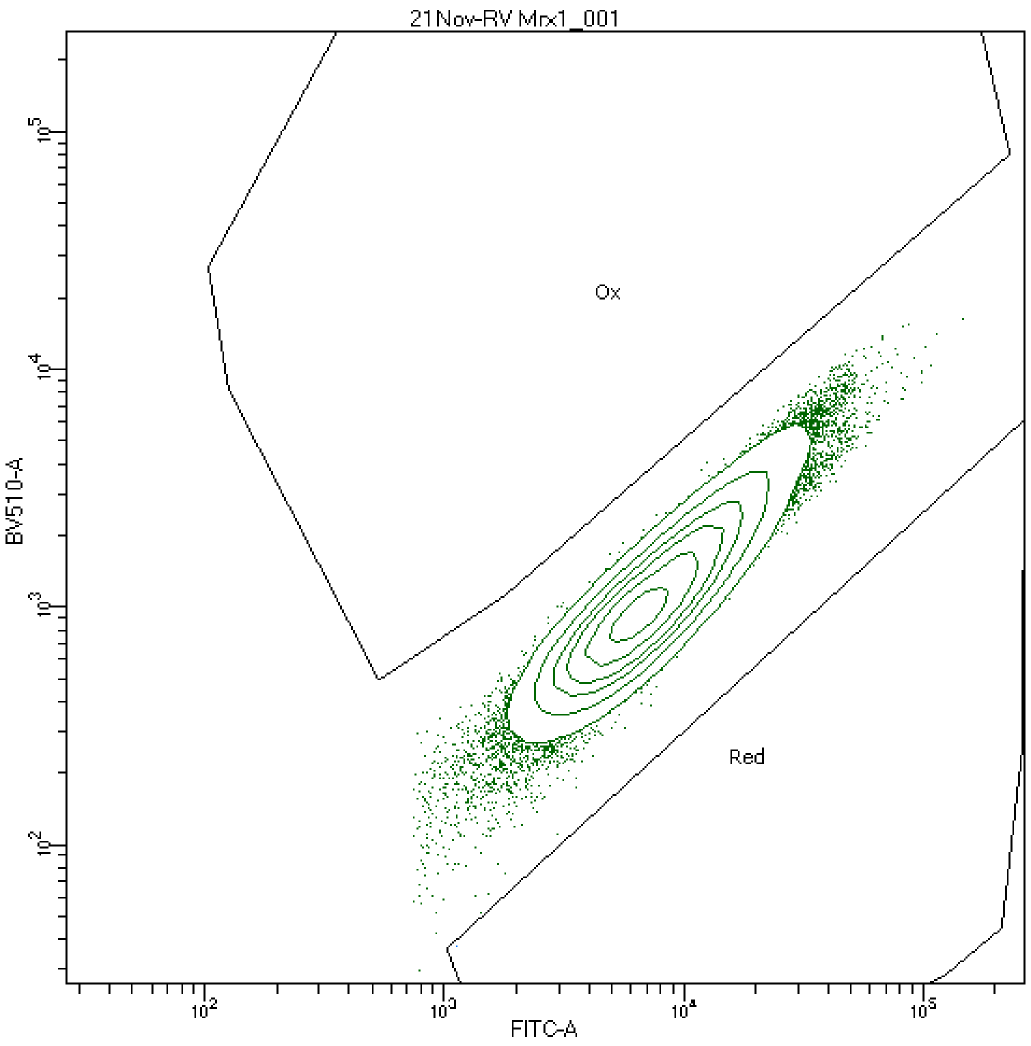

BD FACSDiva 8.0.1

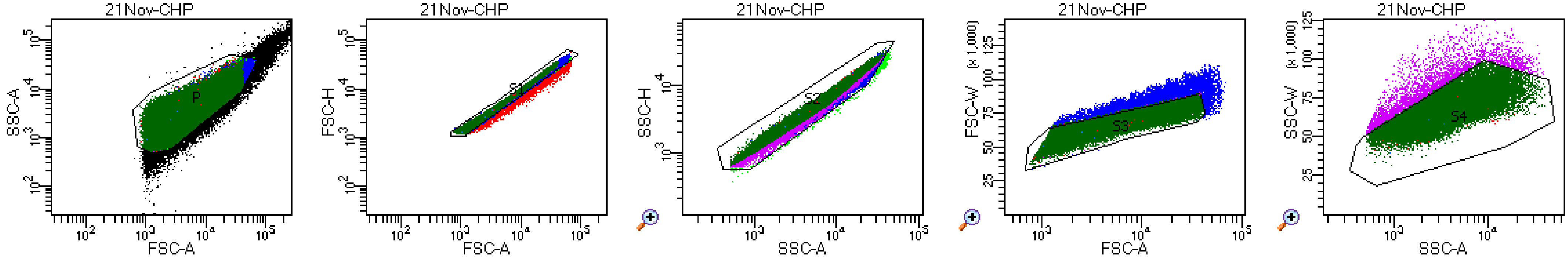

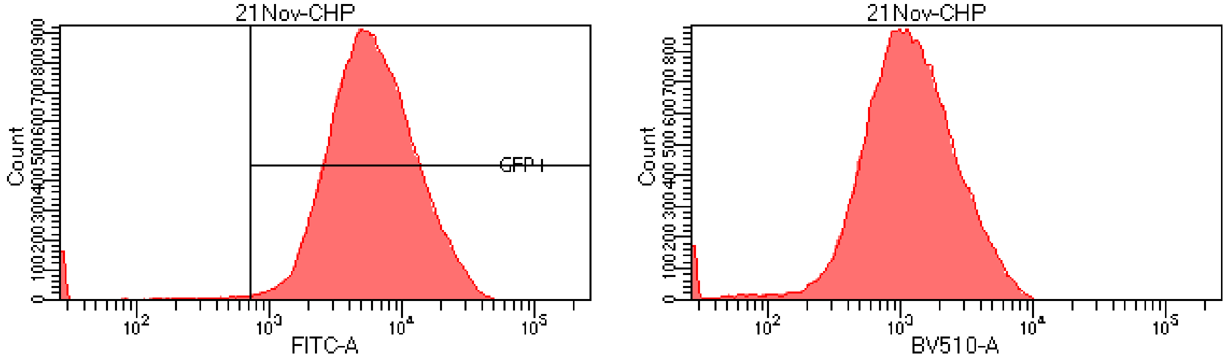

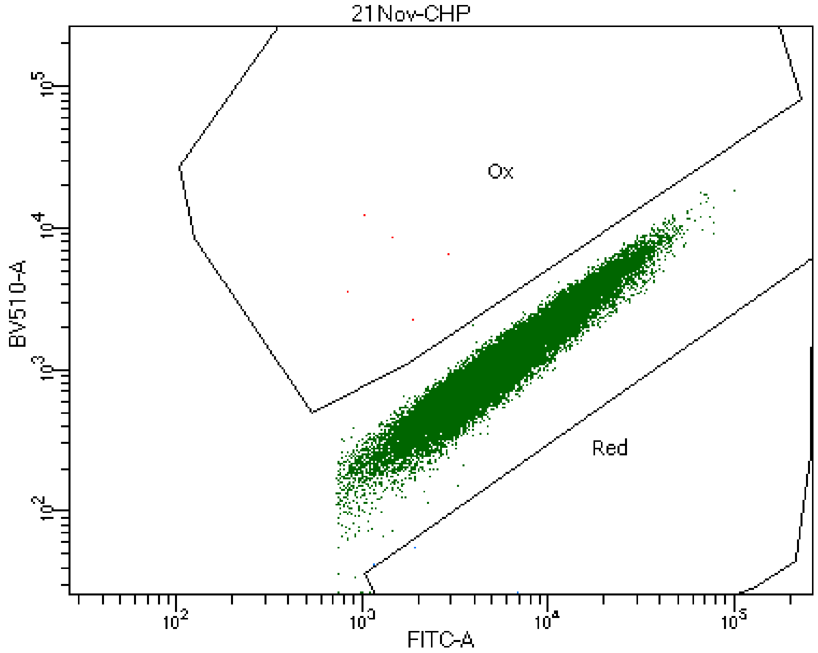

Tube: CHP

Population	#Events	%Parent	%Total
All Events	66,105	####	100.0
P	59,301	89.7	89.7
S1	56,332	95.0	85.2
S2	55,826	99.1	84.5
S3	50,329	90.2	76.1
S4	47,182	93.7	71.4
GFP+	45,833	97.1	69.3
Ox	5	0.0	0.0
Red	3	0.0	0.0

Experiment Name: 21Nov2016 Bac sorting
Specimen Name: 21Nov
Tube Name: CHP
Record Date: Nov 21, 2016 2:31:50 PM
$OP: Administrator
GUID: 4670f93e-731f-4aac-b304-c644...

Population	#Events	%Parent	FITC-A Median	BV510-A Median
S4	47,182	93.7	5,551	1,091
GFP+	45,833	97.1	5,711	1,119
Ox	5	0.0	1,437	6,458
Red	3	0.0	1,873	42

BD FACSDiva 8.0.1

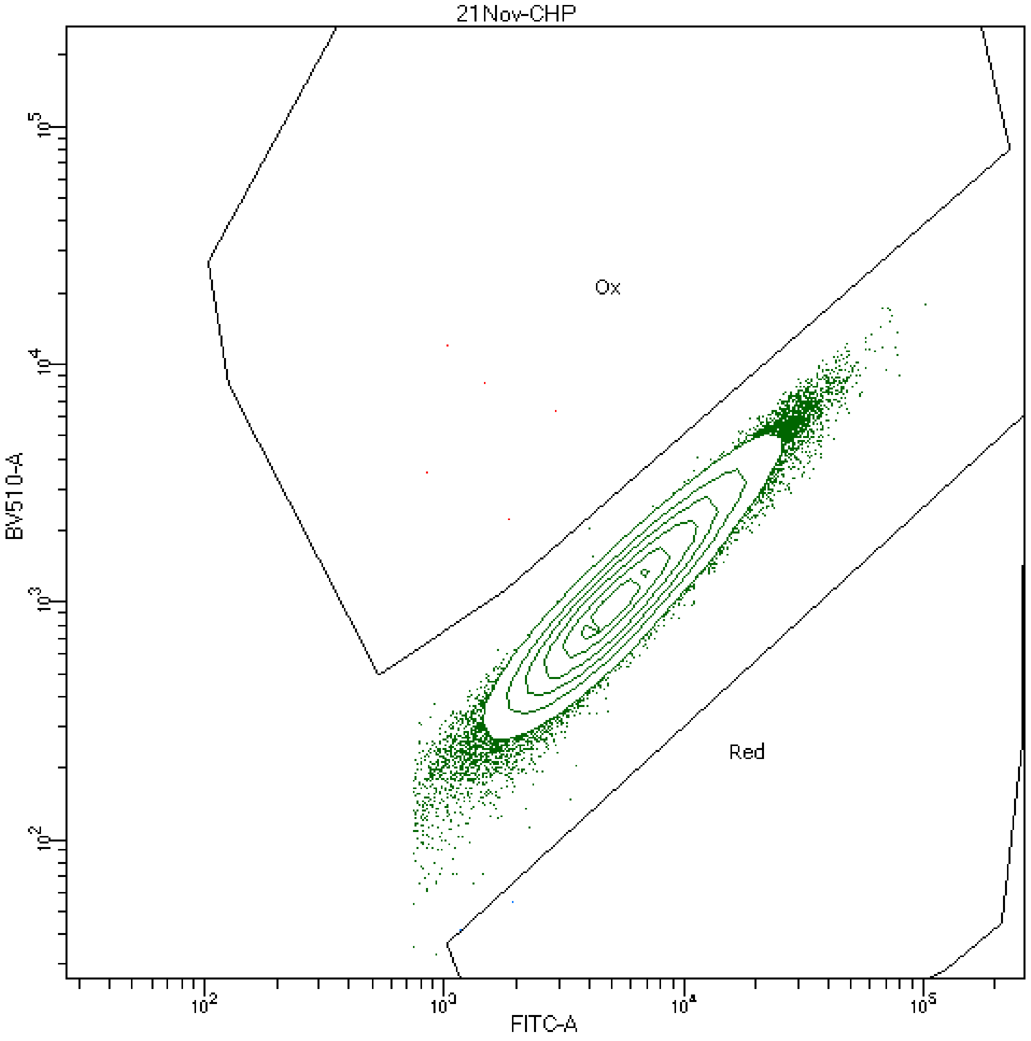

BD FACSDiva 8.0.1

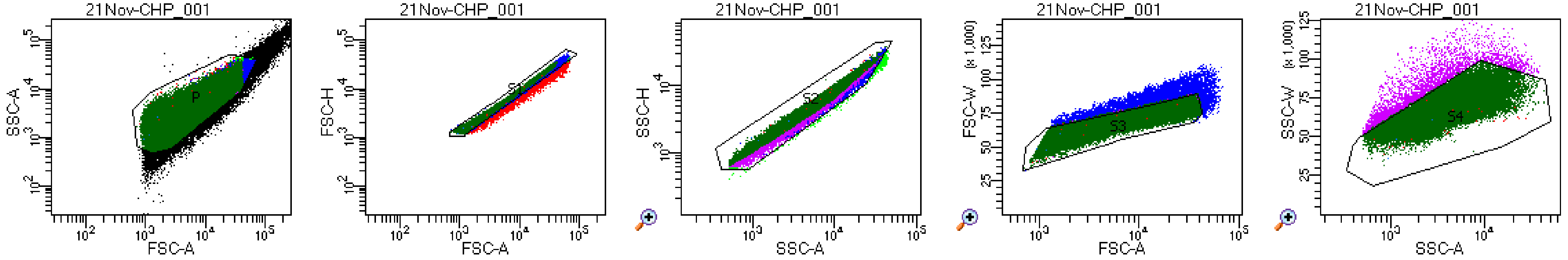

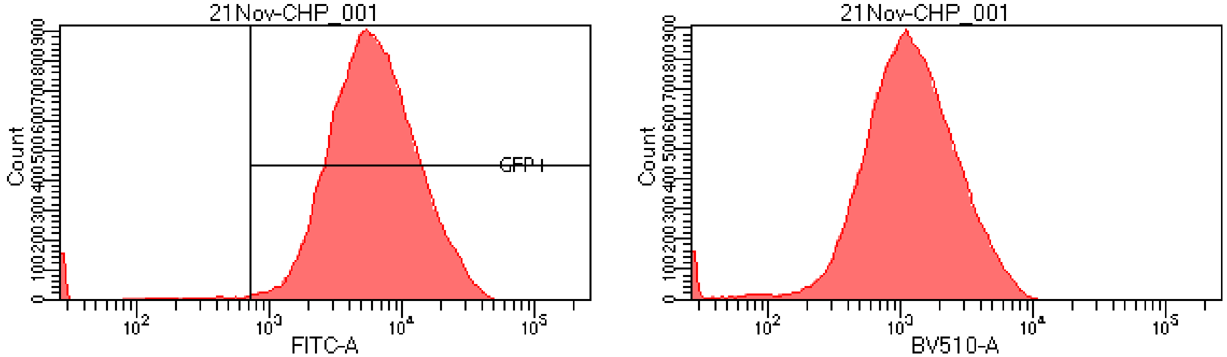

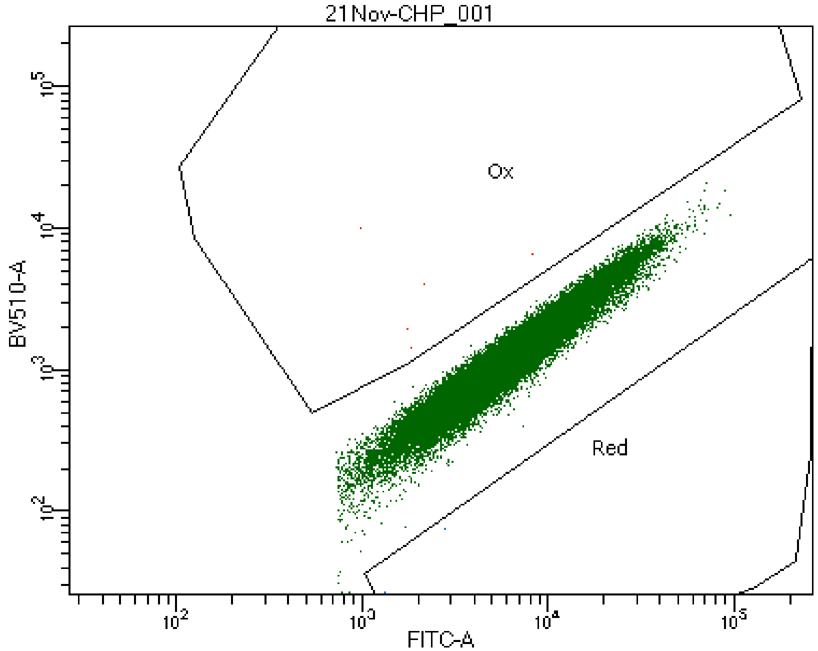

Tube: CHP_001

Population	#Events	%Parent	%Total
All Events	66,176	####	100.0
P	59,246	89.5	89.5
S1	56,332	95.1	85.1
S2	55,812	99.1	84.3
S3	50,331	90.2	76.1
S4	47,191	93.8	71.3
GFP+	45,870	97.2	69.3
Ox	6	0.0	0.0
Red	3	0.0	0.0

Experiment Name:	21Nov2016 Bac sorting
Specimen Name:	21Nov
Tube Name:	CHP_001
Record Date:	Nov 21, 2016 2:32:27 PM
$OP:	Administrator
GUID:	082ac3e6-ea69-4c4a-8f5c-049...

Population	#Events	%Parent	FITC-A Median	BV510-A Median
S4	47,191	93.8	5,680	1,112
GFP+	45,870	97.2	5,832	1,141
Ox	6	0.0	1,960	3,186
Red	3	0.0	1,348	45

BD FACSDiva 8.0.1

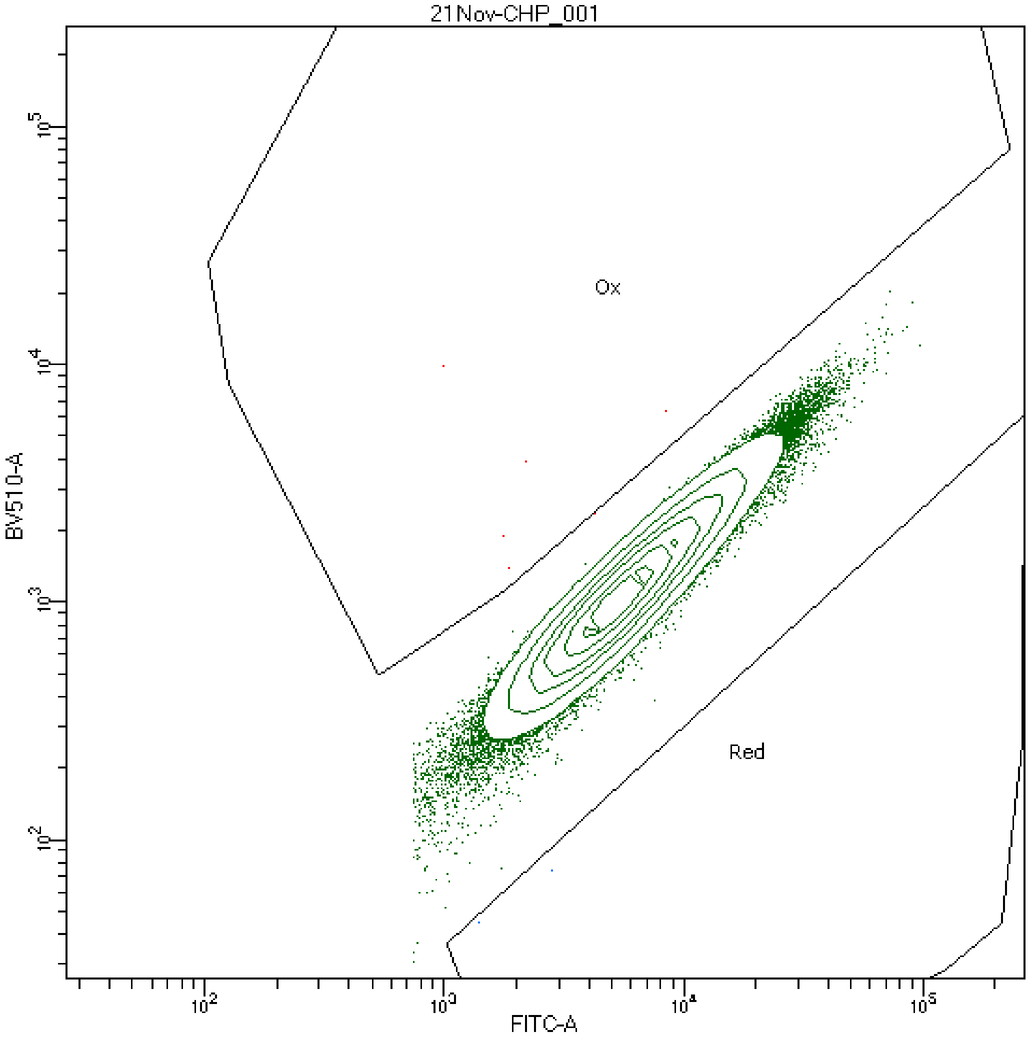

BD FACSDiva 8.0.1

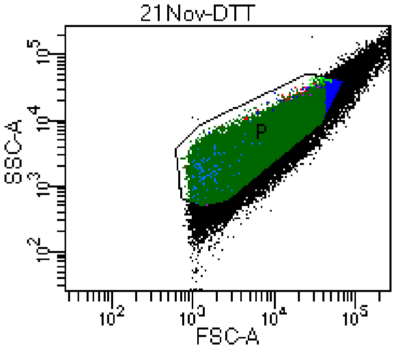

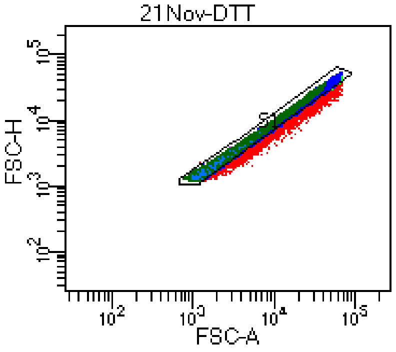

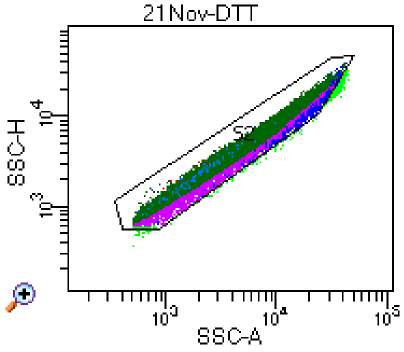

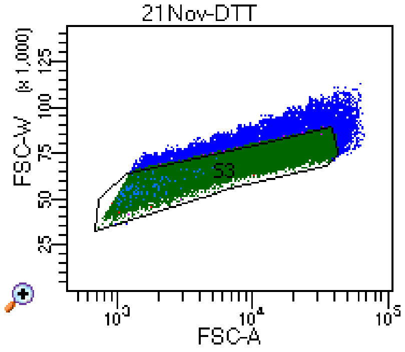

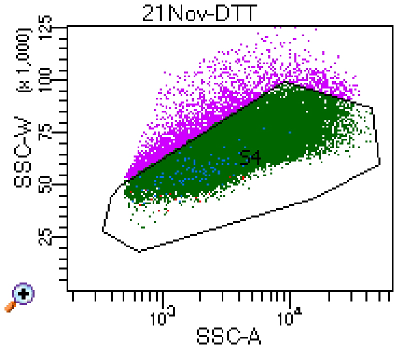

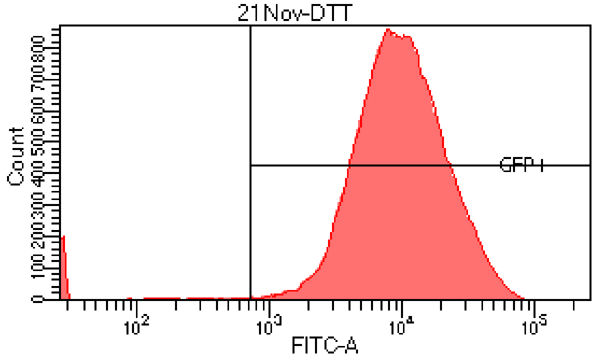

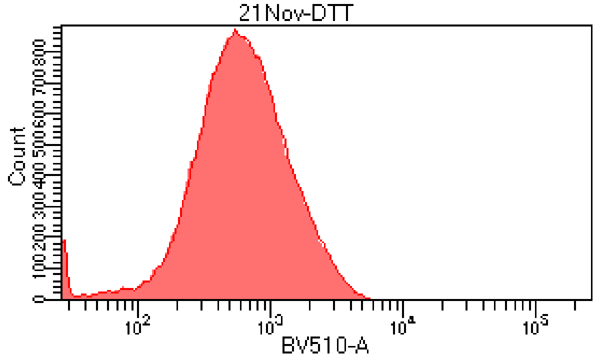

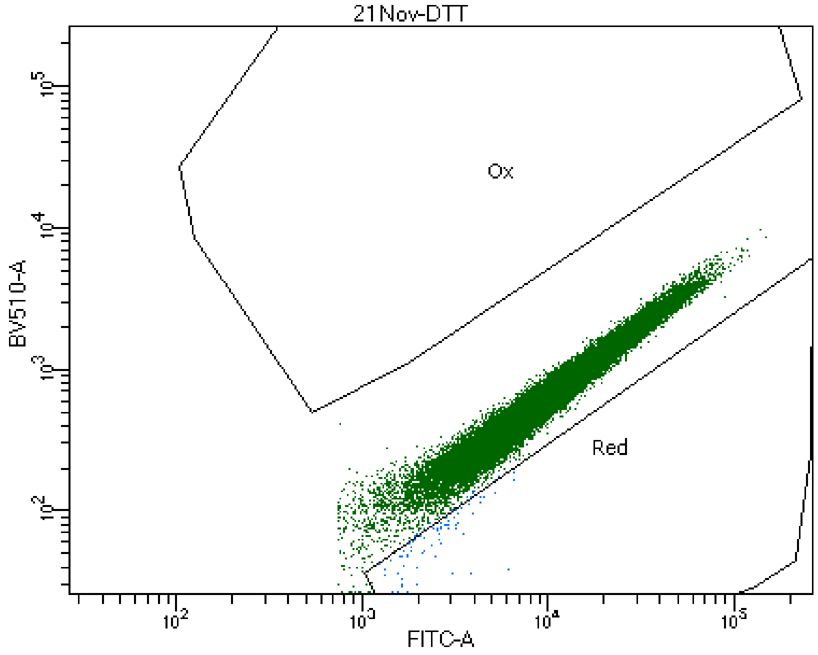

Tube: DTT

Population	#Events	%Parent	%Total
All Events	67,341	####	100.0
P	59,044	87.7	87.7
S1	55,948	94.8	83.1
S2	55,517	99.2	82.4
S3	49,412	89.0	73.4
S4	46,459	94.0	69.0
GFP+	45,227	97.3	67.2
Ox	0	0.0	0.0
Red	91	0.2	0.1

Experiment Name:	21Nov2016 Bac sorting
Specimen Name:	21Nov
Tube Name:	DTT
Record Date:	Nov 21, 2016 2:33:07 PM
$OP:	Administrator
GUID:	ca9c42d3-7f3b-4f51-9755-cb62f...

Population	#Events	%Parent	FITC-A Median	BV510-A Median
S4	46,459	94.0	9,158	574
GFP+	45,227	97.3	9,395	588
Ox	0	0.0	####	####
Red	91	0.2	2,509	71

BD FACSDiva 8.0.1

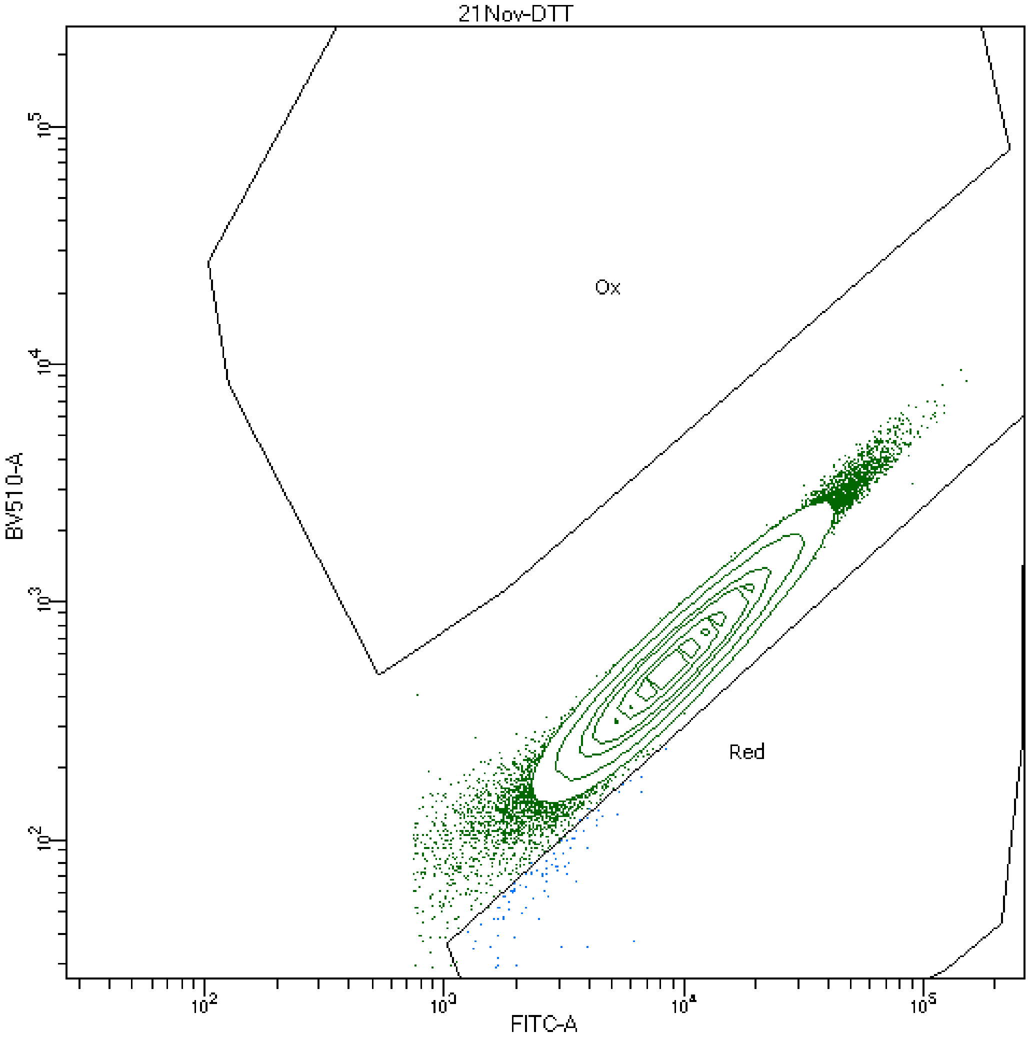

BD FACSDiva 8.0.1

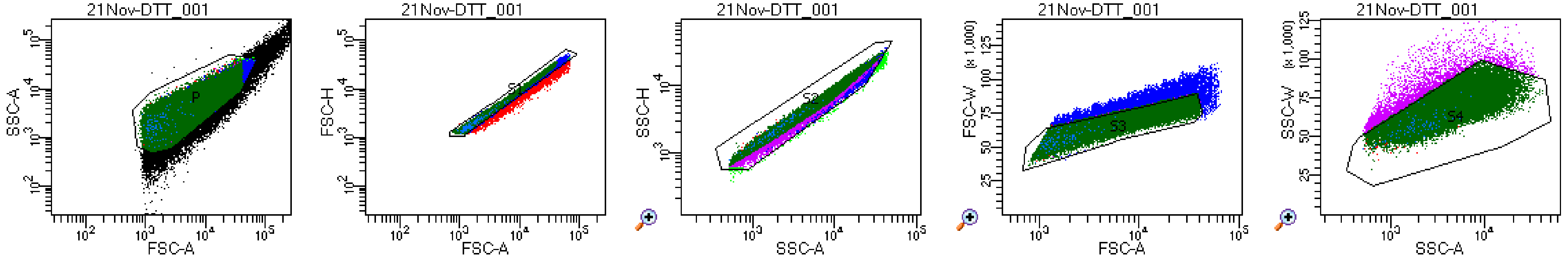

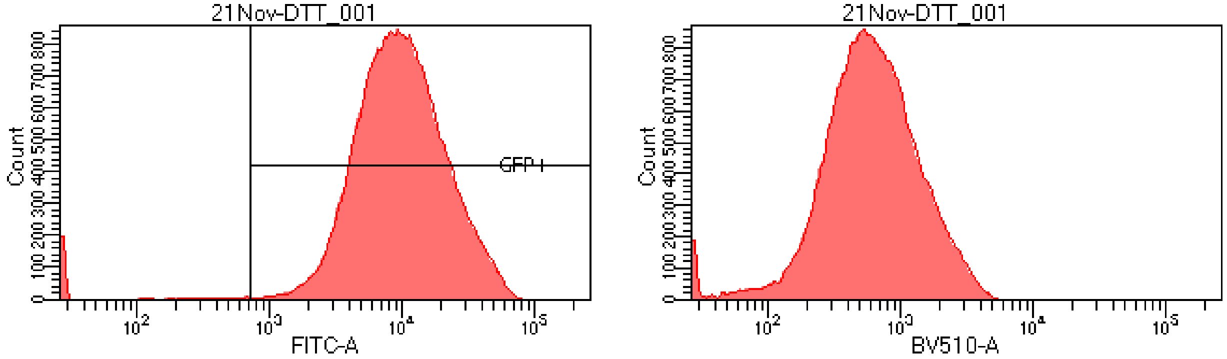

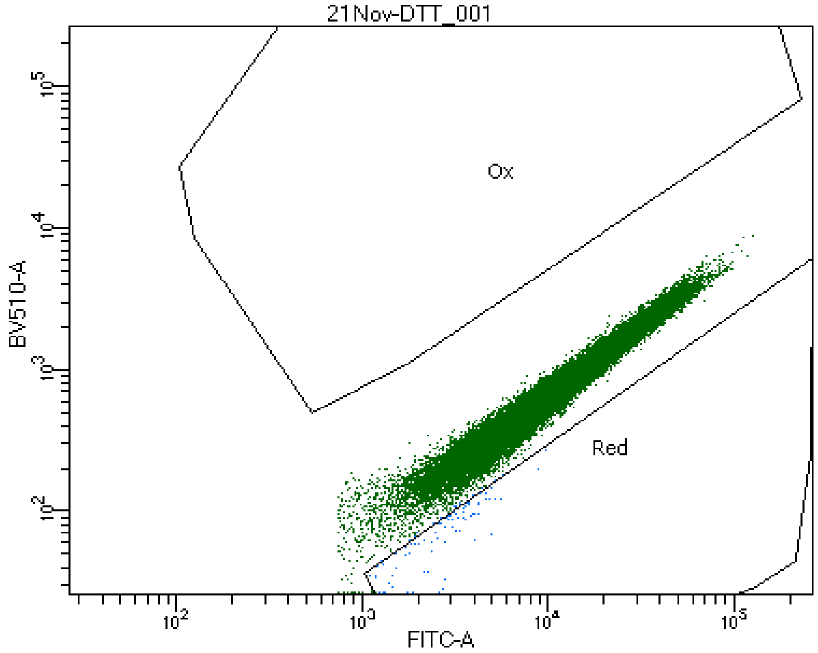

Tube: DTT_001

Population	#Events	%Parent	%Total
All Events	67,314	####	100.0
P	59,252	88.0	88.0
S1	56,030	94.6	83.2
S2	55,604	99.2	82.6
S3	49,283	88.6	73.2
S4	46,330	94.0	68.8
GFP+	45,064	97.3	66.9
Ox	0	0.0	0.0
Red	101	0.2	0.2

Experiment Name: 21Nov2016 Bac sorting
Specimen Name: 21Nov
Tube Name: DTT_001
Record Date: Nov 21, 2016 2:33:51 PM
$OP: Administrator
GUID: 6f0774a9-a53a-45ea-a2aa-82f7...

Population	#Events	%Parent	FITC-A Median	BV510-A Median
S4	46,330	94.0	9,086	569
GFP+	45,064	97.3	9,322	585
Ox	0	0.0	####	####
Red	101	0.2	2,827	81

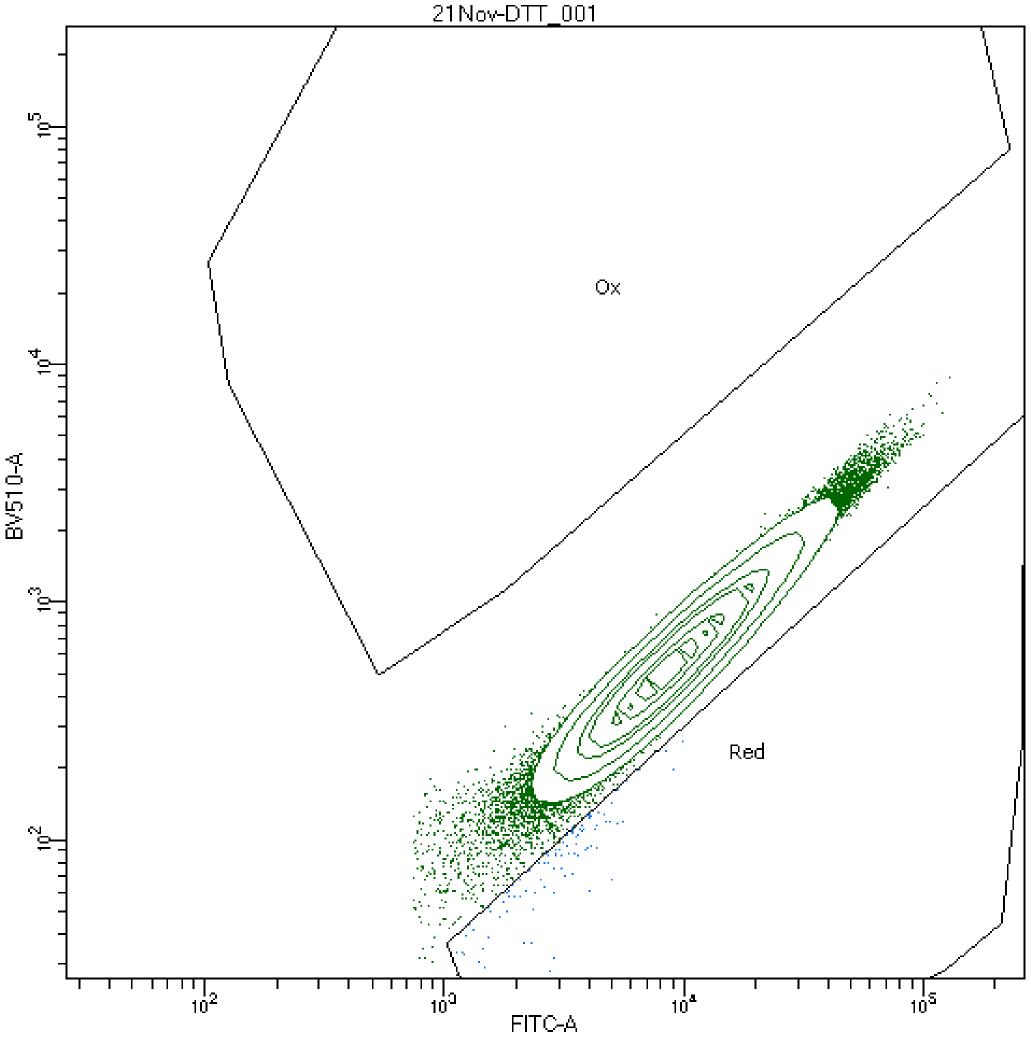
21Nov-DTT_001
Ox
Red
BV510-A
FITC-A
10^2
10^3
10^4
10^5

BD FACSDiva 8.0.1

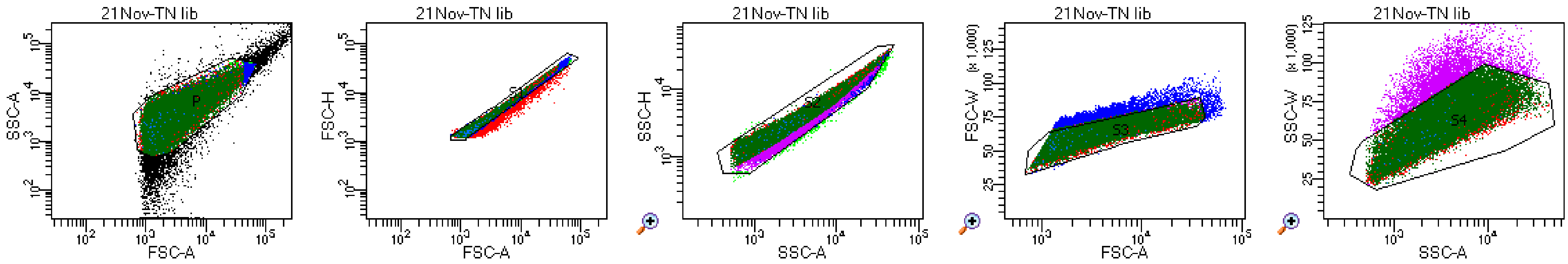

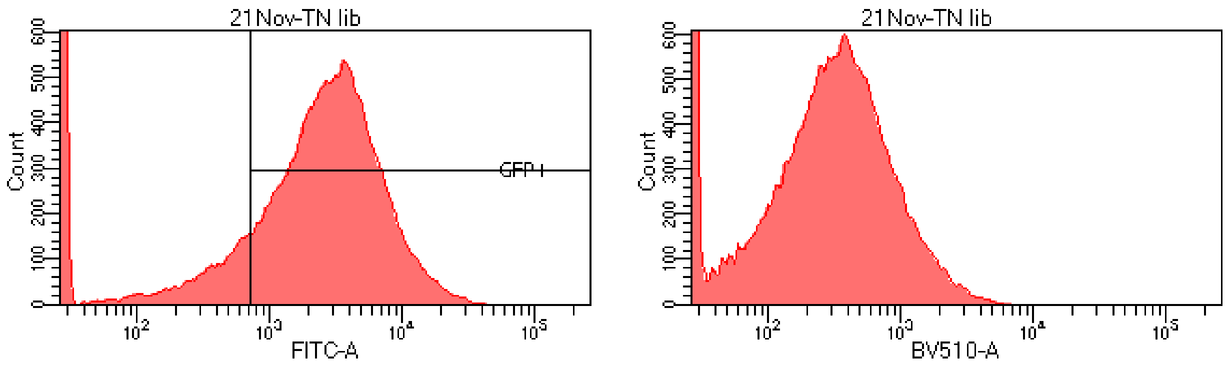

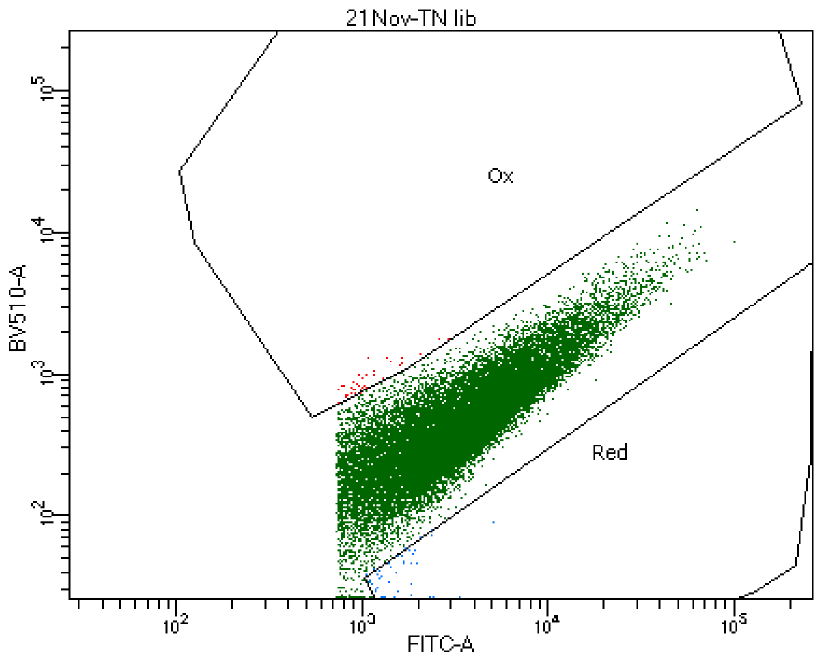

Tube: TN lib

Population	#Events	%Parent	%Total
All Events	59,864	####	100.0
P	55,510	92.7	92.7
S1	51,592	92.9	86.2
S2	51,191	99.2	85.5
S3	45,787	89.4	76.5
S4	41,416	90.5	69.2
GFP+	28,203	68.1	47.1
Ox	37	0.1	0.1
Red	47	0.2	0.1

Experiment Name: 21Nov2016 Bac sorting
Specimen Name: 21Nov
Tube Name: TN lib
Record Date: Nov 21, 2016 2:34:30 PM
$OP: Administrator
GUID: 758ac635-90fc-4e2f-9da8-7906...

Population	#Events	%Parent	FITC-A Median	BV510-A Median
S4	41,416	90.5	1,890	275
GFP+	28,203	68.1	3,106	408
Ox	37	0.1	958	822
Red	47	0.2	1,456	39

BD FACSDiva 8.0.1

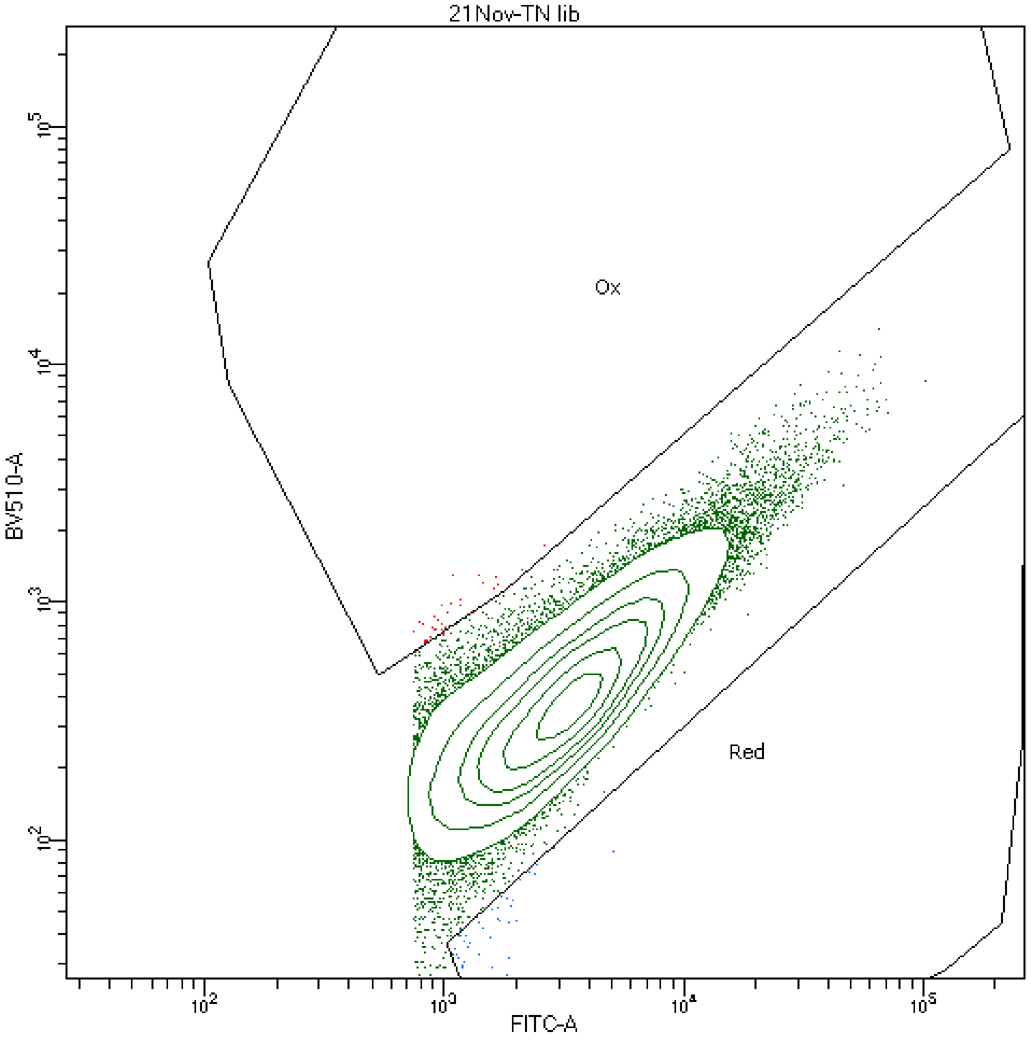

BD FACSDiva 8.0.1

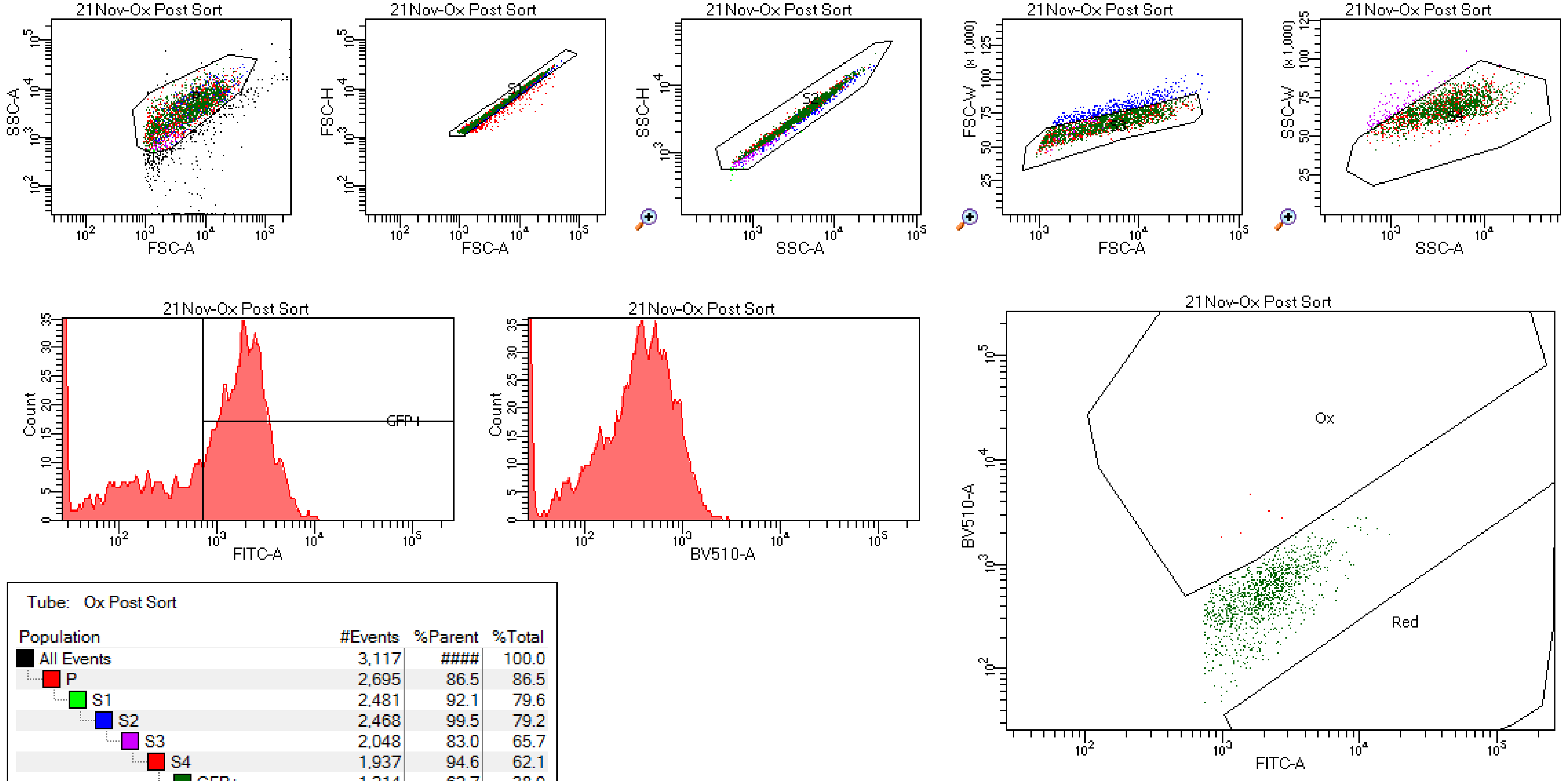

Tube: Ox Post Sort

Population	#Events	%Parent	%Total
All Events	3,117	####	100.0
P	2,695	86.5	86.5
S1	2,481	92.1	79.6
S2	2,468	99.5	79.2
S3	2,048	83.0	65.7
S4	1,937	94.6	62.1
GFP+	1,214	62.7	38.9
Ox	6	0.5	0.2
Red	0	0.0	0.0

Experiment Name:	21Nov2016 Bac sorting
Specimen Name:	21Nov
Tube Name:	Ox Post Sort
Record Date:	Nov 21, 2016 4:36:16 PM
$OP:	Administrator
GUID:	d1ae7021-5e3d-41c2-a068-581...

Population	#Events	%Parent	FITC-A Median	BV510-A Median
S4	1,937	94.6	1,182	354
GFP+	1,214	62.7	1,931	507
Ox	6	0.5	1,861	3,019
Red	0	0.0	####	####

BD FACSDiva 8.0.1

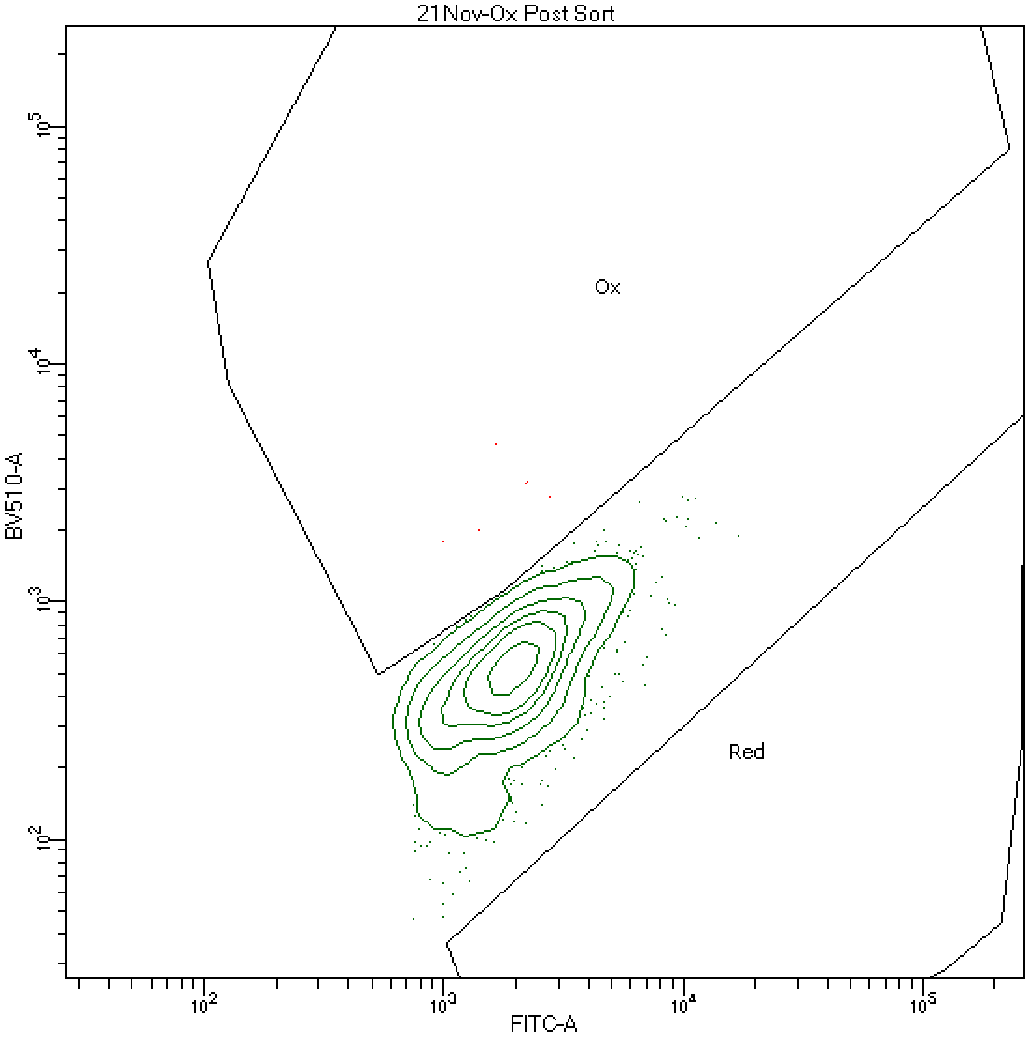

BD FACSDiva 8.0.1

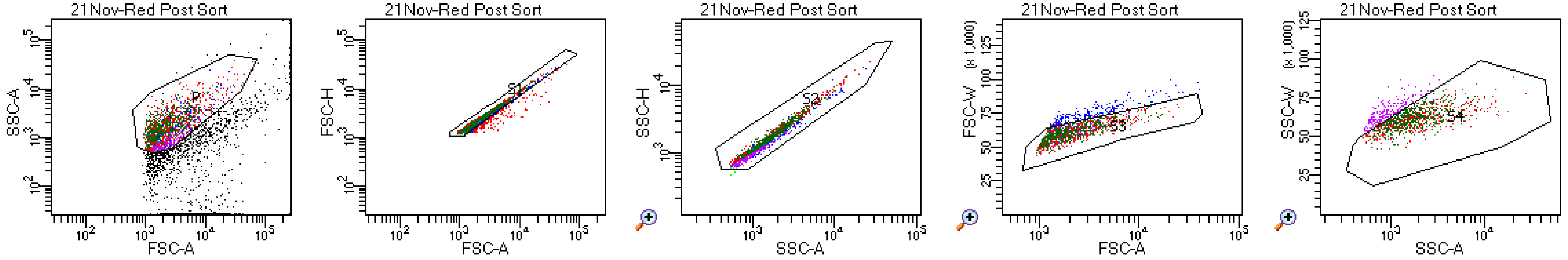

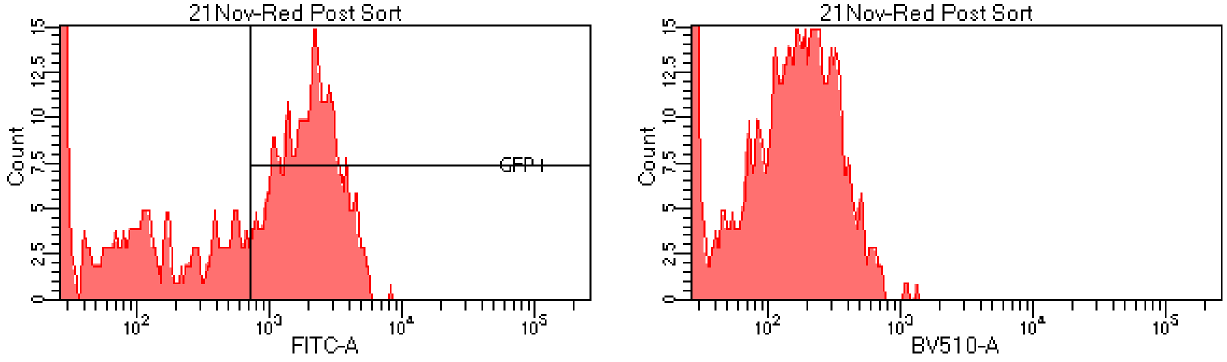

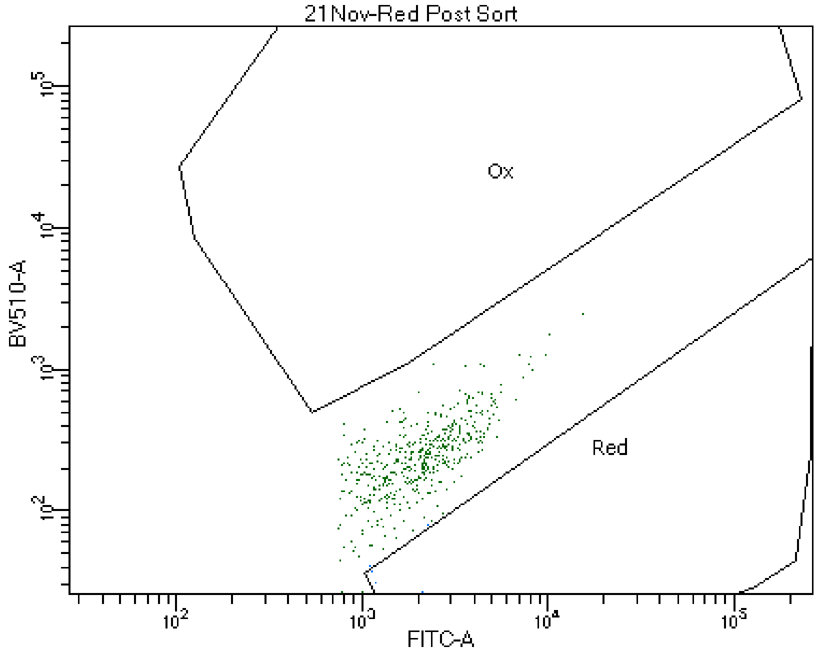

Tube: Red Post Sort

Population	#Events	%Parent	%Total
All Events	2,960	####	100.0
P	1,549	52.3	52.3
S1	1,349	87.1	45.6
S2	1,339	99.3	45.2
S3	1,132	84.5	38.2
S4	940	83.0	31.8
GFP+	483	51.4	16.3
Ox	0	0.0	0.0
Red	6	1.2	0.2

Experiment Name: 21Nov2016 Bac sorting
Specimen Name: 21Nov
Tube Name: Red Post Sort
Record Date: Nov 21, 2016 4:37:09 PM
$OP: Administrator
GUID: a6087b50-f85d-4a1f-8b21-8c89...

Population	#Events	%Parent	FITC-A Median	BV510-A Median
S4	940	83.0	783	139
GFP+	483	51.4	2,063	233
Ox	0	0.0	####	####
Red	6	1.2	1,546	39

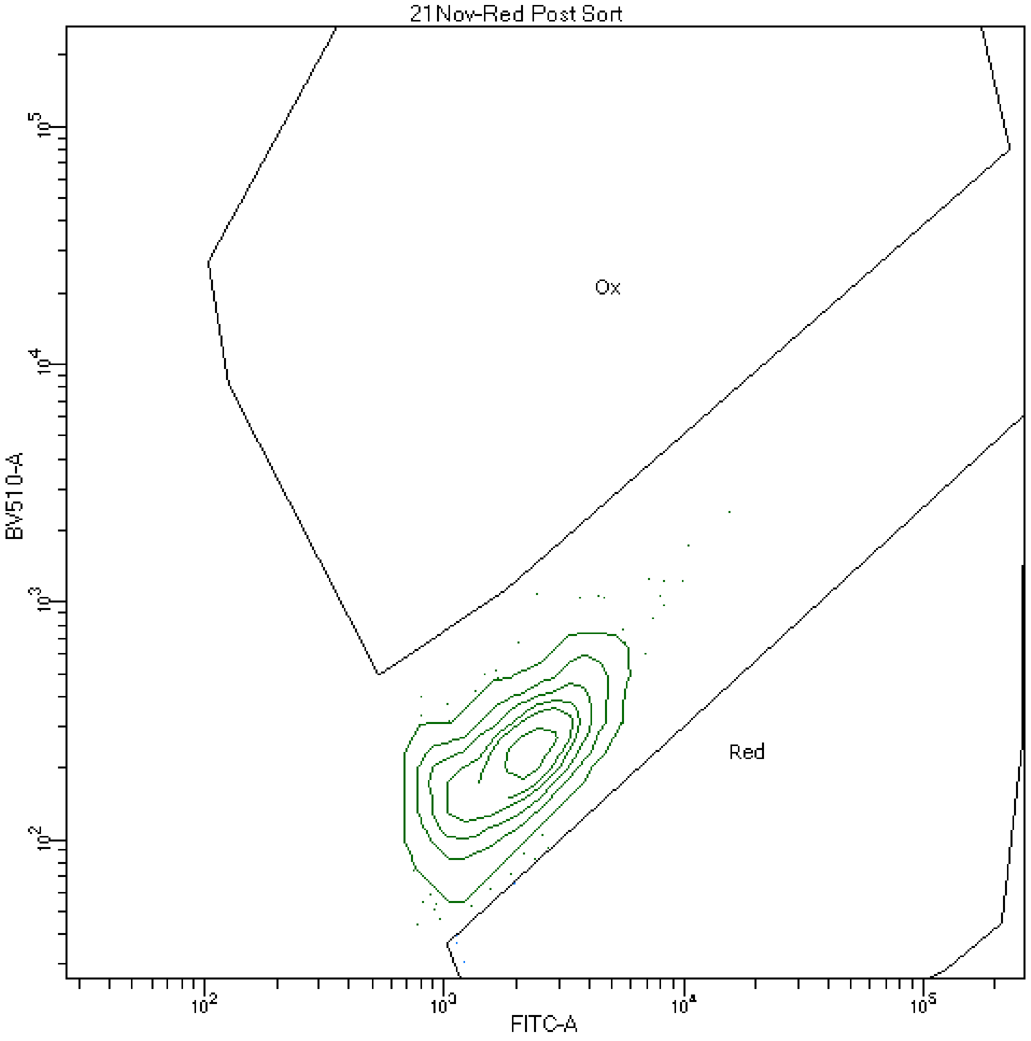
21Nov-Red Post Sort
Ox
Red
BV510-A
FITC-A
10^2
10^3
10^4
10^5

BD FACSDiva 8.0.1

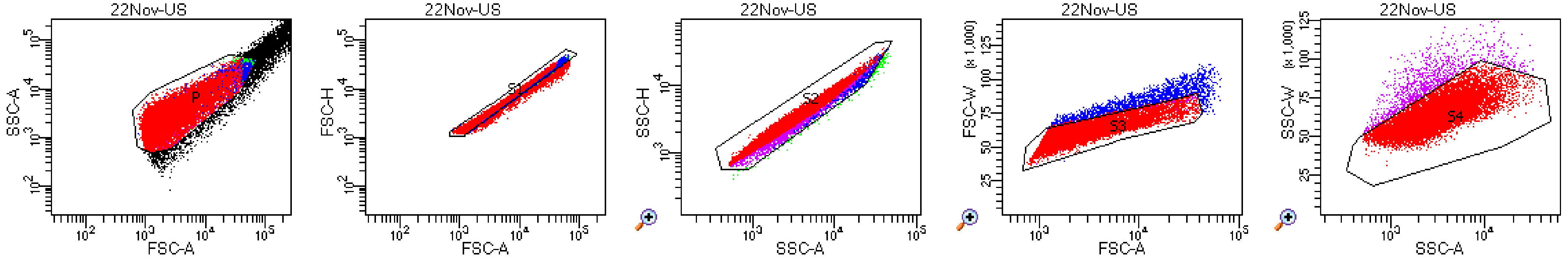

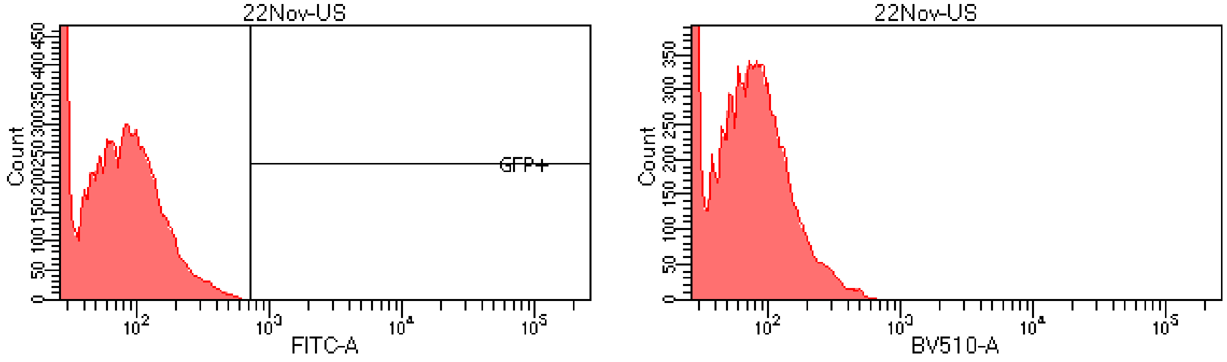

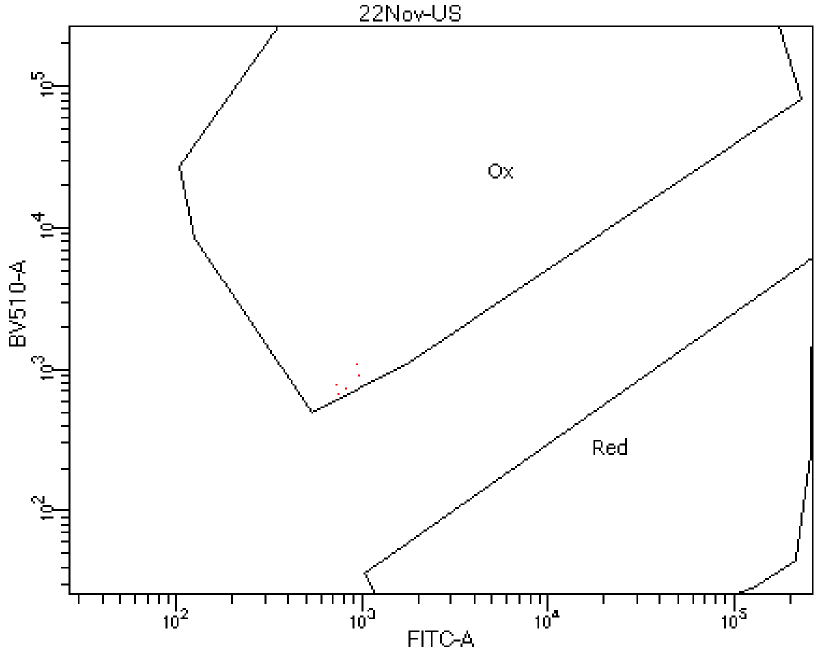

Tube: US

Population	#Events	%Parent	%Total
All Events	26,663	####	100.0
P	22,243	83.4	83.4
S1	20,748	93.3	77.8
S2	20,558	99.1	77.1
S3	18,774	91.3	70.4
S4	17,638	93.9	66.2
GFP+	5	0.0	0.0
Ox	5	100.0	0.0
Red	0	0.0	0.0

Experiment Name:	21Nov2016 Bac sorting
Specimen Name:	22Nov
Tube Name:	US
Record Date:	Nov 22, 2016 2:12:56 PM
$OP:	Administrator
GUID:	abdcd656-7aff-4ad2-bb8e-6567...

Population	#Events	%Parent	FITC-A Median	BV510-A Median
S4	17,638	93.9	58	61
GFP+	5	0.0	797	776
Ox	5	100.0	797	776
Red	0	0.0	####	####

BD FACSDiva 8.0.1

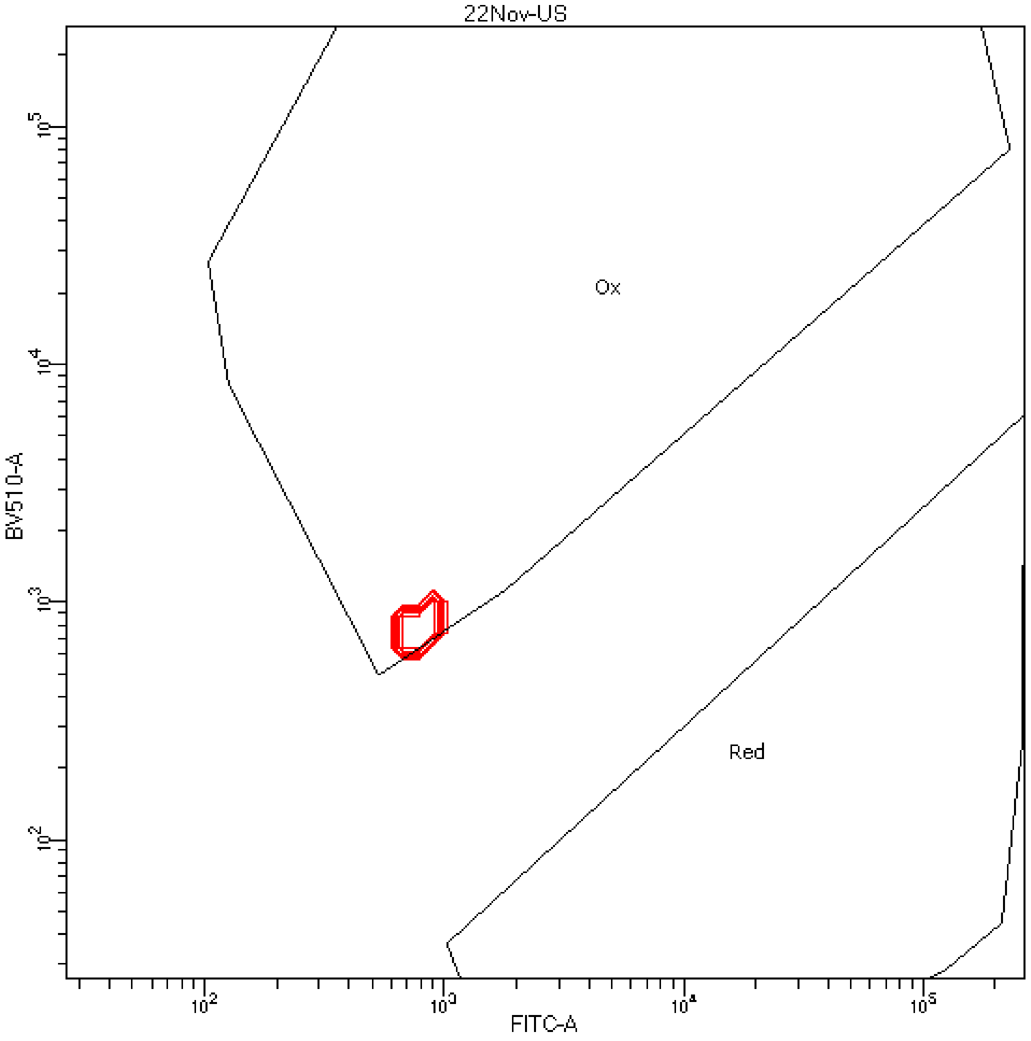

BD FACSDiva 8.0.1

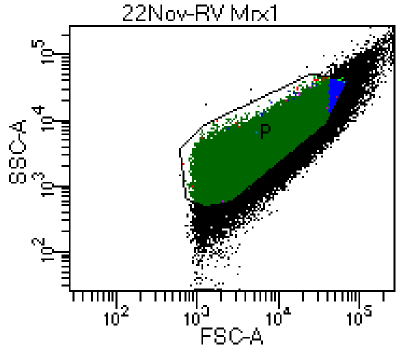

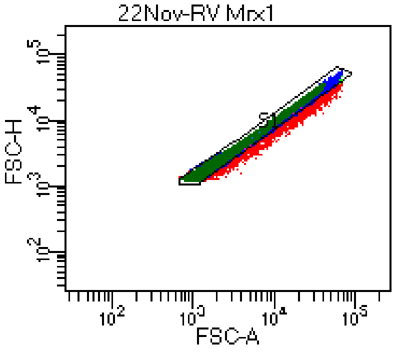

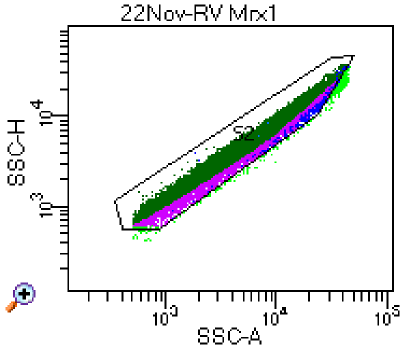

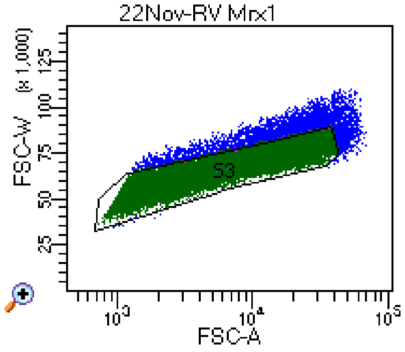

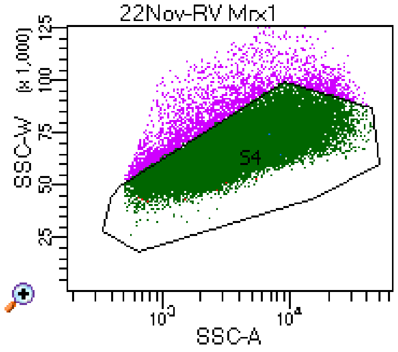

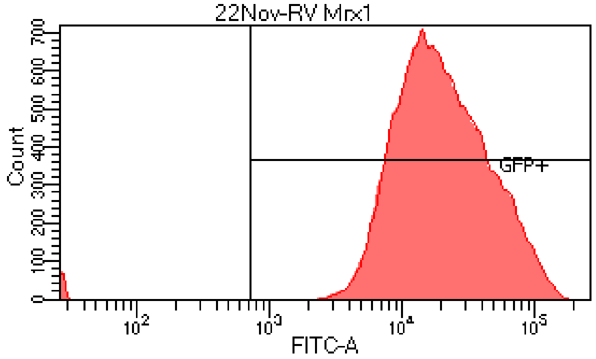

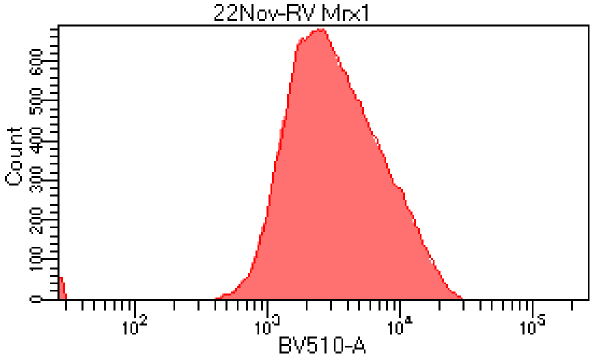

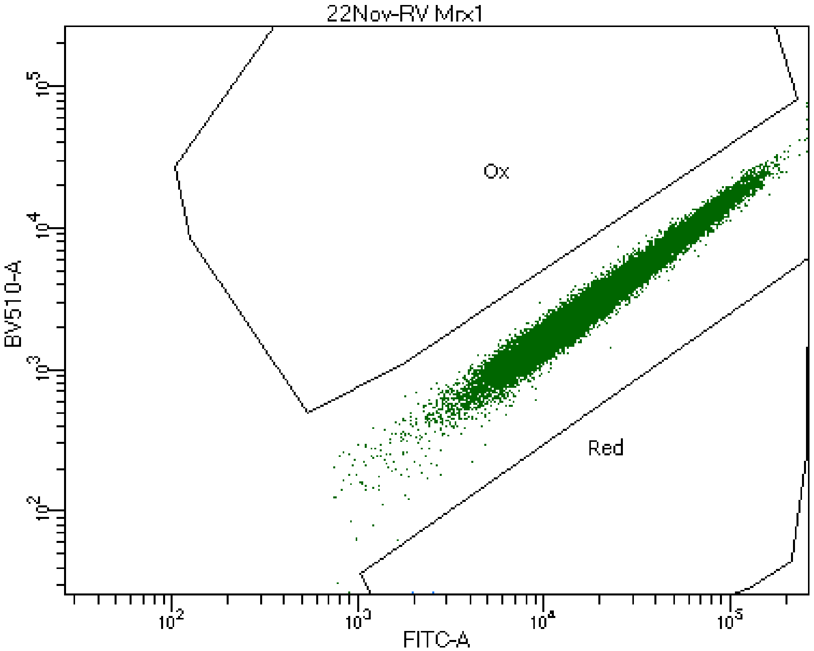

Tube: RV Mrx1

Population	#Events	%Parent	%Total
All Events	58,229	####	100.0
P	47,601	81.7	81.7
S1	45,562	95.7	78.2
S2	45,024	98.8	77.3
S3	41,130	91.4	70.6
S4	38,397	93.4	65.9
GFP+	38,169	99.4	65.5
Ox	0	0.0	0.0
Red	2	0.0	0.0

Experiment Name: 21Nov2016 Bac sorting
Specimen Name: 22Nov
Tube Name: RV Mrx1
Record Date: Nov 22, 2016 2:13:47 PM
$OP: Administrator
GUID: f42a0f51-a1bd-4ccf-a128-adba...

Population	#Events	%Parent	FITC-A Median	BV510-A Median
S4	38,397	93.4	18,230	2,882
GFP+	38,169	99.4	18,334	2,898
Ox	0	0.0	####	####
Red	2	0.0	2,222	15

BD FACSDiva 8.0.1

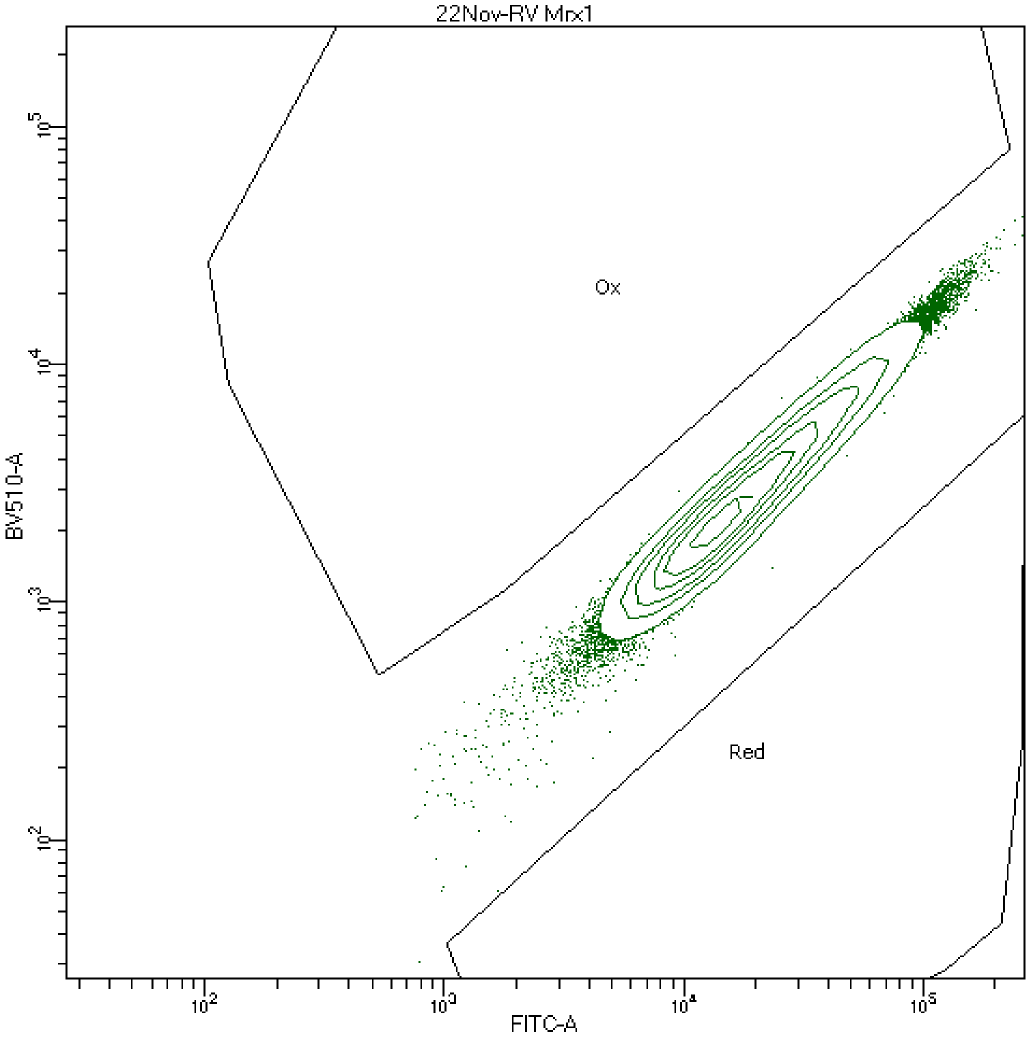

BD FACSDiva 8.0.1

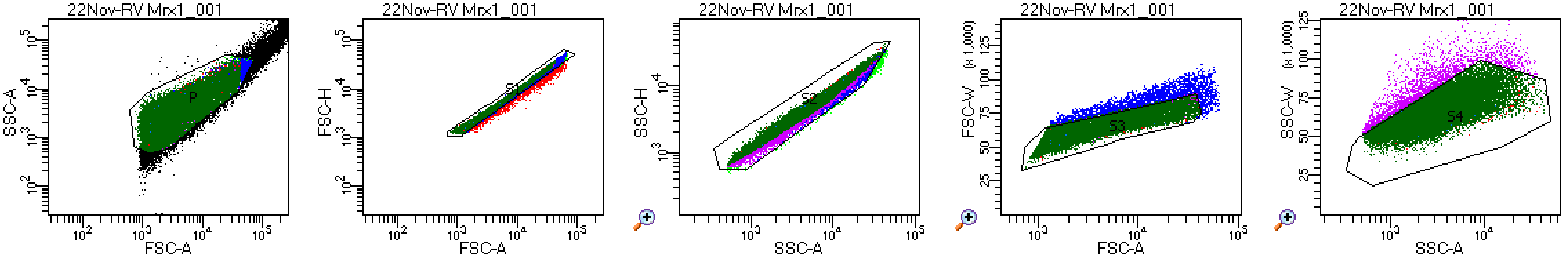

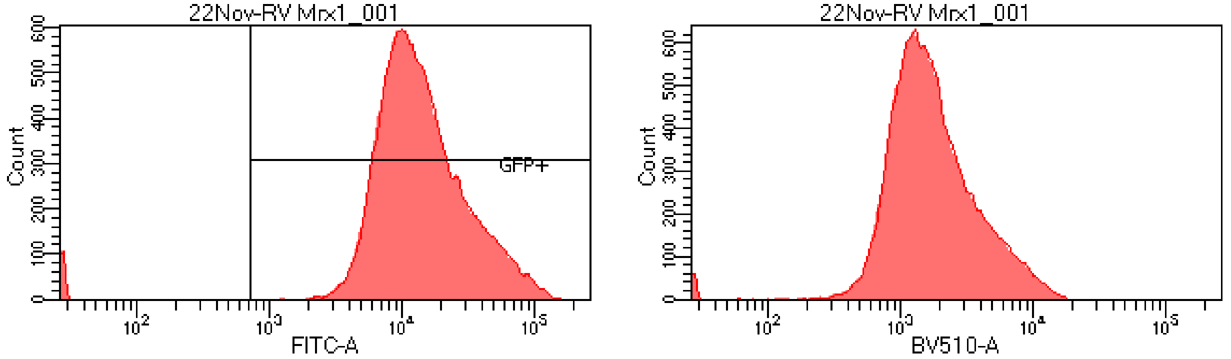

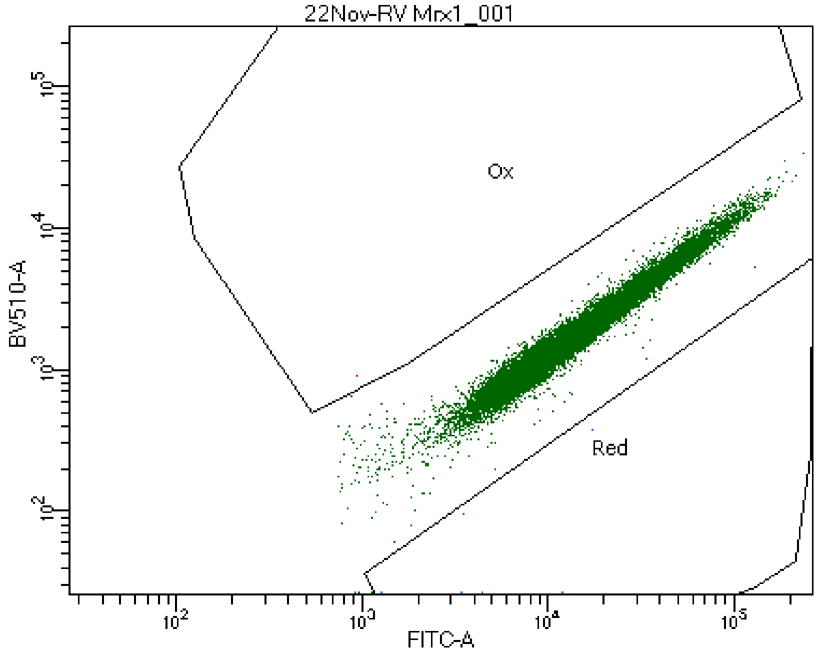

Tube: RV Mrx1_001

Population	#Events	%Parent	%Total
All Events	39,651	####	100.0
P	33,874	85.4	85.4
S1	32,612	96.3	82.2
S2	32,325	99.1	81.5
S3	29,498	91.3	74.4
S4	27,393	92.9	69.1
GFP+	26,964	98.4	68.0
Ox	2	0.0	0.0
Red	7	0.0	0.0

Experiment Name: 21Nov2016 Bac sorting
Specimen Name: 22Nov
Tube Name: RV Mrx1_001
Record Date: Nov 22, 2016 2:14:30 PM
$OP: Administrator
GUID: 9ea0558d-f759-42c4-ba02-f3fa...

Population	#Events	%Parent	FITC-A Median	BV510-A Median
S4	27,393	92.9	12,166	1,504
GFP+	26,964	98.4	12,346	1,522
Ox	2	0.0	2,340	1,520
Red	7	0.0	4,335	-66

BD FACSDiva 8.0.1

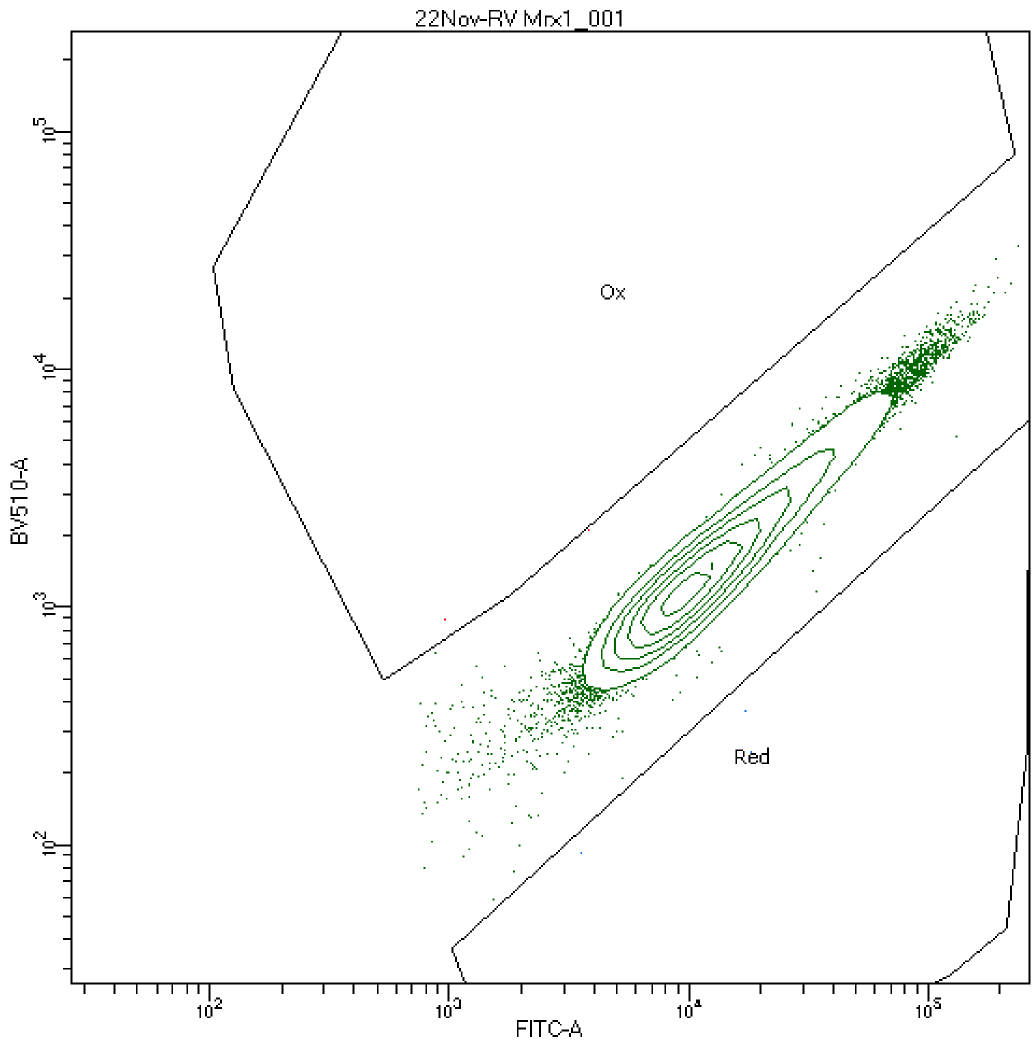

BD FACSDiva 8.0.1

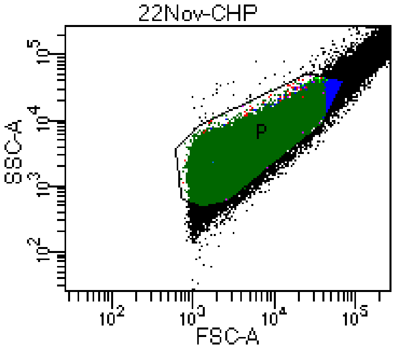

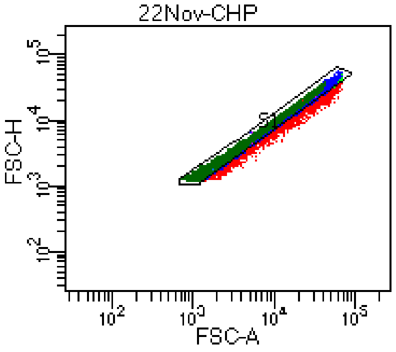

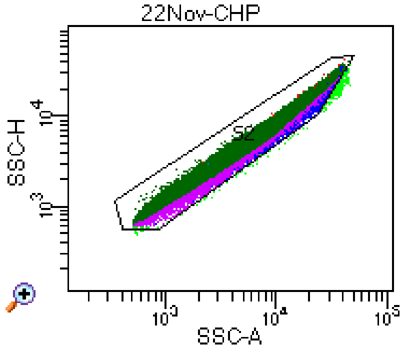

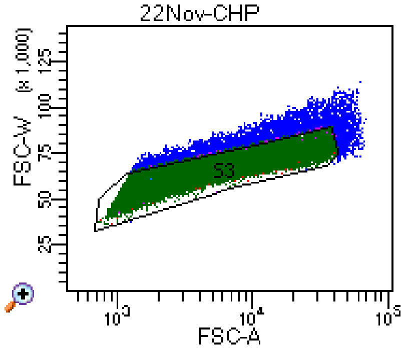

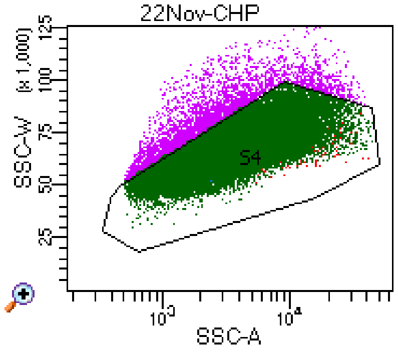

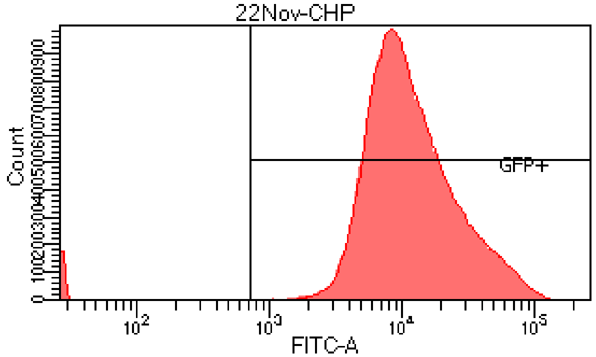

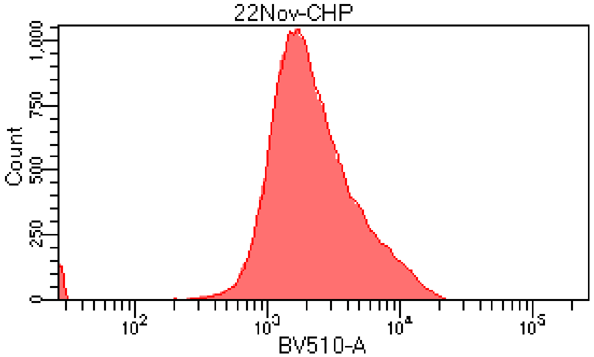

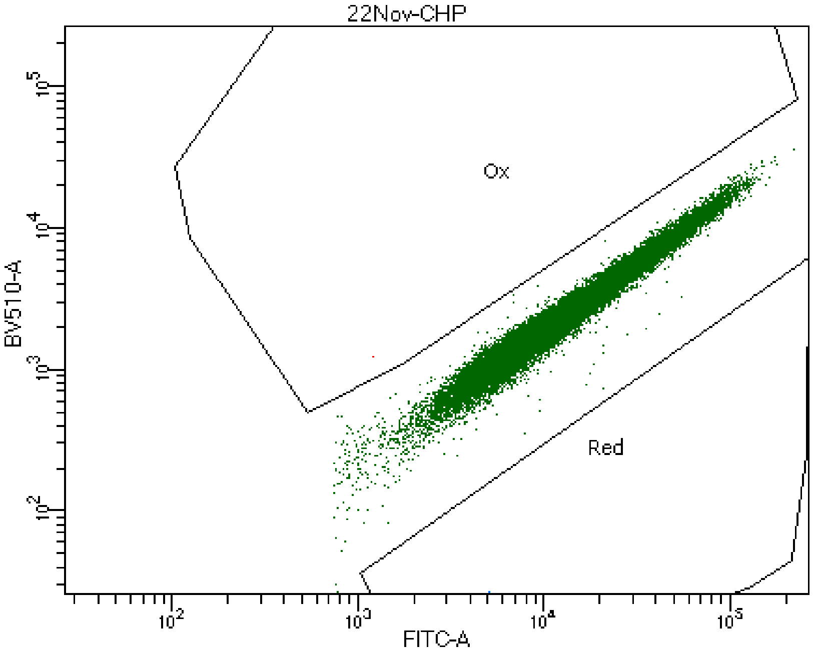

Tube: CHP

Population	#Events	%Parent	%Total
All Events	66,284	####	100.0
P	56,534	85.3	85.3
S1	54,350	96.1	82.0
S2	53,907	99.2	81.3
S3	48,940	90.8	73.8
S4	45,298	92.6	68.3
GFP+	44,582	98.4	67.3
Ox	1	0.0	0.0
Red	1	0.0	0.0

Experiment Name: 21Nov2016 Bac sorting
Specimen Name: 22Nov
Tube Name: CHP
Record Date: Nov 22, 2016 2:15:51 PM
$OP: Administrator
GUID: 3120bf0a-a58c-44c6-a53e-97e...

Population	#Events	%Parent	FITC-A Median	BV510-A Median
S4	45,298	92.6	10,022	1,886
GFP+	44,582	98.4	10,177	1,910
Ox	1	0.0	1,176	1,231
Red	1	0.0	5,048	1

BD FACSDiva 8.0.1

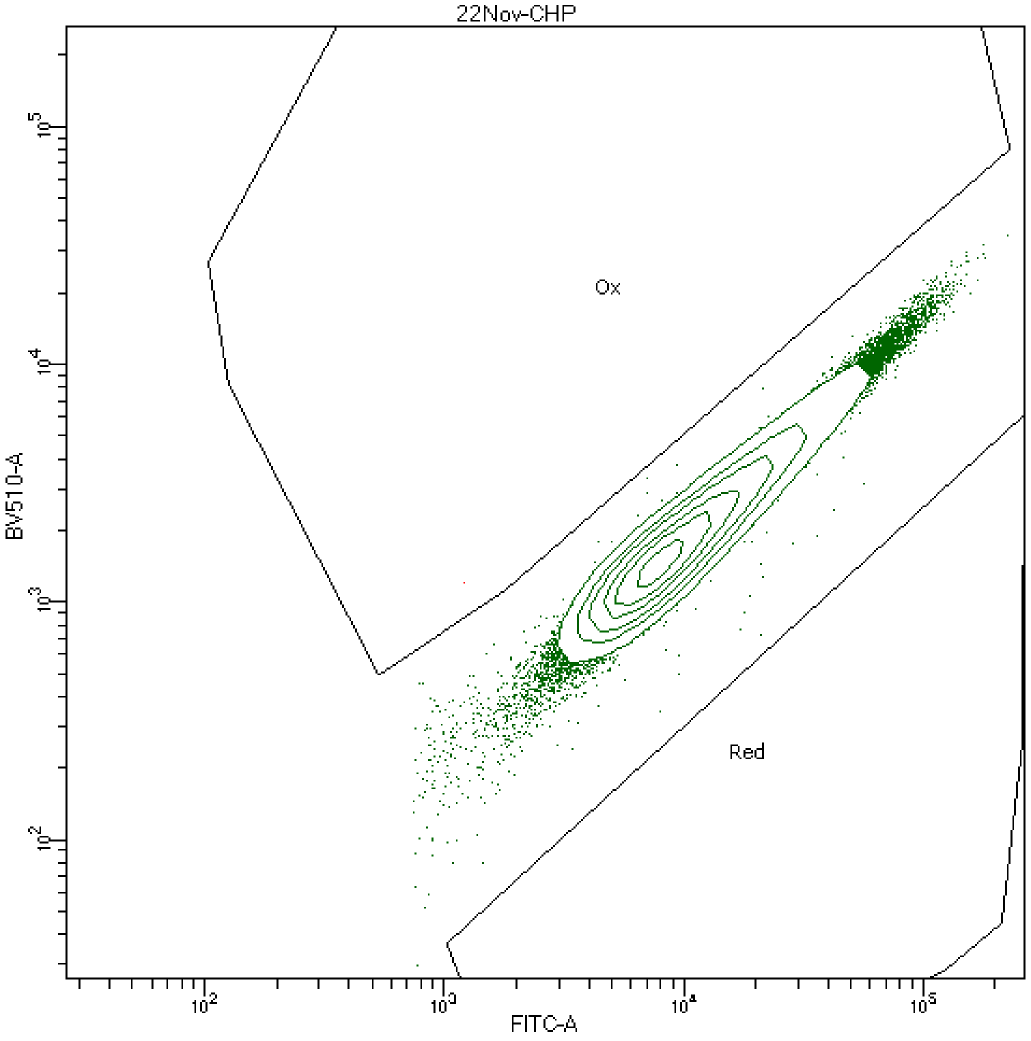

BD FACSDiva 8.0.1

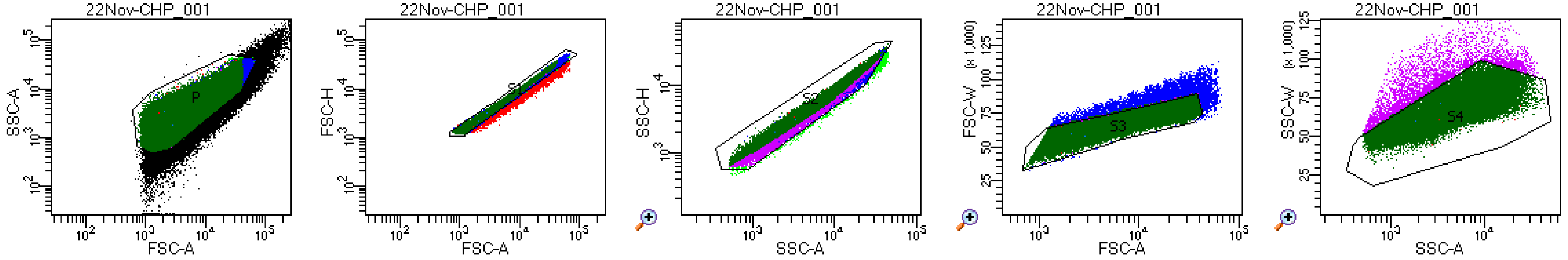

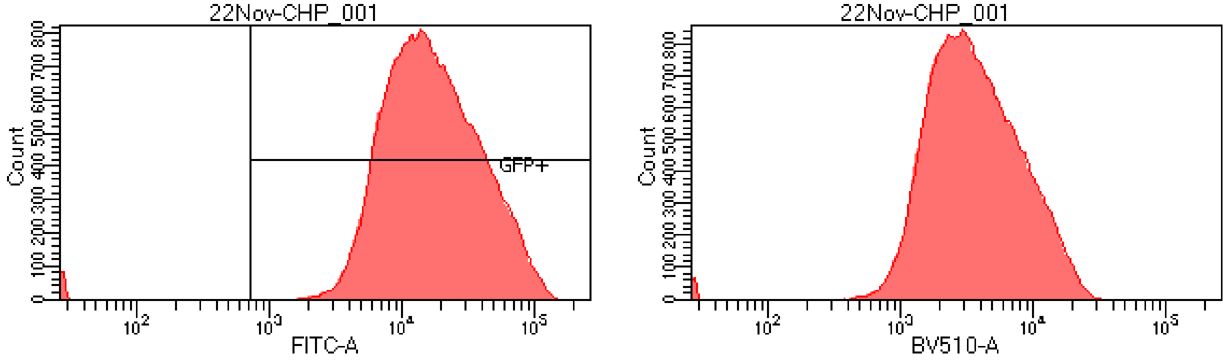

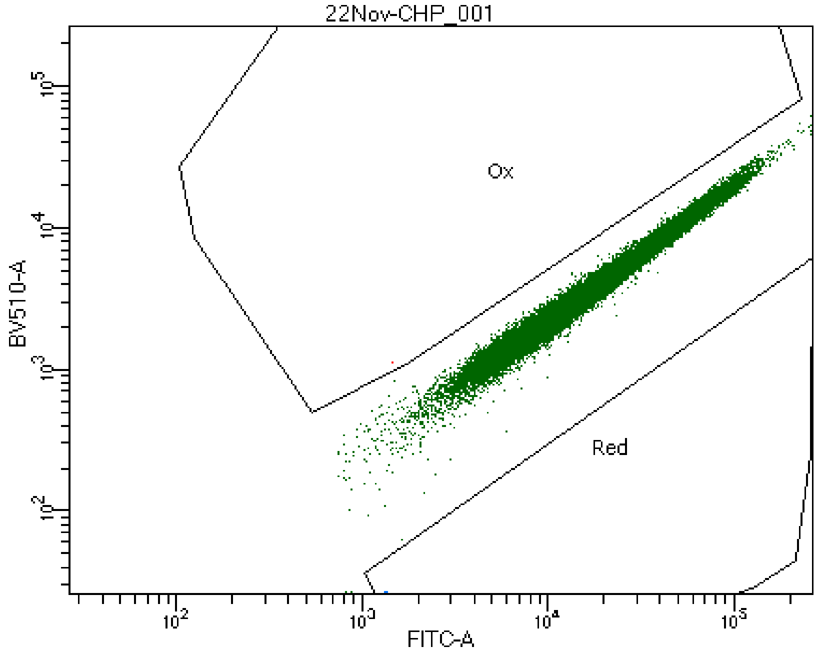

Tube: CHP_001

Population	#Events	%Parent	%Total
All Events	71,712	####	100.0
P	59,410	82.8	82.8
S1	56,740	95.5	79.1
S2	56,019	98.7	78.1
S3	50,959	91.0	71.1
S4	47,550	93.3	66.3
GFP+	47,237	99.3	65.9
Ox	1	0.0	0.0
Red	2	0.0	0.0

Experiment Name:	21Nov2016 Bac sorting
Specimen Name:	22Nov
Tube Name:	CHP_001
Record Date:	Nov 22, 2016 2:16:24 PM
$OP:	Administrator
GUID:	8f47f751-284c-4113-8ff0-8a146...

Population	#Events	%Parent	FITC-A Median	BV510-A Median
S4	47,550	93.3	15,447	3,260
GFP+	47,237	99.3	15,544	3,283
Ox	1	0.0	1,446	1,102
Red	2	0.0	1,325	-66

BD FACSDiva 8.0.1

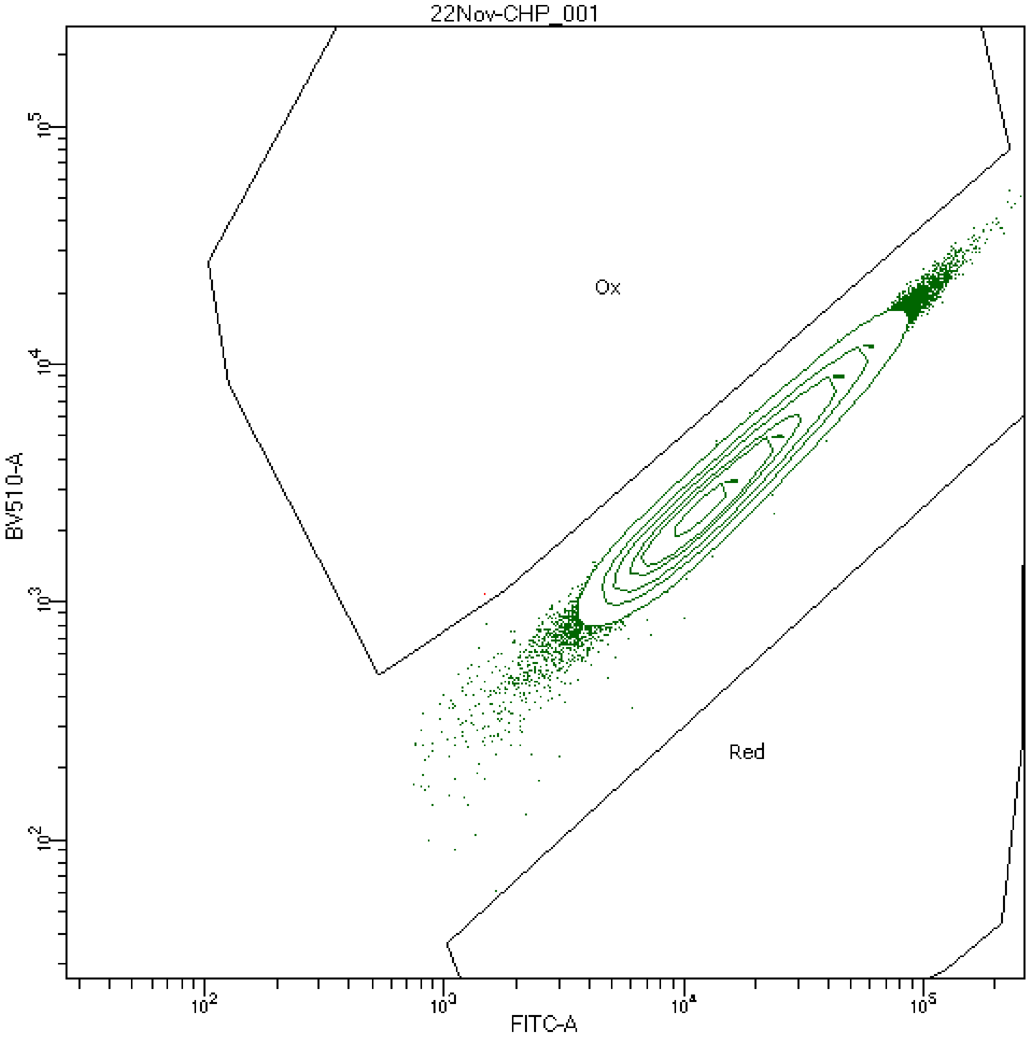

BD FACSDiva 8.0.1

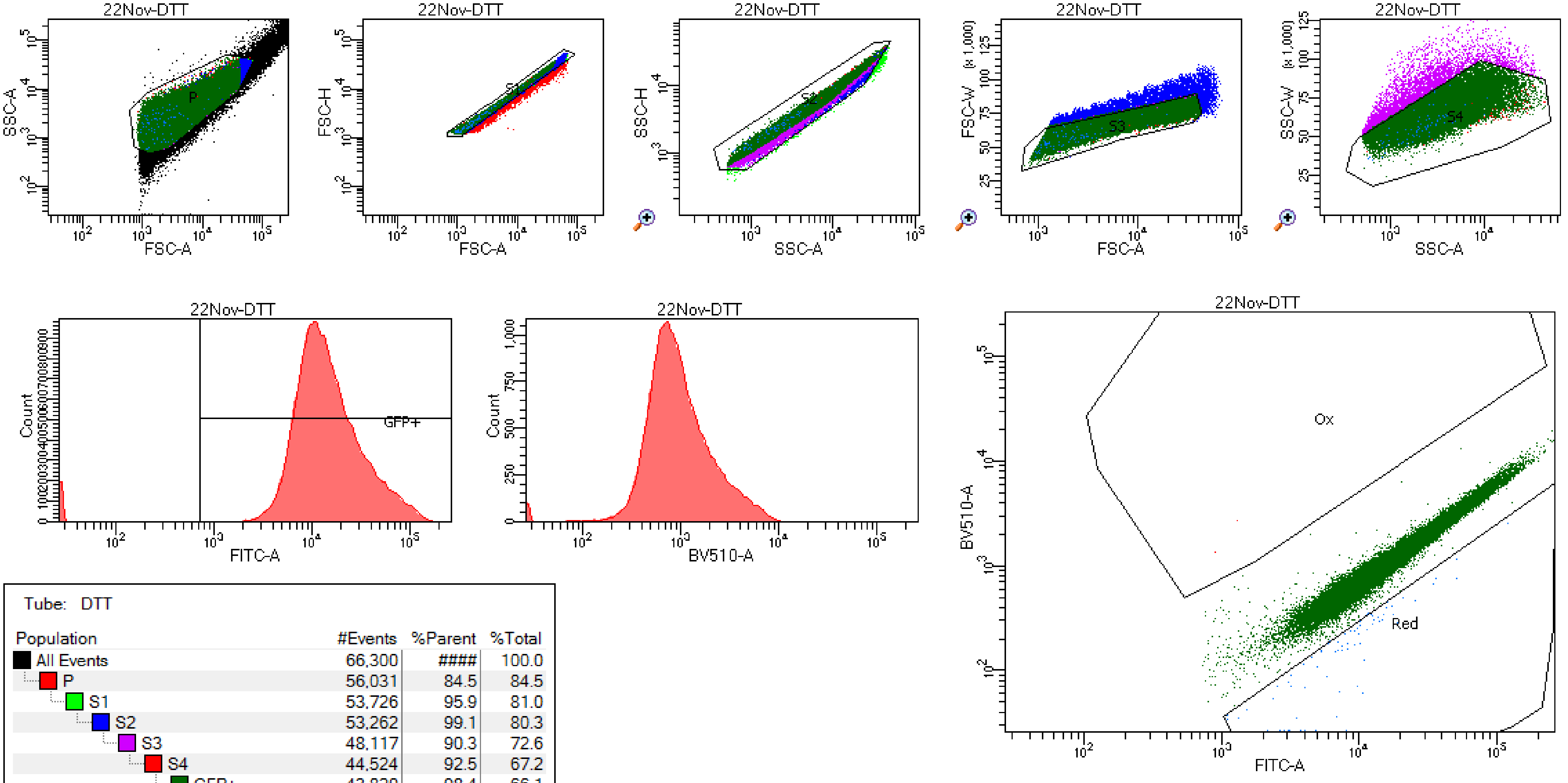

Tube: DTT

Population	#Events	%Parent	%Total
All Events	66,300	####	100.0
P	56,031	84.5	84.5
S1	53,726	95.9	81.0
S2	53,262	99.1	80.3
S3	48,117	90.3	72.6
S4	44,524	92.5	67.2
GFP+	43,820	98.4	66.1
Ox	2	0.0	0.0
Red	76	0.2	0.1

Experiment Name: 21Nov2016 Bac sorting
Specimen Name: 22Nov
Tube Name: DTT
Record Date: Nov 22, 2016 2:17:07 PM
$OP: Administrator
GUID: 196a0967-9d00-4588-a308-c65...

Population	#Events	%Parent	FITC-A Median	BV510-A Median
S4	44,524	92.5	12,696	832
GFP+	43,820	98.4	12,862	842
Ox	2	0.0	1,073	2,019
Red	76	0.2	8,154	176

BD FACSDiva 8.0.1

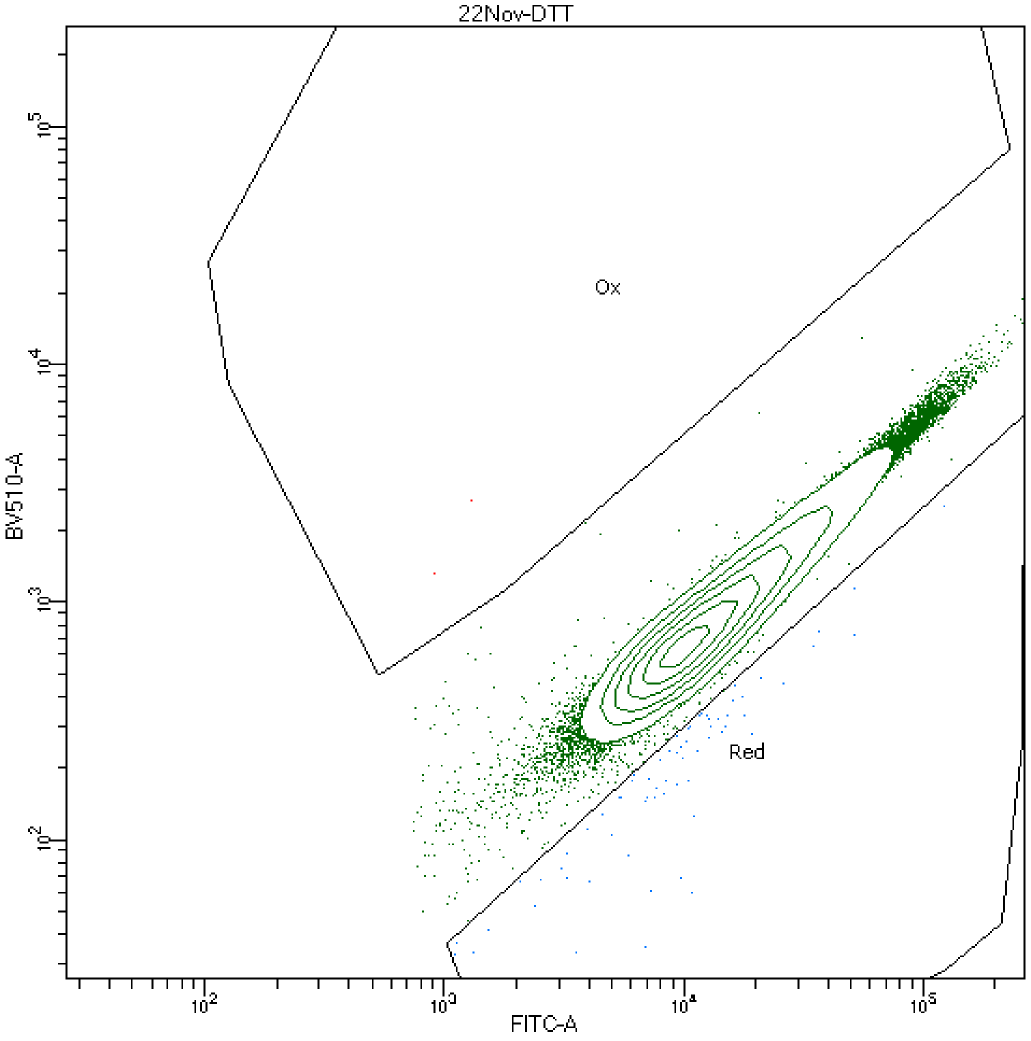

BD FACSDiva 8.0.1

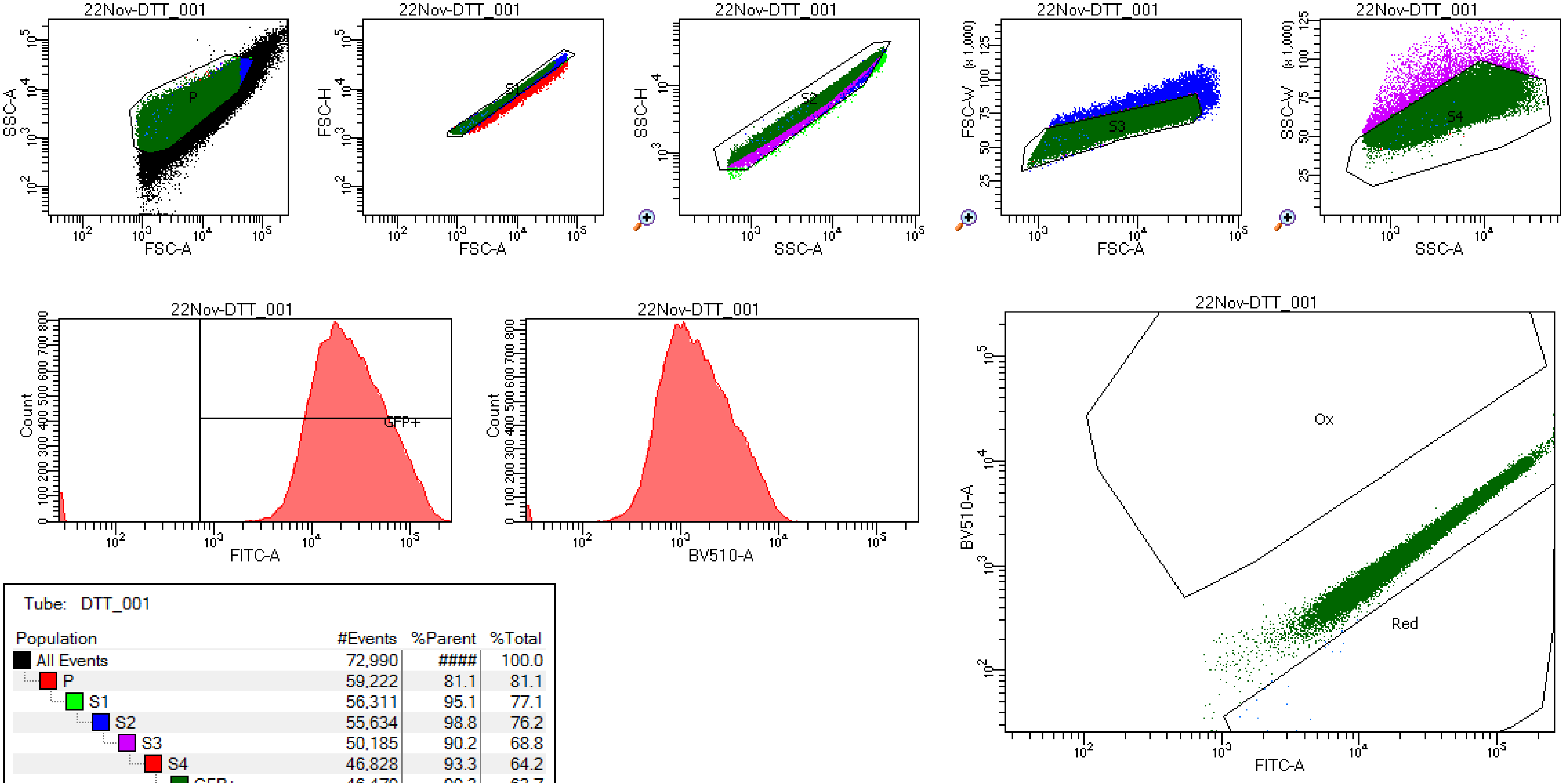

Tube: DTT_001

Population	#Events	%Parent	%Total
All Events	72,990	####	100.0
P	59,222	81.1	81.1
S1	56,311	95.1	77.1
S2	55,634	98.8	76.2
S3	50,185	90.2	68.8
S4	46,828	93.3	64.2
GFP+	46,479	99.3	63.7
Ox	0	0.0	0.0
Red	22	0.0	0.0

Experiment Name: 21Nov2016 Bac sorting
Specimen Name: 22Nov
Tube Name: DTT_001
Record Date: Nov 22, 2016 2:17:39 PM
$OP: Administrator
GUID: f9661cd8-4c75-4ea2-8426-18e...

Population	#Events	%Parent	FITC-A Median	BV510-A Median
S4	46,828	93.3	22,288	1,320
GFP+	46,479	99.3	22,499	1,332
Ox	0	0.0	####	####
Red	22	0.0	3,912	89

BD FACSDiva 8.0.1

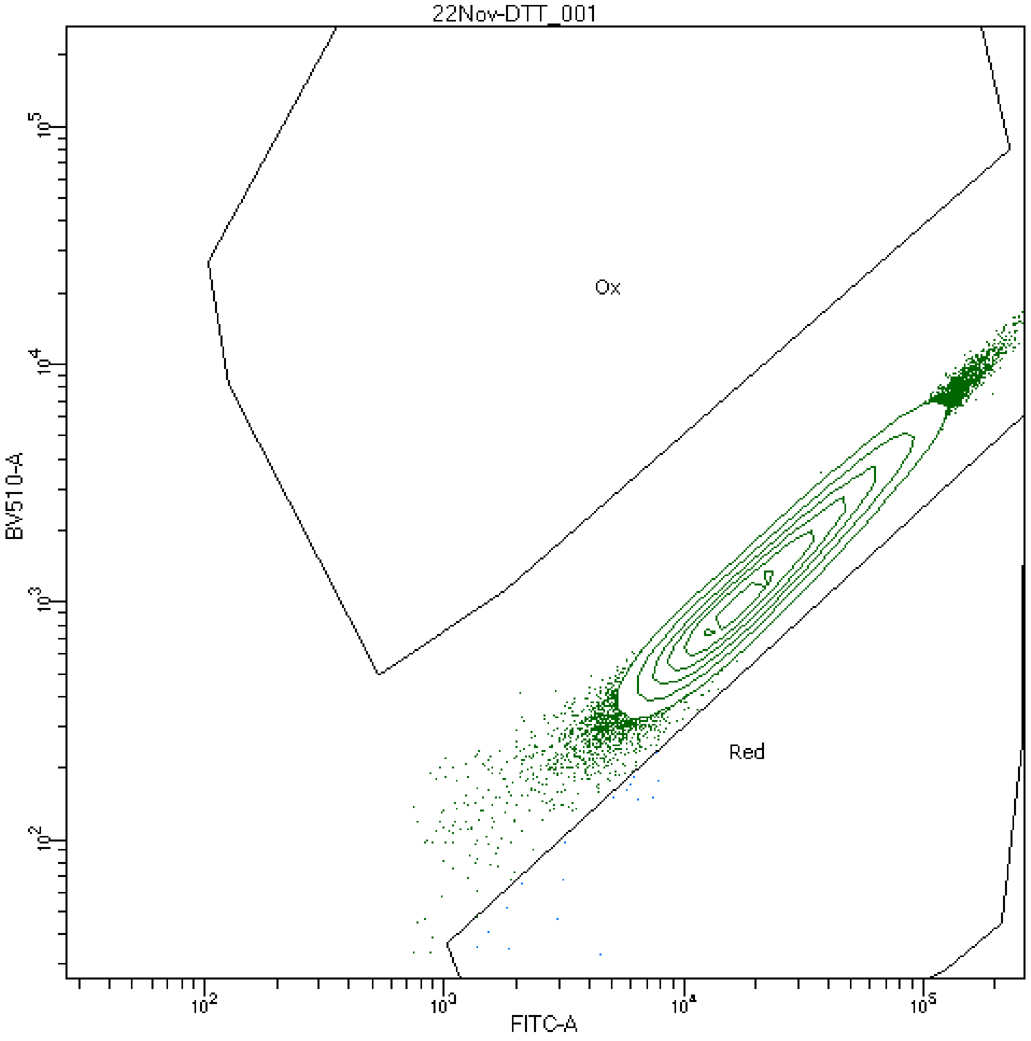

BD FACSDiva 8.0.1

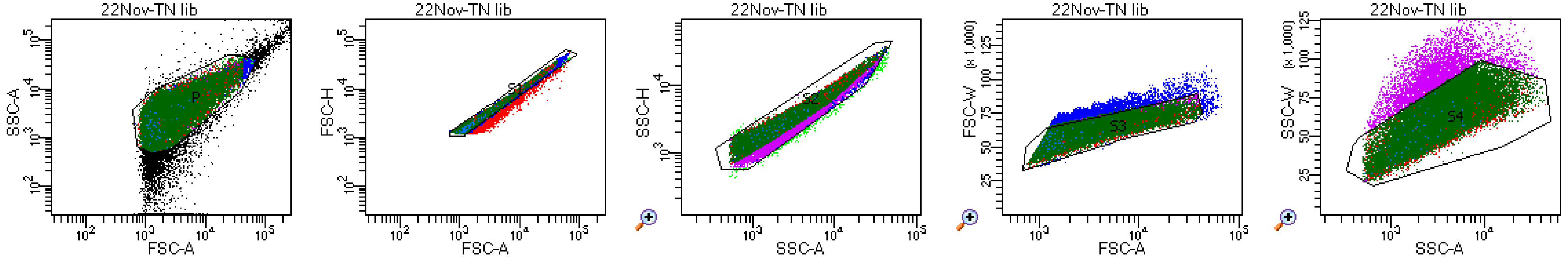

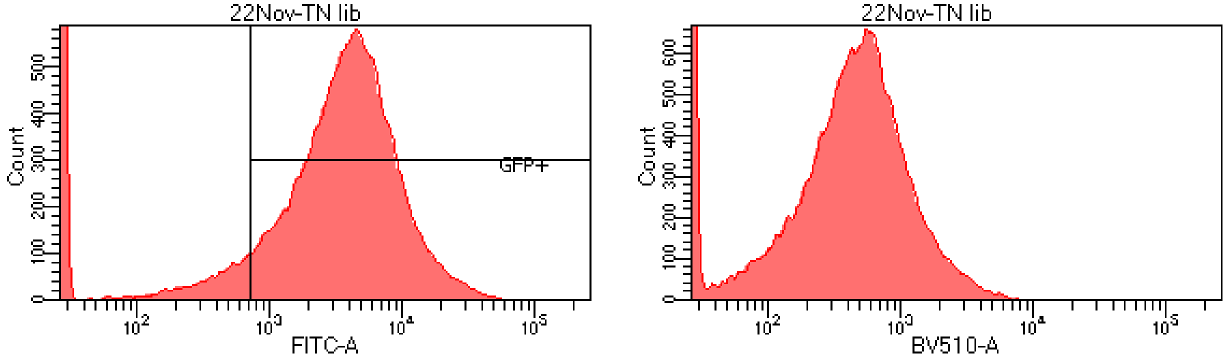

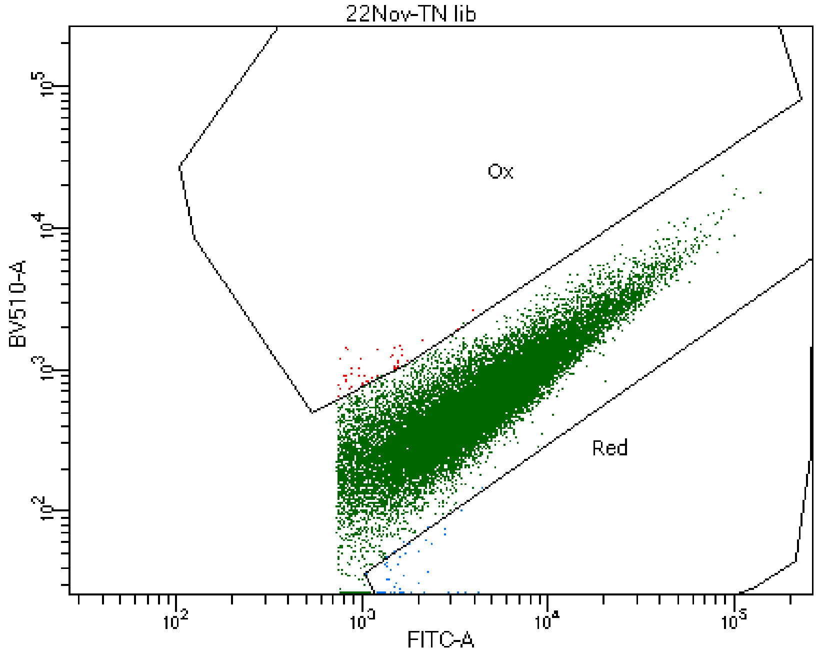

Tube: TN lib

Population	#Events	%Parent	%Total
All Events	58,178	####	100.0
P	53,117	91.3	91.3
S1	49,332	92.9	84.8
S2	48,914	99.2	84.1
S3	43,661	89.3	75.0
S4	38,829	88.9	66.7
GFP+	29,805	76.8	51.2
Ox	45	0.2	0.1
Red	56	0.2	0.1

Experiment Name:	21Nov2016 Bac sorting
Specimen Name:	22Nov
Tube Name:	TN lib
Record Date:	Nov 22, 2016 2:18:23 PM
$OP:	Administrator
GUID:	ce5e6ea0-bacc-415d-88e6-341...

Population	#Events	%Parent	FITC-A Median	BV510-A Median
S4	38,829	88.9	2,994	419
GFP+	29,805	76.8	4,055	538
Ox	45	0.2	1,021	1,006
Red	56	0.2	1,571	32

BD FACSDiva 8.0.1

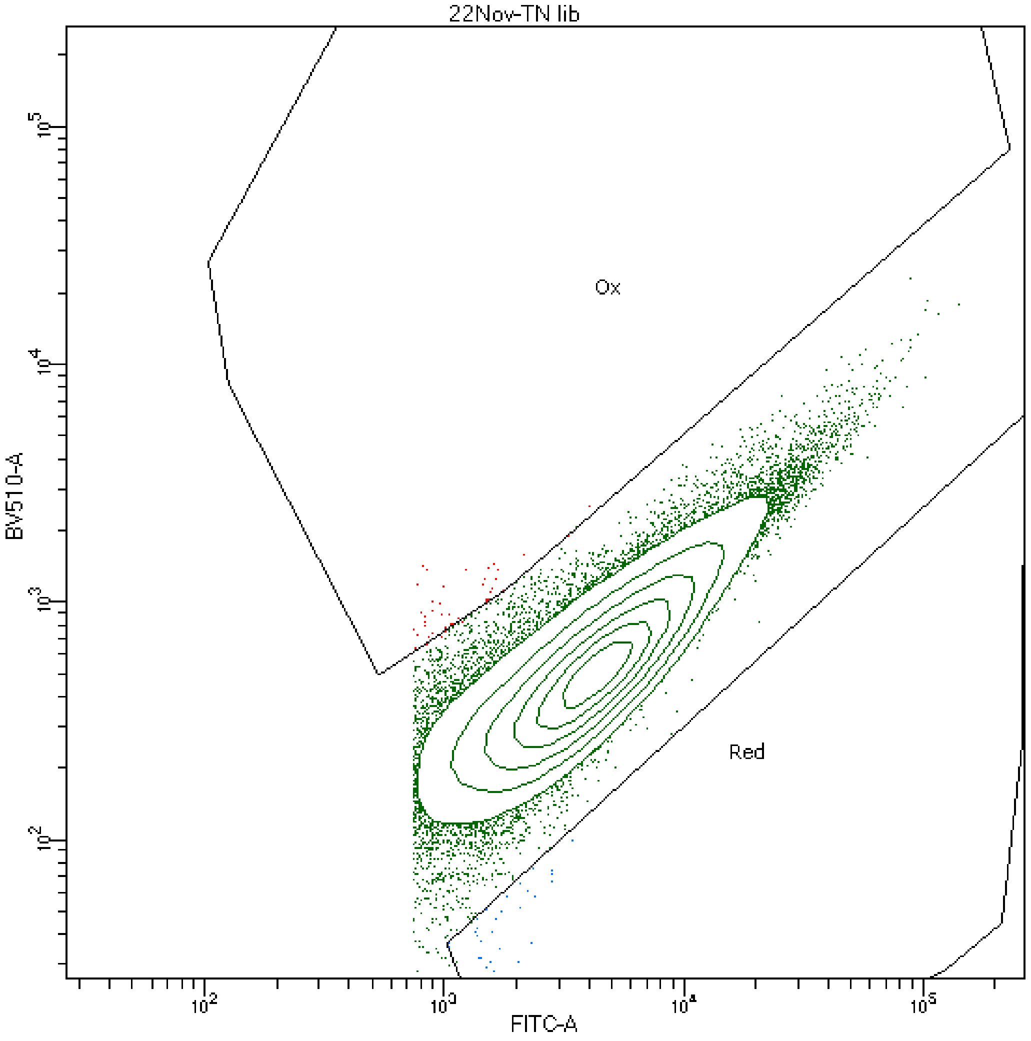

BD FACSDiva 8.0.1

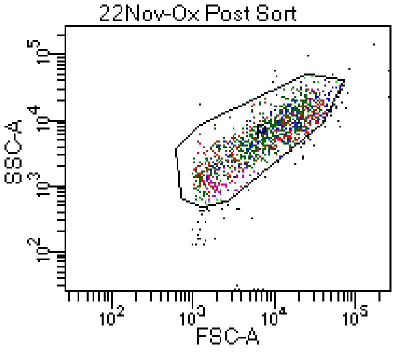

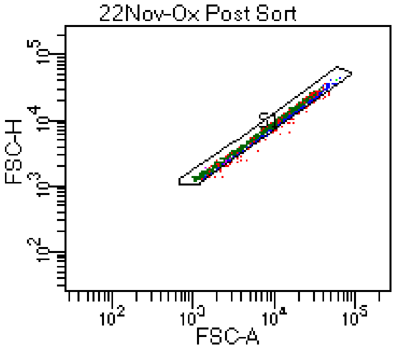

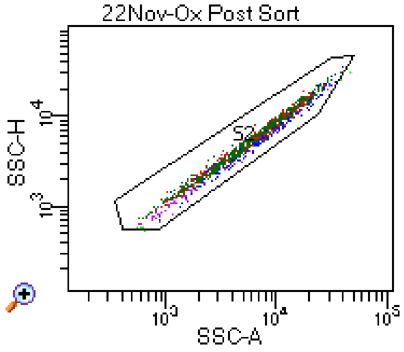

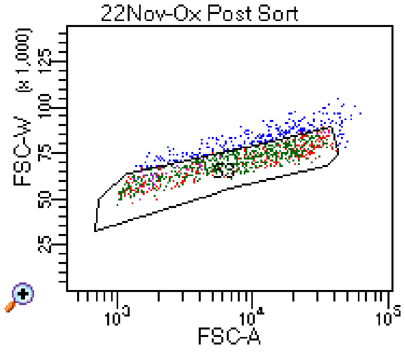

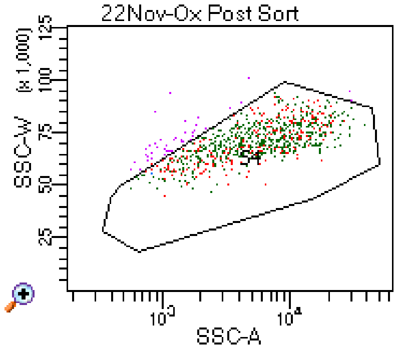

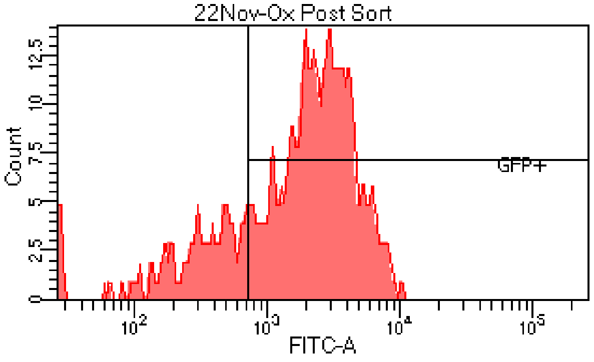

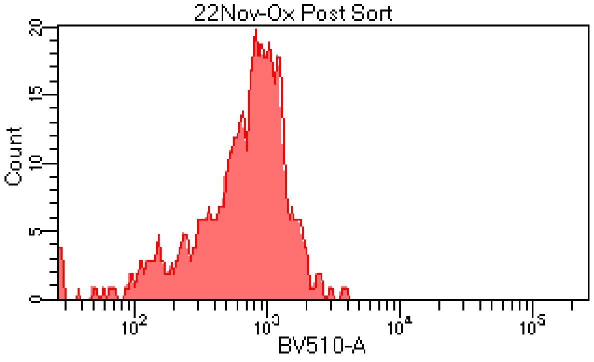

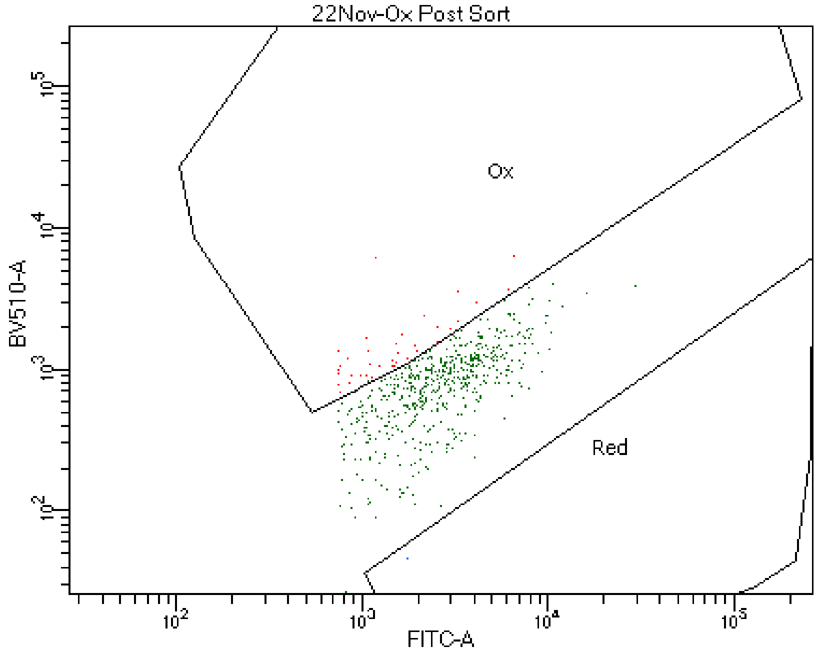

Tube: Ox Post Sort

Population	#Events	%Parent	%Total
All Events	1,246	####	100.0
P	1,156	92.8	92.8
S1	1,101	95.2	88.4
S2	1,093	99.3	87.7
S3	843	77.1	67.7
S4	799	94.8	64.1
GFP+	589	73.7	47.3
Ox	39	6.6	3.1
Red	1	0.2	0.1

Experiment Name: 21Nov2016 Bac sorting
Specimen Name: 22Nov
Tube Name: Ox Post Sort
Record Date: Nov 22, 2016 4:43:05 PM
$OP: Administrator
GUID: 55af95fe-3ca7-40d4-aa93-41ad...

Population	#Events	%Parent	FITC-A Median	BV510-A Median
S4	799	94.8	1,866	722
GFP+	589	73.7	2,527	851
Ox	39	6.6	1,432	1,190
Red	1	0.2	1,739	46

BD FACSDiva 8.0.1

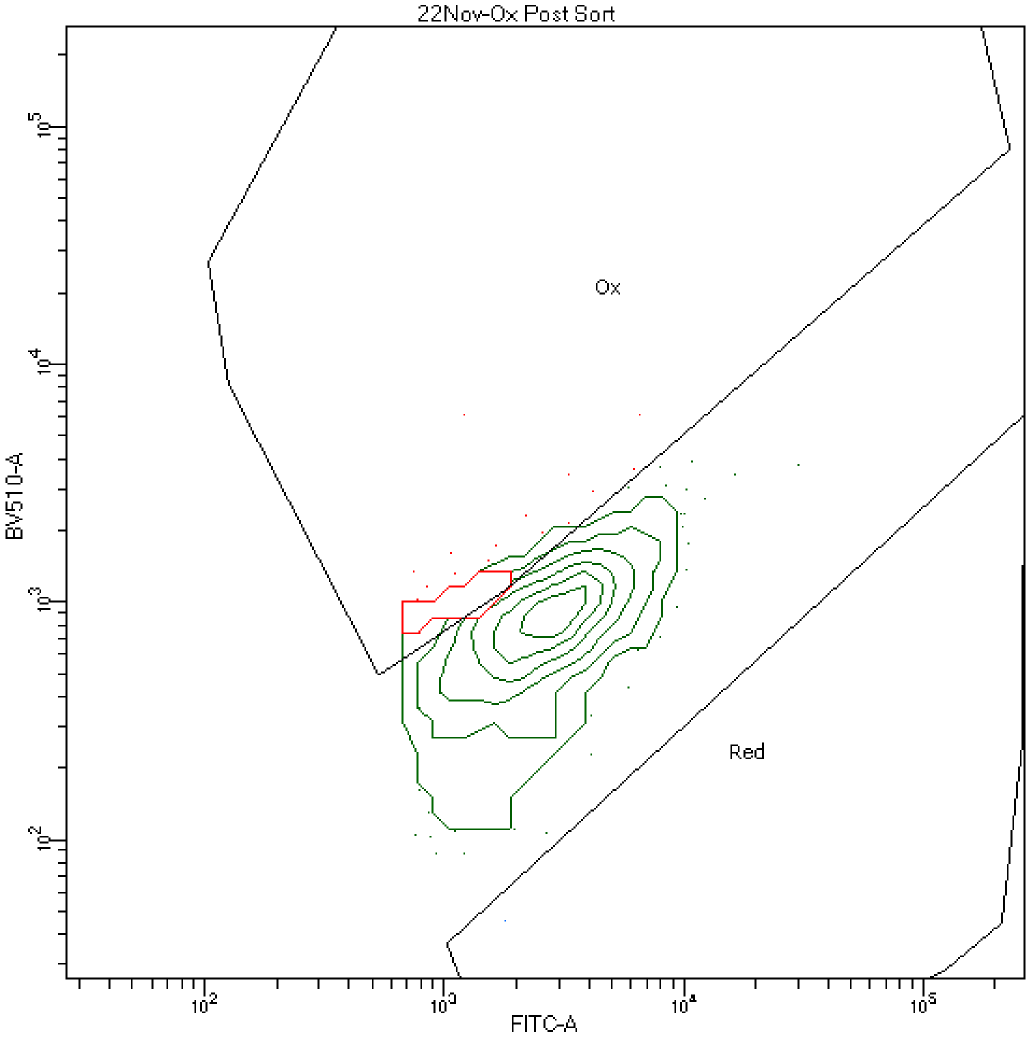

BD FACSDiva 8.0.1

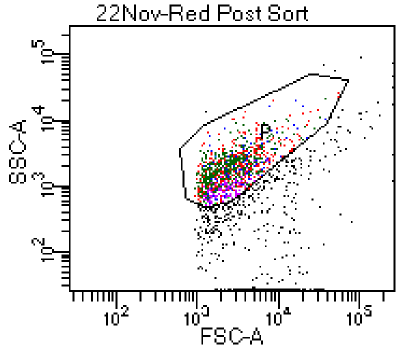

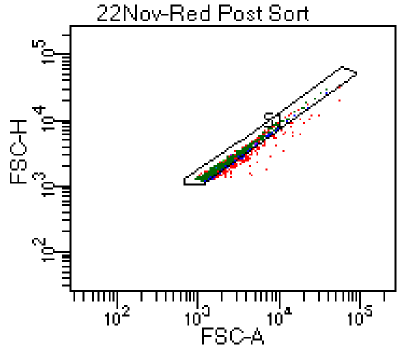

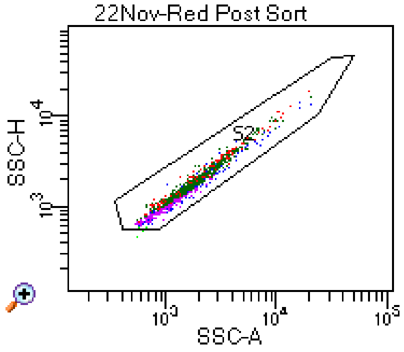

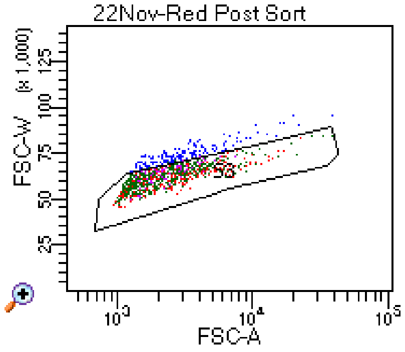

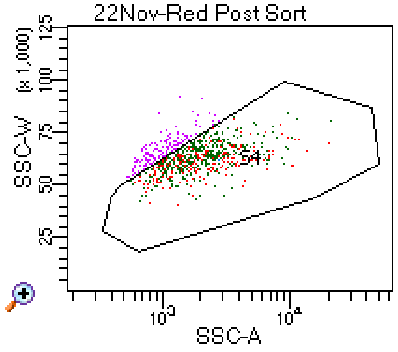

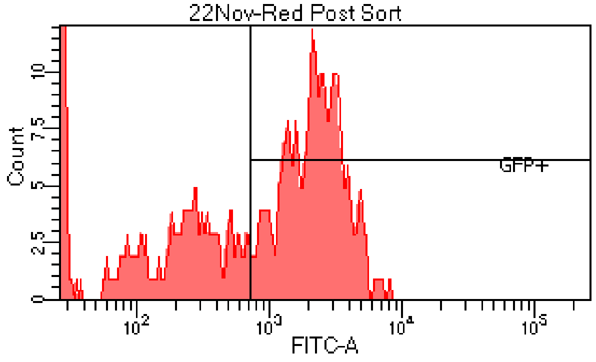

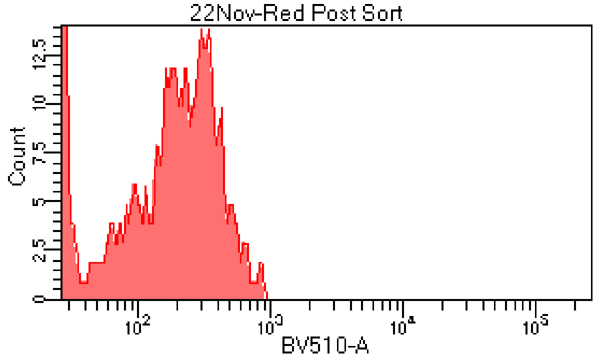

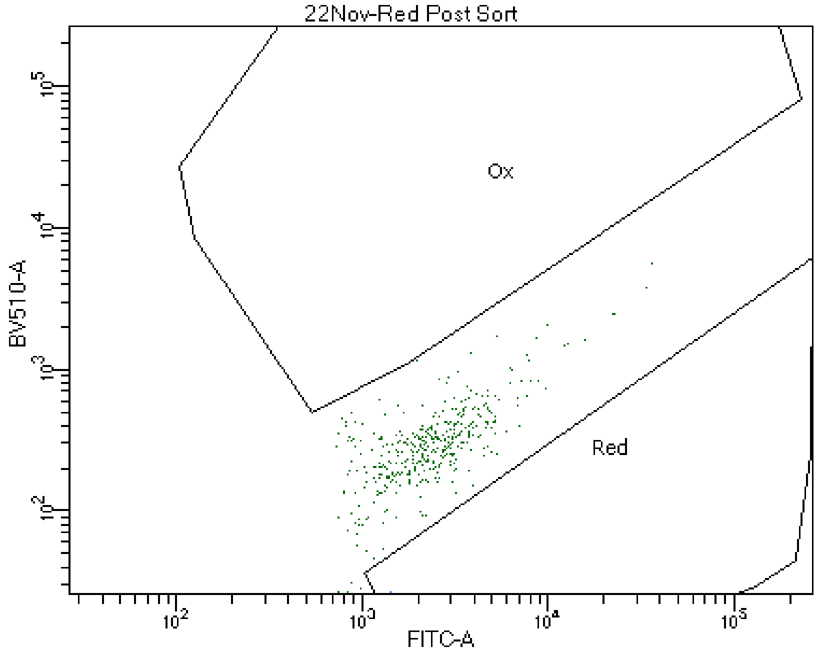

Tube: Red Post Sort

Population	#Events	%Parent	%Total
All Events	1,803	####	100.0
P	1,158	64.2	64.2
S1	1,022	88.3	56.7
S2	1,016	99.4	56.4
S3	848	83.5	47.0
S4	698	82.3	38.7
GFP+	395	56.6	21.9
Ox	0	0.0	0.0
Red	1	0.3	0.1

Experiment Name:	21Nov2016 Bac sorting
Specimen Name:	22Nov
Tube Name:	Red Post Sort
Record Date:	Nov 22, 2016 4:43:47 PM
$OP:	Administrator
GUID:	8158858b-cd1a-47b0-aafc-6e3...

Population	#Events	%Parent	FITC-A Median	BV510-A Median
S4	698	82.3	1,127	187
GFP+	395	56.6	2,213	289
Ox	0	0.0	####	####
Red	1	0.3	1,413	-5

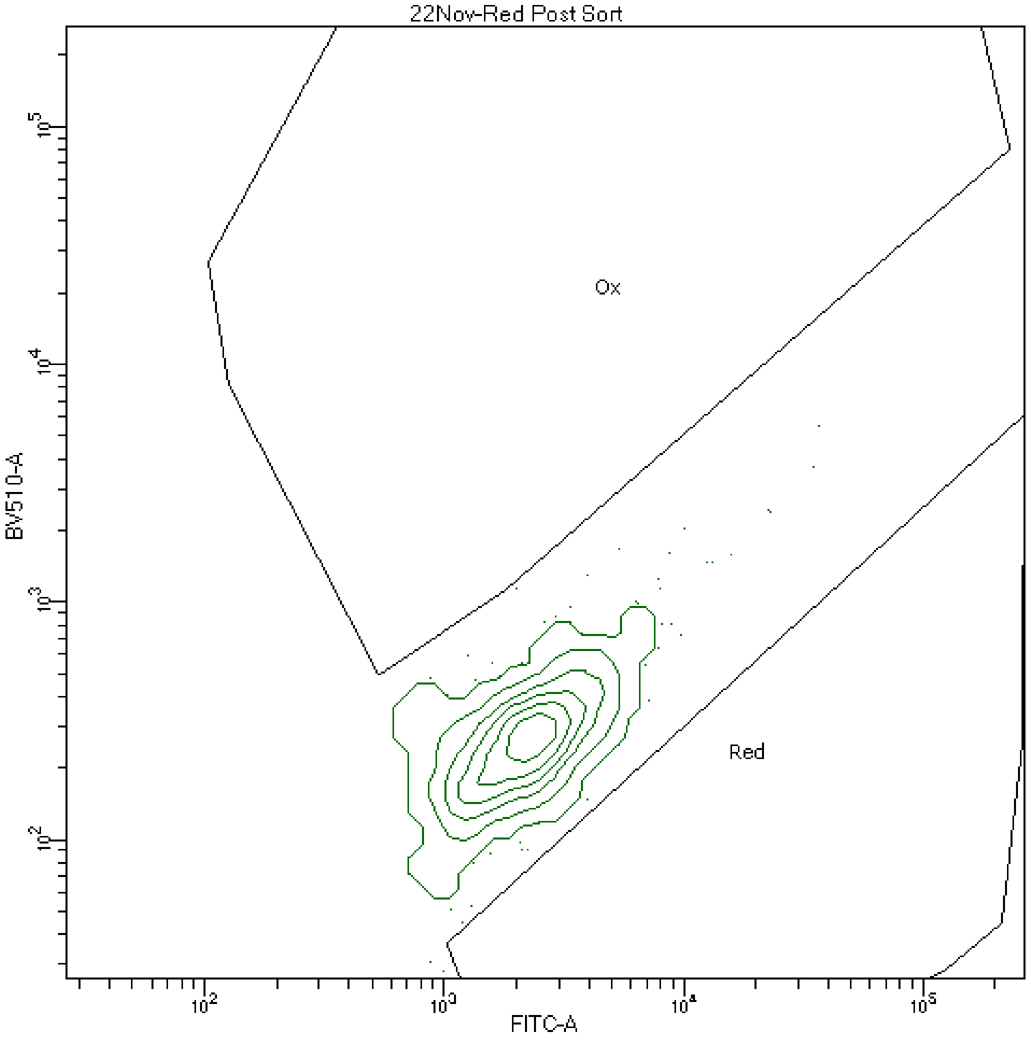
22Nov-Red Post Sort
Ox
Red
BV510-A
FITC-A
10^2
10^3
10^4
10^5
