# Supplementary material for: Biosensor-integrated transposon mutagenesis reveals rv0158 as a coordinator of redox homeostasis in Mycobacterium tuberculosis
Source: eLife. 2023 Aug 29;12:e80218. doi: 10.7554/eLife.80218 (PMC10501769; doi:10.7554/eLife.80218)
Supplement: Figure 1—source data 1. [file elife-80218-fig1-data1.zip › Round 1 Sorting/Sort_Report_02022016154012.pdf]

Experiment : 02Feb2016 Bact Sorting	Sort Report	Report Date : 2016.02.02 at 15:39:24
Specimen : Specimen_001		Device : 4 Tube
Tube : TN40K_003		User ID : Administrator
Sort Layout : Sort Layout_001		Cytometer : FACSAriaIII (P65828254001)
Application : FACSDiva Version 8.0.1		

Sort Settings

Sort Setup	70 micron	Precision	4-Way Purity
Frequency	90.9	Yield Mask	0
Amplitude	6.6	Purity Mask	32
Phase	0.00	Phase Mask	0
Drop Delay	46.40	Single Cell	Off
Attenuation	Off	Plates Voltage	5,500
Sweet Spot	On	Voltage Centering	6
First Drop	210	Sheath Pressure	70.00
Target Gap	6		

Side Stream Voltage (%)

Far Left	Left	Right	Far Right
87.00	34.00	30.00	82.00

Neighboring Drop Charge (%)

2nd	3rd	4th
18.00	9.00	1.00

Acquisition Counters

Threshold Count	2462399
Processed Events Count(evt)	2215971
Electronic Aborts Count(evt)	19511
Sort Elapsed Time(hh:mm:ss)	00:15:04

Sort Counters

	Far Left	Left	Right	Far Right
Sort Rate(evt/s)	0	NA	NA	NA
Conflicts Count(evt)	1084	NA	NA	NA
Conflicts Rate(evt/s)	0	NA	NA	NA
Efficiency(%)	0	NA	NA	NA

Sort Layout

Far Left	Left	Right	Far Right
P5 : 14760			
