# Supplementary material for: Biosensor-integrated transposon mutagenesis reveals rv0158 as a coordinator of redox homeostasis in Mycobacterium tuberculosis
Source: eLife. 2023 Aug 29;12:e80218. doi: 10.7554/eLife.80218 (PMC10501769; doi:10.7554/eLife.80218)
Supplement: Figure 1—source data 1. [file elife-80218-fig1-data1.zip › Round 1 Sorting/Sort_Report_16052016154412.pdf]

Experiment : 16May2016 Bac sorting	Sort Report	Report Date : 2016.05.16 at 15:14:39
Specimen : Specimen_001		Device : 4 Tube
Tube : TN40k		User ID : Administrator
Sort Layout : Sort Layout_006		Cytometer : FACSAriaIII (P65828254001)
Application : FACSDiva Version 8.0.1		

Sort Settings

Sort Setup	70 micron	Precision	4-Way Purity
Frequency	89.8	Yield Mask	0
Amplitude	6.1	Purity Mask	32
Phase	0.00	Phase Mask	0
Drop Delay	45.01	Single Cell	Off
Attenuation	Off	Plates Voltage	6,000
Sweet Spot	On	Voltage Centering	6
First Drop	171	Sheath Pressure	70.00
Target Gap	6		

Side Stream Voltage (%)

Far Left	Left	Right	Far Right
93.00	38.00	27.00	82.00

Neighboring Drop Charge (%)

2nd	3rd	4th
18.00	6.00	0.00

Acquisition Counters

Threshold Count	1470842
Processed Events Count(evt)	1499887
Electronic Aborts Count(evt)	1958
Sort Elapsed Time(hh:mm:ss)	00:35:20

Sort Counters

	Far Left	Left	Right	Far Right
Sort Rate(evt/s)	NA	0	NA	NA
Conflicts Count(evt)	NA	18	NA	NA
Conflicts Rate(evt/s)	NA	0	NA	NA
Efficiency(%)	NA	0	NA	NA

Sort Layout

Far Left	Left	Right	Far Right
	P5 : 2134		
