# Supplementary material for: Biosensor-integrated transposon mutagenesis reveals rv0158 as a coordinator of redox homeostasis in Mycobacterium tuberculosis
Source: eLife. 2023 Aug 29;12:e80218. doi: 10.7554/eLife.80218 (PMC10501769; doi:10.7554/eLife.80218)
Supplement: Figure 1—source data 1. [file elife-80218-fig1-data1.zip › Round 1 Sorting/Sort_Report_16052016154421.pdf]

Experiment : 16May2016 Bac sorting
Specimen : Specimen_001
Tube : TN40k
Sort Layout : Sort Layout_006
Application : FACSDiva Version 8.0.1

Sort Report

Report Date : 2016.05.16 at 12:59:06
Device : 4 Tube
User ID : Administrator
Cytometer : FACSAriaIII (P65828254001)

Sort Settings

Sort Setup	70 micron	Precision	4-Way Purity
Frequency	89.1	Yield Mask	0
Amplitude	5.9	Purity Mask	32
Phase	0.00	Phase Mask	0
Drop Delay	46.09	Single Cell	Off
Attenuation	Off	Plates Voltage	6,000
Sweet Spot	On	Voltage Centering	6
First Drop	183	Sheath Pressure	70.00
Target Gap	6		

Side Stream Voltage (%)

Far Left	Left	Right	Far Right
100.00	43.00	29.00	91.00

Neighboring Drop Charge (%)

2nd	3rd	4th
6.00	5.00	0.00

Acquisition Counters

Threshold Count	2270697
Processed Events Count(evt)	2433941
Electronic Aborts Count(evt)	4190
Sort Elapsed Time(hh:mm:ss)	00:57:28

Sort Counters

	Far Left	Left	Right	Far Right
Sort Rate(evt/s)	NA	0	NA	NA
Conflicts Count(evt)	NA	34	NA	NA
Conflicts Rate(evt/s)	NA	0	NA	NA
Efficiency(%)	NA	0	NA	NA

Sort Layout

Far Left	Left	Right	Far Right
	P5 : 3197		
