# Supplementary material for: Biosensor-integrated transposon mutagenesis reveals rv0158 as a coordinator of redox homeostasis in Mycobacterium tuberculosis
Source: eLife. 2023 Aug 29;12:e80218. doi: 10.7554/eLife.80218 (PMC10501769; doi:10.7554/eLife.80218)
Supplement: Figure 1—source data 1. [file elife-80218-fig1-data1.zip › Round 1 Sorting/Sort_Report_22112016164403.pdf]

Experiment : 21Nov2016 Bac sorting	Sort Report	Report Date : 2016.11.22 at 16:40:29
Specimen : 22Nov		Device : 4 Tube
Tube : TN lib		User ID : Administrator
Sort Layout : Sort Layout_001		Cytometer : FACSAriaIII (P65828254001)
Application : FACSDiva Version 8.0.1		

Sort Settings

Sort Setup	70 micron	Precision	Yield
Frequency	88.3	Yield Mask	32
Amplitude	4.5	Purity Mask	0
Phase	0.00	Phase Mask	0
Drop Delay	44.65	Single Cell	Off
Attenuation	Off	Plates Voltage	4,000
Sweet Spot	On	Voltage Centering	6
First Drop	210	Sheath Pressure	70.00
Target Gap	6		

Side Stream Voltage (%)

Far Left	Left	Right	Far Right
0.00	56.00	43.00	0.00

Neighboring Drop Charge (%)

2nd	3rd	4th
18.00	8.00	0.00

Acquisition Counters

Threshold Count	90050439
Processed Events Count(evt)	89667398
Electronic Aborts Count(evt)	996883
Sort Elapsed Time(hh:mm:ss)	02:11:57

Sort Counters

	Far Left	Left	Right	Far Right
Sort Rate(evt/s)	NA	31	12	NA
Conflicts Count(evt)	NA	0	0	NA
Conflicts Rate(evt/s)	NA	0	0	NA
Efficiency(%)	NA	100	100	NA

Sort Layout

Far Left	Left	Right	Far Right
	Ox : 246261	Red : 99093	
