# Supplementary material for: Biosensor-integrated transposon mutagenesis reveals rv0158 as a coordinator of redox homeostasis in Mycobacterium tuberculosis
Source: eLife. 2023 Aug 29;12:e80218. doi: 10.7554/eLife.80218 (PMC10501769; doi:10.7554/eLife.80218)
Supplement: Figure 1—source data 1. [file elife-80218-fig1-data1.zip › Round 1 Sorting/Sort_Report_22112016164432.pdf]

Experiment : 24Oct2016 Sorting Macro	Sort Report	Report Date : 2016.10.24 at 16:34:57
Specimen : Specimen_001		Device : 2 Tube
Tube : TN lib		User ID : Administrator
Sort Layout : Sort Layout_002		Cytometer : FACSAriaIII (P65828254001)
Application : FACSDiva Version 8.0.1		

Sort Settings

Sort Setup	100 micron	Precision	4-Way Purity
Frequency	43.0	Yield Mask	0
Amplitude	5.6	Purity Mask	32
Phase	0.00	Phase Mask	0
Drop Delay	38.90	Single Cell	Off
Attenuation	Off	Plates Voltage	3,500
Sweet Spot	On	Voltage Centering	-129
First Drop	233	Sheath Pressure	20.00
Target Gap	10		

Side Stream Voltage (%)

Far Left	Left	Right	Far Right
0.00	24.00	20.00	0.00

Neighboring Drop Charge (%)

2nd	3rd	4th
18.00	6.00	1.00

Acquisition Counters

Threshold Count	27618580
Processed Events Count(evt)	27262911
Electronic Aborts Count(evt)	675681
Sort Elapsed Time(hh:mm:ss)	01:45:41

Sort Counters

	Left	Right
Sort Rate(evt/s)	0	0
Conflicts Count(evt)	5022	74278
Conflicts Rate(evt/s)	0	0
Efficiency(%)	100	66

Sort Layout

Left	Right
Ox : 10566	Red : 29553
