# Supplementary material for: Biosensor-integrated transposon mutagenesis reveals rv0158 as a coordinator of redox homeostasis in Mycobacterium tuberculosis
Source: eLife. 2023 Aug 29;12:e80218. doi: 10.7554/eLife.80218 (PMC10501769; doi:10.7554/eLife.80218)

Batch Analysis Report

Run Date: 12/5/16 4:30 PM Experiment: 05EDec2016 Bac sorting

User ID: Administrator

Statistics Output: N/A

Worksheet PDF Output: C:\Users\Admin\Desktop\05EDec2016 Bac sorting-Batch_Analysis_0512 2016163007.pdf

05Dec

Tube	Status	Run Time
US	OK	12/5/16 4:30 PM
RV Mrx1	OK	12/5/16 4:30 PM
RV Mrx1_001	OK	12/5/16 4:30 PM
CHP	OK	12/5/16 4:30 PM
CHP_001	OK	12/5/16 4:30 PM
DTT	OK	12/5/16 4:30 PM
DTT_001	OK	12/5/16 4:30 PM
TN lib	OK	12/5/16 4:30 PM
Ox Post Sort	OK	12/5/16 4:30 PM
Red Post Sort	OK	12/5/16 4:30 PM
Ox_001	OK	12/5/16 4:30 PM
Ox_002	OK	12/5/16 4:30 PM
Ox_003	OK	12/5/16 4:30 PM
CHP_01	OK	12/5/16 4:30 PM
CHP_02	OK	12/5/16 4:30 PM

BD FACSDiva 8.0.1

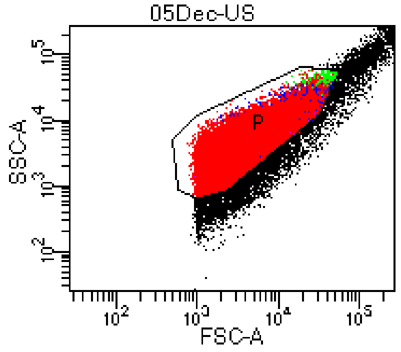

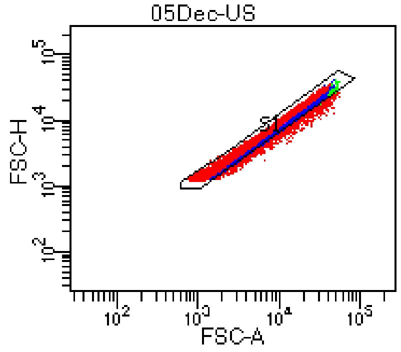

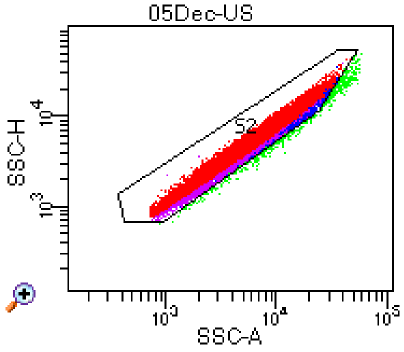

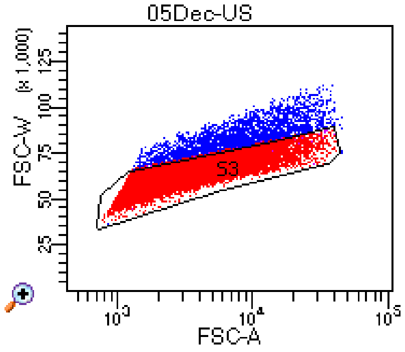

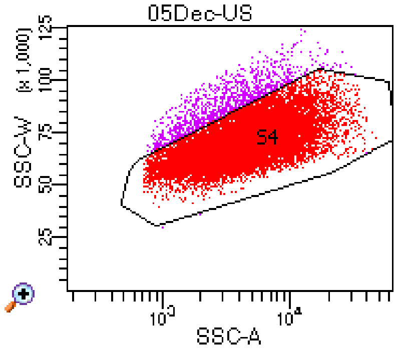

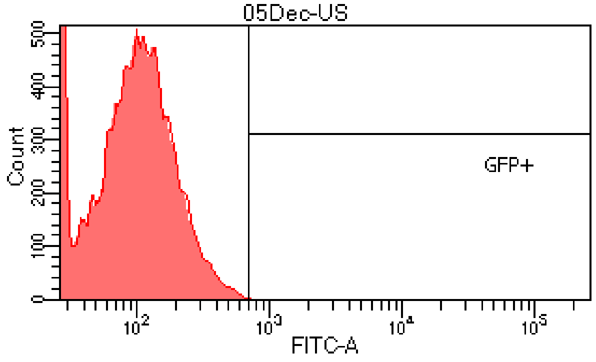

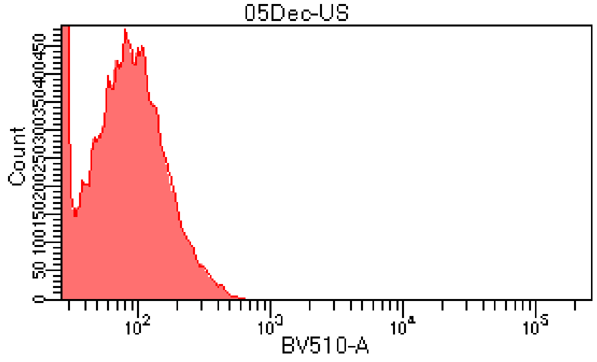

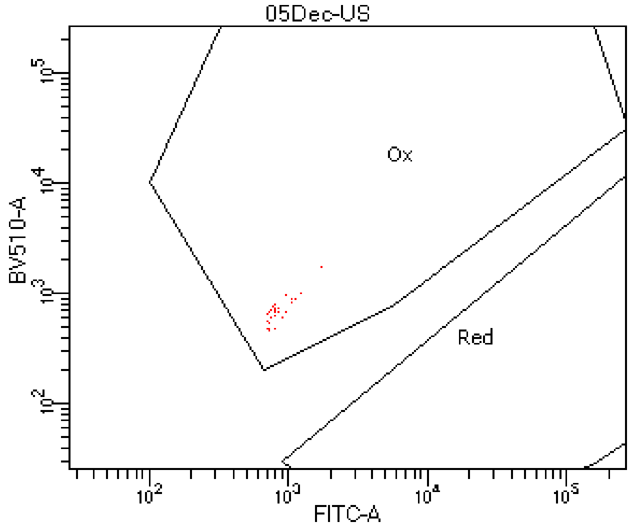

Tube: US

Population	#Events	%Parent	%Total
All Events	33,894	####	100.0
P	28,307	83.5	83.5
S1	27,040	95.5	79.8
S2	26,429	97.7	78.0
S3	23,363	88.4	68.9
S4	22,240	95.2	65.6
GFP+	27	0.1	0.1
Ox	27	100.0	0.1
Red	0	0.0	0.0

Experiment Name: 05EDec2016 Bac sorting
Specimen Name: 05Dec
Tube Name: US
Record Date: Dec 5, 2016 2:13:30 PM
$OP: Administrator
GUID: 6efe2ceb-978a-4869-9f54-ef0d...

Population	#Events	%Parent	FITC-A Median	BV510-A Median
S4	22,240	95.2	95	74
GFP+	27	0.1	786	678
Ox	27	100.0	786	678
Red	0	0.0	####	####

BD FACSDiva 8.0.1

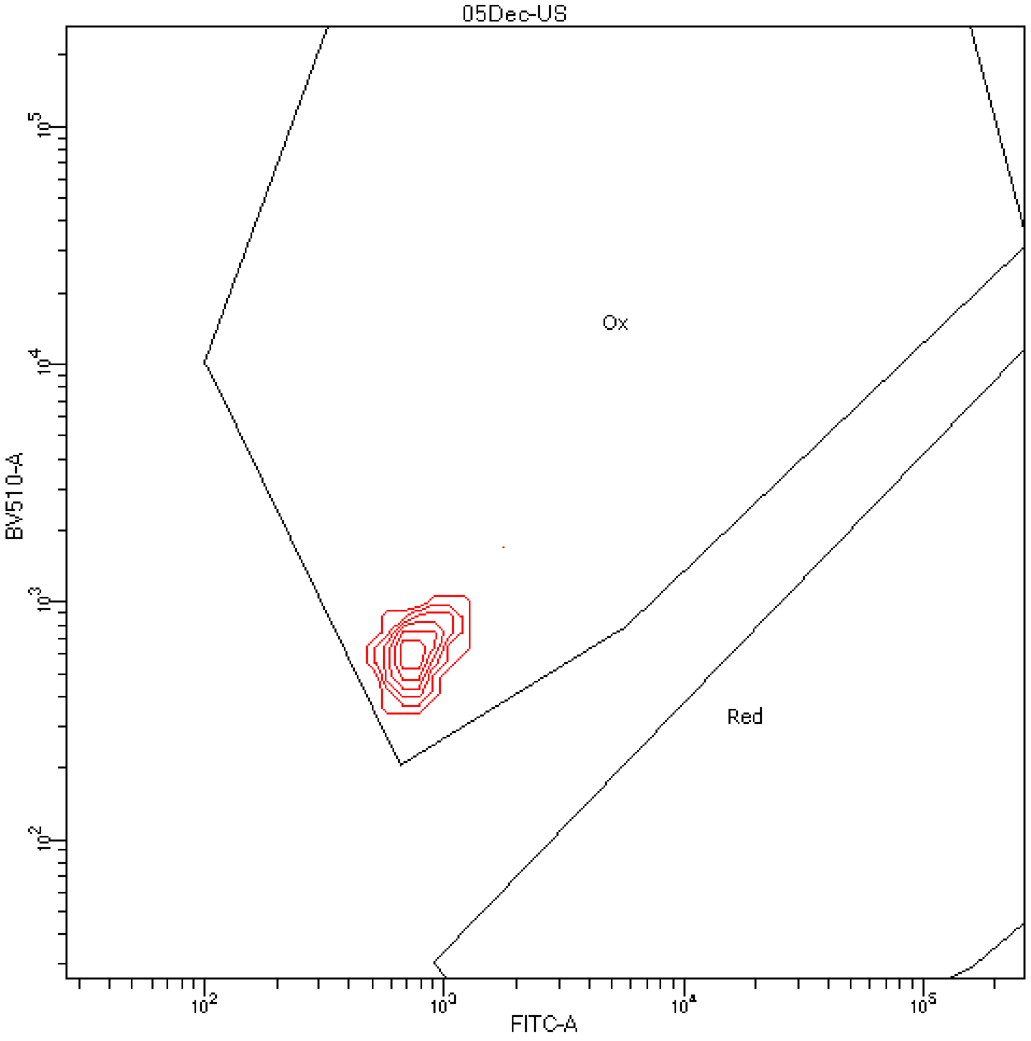

BD FACSDiva 8.0.1

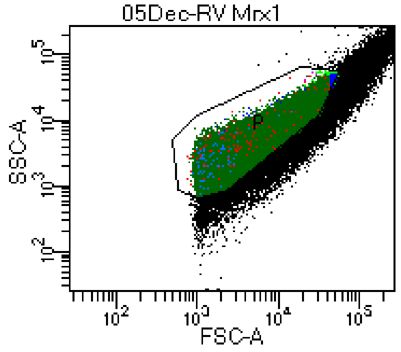

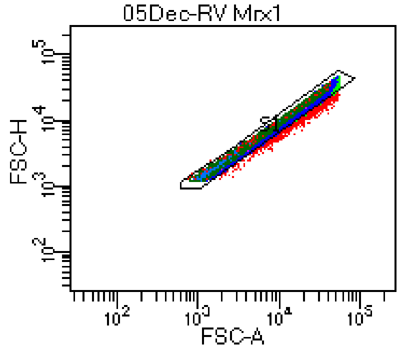

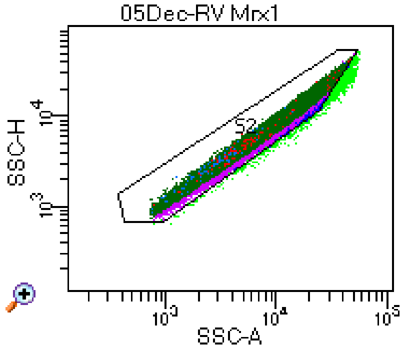

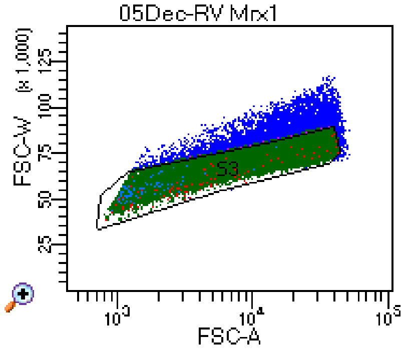

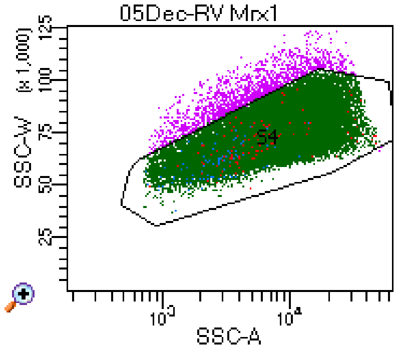

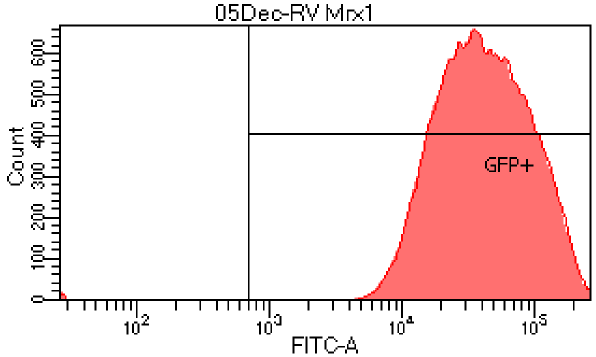

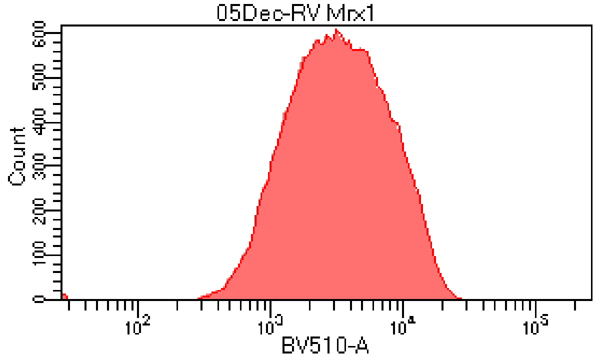

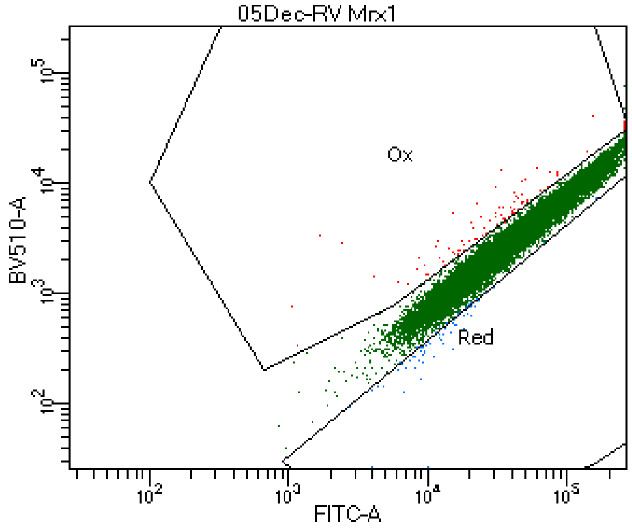

Tube: RV Mrx1

Population	#Events	%Parent	%Total
All Events	73,400	####	100.0
P	50,000	68.1	68.1
S1	48,999	98.0	66.8
S2	47,520	97.0	64.7
S3	41,892	88.2	57.1
S4	40,244	96.1	54.8
GFP+	40,169	99.8	54.7
Ox	99	0.2	0.1
Red	80	0.2	0.1

Experiment Name: 05EDec2016 Bac sorting
Specimen Name: 05Dec
Tube Name: RV Mrx1
Record Date: Dec 5, 2016 4:27:38 PM
$OP: Administrator
GUID: 813183c7-53b3-4ab5-a621-30b...

Population	#Events	%Parent	FITC-A Median	BV510-A Median
S4	40,244	96.1	38,973	3,103
GFP+	40,169	99.8	39,042	3,109
Ox	99	0.2	34,773	5,305
Red	80	0.2	11,255	351

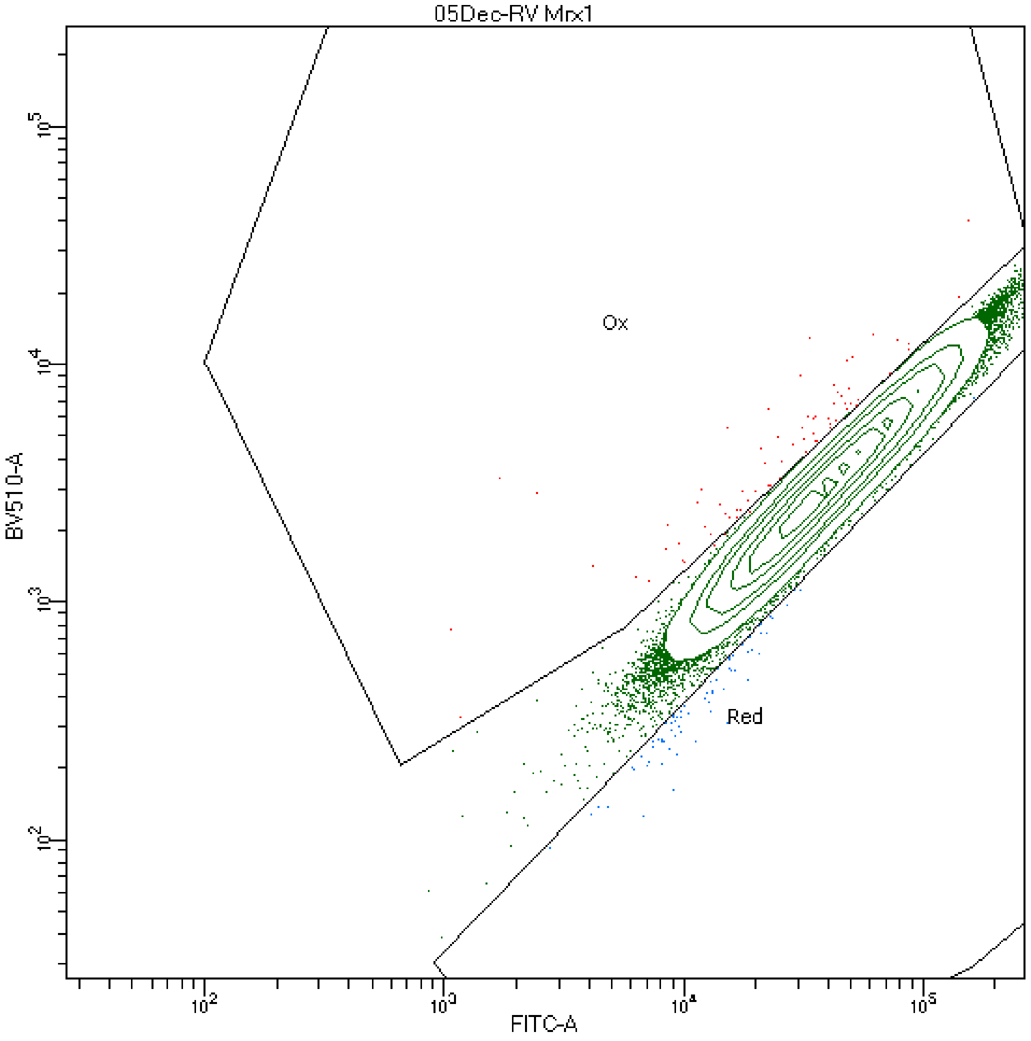

05Dec-RV Mrx1
Ox
Red
BV510-A
FITC-A
10^2
10^3
10^4
10^5

BD FACSDiva 8.0.1

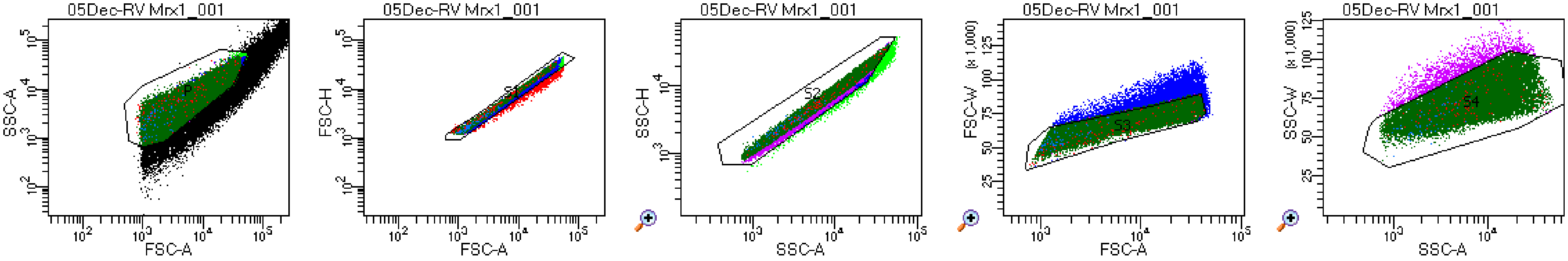

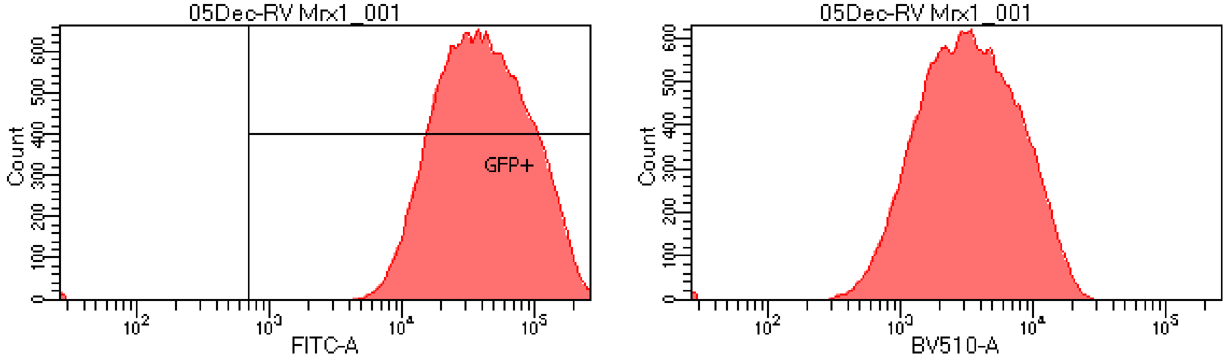

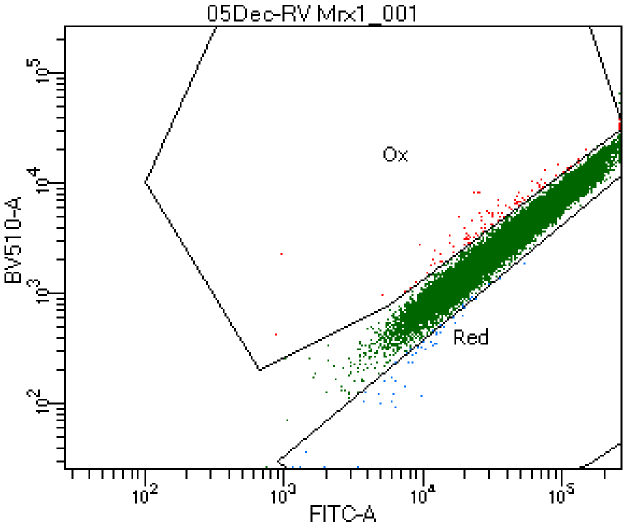

Tube: RV Mrx1_001

Population	#Events	%Parent	%Total
All Events	72,527	####	100.0
P	50,000	68.9	68.9
S1	48,928	97.9	67.5
S2	47,472	97.0	65.5
S3	41,835	88.1	57.7
S4	40,159	96.0	55.4
GFP+	40,090	99.8	55.3
Ox	111	0.3	0.2
Red	65	0.2	0.1

Experiment Name:	05EDec2016 Bac sorting
Specimen Name:	05Dec
Tube Name:	RV Mrx1_001
Record Date:	Dec 5, 2016 4:28:13 PM
$OP:	Administrator
GUID:	48e3b8f3-3235-4b3c-a476-55fc...

Population	#Events	%Parent	FITC-A Median	BV510-A Median
S4	40,159	96.0	38,482	3,119
GFP+	40,090	99.8	38,575	3,126
Ox	111	0.3	34,512	5,441
Red	65	0.2	11,200	369

BD FACSDiva 8.0.1

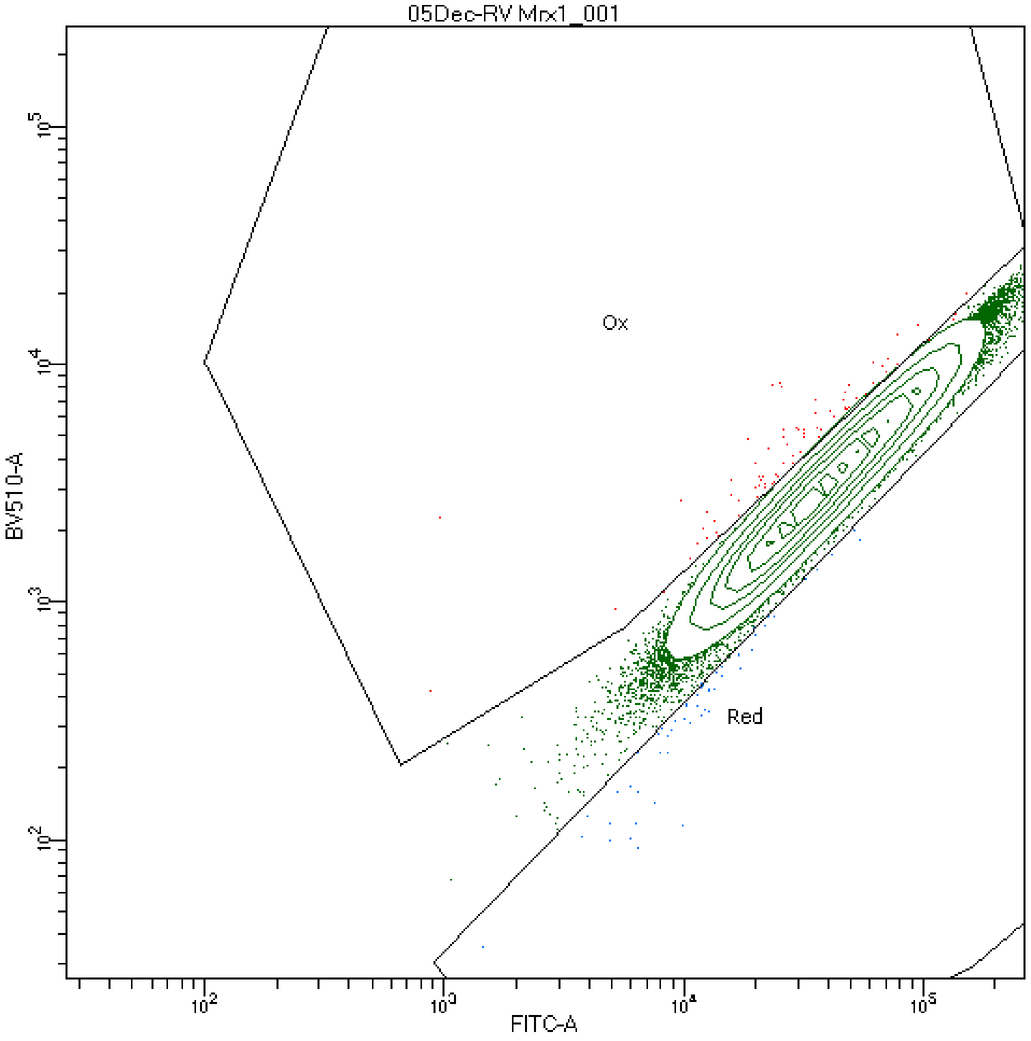

BD FACSDiva 8.0.1

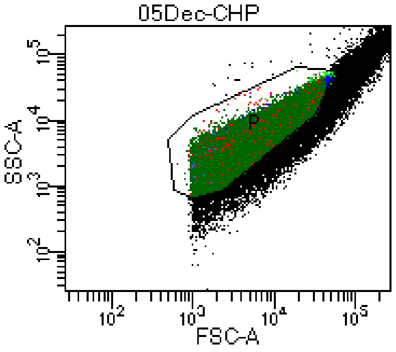

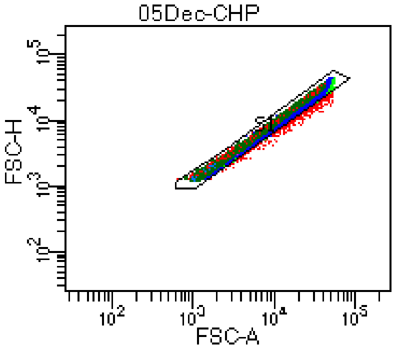

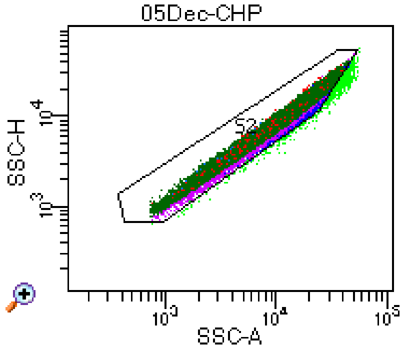

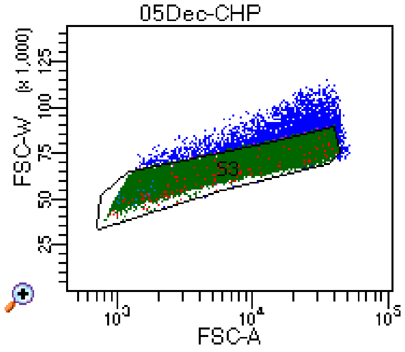

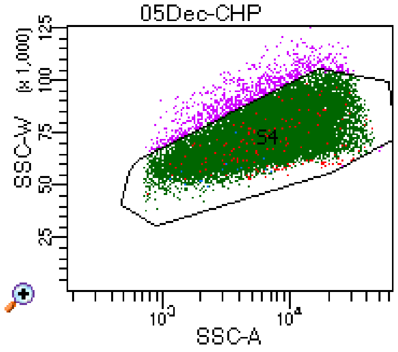

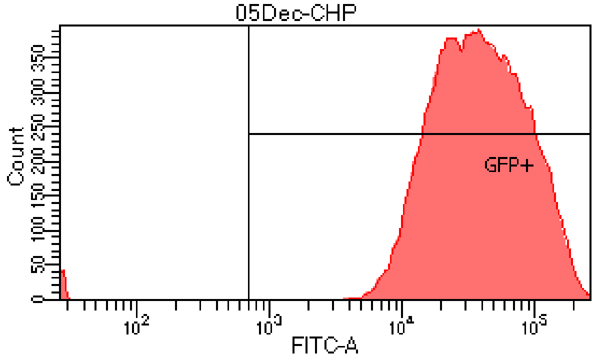

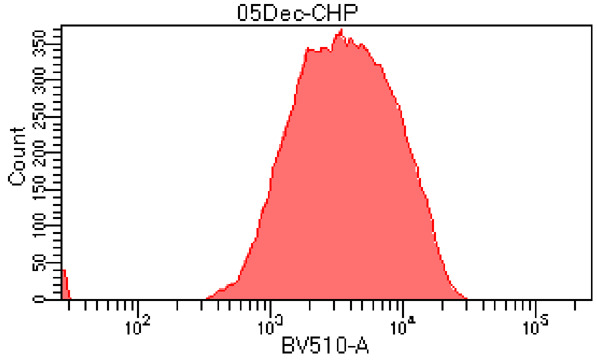

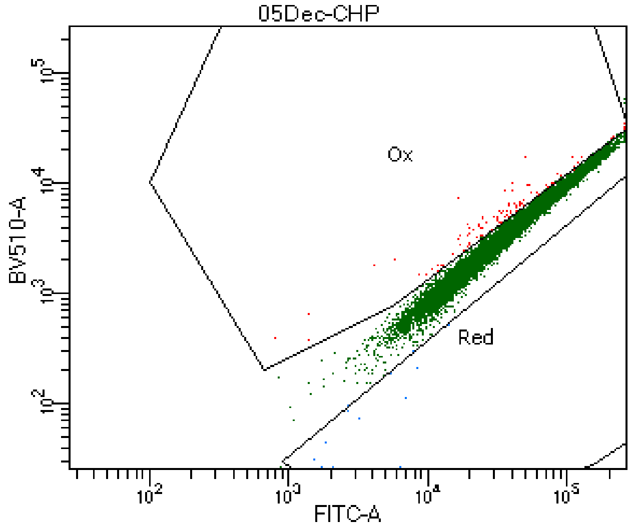

Tube: CHP

Population	#Events	%Parent	%Total
All Events	44,664	####	100.0
P	30,000	67.2	67.2
S1	29,535	98.4	66.1
S2	28,675	97.1	64.2
S3	25,809	90.0	57.8
S4	24,892	96.4	55.7
GFP+	24,758	99.5	55.4
Ox	114	0.5	0.3
Red	14	0.1	0.0

Experiment Name:	05EDec2016 Bac sorting
Specimen Name:	05Dec
Tube Name:	CHP
Record Date:	Dec 5, 2016 2:16:12 PM
$OP:	Administrator
GUID:	03ce0e3c-2747-4f85-9e42-729...

Population	#Events	%Parent	FITC-A Median	BV510-A Median
S4	24,892	96.4	36,316	3,412
GFP+	24,758	99.5	36,574	3,434
Ox	114	0.5	37,371	5,578
Red	14	0.1	2,906	81

BD FACSDiva 8.0.1

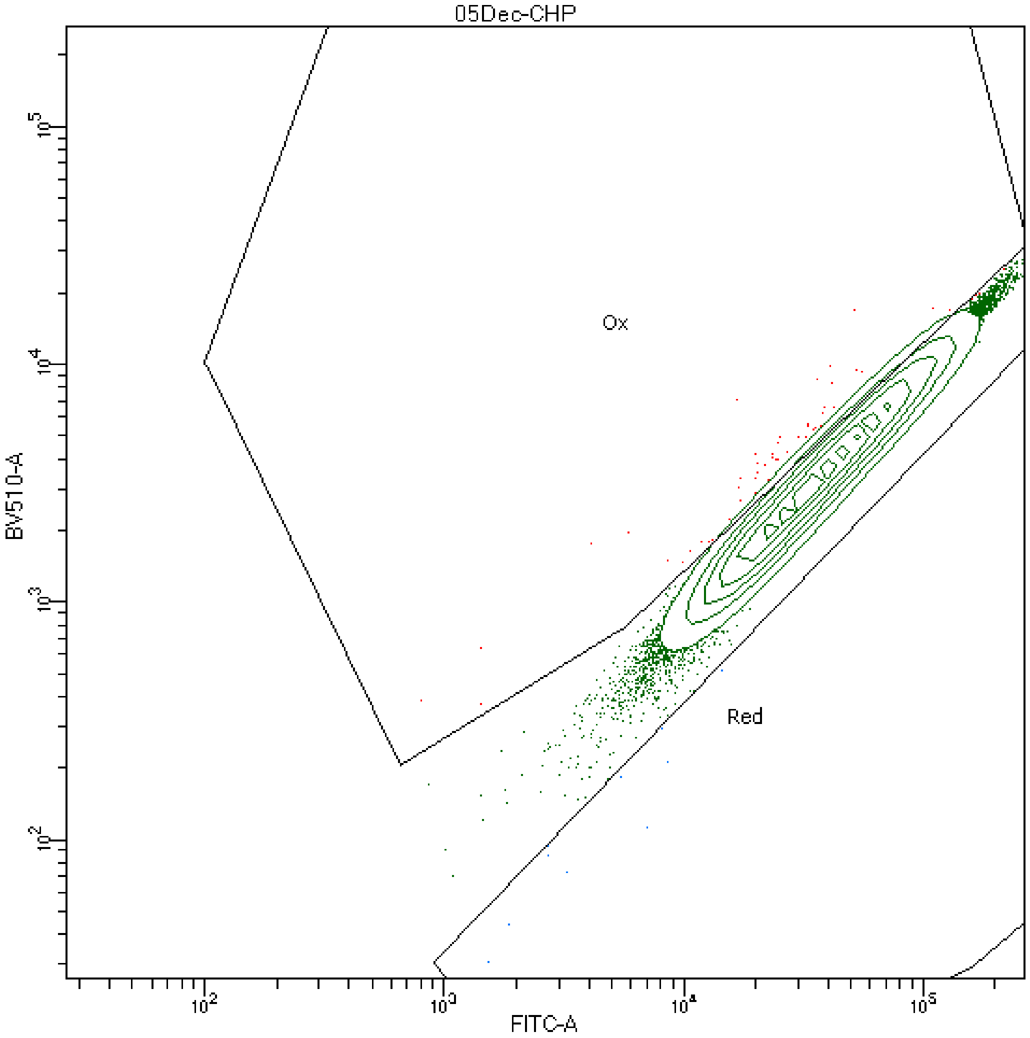

BD FACSDiva 8.0.1

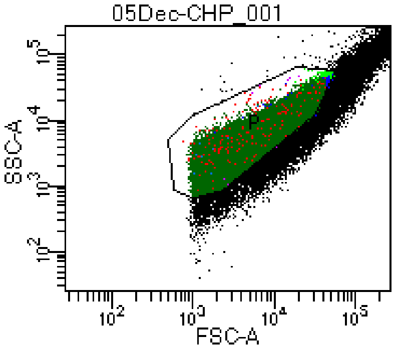

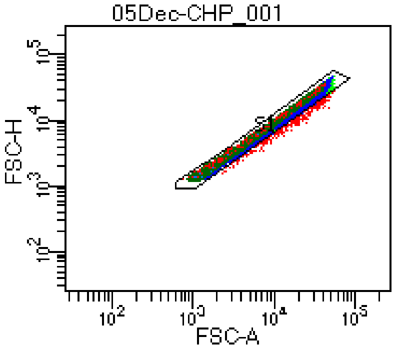

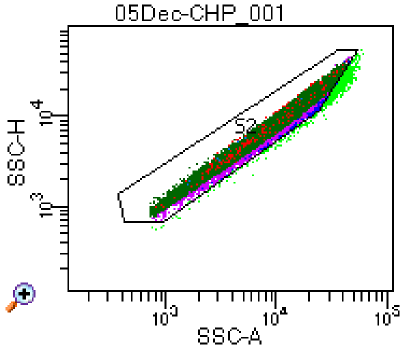

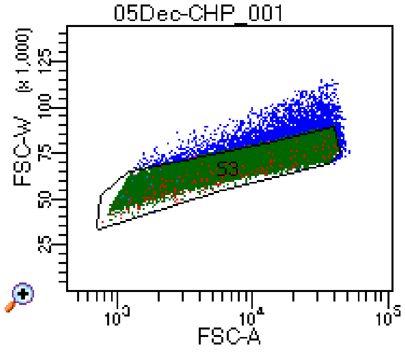

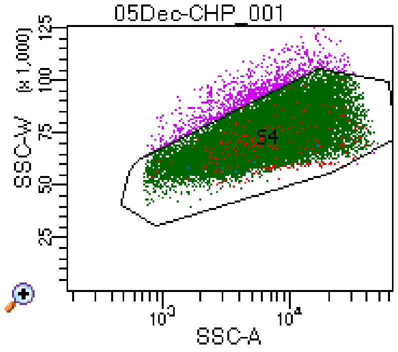

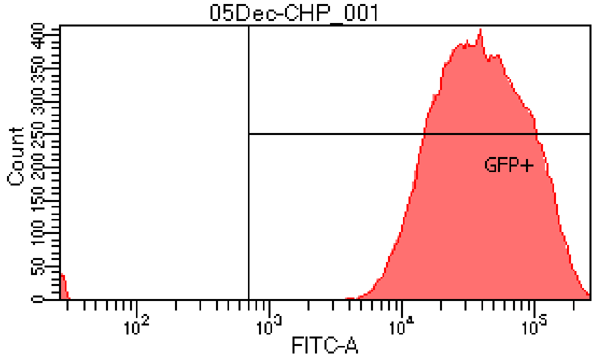

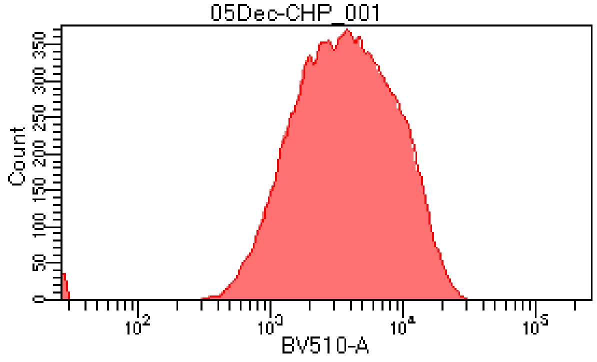

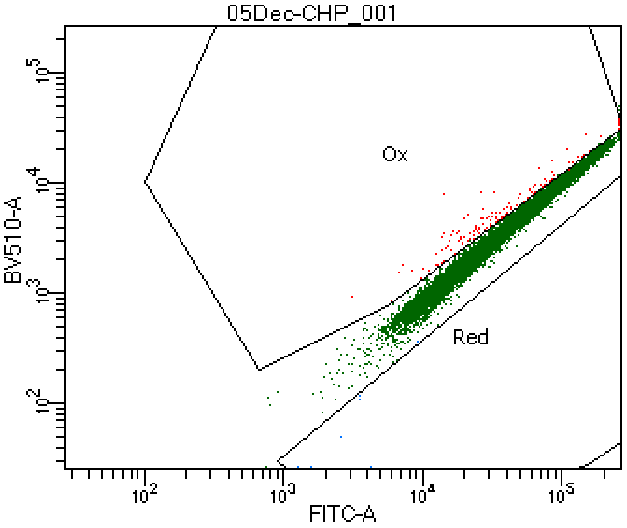

Tube: CHP_001

Population	#Events	%Parent	%Total
All Events	44,871	####	100.0
P	30,000	66.9	66.9
S1	29,488	98.3	65.7
S2	28,652	97.2	63.9
S3	25,772	89.9	57.4
S4	24,840	96.4	55.4
GFP+	24,720	99.5	55.1
Ox	153	0.6	0.3
Red	8	0.0	0.0

Experiment Name: 05EDec2016 Bac sorting
Specimen Name: 05Dec
Tube Name: CHP_001
Record Date: Dec 5, 2016 2:16:48 PM
$OP: Administrator
GUID: 3f155b14-e8e4-4475-b6e8-e65...

Population	#Events	%Parent	FITC-A Median	BV510-A Median
S4	24,840	96.4	36,959	3,501
GFP+	24,720	99.5	37,131	3,521
Ox	153	0.6	33,921	5,178
Red	8	0.0	3,440	80

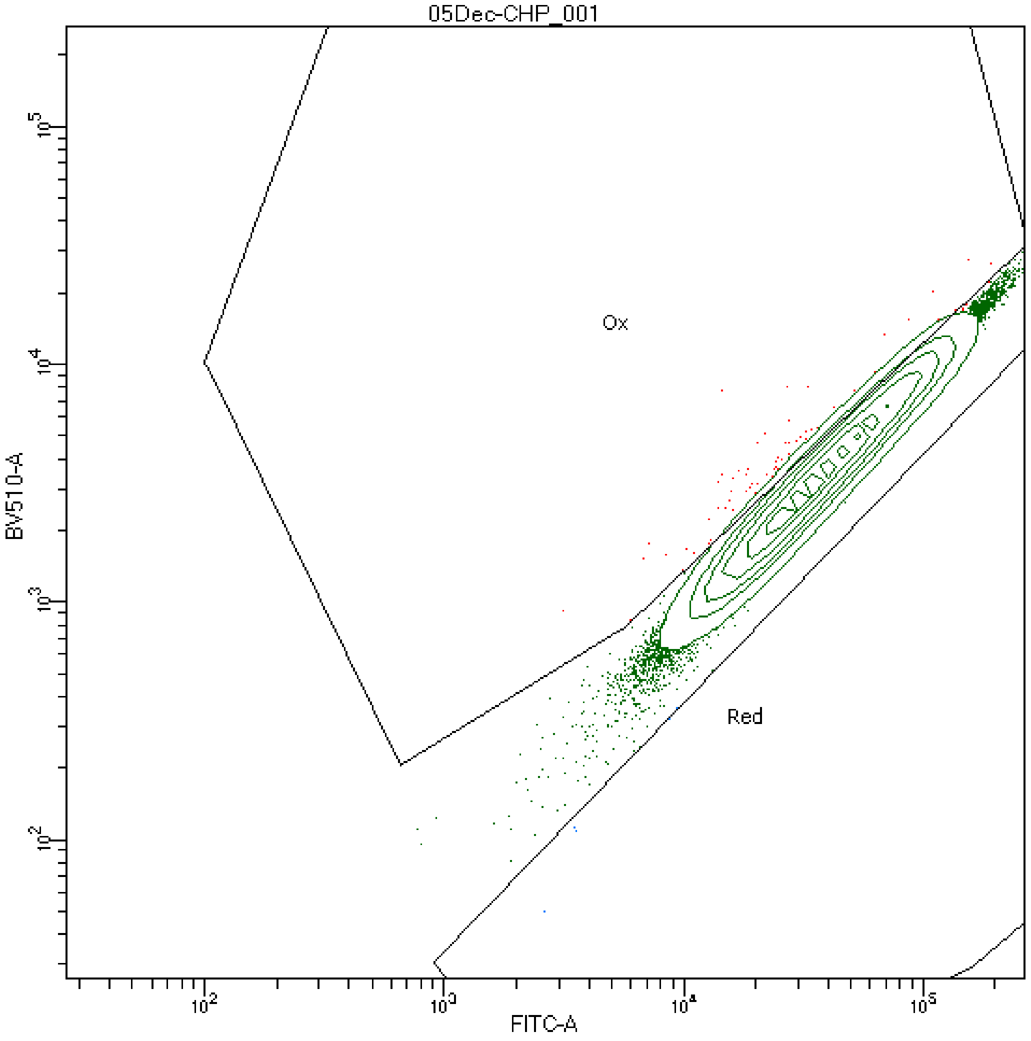
05Dec-CHP_001
Ox
Red
BV510-A
FITC-A
10^2
10^3
10^4
10^5

BD FACSDiva 8.0.1

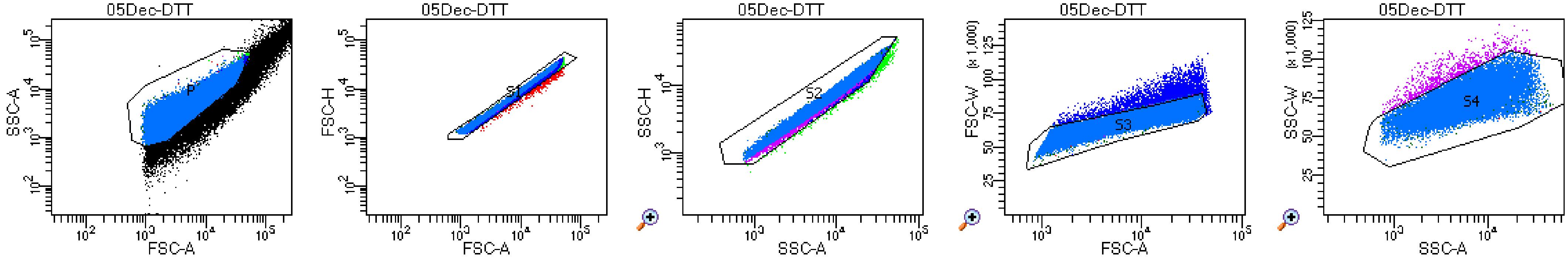

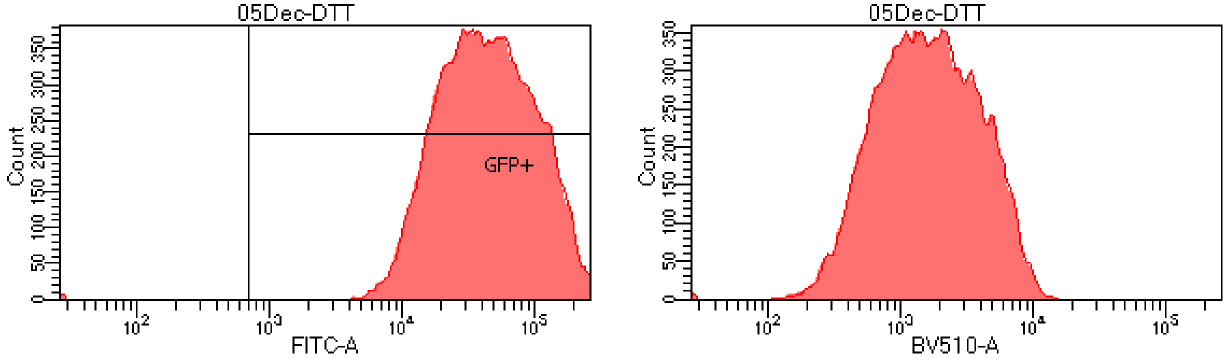

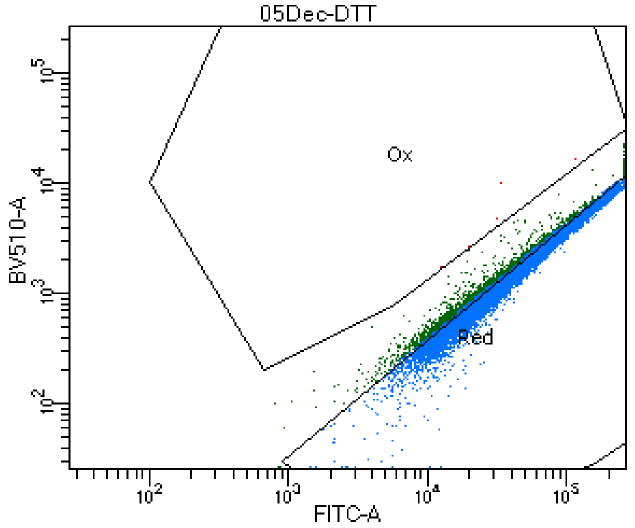

Tube: DTT

Population	#Events	%Parent	%Total
All Events	46,622	####	100.0
P	30,000	64.3	64.3
S1	29,589	98.6	63.5
S2	29,061	98.2	62.3
S3	25,739	88.6	55.2
S4	24,886	96.7	53.4
GFP+	24,840	99.8	53.3
Ox	5	0.0	0.0
Red	22,531	90.7	48.3

Experiment Name: 05EDec2016 Bac sorting
Specimen Name: 05Dec
Tube Name: DTT
Record Date: Dec 5, 2016 2:17:32 PM
$OP: Administrator
GUID: 12713f7e-9ec3-4721-b70f-8612...

Population	#Events	%Parent	FITC-A Median	BV510-A Median
S4	24,886	96.7	41,675	1,517
GFP+	24,840	99.8	41,751	1,522
Ox	5	0.0	31,330	4,762
Red	22,531	90.7	44,170	1,584

BD FACSDiva 8.0.1

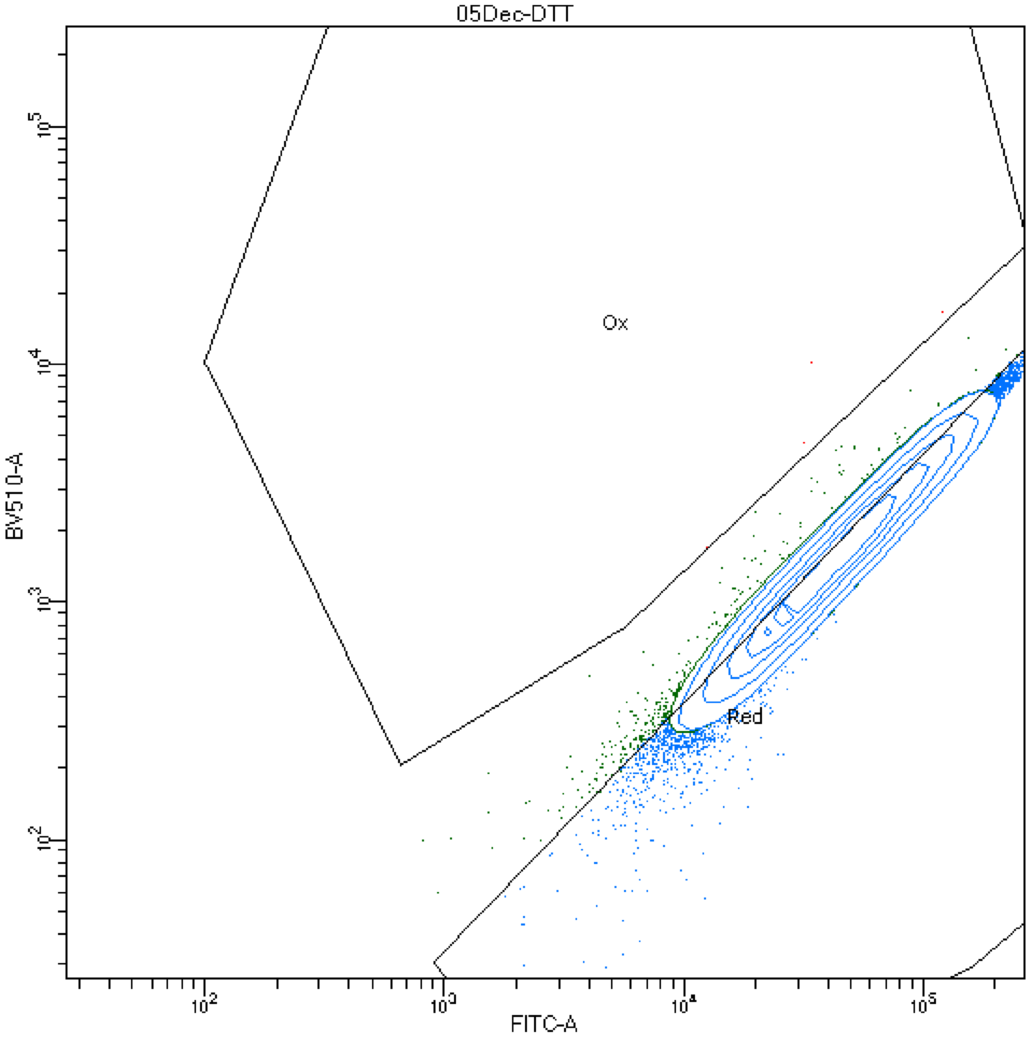

BD FACSDiva 8.0.1

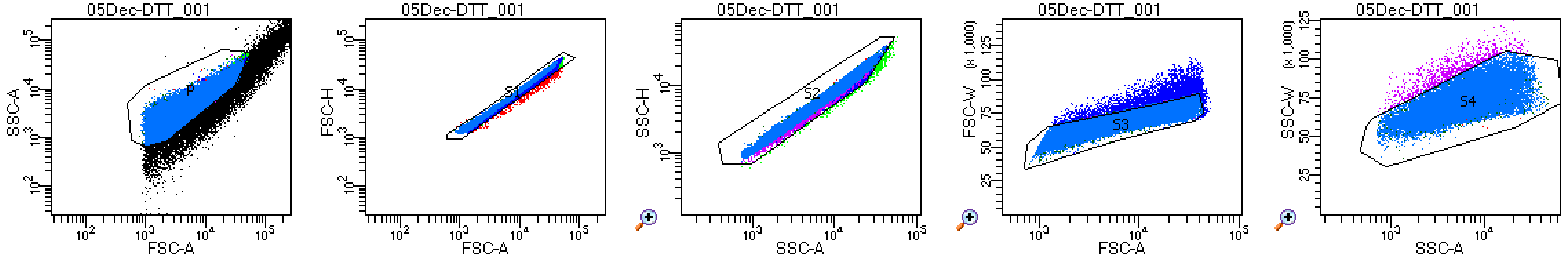

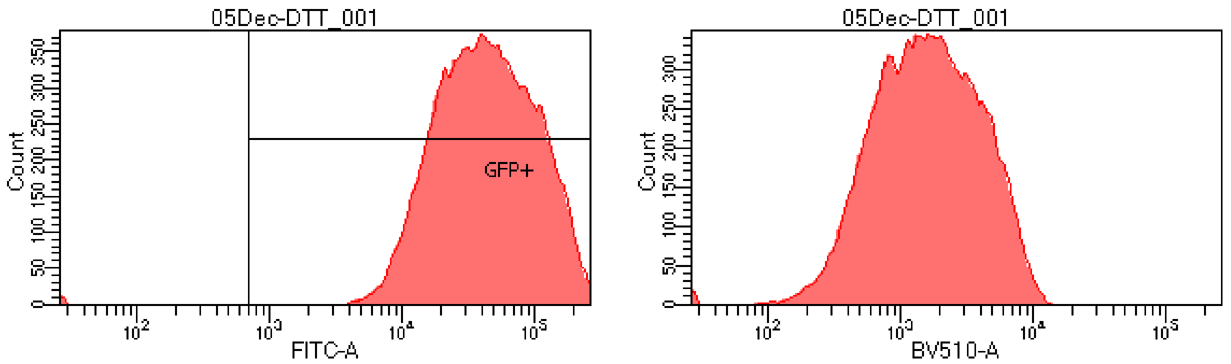

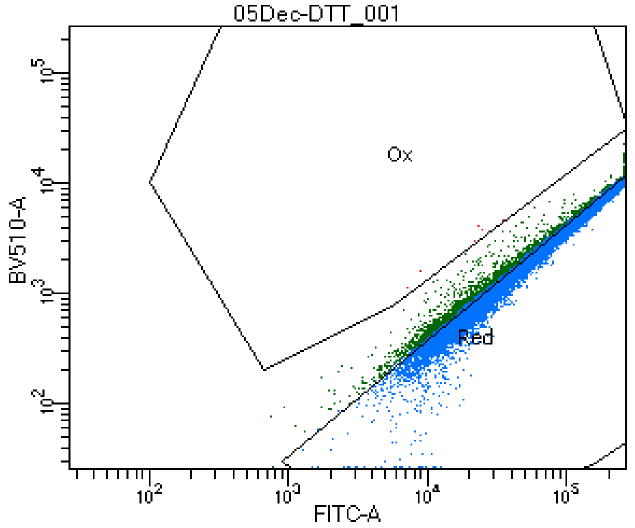

Tube: DTT_001

Population	#Events	%Parent	%Total
All Events	45,911	####	100.0
P	30,086	65.5	65.5
S1	29,598	98.4	64.5
S2	29,085	98.3	63.4
S3	25,567	87.9	55.7
S4	24,674	96.5	53.7
GFP+	24,623	99.8	53.6
Ox	6	0.0	0.0
Red	21,701	88.1	47.3

Experiment Name:	05EDec2016 Bac sorting
Specimen Name:	05Dec
Tube Name:	DTT_001
Record Date:	Dec 5, 2016 2:18:07 PM
$OP:	Administrator
GUID:	c147e877-cb3d-44e6-bc19-948...

Population	#Events	%Parent	FITC-A Median	BV510-A Median
S4	24,674	96.5	41,314	1,521
GFP+	24,623	99.8	41,416	1,524
Ox	6	0.0	22,280	3,358
Red	21,701	88.1	43,937	1,572

BD FACSDiva 8.0.1

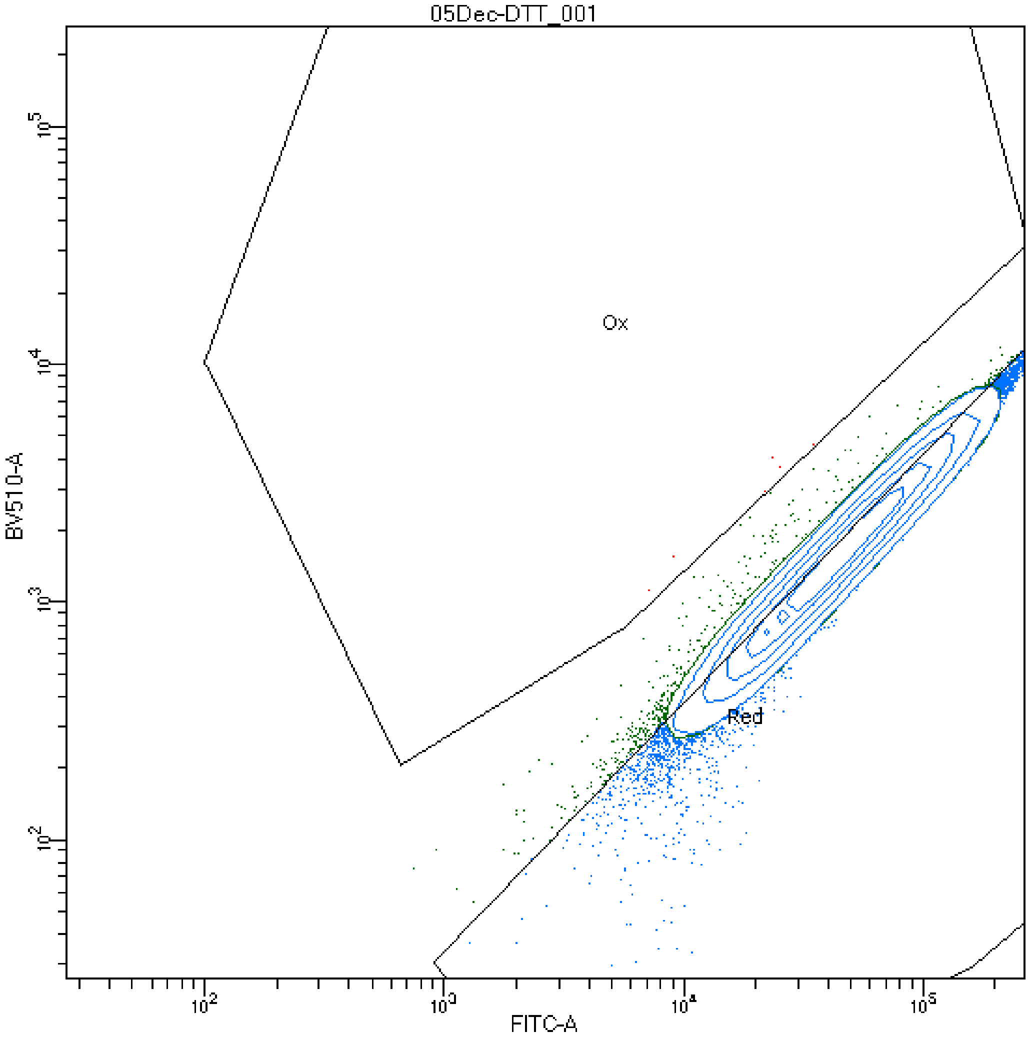

BD FACSDiva 8.0.1

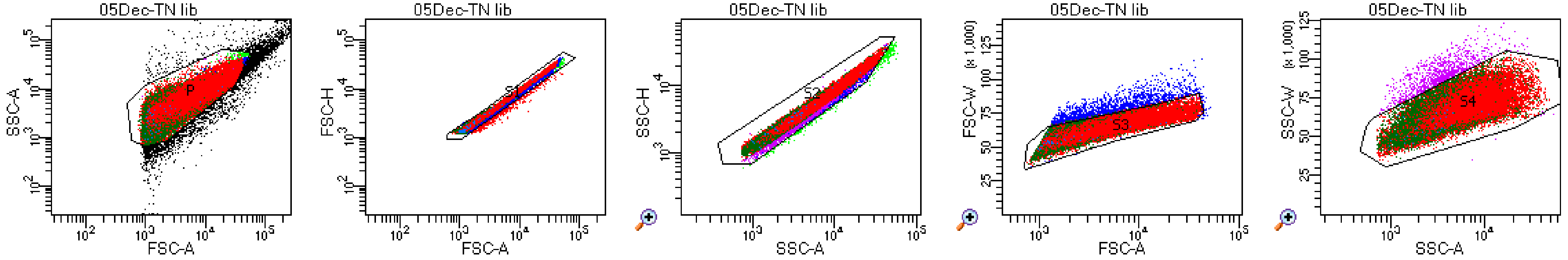

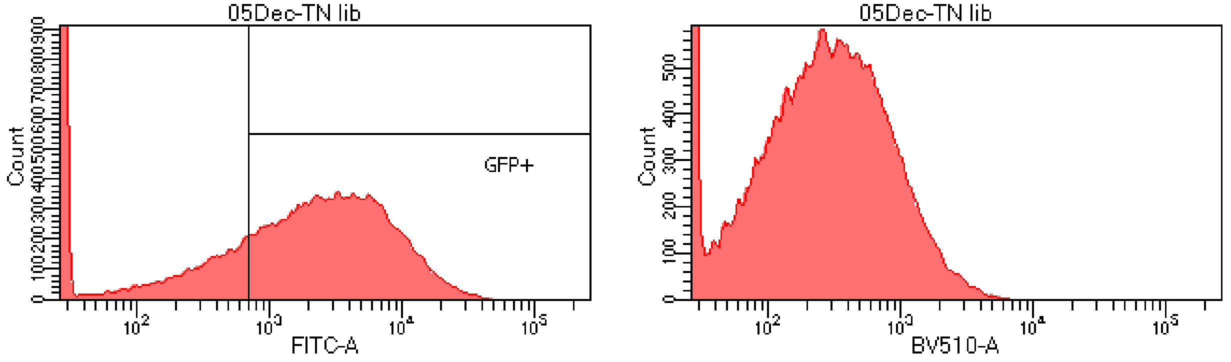

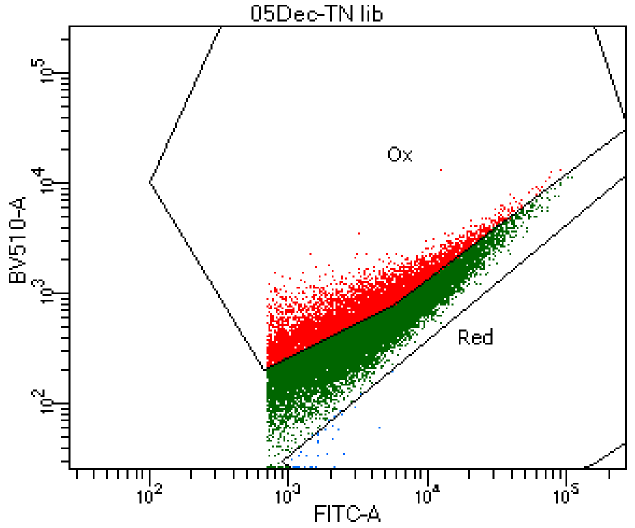

Tube: TN lib

Population	#Events	%Parent	%Total
All Events	52,894	####	100.0
P	50,000	94.5	94.5
S1	49,580	99.2	93.7
S2	49,214	99.3	93.0
S3	47,615	96.8	90.0
S4	46,683	98.0	88.3
GFP+	26,297	56.3	49.7
Ox	6,602	25.1	12.5
Red	38	0.1	0.1

Experiment Name:	05EDec2016 Bac sorting
Specimen Name:	05Dec
Tube Name:	TN lib
Record Date:	Dec 5, 2016 2:18:55 PM
$OP:	Administrator
GUID:	456fb14e-329b-4373-9f97-dfd4...

Population	#Events	%Parent	FITC-A Median	BV510-A Median
S4	46,683	98.0	1,070	250
GFP+	26,297	56.3	3,347	471
Ox	6,602	25.1	2,934	713
Red	38	0.1	1,605	45

BD FACSDiva 8.0.1

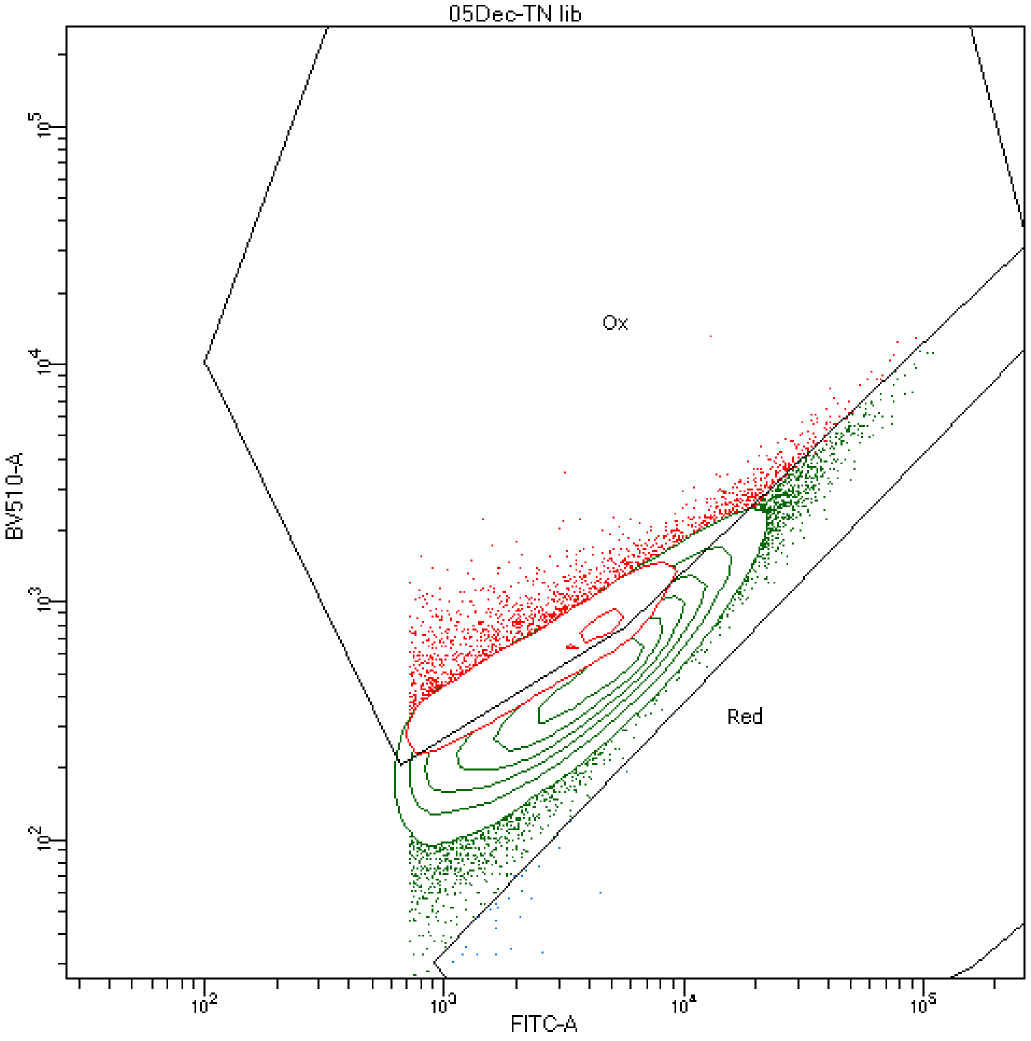

BD FACSDiva 8.0.1

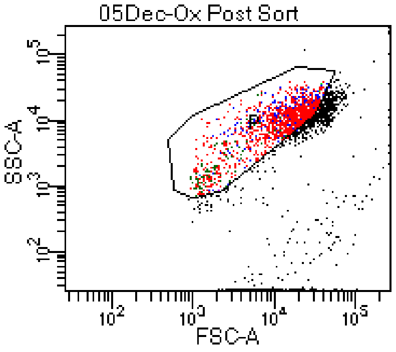

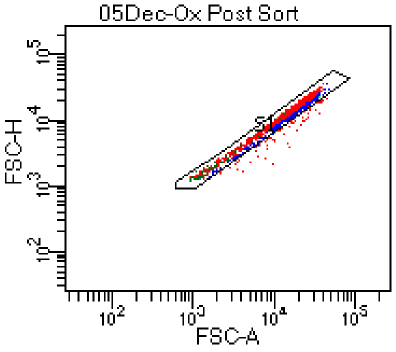

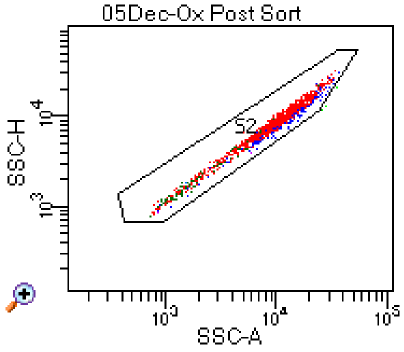

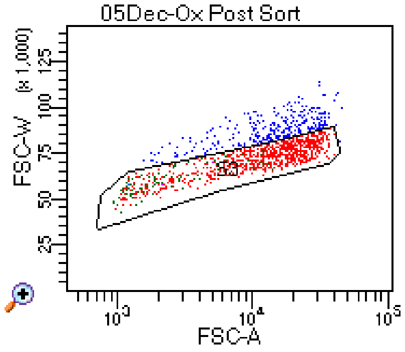

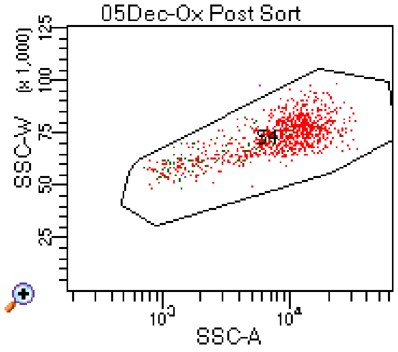

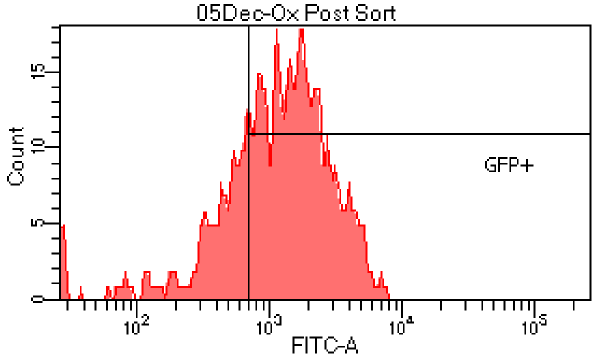

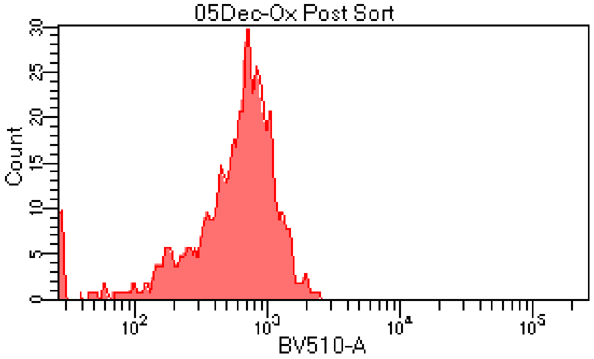

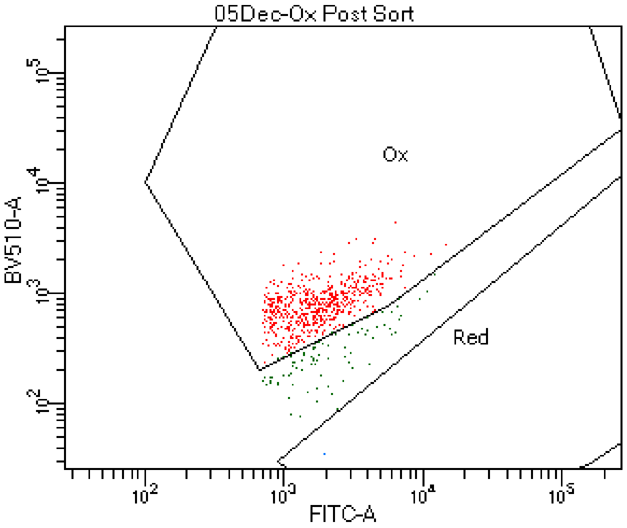

Tube: Ox Post Sort

Population	#Events	%Parent	%Total
All Events	2,228	####	100.0
P	1,358	61.0	61.0
S1	1,276	94.0	57.3
S2	1,273	99.8	57.1
S3	978	76.8	43.9
S4	976	99.8	43.8
GFP+	684	70.1	30.7
Ox	593	86.7	26.6
Red	1	0.1	0.0

Experiment Name: 05EDec2016 Bac sorting
Specimen Name: 05Dec
Tube Name: Ox Post Sort
Record Date: Dec 5, 2016 4:23:30 PM
$OP: Administrator
GUID: 501515bc-0809-4310-97f5-109...

Population	#Events	%Parent	FITC-A Median	BV510-A Median
S4	976	99.8	1,159	623
GFP+	684	70.1	1,663	741
Ox	593	86.7	1,636	794
Red	1	0.1	1,935	36

BD FACSDiva 8.0.1

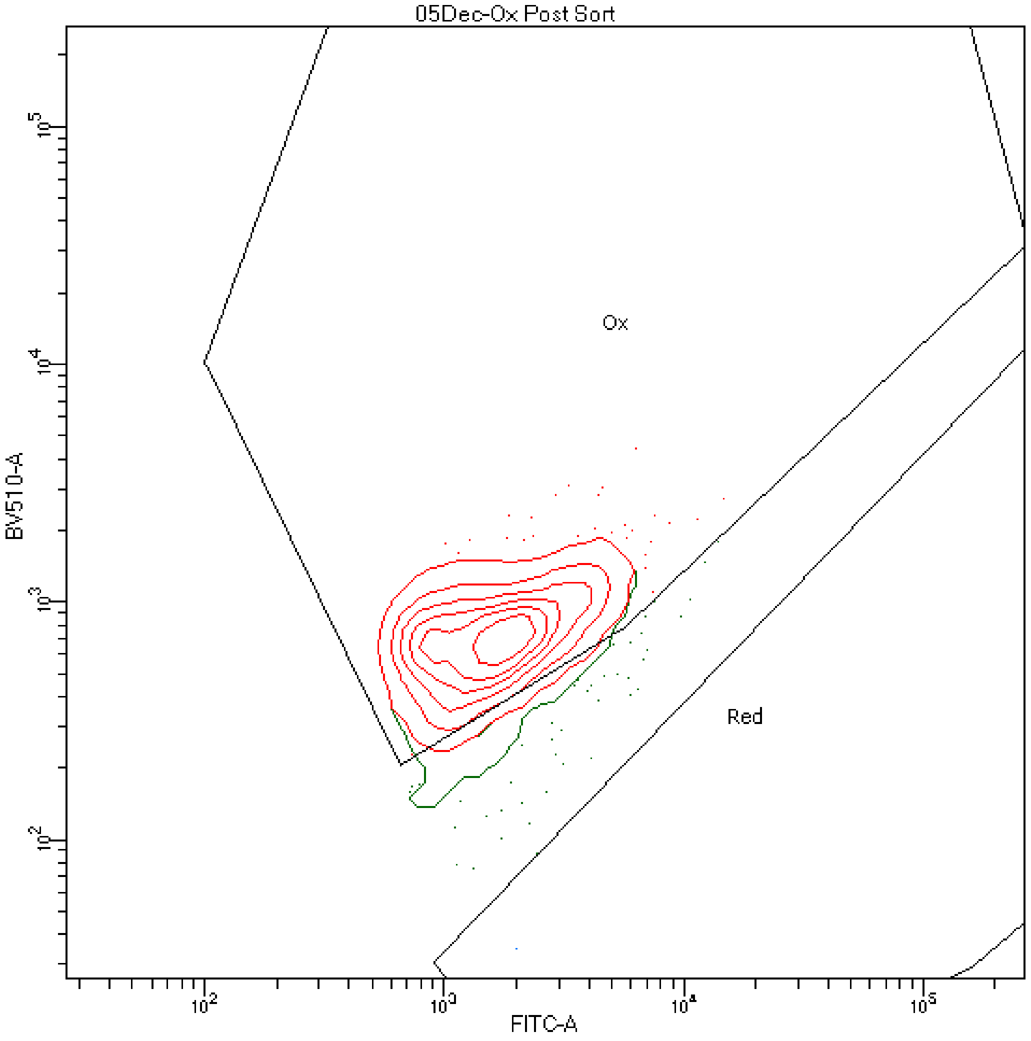

BD FACSDiva 8.0.1

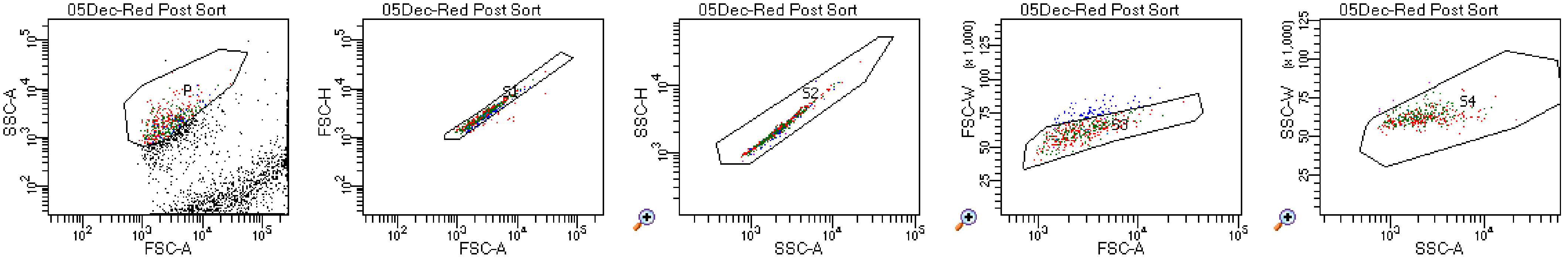

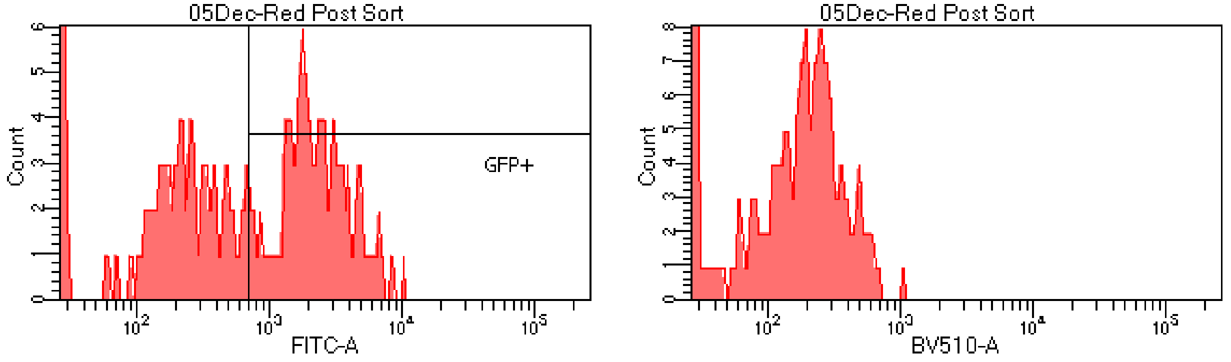

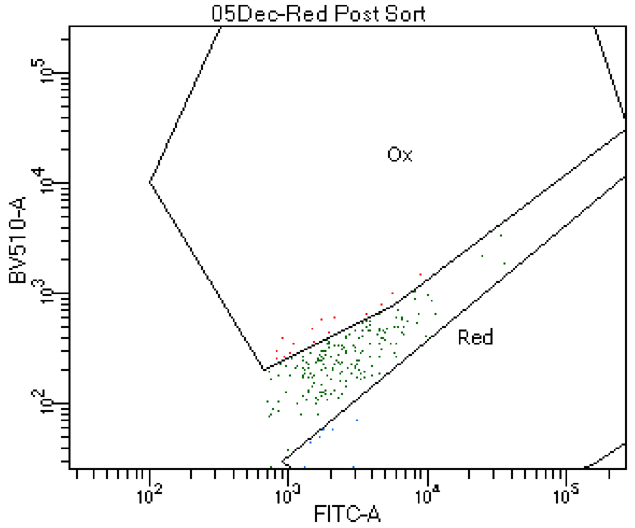

Tube: Red Post Sort

Population	#Events	%Parent	%Total
All Events	4,648	####	100.0
P	513	11.0	11.0
S1	496	96.7	10.7
S2	496	100.0	10.7
S3	421	84.9	9.1
S4	417	99.0	9.0
GFP+	206	49.4	4.4
Ox	15	7.3	0.3
Red	7	3.4	0.2

Experiment Name:	05EDec2016 Bac sorting
Specimen Name:	05Dec
Tube Name:	Red Post Sort
Record Date:	Dec 5, 2016 4:24:26 PM
$OP:	Administrator
GUID:	2cd99c73-31ca-4a5d-a6d5-619...

Population	#Events	%Parent	FITC-A Median	BV510-A Median
S4	417	99.0	662	161
GFP+	206	49.4	2,254	250
Ox	15	7.3	1,494	436
Red	7	3.4	1,756	50

BD FACSDiva 8.0.1

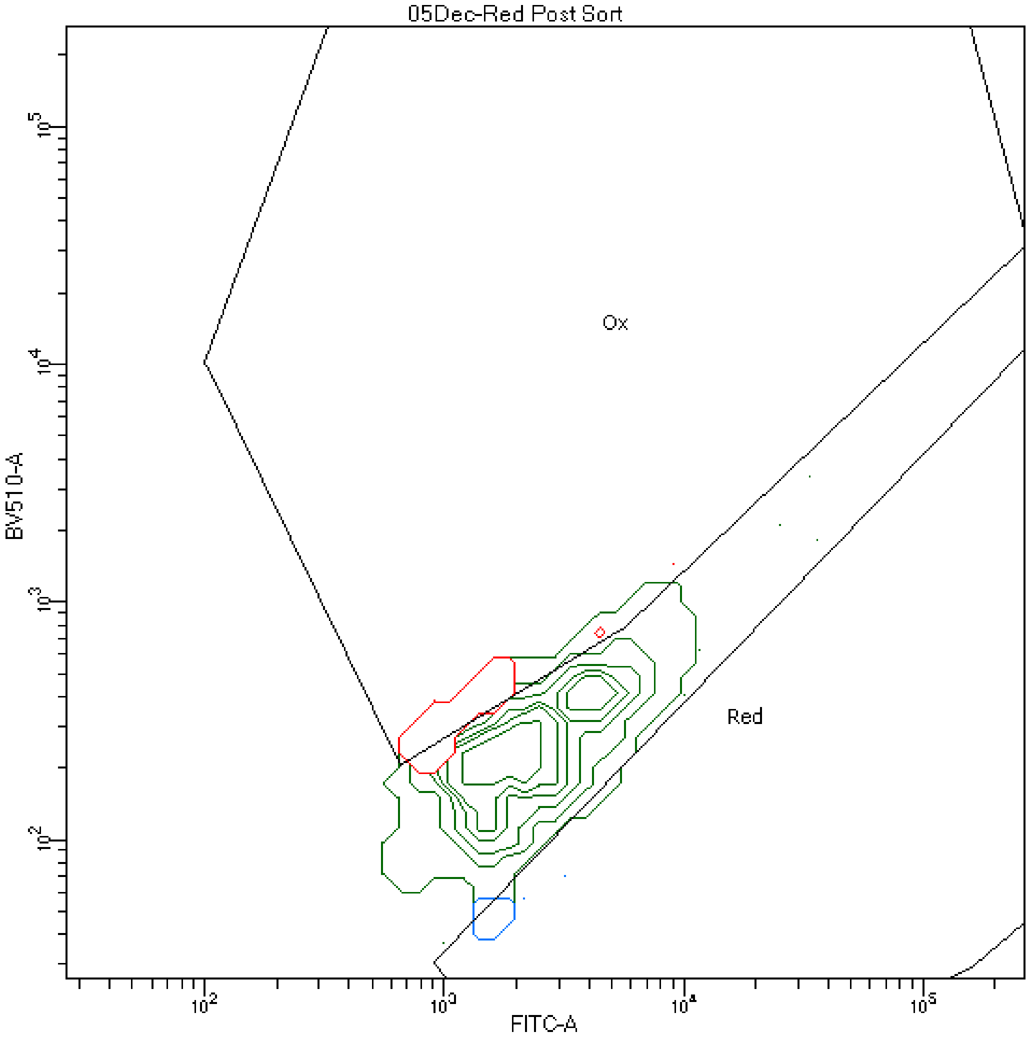

BD FACSDiva 8.0.1

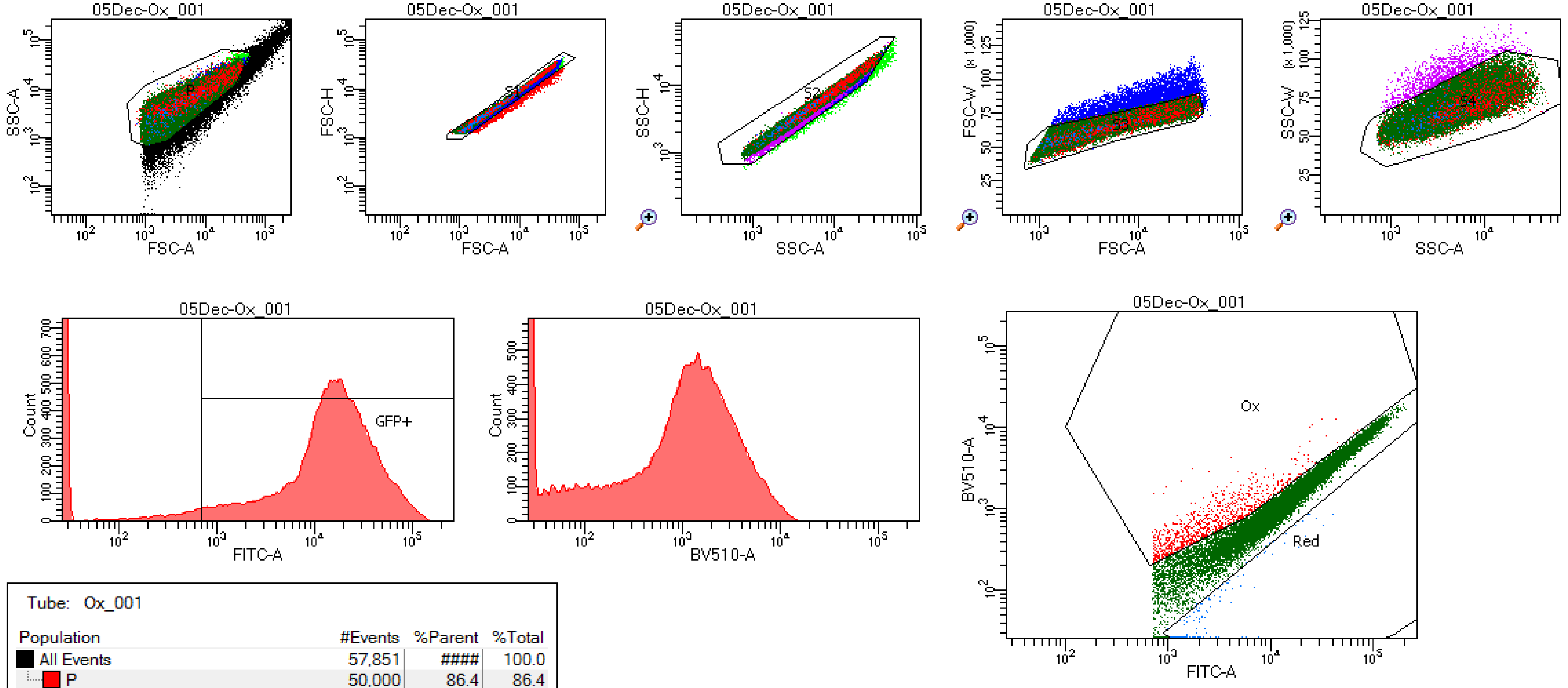

Tube: Ox_001

Population	#Events	%Parent	%Total
All Events	57,851	####	100.0
P	50,000	86.4	86.4
S1	48,685	97.4	84.2
S2	47,755	98.1	82.5
S3	43,037	90.1	74.4
S4	41,709	96.9	72.1
GFP+	29,482	70.7	51.0
Ox	902	3.1	1.6
Red	136	0.5	0.2

Experiment Name: 05EDec2016 Bac sorting
Specimen Name: 05Dec
Tube Name: Ox_001
Record Date: Dec 5, 2016 4:25:28 PM
$OP: Administrator
GUID: d4b9efa1-0167-47af-b938-078f...

Population	#Events	%Parent	FITC-A Median	BV510-A Median
S4	41,709	96.9	9,983	837
GFP+	29,482	70.7	15,811	1,360
Ox	902	3.1	2,257	647
Red	136	0.5	1,999	42

BD FACSDiva 8.0.1

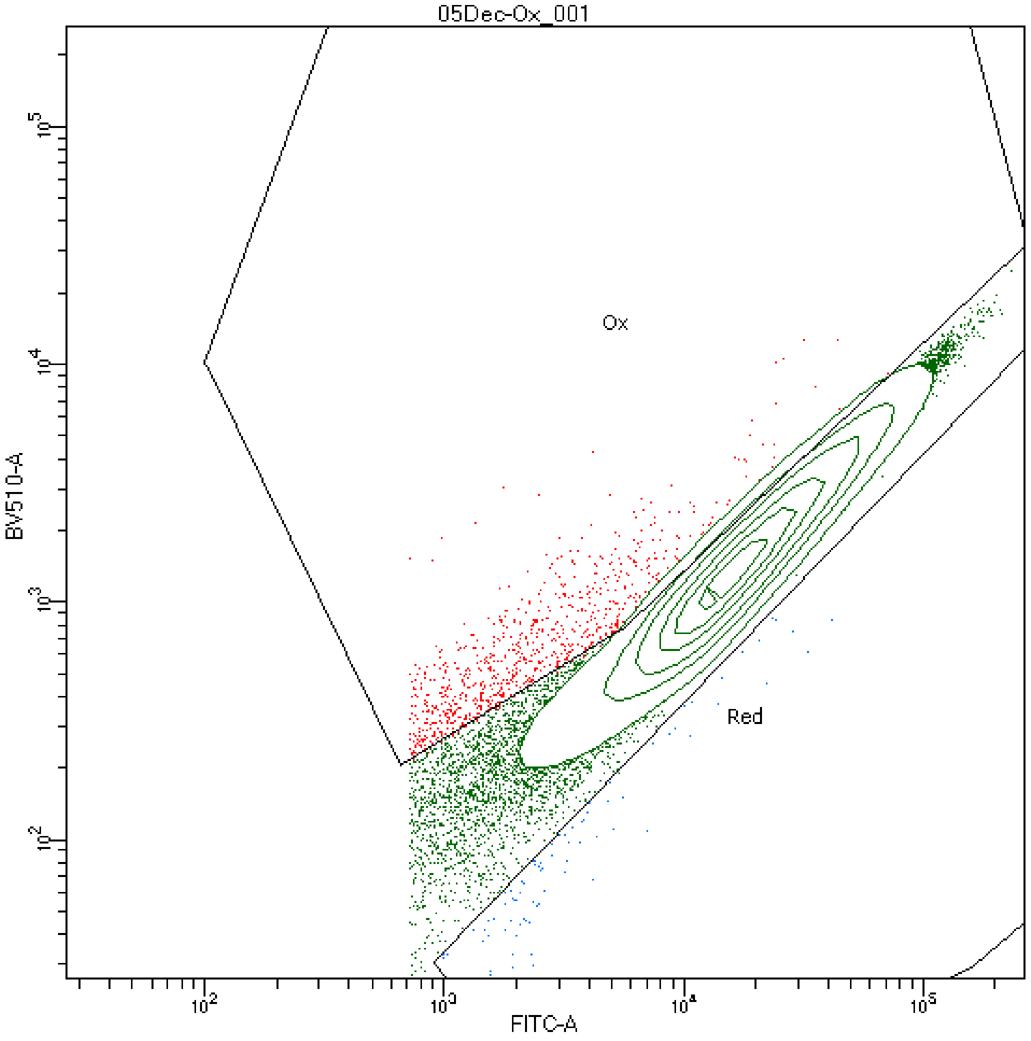

BD FACSDiva 8.0.1

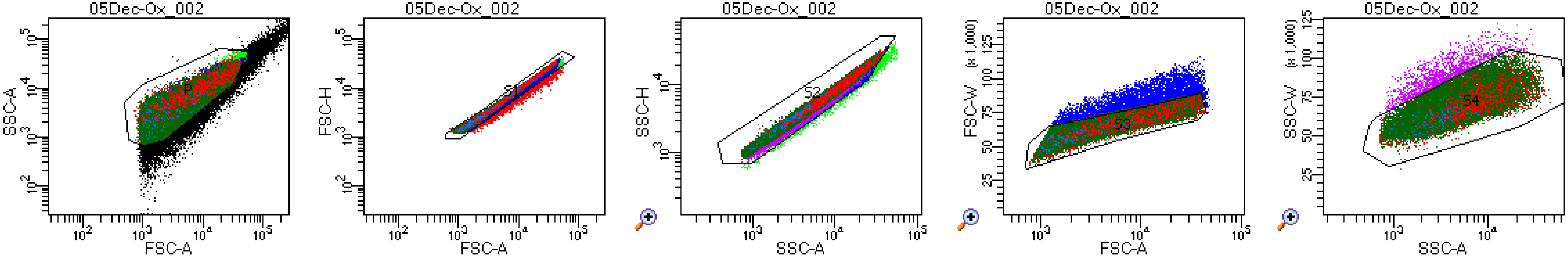

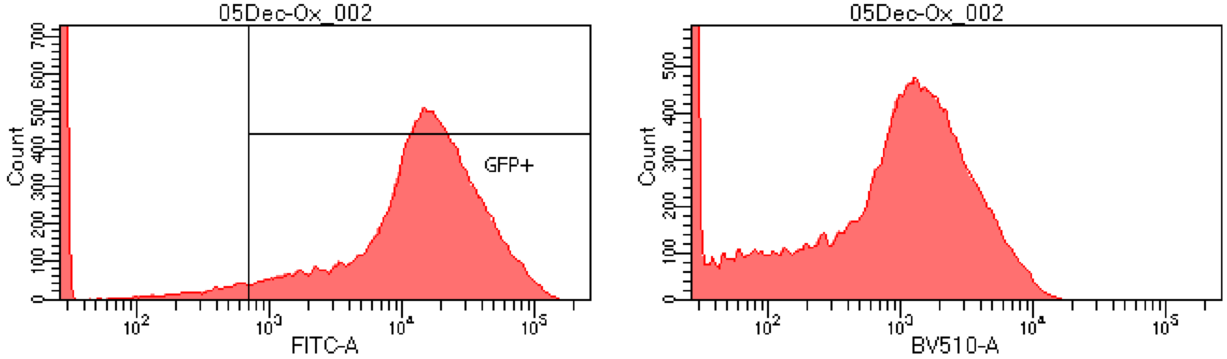

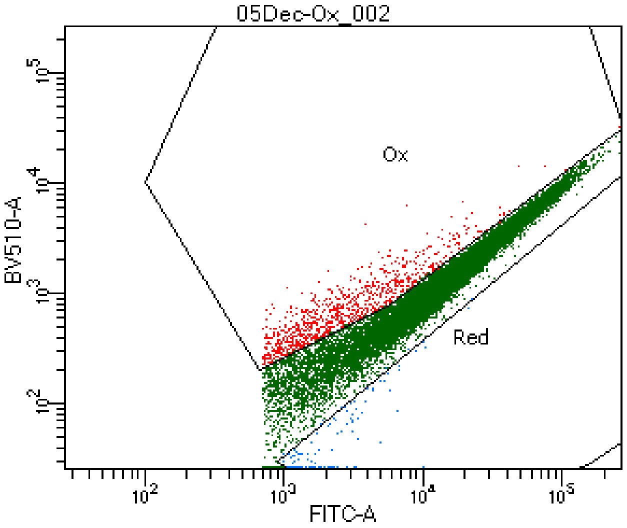

Tube: Ox_002

Population	#Events	%Parent	%Total
All Events	58,137	####	100.0
P	50,000	86.0	86.0
S1	48,577	97.2	83.6
S2	47,655	98.1	82.0
S3	42,806	89.8	73.6
S4	41,437	96.8	71.3
GFP+	29,233	70.5	50.3
Ox	888	3.0	1.5
Red	124	0.4	0.2

Experiment Name: 05EDec2016 Bac sorting
Specimen Name: 05Dec
Tube Name: Ox_002
Record Date: Dec 5, 2016 4:26:04 PM
$OP: Administrator
GUID: 64c36e93-44d9-499b-b0b3-2c3...

Population	#Events	%Parent	FITC-A Median	BV510-A Median
S4	41,437	96.8	9,612	815
GFP+	29,233	70.5	15,378	1,327
Ox	888	3.0	2,395	685
Red	124	0.4	1,792	33

BD FACSDiva 8.0.1

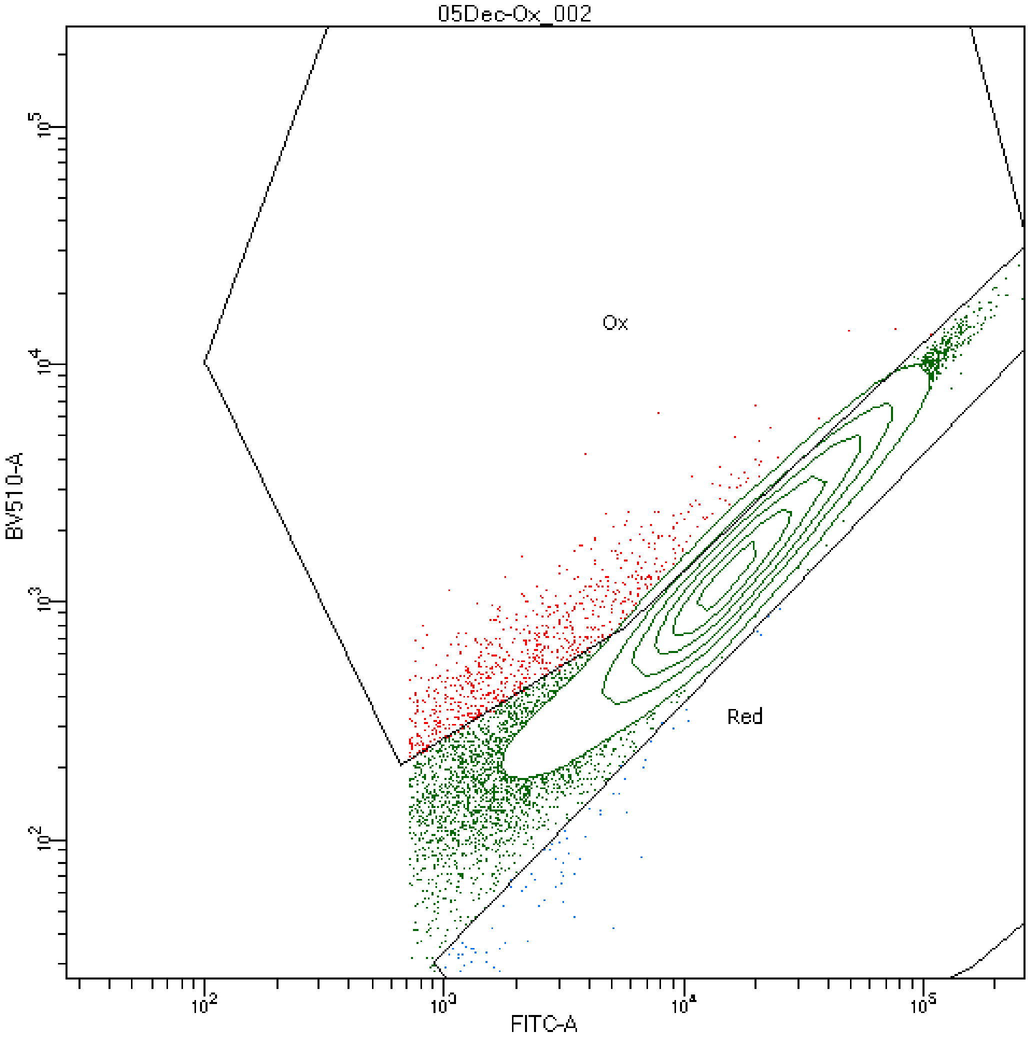

BD FACSDiva 8.0.1

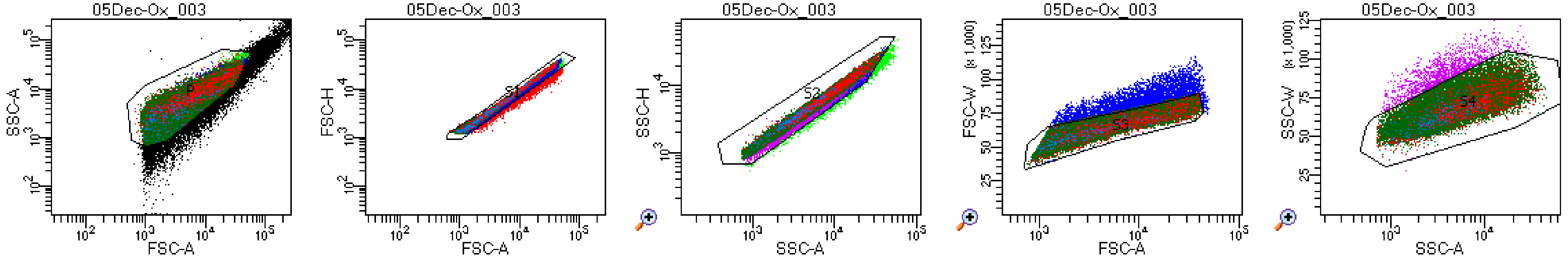

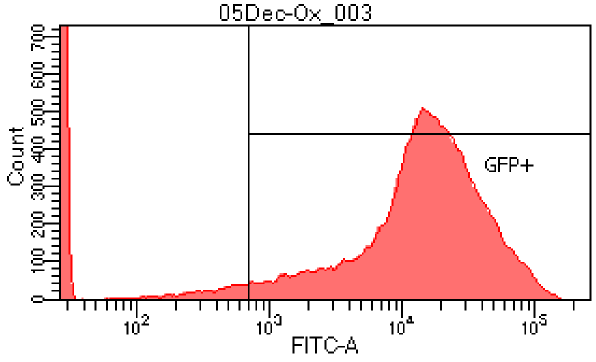

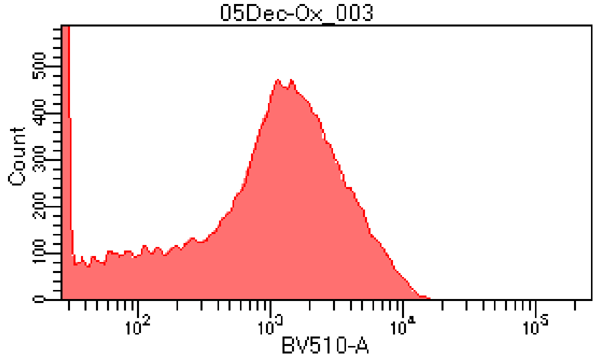

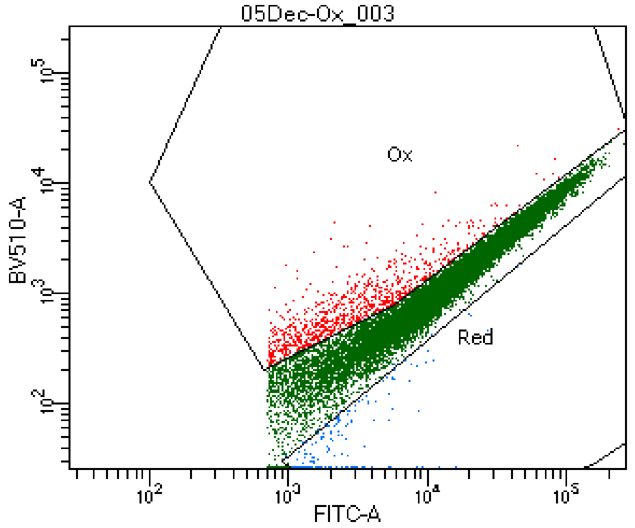

Tube: Ox_003

Population	#Events	%Parent	%Total
All Events	58,647	####	100.0
P	50,000	85.3	85.3
S1	48,536	97.1	82.8
S2	47,643	98.2	81.2
S3	42,963	90.2	73.3
S4	41,573	96.8	70.9
GFP+	29,425	70.8	50.2
Ox	906	3.1	1.5
Red	172	0.6	0.3

Experiment Name: 05EDec2016 Bac sorting
Specimen Name: 05Dec
Tube Name: Ox_003
Record Date: Dec 5, 2016 4:26:38 PM
$OP: Administrator
GUID: 9b49a9cc-c153-4f87-93a6-126...

Population	#Events	%Parent	FITC-A Median	BV510-A Median
S4	41,573	96.8	9,841	820
GFP+	29,425	70.8	15,676	1,341
Ox	906	3.1	2,513	708
Red	172	0.6	1,993	37

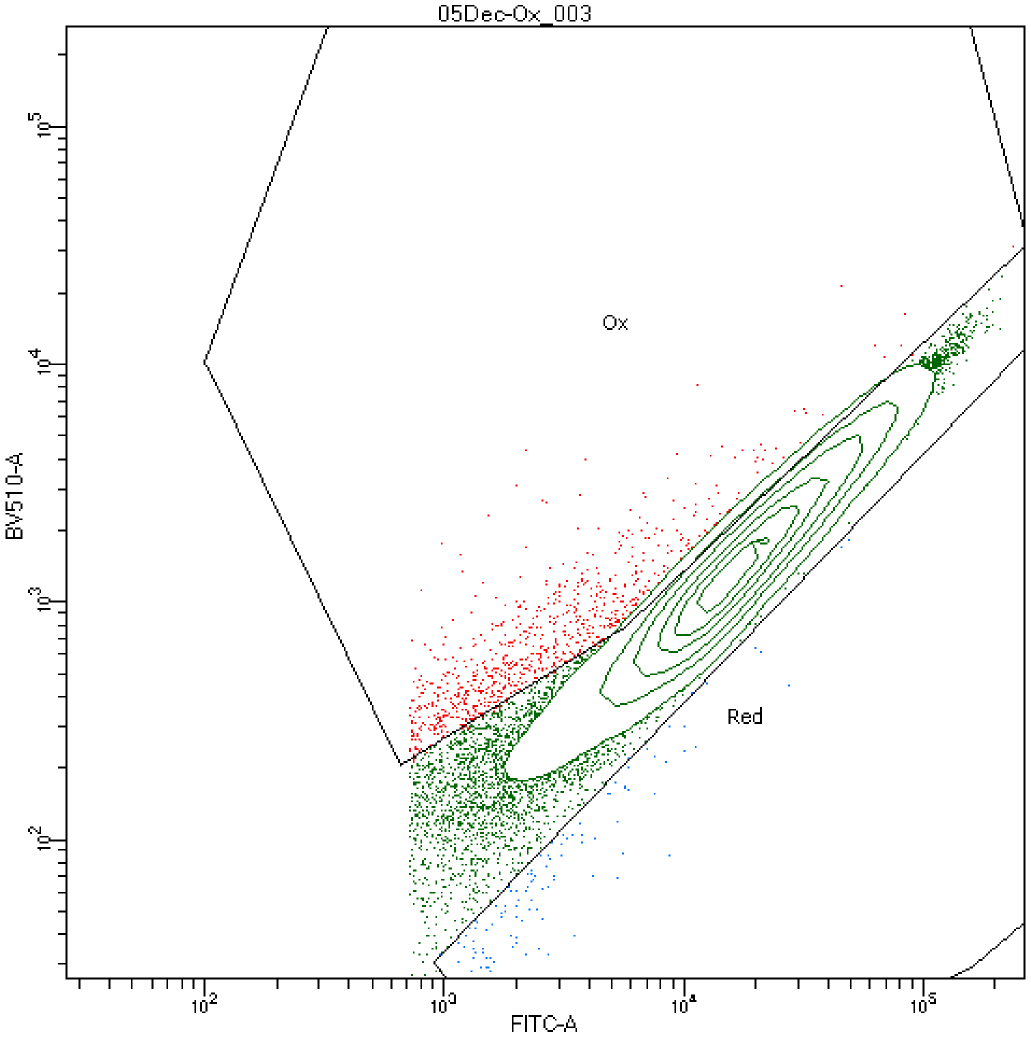
05Dec-Ox_003
Ox
Red
BV510-A
FITC-A
10^2
10^3
10^4
10^5

BD FACSDiva 8.0.1

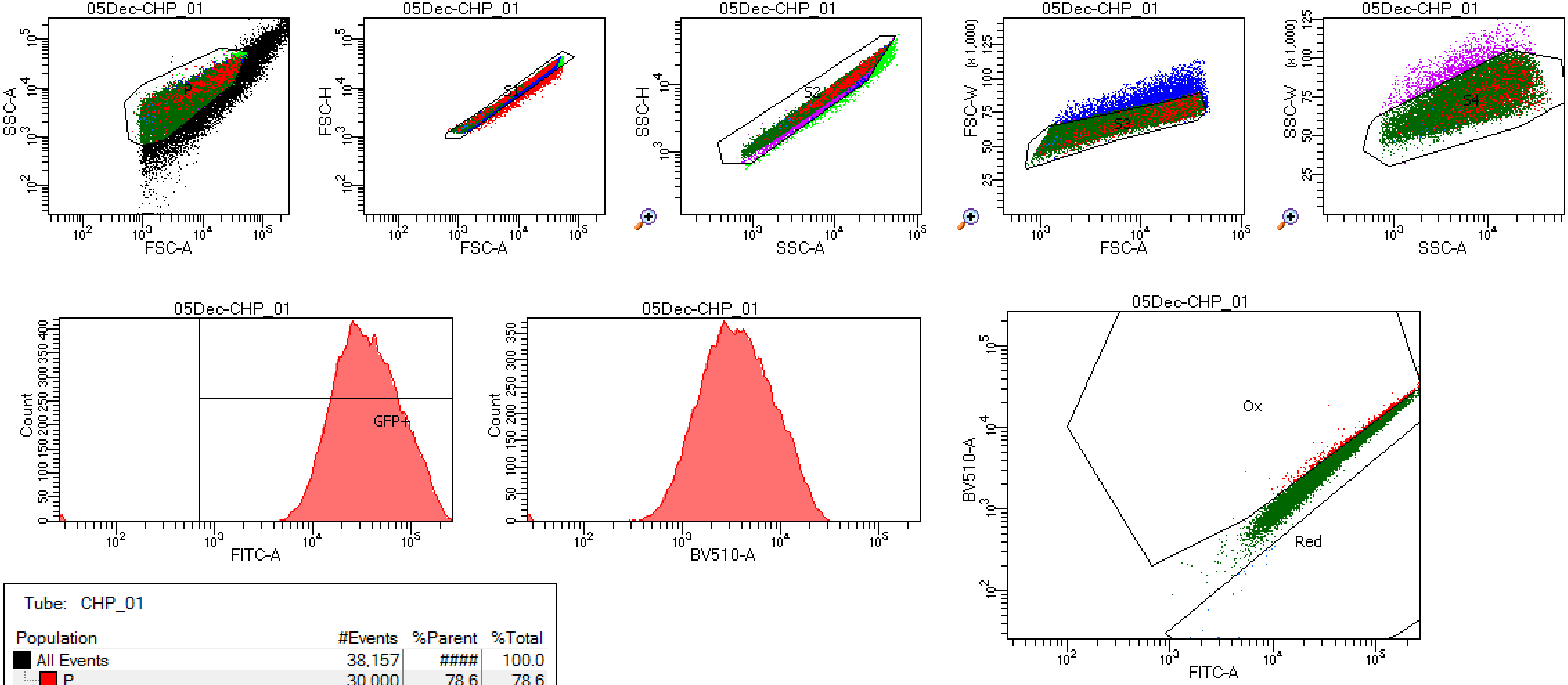

Tube: CHP_01

Population	#Events	%Parent	%Total
All Events	38,157	####	100.0
P	30,000	78.6	78.6
S1	28,951	96.5	75.9
S2	27,801	96.0	72.9
S3	24,211	87.1	63.5
S4	23,006	95.0	60.3
GFP+	22,963	99.8	60.2
Ox	676	2.9	1.8
Red	20	0.1	0.1

Experiment Name:	05EDec2016 Bac sorting
Specimen Name:	05Dec
Tube Name:	CHP_01
Record Date:	Dec 5, 2016 4:28:58 PM
$OP:	Administrator
GUID:	de7e91f5-35f1-4e28-a8a8-a161...

Population	#Events	%Parent	FITC-A Median	BV510-A Median
S4	23,006	95.0	33,763	3,400
GFP+	22,963	99.8	33,833	3,407
Ox	676	2.9	89,685	11,703
Red	20	0.1	4,738	129

BD FACSDiva 8.0.1

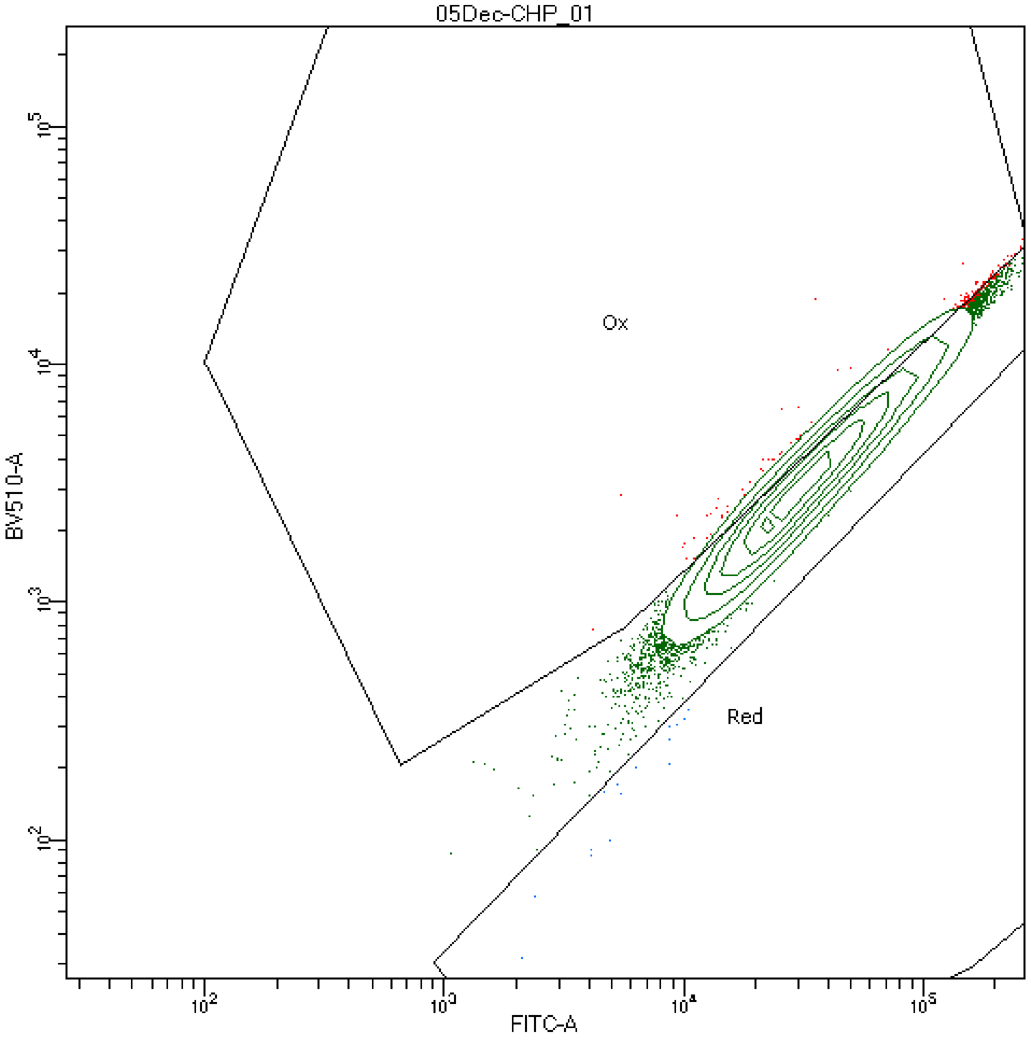

BD FACSDiva 8.0.1

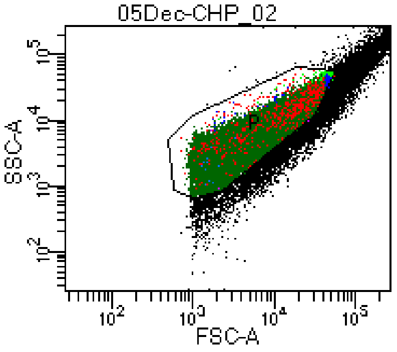

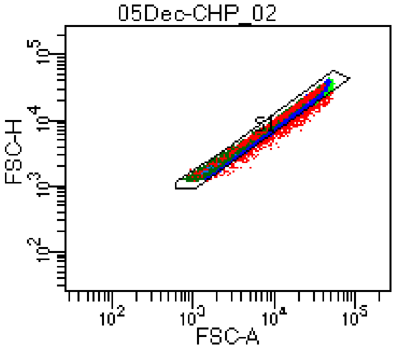

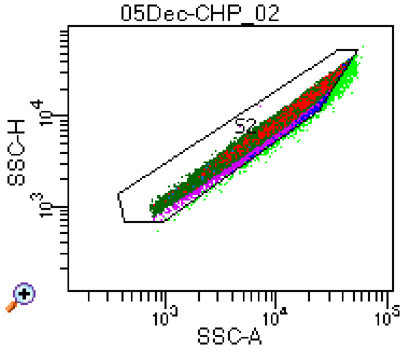

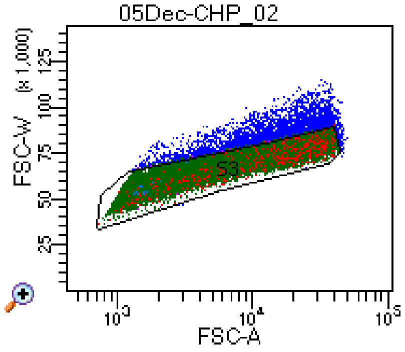

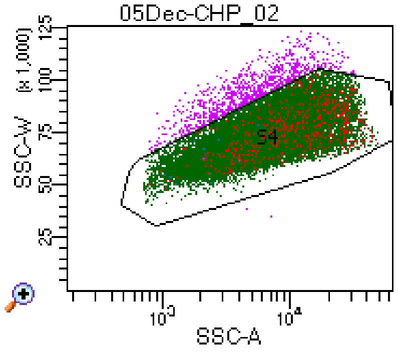

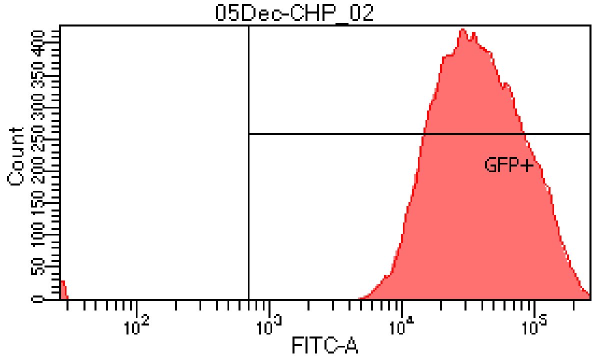

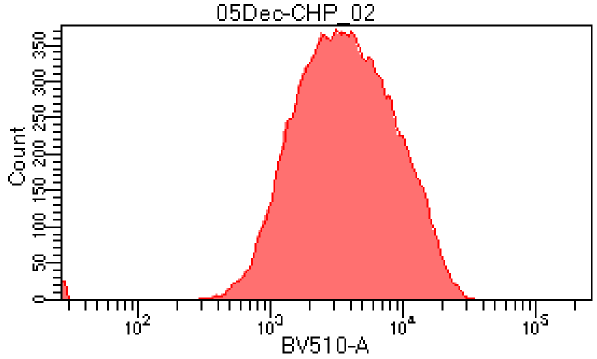

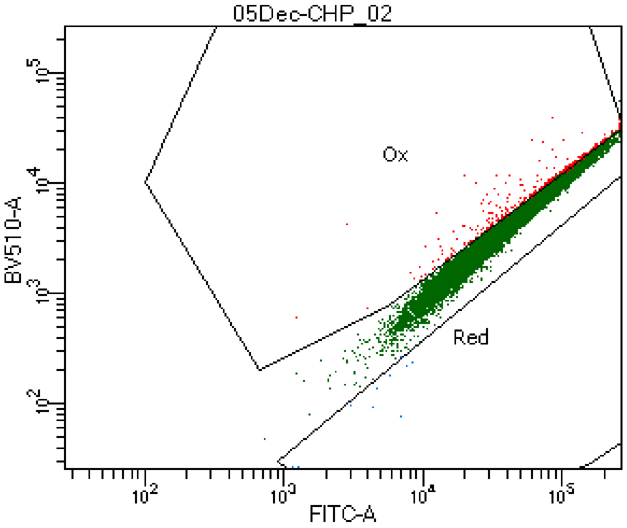

Tube: CHP_02

Population	#Events	%Parent	%Total
All Events	39,242	####	100.0
P	30,000	76.4	76.4
S1	29,258	97.5	74.6
S2	28,379	97.0	72.3
S3	25,294	89.1	64.5
S4	24,323	96.2	62.0
GFP+	24,240	99.7	61.8
Ox	473	2.0	1.2
Red	11	0.0	0.0

Experiment Name:	05EDec2016 Bac sorting
Specimen Name:	05Dec
Tube Name:	CHP_02
Record Date:	Dec 5, 2016 4:29:45 PM
$OP:	Administrator
GUID:	c3eab172-195a-4b10-b966-e27...

Population	#Events	%Parent	FITC-A Median	BV510-A Median
S4	24,323	96.2	35,174	3,454
GFP+	24,240	99.7	35,328	3,465
Ox	473	2.0	92,899	12,537
Red	11	0.0	4,576	97

BD FACSDiva 8.0.1

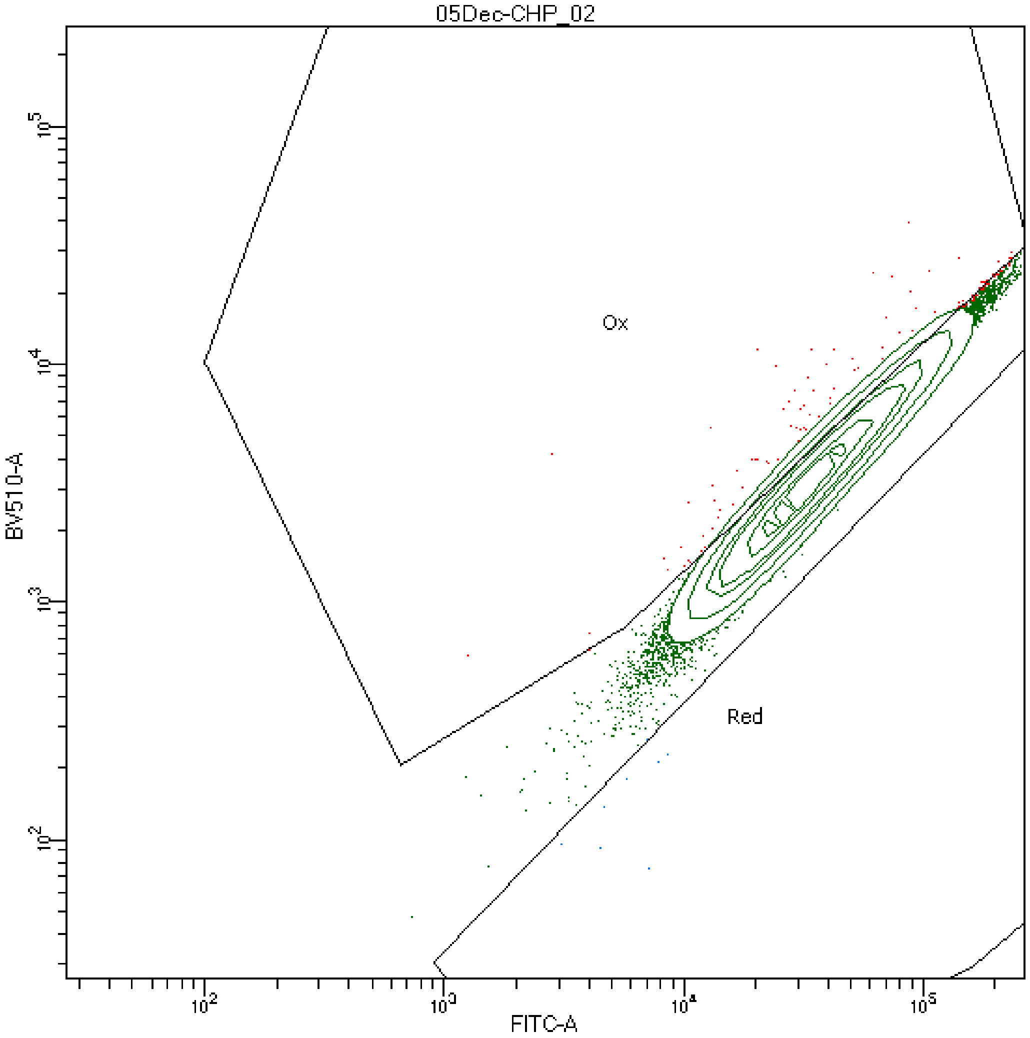

Supplement: Figure 1—source data 1. [file elife-80218-fig1-data1.zip › Round 2 Sorting/05EDec2016 Bac sorting-Batch_Analysis_05122016163007.pdf]
