# Supplementary material for: Biosensor-integrated transposon mutagenesis reveals rv0158 as a coordinator of redox homeostasis in Mycobacterium tuberculosis
Source: eLife. 2023 Aug 29;12:e80218. doi: 10.7554/eLife.80218 (PMC10501769; doi:10.7554/eLife.80218)

Batch Analysis Report

Run Date: 12/12/16 3:11 PM Experiment: 05EDec2016 Bac sorting

User ID: Administrator

Statistics Output: N/A

Worksheet PDF Output: C:\Users\Admin\Desktop\05EDec2016 Bac sorting-Batch_Analysis_1212 2016151104.pdf

12Dec

Tube	Status	Run Time
US	OK	12/12/16 3:11 PM
RV Mrx1	OK	12/12/16 3:11 PM
RV Mrx1_Richa	OK	12/12/16 3:11 PM
CHP	OK	12/12/16 3:11 PM
CHP_001	OK	12/12/16 3:11 PM
DTT	OK	12/12/16 3:11 PM
DTT_001	OK	12/12/16 3:11 PM
Tn Lib_only hyg	OK	12/12/16 3:11 PM
TN lib	OK	12/12/16 3:11 PM
Ox Post Sort	OK	12/12/16 3:11 PM
Red Post Sort	OK	12/12/16 3:11 PM

BD FACSDiva 8.0.1

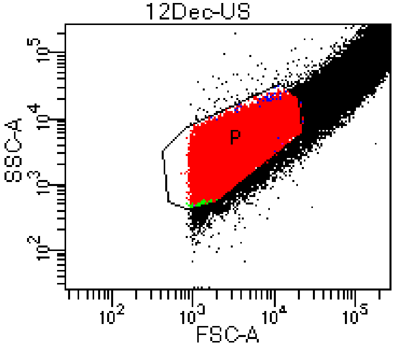

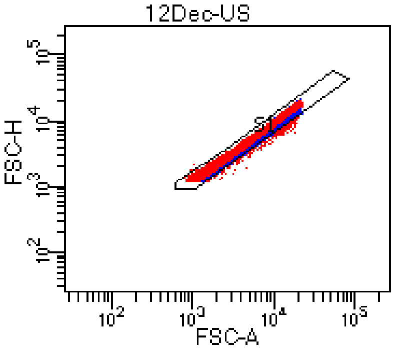

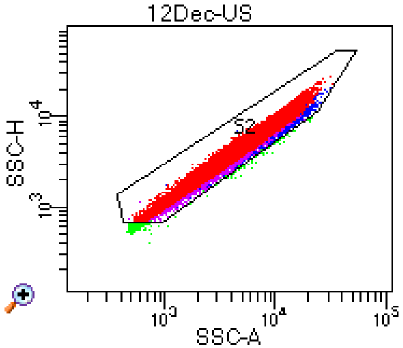

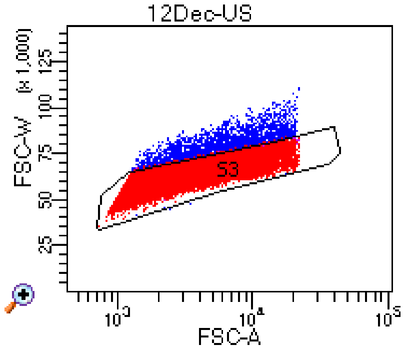

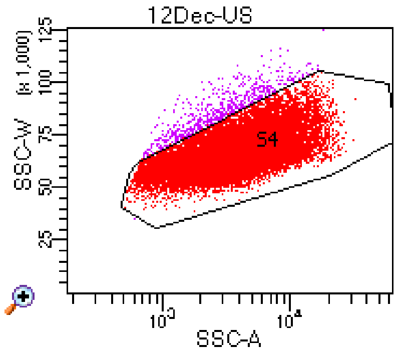

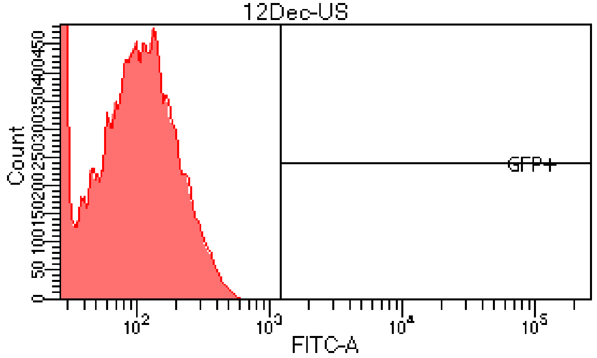

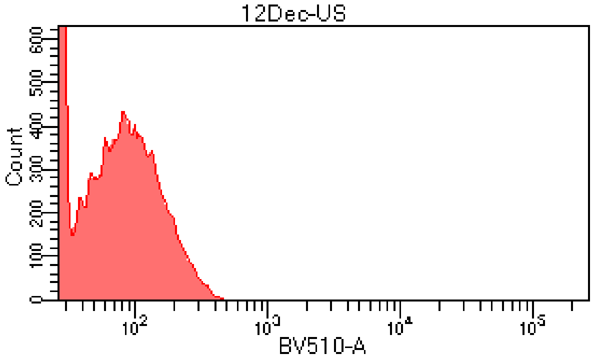

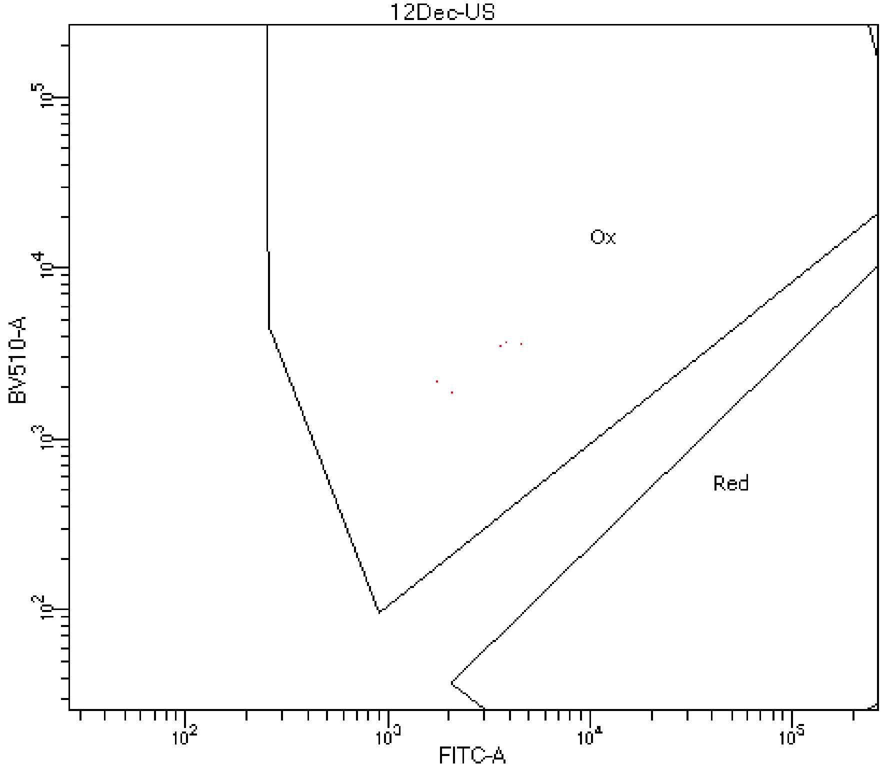

Tube: US

Population	#Events	%Parent	%Total
All Events	78,920	####	100.0
P	28,500	36.1	36.1
S1	28,153	98.8	35.7
S2	27,664	98.3	35.1
S3	25,468	92.1	32.3
S4	24,833	97.5	31.5
GFP+	5	0.0	0.0
Ox	5	100.0	0.0
Red	0	0.0	0.0

Experiment Name:	05EDec2016 Bac sorting
Specimen Name:	12Dec
Tube Name:	US
Record Date:	Dec 12, 2016 11:11:42 AM
$OP:	Administrator
GUID:	b88a611d-6fad-4730-a016-ba816c6...

Population	#Events	%Parent	FITC-A Median	BV510-A Median
S4	24,833	97.5	86	61
GFP+	5	0.0	3,609	3,476
Ox	5	100.0	3,609	3,476
Red	0	0.0	####	####

BD FACSDiva 8.0.1

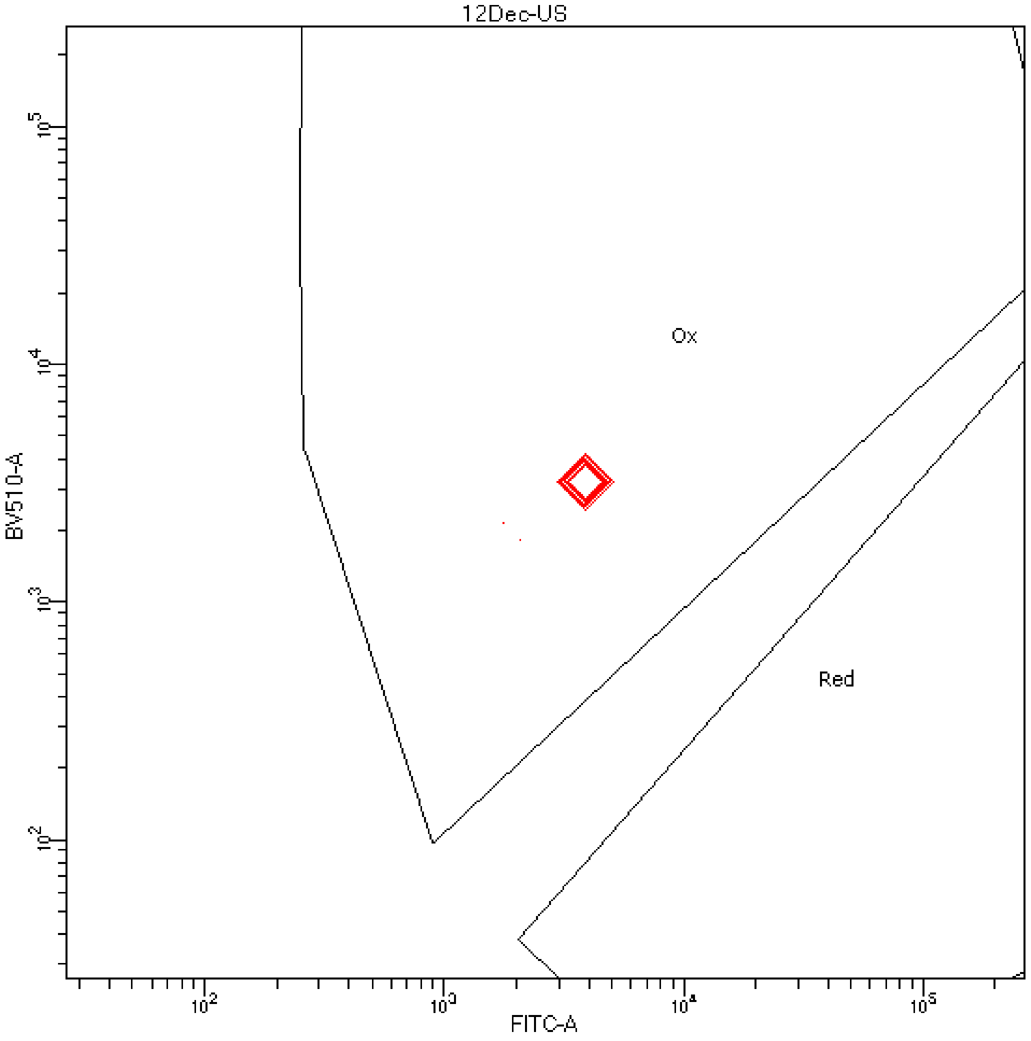

BD FACSDiva 8.0.1

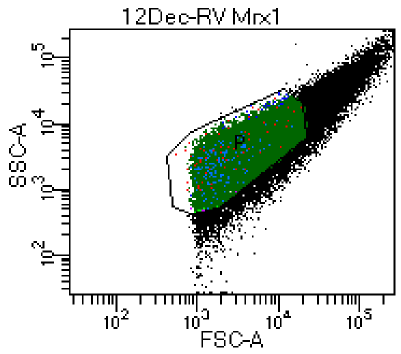

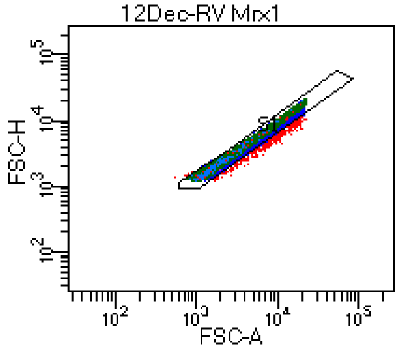

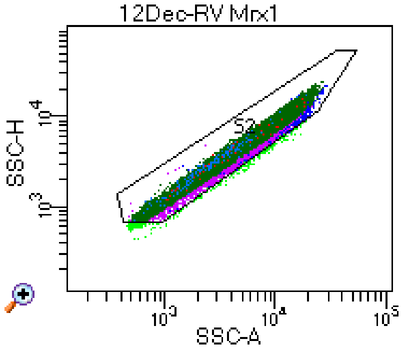

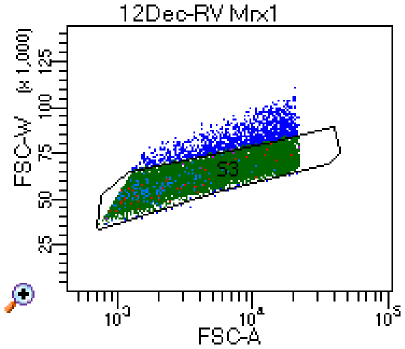

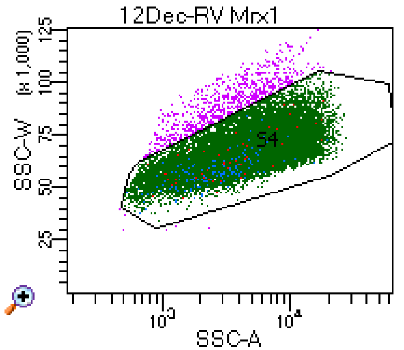

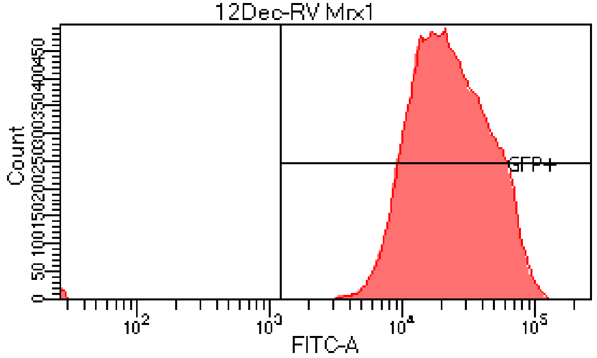

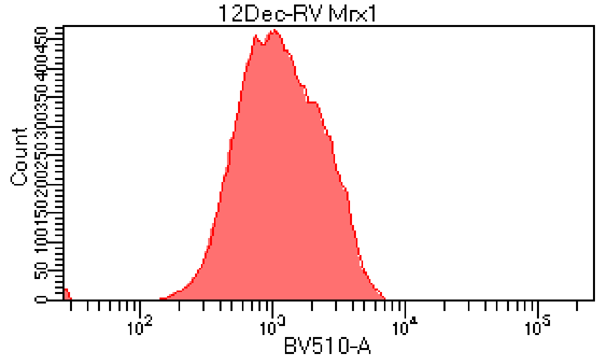

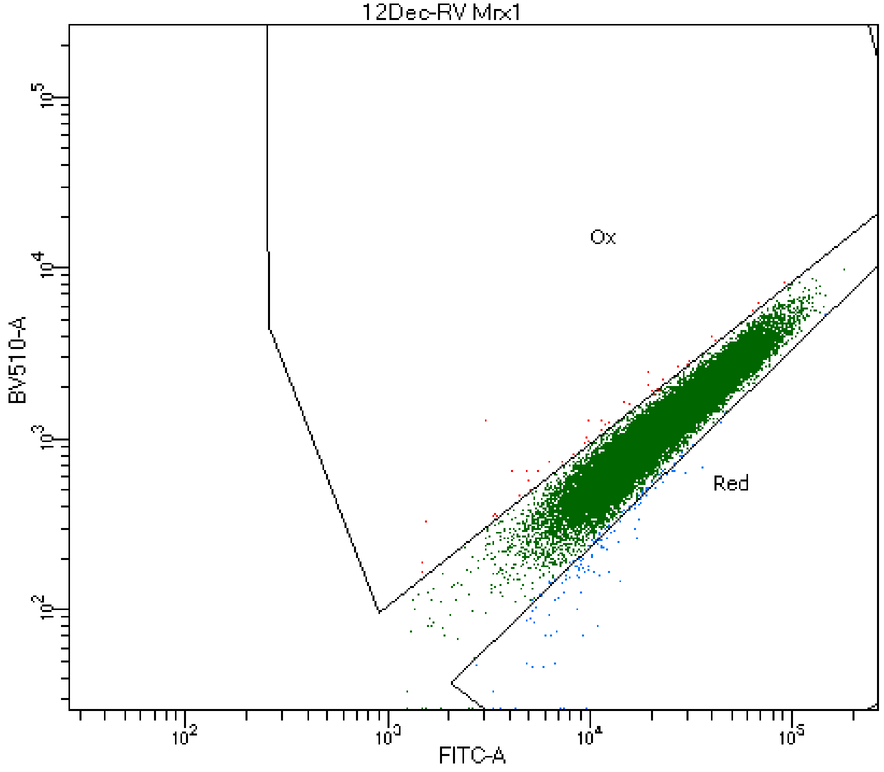

Tube: RV Mrx1

Population	#Events	%Parent	%Total
All Events	39,084	####	100.0
P	28,099	71.9	71.9
S1	27,597	98.2	70.6
S2	27,171	98.5	69.5
S3	25,557	94.1	65.4
S4	24,758	96.9	63.3
GFP+	24,678	99.7	63.1
Ox	49	0.2	0.1
Red	141	0.6	0.4

Experiment Name: 05EDec2016 Bac sorting
Specimen Name: 12Dec
Tube Name: RV Mrx1
Record Date: Dec 12, 2016 11:12:21 AM
$OP: Administrator
GUID: 75acde8a-fcc9-4979-a59f-692f543b...

Population	#Events	%Parent	FITC-A Median	BV510-A Median
S4	24,758	96.9	21,184	1,065
GFP+	24,678	99.7	21,248	1,067
Ox	49	0.2	11,408	1,274
Red	141	0.6	10,954	233

BD FACSDiva 8.0.1

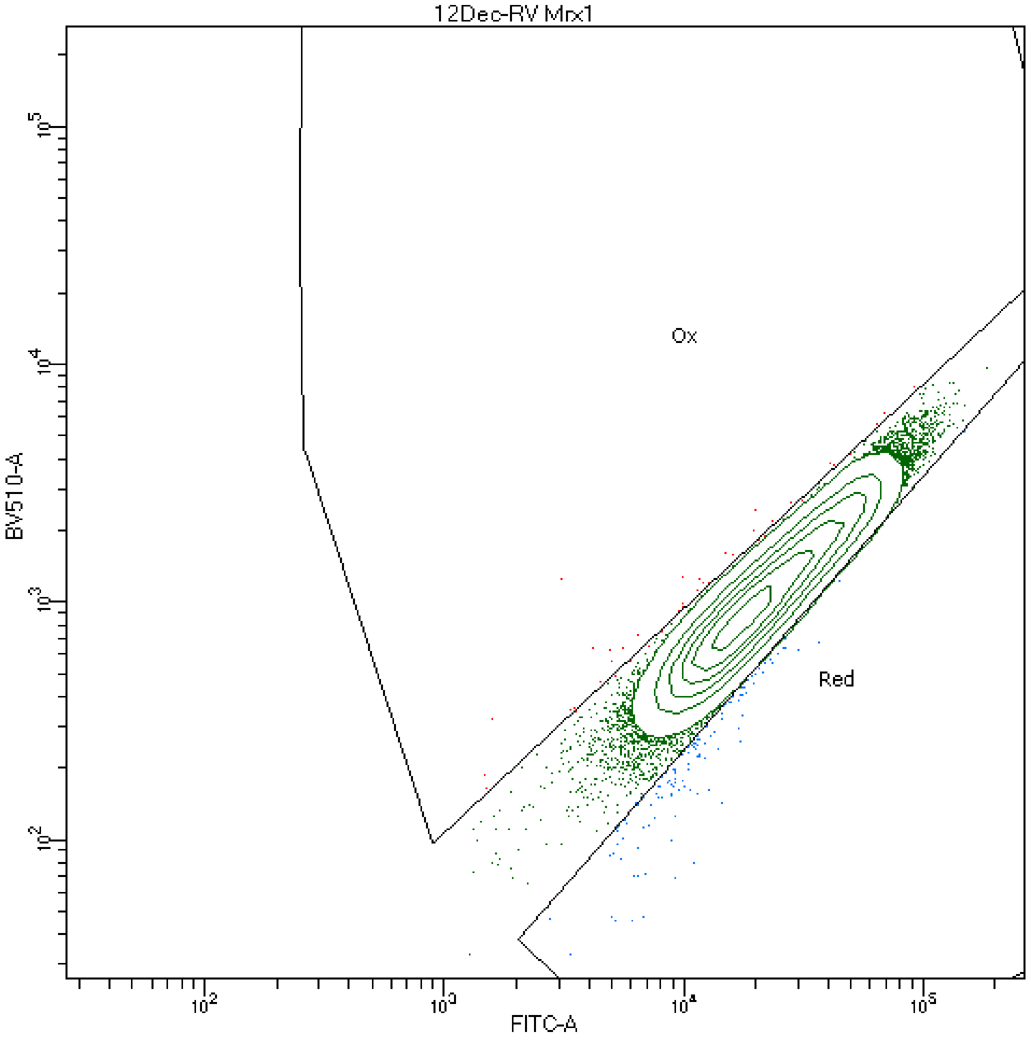

BD FACSDiva 8.0.1

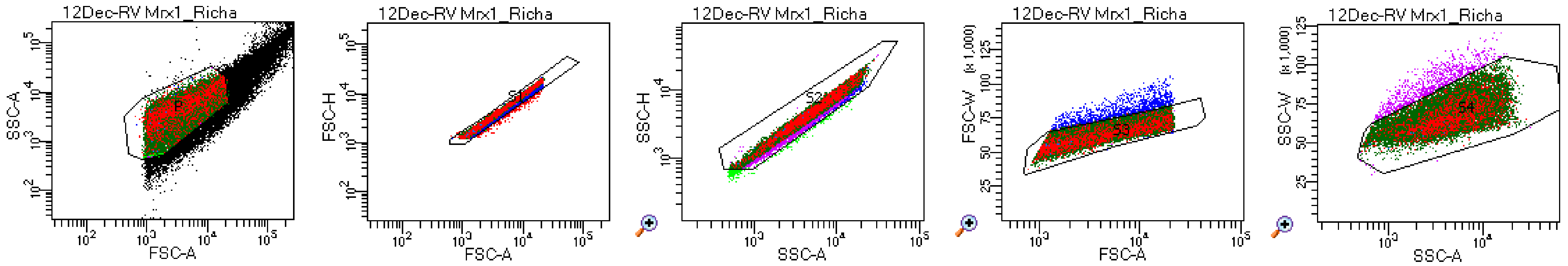

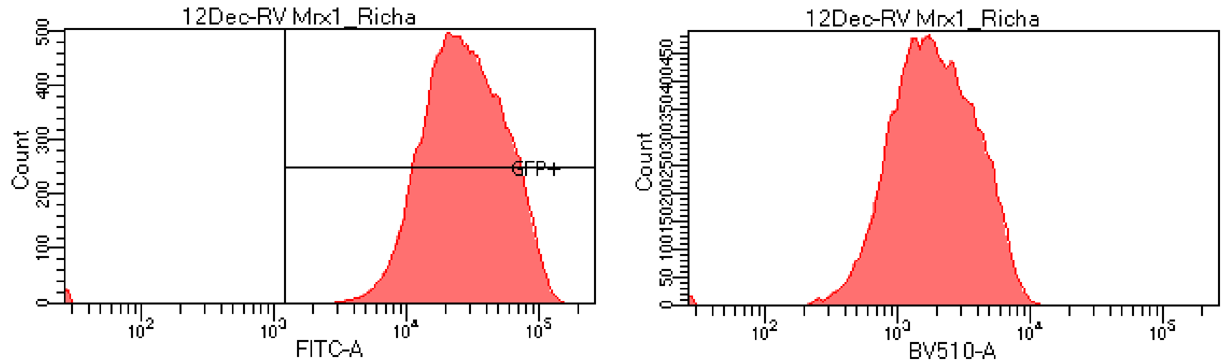

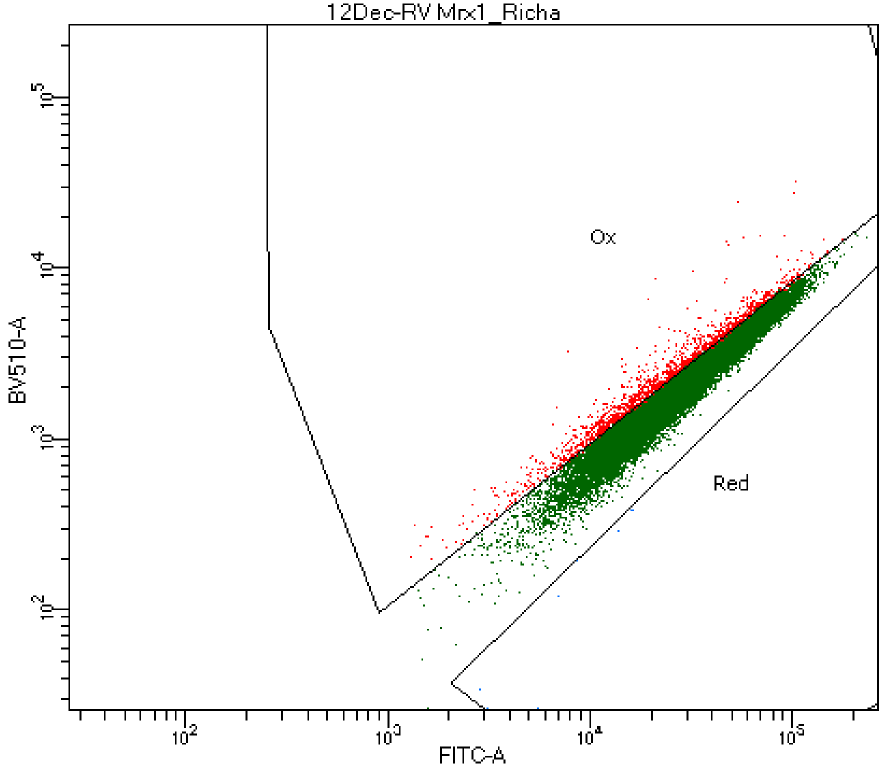

Tube: RV Mrx1_Richa

Population	#Events	%Parent	%Total
All Events	41,273	####	100.0
P	28,541	69.2	69.2
S1	28,290	99.1	68.5
S2	27,830	98.4	67.4
S3	26,673	95.8	64.6
S4	25,783	96.7	62.5
GFP+	25,684	99.6	62.2
Ox	1,570	6.1	3.8
Red	10	0.0	0.0

Experiment Name:	05EDec2016 Bac sorting
Specimen Name:	12Dec
Tube Name:	RV Mrx1_Richa
Record Date:	Dec 12, 2016 11:13:00 AM
$OP:	Administrator
GUID:	ab3c38ff-0e55-4429-bd37-2290bae...

Population	#Events	%Parent	FITC-A Median	BV510-A Median
S4	25,783	96.7	26,042	1,778
GFP+	25,684	99.6	26,125	1,784
Ox	1,570	6.1	21,920	2,229
Red	10	0.0	7,754	157

BD FACSDiva 8.0.1

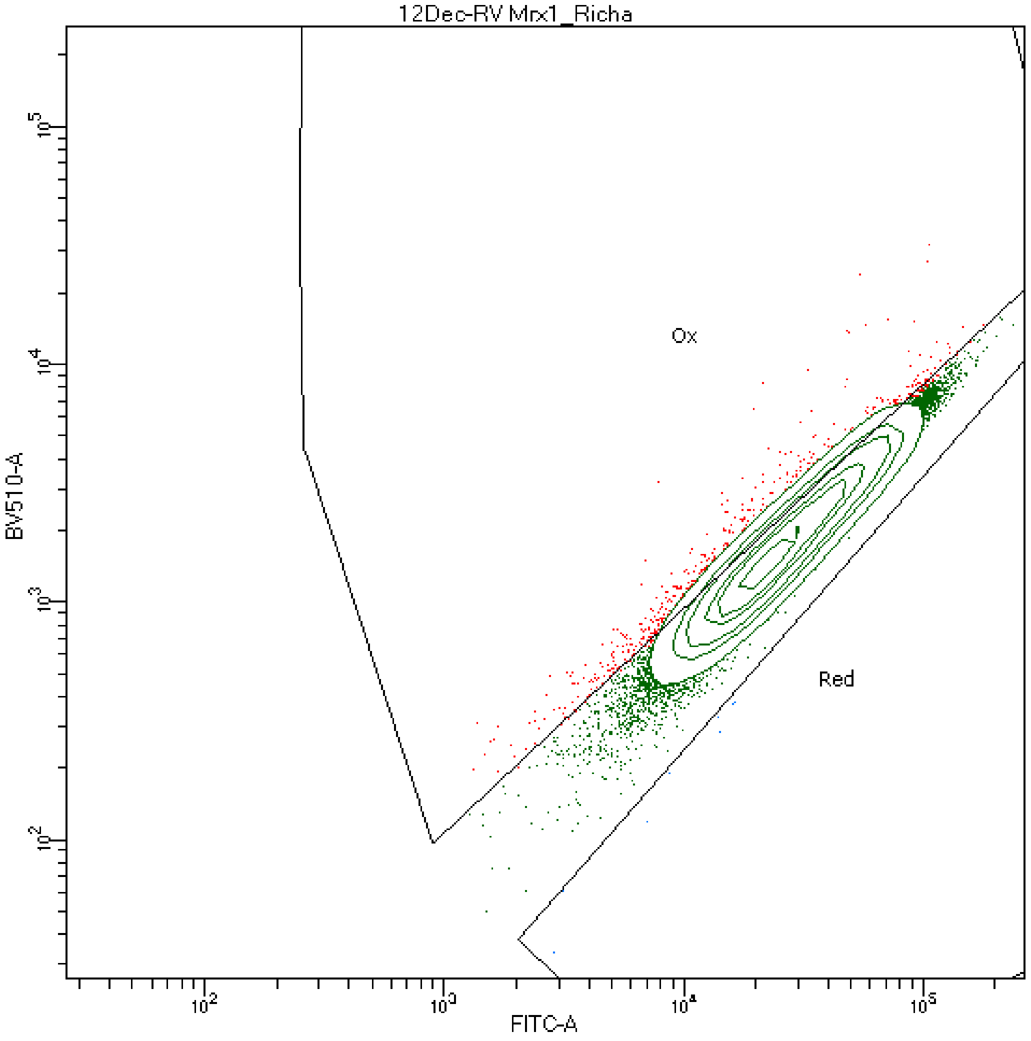

BD FACSDiva 8.0.1

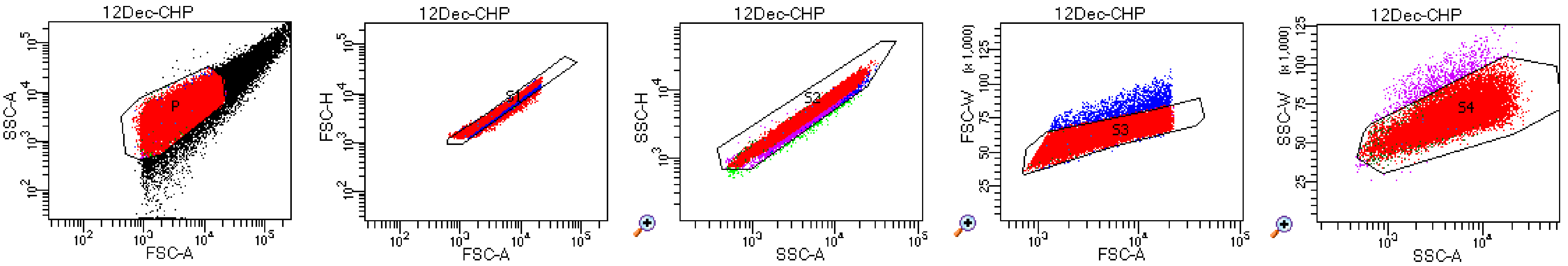

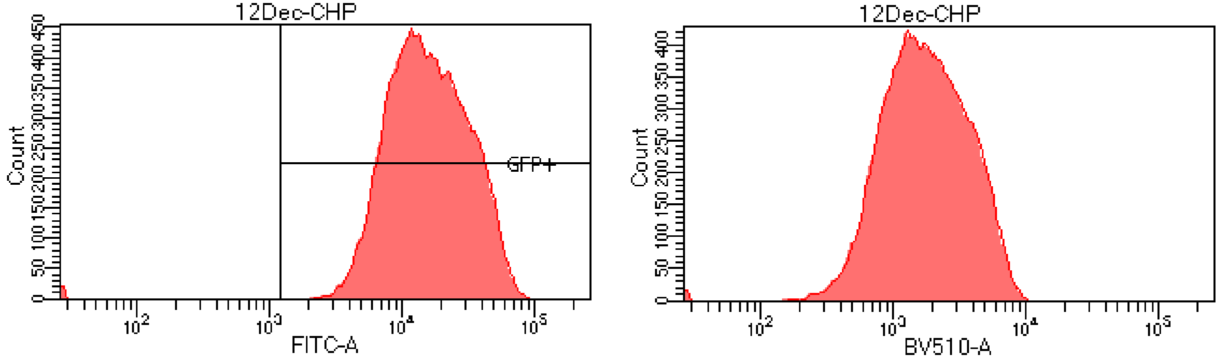

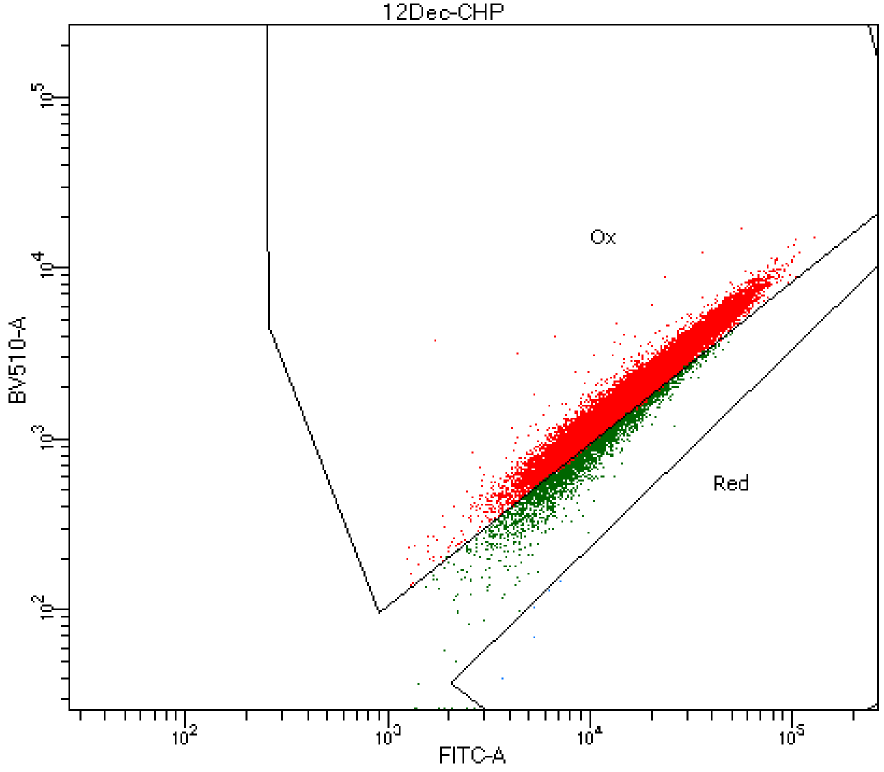

Tube: CHP

Population	#Events	%Parent	%Total
All Events	38,397	####	100.0
P	26,776	69.7	69.7
S1	26,030	97.2	67.8
S2	25,757	99.0	67.1
S3	23,774	92.3	61.9
S4	22,901	96.3	59.6
GFP+	22,809	99.6	59.4
Ox	19,398	85.0	50.5
Red	5	0.0	0.0

Experiment Name:	05EDec2016 Bac sorting
Specimen Name:	12Dec
Tube Name:	CHP
Record Date:	Dec 12, 2016 11:15:35 AM
$OP:	Administrator
GUID:	1bd45bd7-0565-43f1-bd13-57889ea...

Population	#Events	%Parent	FITC-A Median	BV510-A Median
S4	22,901	96.3	14,823	1,656
GFP+	22,809	99.6	14,892	1,661
Ox	19,398	85.0	16,645	1,906
Red	5	0.0	5,270	103

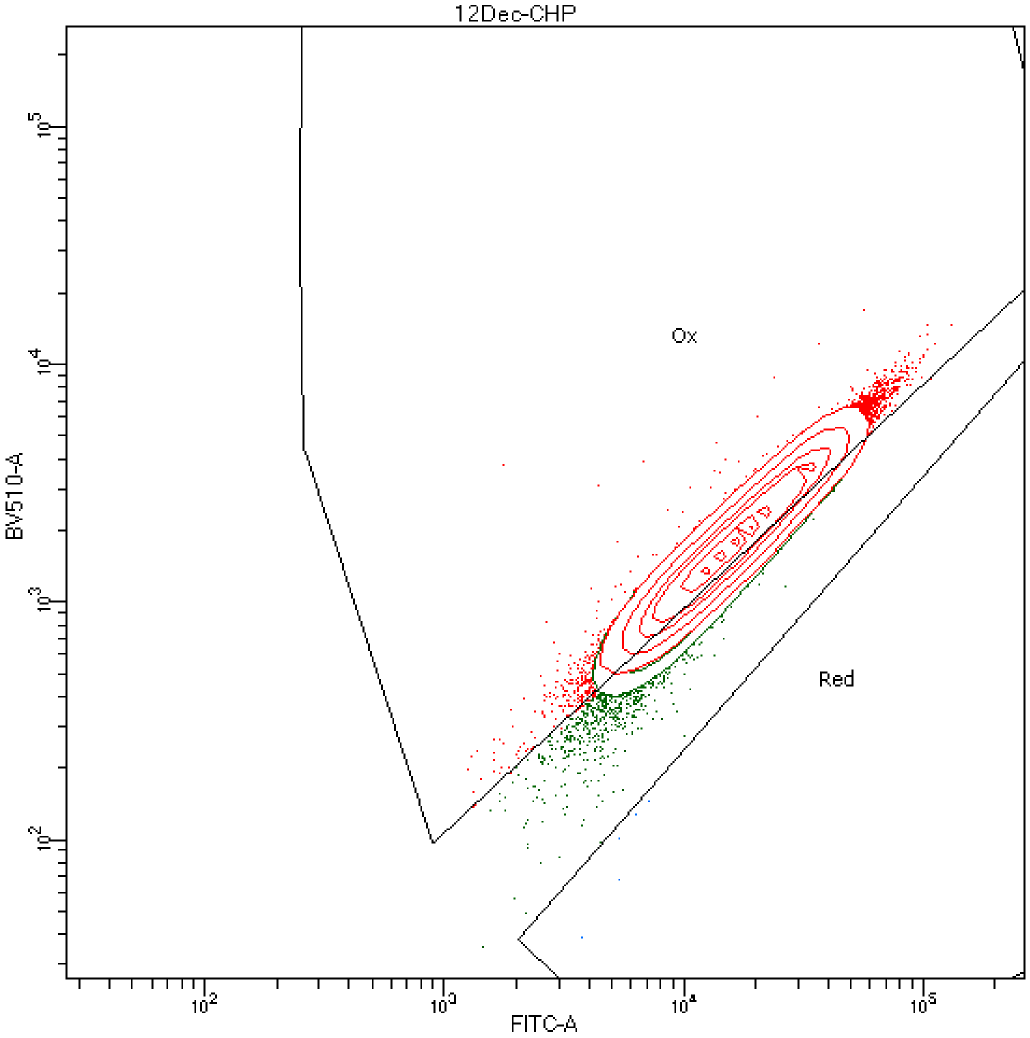
12Dec-CHP
Ox
Red
BV510-A
FITC-A
10^2
10^3
10^4
10^5

BD FACSDiva 8.0.1

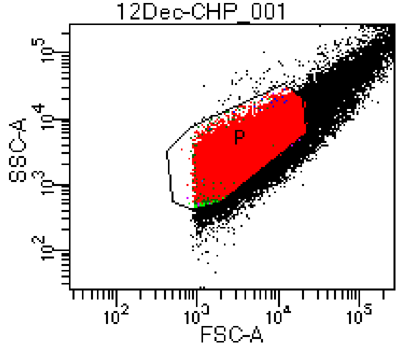

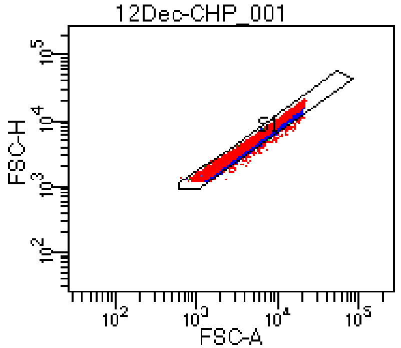

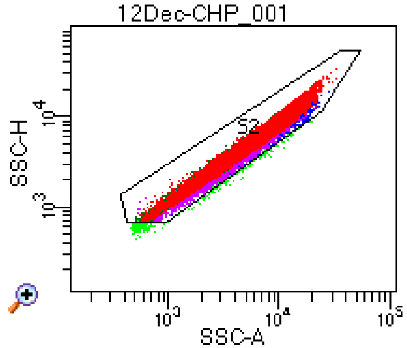

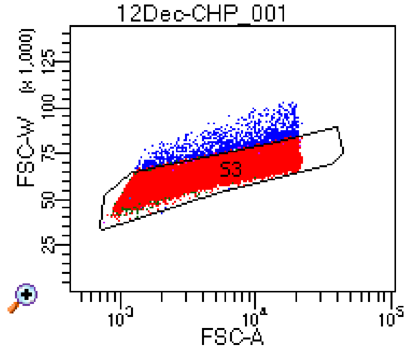

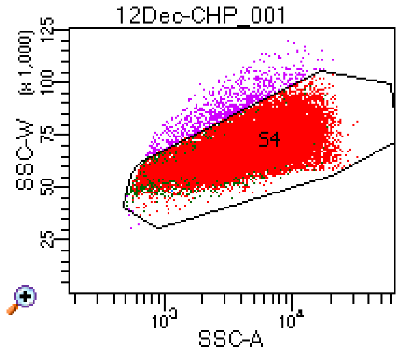

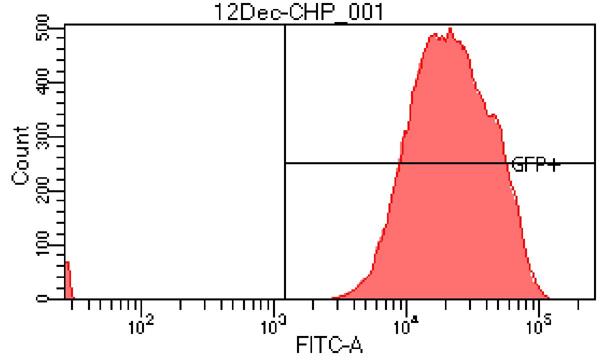

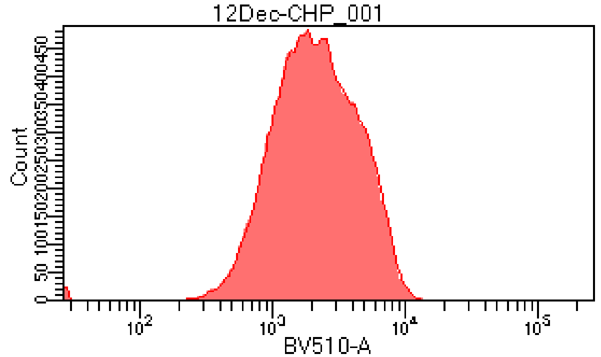

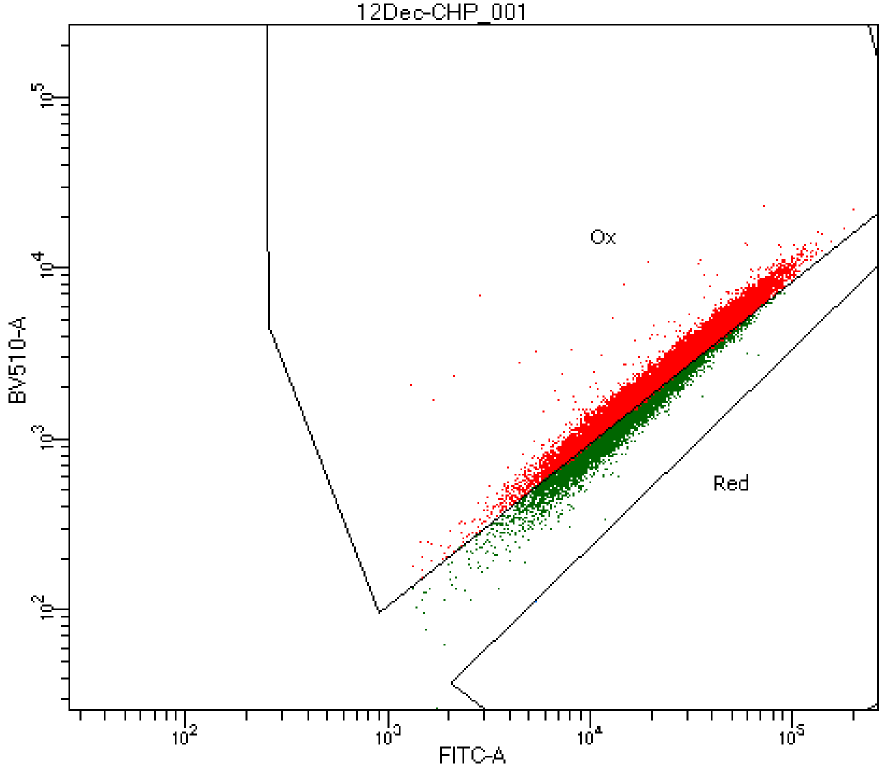

Tube: CHP_001

Population	#Events	%Parent	%Total
All Events	41,443	####	100.0
P	28,792	69.5	69.5
S1	28,604	99.3	69.0
S2	28,141	98.4	67.9
S3	26,892	95.6	64.9
S4	26,080	97.0	62.9
GFP+	25,853	99.1	62.4
Ox	18,163	70.3	43.8
Red	1	0.0	0.0

Experiment Name:	05EDec2016 Bac sorting
Specimen Name:	12Dec
Tube Name:	CHP_001
Record Date:	Dec 12, 2016 11:16:12 AM
$OP:	Administrator
GUID:	adc93f61-4691-4b21-8445-1327397...

Population	#Events	%Parent	FITC-A Median	BV510-A Median
S4	26,080	97.0	20,491	2,002
GFP+	25,853	99.1	20,647	2,011
Ox	18,163	70.3	24,503	2,524
Red	1	0.0	5,419	110

BD FACSDiva 8.0.1

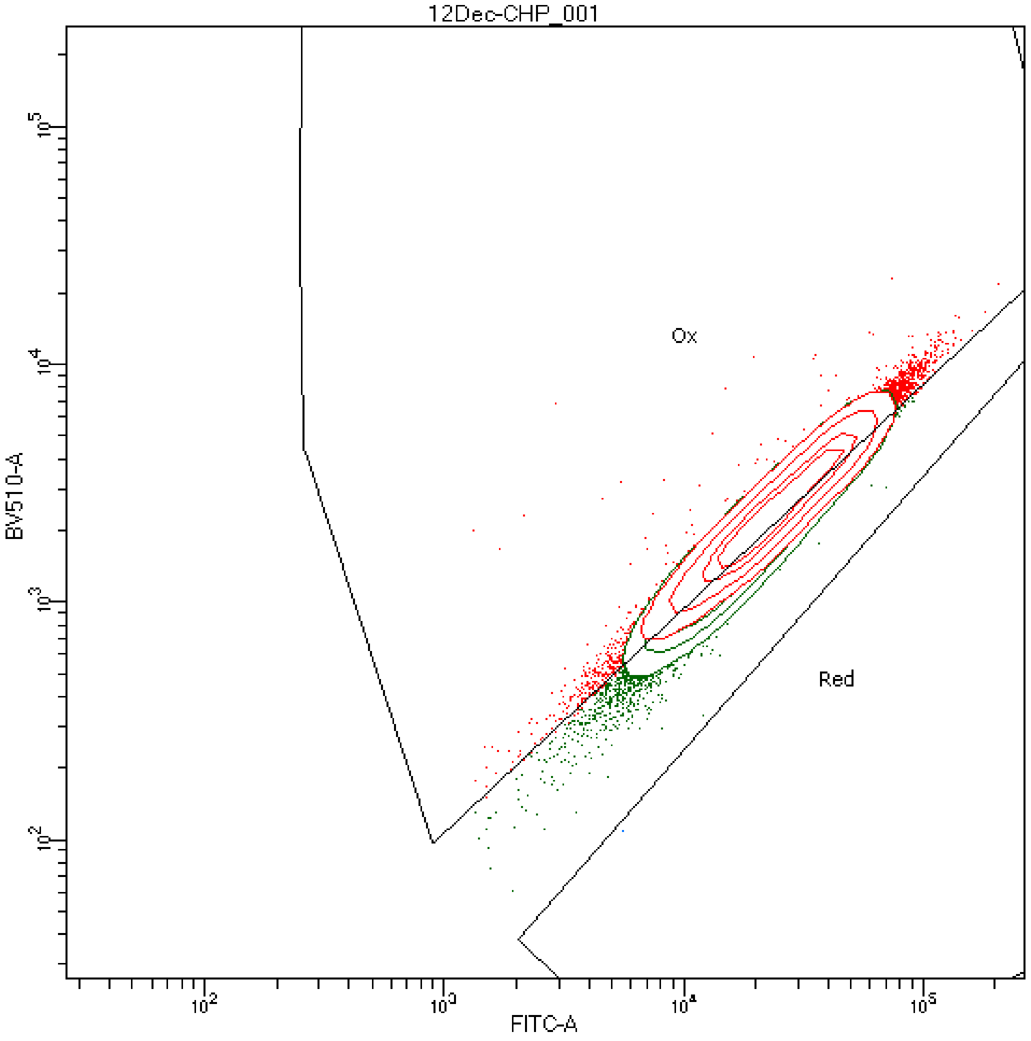

BD FACSDiva 8.0.1

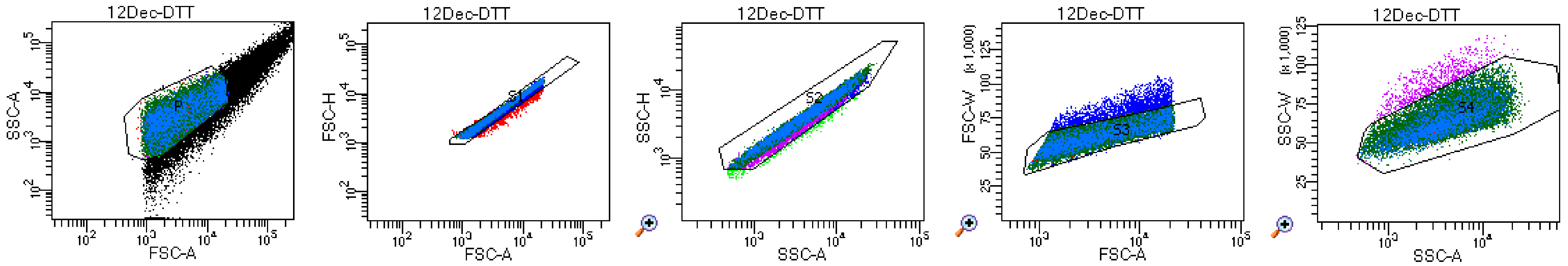

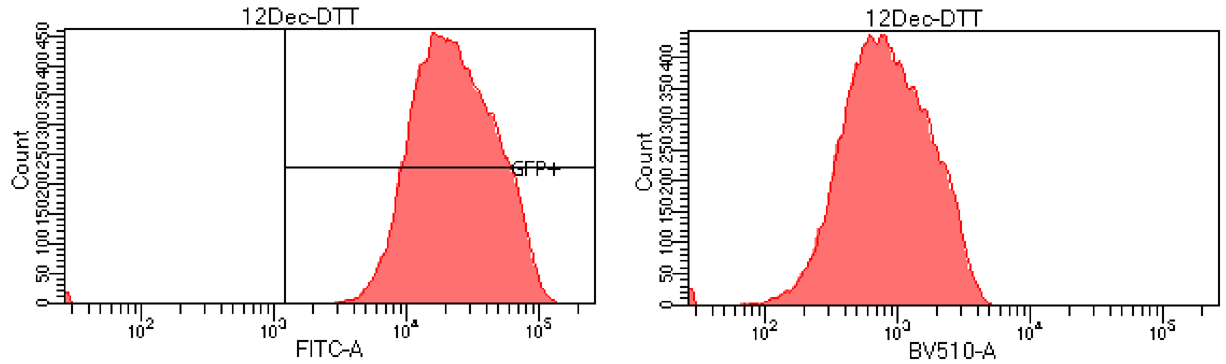

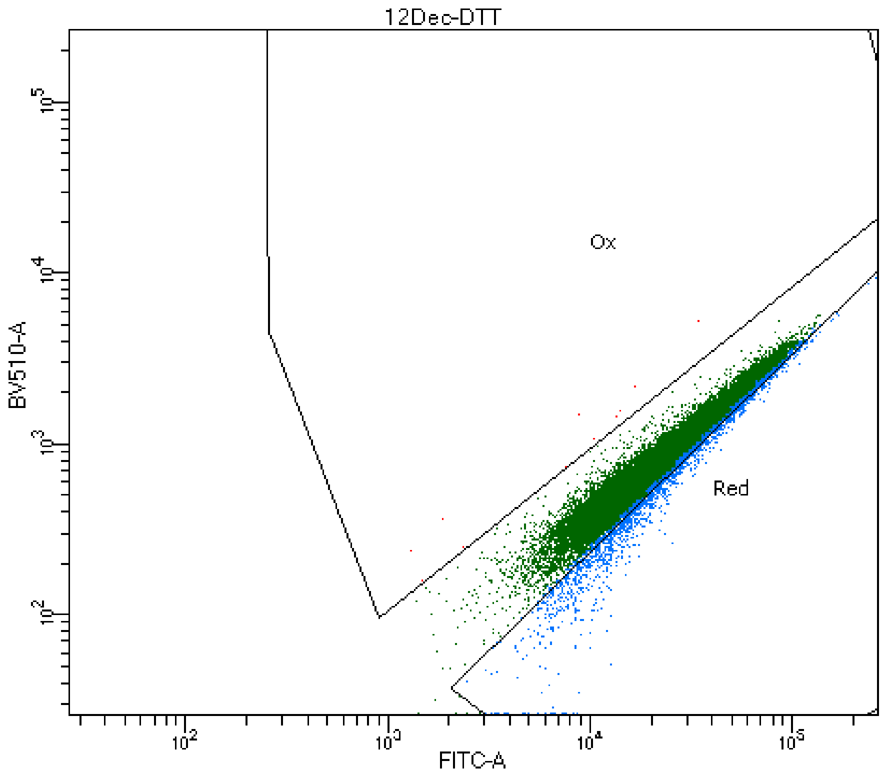

Tube: DTT

Population	#Events	%Parent	%Total
All Events	40,495	####	100.0
P	27,783	68.6	68.6
S1	27,106	97.6	66.9
S2	26,715	98.6	66.0
S3	24,489	91.7	60.5
S4	23,699	96.8	58.5
GFP+	23,614	99.6	58.3
Ox	11	0.0	0.0
Red	2,309	9.8	5.7

Experiment Name: 05EDec2016 Bac sorting
Specimen Name: 12Dec
Tube Name: DTT
Record Date: Dec 12, 2016 11:13:59 AM
$OP: Administrator
GUID: 2f370c86-2a3b-418f-9351-4930318...

Population	#Events	%Parent	FITC-A Median	BV510-A Median
S4	23,699	96.8	21,599	777
GFP+	23,614	99.6	21,669	780
Ox	11	0.0	8,861	1,084
Red	2,309	9.8	22,650	578

BD FACSDiva 8.0.1

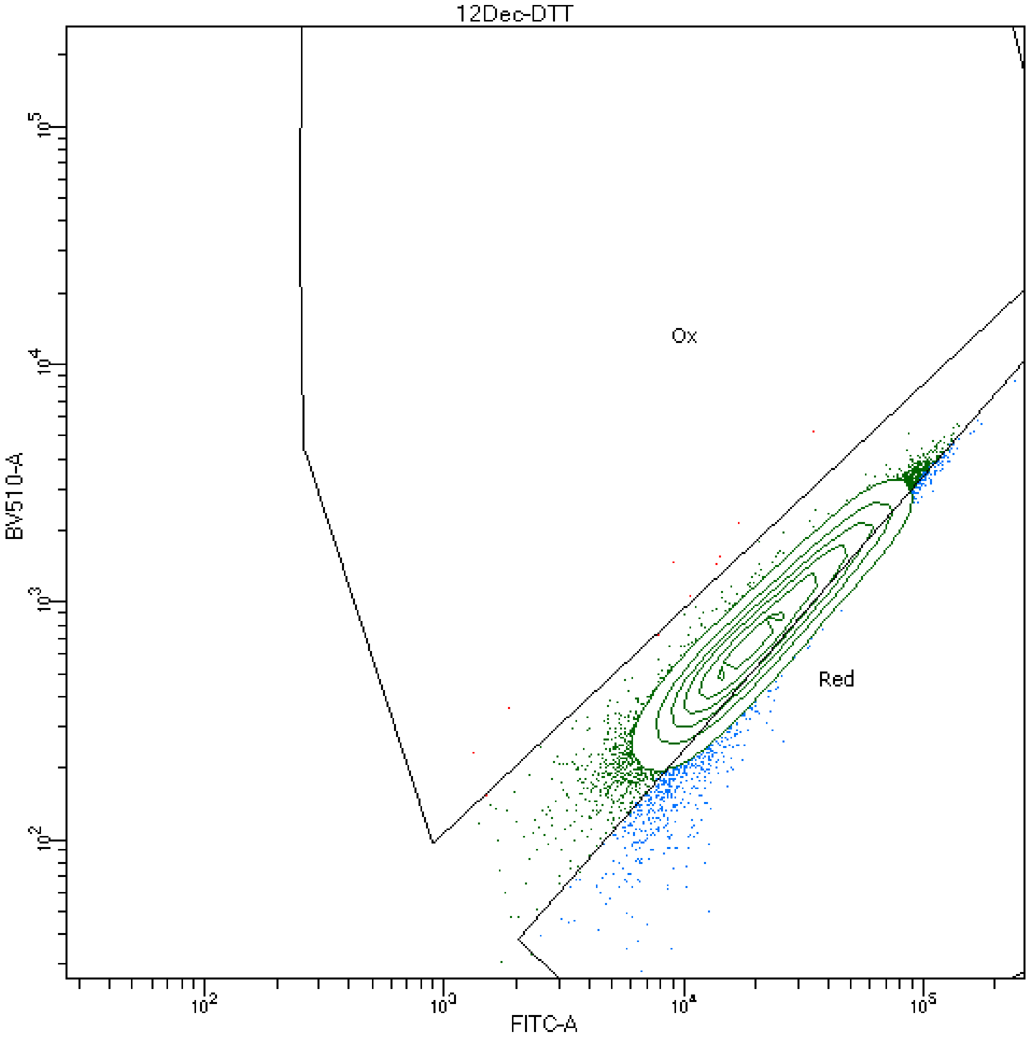

BD FACSDiva 8.0.1

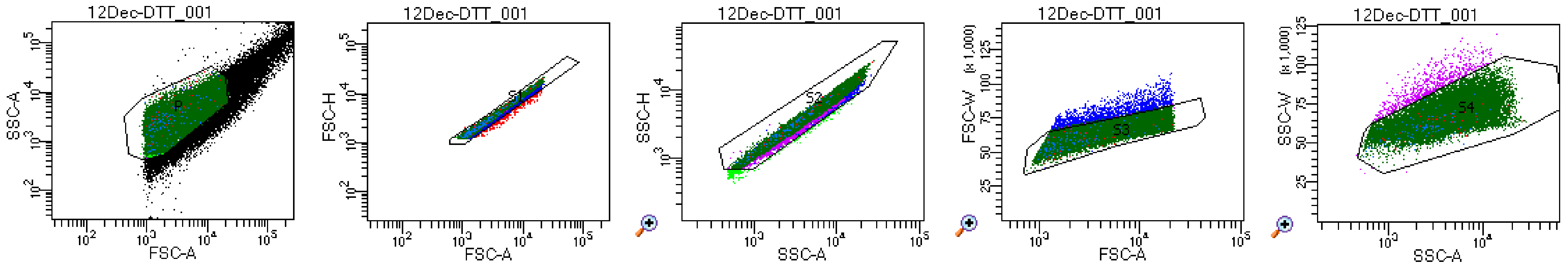

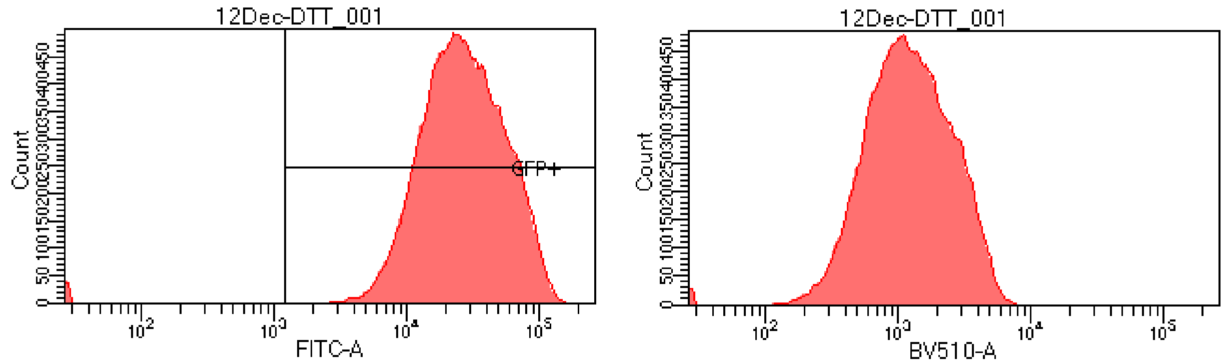

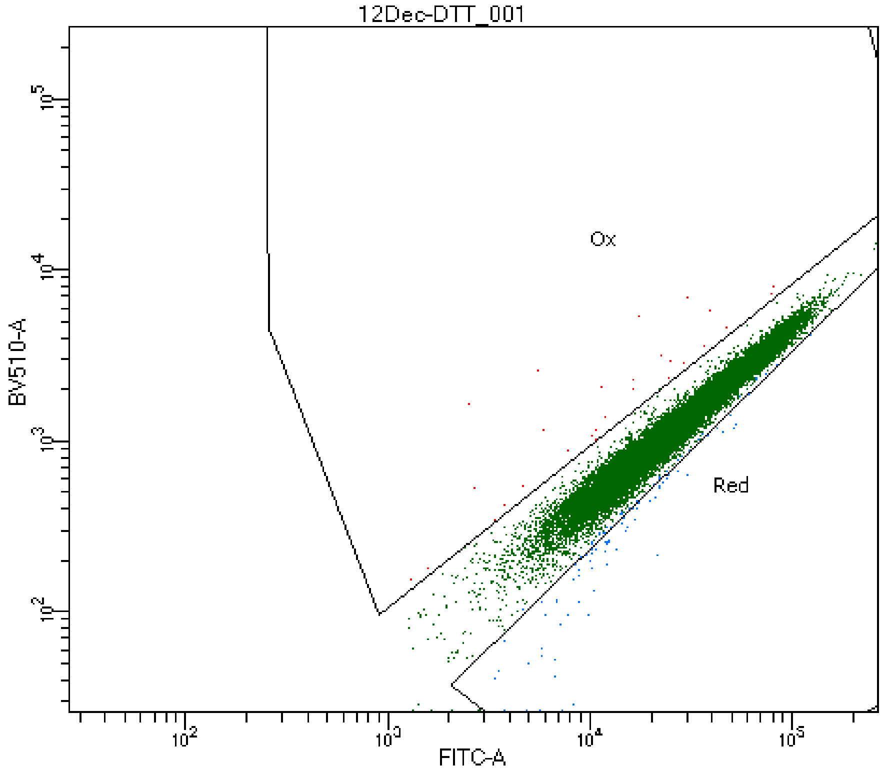

Tube: DTT_001

Population	#Events	%Parent	%Total
All Events	41,736	####	100.0
P	28,873	69.2	69.2
S1	28,583	99.0	68.5
S2	28,144	98.5	67.4
S3	26,451	94.0	63.4
S4	25,681	97.1	61.5
GFP+	25,536	99.4	61.2
Ox	31	0.1	0.1
Red	113	0.4	0.3

Experiment Name:	05EDec2016 Bac sorting
Specimen Name:	12Dec
Tube Name:	DTT_001
Record Date:	Dec 12, 2016 11:15:02 AM
$OP:	Administrator
GUID:	7ede832a-725f-417f-a947-3bd50a1...

Population	#Events	%Parent	FITC-A Median	BV510-A Median
S4	25,681	97.1	25,468	1,145
GFP+	25,536	99.4	25,600	1,150
Ox	31	0.1	11,865	2,080
Red	113	0.4	13,484	296

BD FACSDiva 8.0.1

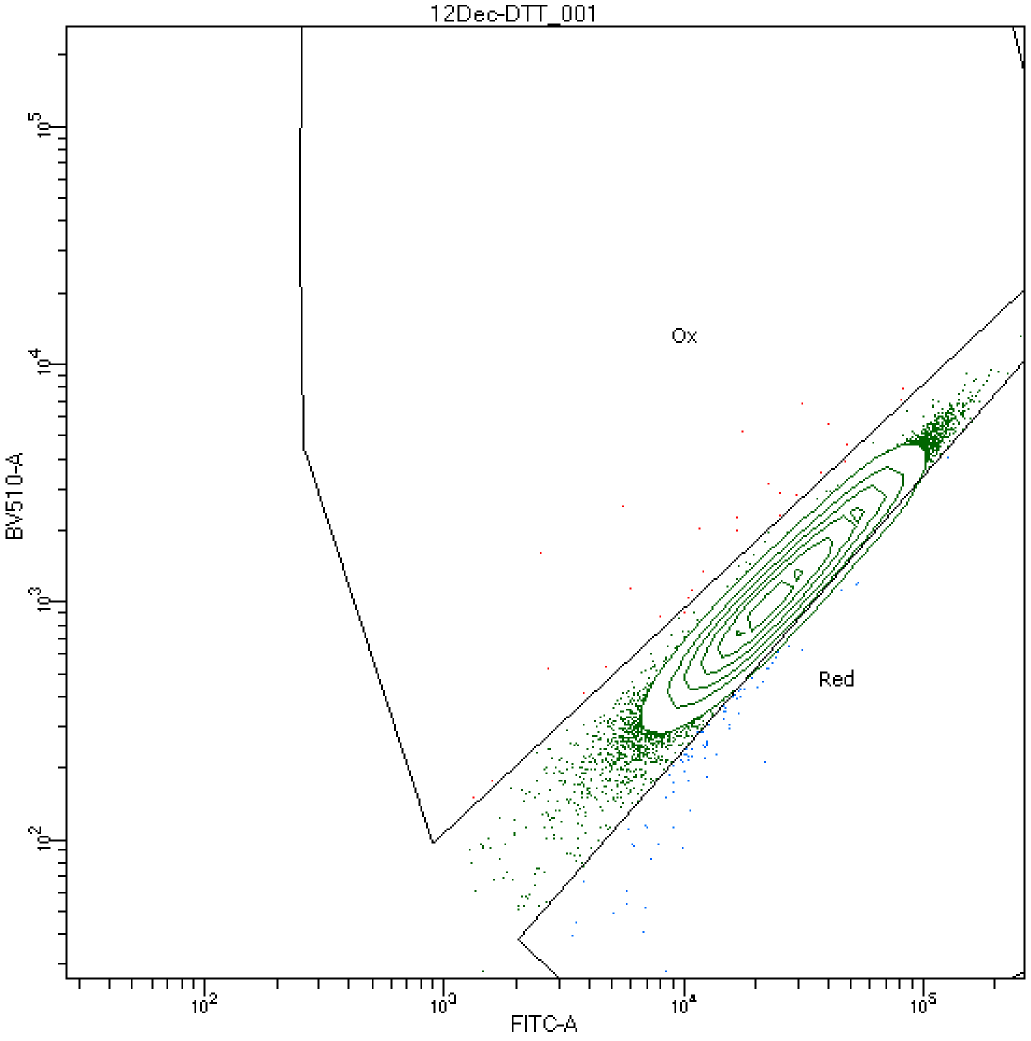

BD FACSDiva 8.0.1

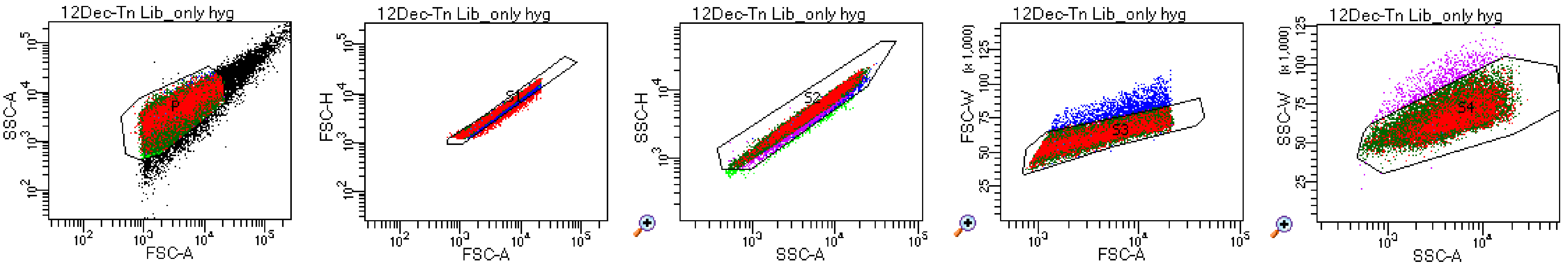

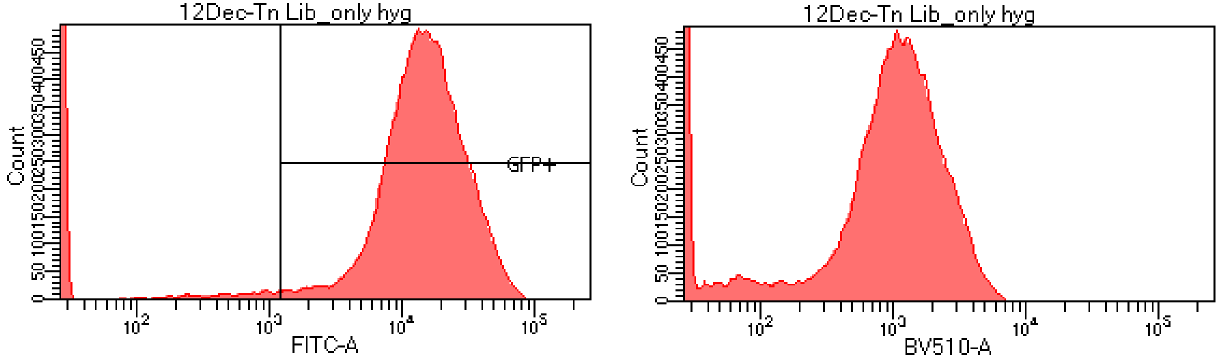

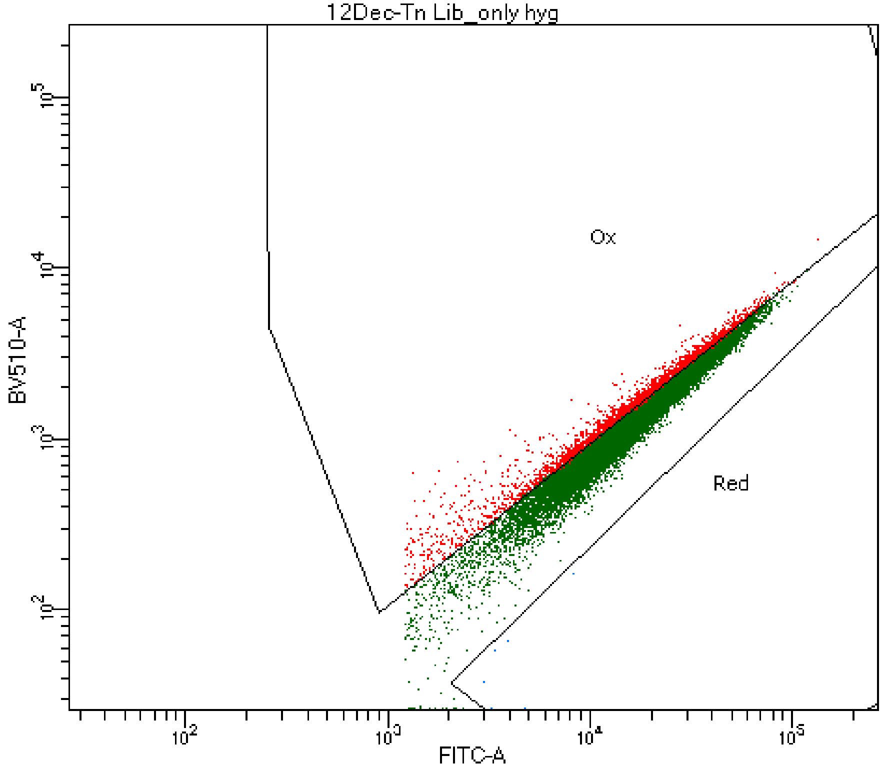

Tube: Tn Lib_only hyg

Population	#Events	%Parent	%Total
All Events	33,489	####	100.0
P	29,146	87.0	87.0
S1	28,568	98.0	85.3
S2	28,240	98.9	84.3
S3	26,691	94.5	79.7
S4	26,000	97.4	77.6
GFP+	22,227	85.5	66.4
Ox	2,663	12.0	8.0
Red	6	0.0	0.0

Experiment Name:	05EDec2016 Bac sorting
Specimen Name:	12Dec
Tube Name:	Tn Lib_only hyg
Record Date:	Dec 12, 2016 11:17:20 AM
$OP:	Administrator
GUID:	f533065a-8e46-4470-9fd7-f5eac910...

Population	#Events	%Parent	FITC-A Median	BV510-A Median
S4	26,000	97.4	12,657	974
GFP+	22,227	85.5	14,527	1,121
Ox	2,663	12.0	16,098	1,551
Red	6	0.0	3,656	48

BD FACSDiva 8.0.1

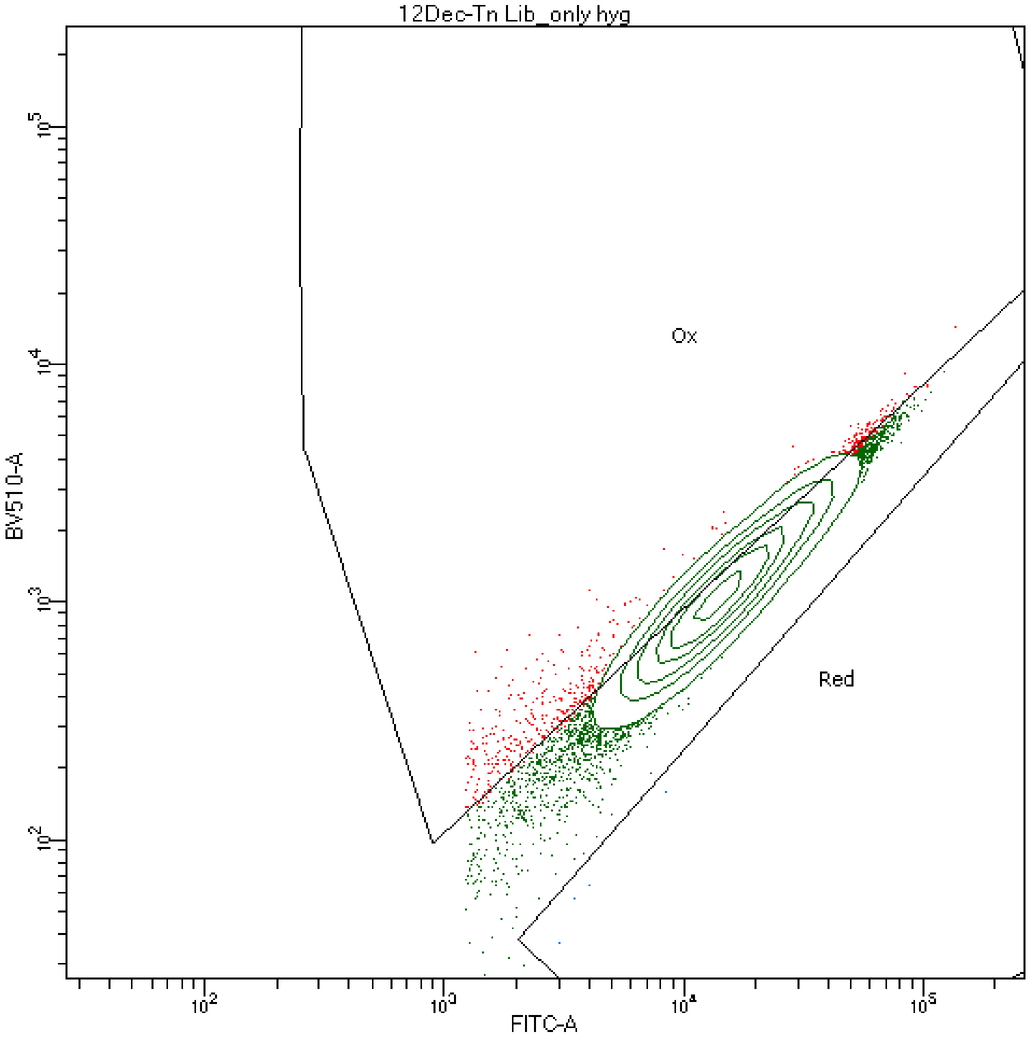

BD FACSDiva 8.0.1

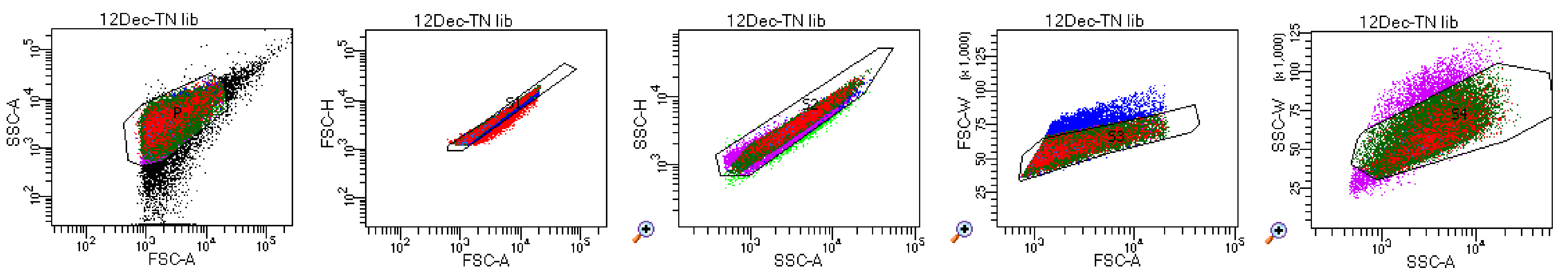

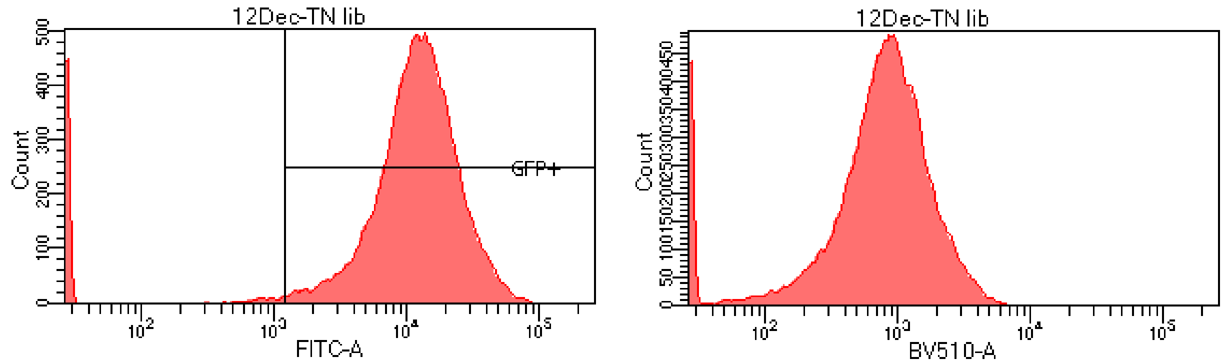

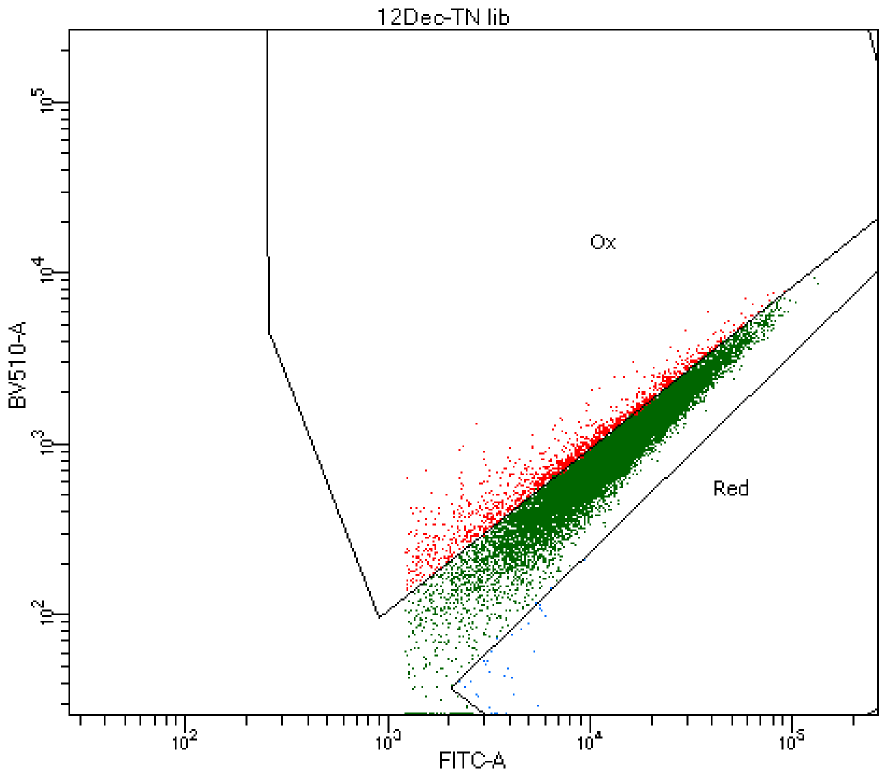

Tube: TN lib

Population	#Events	%Parent	%Total
All Events	34,514	####	100.0
P	30,678	88.9	88.9
S1	29,028	94.6	84.1
S2	28,414	97.9	82.3
S3	25,172	88.6	72.9
S4	22,899	91.0	66.3
GFP+	21,323	93.1	61.8
Ox	1,427	6.7	4.1
Red	34	0.2	0.1

Experiment Name: 05EDec2016 Bac sorting
Specimen Name: 12Dec
Tube Name: TN lib
Record Date: Dec 12, 2016 11:17:55 AM
$OP: Administrator
GUID: 61059bc0-924a-4566-b2a9-bb7c85...

Population	#Events	%Parent	FITC-A Median	BV510-A Median
S4	22,899	91.0	11,467	775
GFP+	21,323	93.1	12,131	822
Ox	1,427	6.7	7,391	800
Red	34	0.2	3,583	48

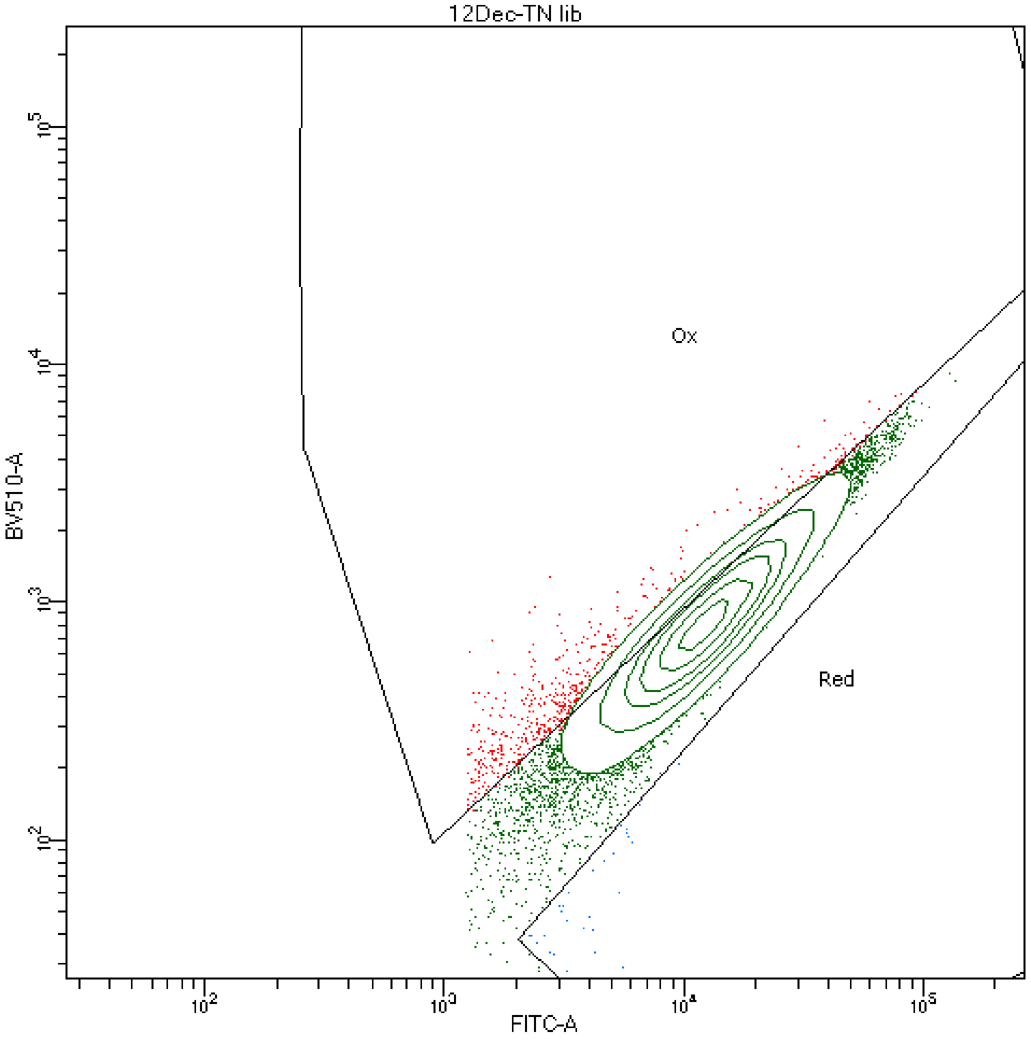

12Dec-TN lib
Ox
Red
BV510-A
FITC-A
10^2
10^3
10^4
10^5

BD FACSDiva 8.0.1

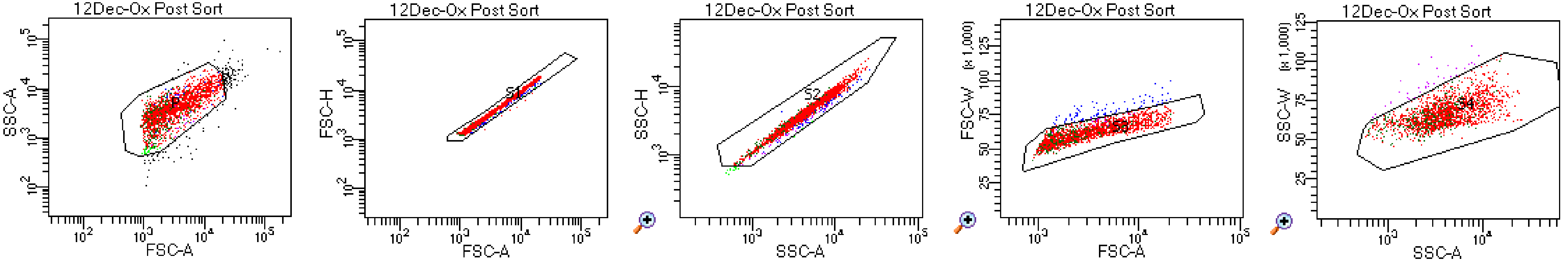

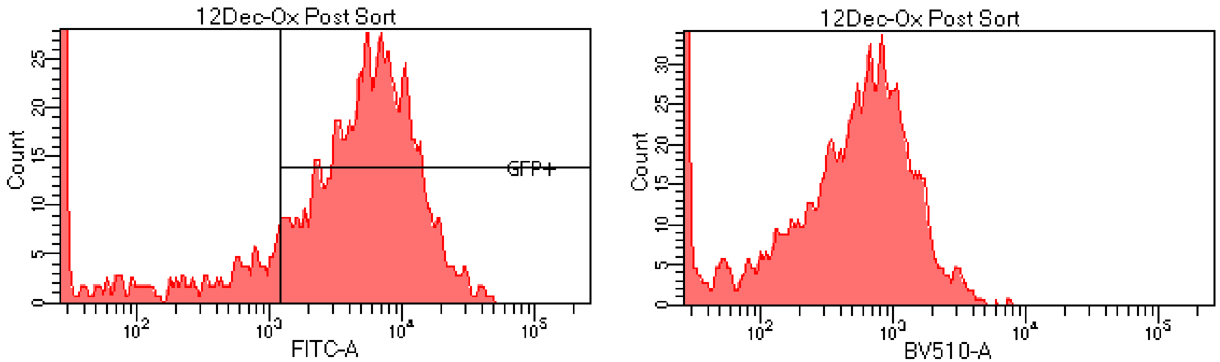

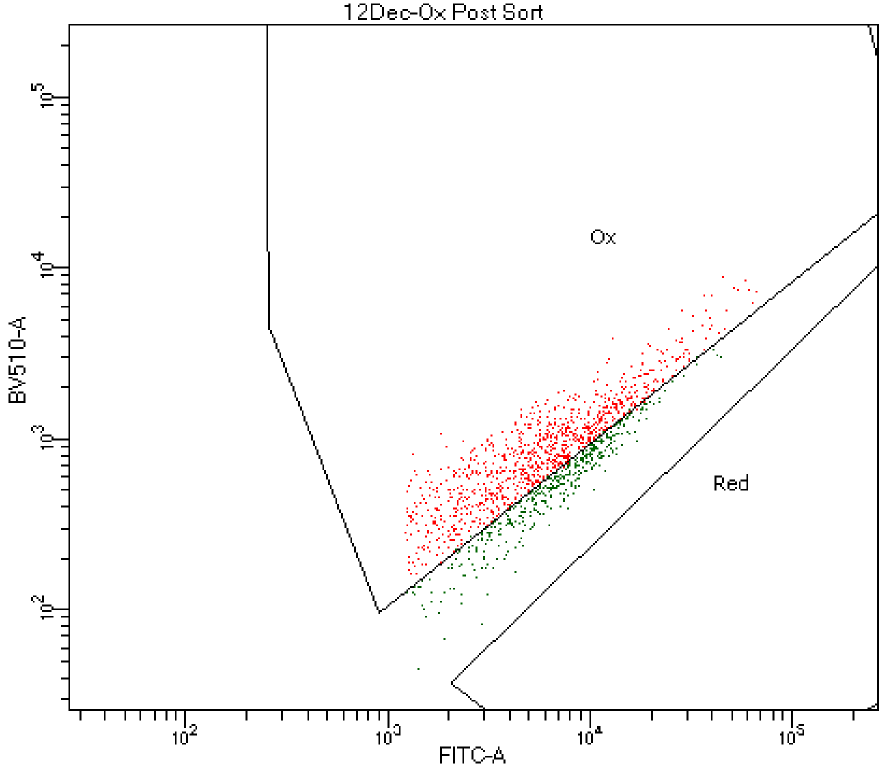

Tube: Ox Post Sort

Population	#Events	%Parent	%Total
All Events	2,226	####	100.0
P	2,078	93.4	93.4
S1	2,071	99.7	93.0
S2	2,042	98.6	91.7
S3	1,954	95.7	87.8
S4	1,919	98.2	86.2
GFP+	1,403	73.1	63.0
Ox	954	68.0	42.9
Red	0	0.0	0.0

Experiment Name: 05EDec2016 Bac sorting
Specimen Name: 12Dec
Tube Name: Ox Post Sort
Record Date: Dec 12, 2016 3:09:48 PM
$OP: Administrator
GUID: 320e8542-f8f2-4ec4-ab6d-f1468a19...

Population	#Events	%Parent	FITC-A Median	BV510-A Median
S4	1,919	98.2	4,056	523
GFP+	1,403	73.1	6,027	727
Ox	954	68.0	5,492	821
Red	0	0.0	####	####

BD FACSDiva 8.0.1

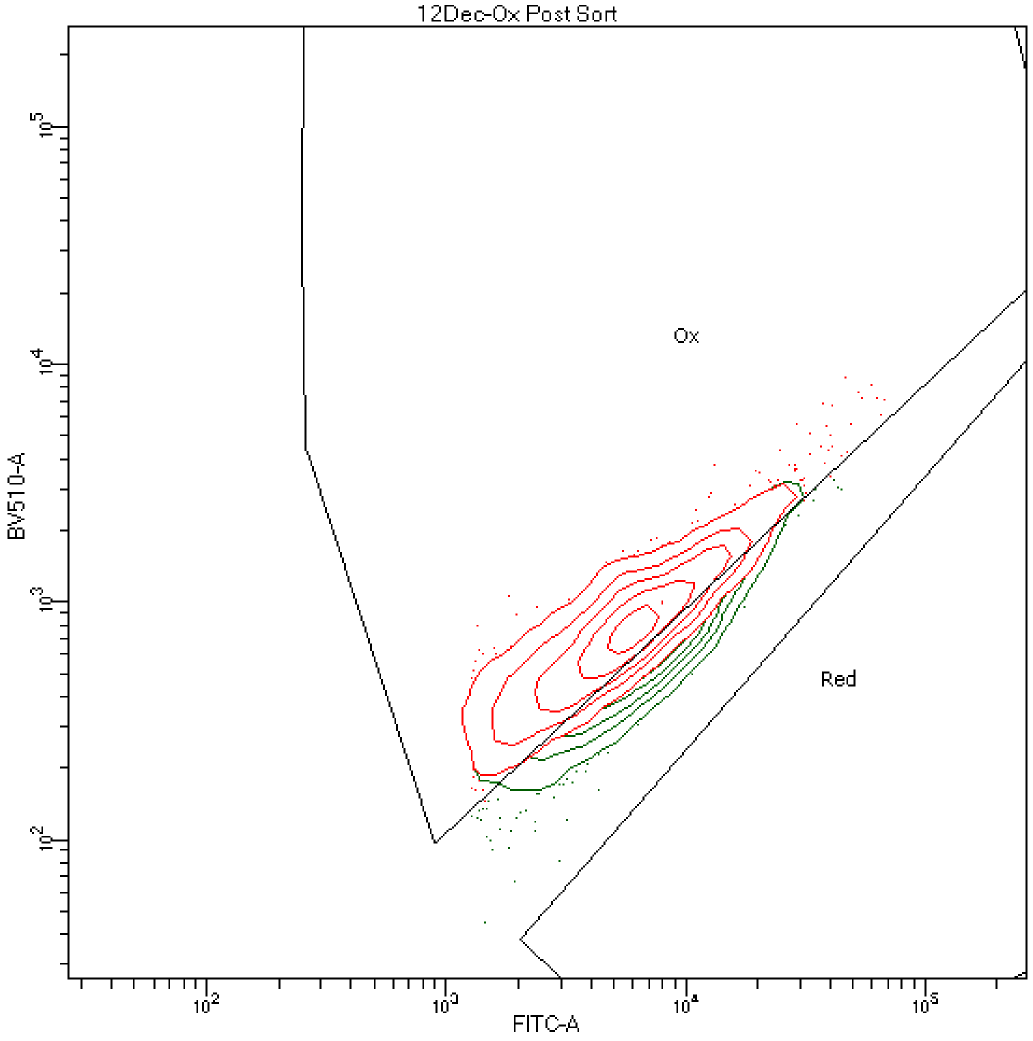

BD FACSDiva 8.0.1

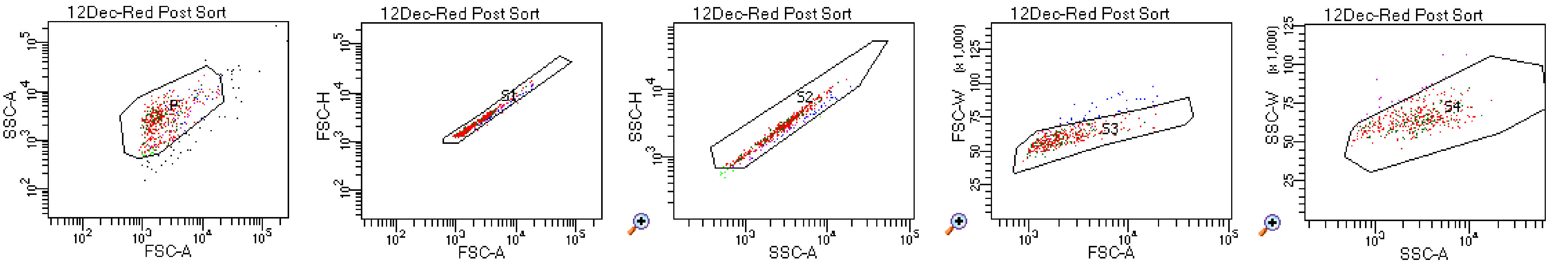

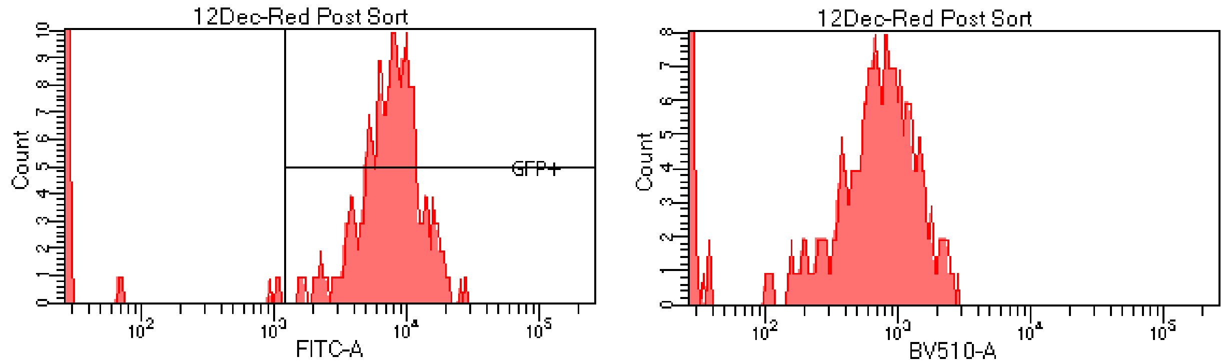

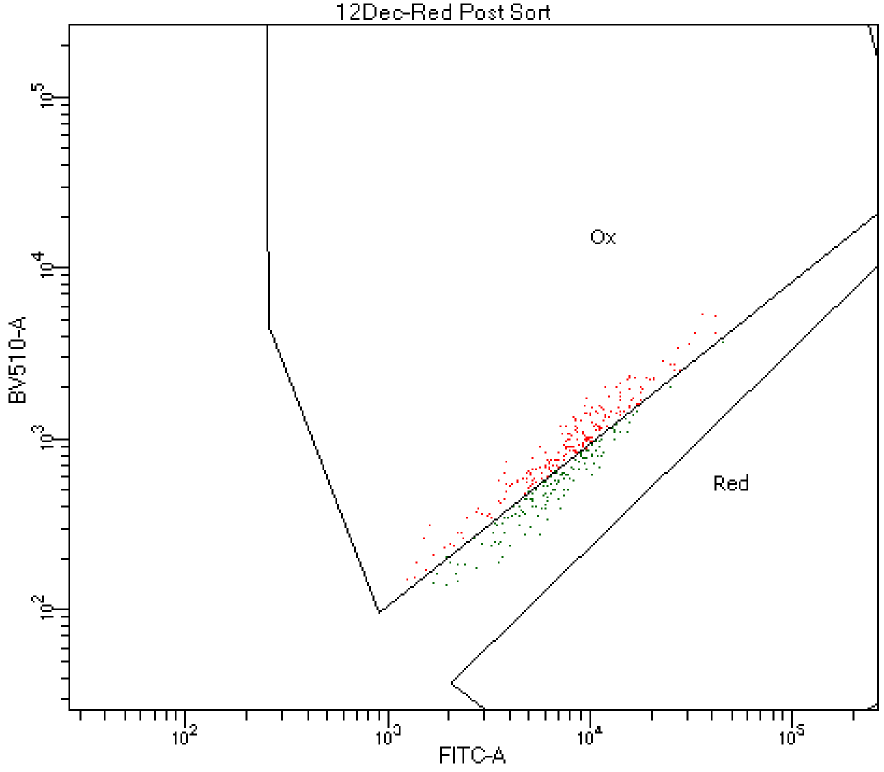

Tube: Red Post Sort

Population	#Events	%Parent	%Total
All Events	531	####	100.0
P	480	90.4	90.4
S1	474	98.8	89.3
S2	461	97.3	86.8
S3	431	93.5	81.2
S4	413	95.8	77.8
GFP+	343	83.1	64.6
Ox	206	60.1	38.8
Red	0	0.0	0.0

Experiment Name: 05EDec2016 Bac sorting
Specimen Name: 12Dec
Tube Name: Red Post Sort
Record Date: Dec 12, 2016 3:10:29 PM
$OP: Administrator
GUID: 25a9223e-6a10-48cd-87aa-294fff6a...

Population	#Events	%Parent	FITC-A Median	BV510-A Median
S4	413	95.8	6,589	632
GFP+	343	83.1	7,594	758
Ox	206	60.1	8,263	973
Red	0	0.0	####	####

BD FACSDiva 8.0.1

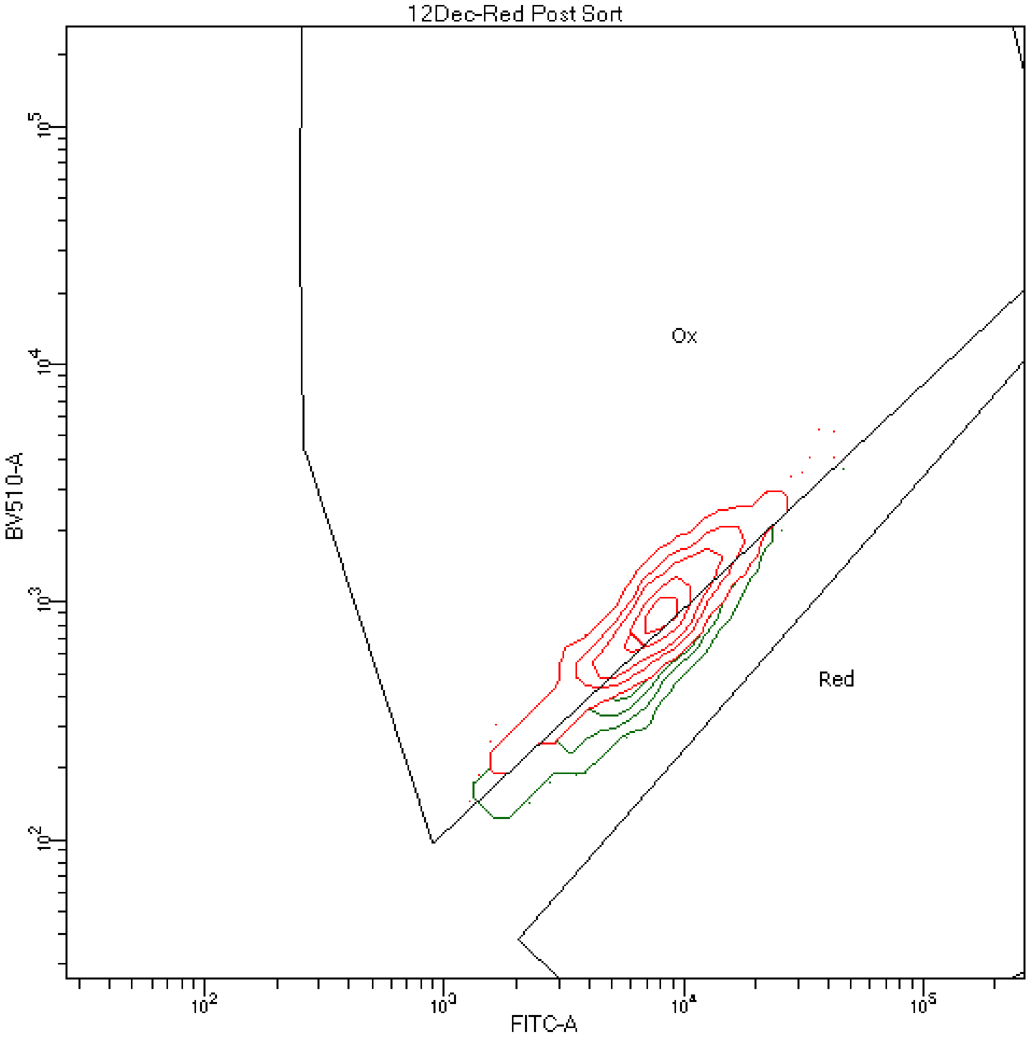

Supplement: Figure 1—source data 1. [file elife-80218-fig1-data1.zip › Round 2 Sorting/12Dec2016 Bac sorting-Batch_Analysis.pdf]
