# Supplementary material for: Biosensor-integrated transposon mutagenesis reveals rv0158 as a coordinator of redox homeostasis in Mycobacterium tuberculosis
Source: eLife. 2023 Aug 29;12:e80218. doi: 10.7554/eLife.80218 (PMC10501769; doi:10.7554/eLife.80218)

Batch Analysis Report

Run Date: 12/6/16 4:26 PM Experiment: 05EDec2016 Bac sorting
User ID: Administrator
Statistics Output: N/A
Worksheet PDF Output: C:\Users\Admin\Desktop\06EDec2016 Bac sorting-Batch_Analysis_0612 2016162608.pdf

06Dec

Tube	Status	Run Time
US	OK	12/6/16 4:26 PM
RV Mrx1	OK	12/6/16 4:26 PM
RV Mrx1_001	OK	12/6/16 4:26 PM
RV Mrx1_002	OK	12/6/16 4:26 PM
TN lib	OK	12/6/16 4:26 PM
Ox Post Sort	OK	12/6/16 4:26 PM
Red Post Sort	OK	12/6/16 4:26 PM

BD FACSDiva 8.0.1

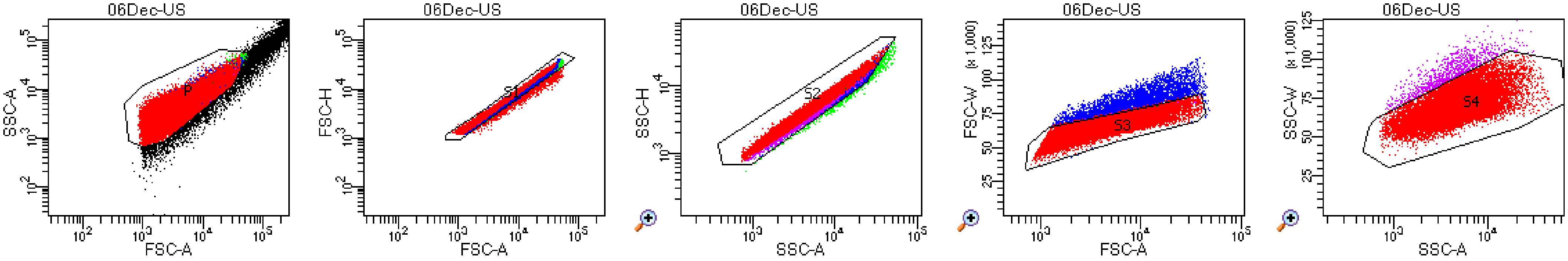

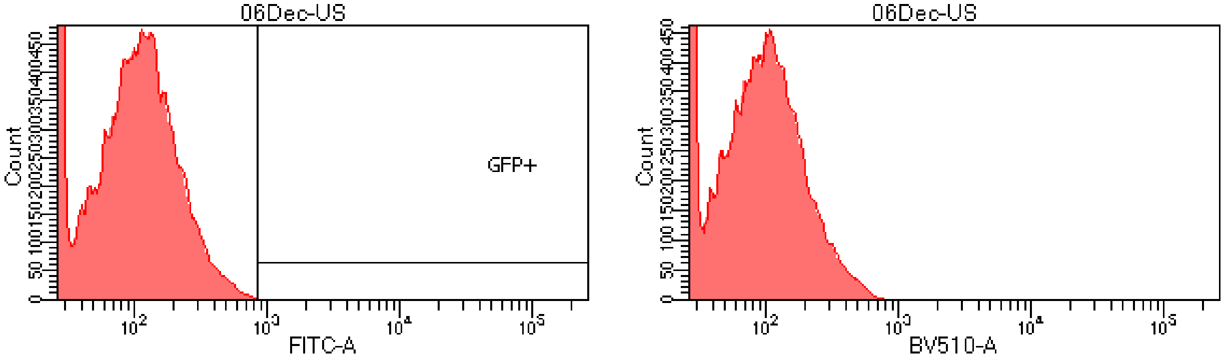

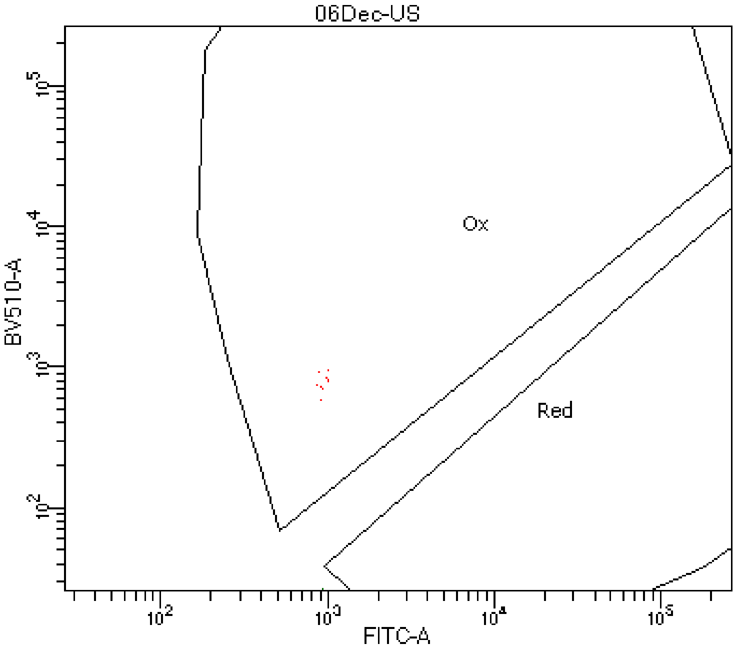

Tube: US

Population	#Events	%Parent	%Total
All Events	35,192	####	100.0
P	30,000	85.2	85.2
S1	28,627	95.4	81.3
S2	27,978	97.7	79.5
S3	23,944	85.6	68.0
S4	23,023	96.2	65.4
GFP+	10	0.0	0.0
Ox	9	90.0	0.0
Red	0	0.0	0.0

Experiment Name:	05EDec2016 Bac sorting
Specimen Name:	06Dec
Tube Name:	US
Record Date:	Dec 6, 2016 2:12:29 PM
$OP:	Administrator
GUID:	bab90d99-ffe7-4c0d-9fda-2221...

Population	#Events	%Parent	FITC-A Median	BV510-A Median
S4	23,023	96.2	97	83
GFP+	10	0.0	917	759
Ox	9	90.0	924	784
Red	0	0.0	####	####

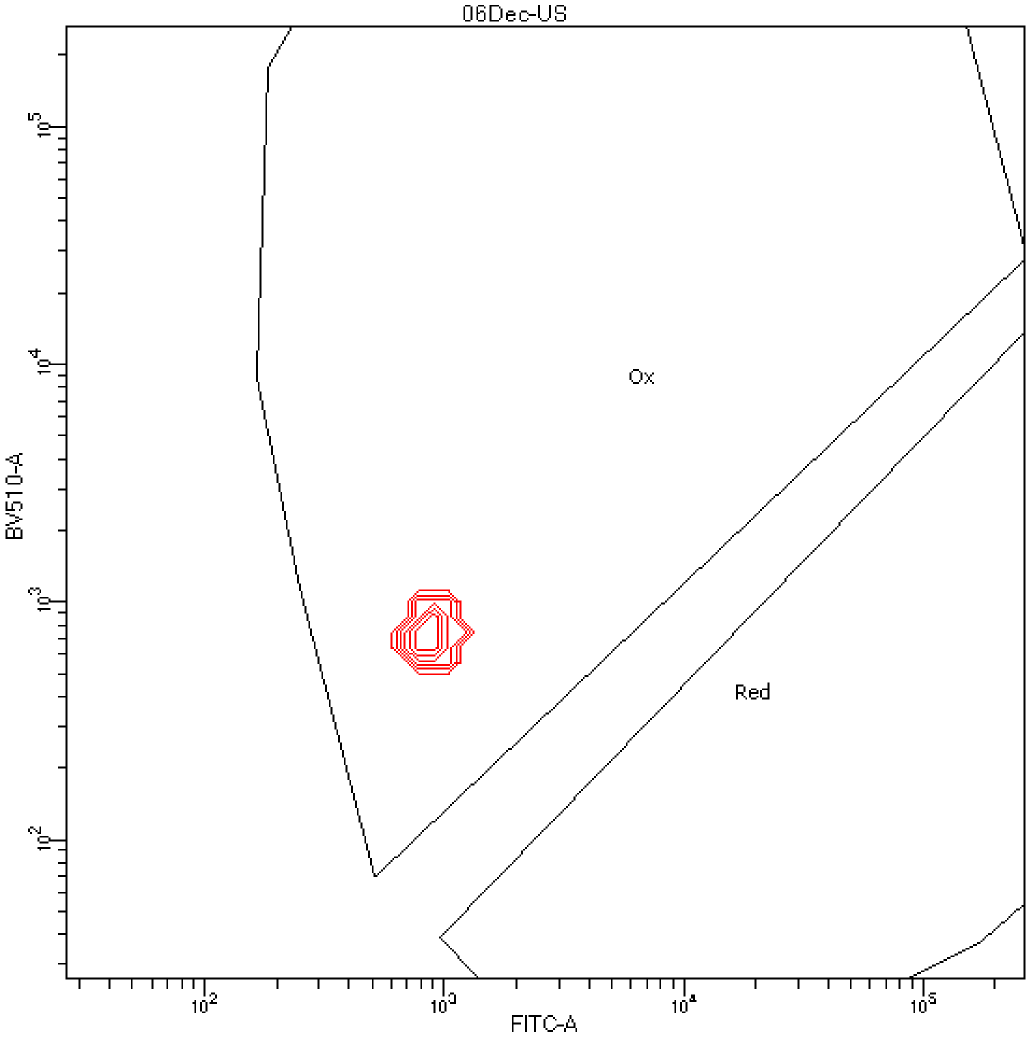
06Dec-US
Ox
Red
BV510-A
FITC-A
10^2
10^3
10^4
10^5

BD FACSDiva 8.0.1

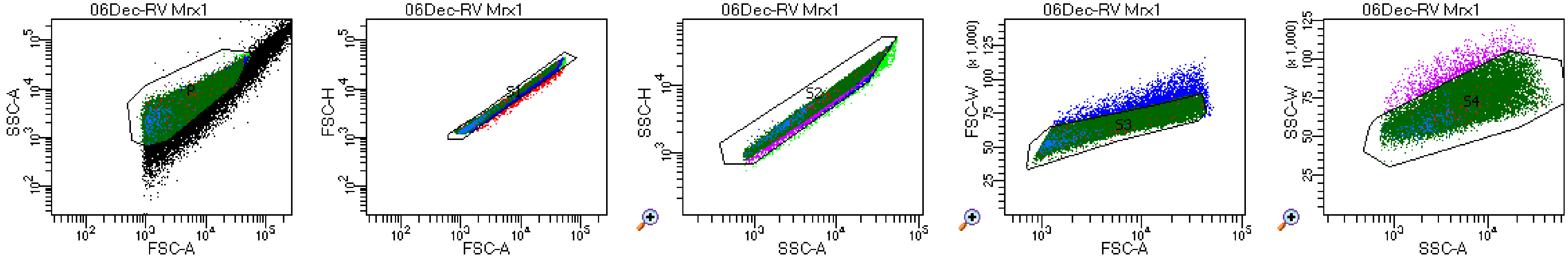

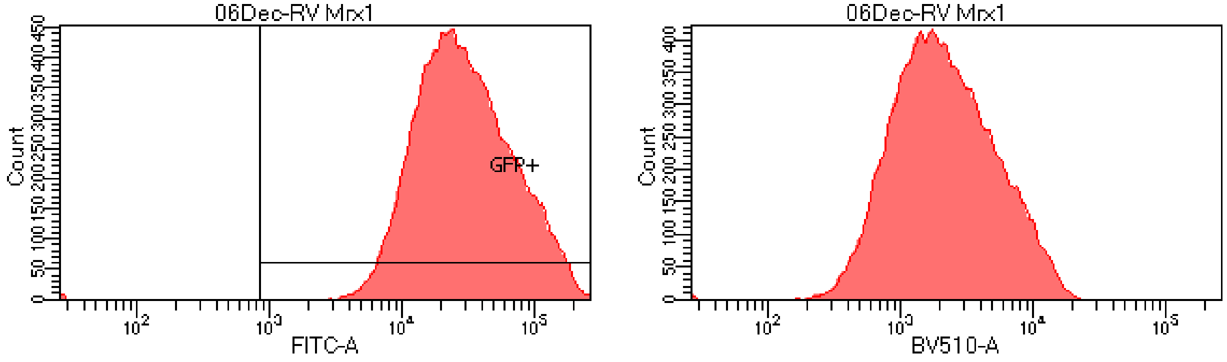

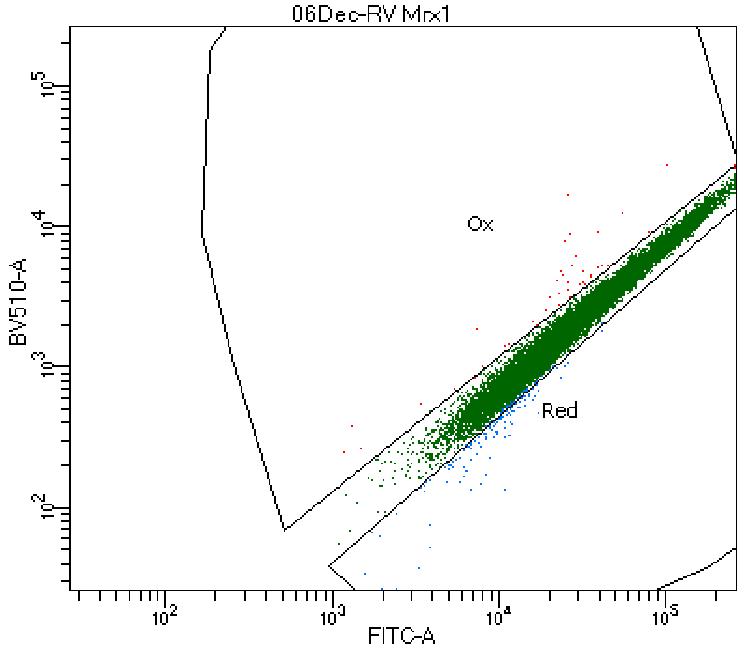

Tube: RV Mrx1

Population	#Events	%Parent	%Total
All Events	38,323	####	100.0
P	30,000	78.3	78.3
S1	29,618	98.7	77.3
S2	29,115	98.3	76.0
S3	26,784	92.0	69.9
S4	25,837	96.5	67.4
GFP+	25,793	99.8	67.3
Ox	49	0.2	0.1
Red	175	0.7	0.5

Experiment Name: 05EDec2016 Bac sorting
Specimen Name: 06Dec
Tube Name: RV Mrx1
Record Date: Dec 6, 2016 2:13:21 PM
$OP: Administrator
GUID: e18fffd3-0e6f-4822-8d3e-8089d...

Population	#Events	%Parent	FITC-A Median	BV510-A Median
S4	25,837	96.5	27,036	1,935
GFP+	25,793	99.8	27,077	1,938
Ox	49	0.2	24,624	3,952
Red	175	0.7	10,702	432

BD FACSDiva 8.0.1

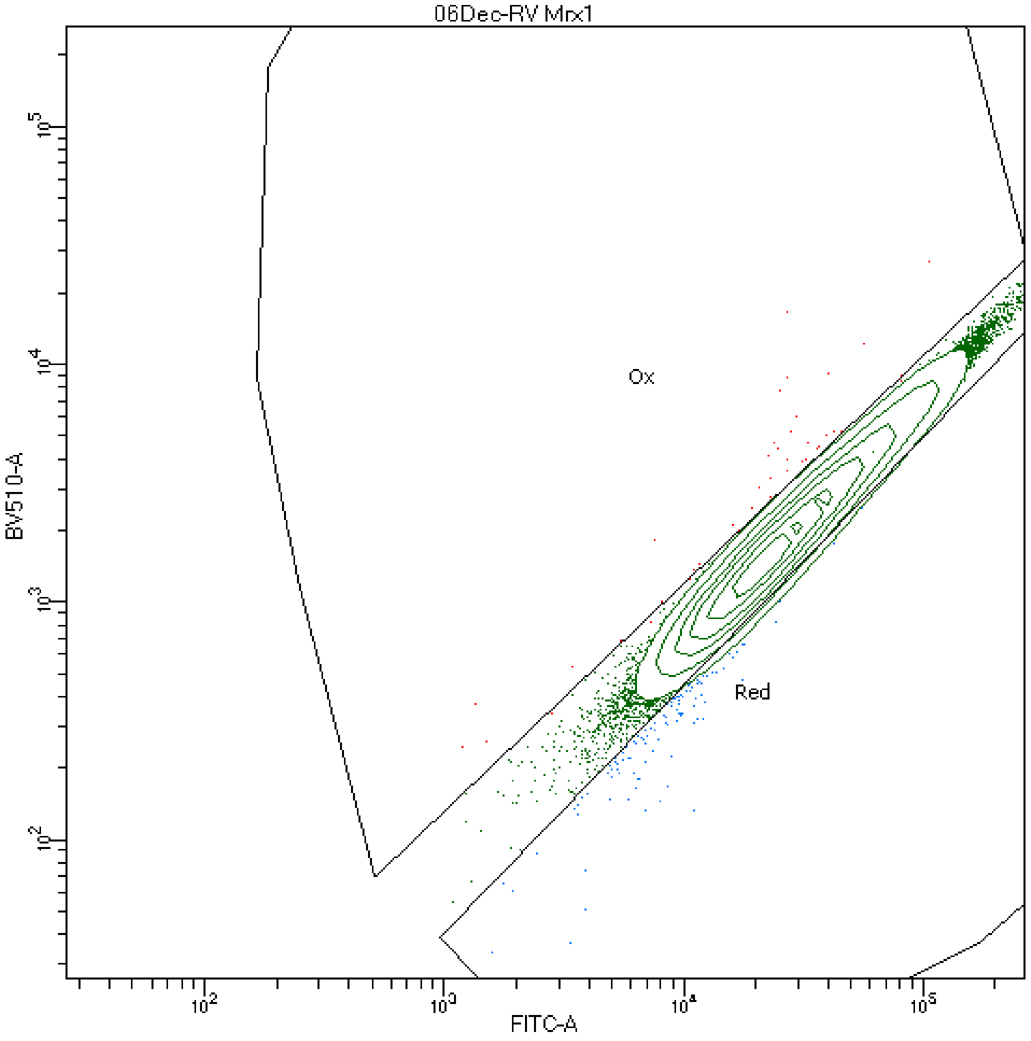

BD FACSDiva 8.0.1

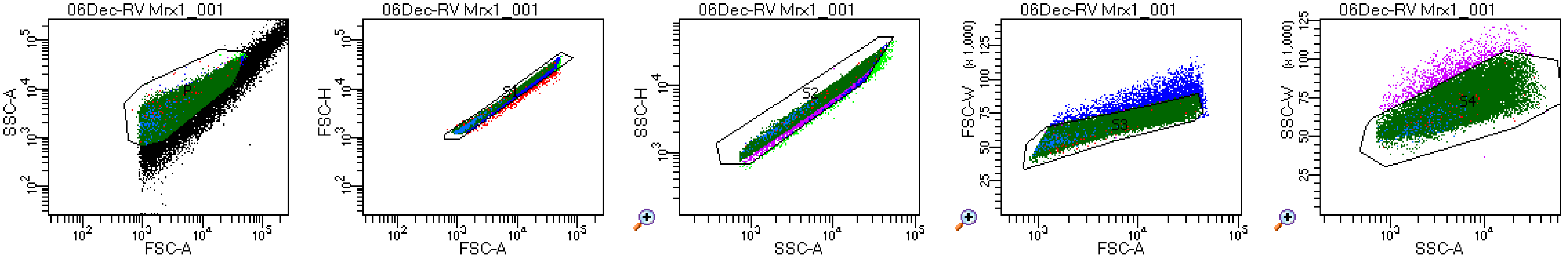

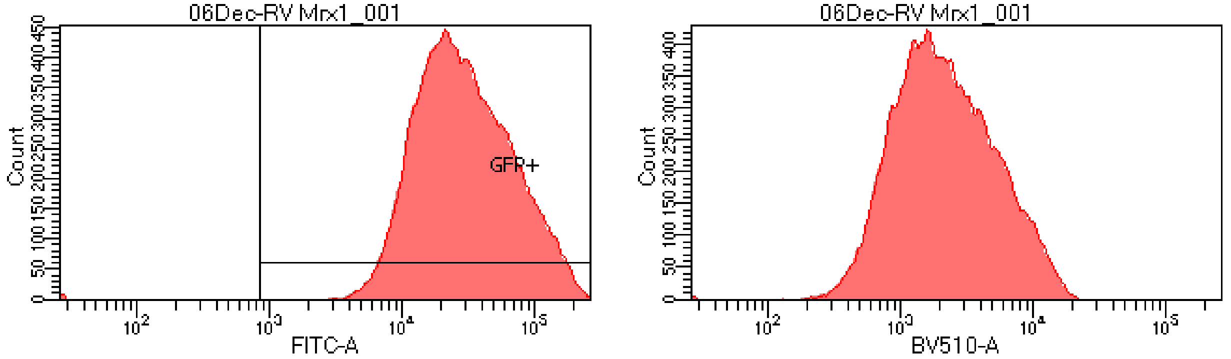

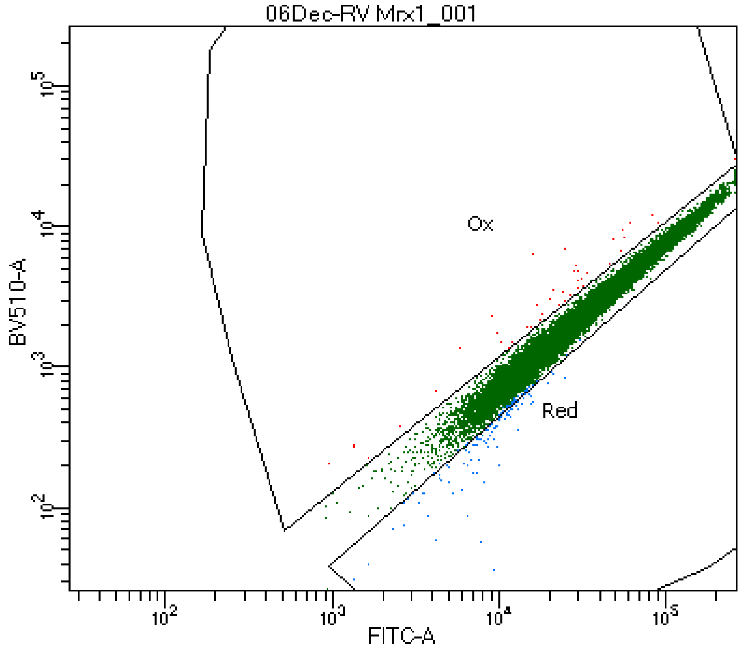

Tube: RV Mrx1_001

Population	#Events	%Parent	%Total
All Events	38,403	####	100.0
P	30,000	78.1	78.1
S1	29,593	98.6	77.1
S2	29,008	98.0	75.5
S3	26,517	91.4	69.0
S4	25,567	96.4	66.6
GFP+	25,526	99.8	66.5
Ox	47	0.2	0.1
Red	154	0.6	0.4

Experiment Name:	05EDec2016 Bac sorting
Specimen Name:	06Dec
Tube Name:	RV Mrx1_001
Record Date:	Dec 6, 2016 2:13:56 PM
$OP:	Administrator
GUID:	36805fdd-16e1-42e4-8f22-3aac...

Population	#Events	%Parent	FITC-A Median	BV510-A Median
S4	25,567	96.4	26,760	1,945
GFP+	25,526	99.8	26,840	1,949
Ox	47	0.2	24,579	3,516
Red	154	0.6	9,735	388

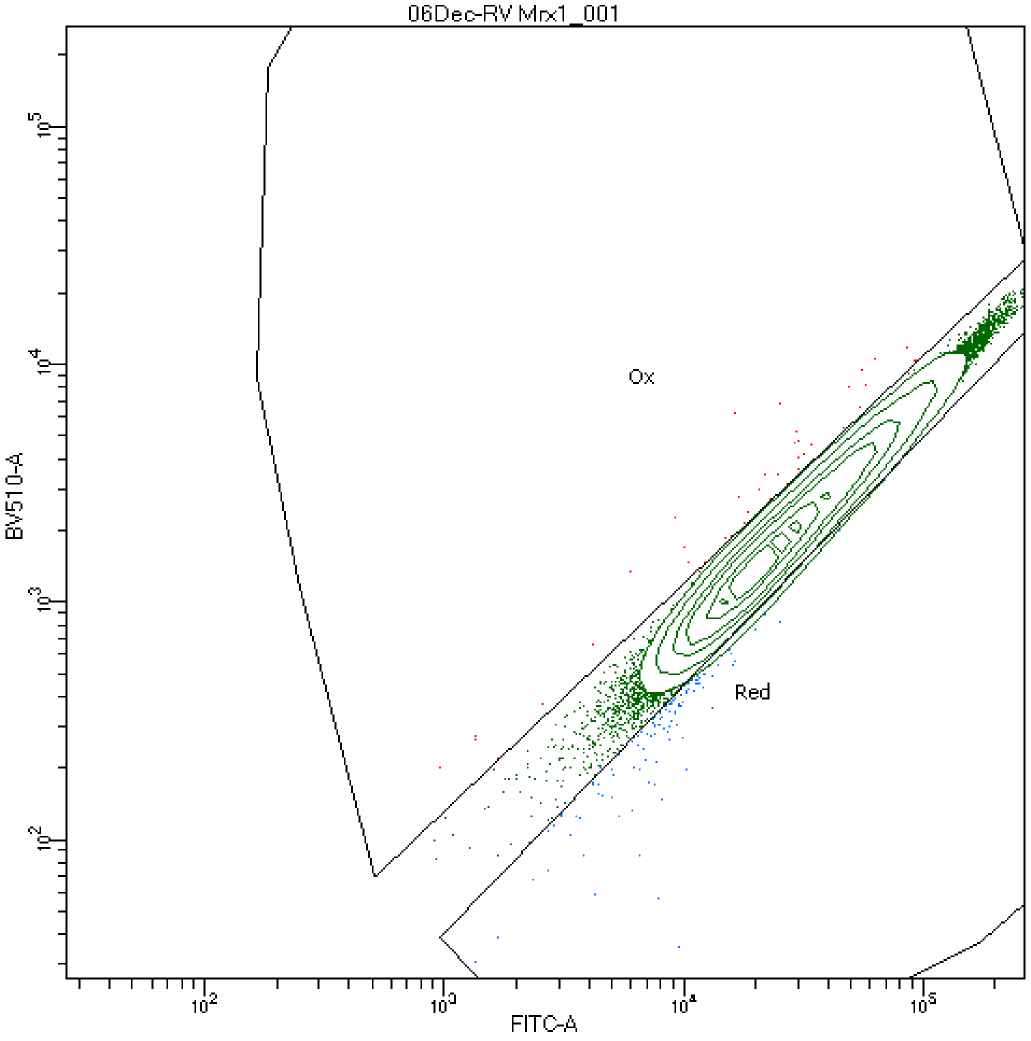

06Dec-RV Mrx1_001
Ox
Red
BV510-A
FITC-A

BD FACSDiva 8.0.1

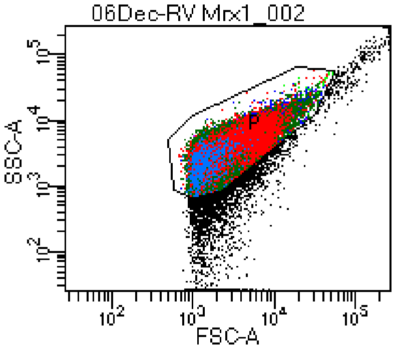

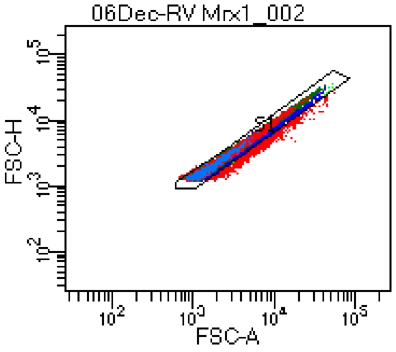

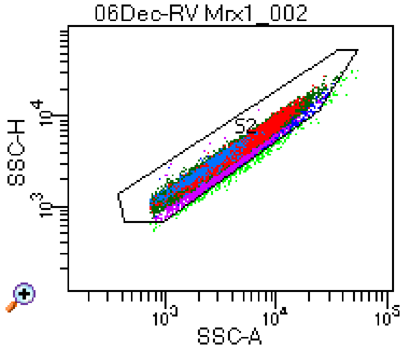

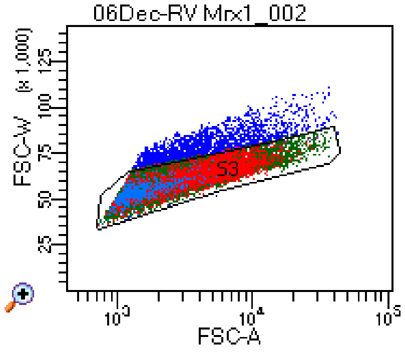

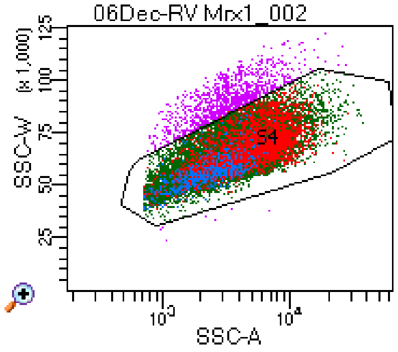

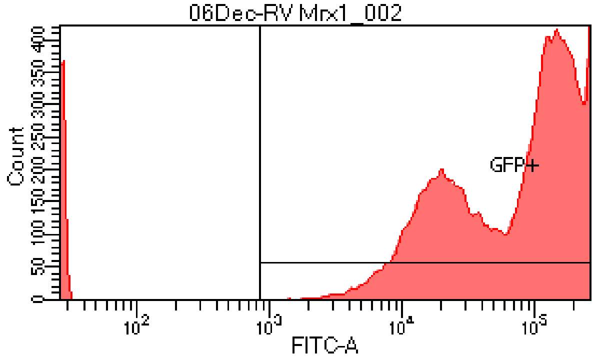

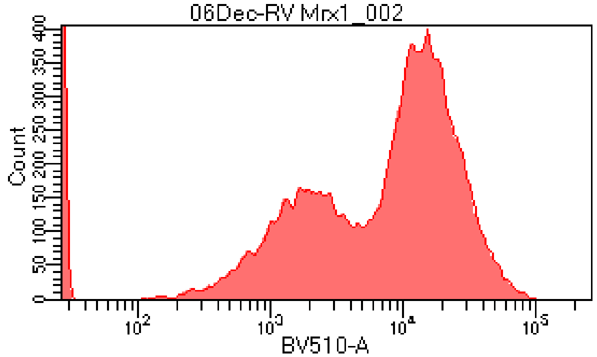

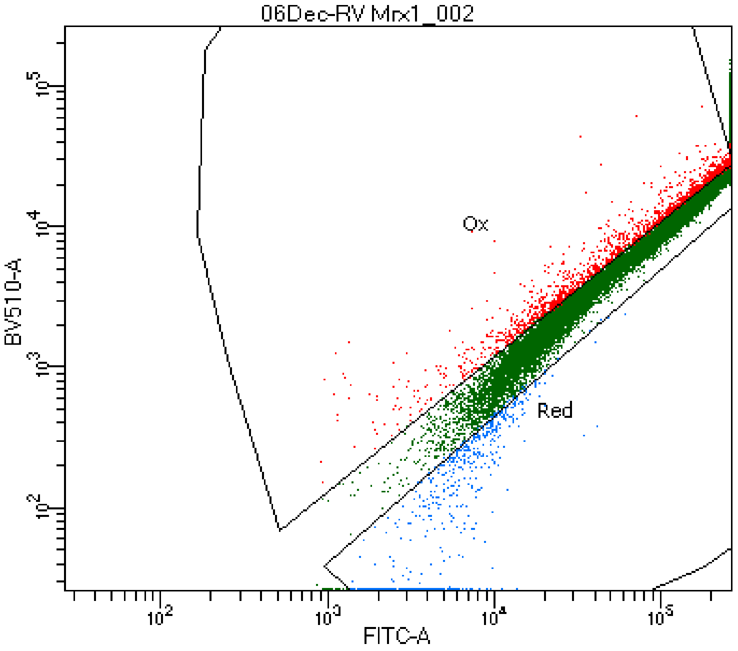

Tube: RV Mrx1_002

Population	#Events	%Parent	%Total
All Events	33,015	####	100.0
P	30,000	90.9	90.9
S1	28,921	96.4	87.6
S2	28,507	98.6	86.3
S3	26,447	92.8	80.1
S4	25,230	95.4	76.4
GFP+	24,255	96.1	73.5
Ox	3,288	13.6	10.0
Red	579	2.4	1.8

Experiment Name:	05EDec2016 Bac sorting
Specimen Name:	06Dec
Tube Name:	RV Mrx1_002
Record Date:	Dec 6, 2016 2:14:28 PM
$OP:	Administrator
GUID:	2aac91fc-8fc5-420a-a2eb-e499...

Population	#Events	%Parent	FITC-A Median	BV510-A Median
S4	25,230	95.4	98,597	9,038
GFP+	24,255	96.1	104,496	9,599
Ox	3,288	13.6	245,682	26,778
Red	579	2.4	6,204	178

BD FACSDiva 8.0.1

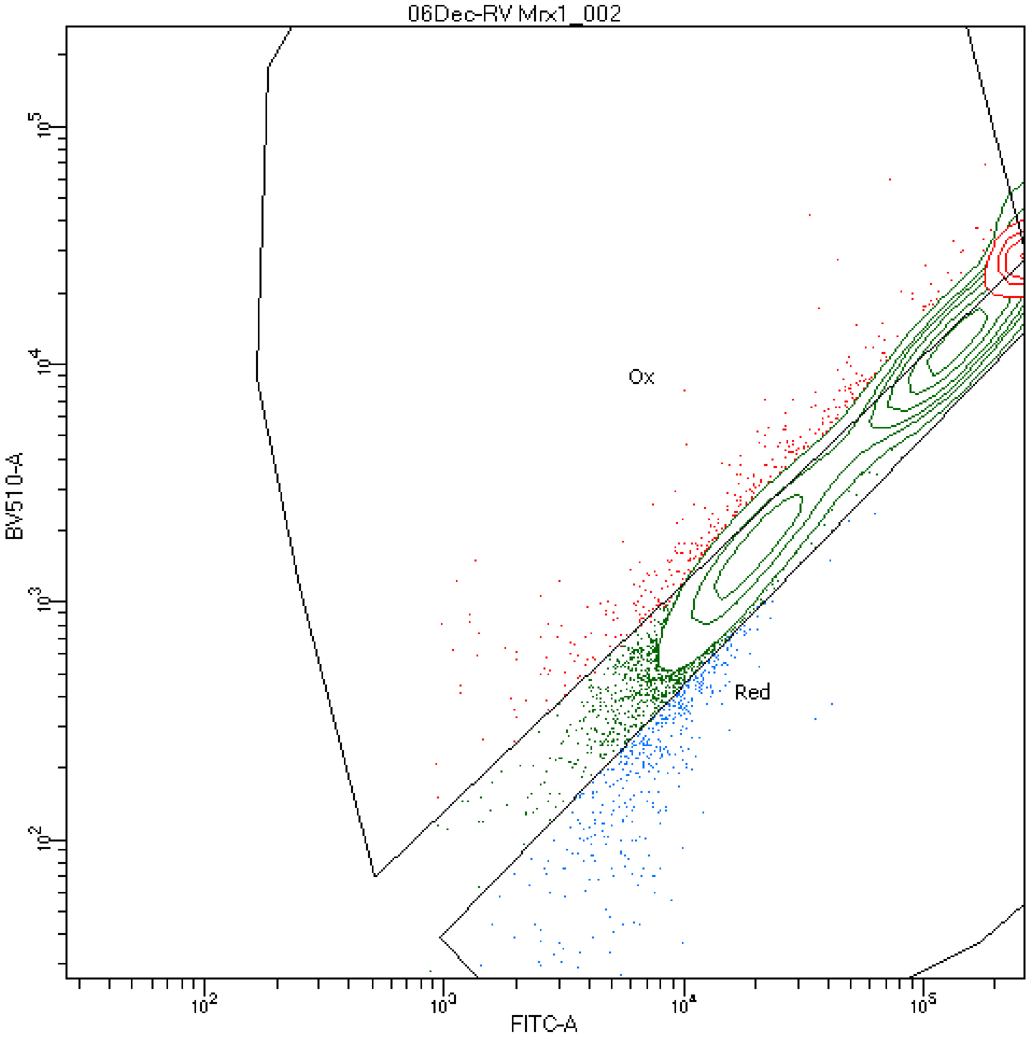

BD FACSDiva 8.0.1

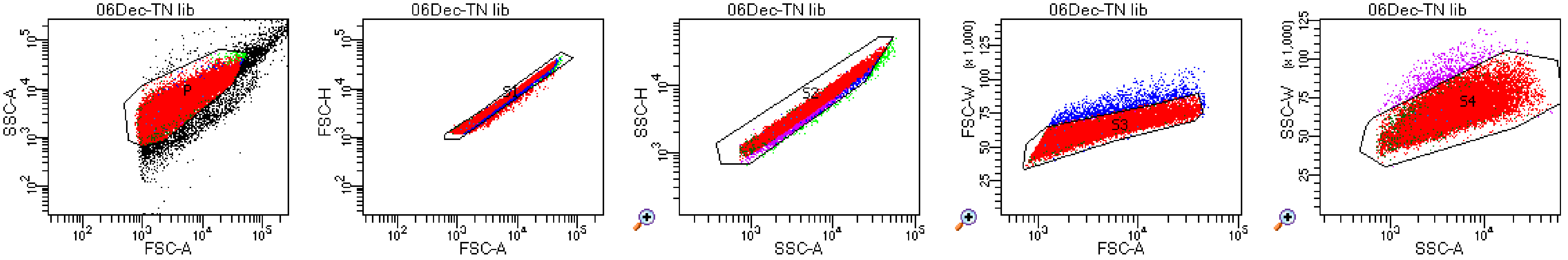

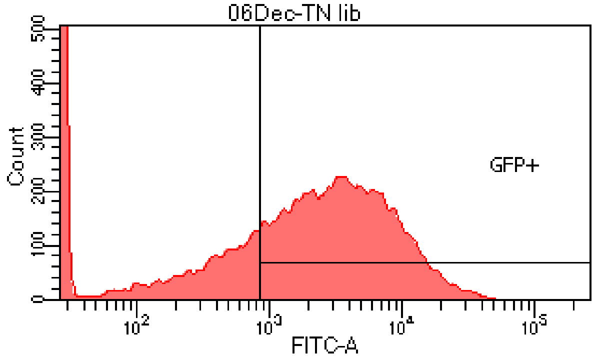

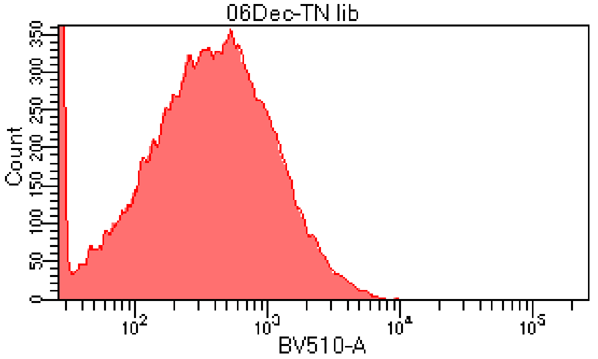

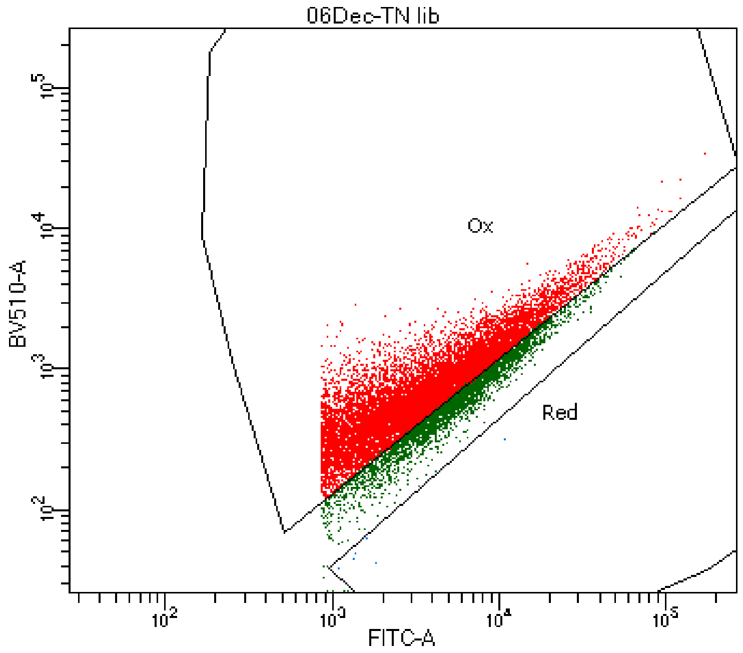

Tube: TN lib

Population	#Events	%Parent	%Total
All Events	32,644	####	100.0
P	30,000	91.9	91.9
S1	29,618	98.7	90.7
S2	29,317	99.0	89.8
S3	27,964	95.4	85.7
S4	27,239	97.4	83.4
GFP+	15,353	56.4	47.0
Ox	11,664	76.0	35.7
Red	8	0.1	0.0

Experiment Name: 05EDec2016 Bac sorting
Specimen Name: 06Dec
Tube Name: TN lib
Record Date: Dec 6, 2016 2:15:39 PM
$OP: Administrator
GUID: bfe7caeb-0f50-4da1-b0d5-c71e...

Population	#Events	%Parent	FITC-A Median	BV510-A Median
S4	27,239	97.4	1,262	344
GFP+	15,353	56.4	3,656	632
Ox	11,664	76.0	3,129	652
Red	8	0.1	1,693	55

BD FACSDiva 8.0.1

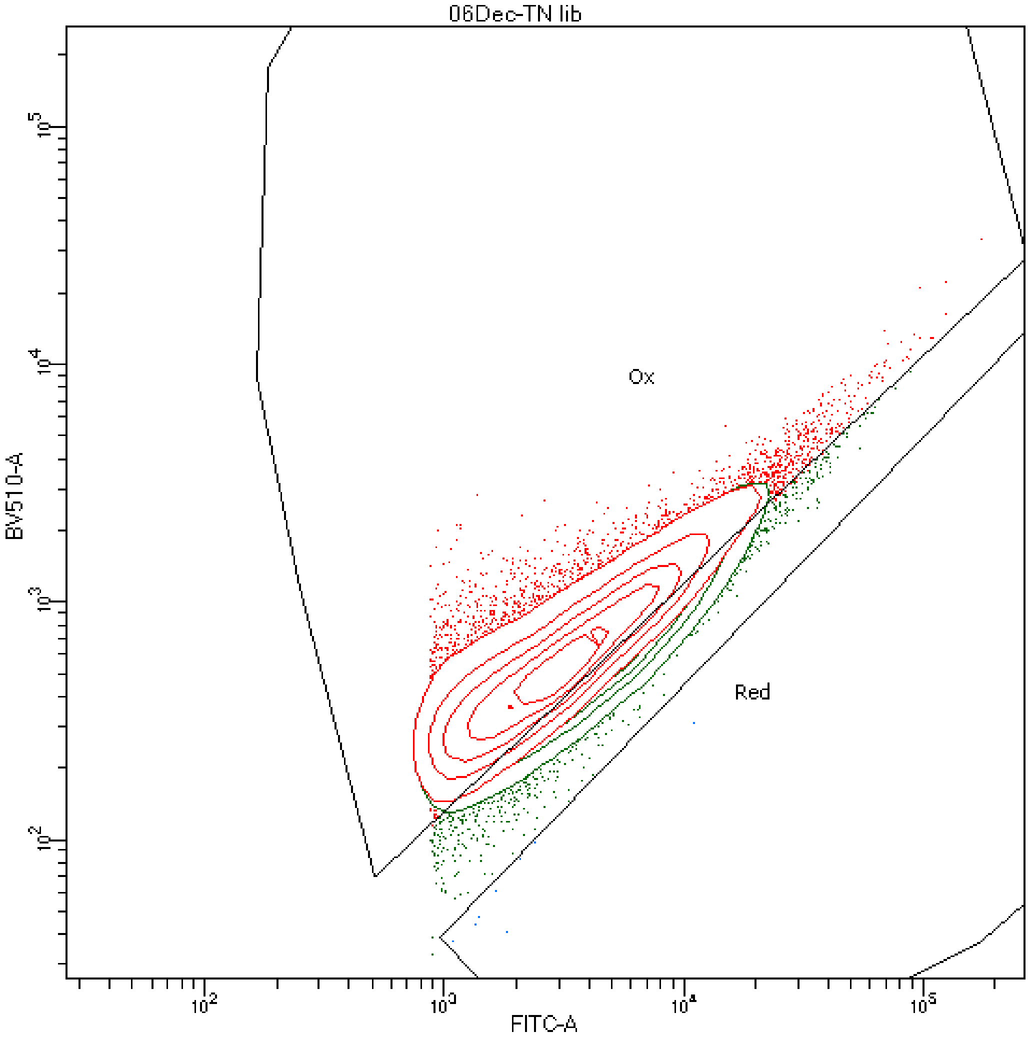

BD FACSDiva 8.0.1

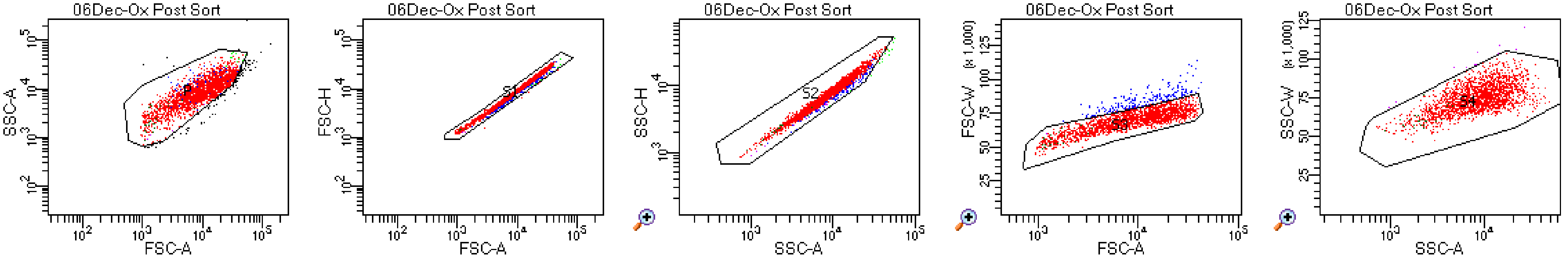

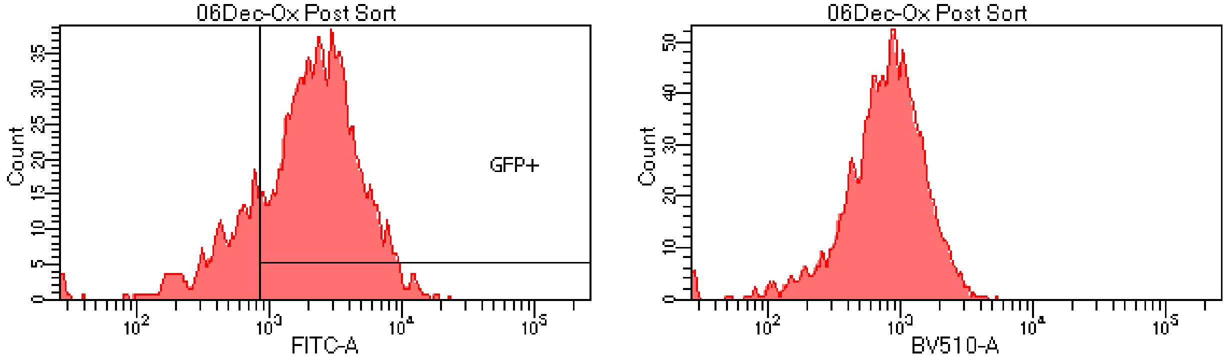

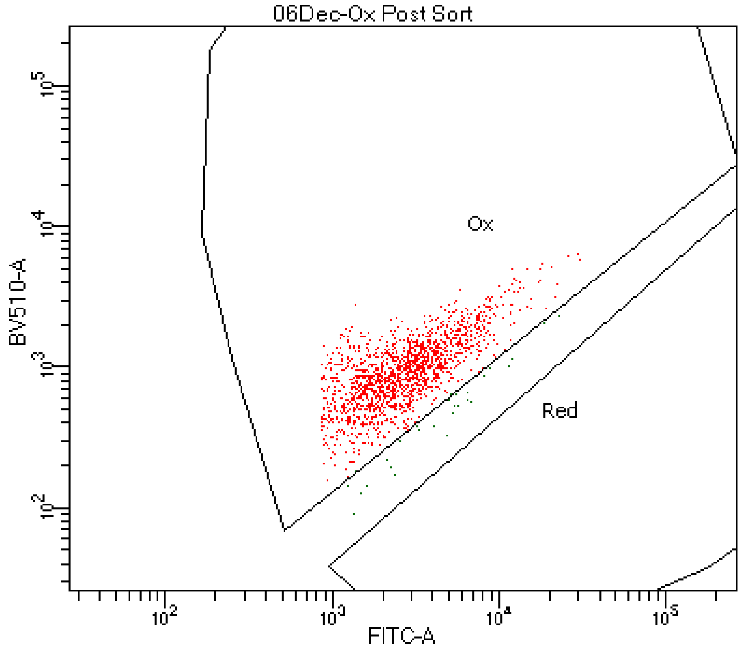

Tube: Ox Post Sort

Population	#Events	%Parent	%Total
All Events	2,480	####	100.0
P	2,342	94.4	94.4
S1	2,333	99.6	94.1
S2	2,315	99.2	93.3
S3	2,098	90.6	84.6
S4	2,084	99.3	84.0
GFP+	1,623	77.9	65.4
Ox	1,590	98.0	64.1
Red	0	0.0	0.0

Experiment Name: 05EDec2016 Bac sorting
Specimen Name: 06Dec
Tube Name: Ox Post Sort
Record Date: Dec 6, 2016 4:23:30 PM
$OP: Administrator
GUID: 4cbcdd5b-3037-45e0-933c-910...

Population	#Events	%Parent	FITC-A Median	BV510-A Median
S4	2,084	99.3	1,986	781
GFP+	1,623	77.9	2,518	894
Ox	1,590	98.0	2,494	901
Red	0	0.0	####	####

BD FACSDiva 8.0.1

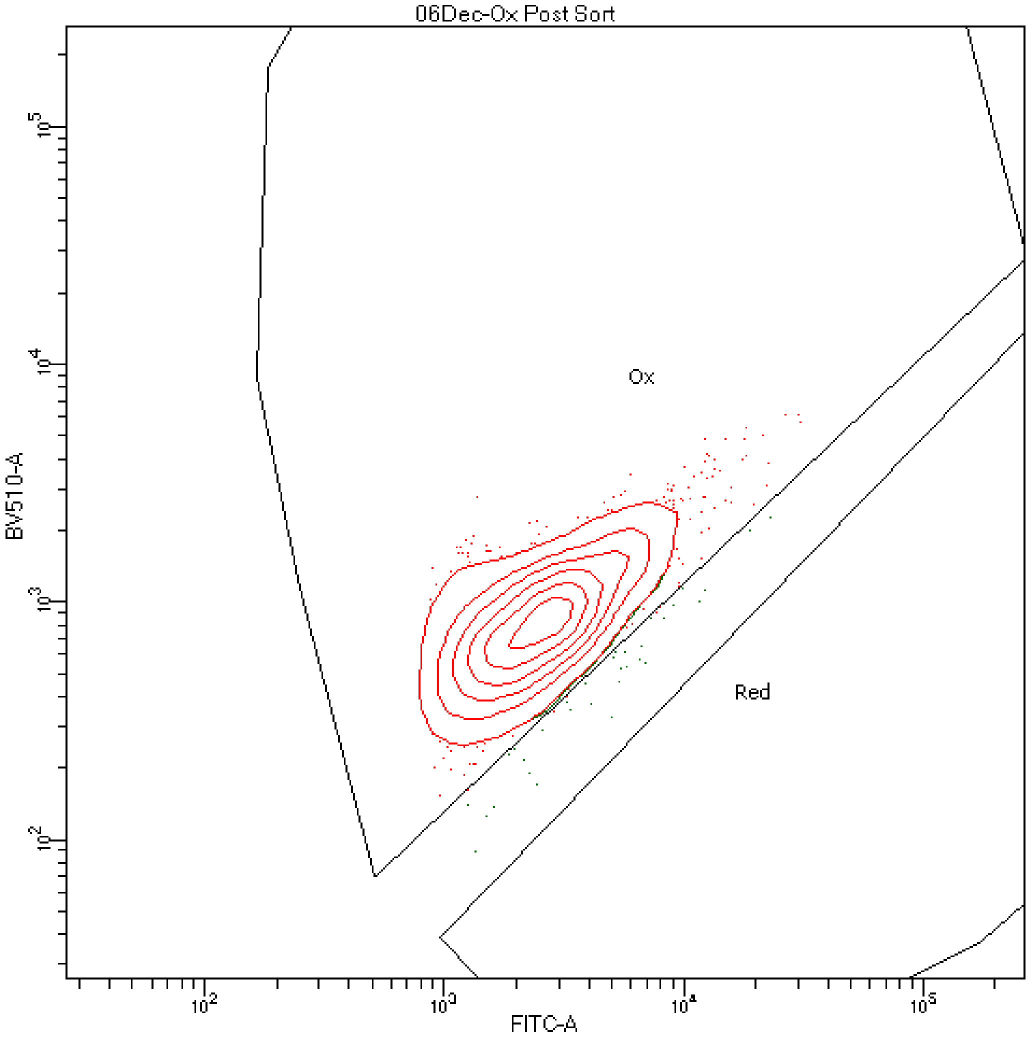

BD FACSDiva 8.0.1

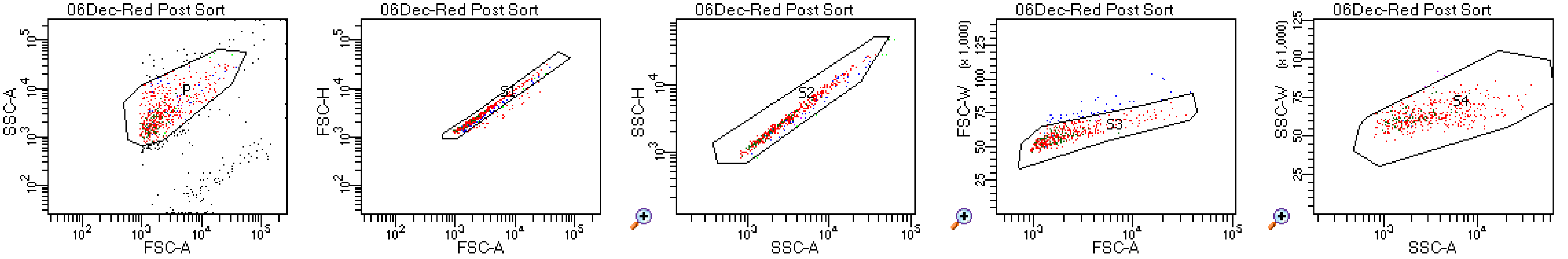

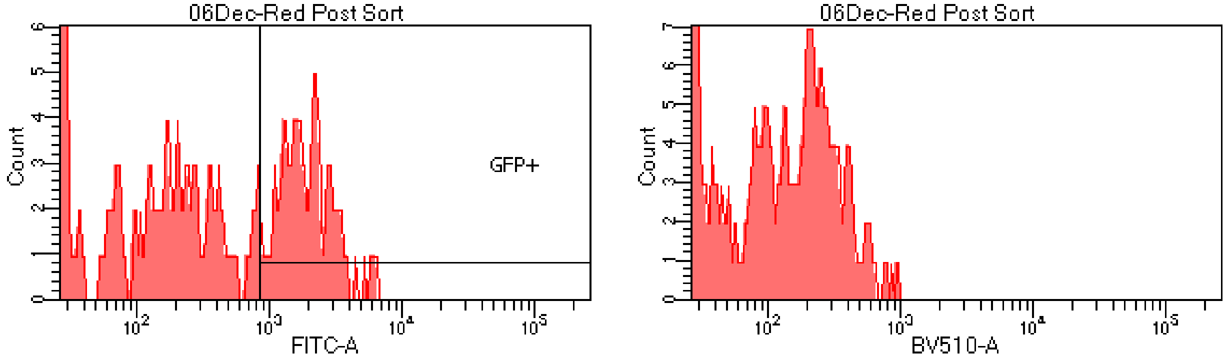

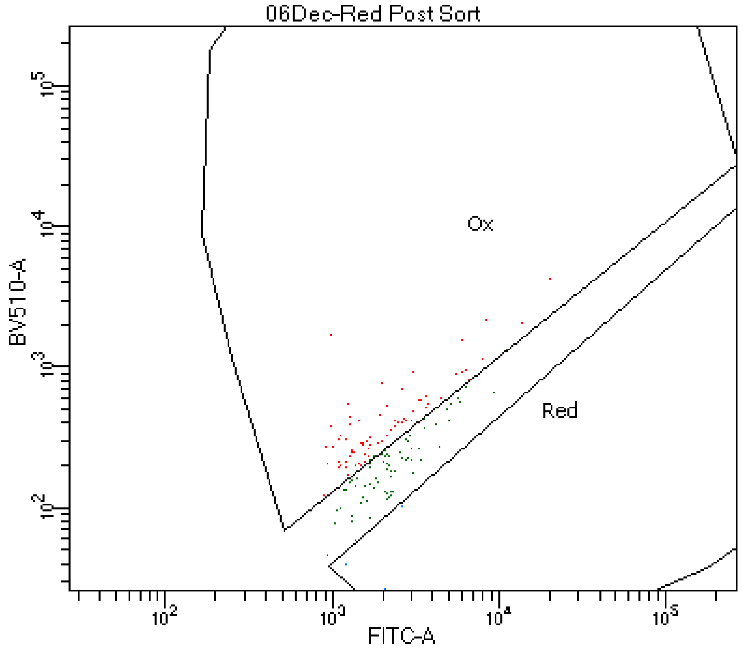

Tube: Red Post Sort

Population	#Events	%Parent	%Total
All Events	716	####	100.0
P	520	72.6	72.6
S1	488	93.8	68.2
S2	480	98.4	67.0
S3	442	92.1	61.7
S4	439	99.3	61.3
GFP+	149	33.9	20.8
Ox	73	49.0	10.2
Red	3	2.0	0.4

Experiment Name:	05EDec2016 Bac sorting
Specimen Name:	06Dec
Tube Name:	Red Post Sort
Record Date:	Dec 6, 2016 4:24:19 PM
$OP:	Administrator
GUID:	62e0500e-311c-4bb2-ae4f-c8e...

Population	#Events	%Parent	FITC-A Median	BV510-A Median
S4	439	99.3	258	114
GFP+	149	33.9	2,055	247
Ox	73	49.0	1,646	375
Red	3	2.0	2,064	39

BD FACSDiva 8.0.1

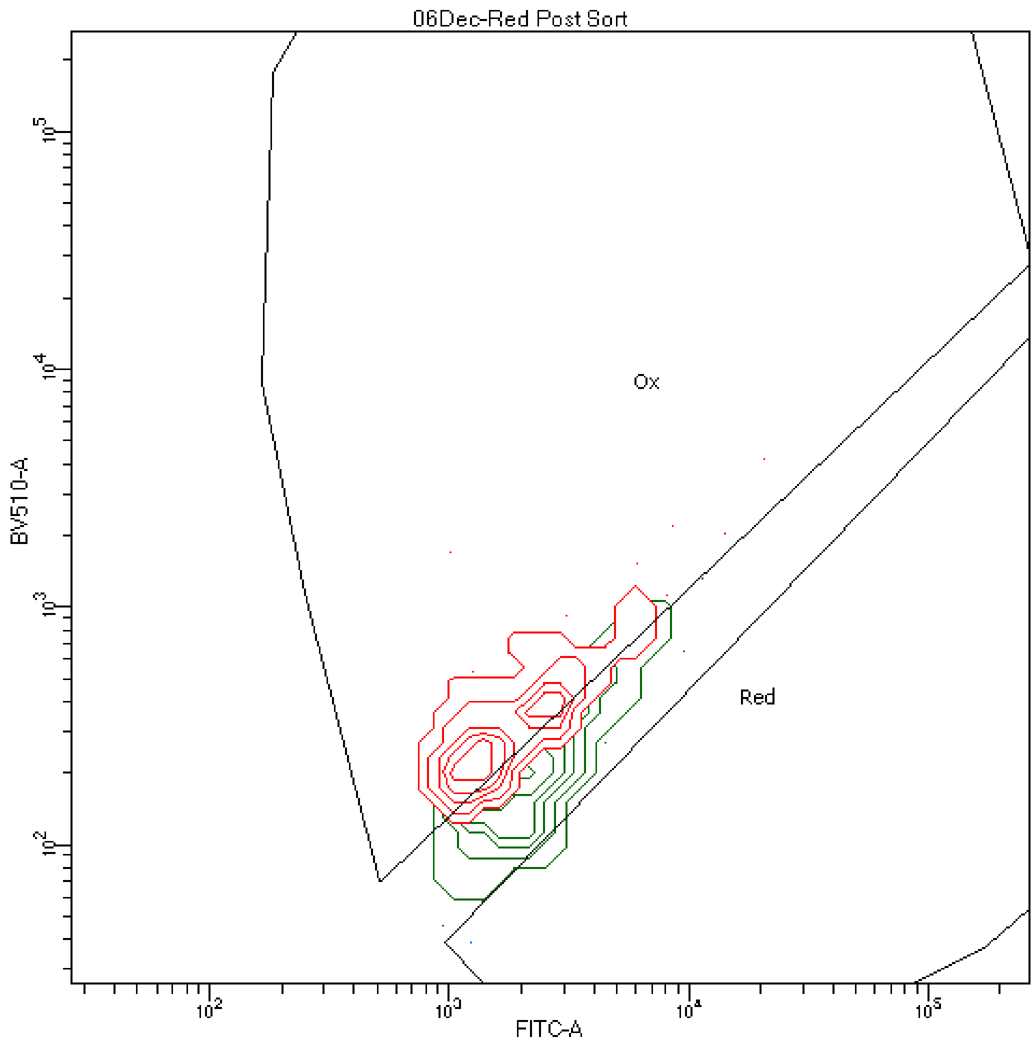

Supplement: Figure 1—source data 1. [file elife-80218-fig1-data1.zip › Round 3 Sorting/06EDec2016 Bac sorting-Batch_Analysis.pdf]
