# Supplementary material for: Biosensor-integrated transposon mutagenesis reveals rv0158 as a coordinator of redox homeostasis in Mycobacterium tuberculosis
Source: eLife. 2023 Aug 29;12:e80218. doi: 10.7554/eLife.80218 (PMC10501769; doi:10.7554/eLife.80218)
Supplement: Figure 1—source data 1. [file elife-80218-fig1-data1.zip › Round 3 Sorting/20Mar2017 Bac_001-Batch_Analysis_20032017150335.pdf]

Batch Analysis Report

Run Date: 3/20/17 3:03 PM Experiment: 09Mar2017 Bac_001

User ID: Administrator

Statistics Output: C:\Users\Admin\Desktop\09Mar2017 Bac_001-Batch_Analysis_20032017150335.csv

Worksheet PDF Output: C:\Users\Admin\Desktop\09Mar2017 Bac_001-Batch_Analysis_20032017150335.pdf

RVMrx1

Tube	Status	Run Time
US	OK	3/20/17 3:03 PM
RVMrx1	OK	3/20/17 3:03 PM
RVMrx1_001	OK	3/20/17 3:03 PM
TNlib R2	OK	3/20/17 3:03 PM
post sort	OK	3/20/17 3:03 PM
chp1	OK	3/20/17 3:03 PM
chp1_001	OK	3/20/17 3:03 PM
dtt1	OK	3/20/17 3:03 PM
dtt1_001	OK	3/20/17 3:03 PM

BD FACSDiva 8.0.1

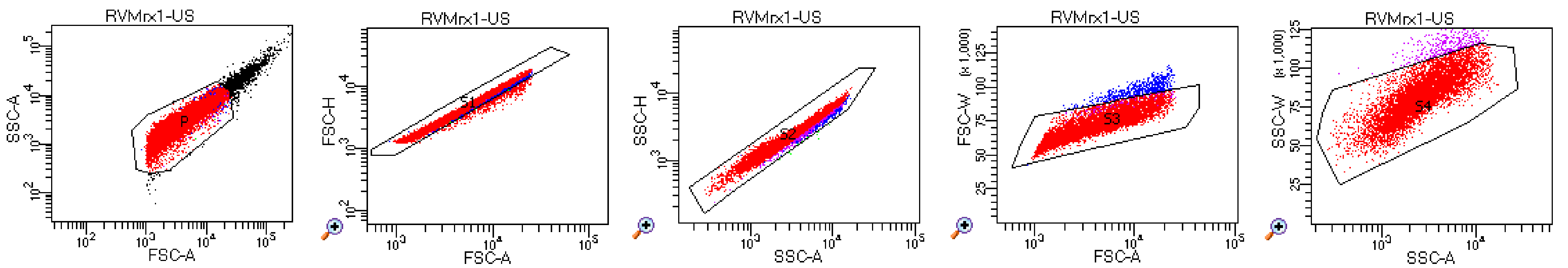

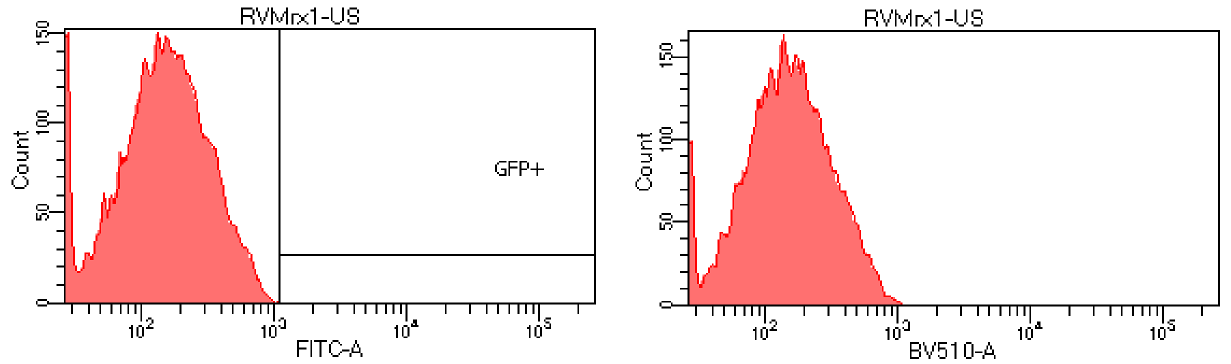

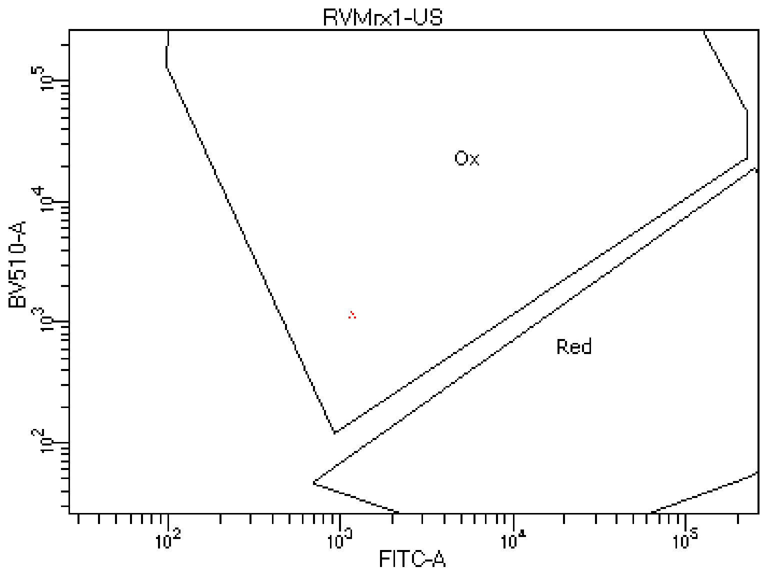

Tube: US

Population	#Events	%Parent	%Total
All Events	10,952	####	100.0
P	9,462	86.4	86.4
S1	8,838	93.4	80.7
S2	8,819	99.8	80.5
S3	8,175	92.7	74.6
S4	7,803	95.4	71.2
GFP+	4	0.1	0.0
Ox	4	100.0	0.0
Red	0	0.0	0.0

Experiment Name: 09Mar2017 Bac_001
Specimen Name: RVMrx1
Tube Name: US
Record Date: Mar 20, 2017 10:23:00 AM
$OP: Administrator
GUID: 787426f1-3435-4d5b-9b72-df6cb91...

Population	#Events	%Parent	FITC-A Median	BV510-A Median
S4	7,803	95.4	146	143
GFP+	4	0.1	1,149	1,128
Ox	4	100.0	1,149	1,128
Red	0	0.0	####	####

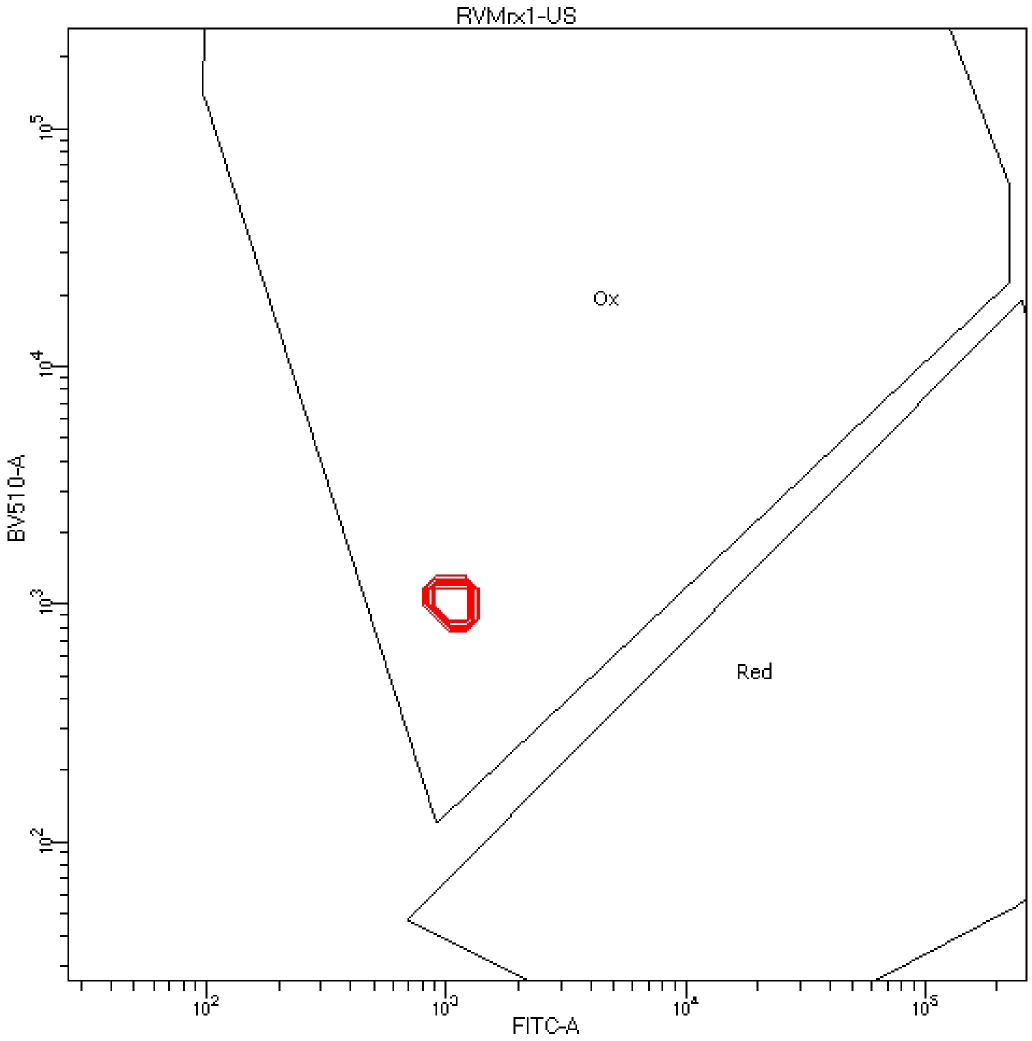

RVMrx1-US
Ox
Red
BV510-A
FITC-A
10^2
10^3
10^4
10^5

BD FACSDiva 8.0.1

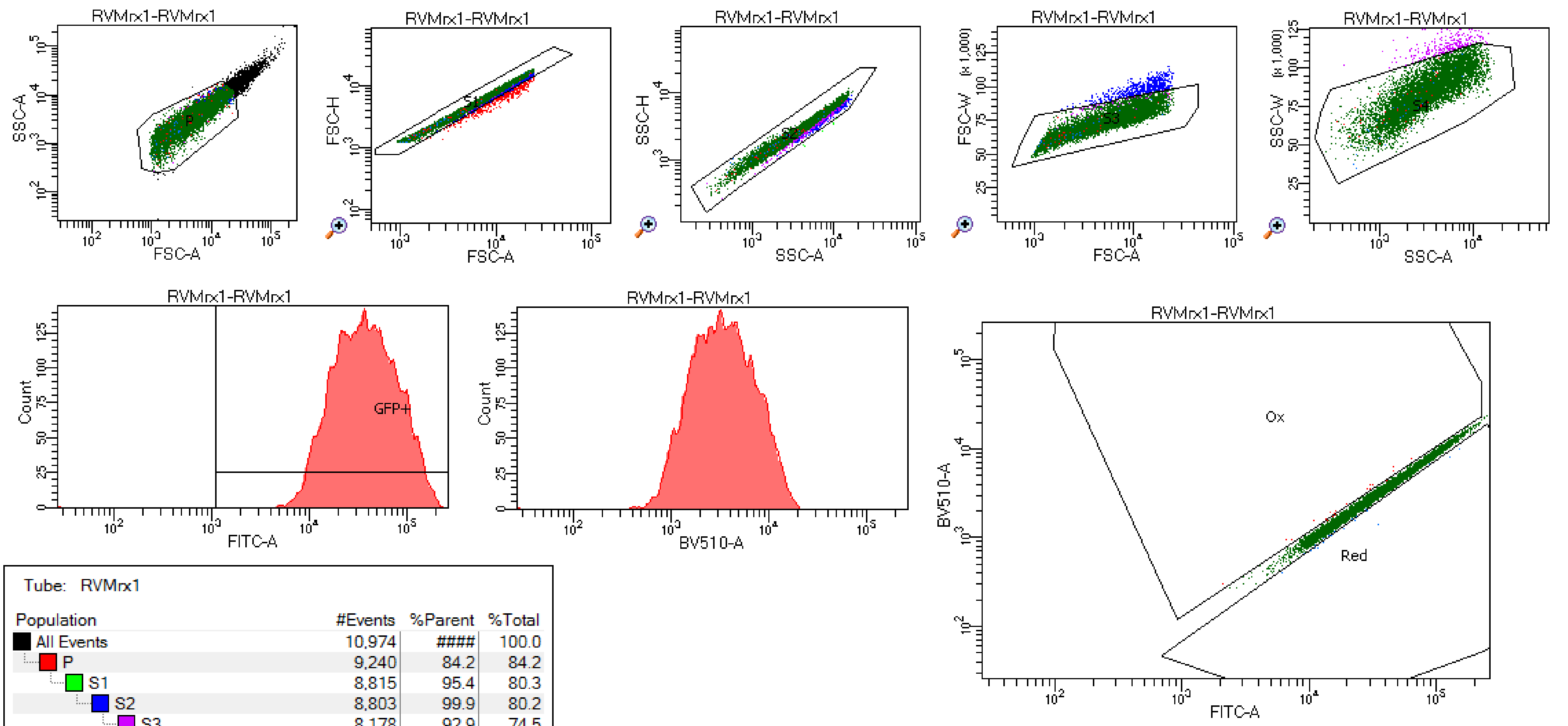

Tube: RVMrx1

Population	#Events	%Parent	%Total
All Events	10,974	####	100.0
P	9,240	84.2	84.2
S1	8,815	95.4	80.3
S2	8,803	99.9	80.2
S3	8,178	92.9	74.5
S4	7,861	96.1	71.6
GFP+	7,835	99.7	71.4
Ox	31	0.4	0.3
Red	32	0.4	0.3

Experiment Name:	09Mar2017 Bac_001
Specimen Name:	RVMrx1
Tube Name:	RVMrx1
Record Date:	Mar 20, 2017 10:23:38 AM
$OP:	Administrator
GUID:	1cab095d-0d4f-434f-98da-b3612d1...

Population	#Events	%Parent	FITC-A Median	BV510-A Median
S4	7,861	96.1	35,461	3,193
GFP+	7,835	99.7	35,615	3,200
Ox	31	0.4	28,478	3,611
Red	32	0.4	15,303	1,115

BD FACSDiva 8.0.1

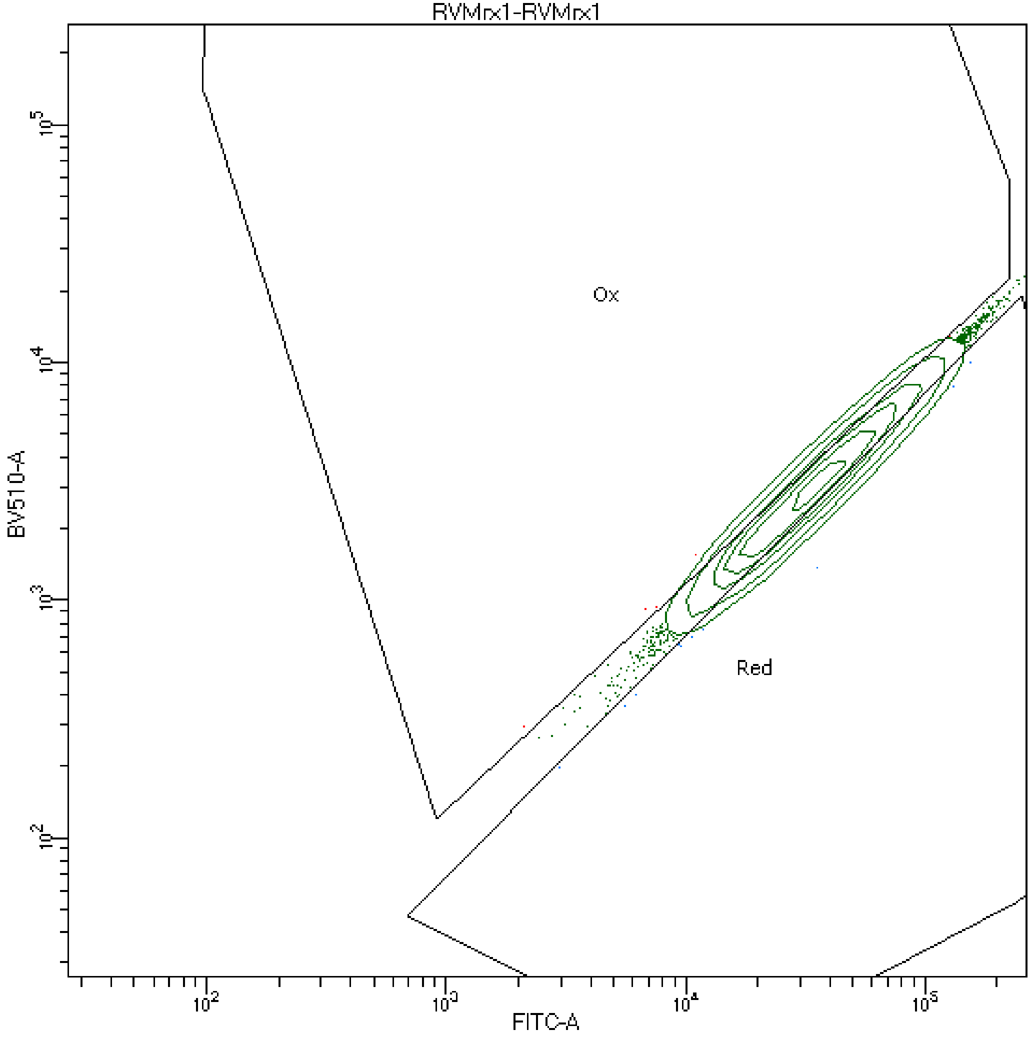

BD FACSDiva 8.0.1

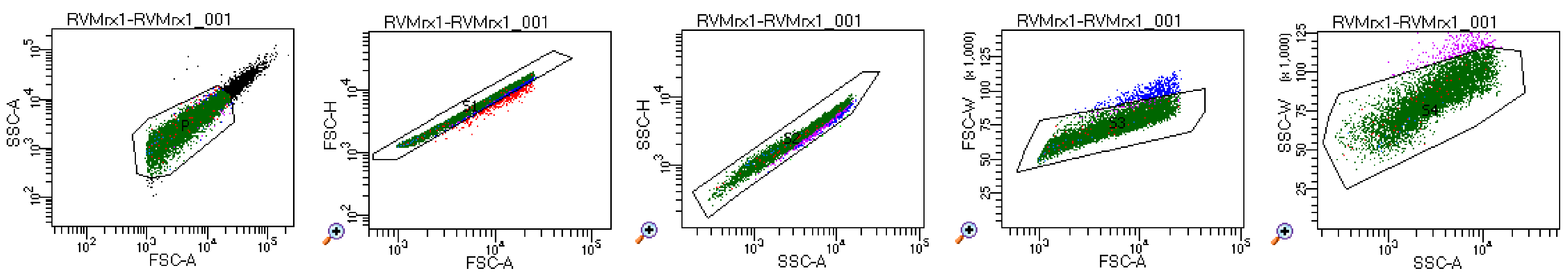

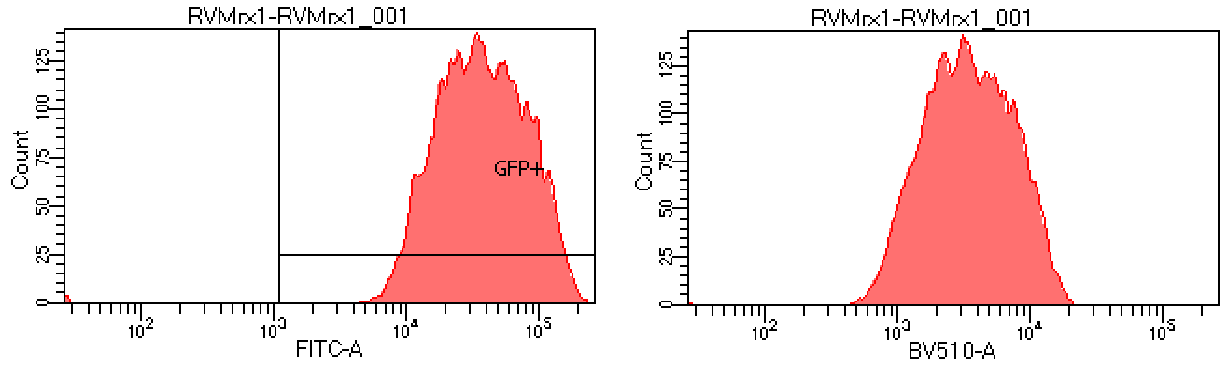

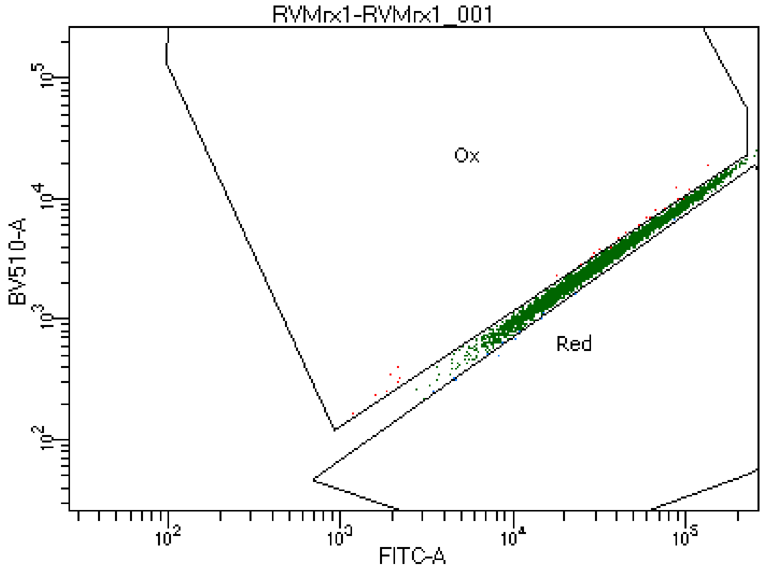

Tube: RVMrx1_001

Population	#Events	%Parent	%Total
All Events	10,927	####	100.0
P	9,328	85.4	85.4
S1	8,957	96.0	82.0
S2	8,948	99.9	81.9
S3	8,382	93.7	76.7
S4	8,080	96.4	73.9
GFP+	8,046	99.6	73.6
Ox	30	0.4	0.3
Red	17	0.2	0.2

Experiment Name: 09Mar2017 Bac_001
Specimen Name: RVMrx1
Tube Name: RVMrx1_001
Record Date: Mar 20, 2017 10:24:16 AM
$OP: Administrator
GUID: 452fdc5f-2402-47fc-b5b8-b2d4708a...

Population	#Events	%Parent	FITC-A Median	BV510-A Median
S4	8,080	96.4	35,518	3,236
GFP+	8,046	99.6	35,683	3,256
Ox	30	0.4	37,058	4,016
Red	17	0.2	10,318	696

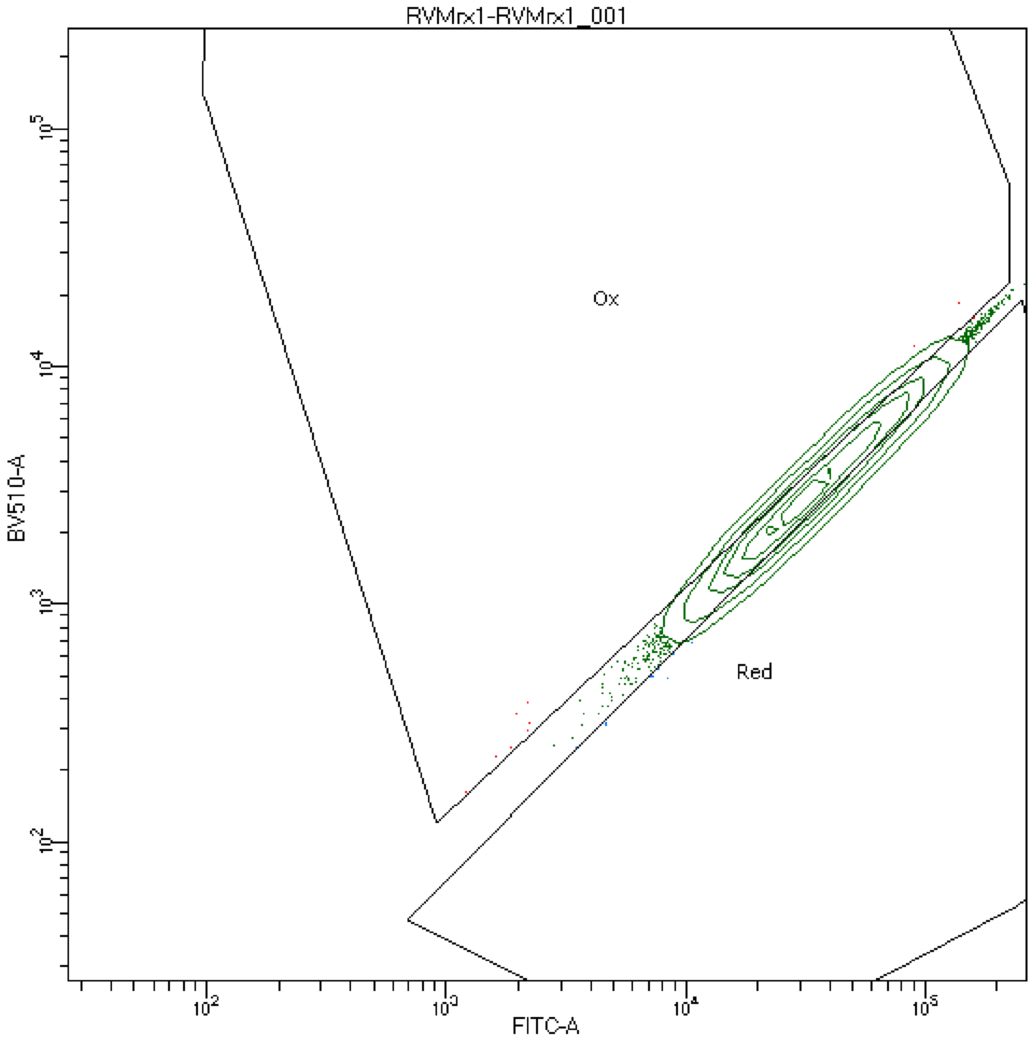

RVMrx1-RVMrx1_001
Ox
Red
BV510-A
FITC-A
10^2
10^3
10^4
10^5

BD FACSDiva 8.0.1

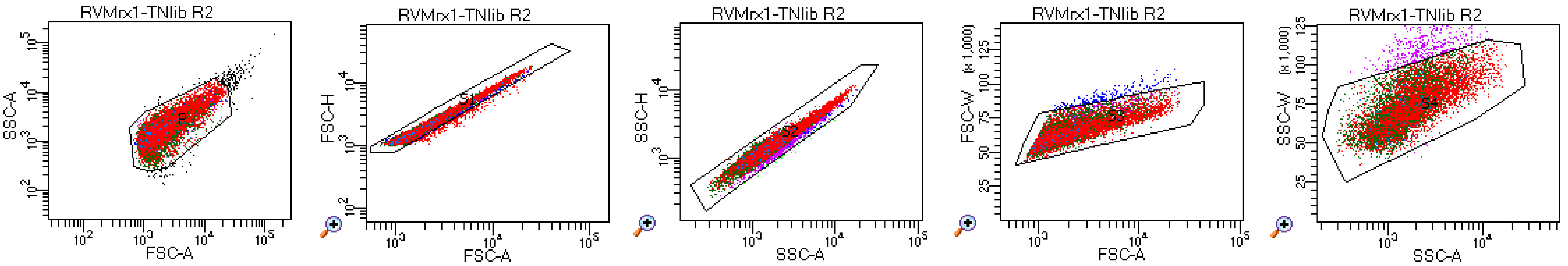

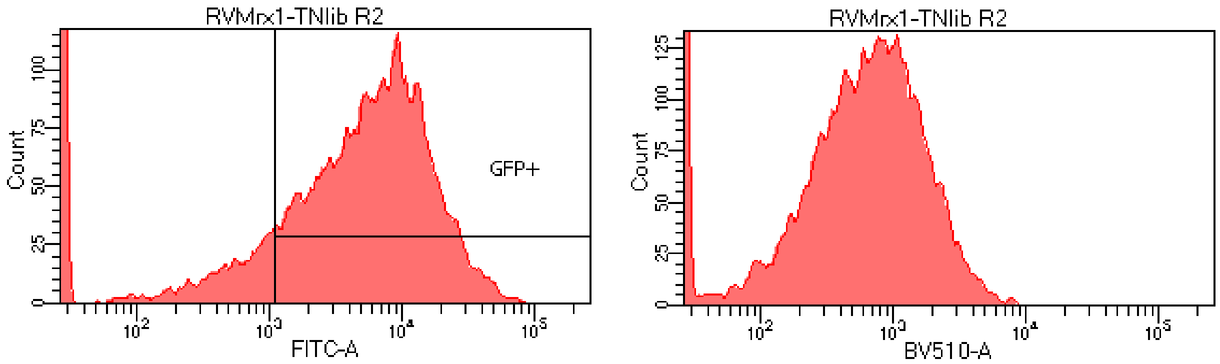

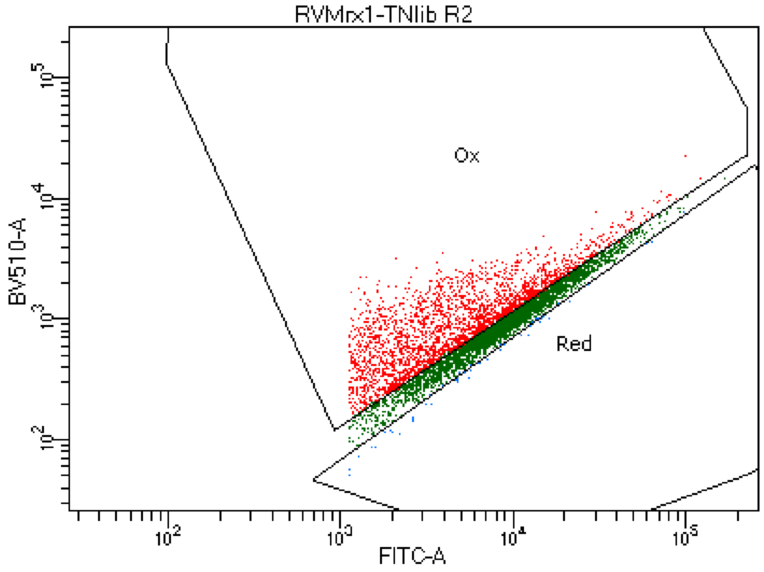

Tube: TNlib R2

Population	#Events	%Parent	%Total
All Events	10,218	####	100.0
P	10,000	97.9	97.9
S1	9,686	96.9	94.8
S2	9,647	99.6	94.4
S3	9,444	97.9	92.4
S4	9,003	95.3	88.1
GFP+	6,403	71.1	62.7
Ox	2,541	39.7	24.9
Red	70	1.1	0.7

Experiment Name: 09Mar2017 Bac_001
Specimen Name: RVMrx1
Tube Name: TNlib R2
Record Date: Mar 20, 2017 12:52:13 PM
$OP: Administrator
GUID: 5c60819e-6c23-47ff-8931-37bb869...

Population	#Events	%Parent	FITC-A Median	BV510-A Median
S4	9,003	95.3	3,962	617
GFP+	6,403	71.1	6,849	871
Ox	2,541	39.7	3,989	905
Red	70	1.1	6,262	433

BD FACSDiva 8.0.1

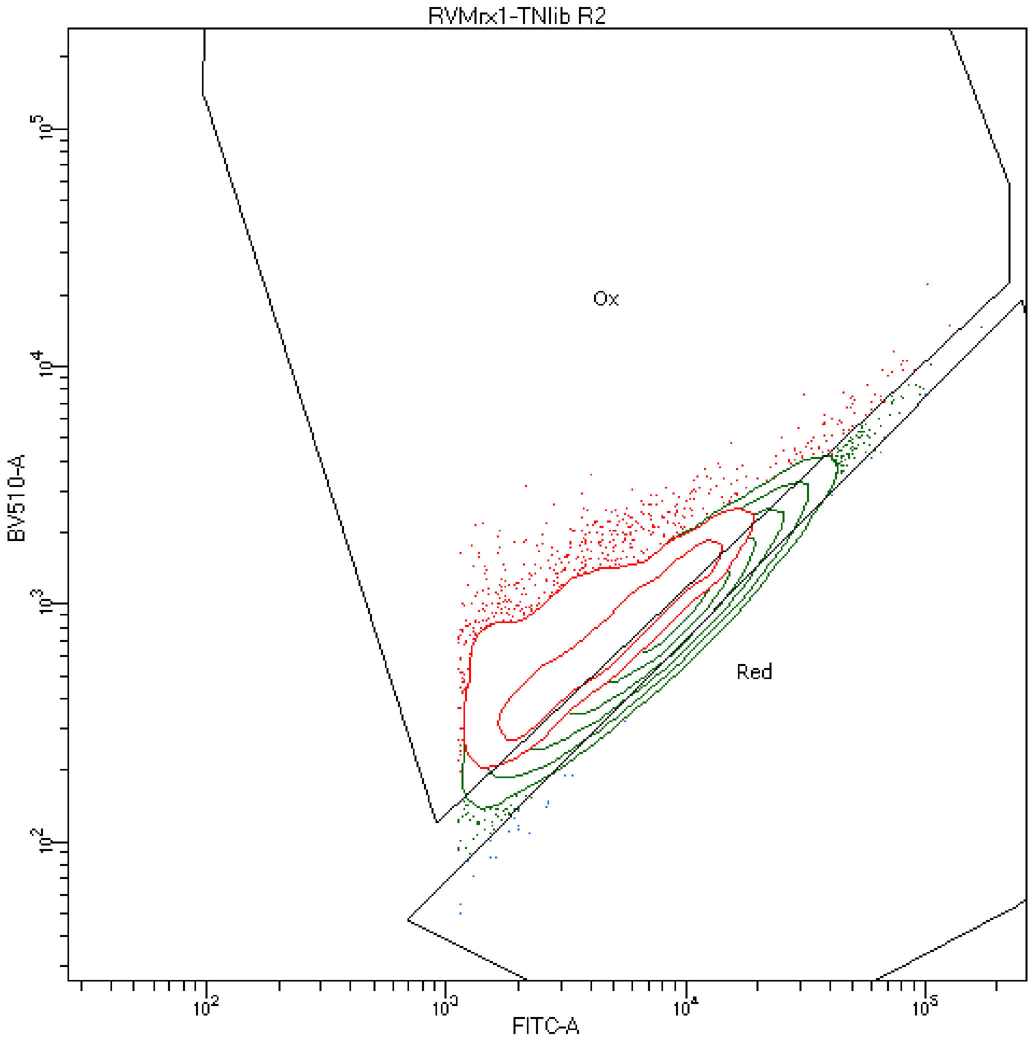

BD FACSDiva 8.0.1

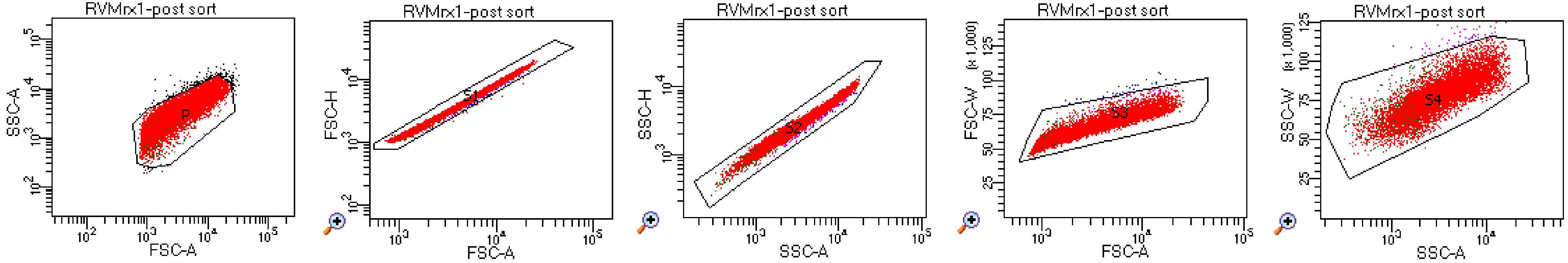

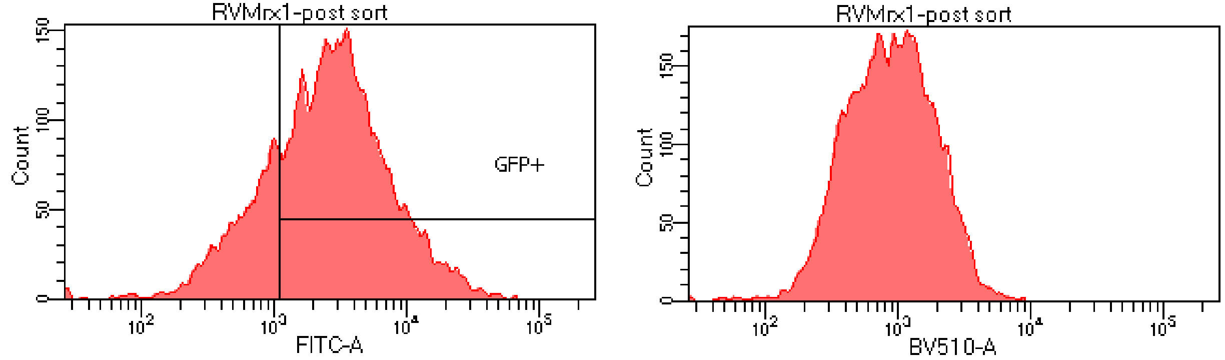

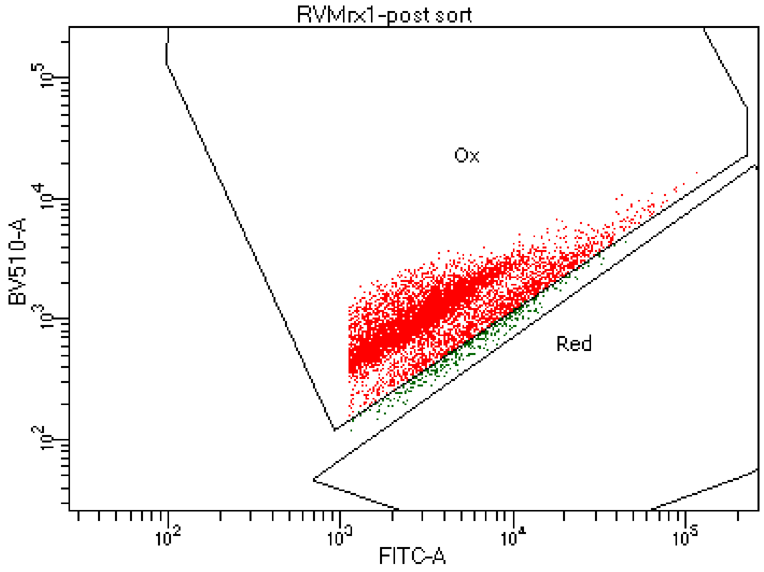

Tube: post sort

Population	#Events	%Parent	%Total
All Events	10,195	####	100.0
P	10,000	98.1	98.1
S1	9,981	99.8	97.9
S2	9,979	100.0	97.9
S3	9,945	99.7	97.5
S4	9,877	99.3	96.9
GFP+	7,508	76.0	73.6
Ox	7,095	94.5	69.6
Red	0	0.0	0.0

Experiment Name: 09Mar2017 Bac_001
Specimen Name: RVMrx1
Tube Name: post sort
Record Date: Mar 20, 2017 2:39:48 PM
$OP: Administrator
GUID: cb10e07c-d1d2-4262-aa87-db5552...

Population	#Events	%Parent	FITC-A Median	BV510-A Median
S4	9,877	99.3	2,470	847
GFP+	7,508	76.0	3,330	1,089
Ox	7,095	94.5	3,232	1,116
Red	0	0.0	####	####

BD FACSDiva 8.0.1

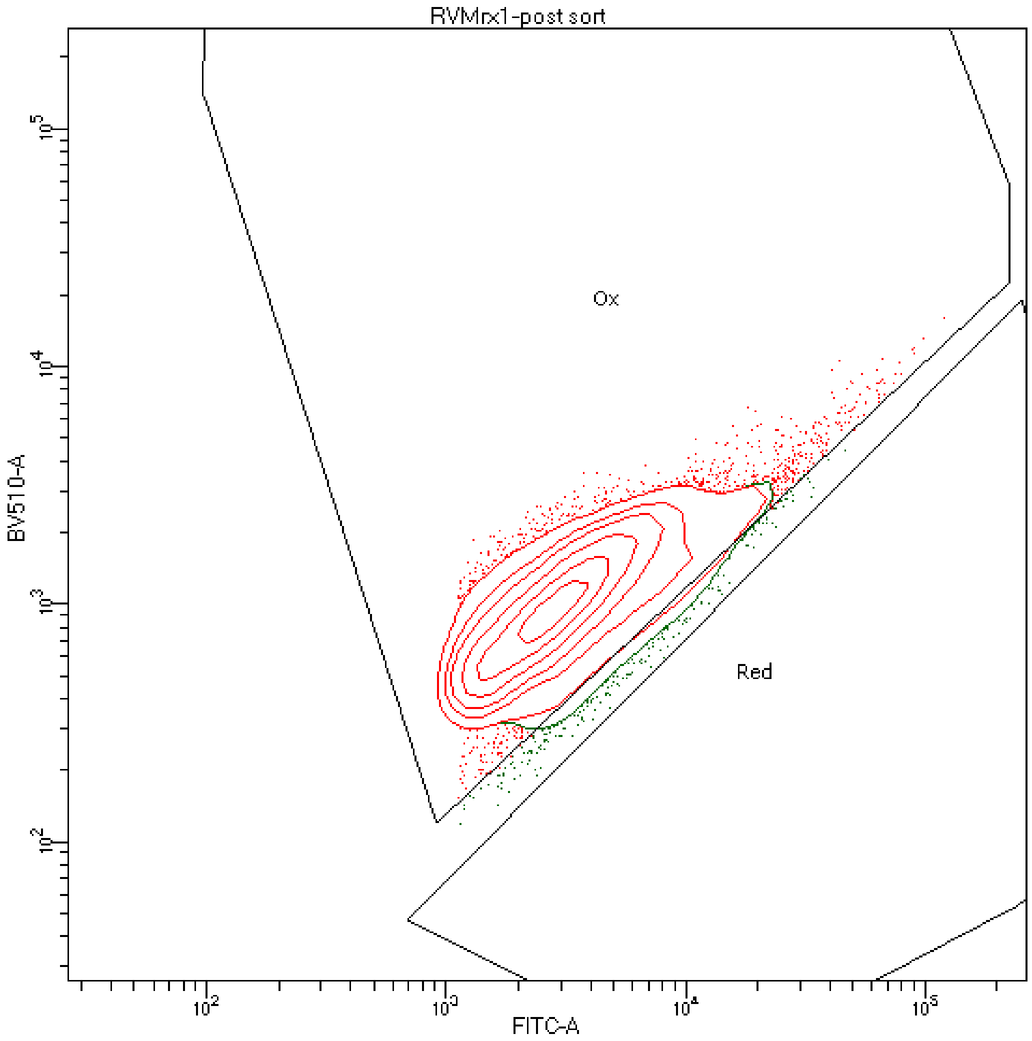

BD FACSDiva 8.0.1

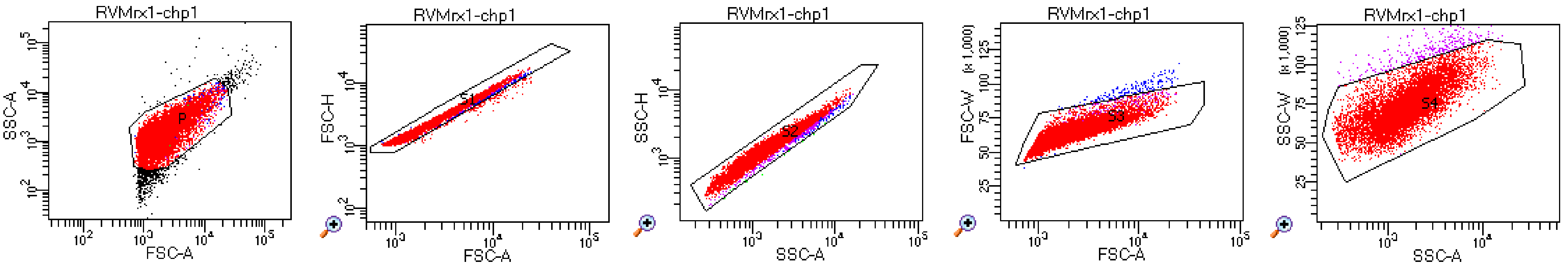

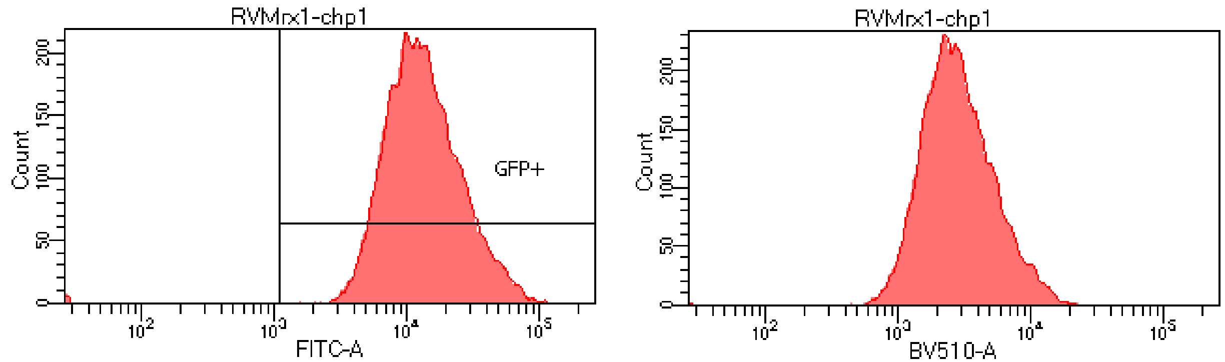

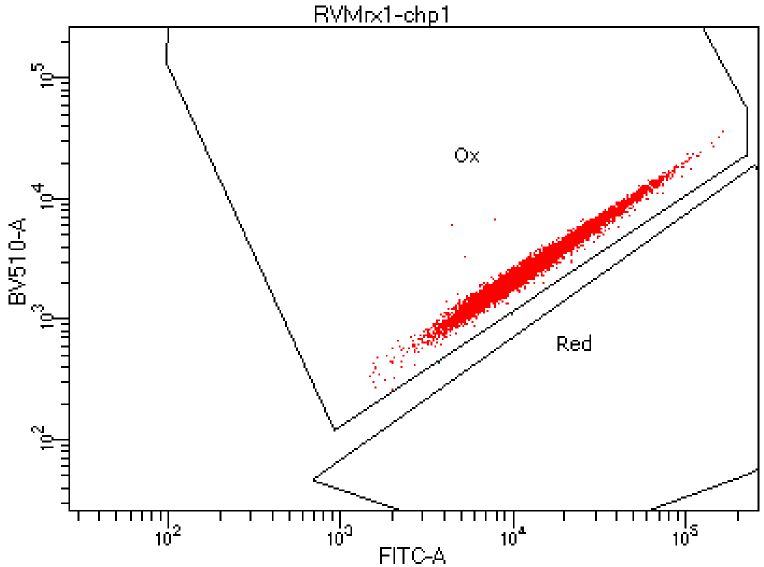

Tube: chp1

Population	#Events	%Parent	%Total
All Events	10,639	####	100.0
P	10,000	94.0	94.0
S1	9,843	98.4	92.5
S2	9,827	99.8	92.4
S3	9,646	98.2	90.7
S4	9,337	96.8	87.8
GFP+	9,306	99.7	87.5
Ox	9,304	100.0	87.5
Red	0	0.0	0.0

Experiment Name:	09Mar2017 Bac_001
Specimen Name:	RVMrx1
Tube Name:	chp1
Record Date:	Mar 20, 2017 2:40:58 PM
$OP:	Administrator
GUID:	603a787e-4a19-483a-887f-3958de4...

Population	#Events	%Parent	FITC-A Median	BV510-A Median
S4	9,337	96.8	12,324	2,603
GFP+	9,306	99.7	12,350	2,606
Ox	9,304	100.0	12,352	2,606
Red	0	0.0	####	####

BD FACSDiva 8.0.1

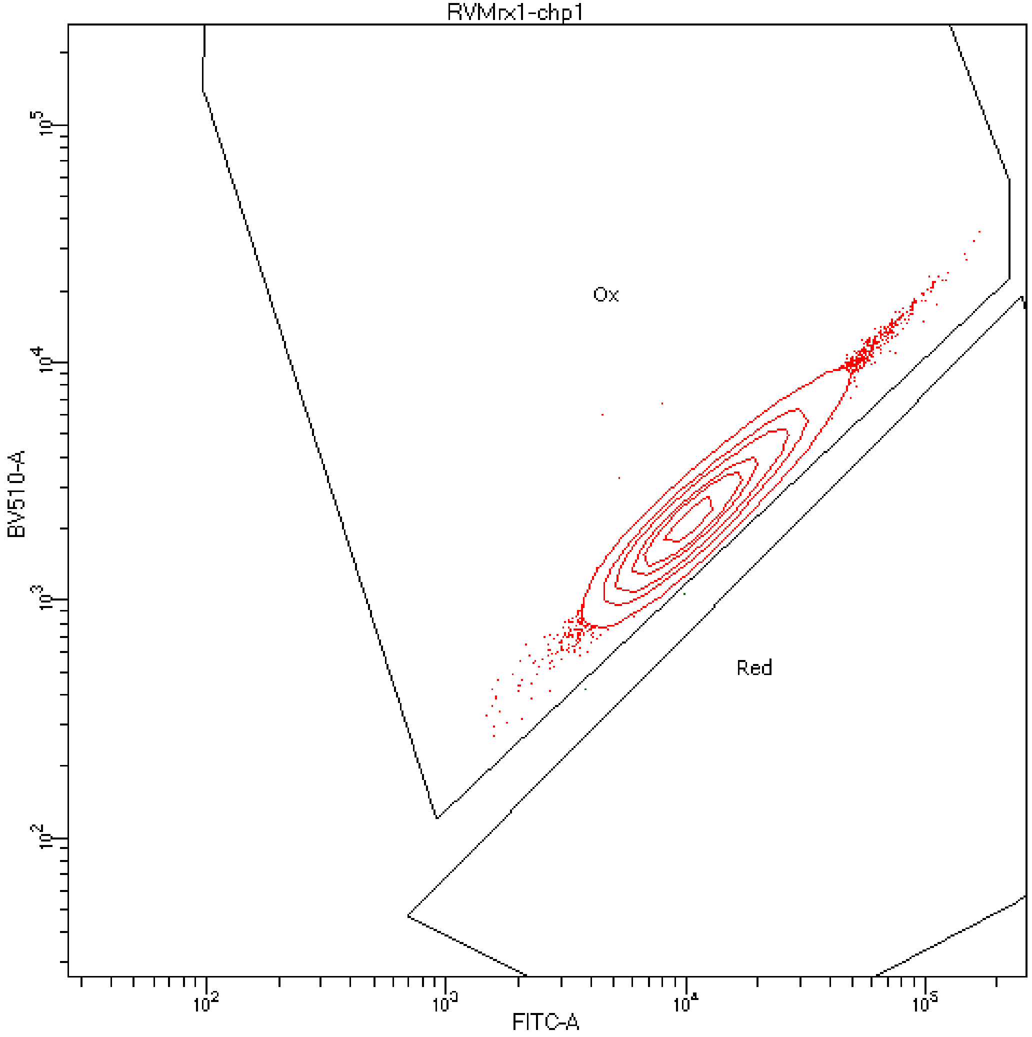

BD FACSDiva 8.0.1

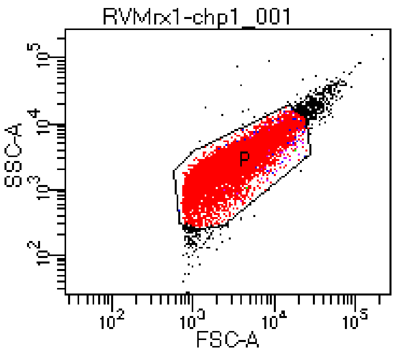

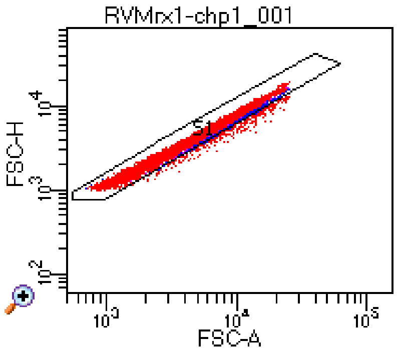

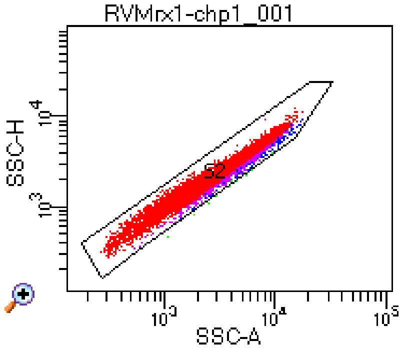

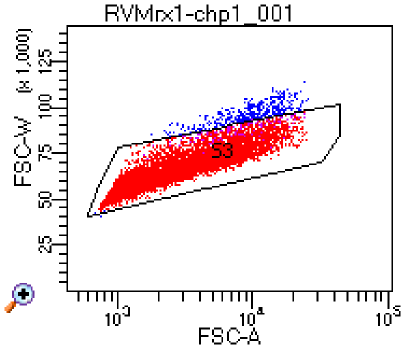

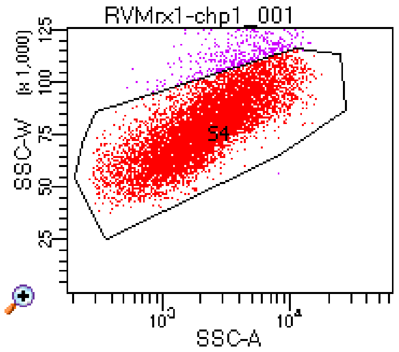

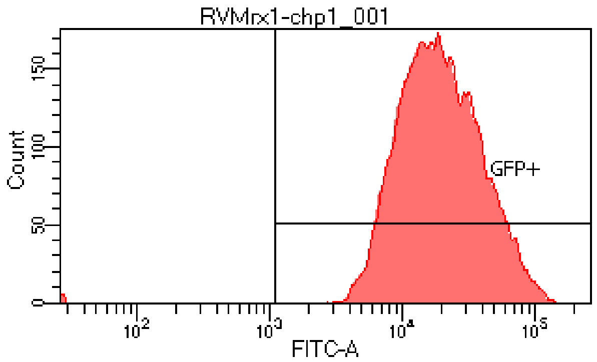

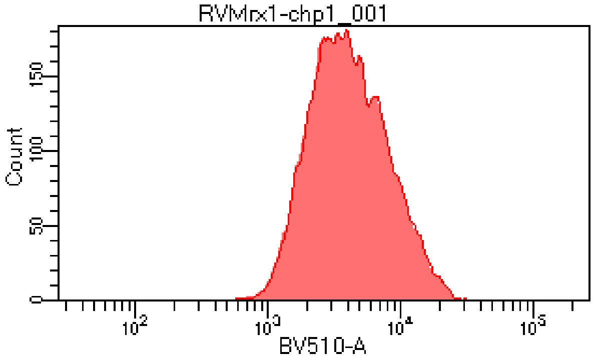

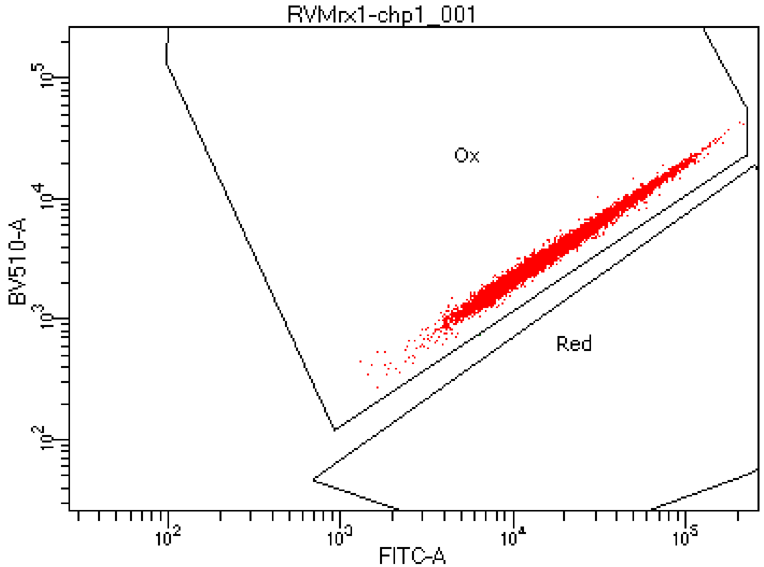

Tube: chp1_001

Population	#Events	%Parent	%Total
All Events	10,642	####	100.0
P	10,000	94.0	94.0
S1	9,667	96.7	90.8
S2	9,648	99.8	90.7
S3	9,219	95.6	86.6
S4	8,809	95.6	82.8
GFP+	8,784	99.7	82.5
Ox	8,783	100.0	82.5
Red	0	0.0	0.0

Experiment Name:	09Mar2017 Bac_001
Specimen Name:	RVMrx1
Tube Name:	chp1_001
Record Date:	Mar 20, 2017 2:41:51 PM
$OP:	Administrator
GUID:	97088fa8-be80-4712-8fc7-9486c5a...

Population	#Events	%Parent	FITC-A Median	BV510-A Median
S4	8,809	95.6	17,920	3,761
GFP+	8,784	99.7	17,985	3,767
Ox	8,783	100.0	17,987	3,767
Red	0	0.0	####	####

BD FACSDiva 8.0.1

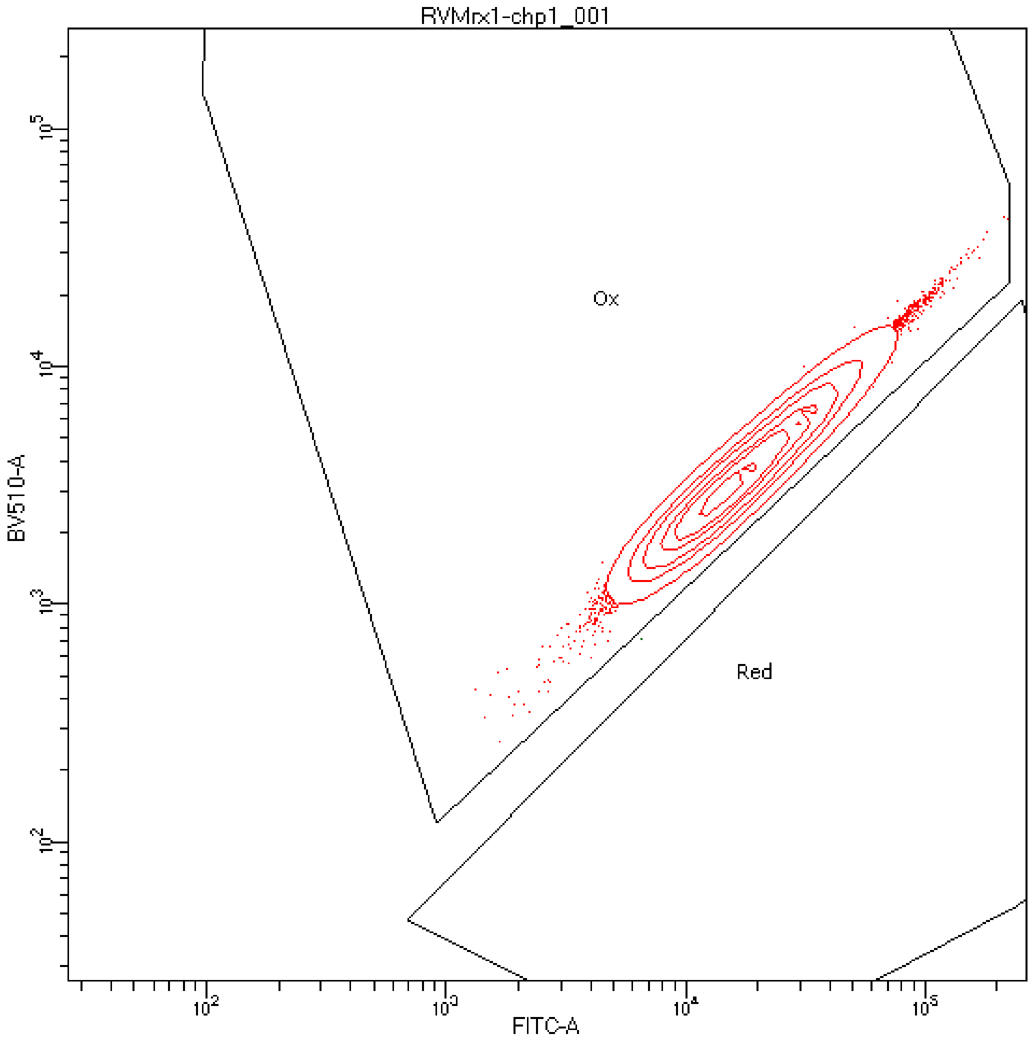

BD FACSDiva 8.0.1

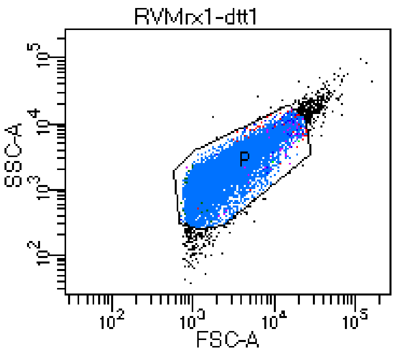

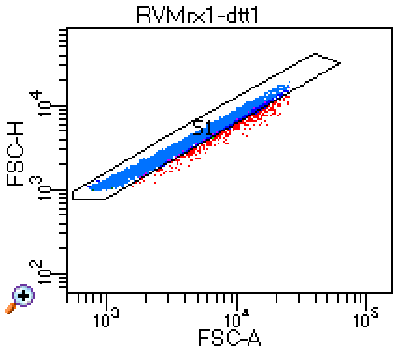

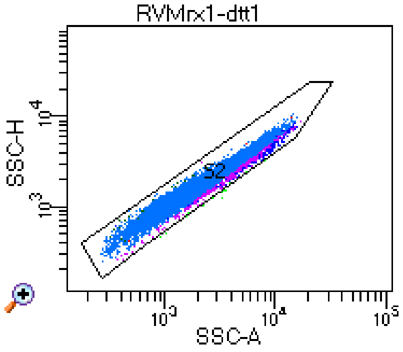

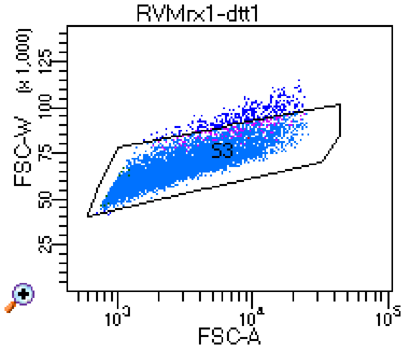

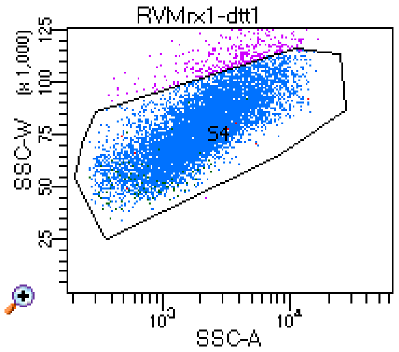

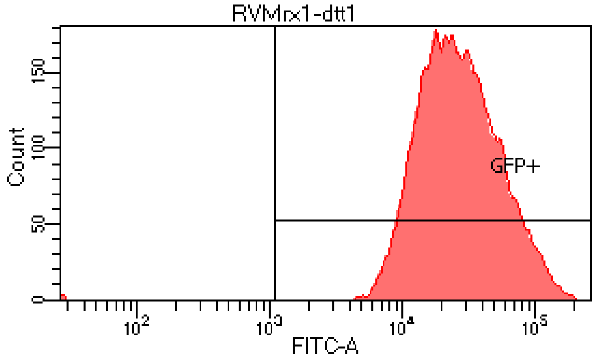

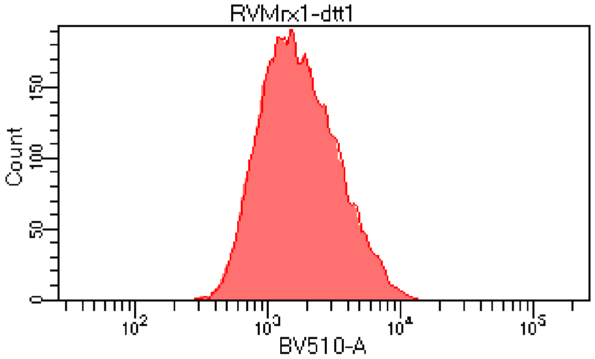

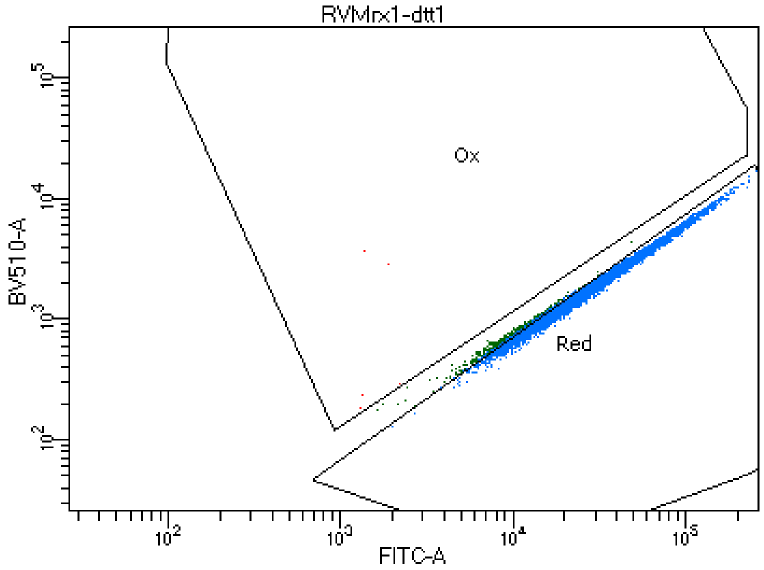

Tube: dtt1

Population	#Events	%Parent	%Total
All Events	10,541	####	100.0
P	10,000	94.9	94.9
S1	9,658	96.6	91.6
S2	9,639	99.8	91.4
S3	9,272	96.2	88.0
S4	8,873	95.7	84.2
GFP+	8,855	99.8	84.0
Ox	5	0.1	0.0
Red	8,500	96.0	80.6

Experiment Name:	09Mar2017 Bac_001
Specimen Name:	RVMrx1
Tube Name:	dtt1
Record Date:	Mar 20, 2017 2:42:52 PM
$OP:	Administrator
GUID:	87d22f1c-cd15-4ee9-9337-f8c4b08f...

Population	#Events	%Parent	FITC-A Median	BV510-A Median
S4	8,873	95.7	24,823	1,566
GFP+	8,855	99.8	24,868	1,570
Ox	5	0.1	1,348	289
Red	8,500	96.0	25,711	1,625

BD FACSDiva 8.0.1

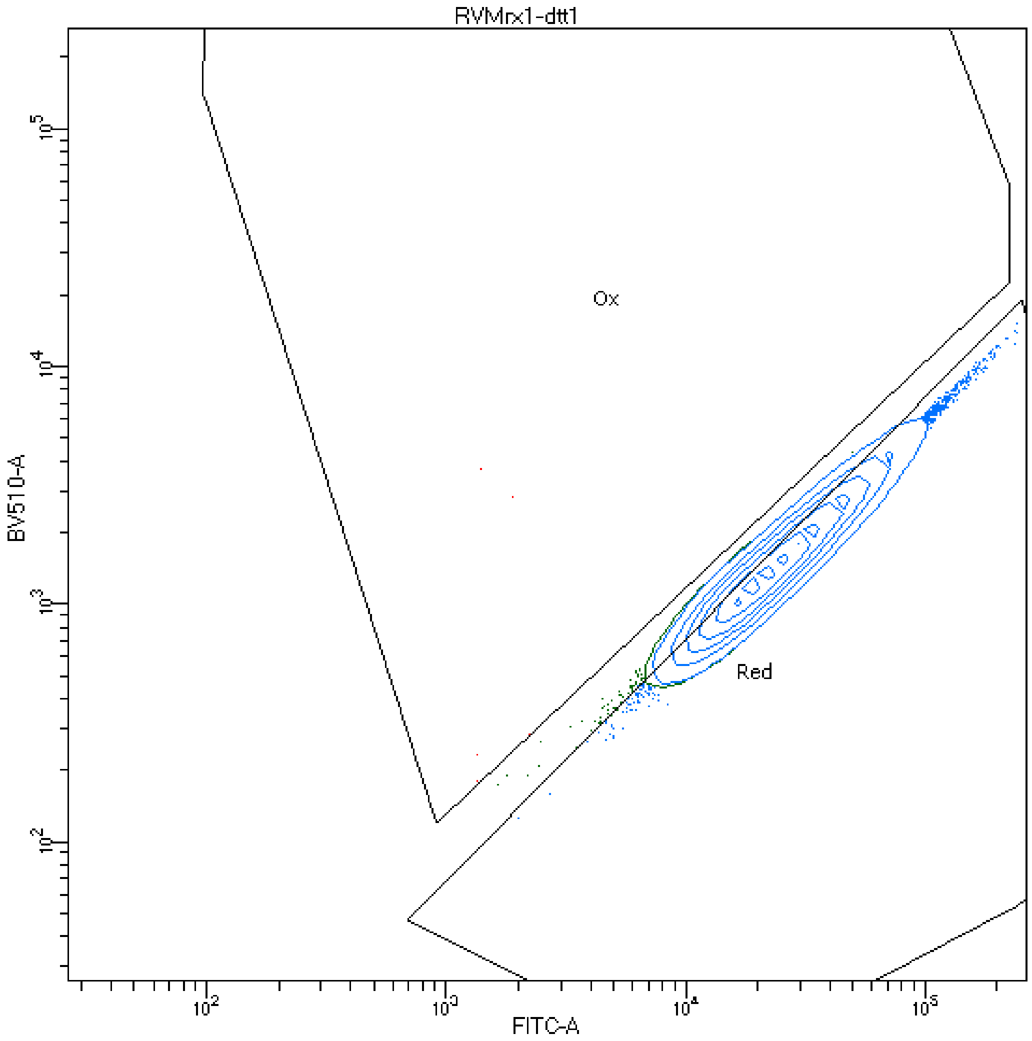

BD FACSDiva 8.0.1

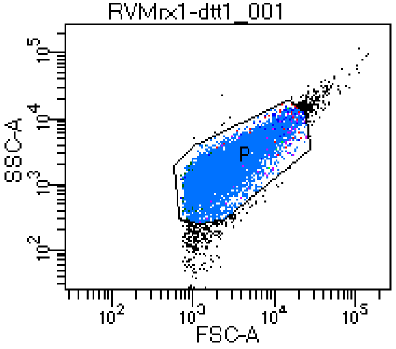

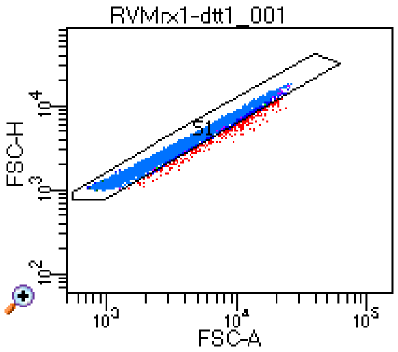

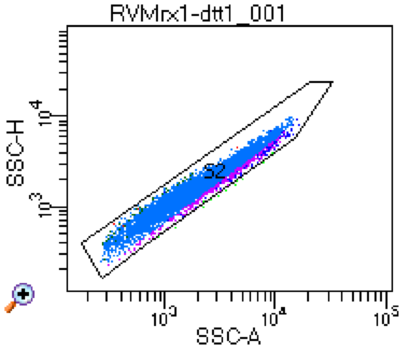

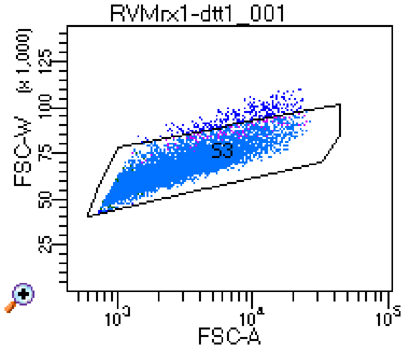

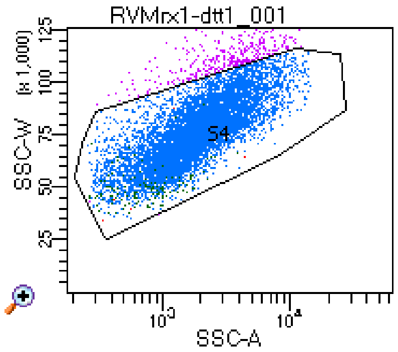

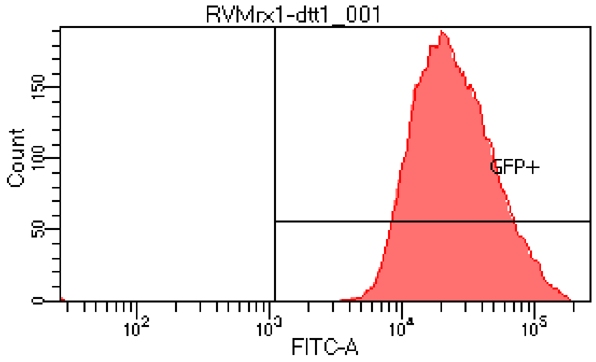

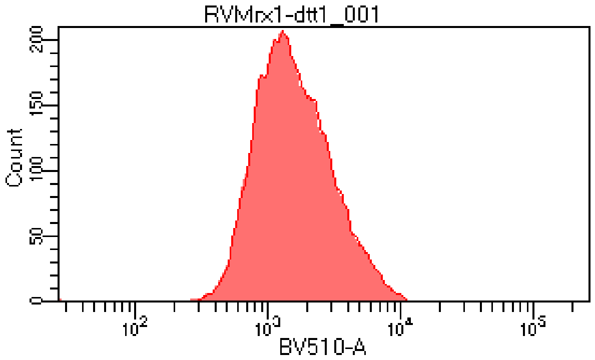

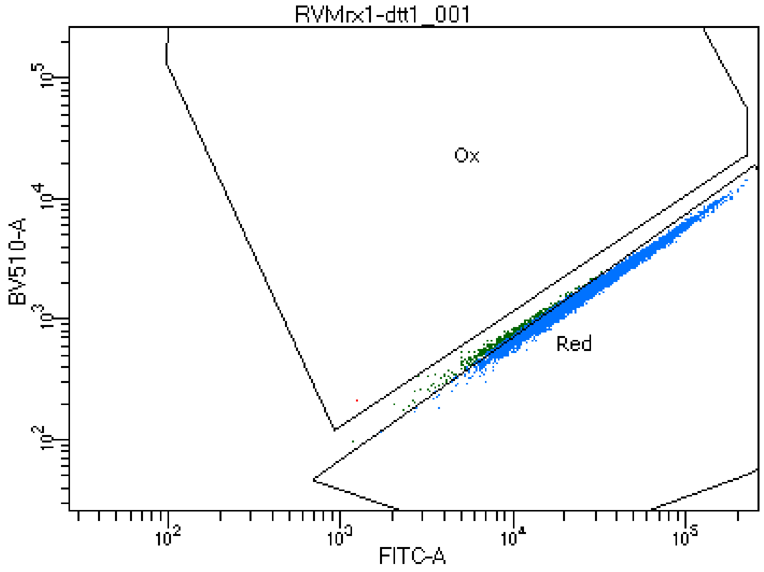

Tube: dtt1_001

Population	#Events	%Parent	%Total
All Events	10,547	####	100.0
P	10,000	94.8	94.8
S1	9,745	97.4	92.4
S2	9,726	99.8	92.2
S3	9,411	96.8	89.2
S4	9,050	96.2	85.8
GFP+	9,034	99.8	85.7
Ox	1	0.0	0.0
Red	8,477	93.8	80.4

Experiment Name: 09Mar2017 Bac_001
Specimen Name: RVMrx1
Tube Name: dtt1_001
Record Date: Mar 20, 2017 2:44:57 PM
$OP: Administrator
GUID: d63165cf-4c1d-45f7-a1da-ee1cfa79f...

Population	#Events	%Parent	FITC-A Median	BV510-A Median
S4	9,050	96.2	22,450	1,419
GFP+	9,034	99.8	22,472	1,421
Ox	1	0.0	1,212	210
Red	8,477	93.8	23,611	1,492

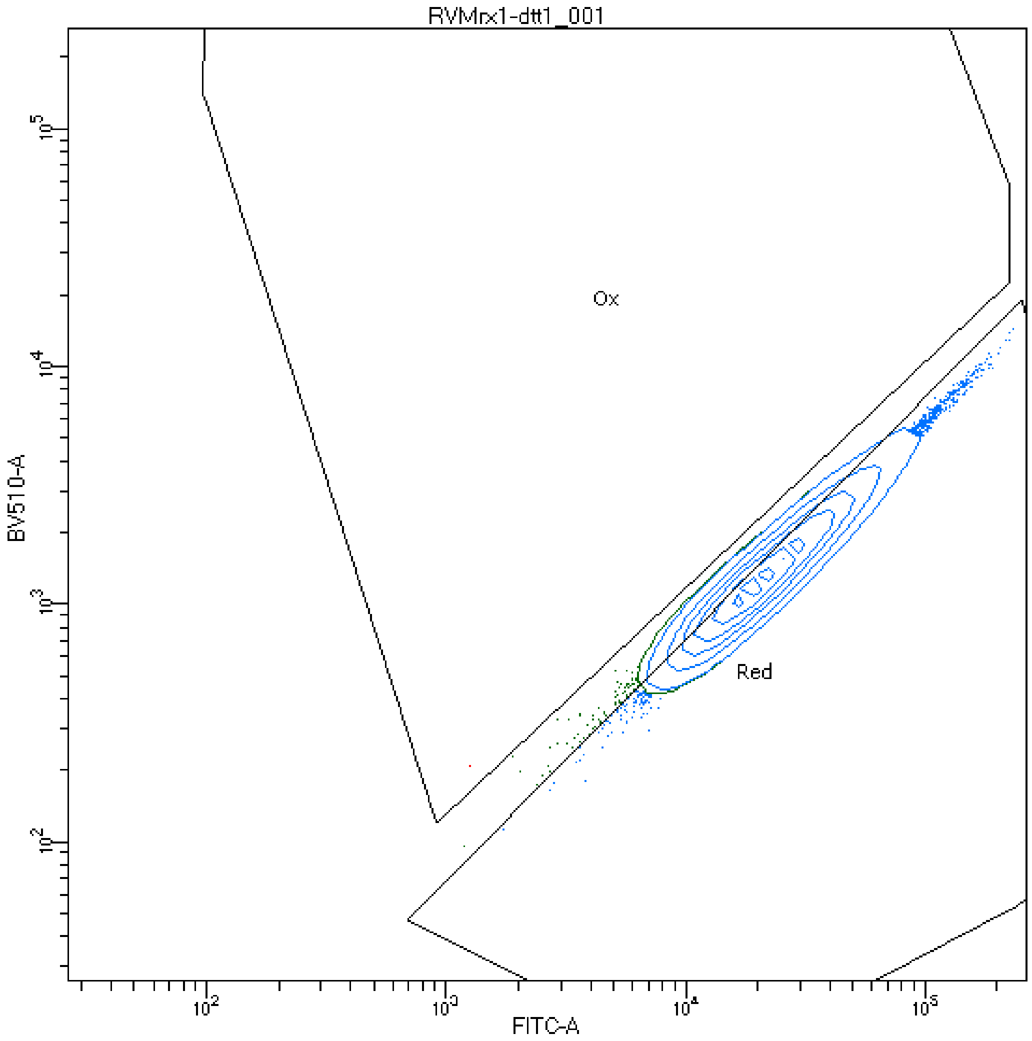
RVMrx1-dtt1_001
Ox
Red
BV510-A
FITC-A
10^2
10^3
10^4
10^5
