# Supplementary material for: Biosensor-integrated transposon mutagenesis reveals rv0158 as a coordinator of redox homeostasis in Mycobacterium tuberculosis
Source: eLife. 2023 Aug 29;12:e80218. doi: 10.7554/eLife.80218 (PMC10501769; doi:10.7554/eLife.80218)

Batch Analysis Report

Run Date: 2/28/17 12:55 PM　　Experiment: 31Jan2017 Bac sorting

User ID: Administrator

Statistics Output: N/A

Worksheet PDF Output: C:\Users\Admin\Desktop\31Jan2017 Bac sorting-Batch_Analysis_28022017125550.pdf

SS RVMrx1

Tube	Status	Run Time
US	OK	2/28/17 12:55 PM
CHP	OK	2/28/17 12:55 PM
CHP_001	OK	2/28/17 12:55 PM
UT	OK	2/28/17 12:55 PM
UT_001	OK	2/28/17 12:55 PM
DTT	OK	2/28/17 12:56 PM
DTT_001	OK	2/28/17 12:56 PM
TN lib ox	OK	2/28/17 12:56 PM
TN lib ox_001	OK	2/28/17 12:56 PM
Post Sort	OK	2/28/17 12:56 PM

KA RVMrx1

Tube	Status	Run Time
CHP	OK	2/28/17 12:56 PM
CHP_001	OK	2/28/17 12:56 PM
UT	OK	2/28/17 12:56 PM
UT_001	OK	2/28/17 12:56 PM
DTT	OK	2/28/17 12:56 PM
DTT_001	OK	2/28/17 12:56 PM

PP RVMrx1_001

Tube	Status	Run Time
CHP	OK	2/28/17 12:56 PM
CHP_001	OK	2/28/17 12:56 PM
UT	OK	2/28/17 12:56 PM
UT_001	OK	2/28/17 12:56 PM
DTT	OK	2/28/17 12:56 PM
DTT_001	OK	2/28/17 12:56 PM

SC RVMrx1_002

Tube	Status	Run Time
CHP	OK	2/28/17 12:56 PM
CHP_001	OK	2/28/17 12:56 PM
UT	OK	2/28/17 12:56 PM
UT_001	OK	2/28/17 12:56 PM
DTT	OK	2/28/17 12:56 PM
DTT_001	OK	2/28/17 12:56 PM

BD FACSDiva 8.0.1

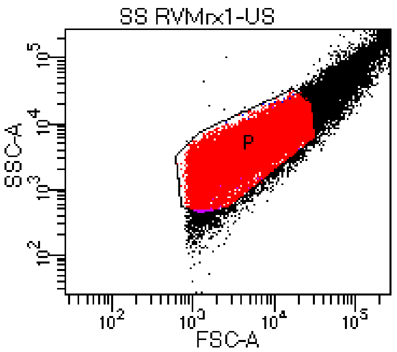

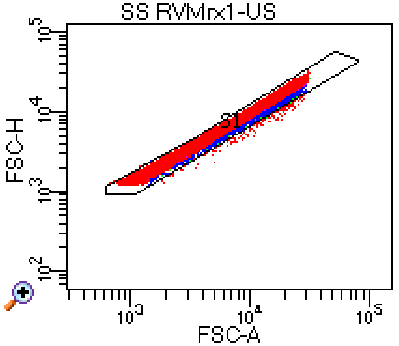

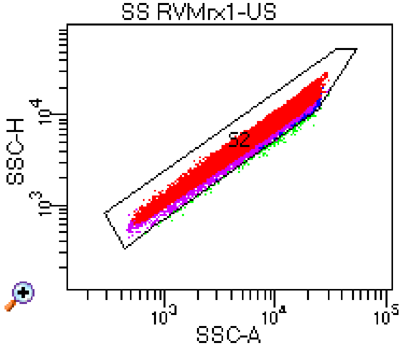

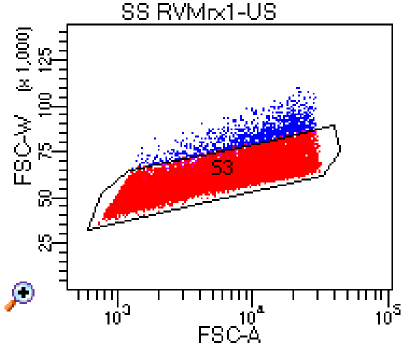

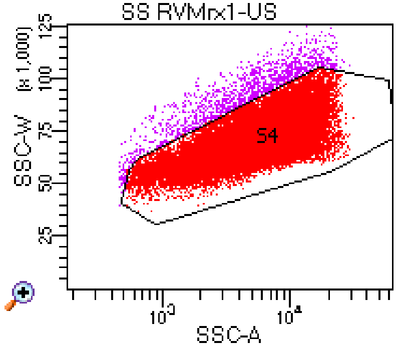

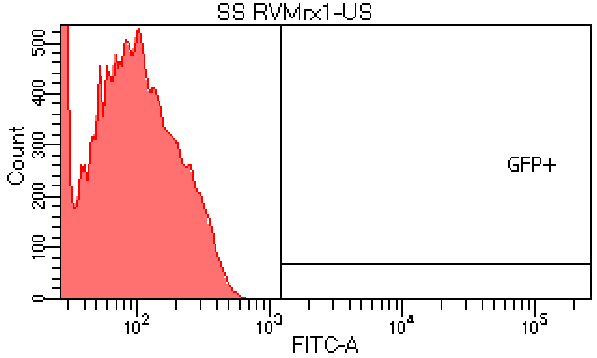

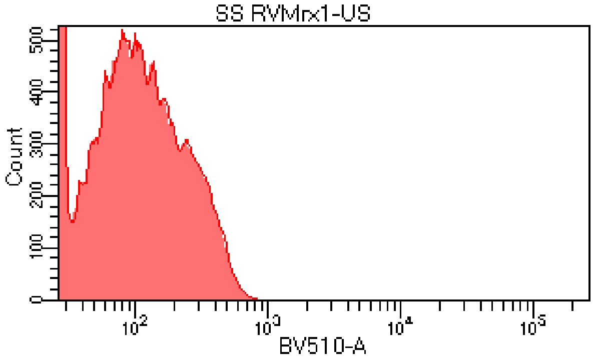

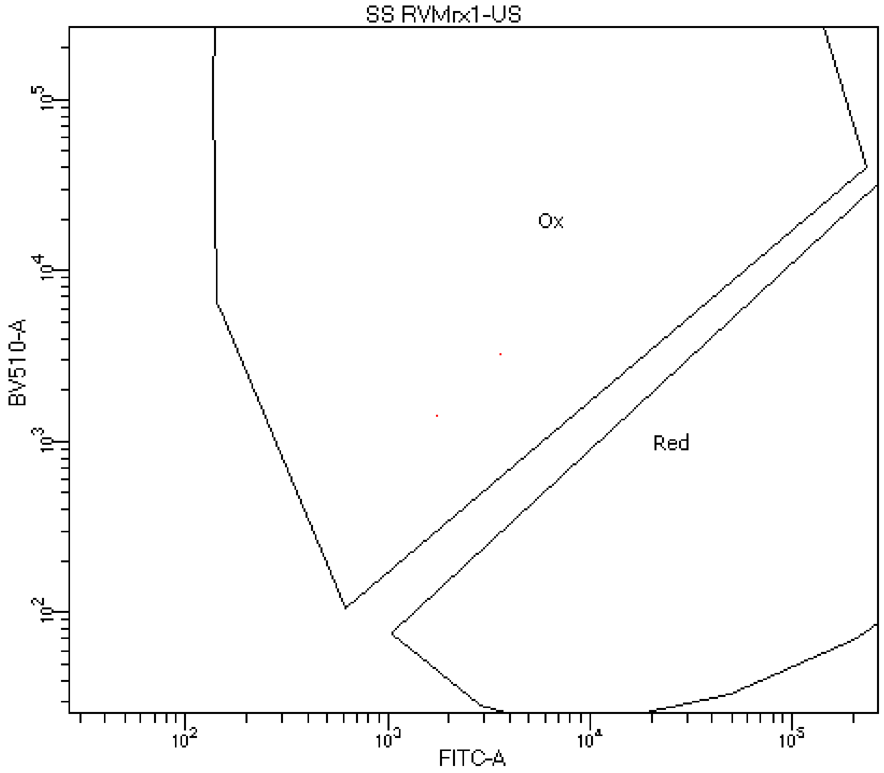

Tube: US

Population	#Events	%Parent	%Total
All Events	41,630	####	100.0
P	32,648	78.4	78.4
S1	32,439	99.4	77.9
S2	32,214	99.3	77.4
S3	31,016	96.3	74.5
S4	29,632	95.5	71.2
GFP+	2	0.0	0.0
Ox	2	100.0	0.0
Red	0	0.0	0.0

Experiment Name: 31Jan2017 Bac sorting
Specimen Name: SS RVMrx1
Tube Name: US
Record Date: Jan 31, 2017 2:44:47 PM
$OP: Administrator
GUID: 15f29c7e-7aea-4d68-8a82-41ae478...

Population	#Events	%Parent	FITC-A Median	BV510-A Median
S4	29,632	95.5	80	94
GFP+	2	0.0	2,641	2,313
Ox	2	100.0	2,641	2,313
Red	0	0.0	####	####

BD FACSDiva 8.0.1

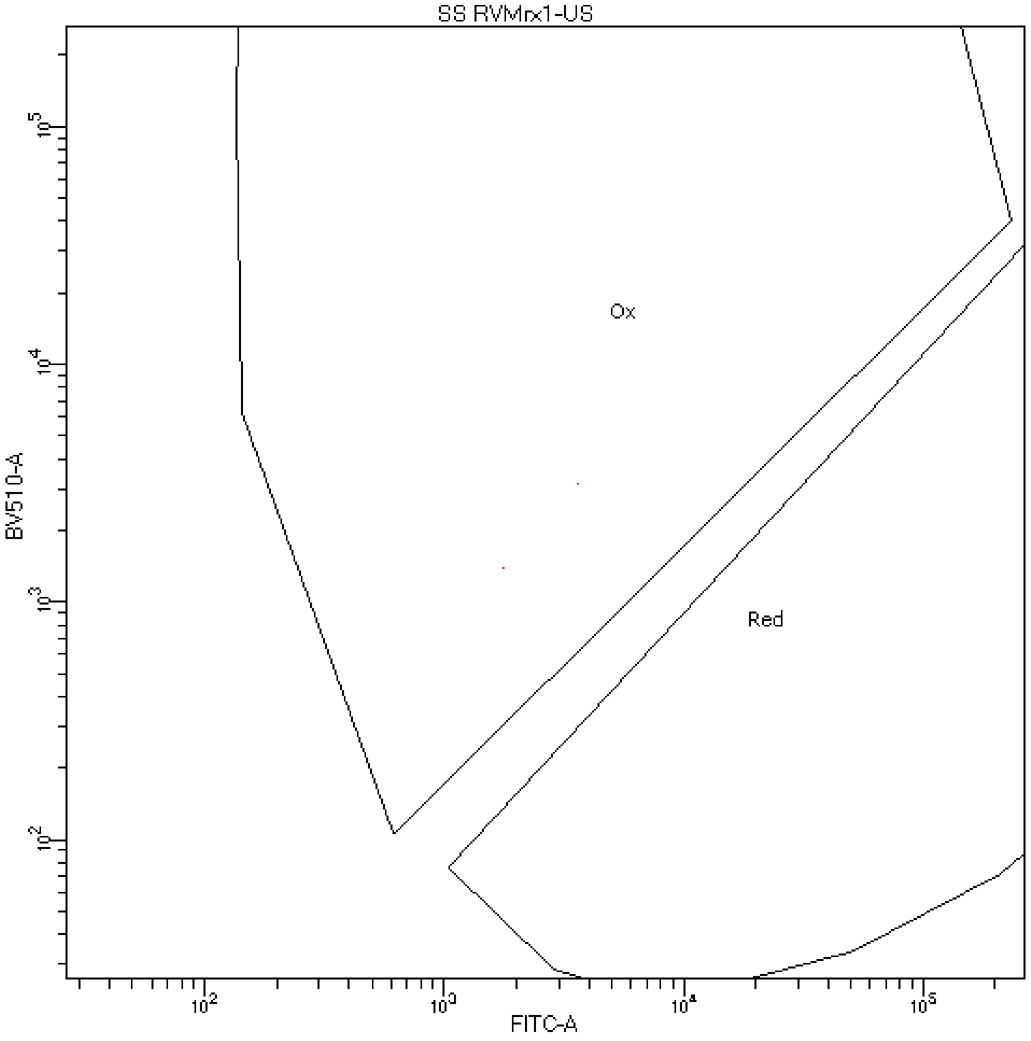

BD FACSDiva 8.0.1

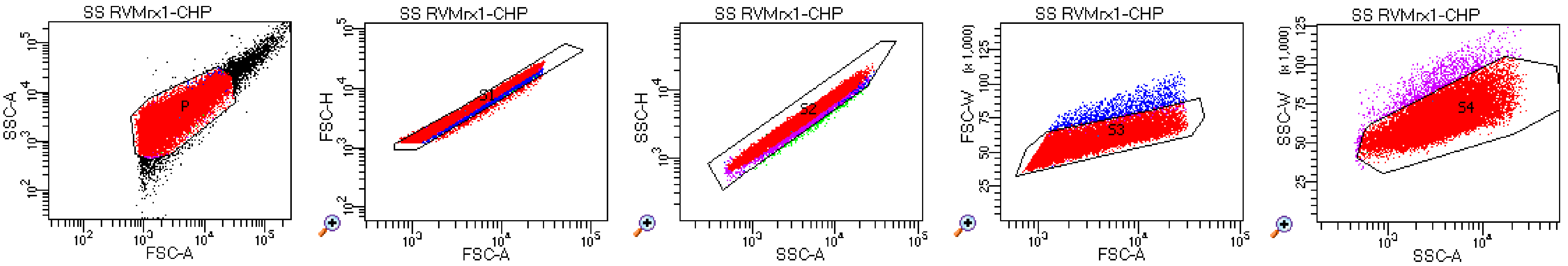

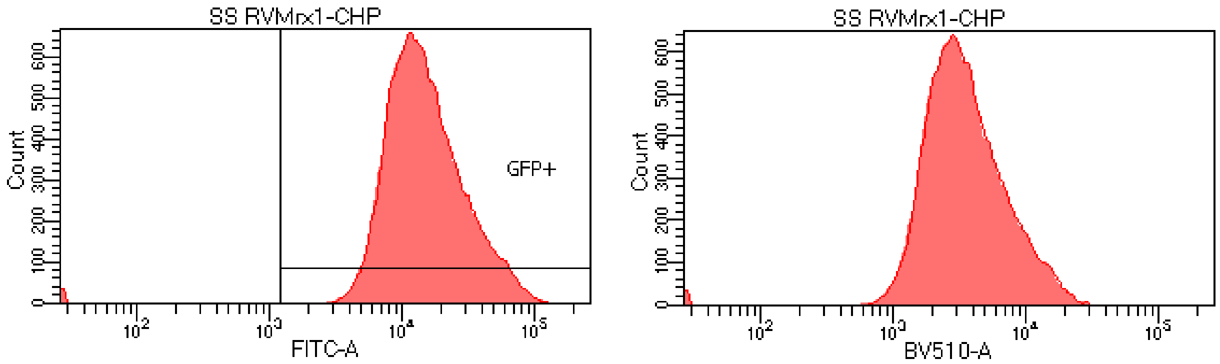

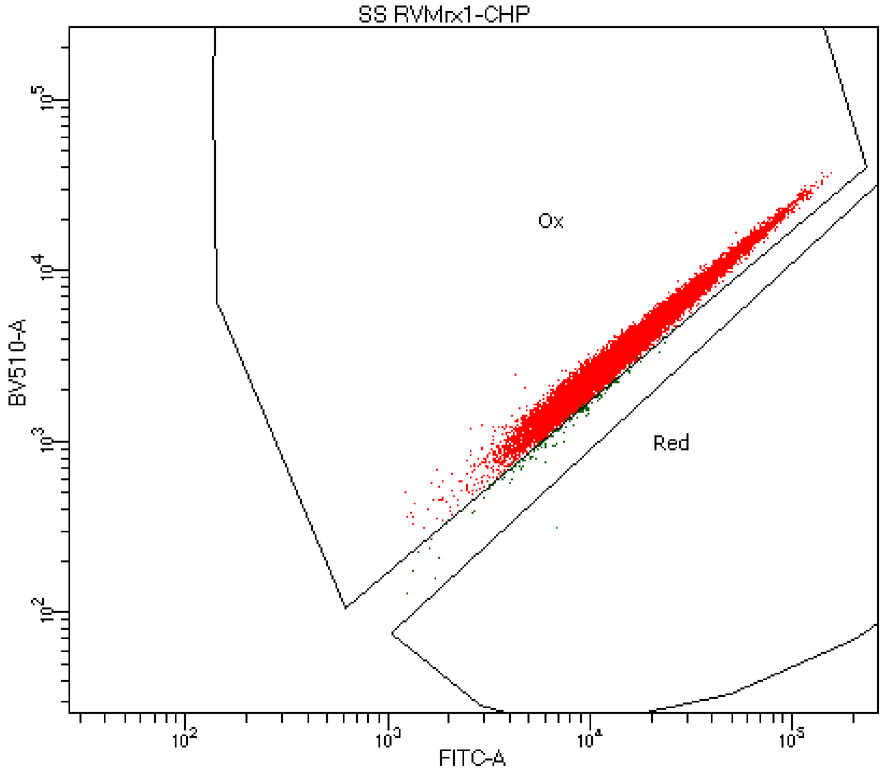

Tube: CHP

Population	#Events	%Parent	%Total
All Events	32,910	####	100.0
P	30,737	93.4	93.4
S1	30,263	98.5	92.0
S2	29,975	99.0	91.1
S3	28,940	96.5	87.9
S4	27,728	95.8	84.3
GFP+	27,559	99.4	83.7
Ox	27,341	99.2	83.1
Red	1	0.0	0.0

Experiment Name:	31Jan2017 Bac sorting
Specimen Name:	SS RVMrx1
Tube Name:	CHP
Record Date:	Jan 31, 2017 2:45:57 PM
$OP:	Administrator
GUID:	11453805-9c55-41a4-b529-29bf8d5...

Population	#Events	%Parent	FITC-A Median	BV510-A Median
S4	27,728	95.8	13,465	3,121
GFP+	27,559	99.4	13,518	3,135
Ox	27,341	99.2	13,584	3,156
Red	1	0.0	6,797	310

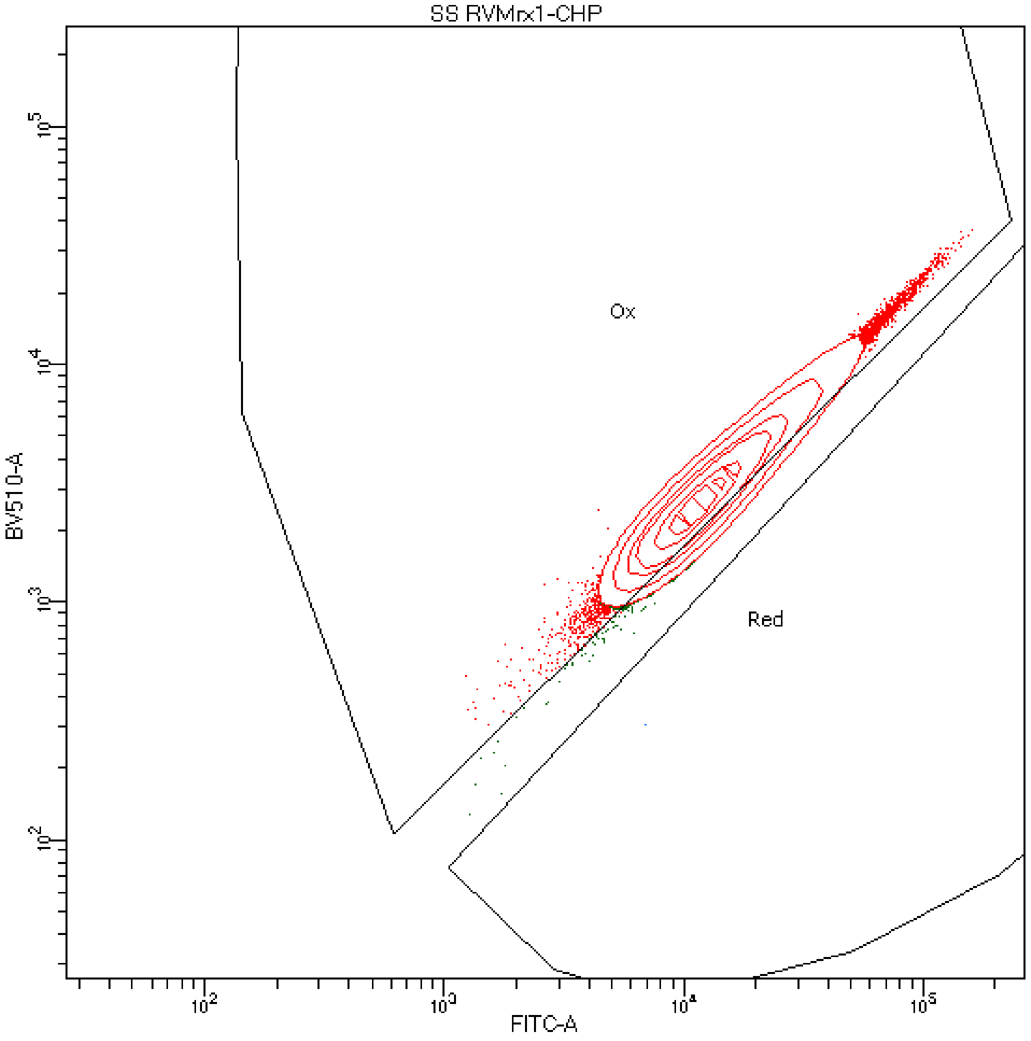
SS RVMrx1-CHP
Ox
Red
BV510-A
FITC-A
10^2
10^3
10^4
10^5

BD FACSDiva 8.0.1

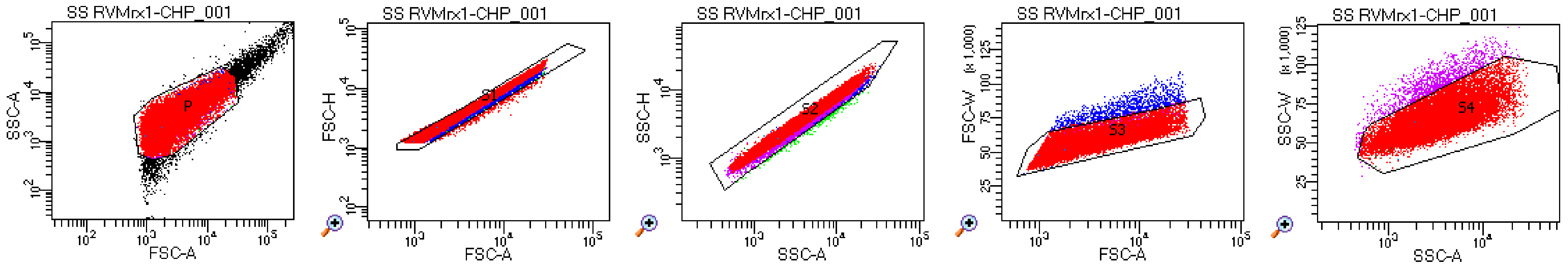

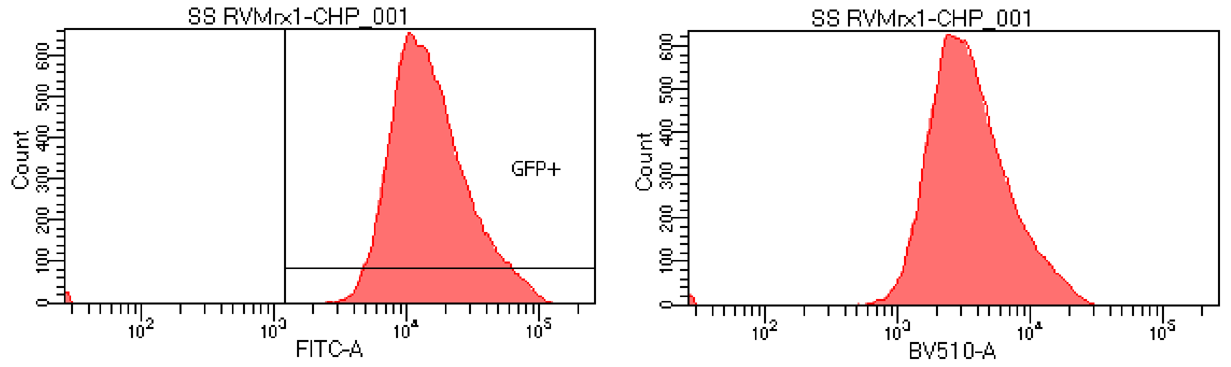

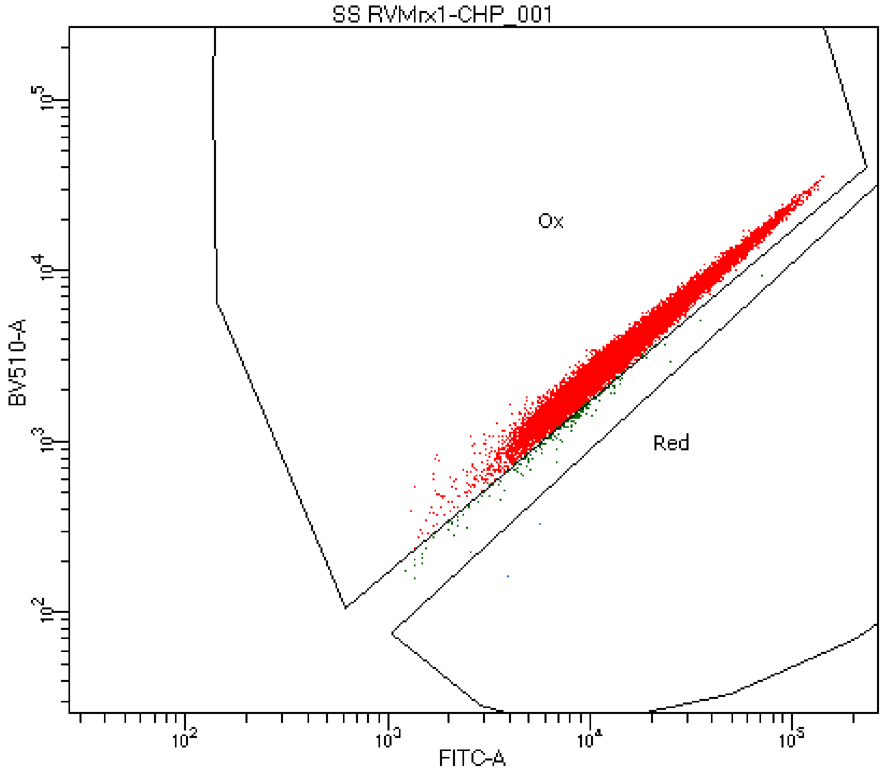

Tube: CHP_001

Population	#Events	%Parent	%Total
All Events	32,966	####	100.0
P	30,777	93.4	93.4
S1	30,317	98.5	92.0
S2	30,041	99.1	91.1
S3	28,935	96.3	87.8
S4	27,661	95.6	83.9
GFP+	27,514	99.5	83.5
Ox	27,227	99.0	82.6
Red	2	0.0	0.0

Experiment Name: 31Jan2017 Bac sorting
Specimen Name: SS RVMrx1
Tube Name: CHP_001
Record Date: Jan 31, 2017 2:46:30 PM
$OP: Administrator
GUID: 91490226-8fc7-4d50-b4ed-66faae9...

Population	#Events	%Parent	FITC-A Median	BV510-A Median
S4	27,661	95.6	13,494	3,131
GFP+	27,514	99.5	13,548	3,144
Ox	27,227	99.0	13,630	3,163
Red	2	0.0	4,784	245

BD FACSDiva 8.0.1

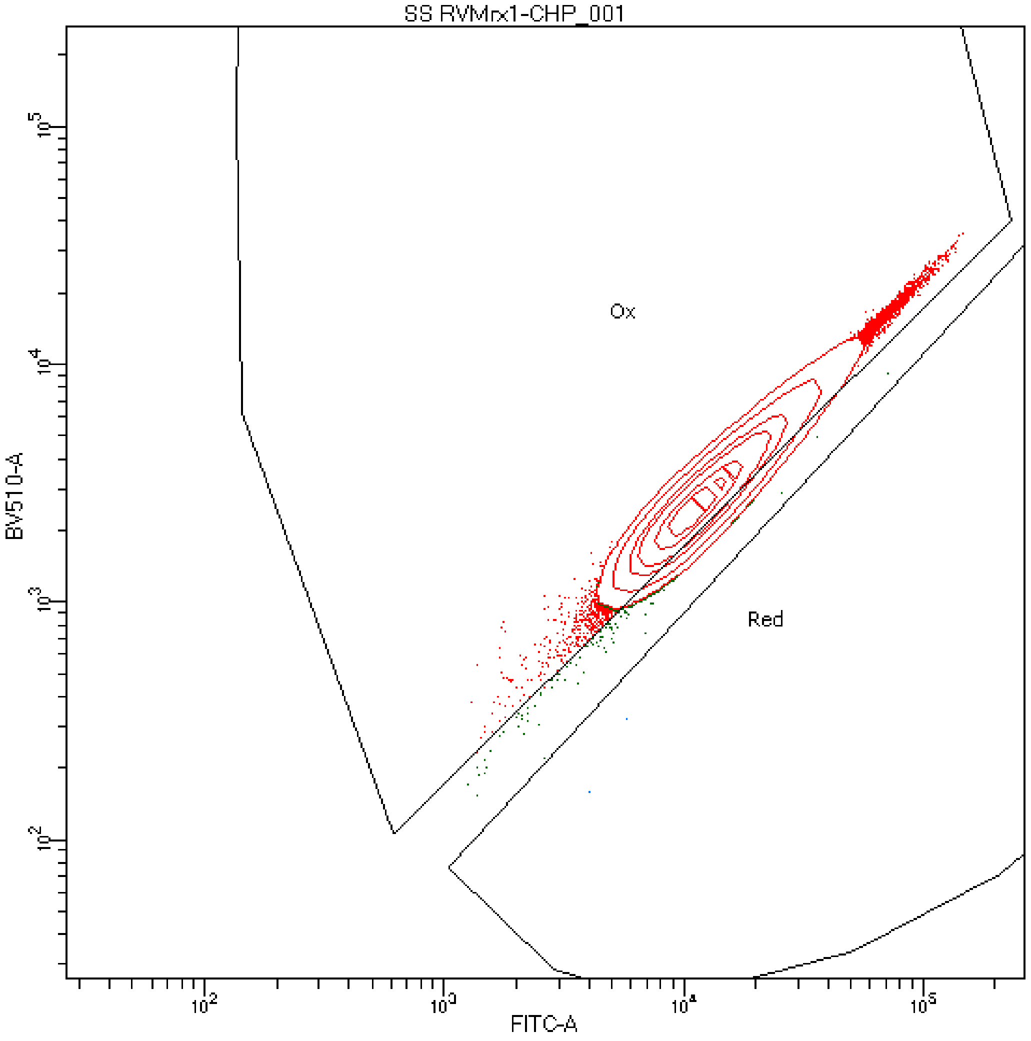

BD FACSDiva 8.0.1

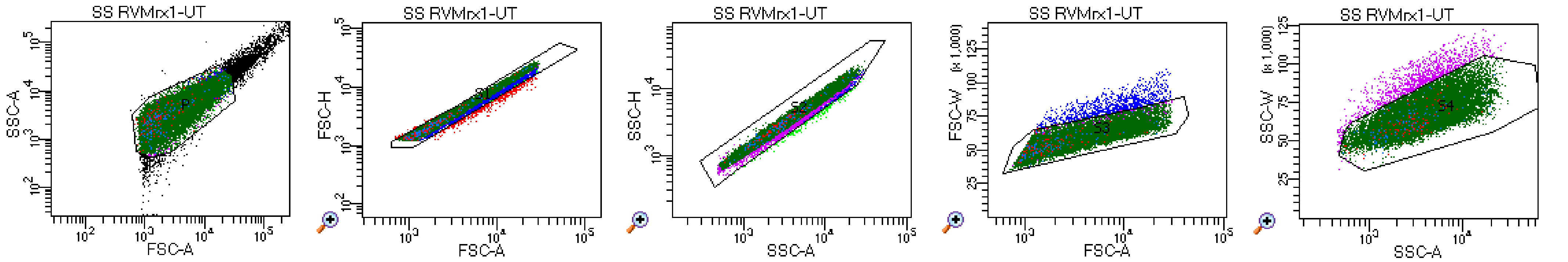

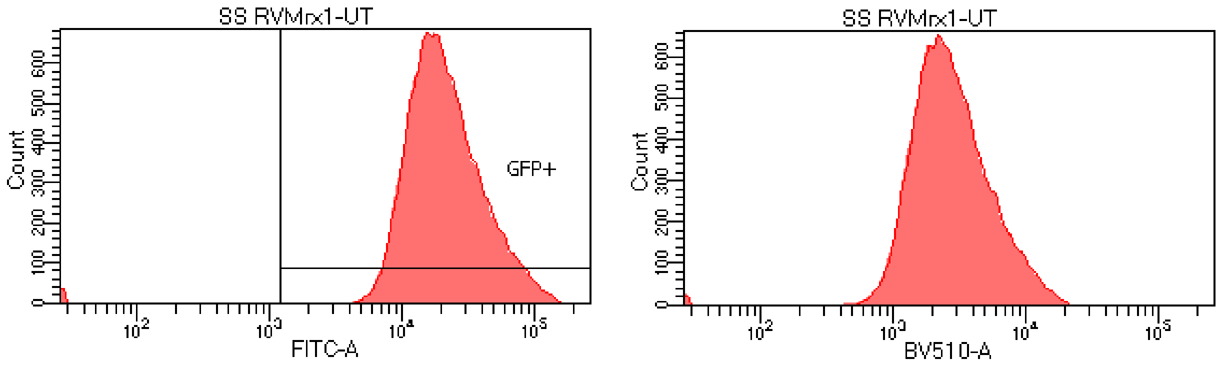

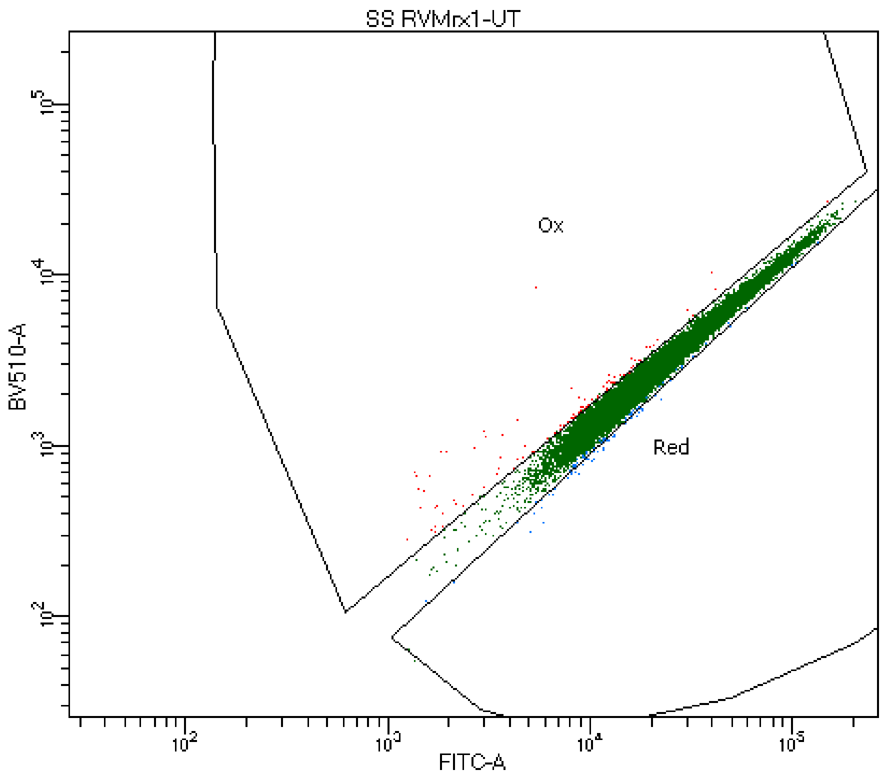

Tube: UT

Population	#Events	%Parent	%Total
All Events	32,742	####	100.0
P	30,702	93.8	93.8
S1	30,201	98.4	92.2
S2	29,905	99.0	91.3
S3	28,966	96.9	88.5
S4	27,872	96.2	85.1
GFP+	27,716	99.4	84.6
Ox	93	0.3	0.3
Red	107	0.4	0.3

Experiment Name:	31Jan2017 Bac sorting
Specimen Name:	SS RVMrx1
Tube Name:	UT
Record Date:	Jan 31, 2017 2:47:21 PM
$OP:	Administrator
GUID:	8dd4070b-59cc-423c-a32e-18b687...

Population	#Events	%Parent	FITC-A Median	BV510-A Median
S4	27,872	96.2	19,021	2,474
GFP+	27,716	99.4	19,102	2,484
Ox	93	0.3	8,456	1,615
Red	107	0.4	11,683	1,086

BD FACSDiva 8.0.1

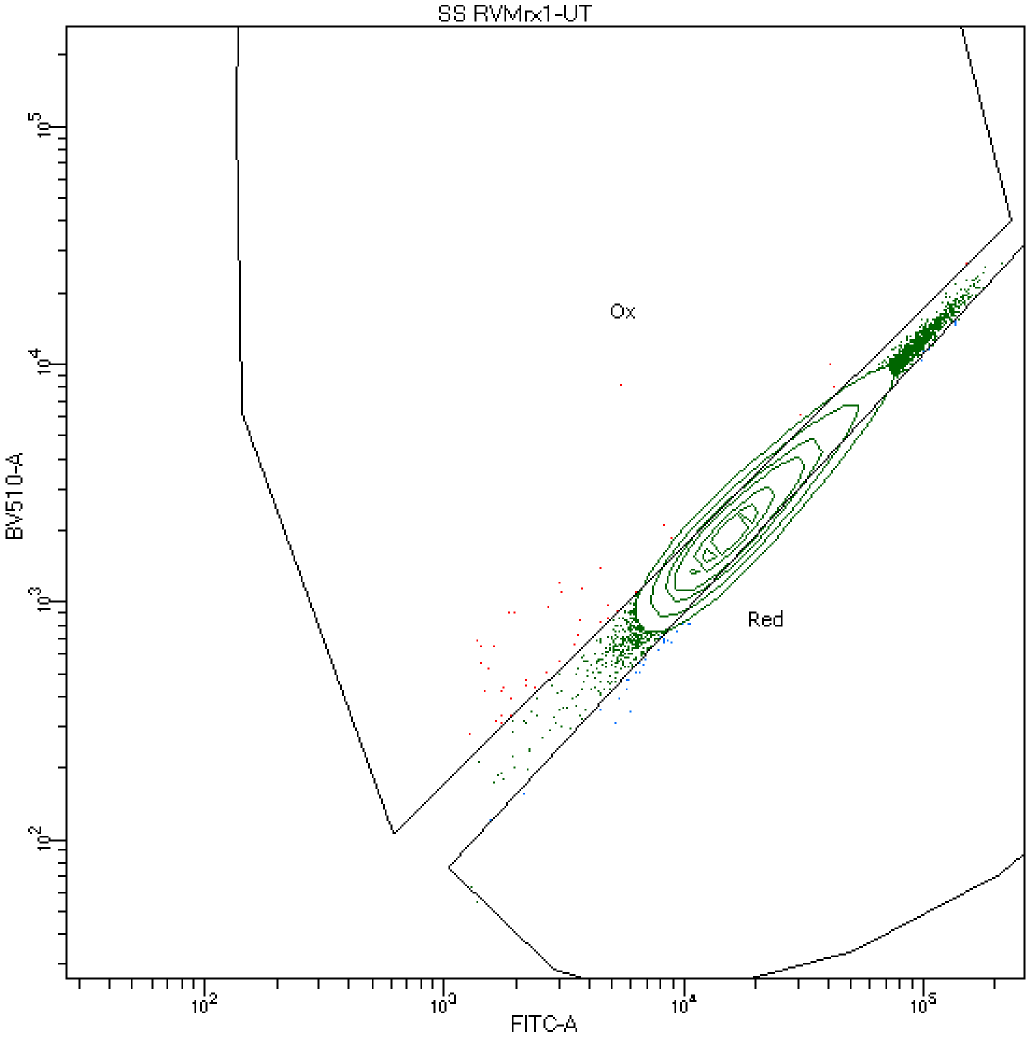

BD FACSDiva 8.0.1

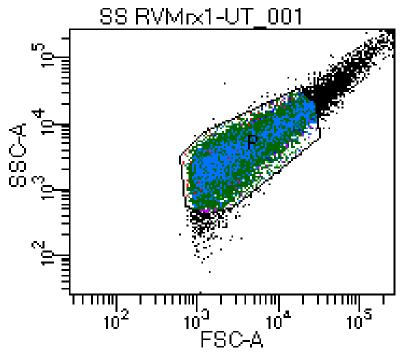

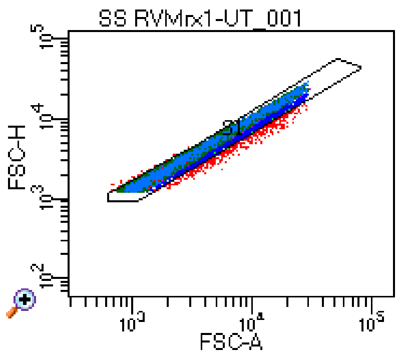

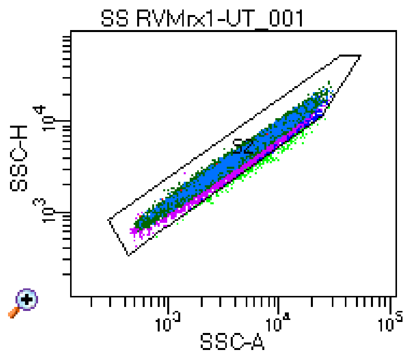

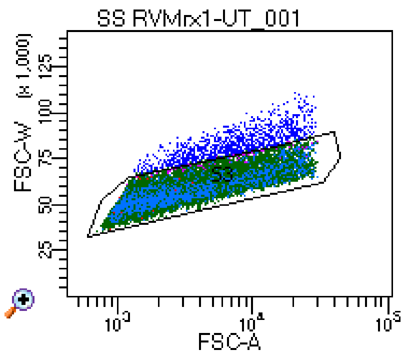

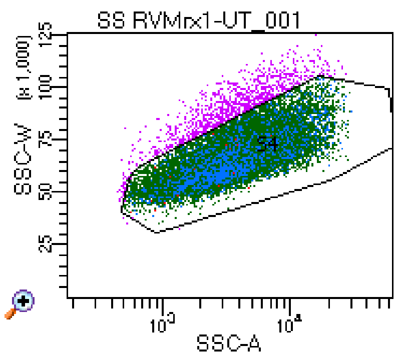

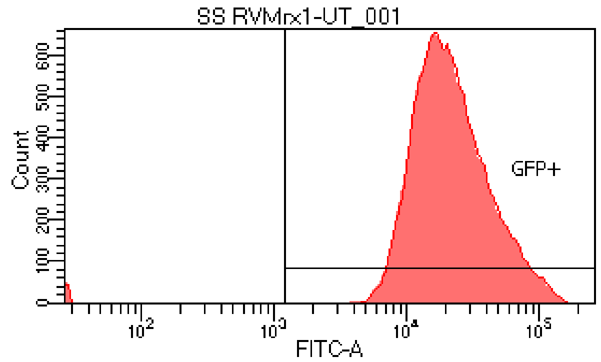

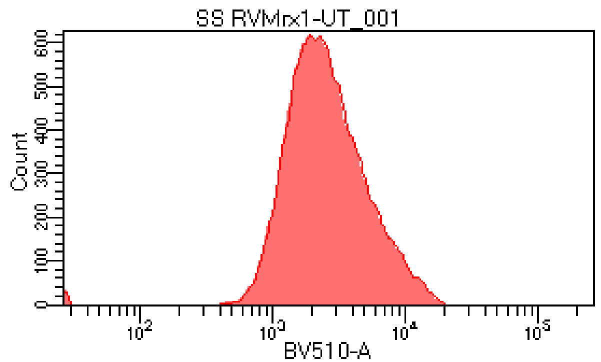

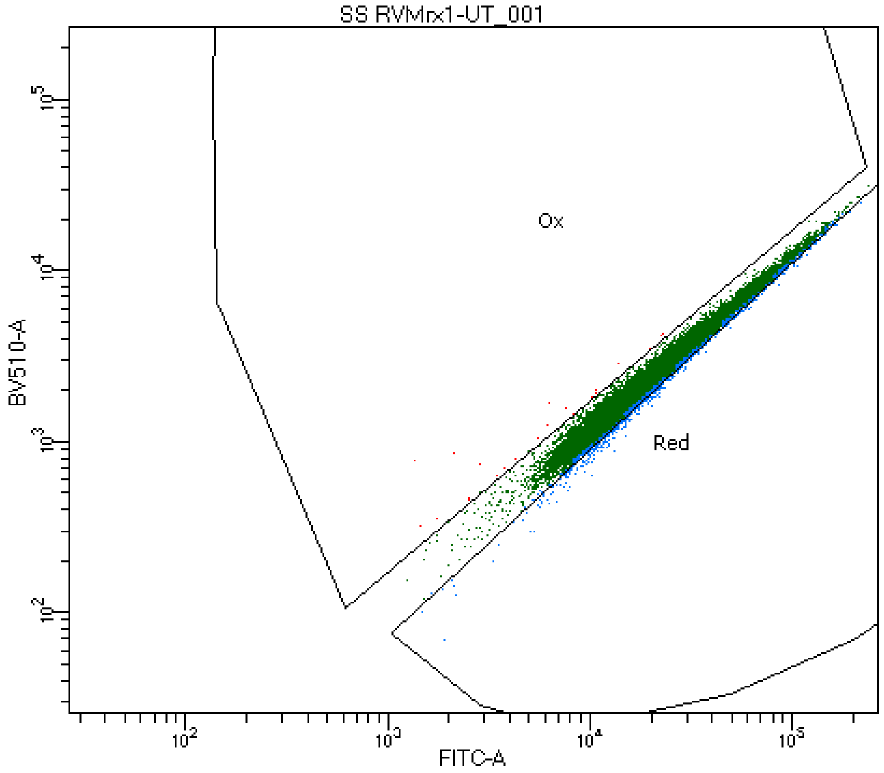

Tube: UT_001

Population	#Events	%Parent	%Total
All Events	32,775	####	100.0
P	30,749	93.8	93.8
S1	30,217	98.3	92.2
S2	29,921	99.0	91.3
S3	28,886	96.5	88.1
S4	27,816	96.3	84.9
GFP+	27,629	99.3	84.3
Ox	24	0.1	0.1
Red	1,323	4.8	4.0

Experiment Name: 31Jan2017 Bac sorting
Specimen Name: SS RVMrx1
Tube Name: UT_001
Record Date: Jan 31, 2017 2:47:54 PM
$OP: Administrator
GUID: 842b0e0b-1f86-4b7a-876a-f338811...

Population	#Events	%Parent	FITC-A Median	BV510-A Median
S4	27,816	96.3	19,580	2,294
GFP+	27,629	99.3	19,673	2,307
Ox	24	0.1	4,851	952
Red	1,323	4.8	20,869	1,999

BD FACSDiva 8.0.1

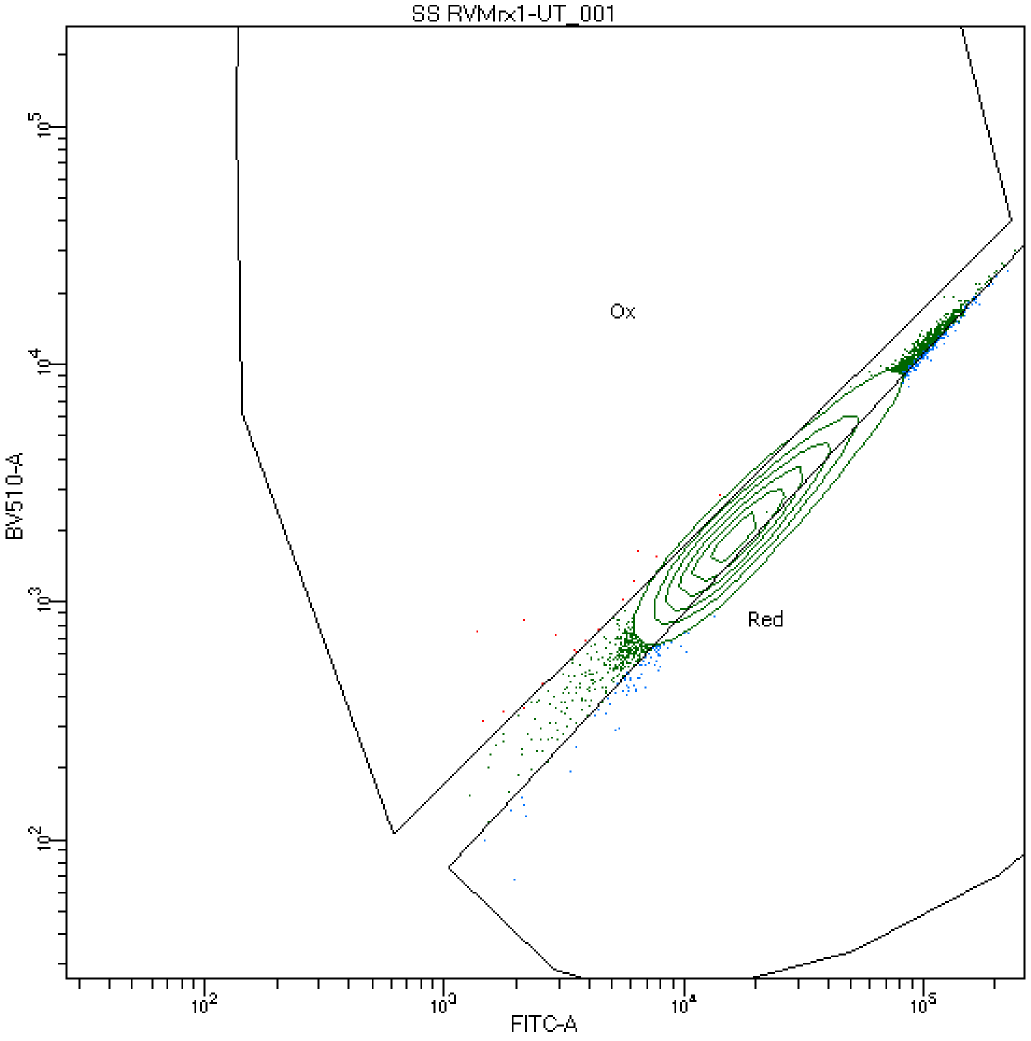

BD FACSDiva 8.0.1

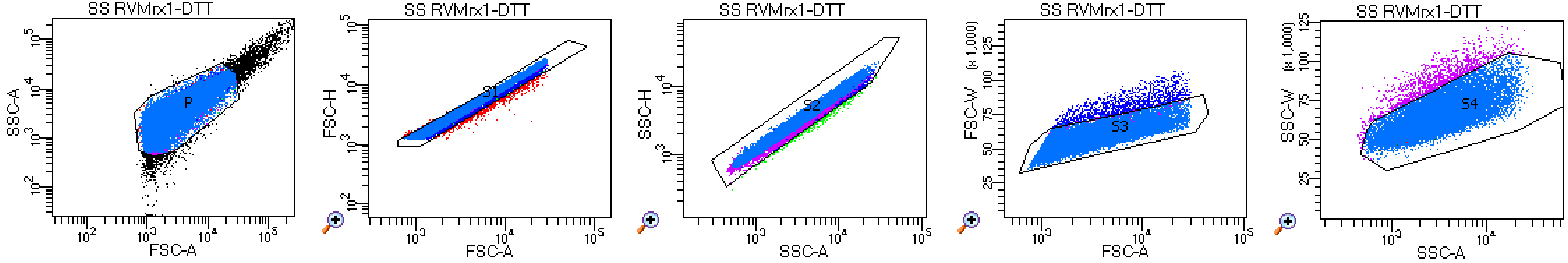

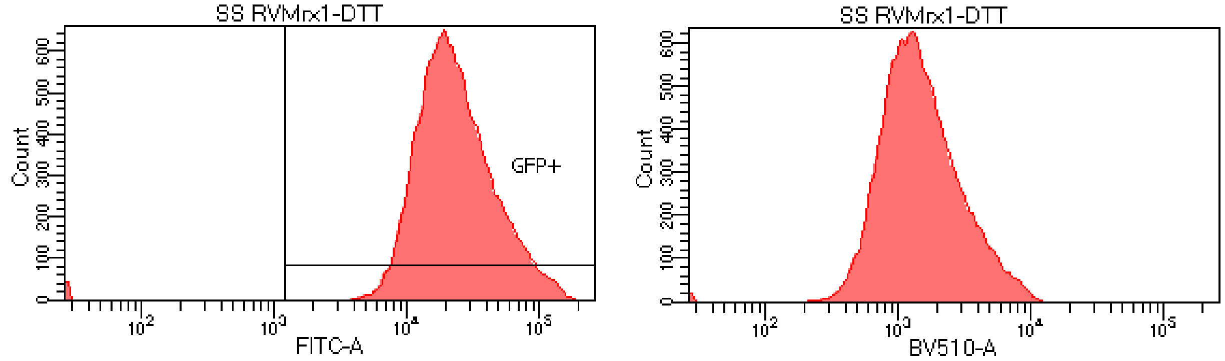

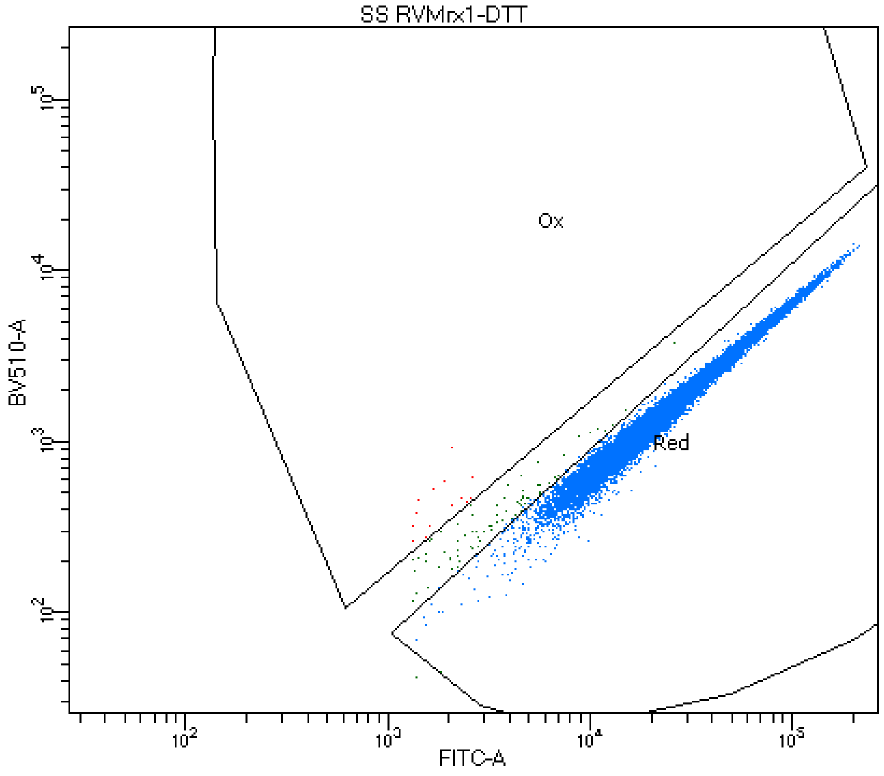

Tube: DTT

Population	#Events	%Parent	%Total
All Events	33,023	####	100.0
P	30,824	93.3	93.3
S1	30,162	97.9	91.3
S2	29,844	98.9	90.4
S3	28,578	95.8	86.5
S4	27,292	95.5	82.6
GFP+	27,124	99.4	82.1
Ox	14	0.1	0.0
Red	27,027	99.6	81.8

Experiment Name: 31Jan2017 Bac sorting
Specimen Name: SS RVMrx1
Tube Name: DTT
Record Date: Jan 31, 2017 2:48:27 PM
$OP: Administrator
GUID: 52e971b1-d71a-4596-8f36-63f7591...

Population	#Events	%Parent	FITC-A Median	BV510-A Median
S4	27,292	95.5	21,125	1,323
GFP+	27,124	99.4	21,215	1,330
Ox	14	0.1	1,767	451
Red	27,027	99.6	21,270	1,334

BD FACSDiva 8.0.1

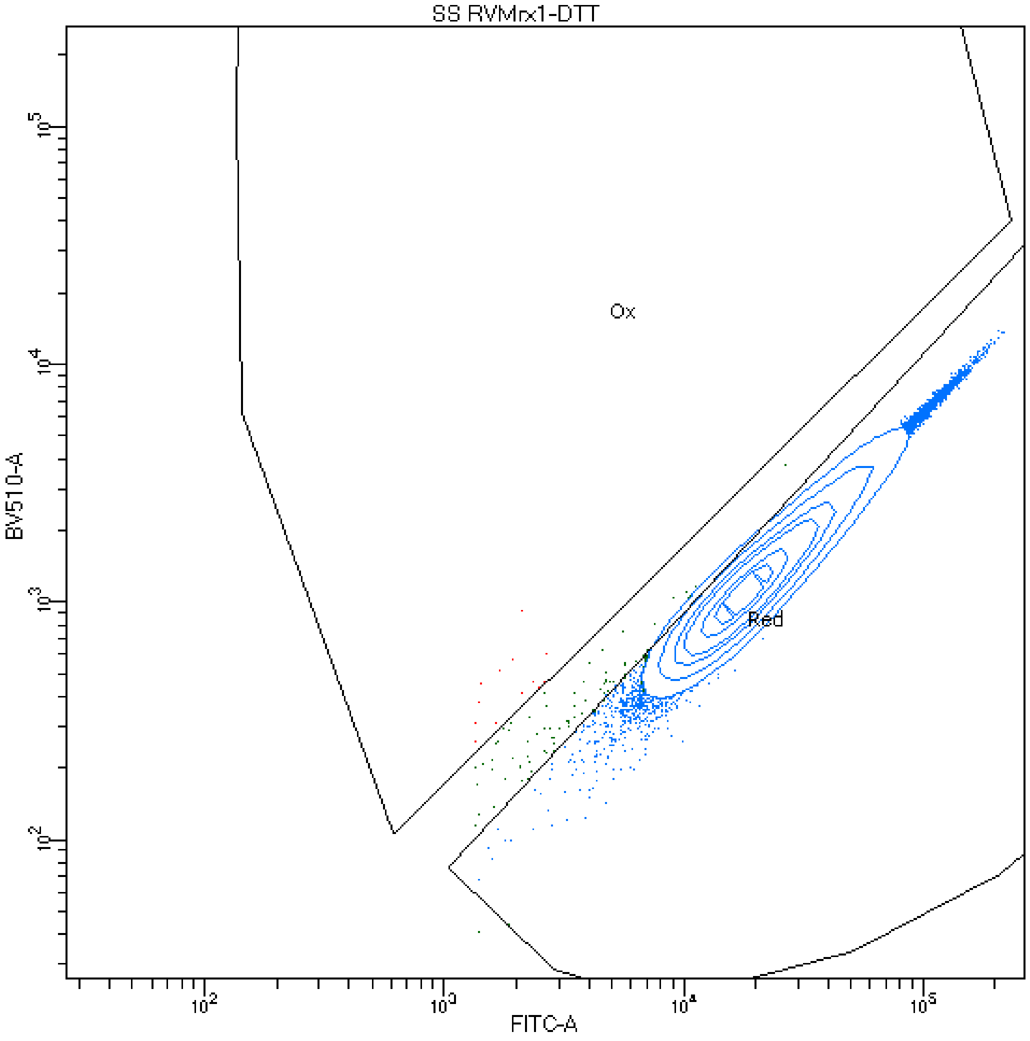

BD FACSDiva 8.0.1

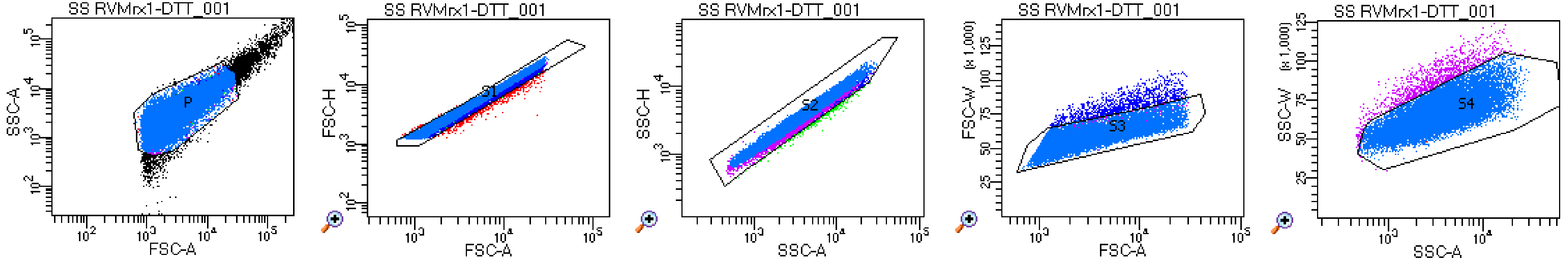

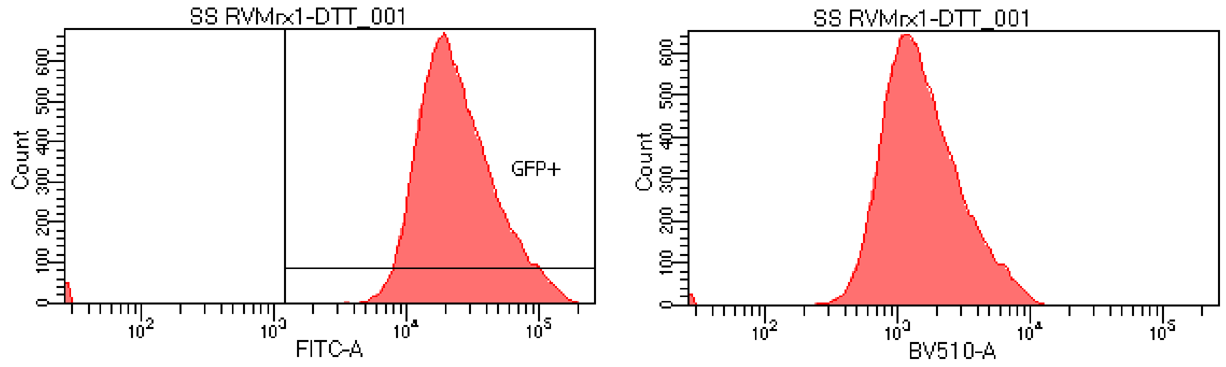

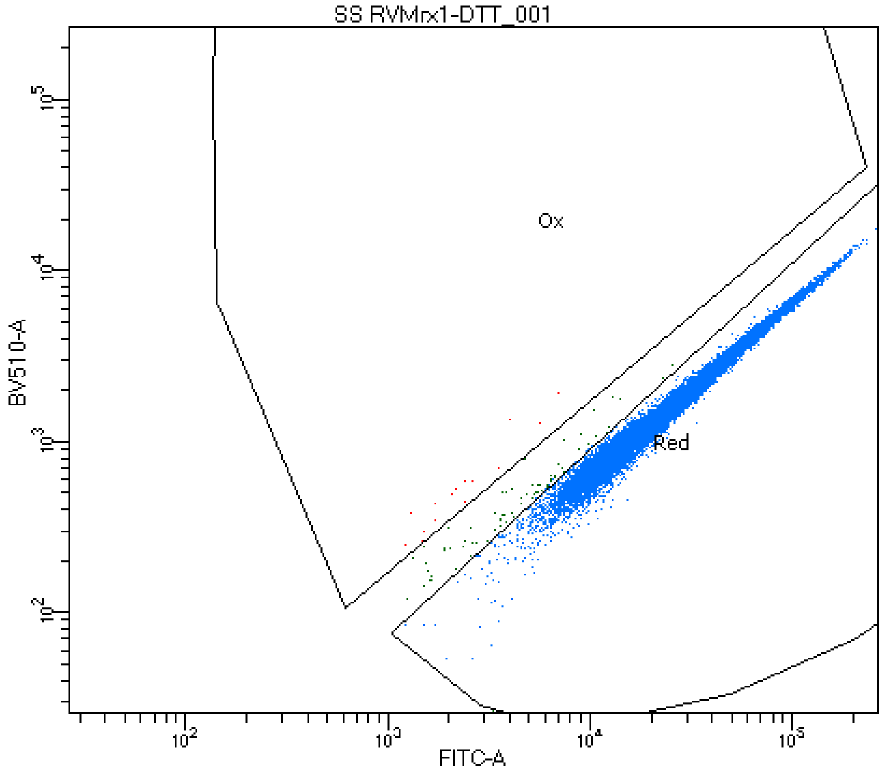

Tube: DTT_001

Population	#Events	%Parent	%Total
All Events	33,041	####	100.0
P	30,818	93.3	93.3
S1	30,274	98.2	91.6
S2	29,981	99.0	90.7
S3	28,801	96.1	87.2
S4	27,684	96.1	83.8
GFP+	27,500	99.3	83.2
Ox	15	0.1	0.0
Red	27,415	99.7	83.0

Experiment Name: 31Jan2017 Bac sorting
Specimen Name: SS RVMrx1
Tube Name: DTT_001
Record Date: Jan 31, 2017 2:48:57 PM
$OP: Administrator
GUID: d0a2f93b-f57c-4b21-87bd-8f67cb40...

Population	#Events	%Parent	FITC-A Median	BV510-A Median
S4	27,684	96.1	21,422	1,350
GFP+	27,500	99.3	21,540	1,357
Ox	15	0.1	2,153	492
Red	27,415	99.7	21,602	1,359

BD FACSDiva 8.0.1

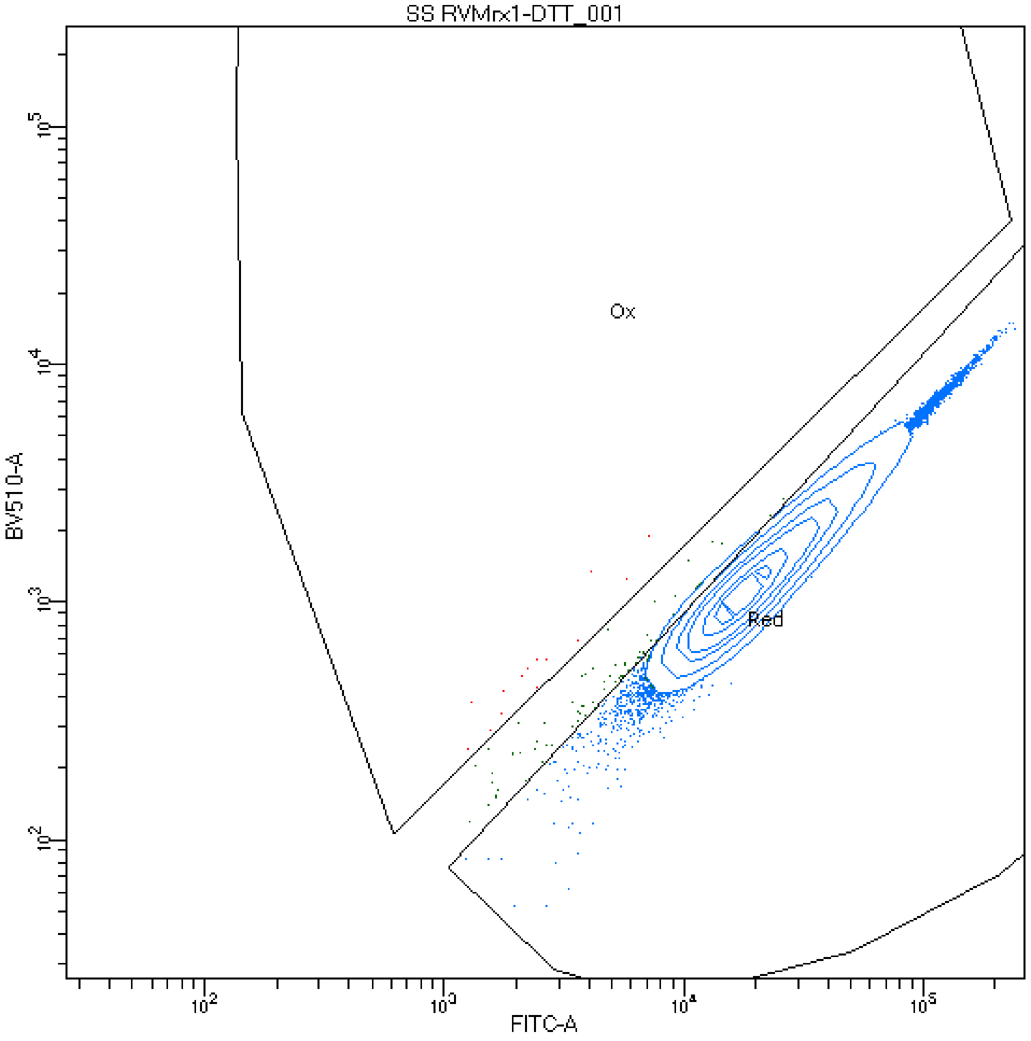

BD FACSDiva 8.0.1

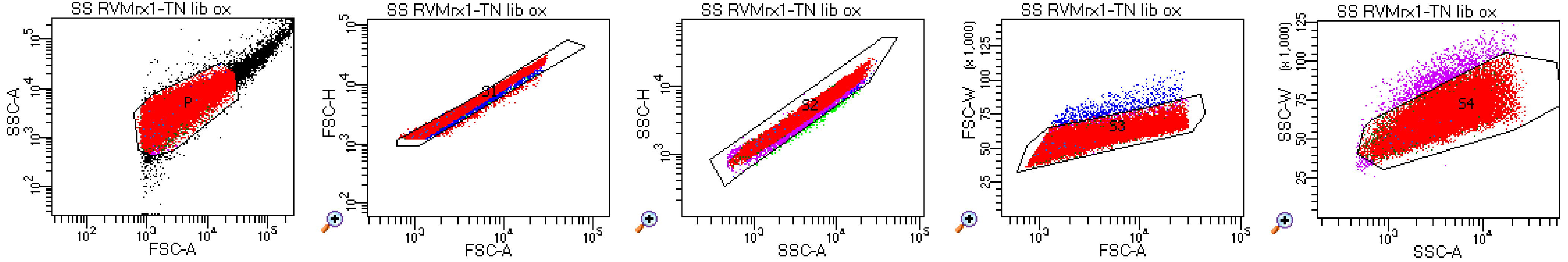

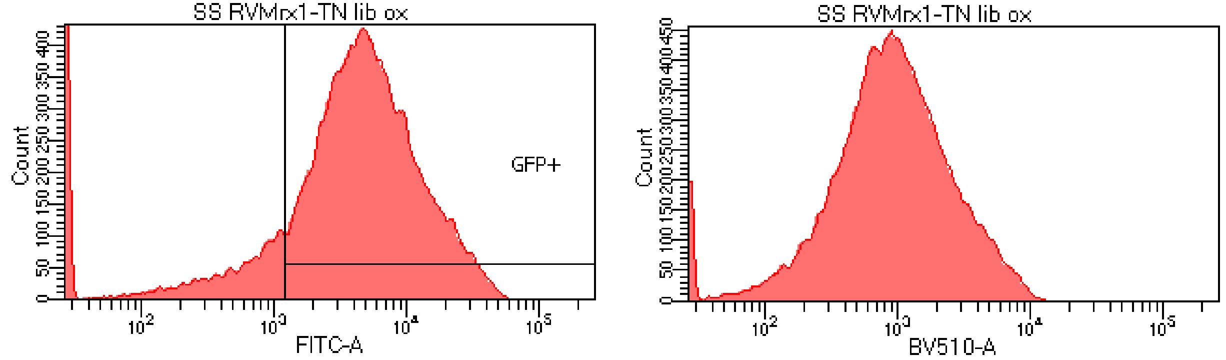

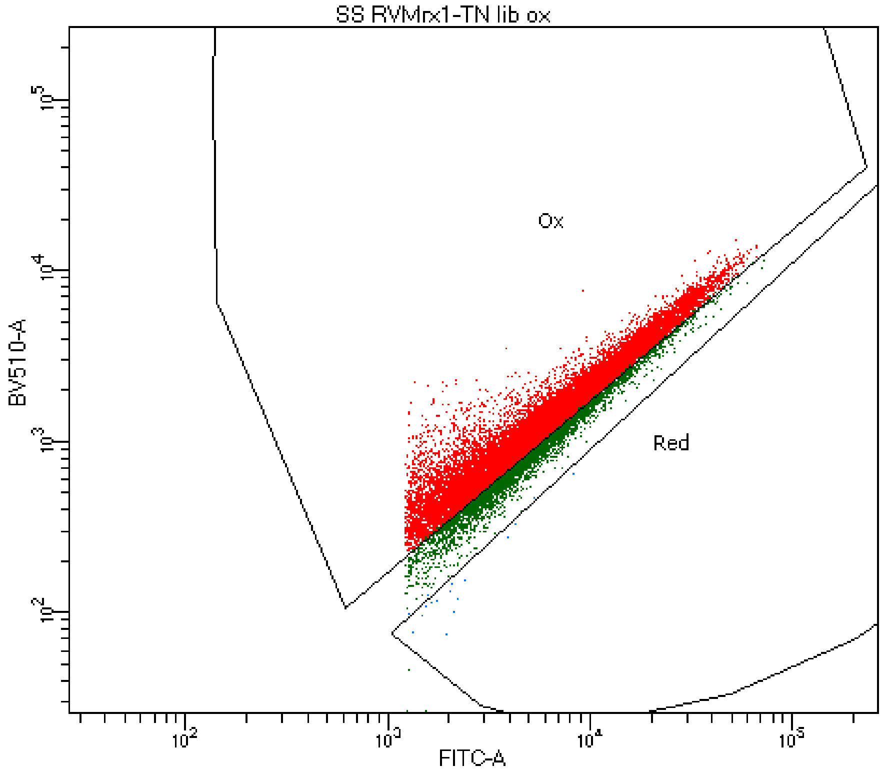

Tube: TN lib ox

Population	#Events	%Parent	%Total
All Events	33,732	####	100.0
P	30,876	91.5	91.5
S1	30,526	98.9	90.5
S2	30,290	99.2	89.8
S3	29,646	97.9	87.9
S4	28,309	95.5	83.9
GFP+	23,438	82.8	69.5
Ox	18,686	79.7	55.4
Red	18	0.1	0.1

Experiment Name: 31Jan2017 Bac sorting
Specimen Name: SS RVMrx1
Tube Name: TN lib ox
Record Date: Jan 31, 2017 3:04:03 PM
$OP: Administrator
GUID: 17fe9740-a53c-4ba2-8c74-0f2db07...

Population	#Events	%Parent	FITC-A Median	BV510-A Median
S4	28,309	95.5	4,100	863
GFP+	23,438	82.8	5,032	1,052
Ox	18,686	79.7	5,123	1,142
Red	18	0.1	1,948	118

BD FACSDiva 8.0.1

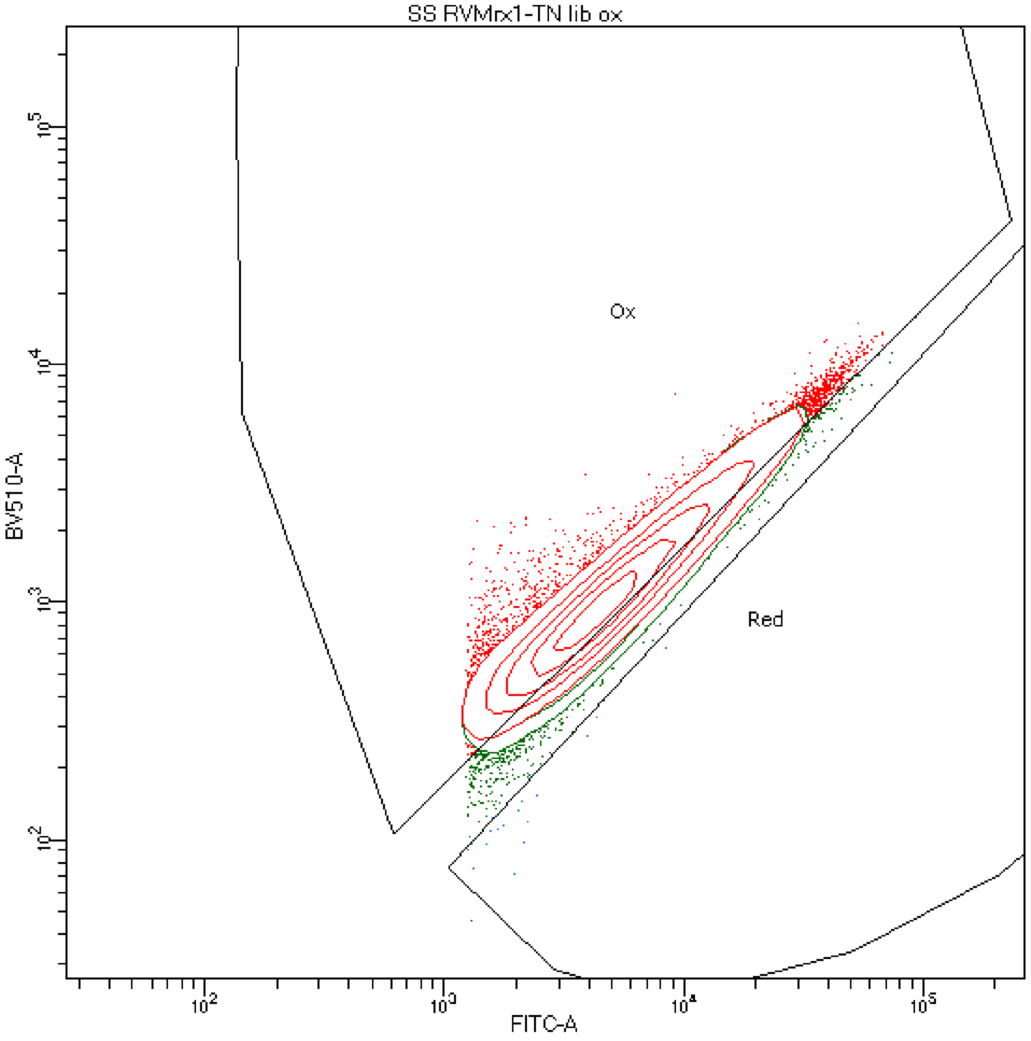

BD FACSDiva 8.0.1

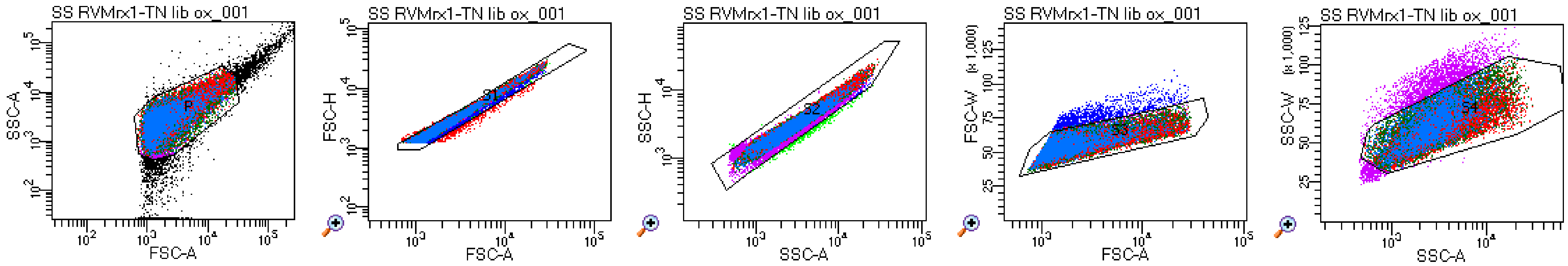

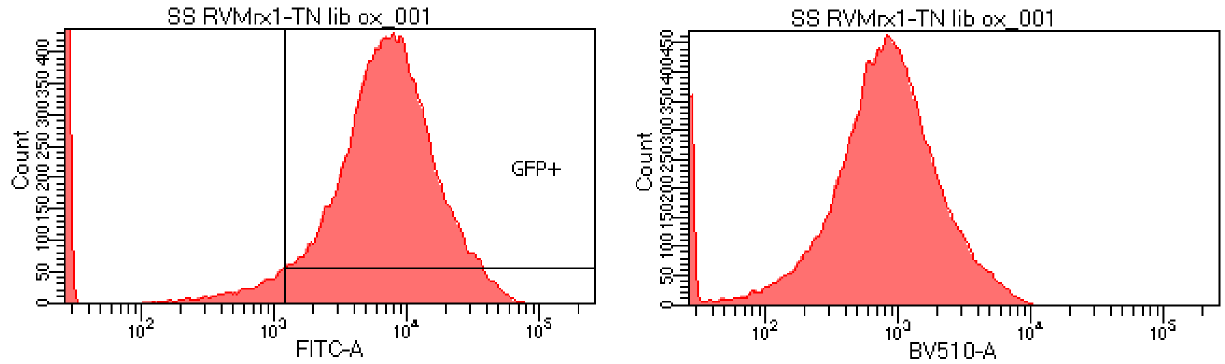

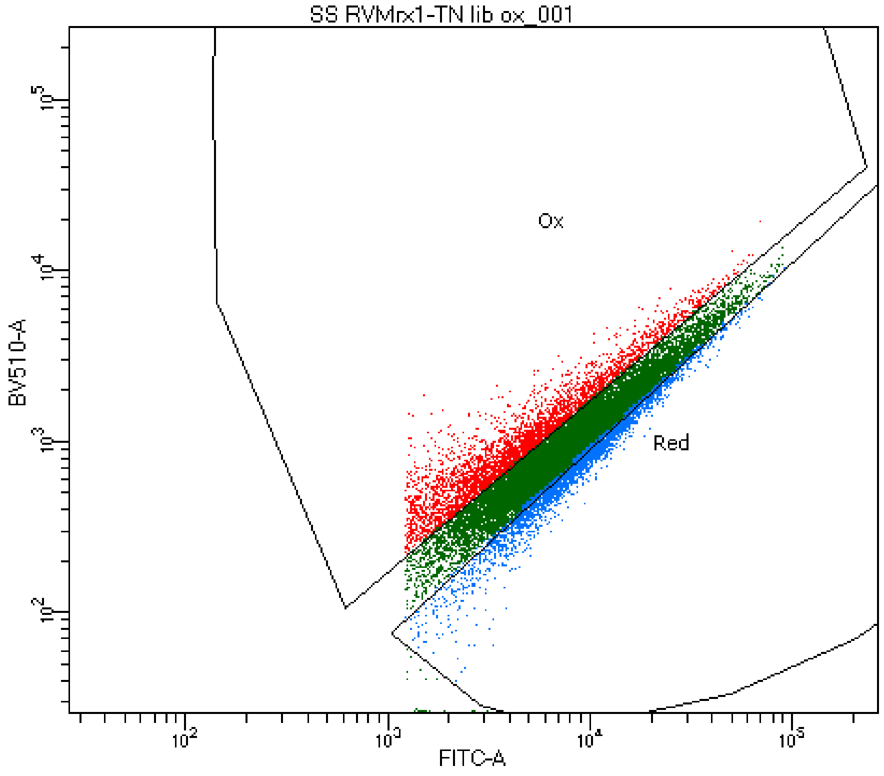

Tube: TN lib ox_001

Population	#Events	%Parent	%Total
All Events	33,226	####	100.0
P	30,691	92.4	92.4
S1	30,173	98.3	90.8
S2	29,700	98.4	89.4
S3	28,530	96.1	85.9
S4	25,936	90.9	78.1
GFP+	22,462	86.6	67.6
Ox	3,446	15.3	10.4
Red	3,465	15.4	10.4

Experiment Name:	31Jan2017 Bac sorting
Specimen Name:	SS RVMrx1
Tube Name:	TN lib ox_001
Record Date:	Jan 31, 2017 3:04:43 PM
$OP:	Administrator
GUID:	2c888717-4ed2-491e-a1d0-725760...

Population	#Events	%Parent	FITC-A Median	BV510-A Median
S4	25,936	90.9	6,237	772
GFP+	22,462	86.6	7,221	882
Ox	3,446	15.3	4,149	928
Red	3,465	15.4	8,672	713

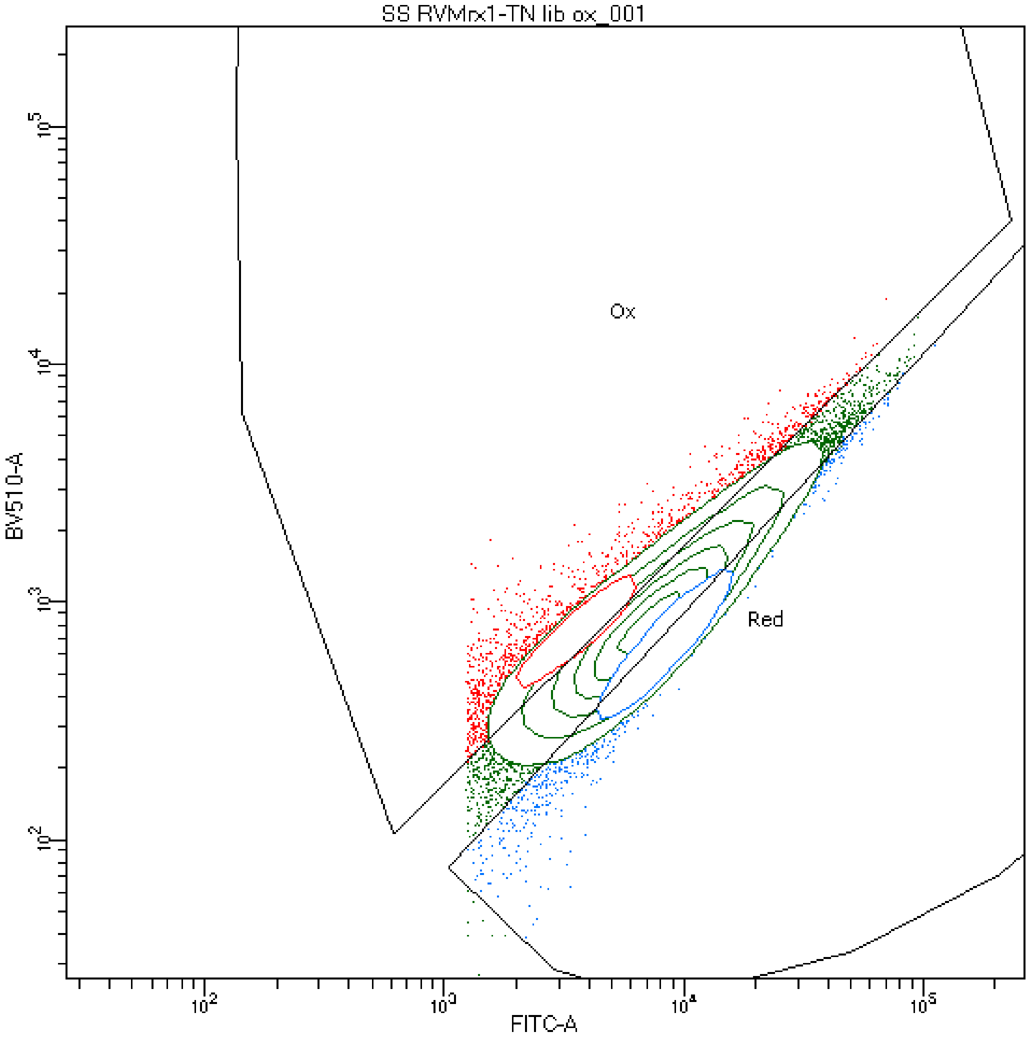
SS RVMrx1-TN lib ox_001
Ox
Red
BV510-A
FITC-A
10^2
10^3
10^4
10^5

BD FACSDiva 8.0.1

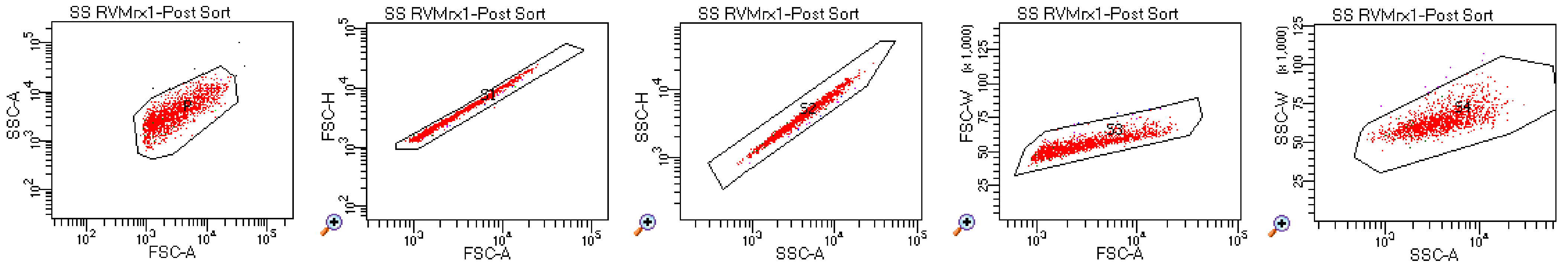

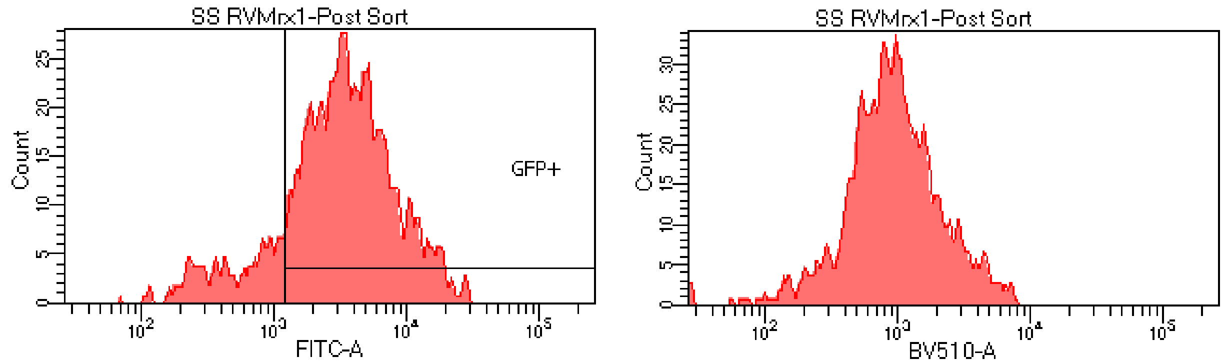

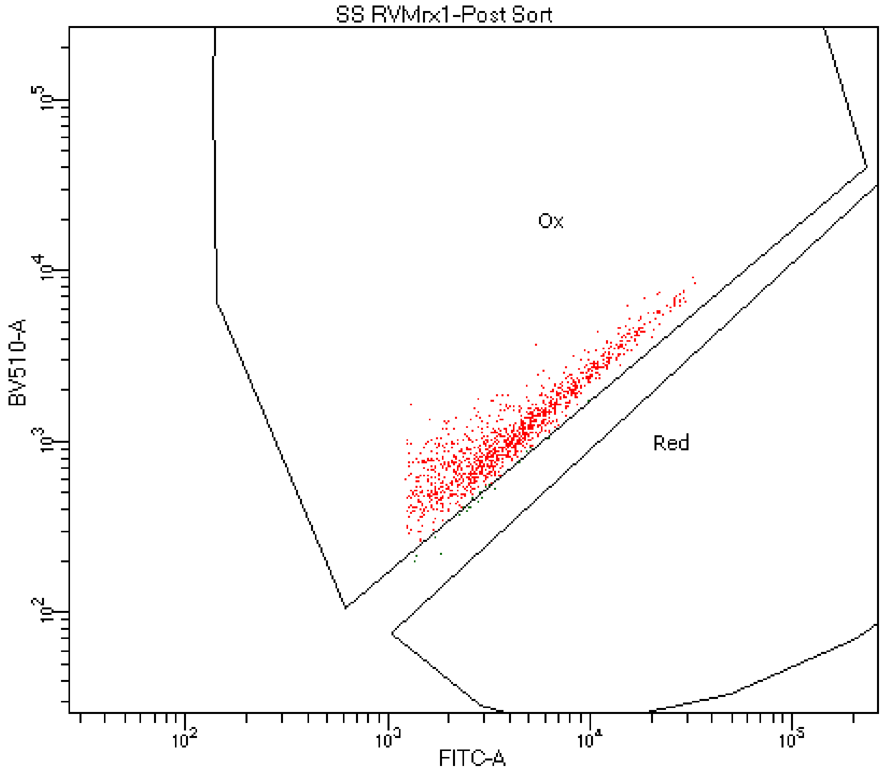

Tube: Post Sort

Population	#Events	%Parent	%Total
All Events	1,583	####	100.0
P	1,575	99.5	99.5
S1	1,574	99.9	99.4
S2	1,573	99.9	99.4
S3	1,570	99.8	99.2
S4	1,561	99.4	98.6
GFP+	1,273	81.6	80.4
Ox	1,243	97.6	78.5
Red	0	0.0	0.0

Experiment Name: 31Jan2017 Bac sorting
Specimen Name: SS RVMrx1
Tube Name: Post Sort
Record Date: Jan 31, 2017 4:54:21 PM
$OP: Administrator
GUID: d6a42b14-f70c-4d10-9030-9a749c8...

Population	#Events	%Parent	FITC-A Median	BV510-A Median
S4	1,561	99.4	3,073	868
GFP+	1,273	81.6	3,778	1,009
Ox	1,243	97.6	3,811	1,019
Red	0	0.0	####	####

BD FACSDiva 8.0.1

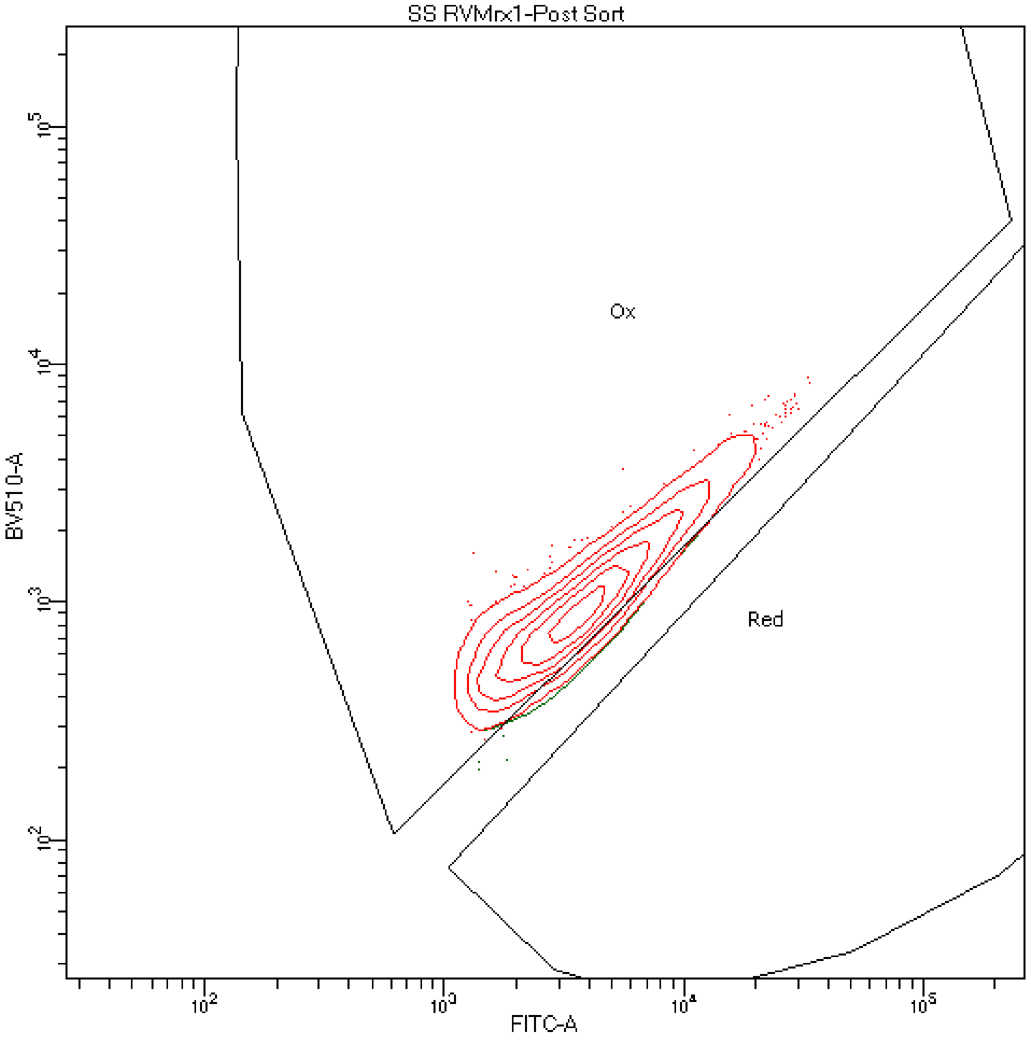

BD FACSDiva 8.0.1

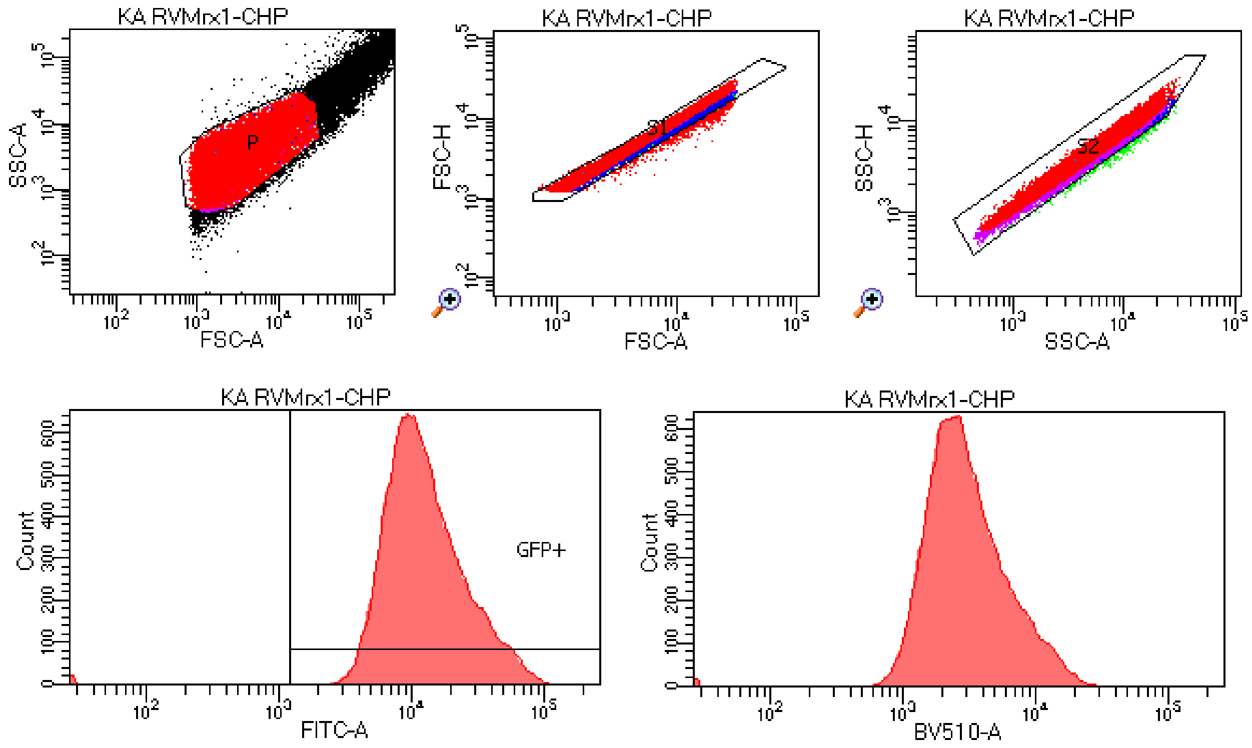

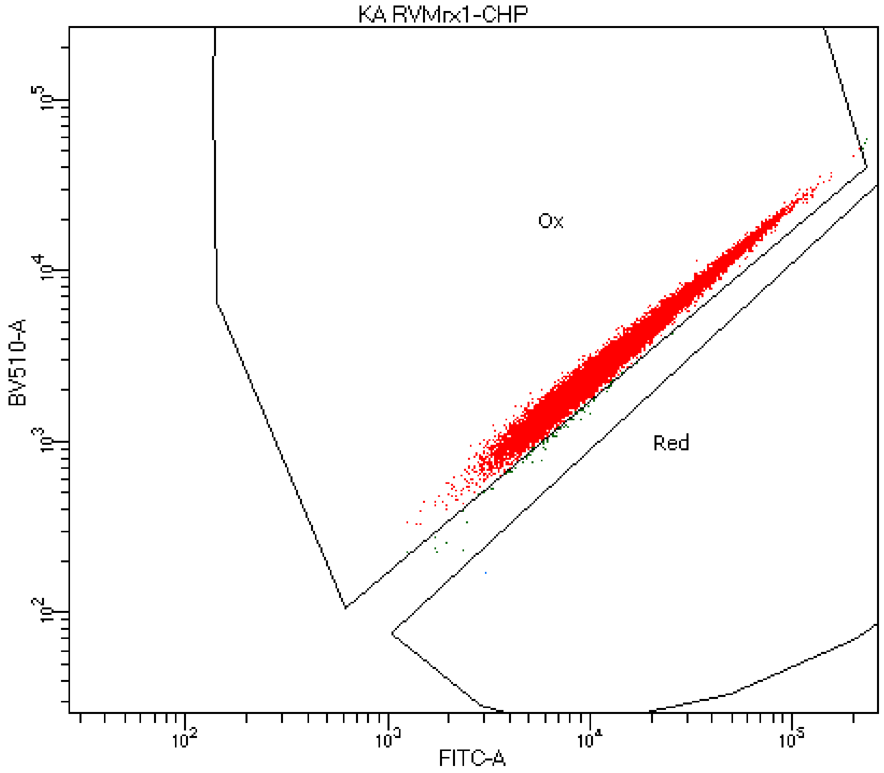

Tube: CHP

Population	#Events	%Parent	%Total
All Events	44,825	####	100.0
P	32,195	71.8	71.8
S1	31,482	97.8	70.2
S2	31,074	98.7	69.3
S3	28,906	93.0	64.5
S4	27,056	93.6	60.4
GFP+	26,808	99.1	59.8
Ox	26,711	99.6	59.6
Red	1	0.0	0.0

Experiment Name:	31Jan2017 Bac sorting
Specimen Name:	KA RVMrx1
Tube Name:	CHP
Record Date:	Jan 31, 2017 2:53:28 PM
$OP:	Administrator
GUID:	25285a88-022e-4307-818f-ee118a9...

Population	#Events	%Parent	FITC-A Median	BV510-A Median
S4	27,056	93.6	11,068	2,650
GFP+	26,808	99.1	11,155	2,665
Ox	26,711	99.6	11,174	2,672
Red	1	0.0	2,996	169

BD FACSDiva 8.0.1

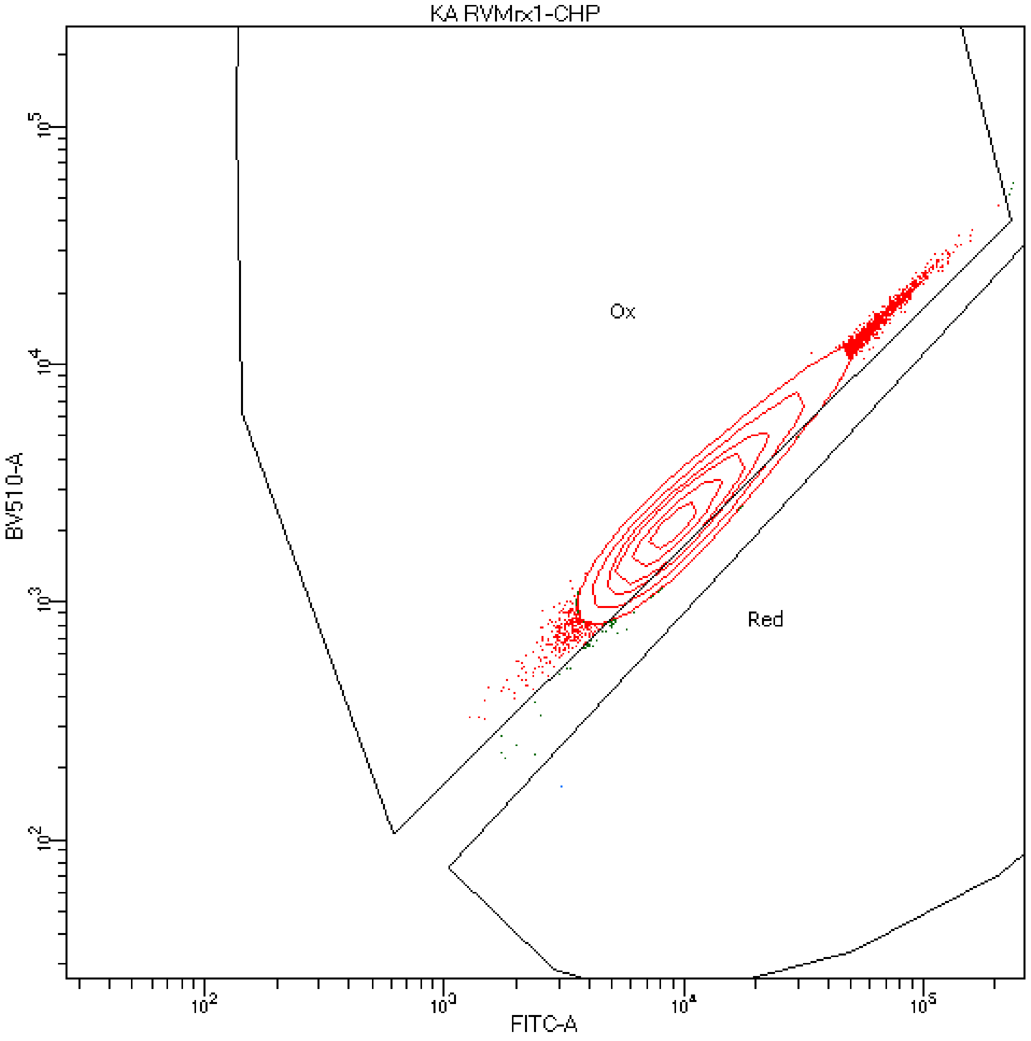

BD FACSDiva 8.0.1

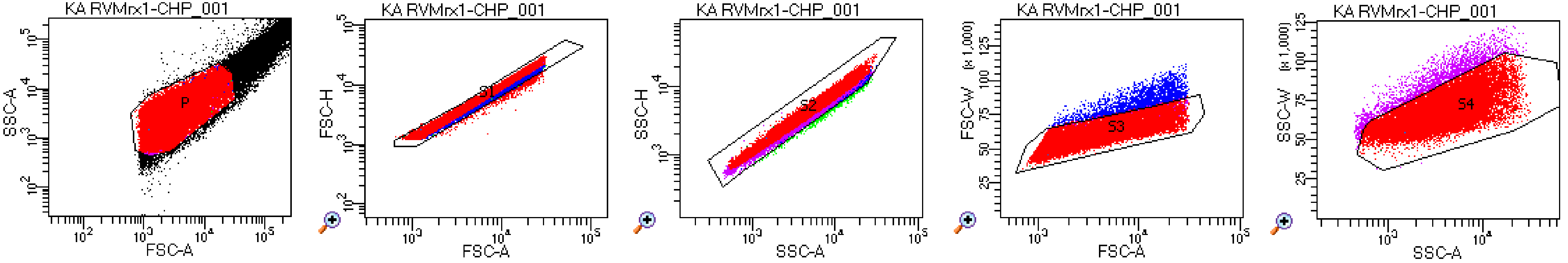

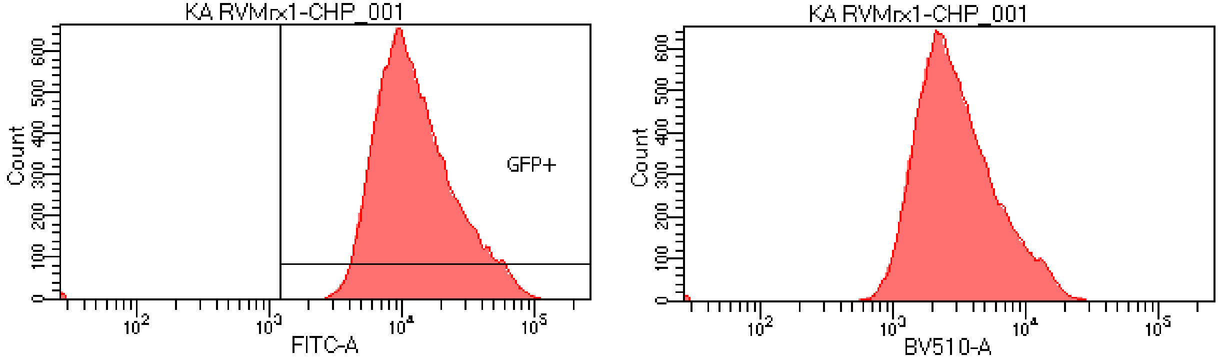

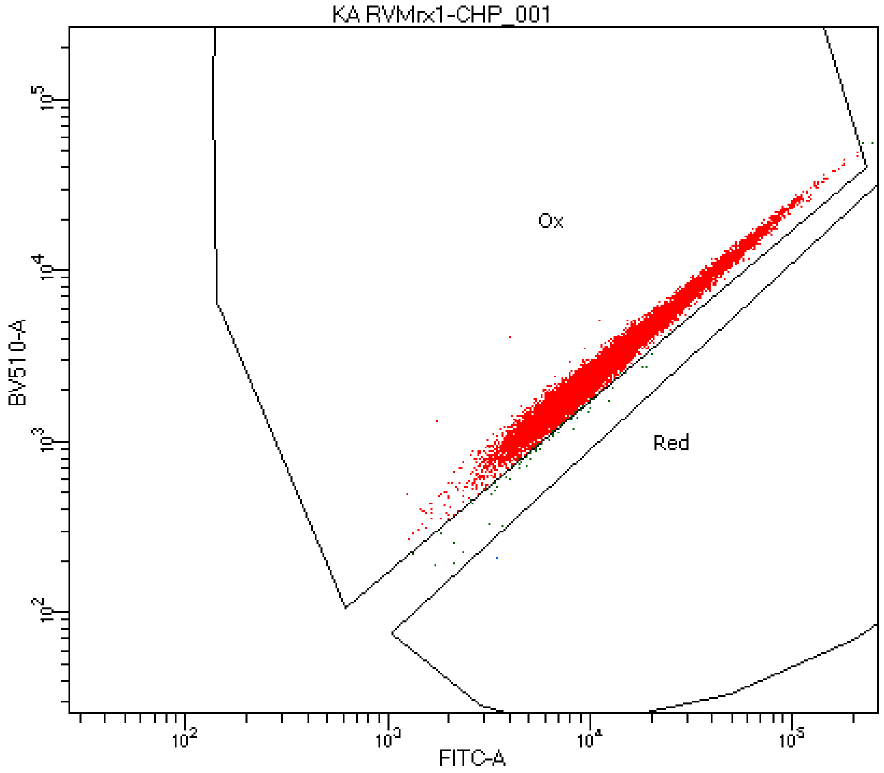

Tube: CHP_001

Population	#Events	%Parent	%Total
All Events	45,066	####	100.0
P	32,271	71.6	71.6
S1	31,617	98.0	70.2
S2	31,123	98.4	69.1
S3	28,972	93.1	64.3
S4	27,107	93.6	60.1
GFP+	26,891	99.2	59.7
Ox	26,788	99.6	59.4
Red	1	0.0	0.0

Experiment Name:	31Jan2017 Bac sorting
Specimen Name:	KA RVMrx1
Tube Name:	CHP_001
Record Date:	Jan 31, 2017 2:54:09 PM
$OP:	Administrator
GUID:	5d2d6c8d-5814-44ea-847d-b4133d...

Population	#Events	%Parent	FITC-A Median	BV510-A Median
S4	27,107	93.6	11,099	2,629
GFP+	26,891	99.2	11,164	2,646
Ox	26,788	99.6	11,199	2,653
Red	1	0.0	3,413	210

BD FACSDiva 8.0.1

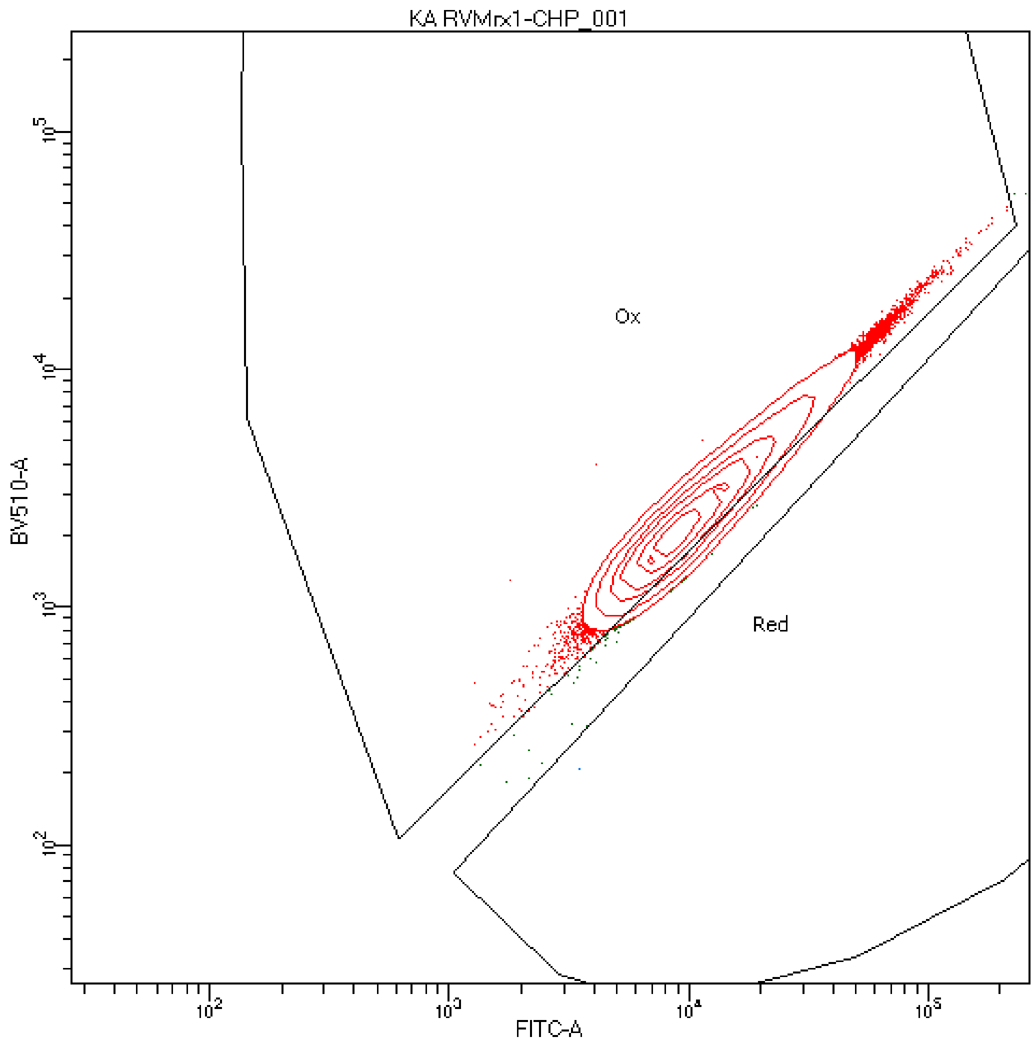

BD FACSDiva 8.0.1

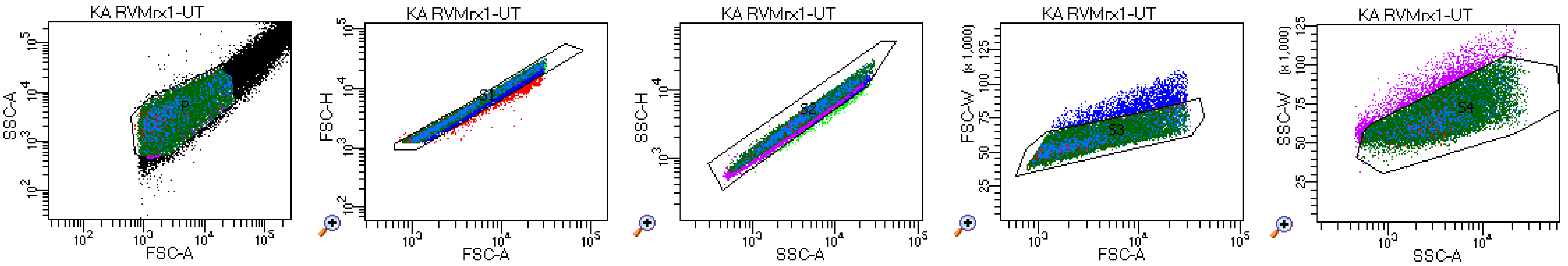

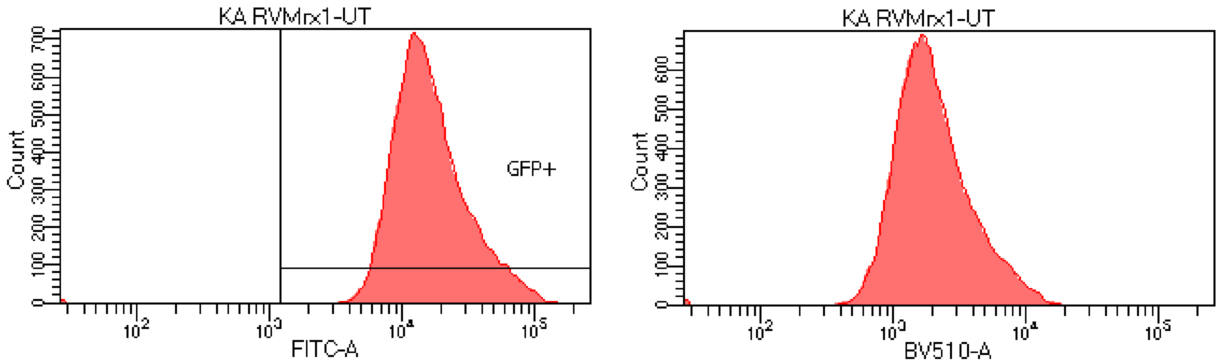

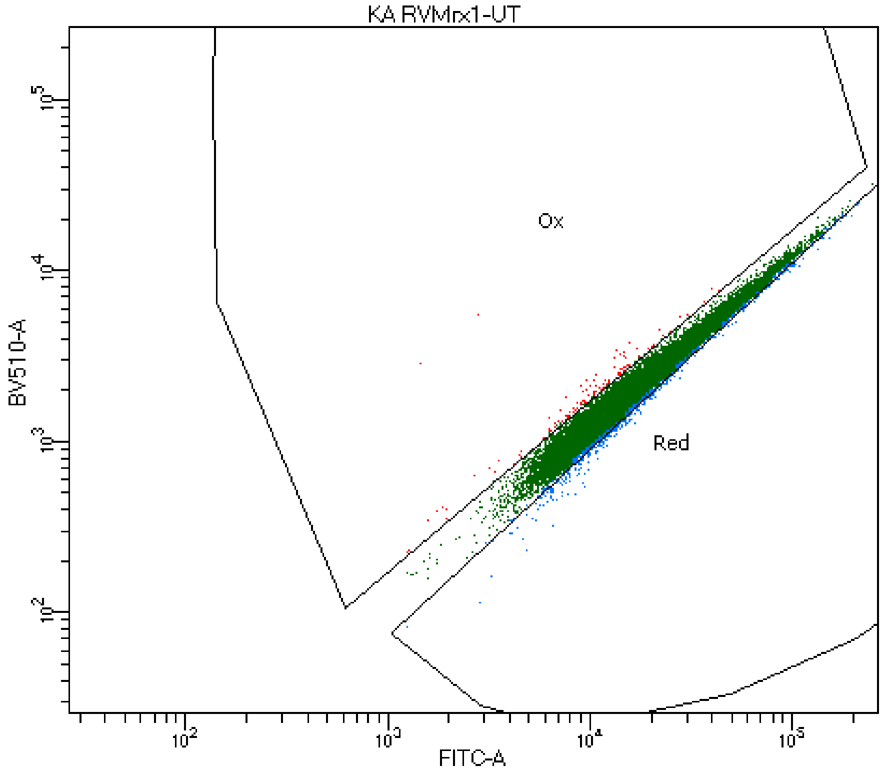

Tube: UT

Population	#Events	%Parent	%Total
All Events	44,103	####	100.0
P	31,739	72.0	72.0
S1	31,102	98.0	70.5
S2	30,695	98.7	69.6
S3	28,868	94.0	65.5
S4	27,182	94.2	61.6
GFP+	26,966	99.2	61.1
Ox	101	0.4	0.2
Red	770	2.9	1.7

Experiment Name: 31Jan2017 Bac sorting
Specimen Name: KA RVMrx1
Tube Name: UT
Record Date: Jan 31, 2017 2:51:53 PM
$OP: Administrator
GUID: d32366f1-1f74-4868-a301-c4a423d...

Population	#Events	%Parent	FITC-A Median	BV510-A Median
S4	27,182	94.2	14,448	1,766
GFP+	26,966	99.2	14,539	1,778
Ox	101	0.4	9,647	1,979
Red	770	2.9	16,366	1,554

BD FACSDiva 8.0.1

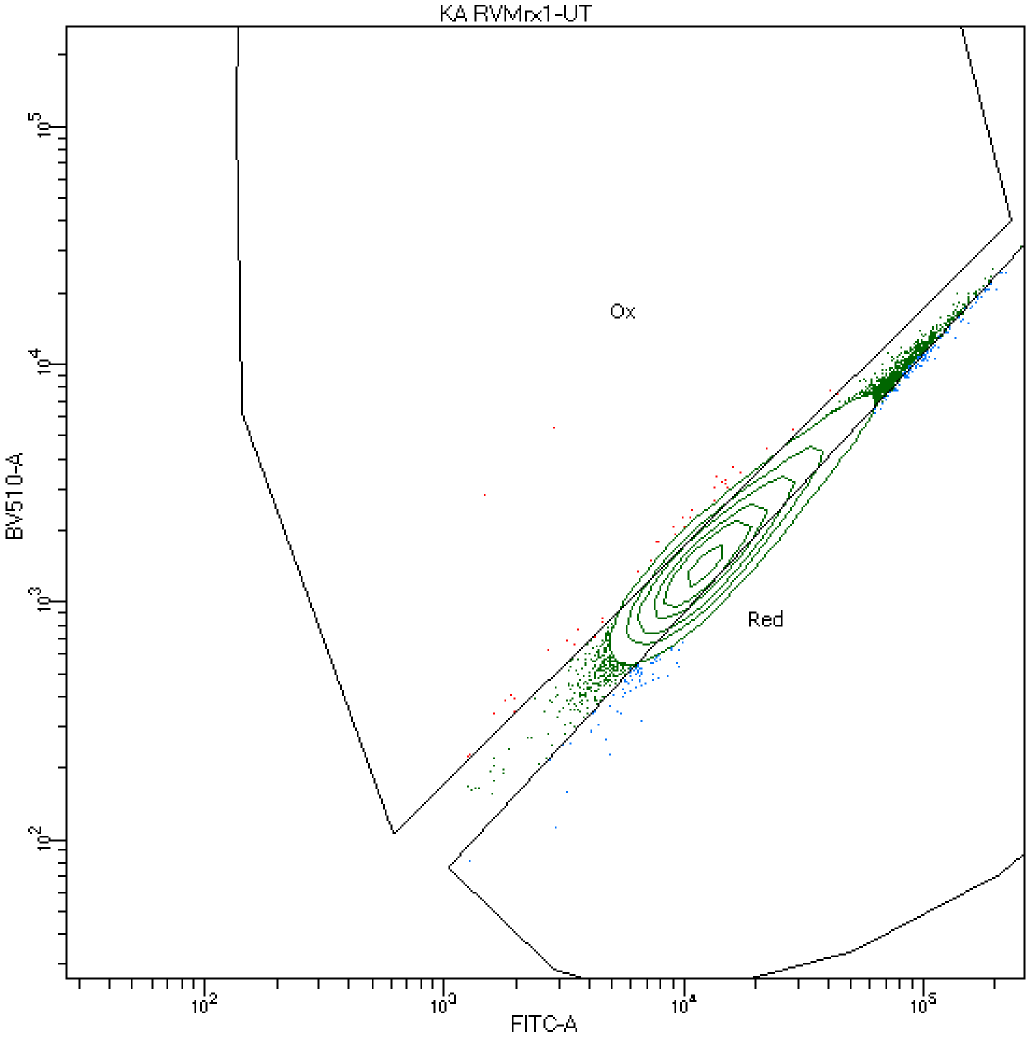

BD FACSDiva 8.0.1

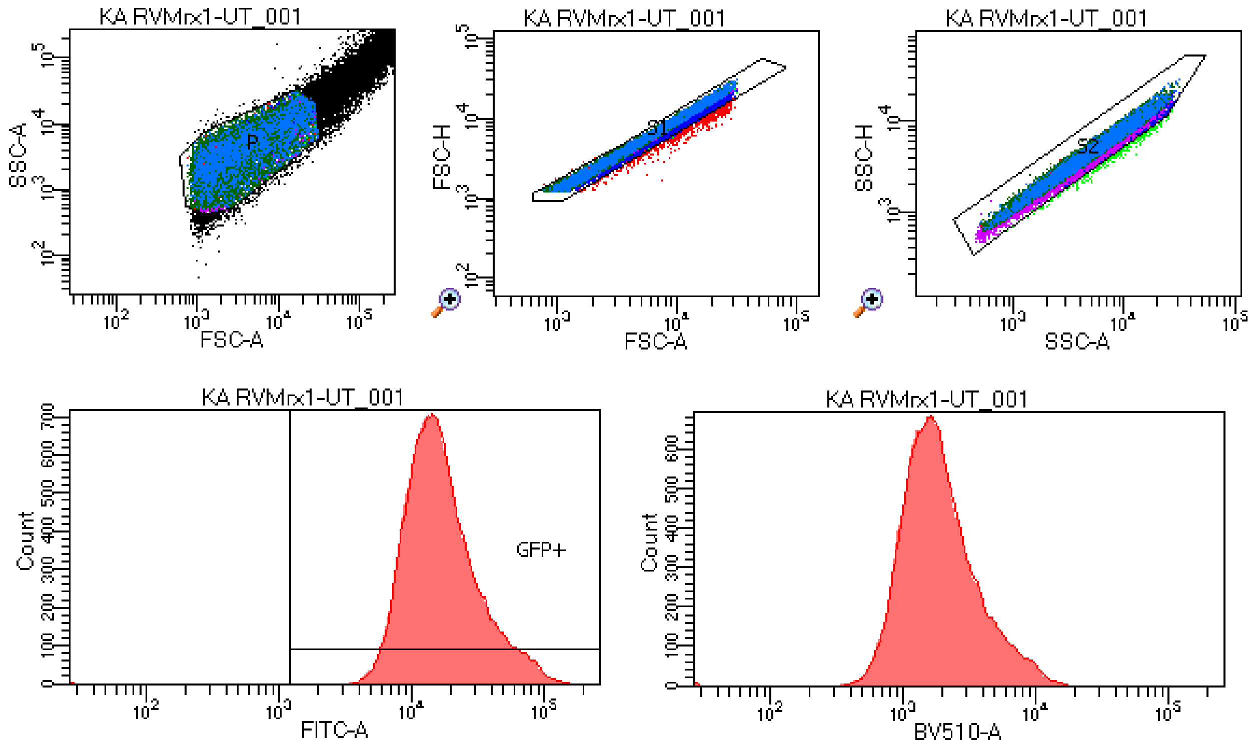

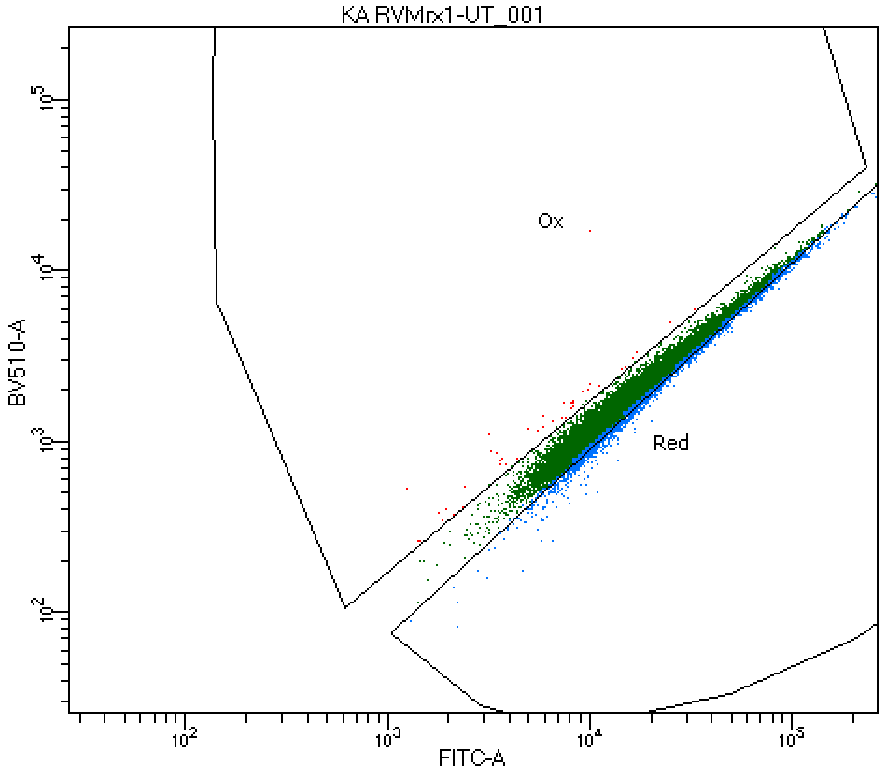

Tube: UT_001

Population	#Events	%Parent	%Total
All Events	43,872	####	100.0
P	31,784	72.4	72.4
S1	31,115	97.9	70.9
S2	30,688	98.6	69.9
S3	28,865	94.1	65.8
S4	27,210	94.3	62.0
GFP+	27,001	99.2	61.5
Ox	42	0.2	0.1
Red	2,913	10.8	6.6

Experiment Name:	31Jan2017 Bac sorting
Specimen Name:	KA RVMrx1
Tube Name:	UT_001
Record Date:	Jan 31, 2017 2:52:44 PM
$OP:	Administrator
GUID:	bb74f05f-8289-46cb-97a6-82b765d...

Population	#Events	%Parent	FITC-A Median	BV510-A Median
S4	27,210	94.3	14,912	1,668
GFP+	27,001	99.2	14,994	1,677
Ox	42	0.2	6,218	1,338
Red	2,913	10.8	19,809	1,877

BD FACSDiva 8.0.1

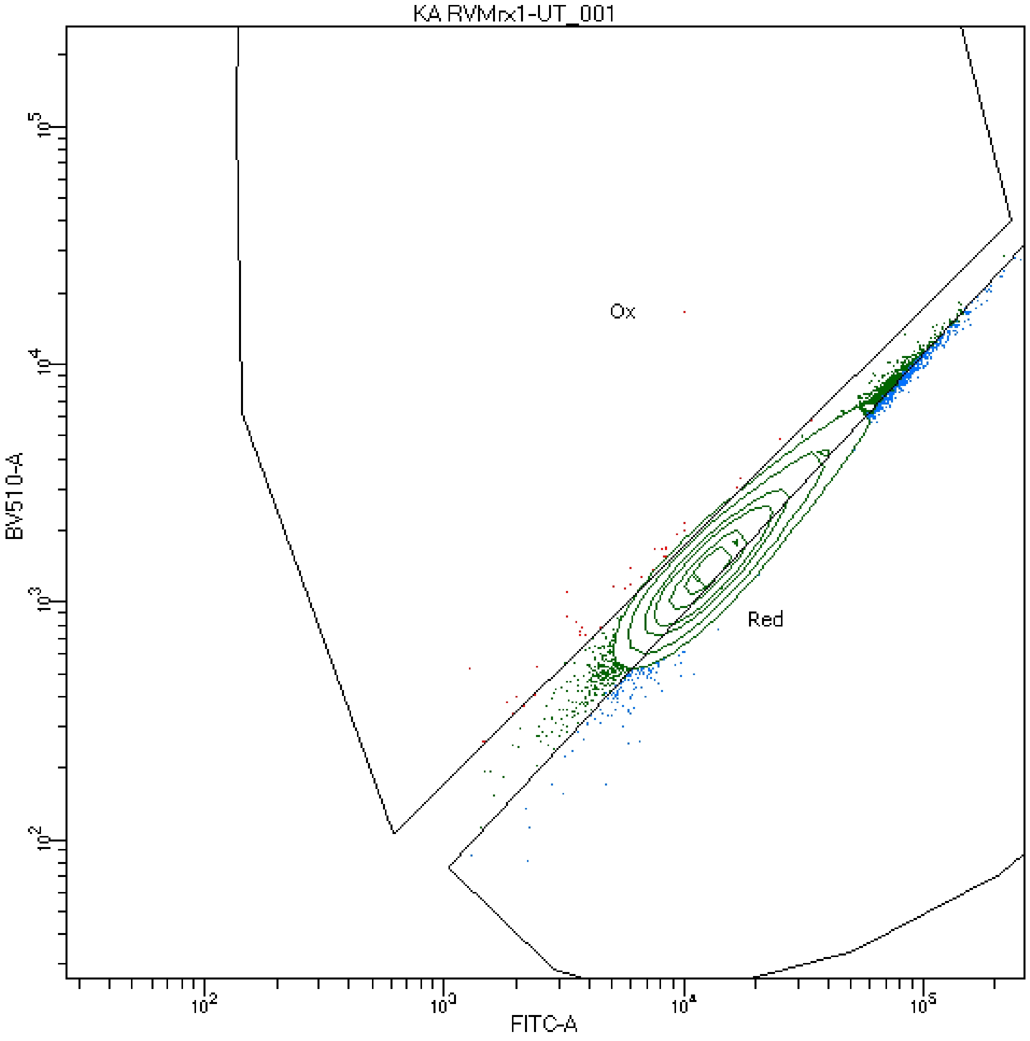

BD FACSDiva 8.0.1

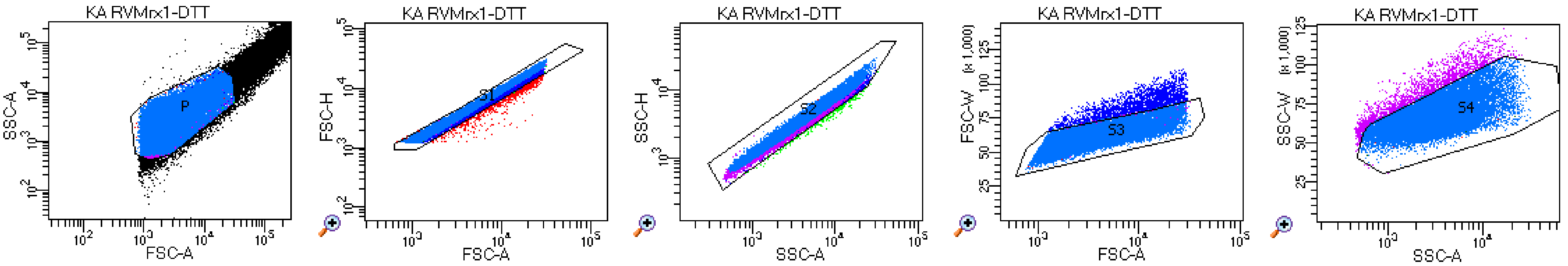

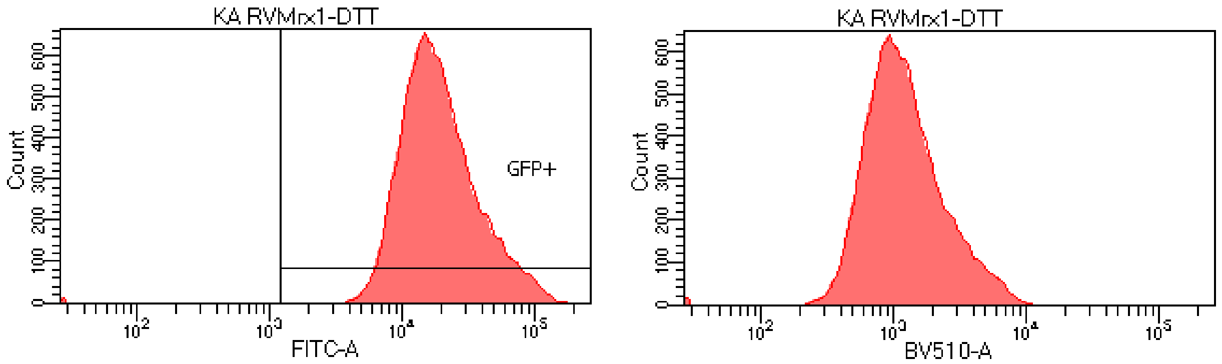

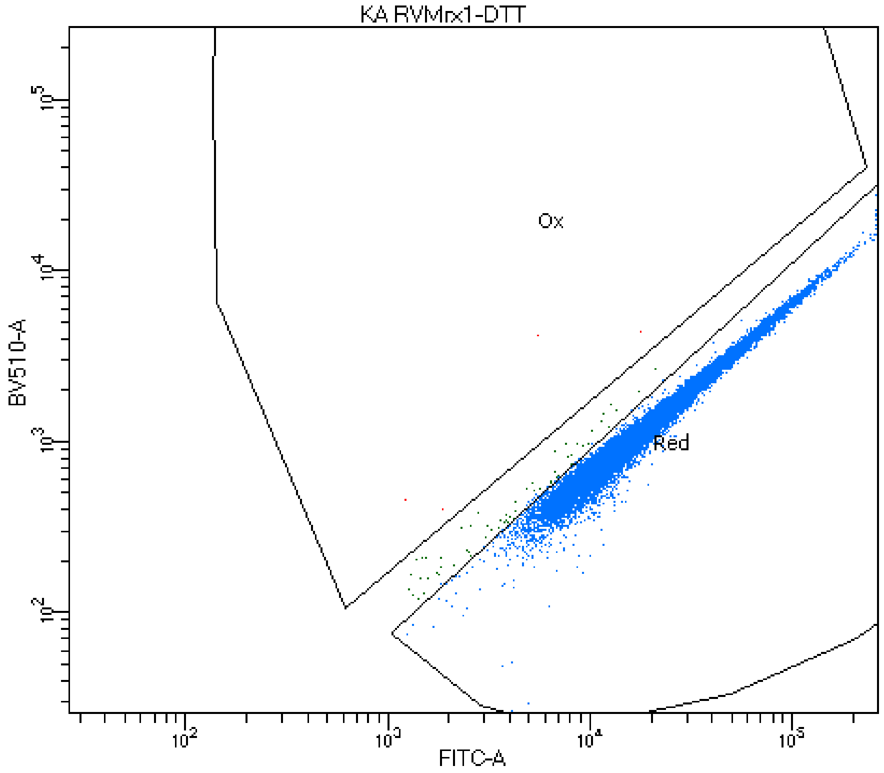

Tube: DTT

Population	#Events	%Parent	%Total
All Events	44,885	####	100.0
P	32,113	71.5	71.5
S1	31,331	97.6	69.8
S2	30,944	98.8	68.9
S3	28,752	92.9	64.1
S4	26,859	93.4	59.8
GFP+	26,635	99.2	59.3
Ox	4	0.0	0.0
Red	26,566	99.7	59.2

Experiment Name: 31Jan2017 Bac sorting
Specimen Name: KA RVMrx1
Tube Name: DTT
Record Date: Jan 31, 2017 2:54:50 PM
$OP: Administrator
GUID: 48236b22-91eb-4017-b498-f3eb249...

Population	#Events	%Parent	FITC-A Median	BV510-A Median
S4	26,859	93.4	17,078	1,070
GFP+	26,635	99.2	17,192	1,078
Ox	4	0.0	3,658	2,314
Red	26,566	99.7	17,214	1,079

BD FACSDiva 8.0.1

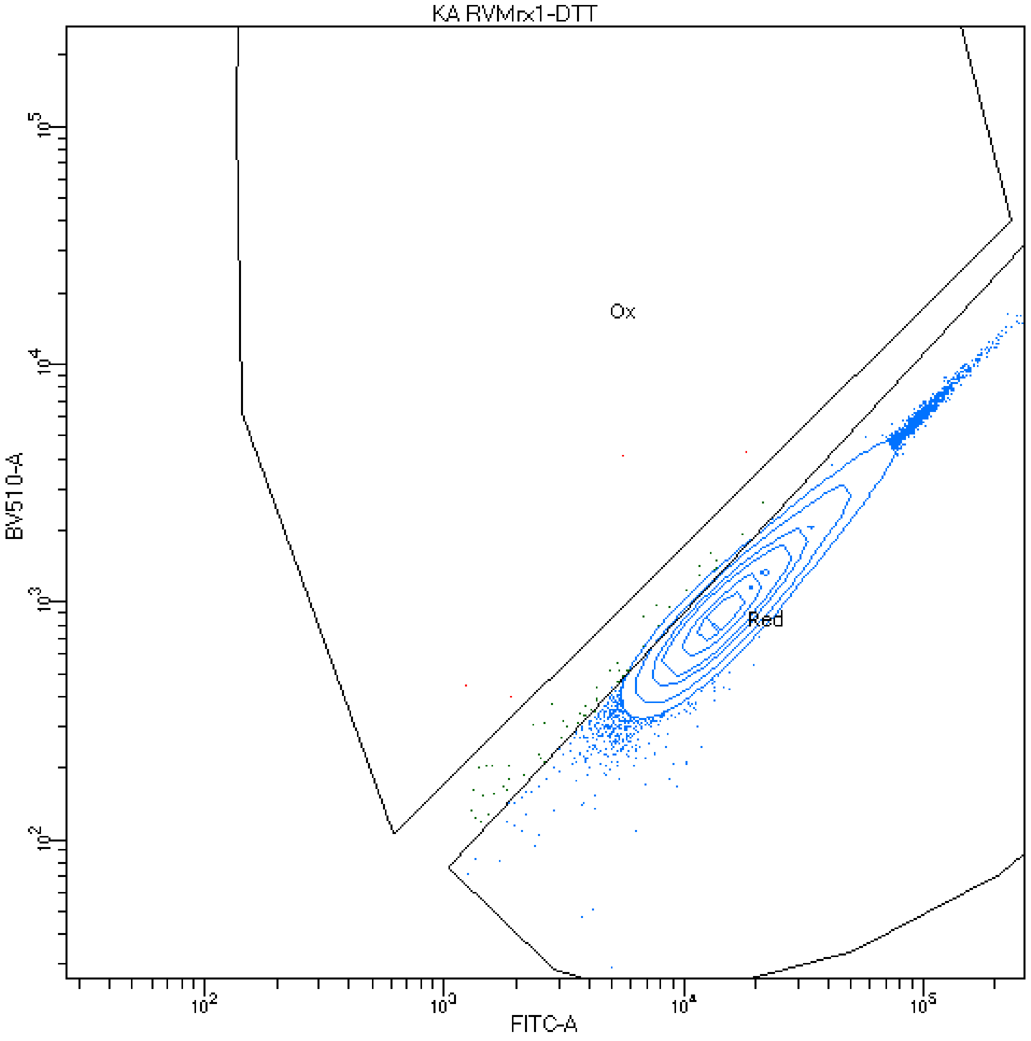

BD FACSDiva 8.0.1

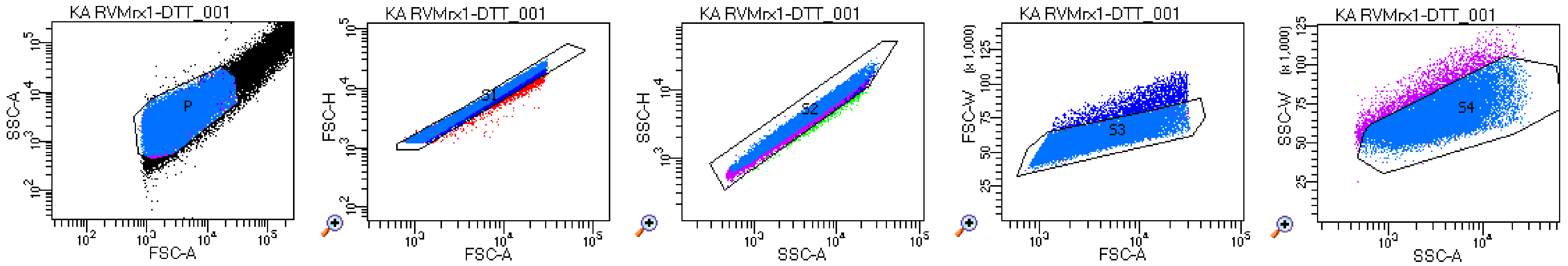

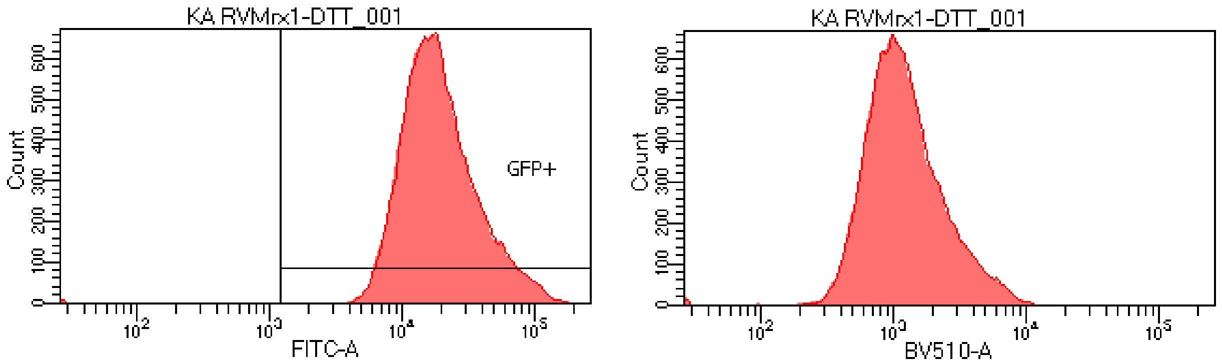

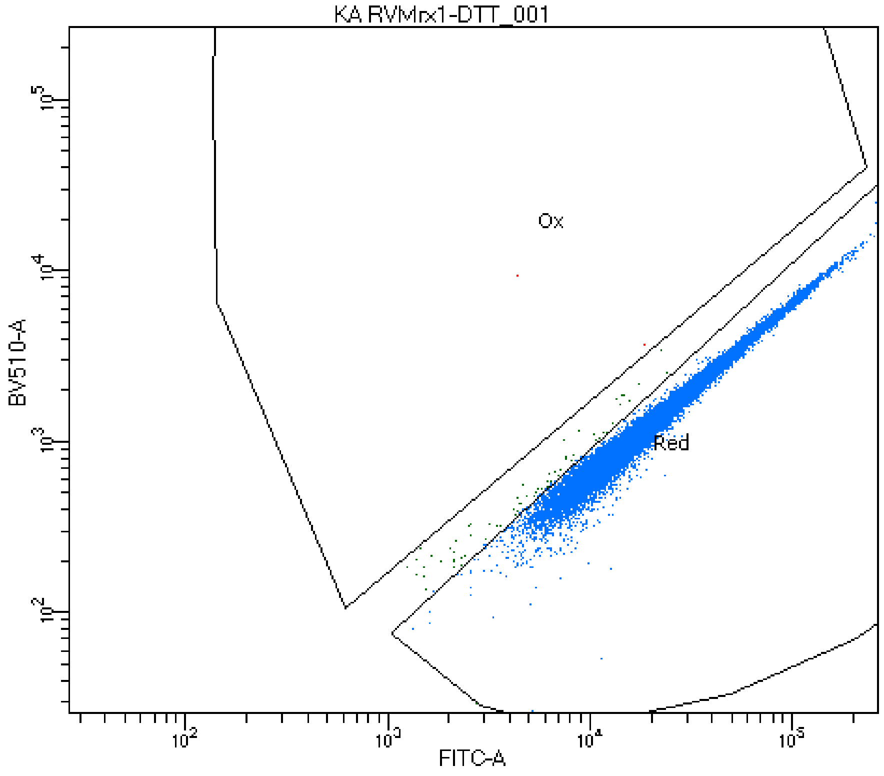

Tube: DTT_001

Population	#Events	%Parent	%Total
All Events	44,689	####	100.0
P	32,051	71.7	71.7
S1	31,354	97.8	70.2
S2	30,954	98.7	69.3
S3	28,932	93.5	64.7
S4	27,126	93.8	60.7
GFP+	26,896	99.2	60.2
Ox	2	0.0	0.0
Red	26,827	99.7	60.0

Experiment Name:	31Jan2017 Bac sorting
Specimen Name:	KA RVMrx1
Tube Name:	DTT_001
Record Date:	Jan 31, 2017 2:55:32 PM
$OP:	Administrator
GUID:	730e0b60-bd8d-4b09-a558-1ac167...

Population	#Events	%Parent	FITC-A Median	BV510-A Median
S4	27,126	93.8	17,172	1,075
GFP+	26,896	99.2	17,273	1,081
Ox	2	0.0	11,379	6,481
Red	26,827	99.7	17,299	1,083

BD FACSDiva 8.0.1

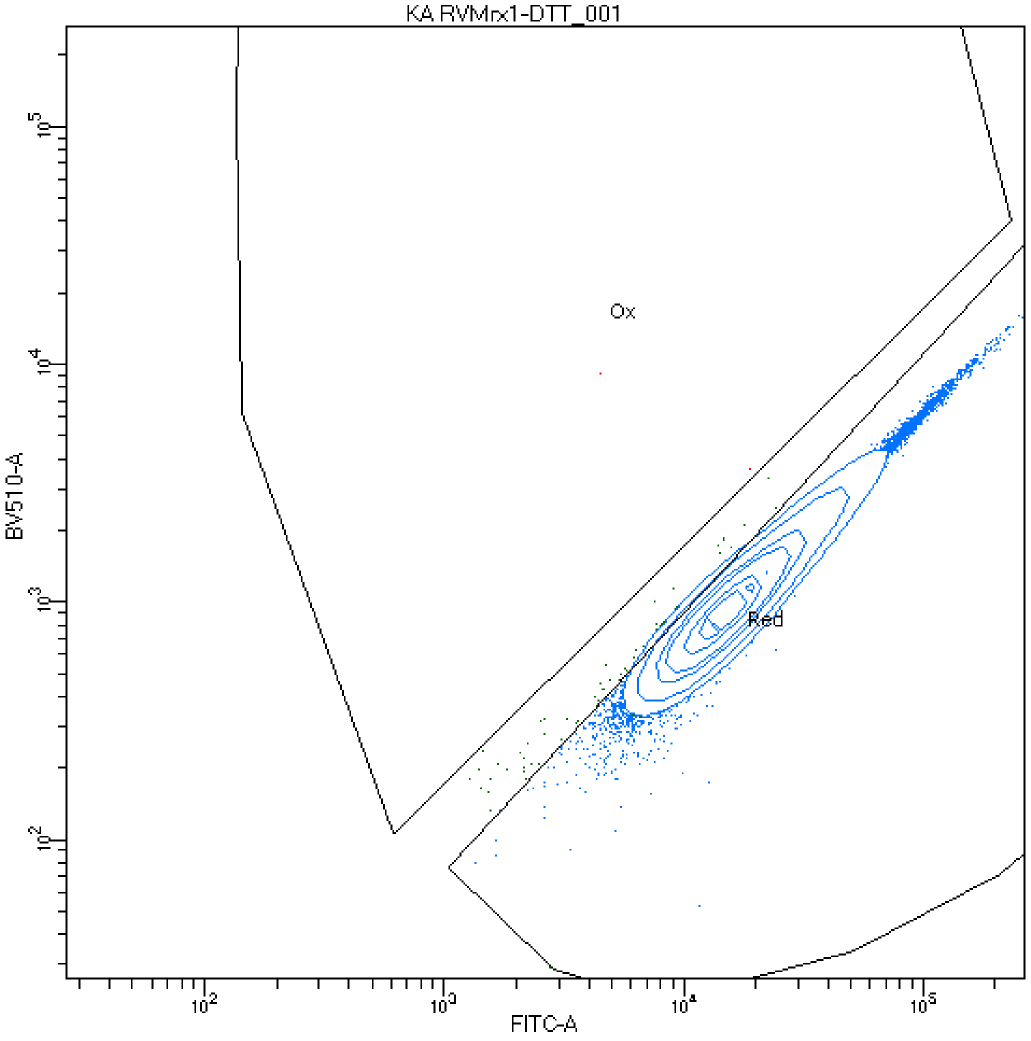

BD FACSDiva 8.0.1

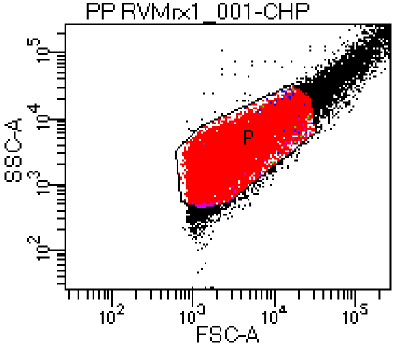

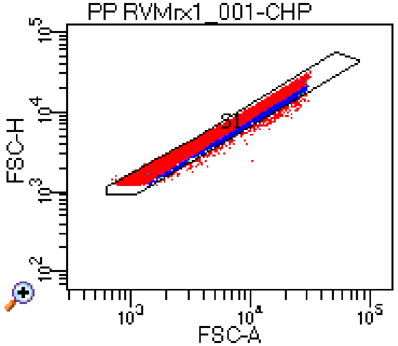

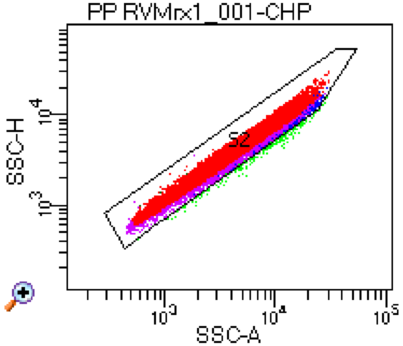

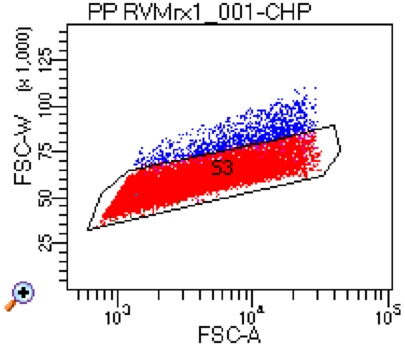

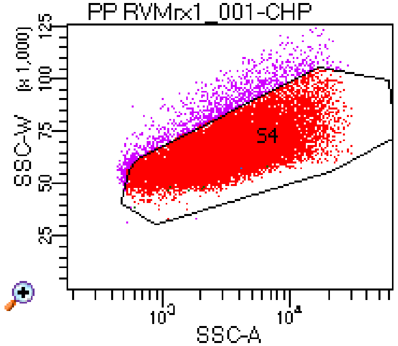

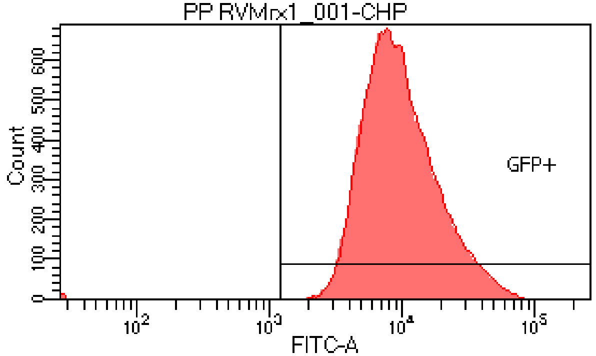

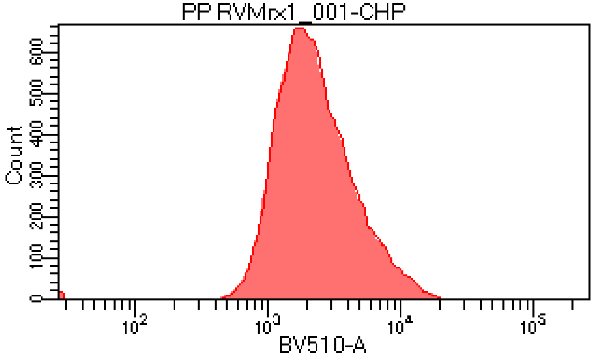

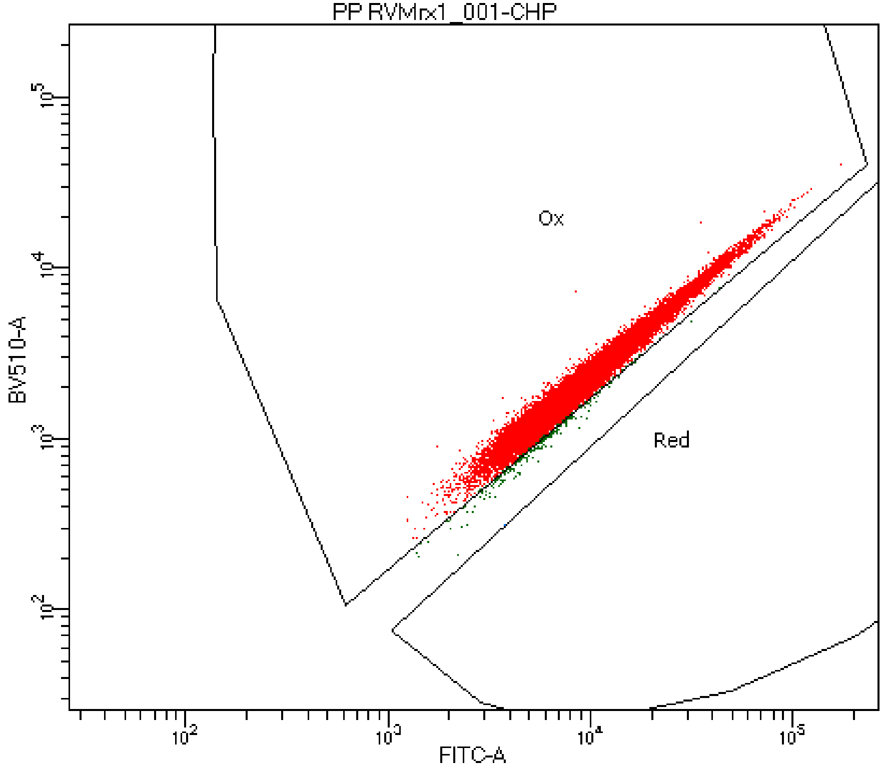

Tube: CHP

Population	#Events	%Parent	%Total
All Events	35,756	####	100.0
P	31,150	87.1	87.1
S1	30,784	98.8	86.1
S2	30,501	99.1	85.3
S3	29,369	96.3	82.1
S4	28,104	95.7	78.6
GFP+	27,912	99.3	78.1
Ox	27,557	98.7	77.1
Red	1	0.0	0.0

Experiment Name:	31Jan2017 Bac sorting
Specimen Name:	PP RVMrx1_001
Tube Name:	CHP
Record Date:	Jan 31, 2017 2:58:58 PM
$OP:	Administrator
GUID:	925a5e5b-f631-4c59-864f-72b4093...

Population	#Events	%Parent	FITC-A Median	BV510-A Median
S4	28,104	95.7	8,701	2,018
GFP+	27,912	99.3	8,750	2,029
Ox	27,557	98.7	8,807	2,050
Red	1	0.0	3,763	312

BD FACSDiva 8.0.1

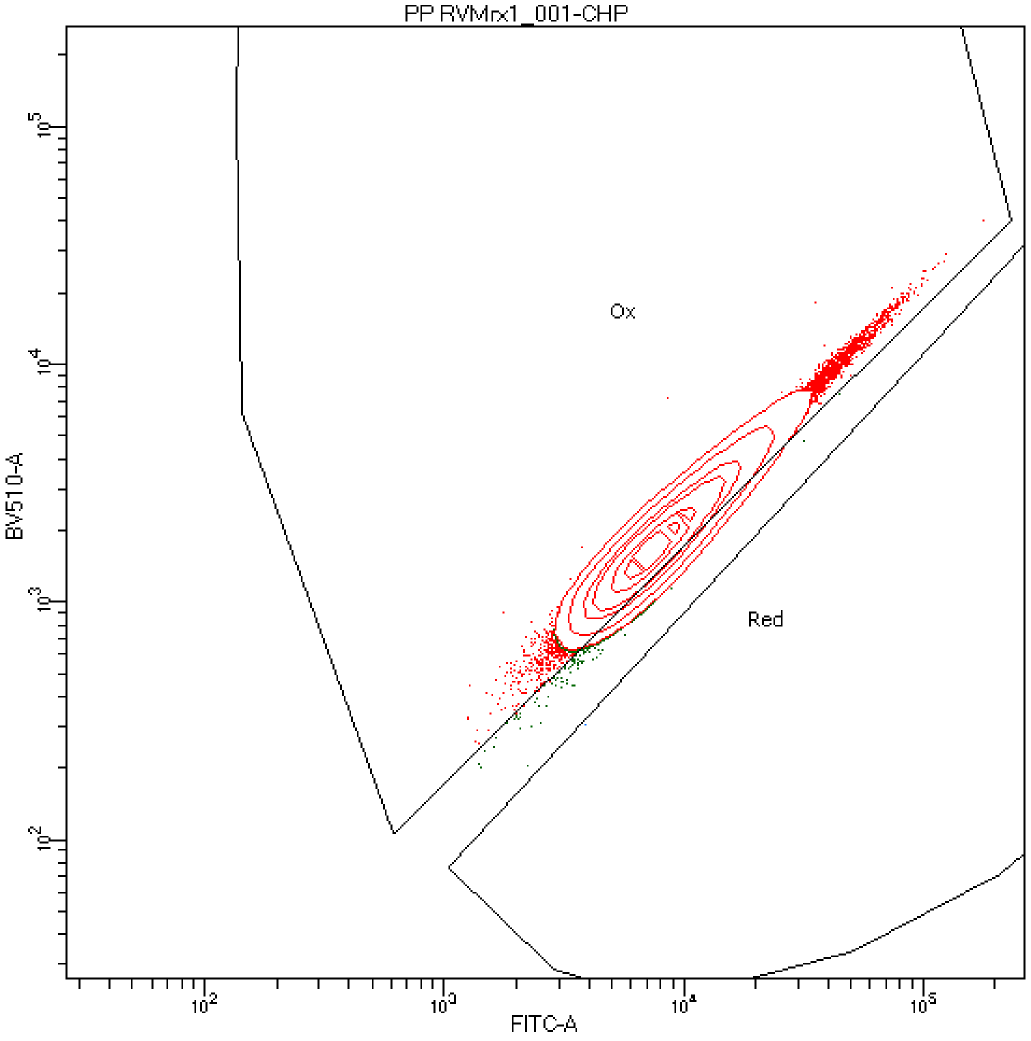

BD FACSDiva 8.0.1

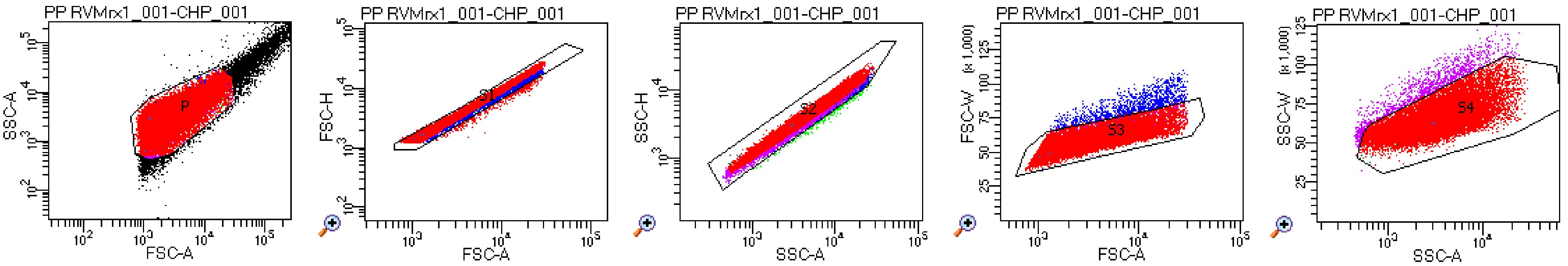

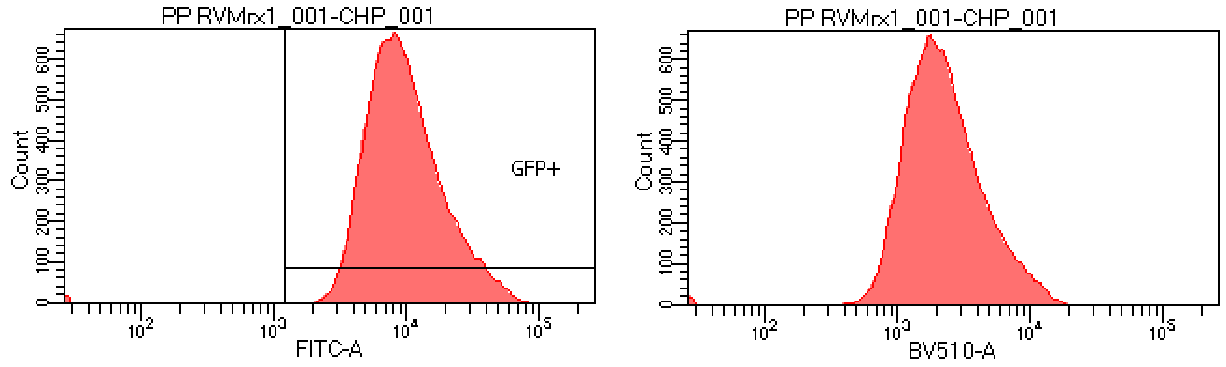

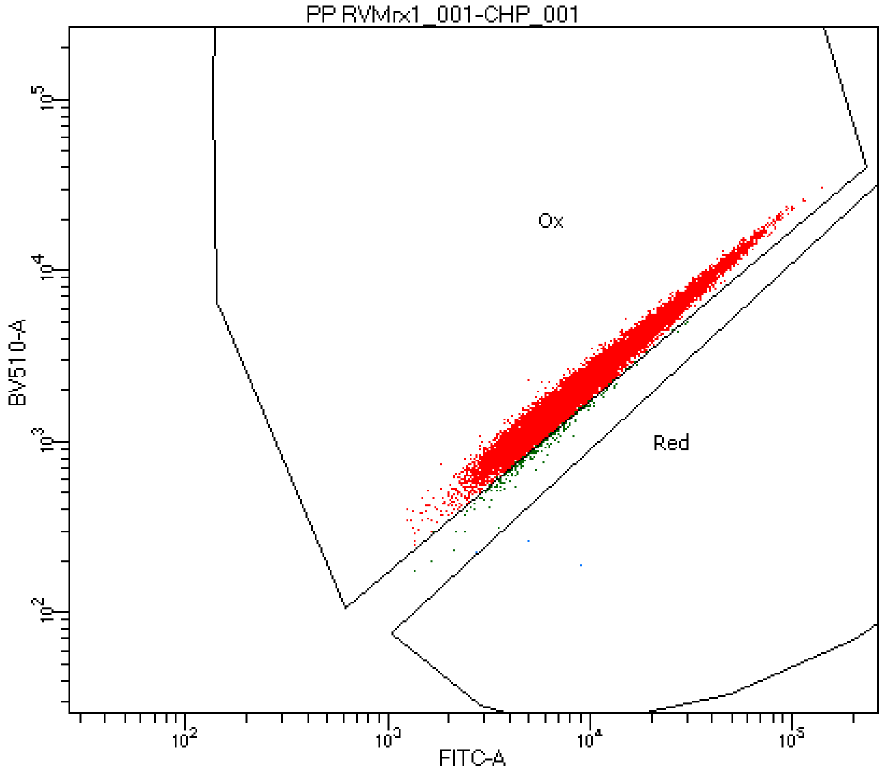

Tube: CHP_001

Population	#Events	%Parent	%Total
All Events	35,602	####	100.0
P	31,172	87.6	87.6
S1	30,751	98.6	86.4
S2	30,460	99.1	85.6
S3	29,272	96.1	82.2
S4	28,039	95.8	78.8
GFP+	27,826	99.2	78.2
Ox	27,496	98.8	77.2
Red	3	0.0	0.0

Experiment Name:	31Jan2017 Bac sorting
Specimen Name:	PP RVMrx1_001
Tube Name:	CHP_001
Record Date:	Jan 31, 2017 2:59:32 PM
$OP:	Administrator
GUID:	89180d18-22f9-471d-8077-9453b80...

Population	#Events	%Parent	FITC-A Median	BV510-A Median
S4	28,039	95.8	8,716	2,017
GFP+	27,826	99.2	8,765	2,027
Ox	27,496	98.8	8,808	2,043
Red	3	0.0	4,879	224

BD FACSDiva 8.0.1

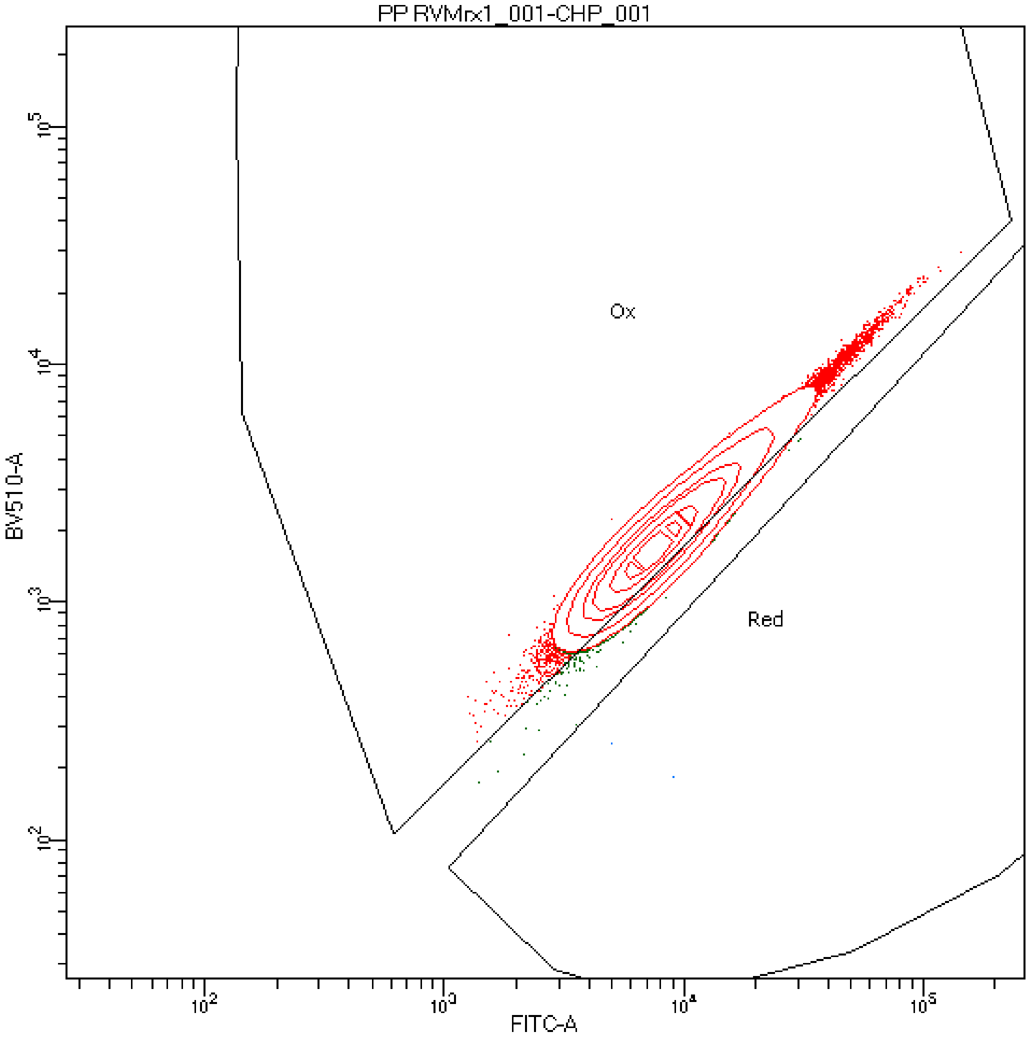

BD FACSDiva 8.0.1

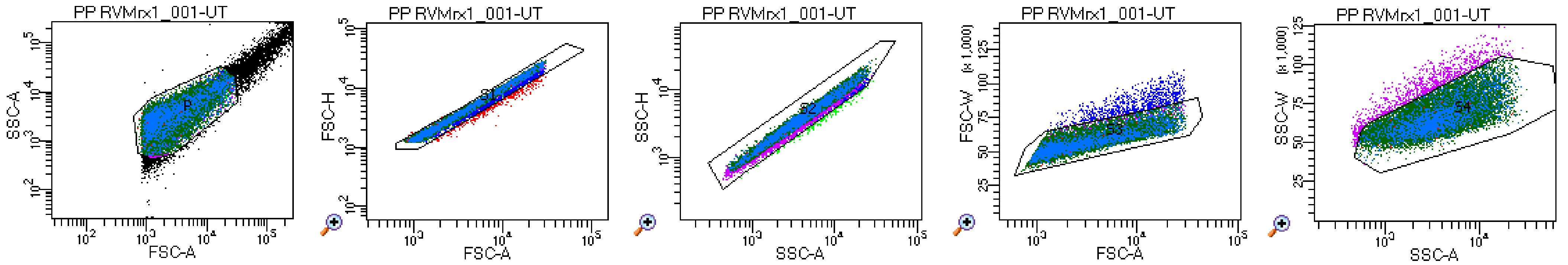

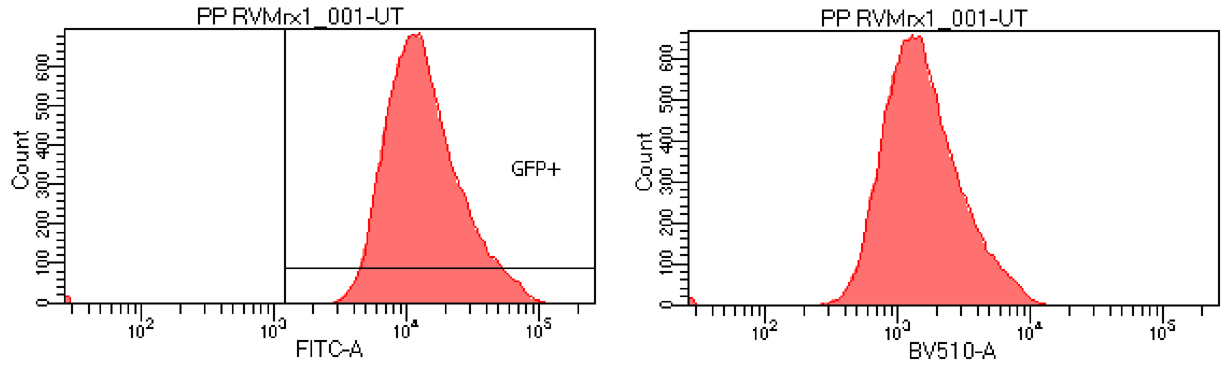

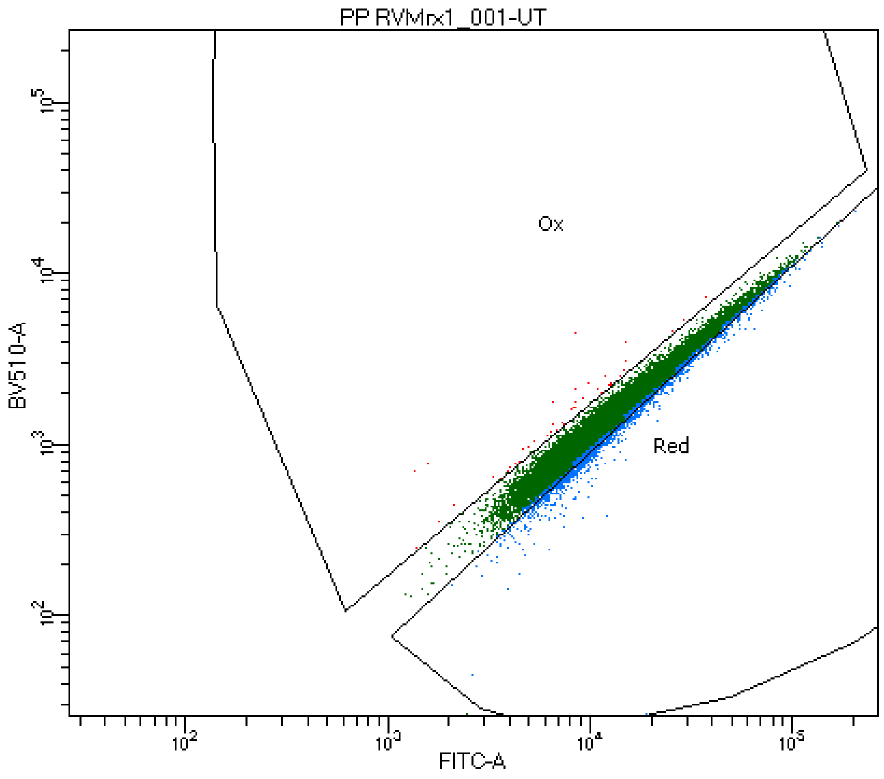

Tube: UT

Population	#Events	%Parent	%Total
All Events	34,532	####	100.0
P	30,909	89.5	89.5
S1	30,553	98.8	88.5
S2	30,334	99.3	87.8
S3	29,516	97.3	85.5
S4	28,449	96.4	82.4
GFP+	28,235	99.2	81.8
Ox	44	0.2	0.1
Red	2,043	7.2	5.9

Experiment Name:	31Jan2017 Bac sorting
Specimen Name:	PP RVMrx1_001
Tube Name:	UT
Record Date:	Jan 31, 2017 2:57:49 PM
$OP:	Administrator
GUID:	6182d615-c230-49b2-afcd-5411562...

Population	#Events	%Parent	FITC-A Median	BV510-A Median
S4	28,449	96.4	12,125	1,382
GFP+	28,235	99.2	12,196	1,392
Ox	44	0.2	7,683	1,611
Red	2,043	7.2	13,820	1,248

BD FACSDiva 8.0.1

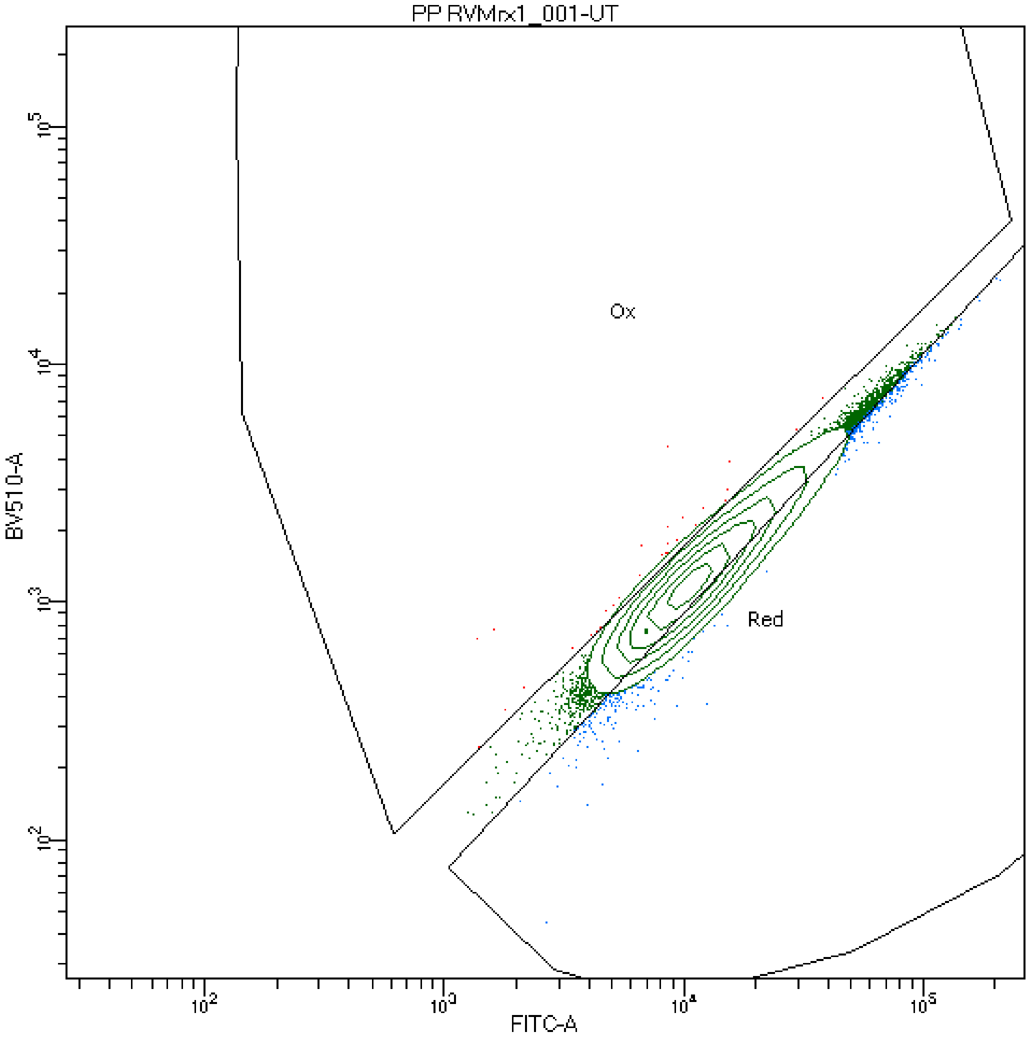

BD FACSDiva 8.0.1

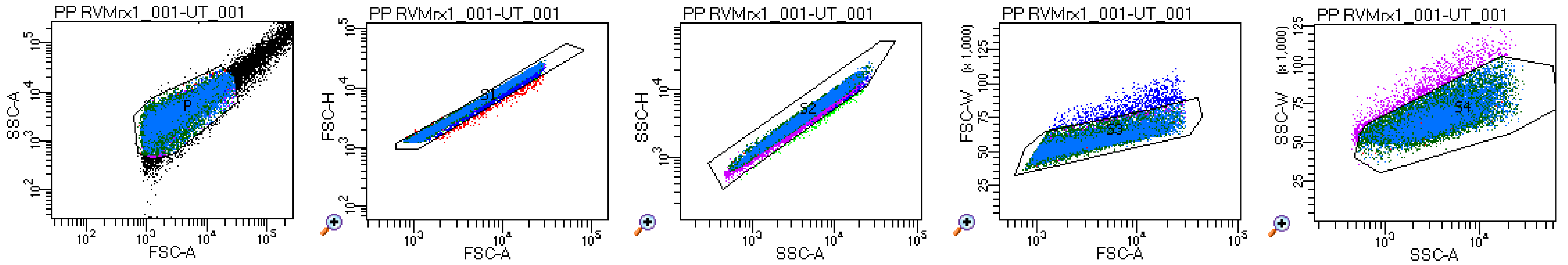

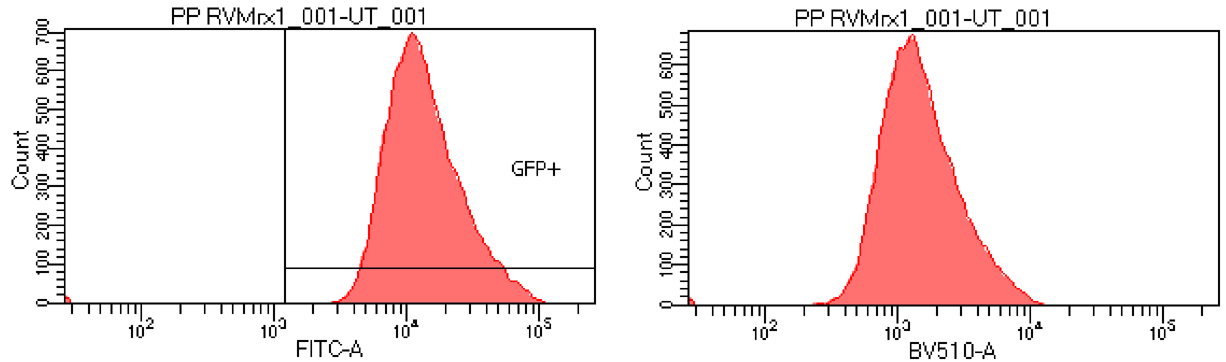

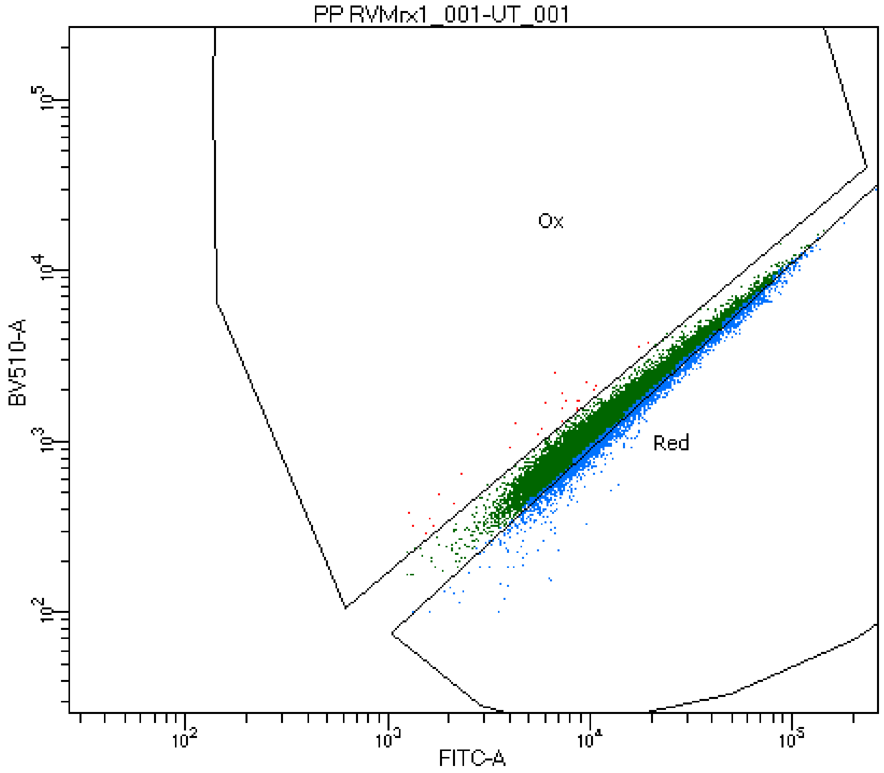

Tube: UT_001

Population	#Events	%Parent	%Total
All Events	35,007	####	100.0
P	30,900	88.3	88.3
S1	30,542	98.8	87.2
S2	30,331	99.3	86.6
S3	29,463	97.1	84.2
S4	28,399	96.4	81.1
GFP+	28,206	99.3	80.6
Ox	27	0.1	0.1
Red	4,271	15.1	12.2

Experiment Name: 31Jan2017 Bac sorting
Specimen Name: PP RVMrx1_001
Tube Name: UT_001
Record Date: Jan 31, 2017 2:58:23 PM
$OP: Administrator
GUID: 11f6b488-1989-4136-8008-bd4b37b...

Population	#Events	%Parent	FITC-A Median	BV510-A Median
S4	28,399	96.4	12,133	1,318
GFP+	28,206	99.3	12,200	1,324
Ox	27	0.1	6,650	1,524
Red	4,271	15.1	16,015	1,494

BD FACSDiva 8.0.1

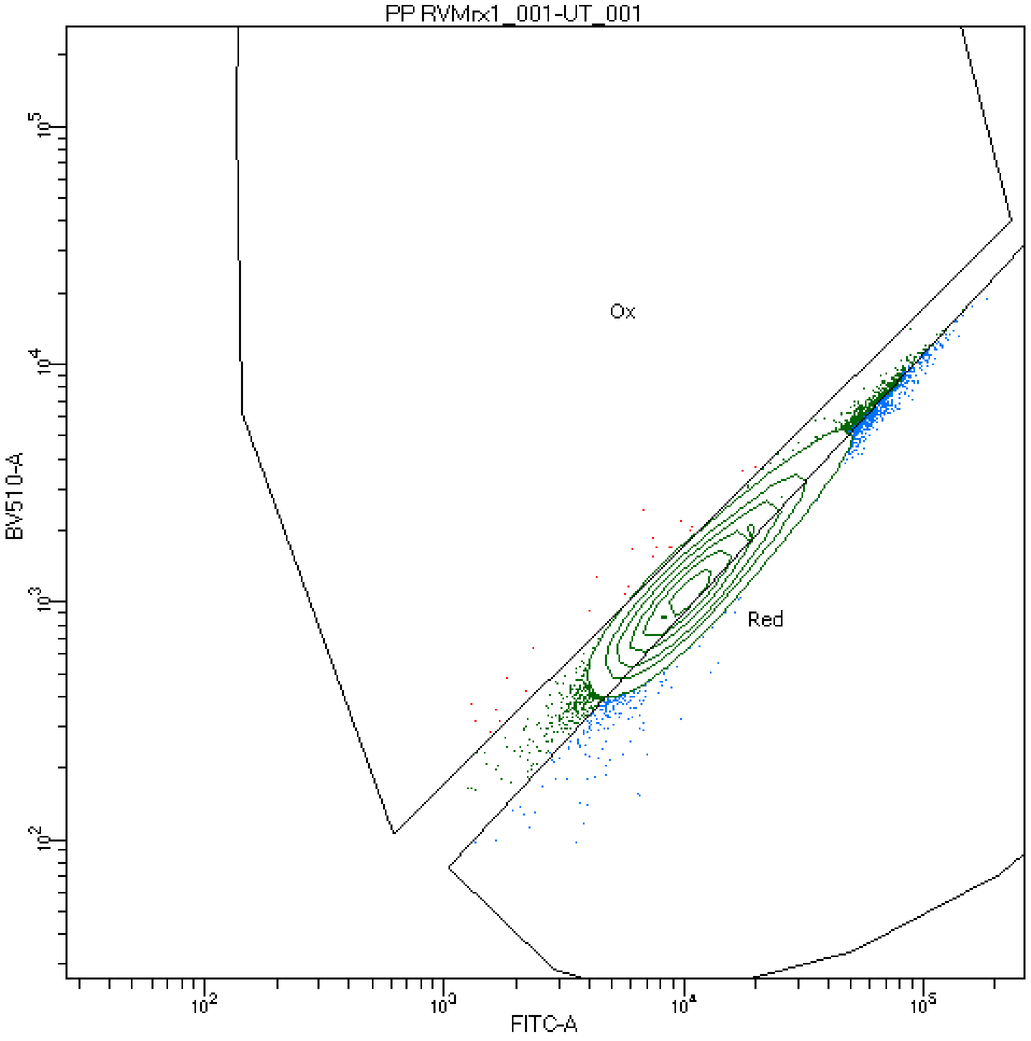

BD FACSDiva 8.0.1

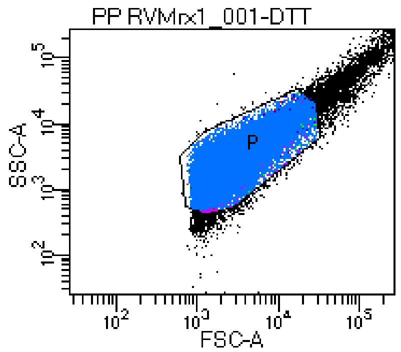

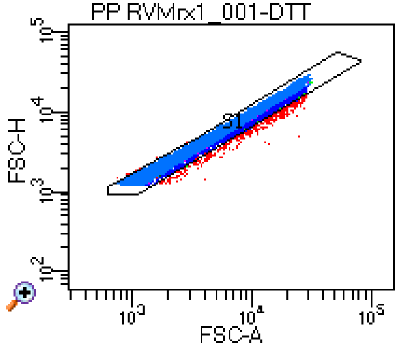

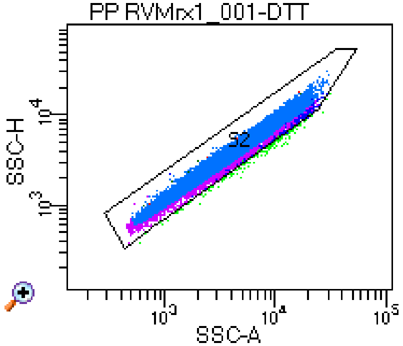

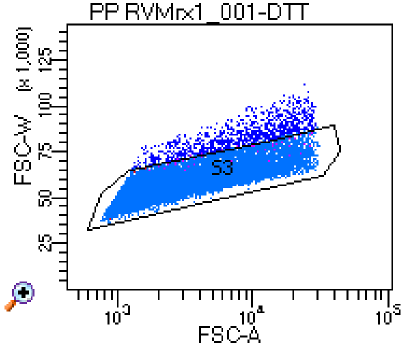

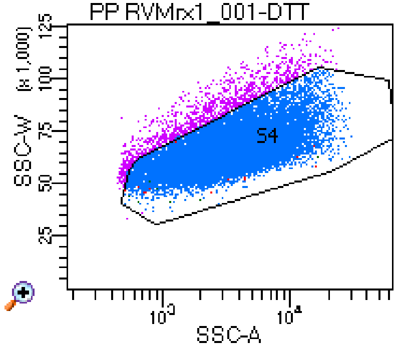

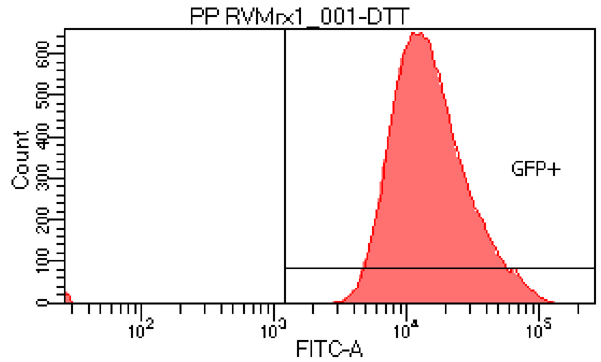

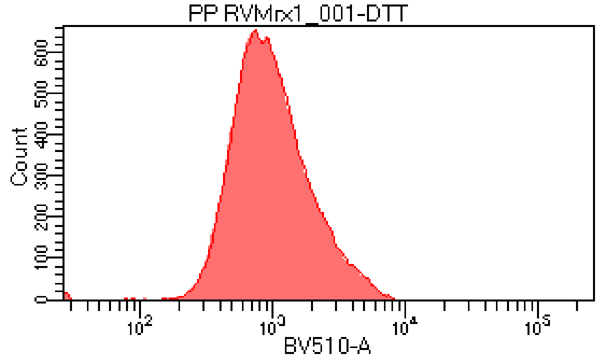

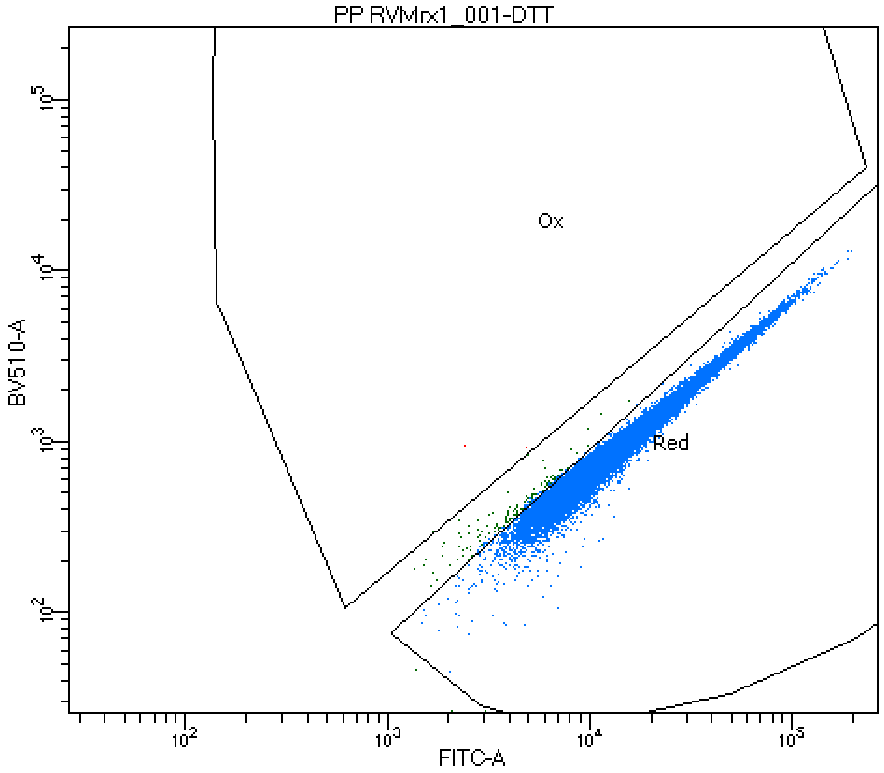

Tube: DTT

Population	#Events	%Parent	%Total
All Events	34,972	####	100.0
P	31,053	88.8	88.8
S1	30,606	98.6	87.5
S2	30,329	99.1	86.7
S3	29,291	96.6	83.8
S4	28,116	96.0	80.4
GFP+	27,882	99.2	79.7
Ox	2	0.0	0.0
Red	27,740	99.5	79.3

Experiment Name: 31Jan2017 Bac sorting
Specimen Name: PP RVMrx1_001
Tube Name: DTT
Record Date: Jan 31, 2017 2:56:37 PM
$OP: Administrator
GUID: c0f460a6-d2f6-47bc-a856-a797893...

Population	#Events	%Parent	FITC-A Median	BV510-A Median
S4	28,116	96.0	13,533	876
GFP+	27,882	99.2	13,620	882
Ox	2	0.0	3,603	936
Red	27,740	99.5	13,690	885

BD FACSDiva 8.0.1

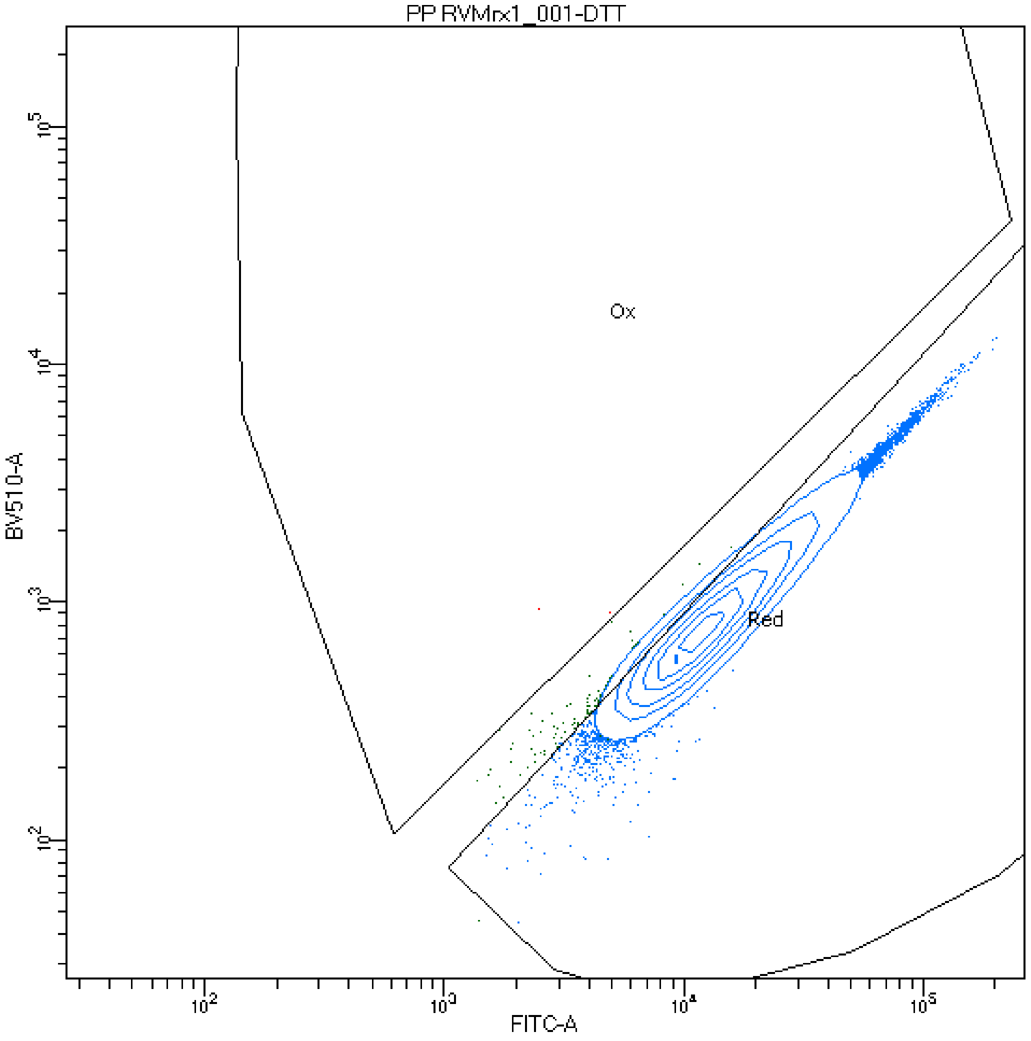

BD FACSDiva 8.0.1

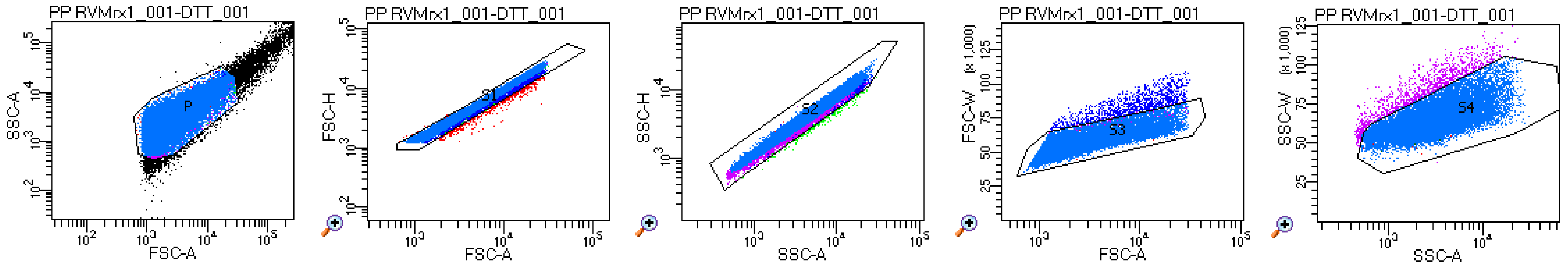

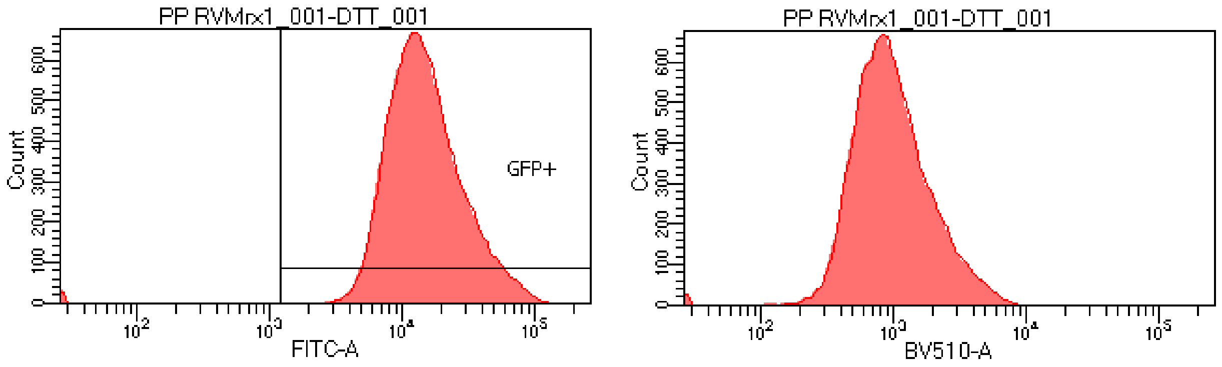

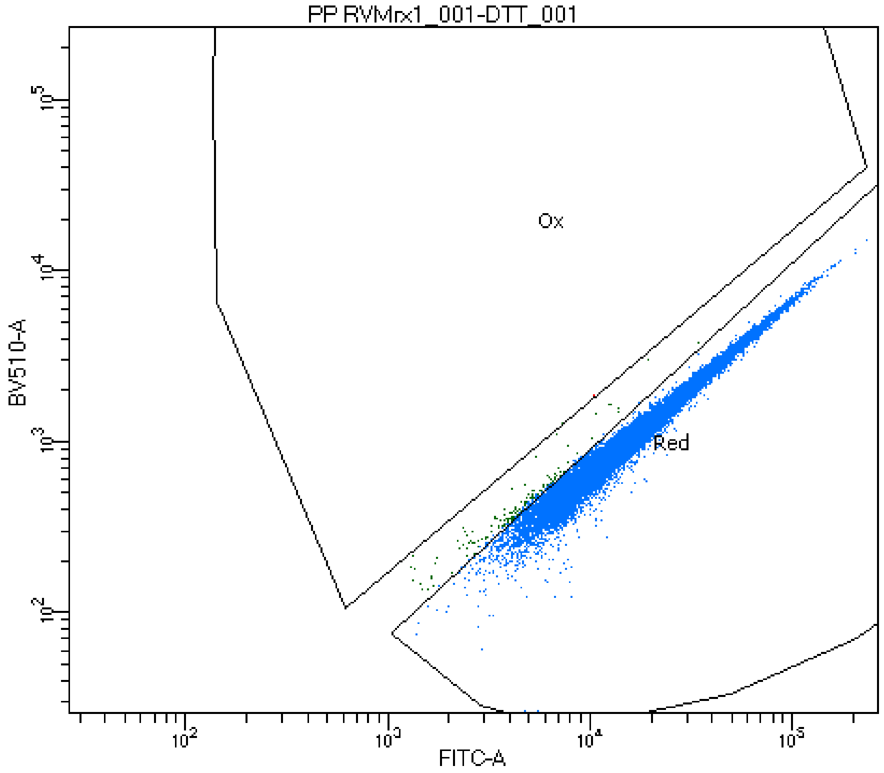

Tube: DTT_001

Population	#Events	%Parent	%Total
All Events	34,653	####	100.0
P	31,003	89.5	89.5
S1	30,575	98.6	88.2
S2	30,331	99.2	87.5
S3	29,303	96.6	84.6
S4	28,108	95.9	81.1
GFP+	27,834	99.0	80.3
Ox	1	0.0	0.0
Red	27,687	99.5	79.9

Experiment Name: 31Jan2017 Bac sorting
Specimen Name: PP RVMrx1_001
Tube Name: DTT_001
Record Date: Jan 31, 2017 2:57:10 PM
$OP: Administrator
GUID: d32b9670-0c53-4d5a-a155-20fbb02...

Population	#Events	%Parent	FITC-A Median	BV510-A Median
S4	28,108	95.9	13,441	869
GFP+	27,834	99.0	13,540	875
Ox	1	0.0	10,401	1,873
Red	27,687	99.5	13,600	878

BD FACSDiva 8.0.1

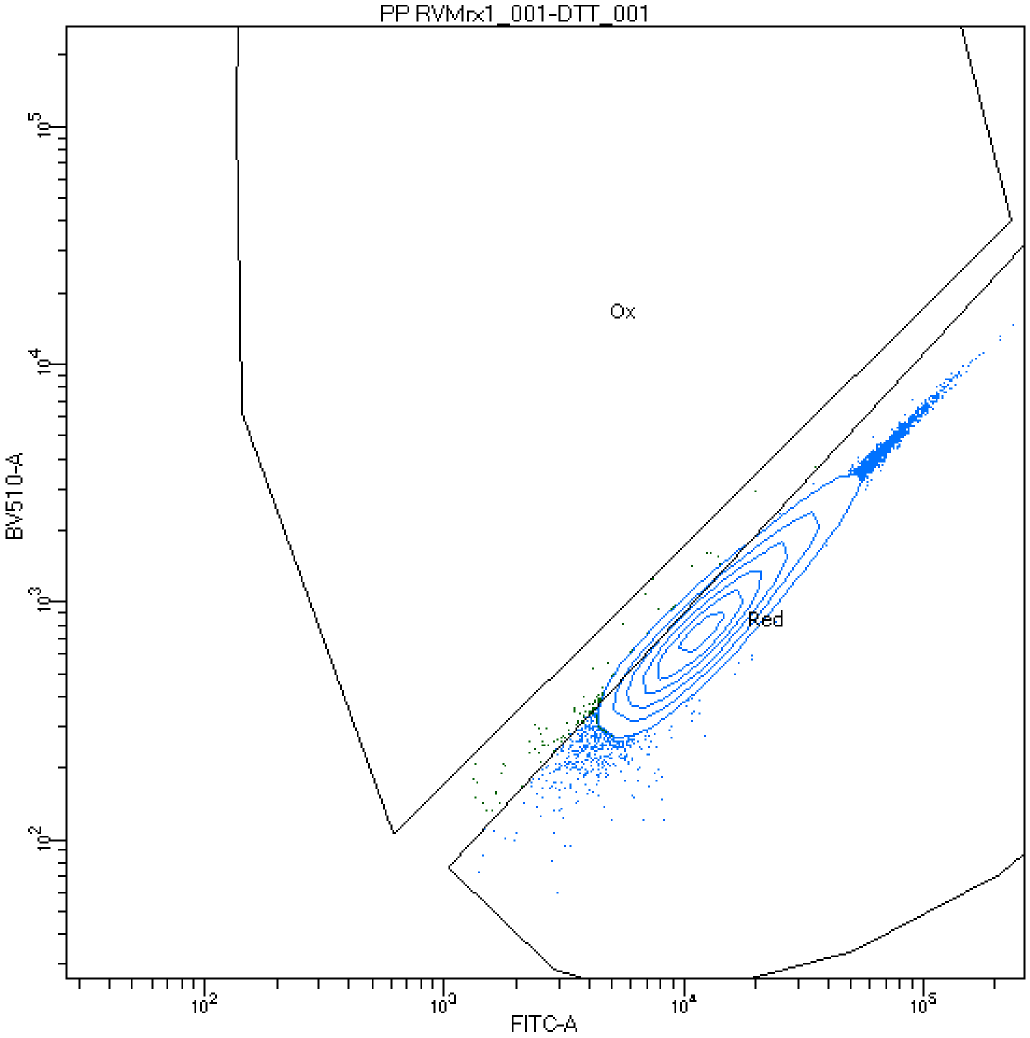

BD FACSDiva 8.0.1

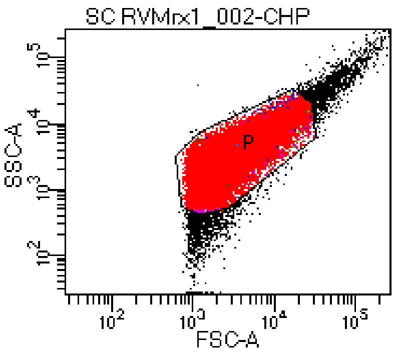

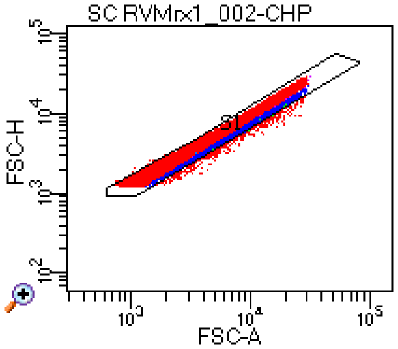

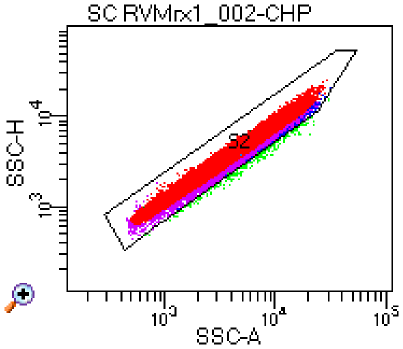

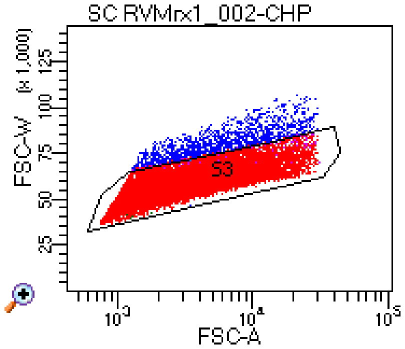

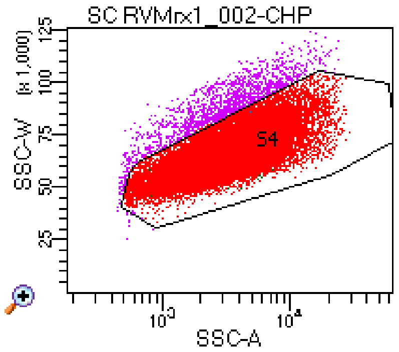

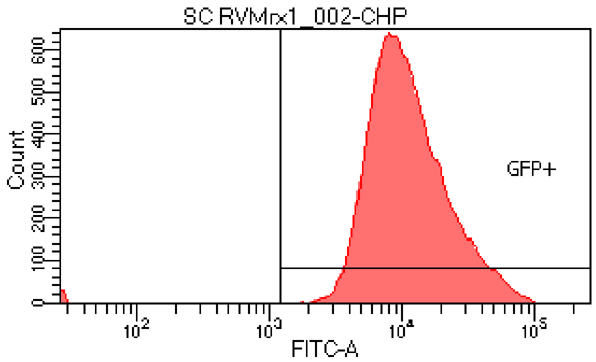

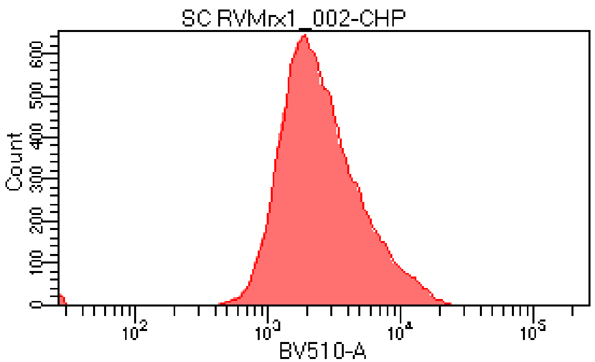

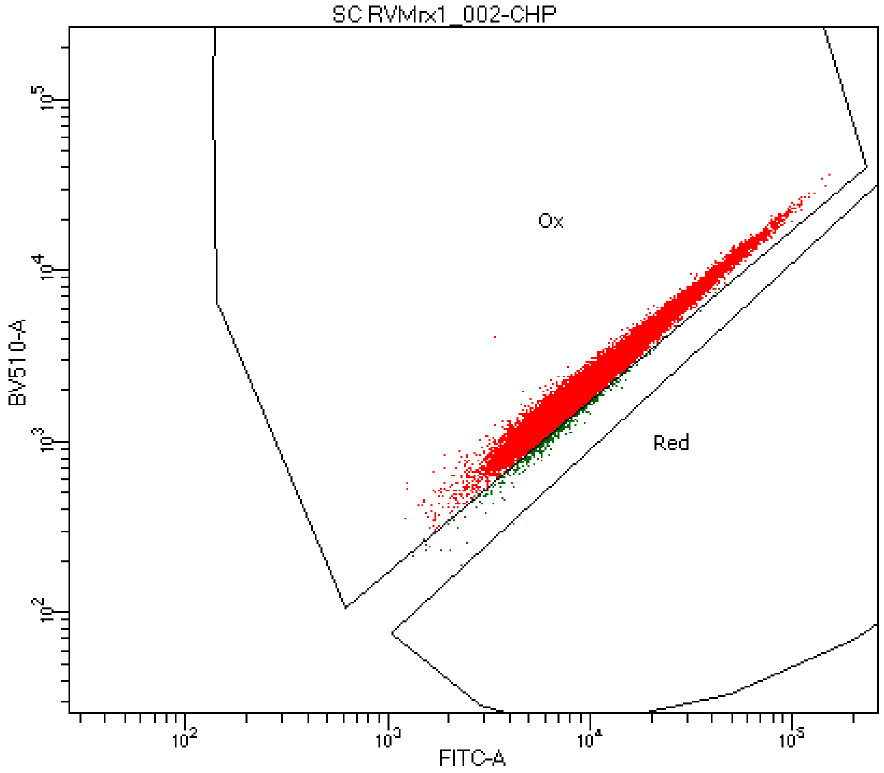

Tube: CHP

Population	#Events	%Parent	%Total
All Events	33,467	####	100.0
P	30,906	92.3	92.3
S1	30,351	98.2	90.7
S2	29,993	98.8	89.6
S3	28,633	95.5	85.6
S4	27,000	94.3	80.7
GFP+	26,842	99.4	80.2
Ox	26,199	97.6	78.3
Red	1	0.0	0.0

Experiment Name: 31Jan2017 Bac sorting
Specimen Name: SC RVMrx1_002
Tube Name: CHP
Record Date: Jan 31, 2017 3:01:35 PM
$OP: Administrator
GUID: be779ee7-cc8f-4b7d-9d3e-4ad3c2d...

Population	#Events	%Parent	FITC-A Median	BV510-A Median
S4	27,000	94.3	9,903	2,207
GFP+	26,842	99.4	9,953	2,216
Ox	26,199	97.6	10,084	2,254
Red	1	0.0	4,114	331

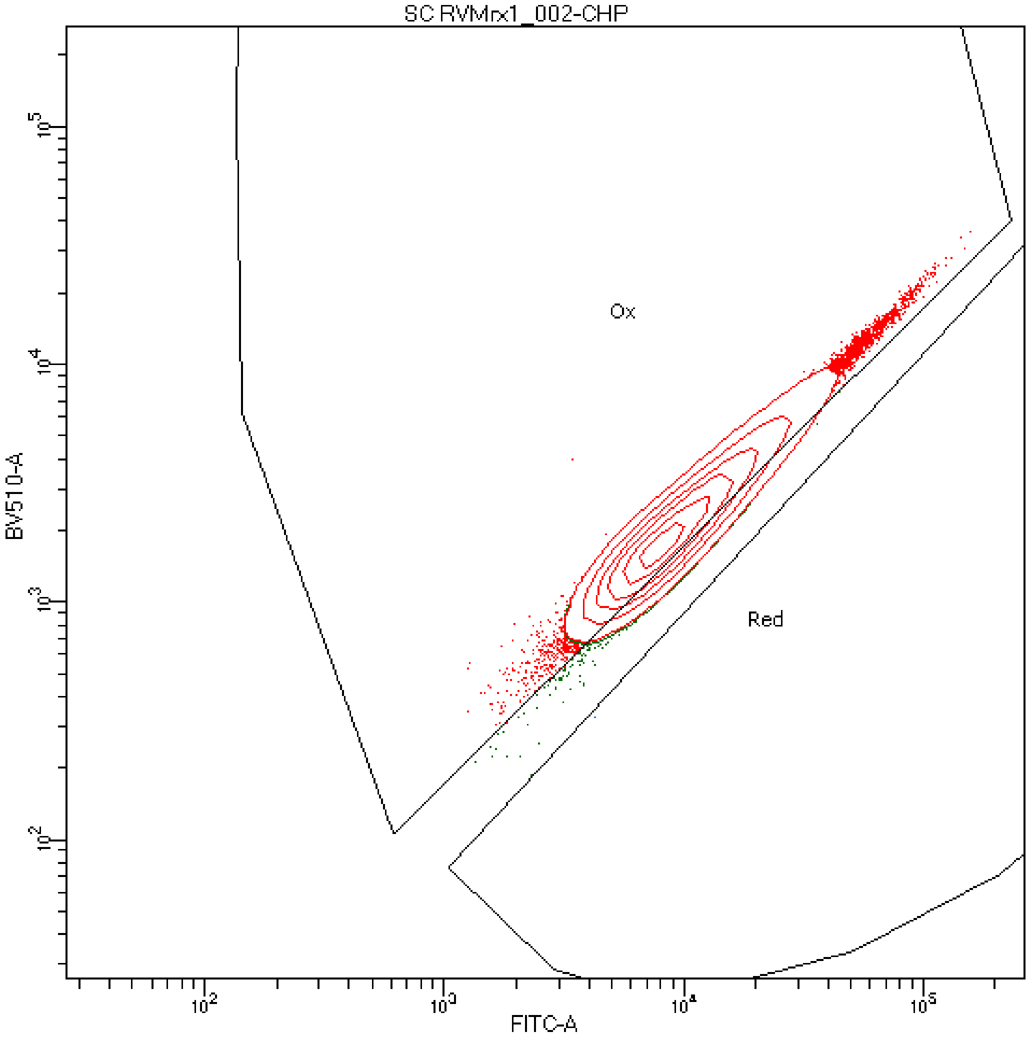
SC RVMrx1_002-CHP
Ox
Red
BV510-A
FITC-A
10^2
10^3
10^4
10^5

BD FACSDiva 8.0.1

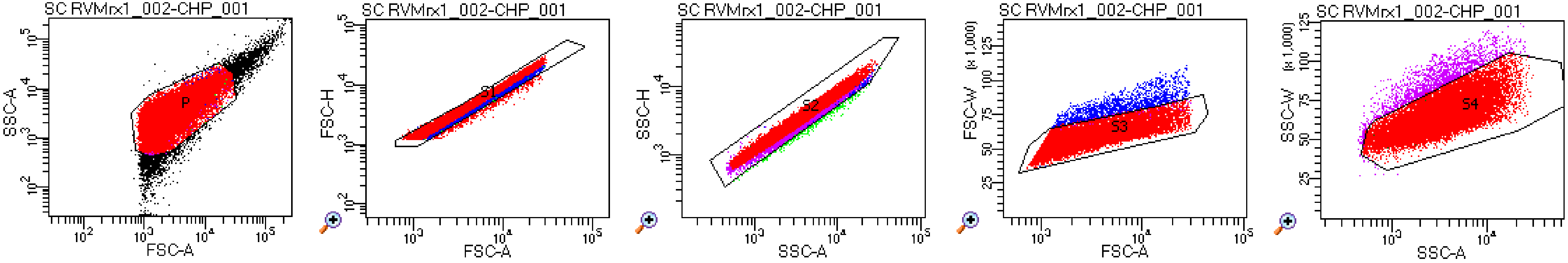

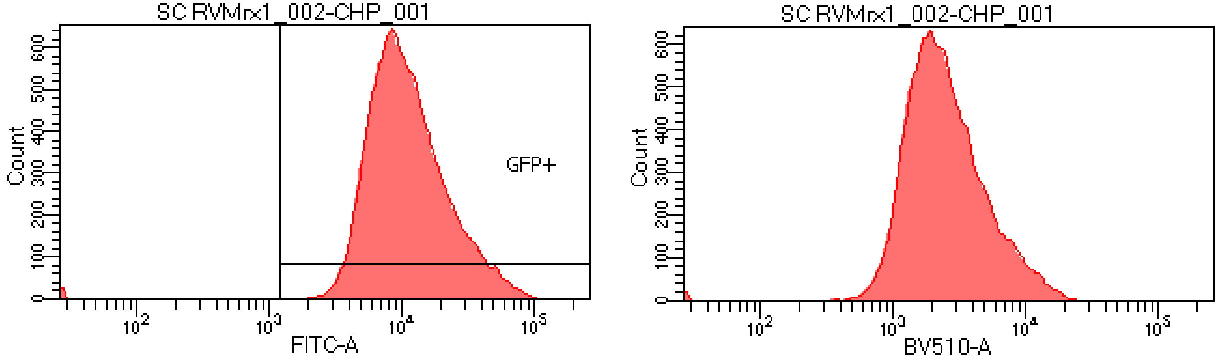

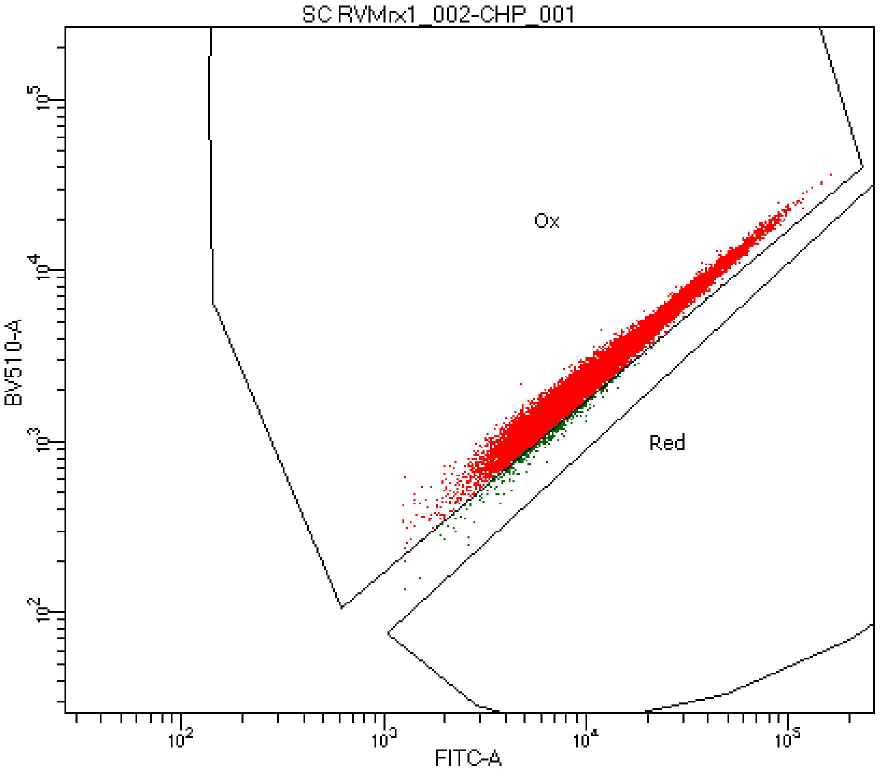

Tube: CHP_001

Population	#Events	%Parent	%Total
All Events	33,616	####	100.0
P	30,933	92.0	92.0
S1	30,384	98.2	90.4
S2	29,945	98.6	89.1
S3	28,535	95.3	84.9
S4	26,819	94.0	79.8
GFP+	26,655	99.4	79.3
Ox	26,128	98.0	77.7
Red	0	0.0	0.0

Experiment Name: 31Jan2017 Bac sorting
Specimen Name: SC RVMrx1_002
Tube Name: CHP_001
Record Date: Jan 31, 2017 3:02:06 PM
$OP: Administrator
GUID: 79244162-01c7-4099-b6f3-b9ab3ae...

Population	#Events	%Parent	FITC-A Median	BV510-A Median
S4	26,819	94.0	9,856	2,209
GFP+	26,655	99.4	9,911	2,220
Ox	26,128	98.0	10,016	2,250
Red	0	0.0	####	####

BD FACSDiva 8.0.1

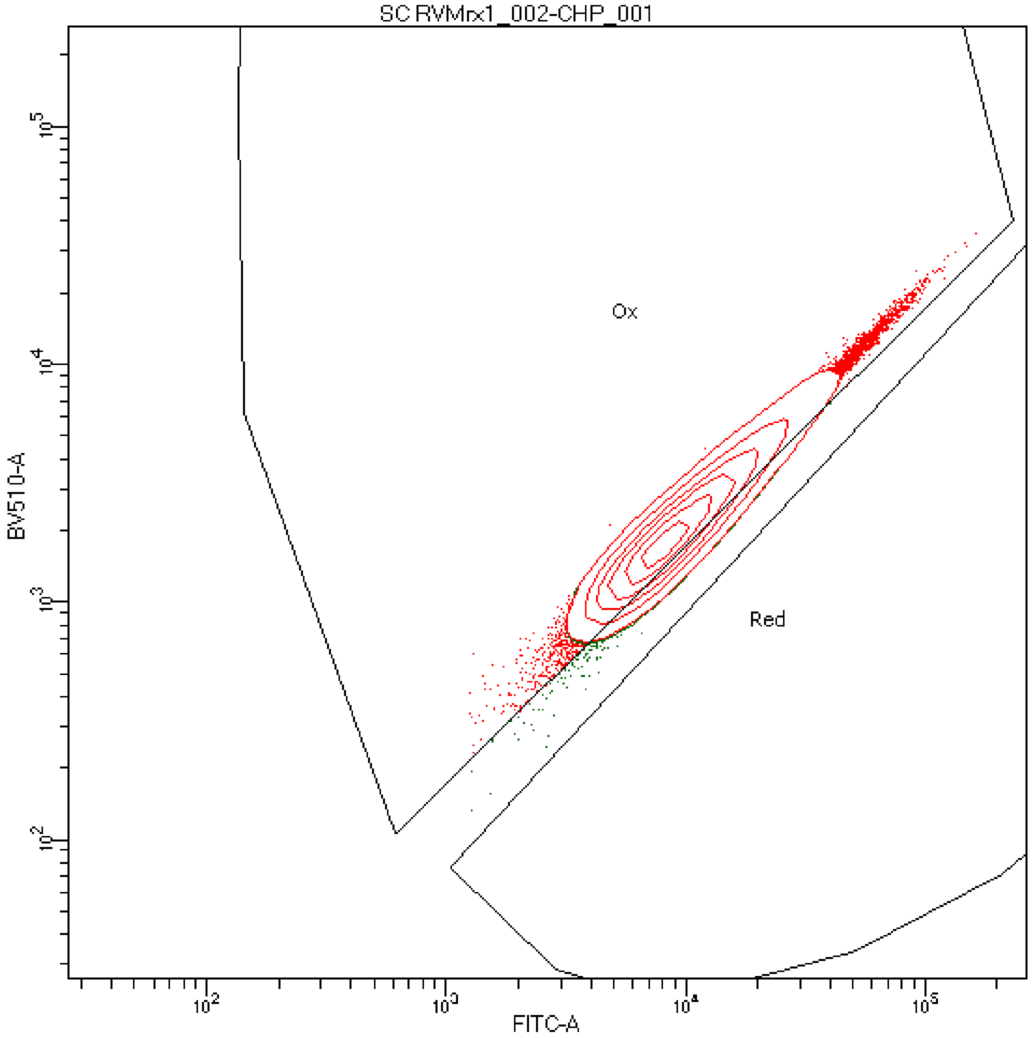

BD FACSDiva 8.0.1

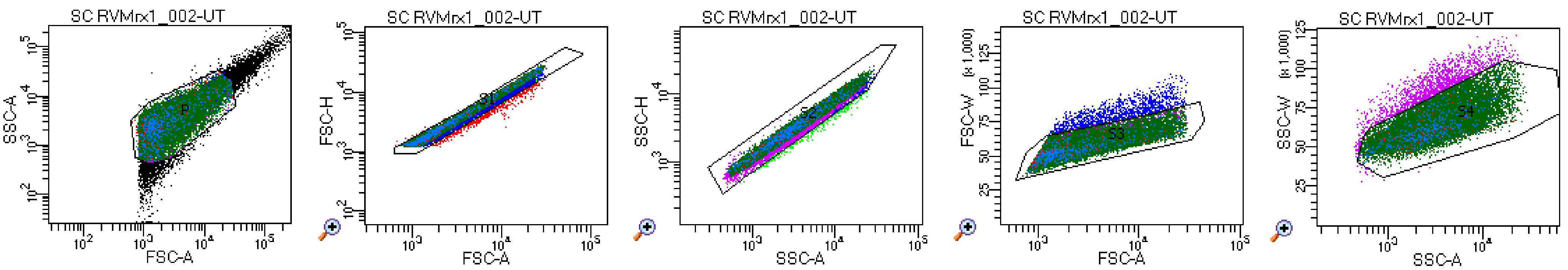

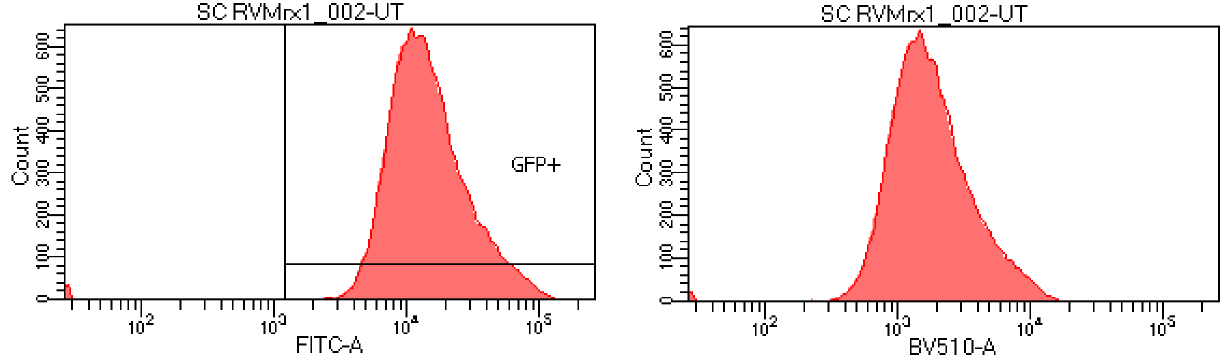

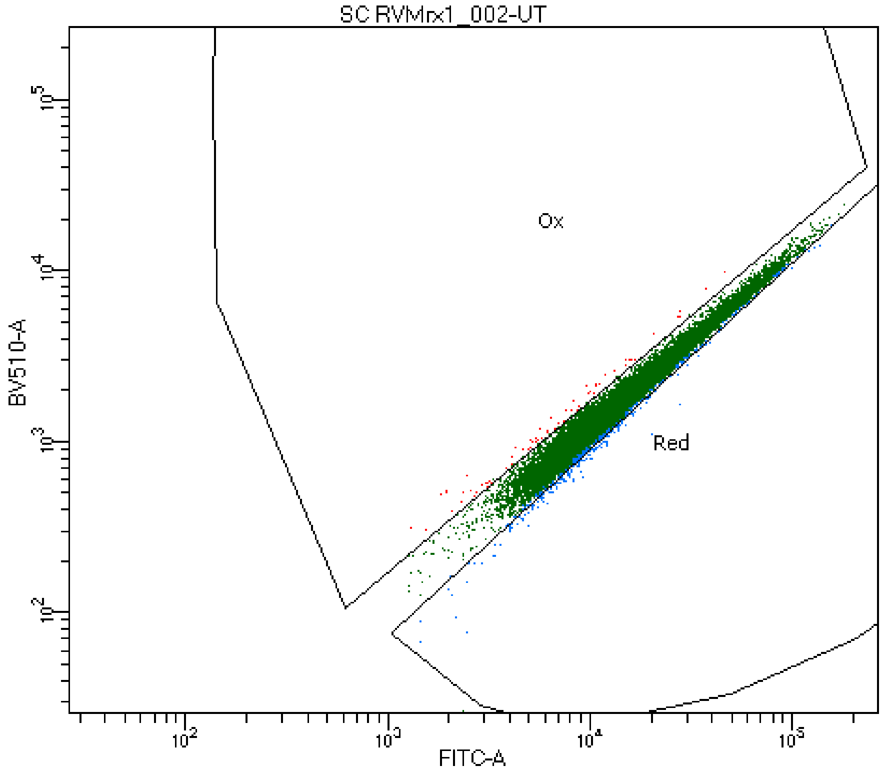

Tube: UT

Population	#Events	%Parent	%Total
All Events	33,599	####	100.0
P	30,896	92.0	92.0
S1	30,349	98.2	90.3
S2	29,979	98.8	89.2
S3	28,705	95.8	85.4
S4	27,167	94.6	80.9
GFP+	27,003	99.4	80.4
Ox	64	0.2	0.2
Red	507	1.9	1.5

Experiment Name: 31Jan2017 Bac sorting
Specimen Name: SC RVMrx1_002
Tube Name: UT
Record Date: Jan 31, 2017 3:02:38 PM
$OP: Administrator
GUID: 52b09284-8da2-4f00-96a6-278a802...

Population	#Events	%Parent	FITC-A Median	BV510-A Median
S4	27,167	94.6	13,001	1,577
GFP+	27,003	99.4	13,057	1,586
Ox	64	0.2	5,911	1,129
Red	507	1.9	11,009	998

BD FACSDiva 8.0.1

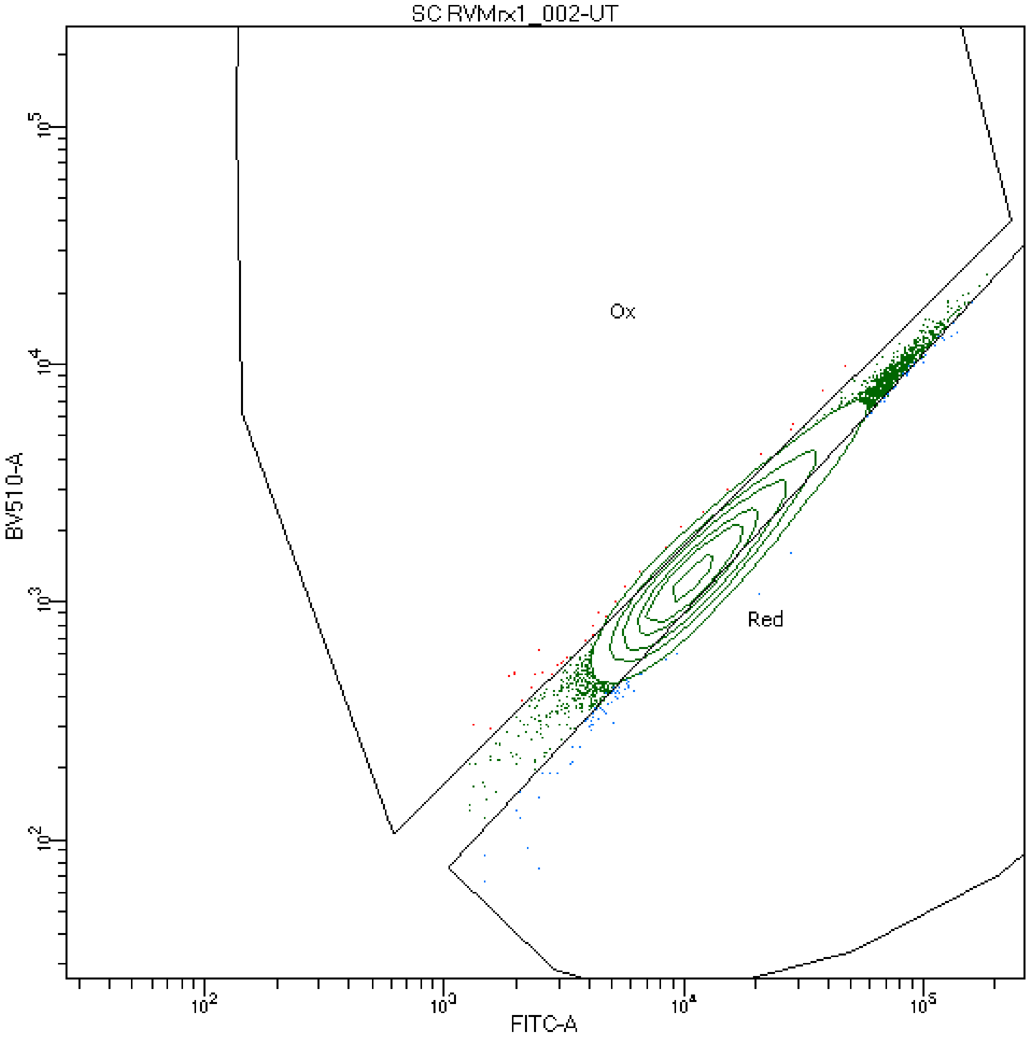

BD FACSDiva 8.0.1

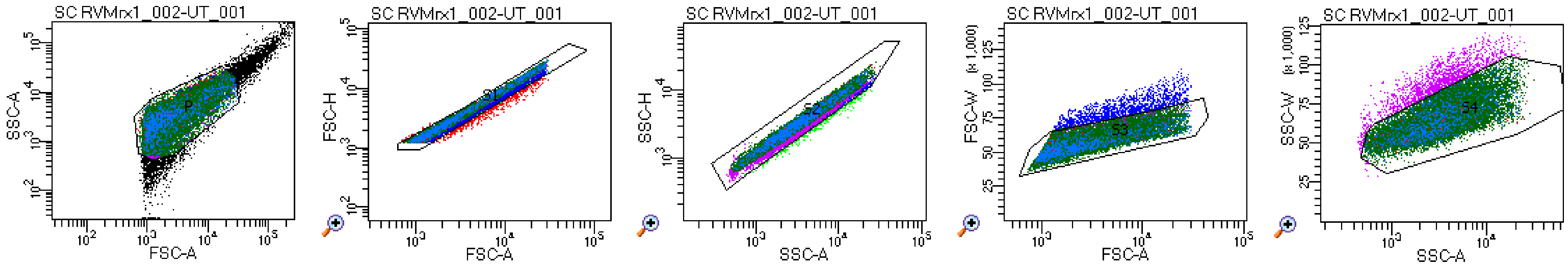

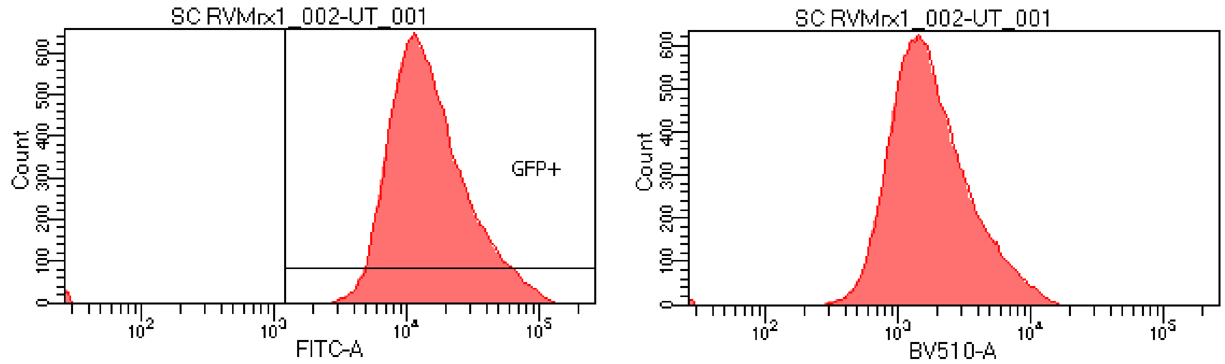

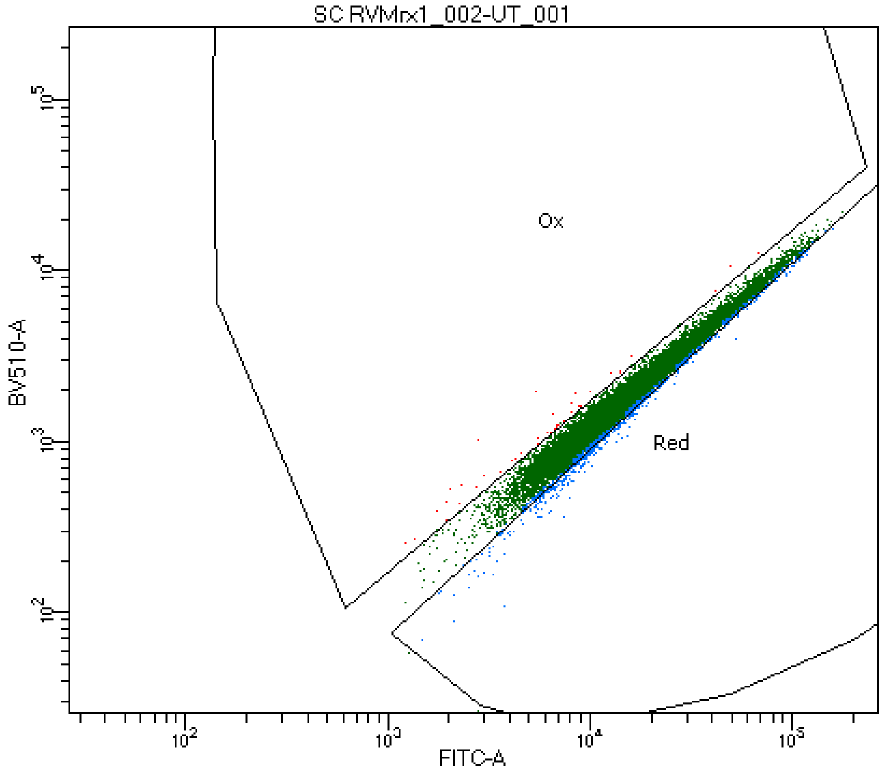

Tube: UT_001

Population	#Events	%Parent	%Total
All Events	33,646	####	100.0
P	30,881	91.8	91.8
S1	30,270	98.0	90.0
S2	29,912	98.8	88.9
S3	28,587	95.6	85.0
S4	26,993	94.4	80.2
GFP+	26,841	99.4	79.8
Ox	42	0.2	0.1
Red	1,008	3.8	3.0

Experiment Name:	31Jan2017 Bac sorting
Specimen Name:	SC RVMrx1_002
Tube Name:	UT_001
Record Date:	Jan 31, 2017 3:03:10 PM
$OP:	Administrator
GUID:	e84988a1-341b-4af2-8572-1028f82...

Population	#Events	%Parent	FITC-A Median	BV510-A Median
S4	26,993	94.4	13,161	1,558
GFP+	26,841	99.4	13,225	1,566
Ox	42	0.2	6,447	1,181
Red	1,008	3.8	12,816	1,167

BD FACSDiva 8.0.1

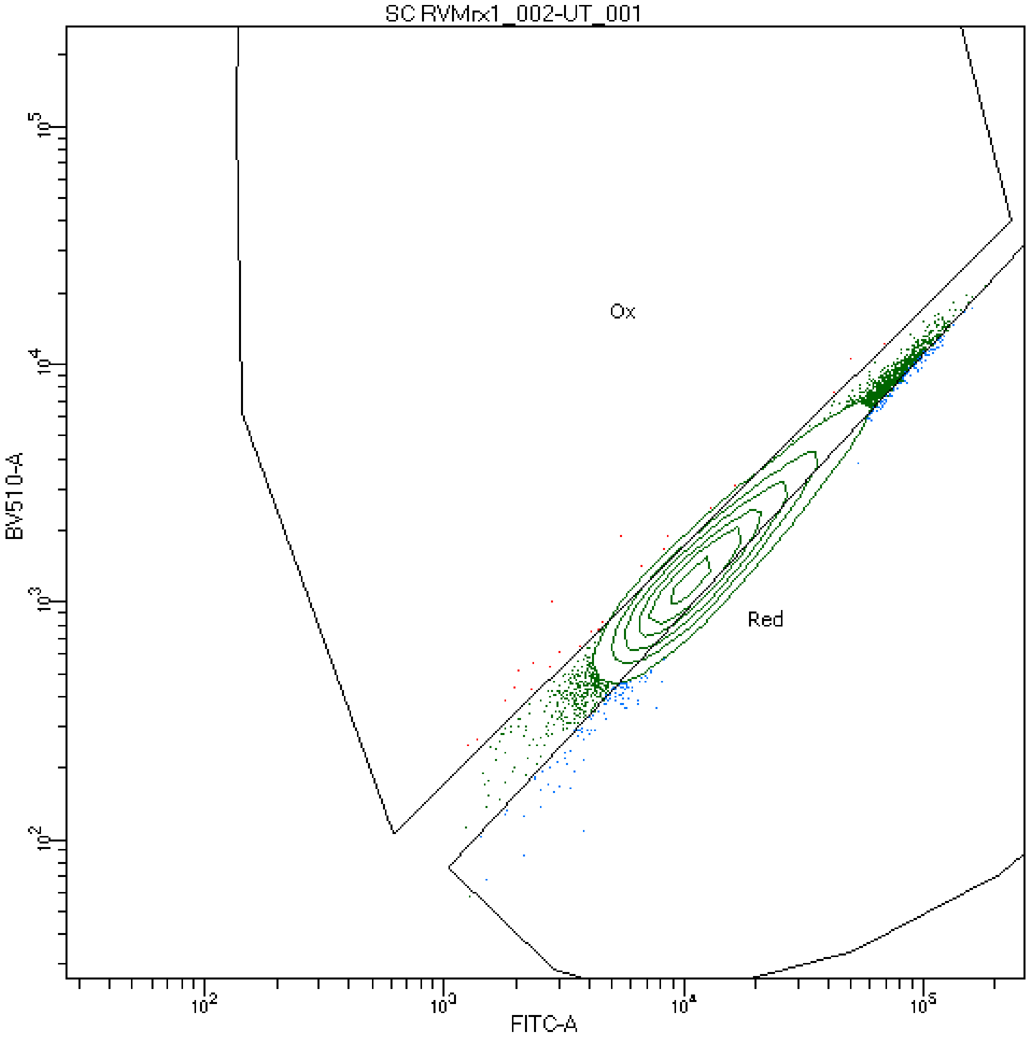

BD FACSDiva 8.0.1

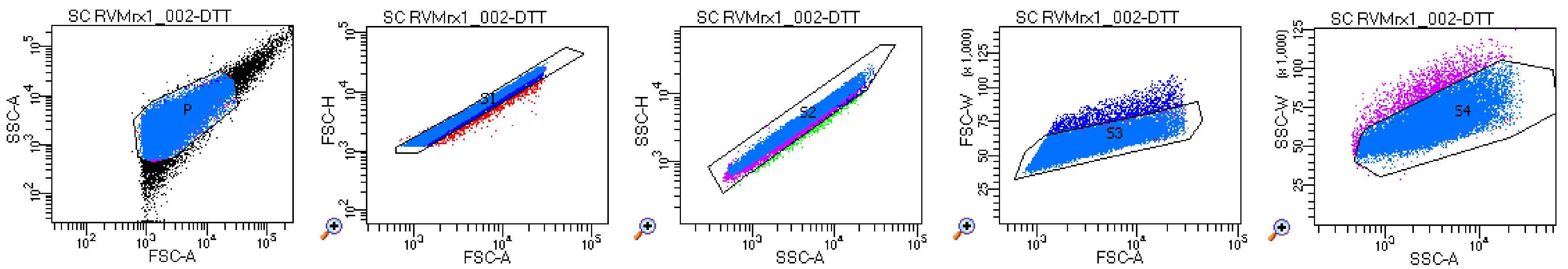

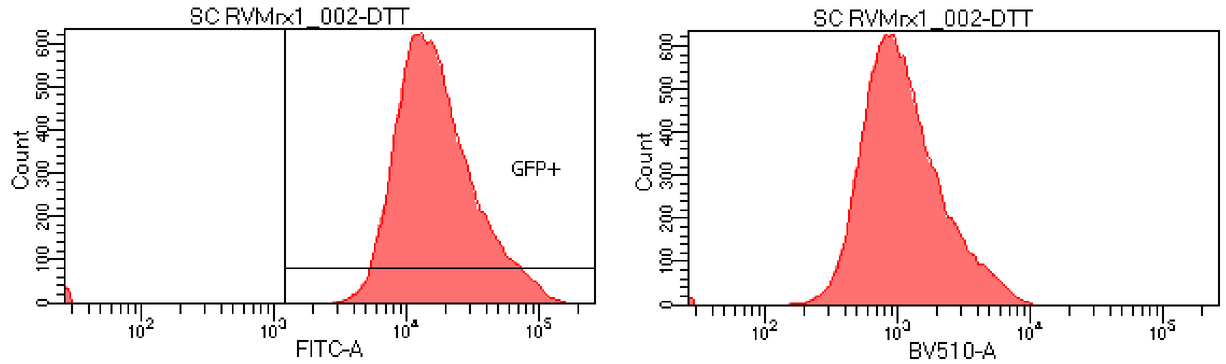

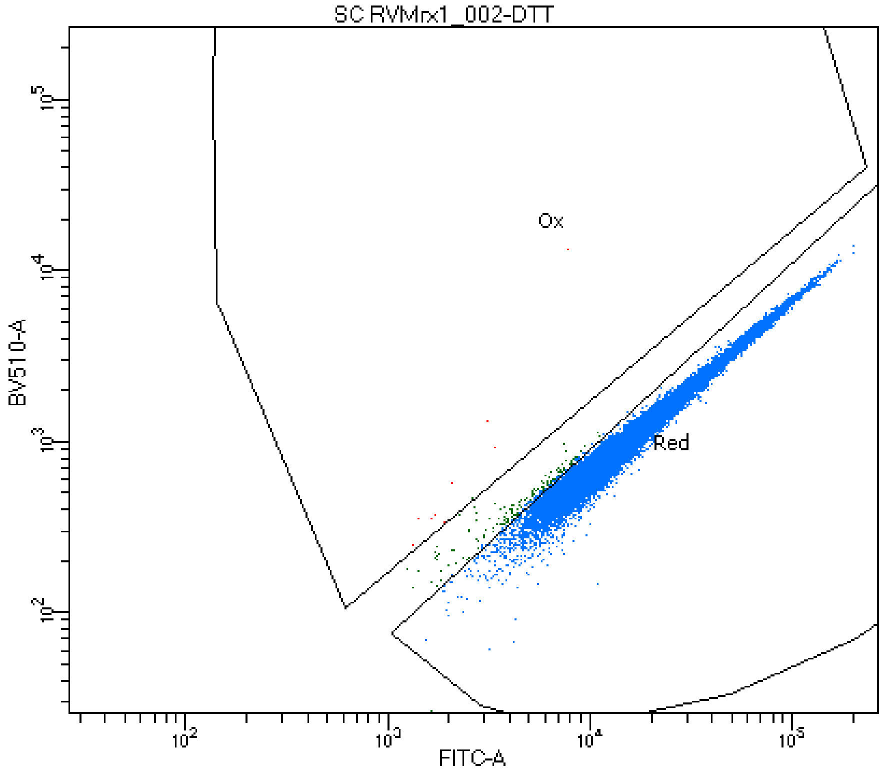

Tube: DTT

Population	#Events	%Parent	%Total
All Events	33,815	####	100.0
P	30,972	91.6	91.6
S1	30,357	98.0	89.8
S2	29,960	98.7	88.6
S3	28,506	95.1	84.3
S4	26,951	94.5	79.7
GFP+	26,785	99.4	79.2
Ox	9	0.0	0.0
Red	26,626	99.4	78.7

Experiment Name: 31Jan2017 Bac sorting
Specimen Name: SC RVMrx1_002
Tube Name: DTT
Record Date: Jan 31, 2017 3:00:24 PM
$OP: Administrator
GUID: 92d65dd3-fd3a-4941-892a-59831eb...

Population	#Events	%Parent	FITC-A Median	BV510-A Median
S4	26,951	94.5	14,964	969
GFP+	26,785	99.4	15,047	974
Ox	9	0.0	1,887	375
Red	26,626	99.4	15,118	979

BD FACSDiva 8.0.1

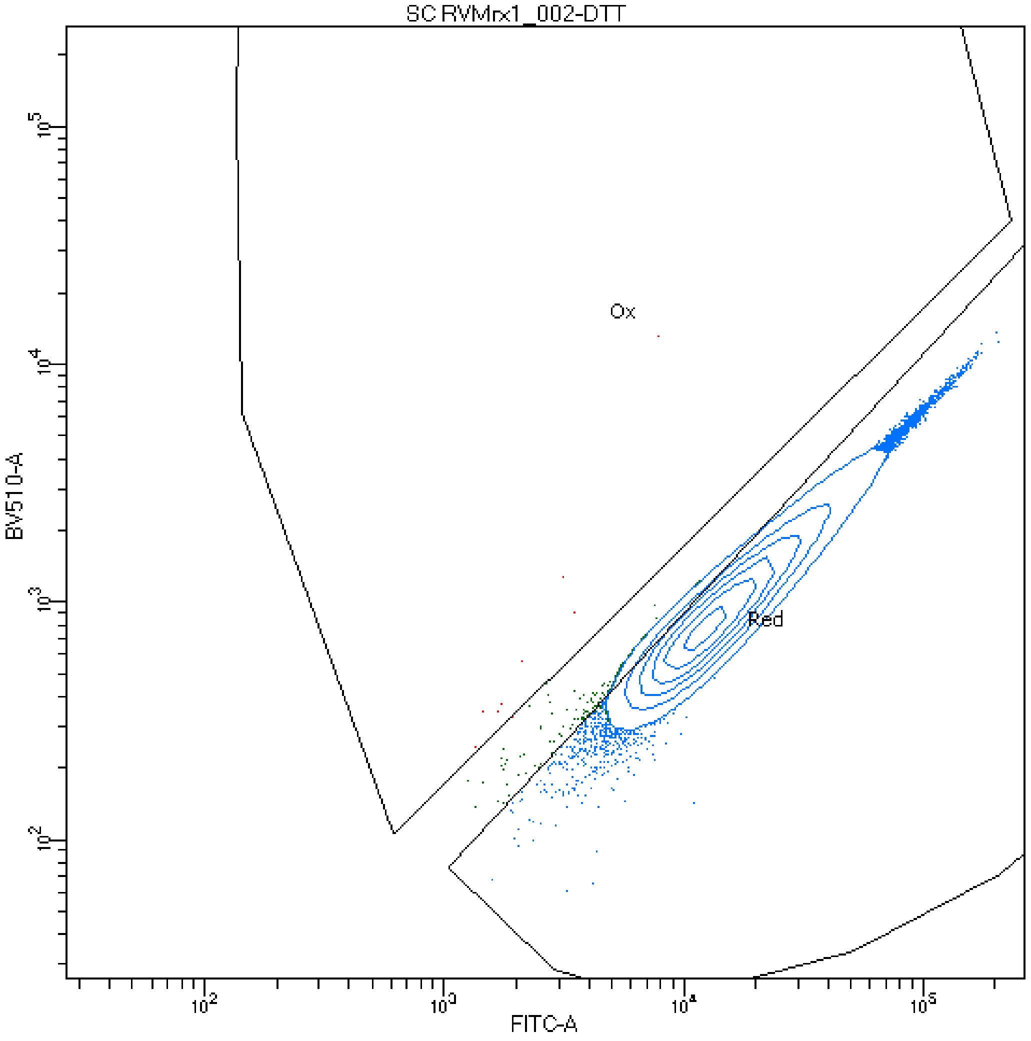

BD FACSDiva 8.0.1

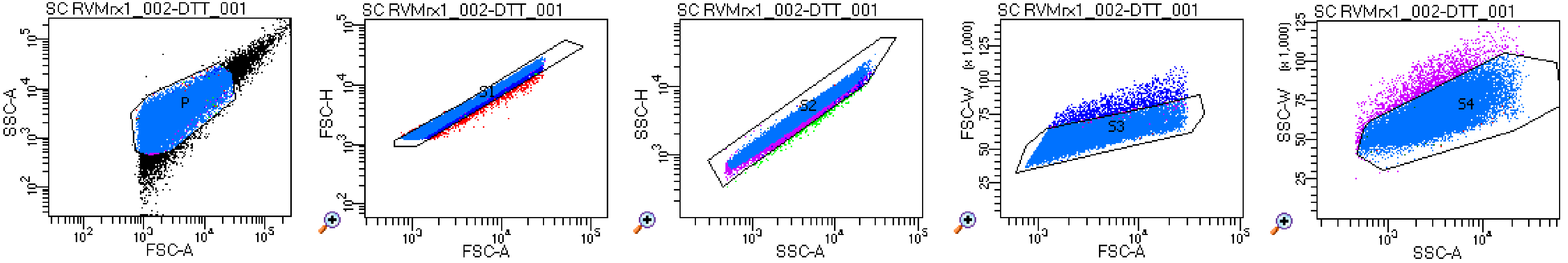

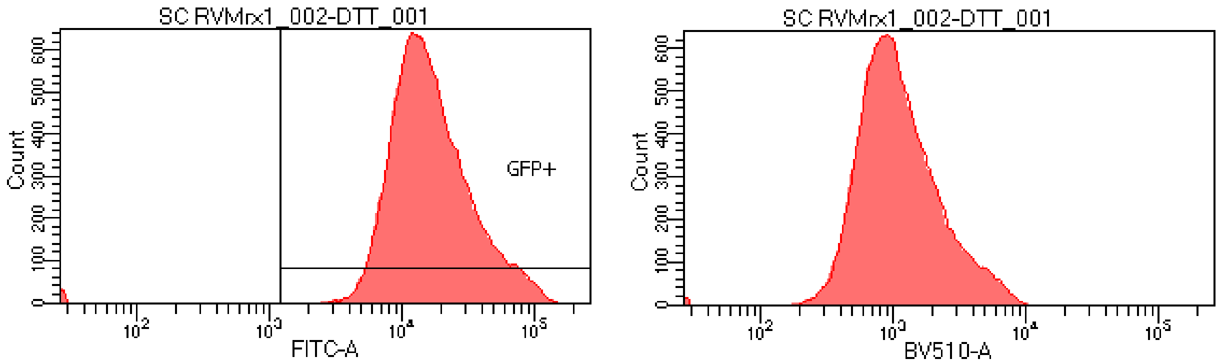

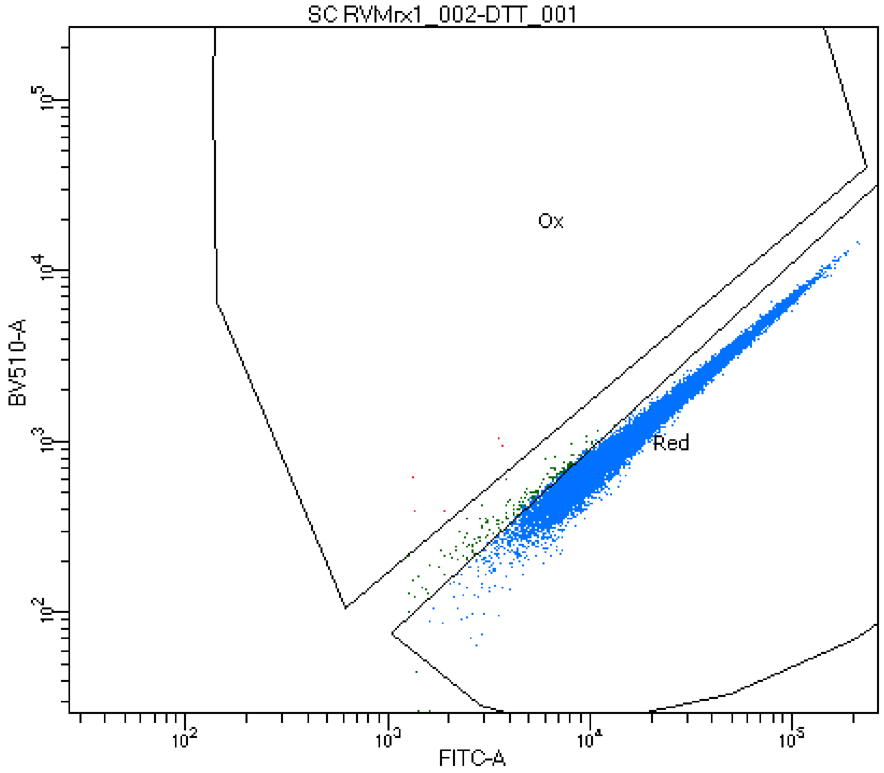

Tube: DTT_001

Population	#Events	%Parent	%Total
All Events	33,833	####	100.0
P	31,038	91.7	91.7
S1	30,416	98.0	89.9
S2	30,039	98.8	88.8
S3	28,633	95.3	84.6
S4	27,027	94.4	79.9
GFP+	26,885	99.5	79.5
Ox	5	0.0	0.0
Red	26,696	99.3	78.9

Experiment Name:	31Jan2017 Bac sorting
Specimen Name:	SC RVMrx1_002
Tube Name:	DTT_001
Record Date:	Jan 31, 2017 3:00:56 PM
$OP:	Administrator
GUID:	90cd5d7b-22a2-4787-8681-a94b9c...

Population	#Events	%Parent	FITC-A Median	BV510-A Median
S4	27,027	94.4	14,749	972
GFP+	26,885	99.5	14,819	976
Ox	5	0.0	1,896	611
Red	26,696	99.3	14,900	981

BD FACSDiva 8.0.1

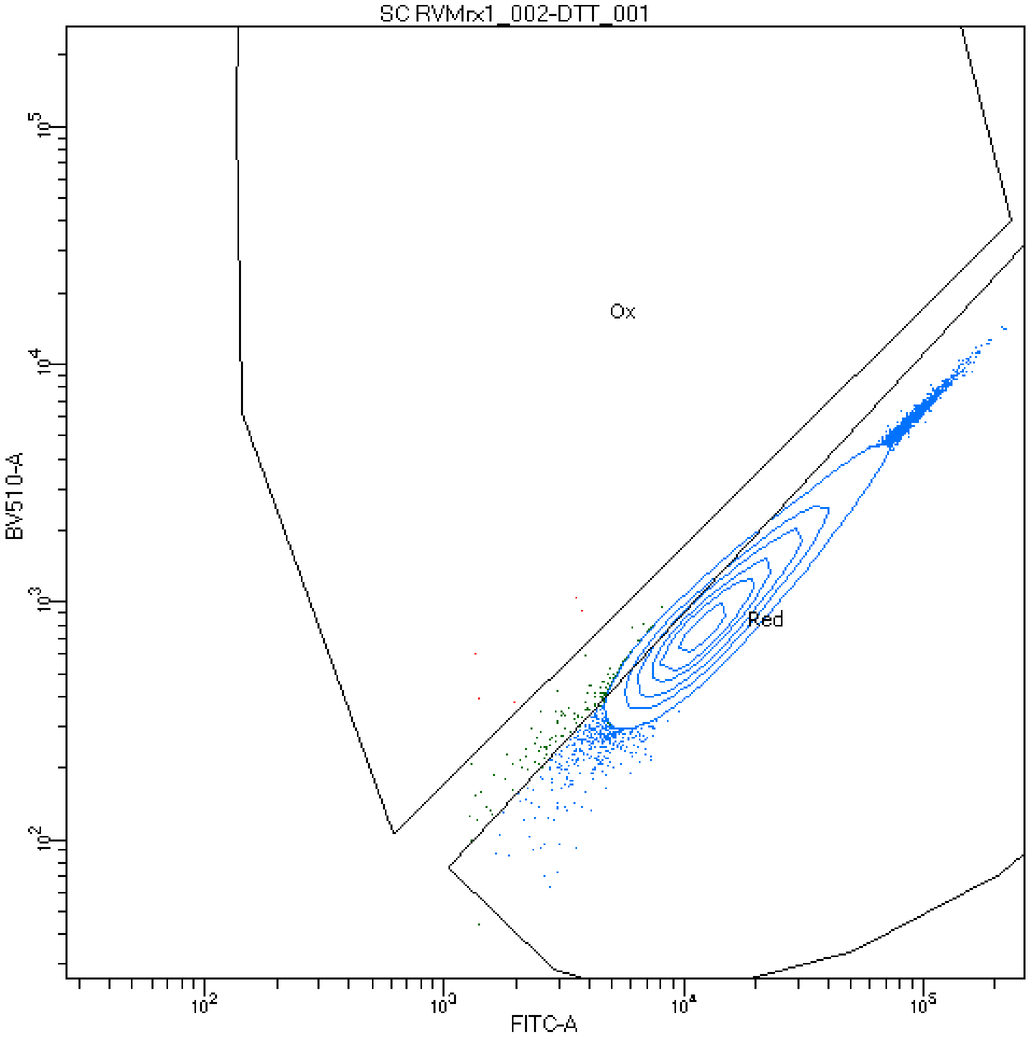

Supplement: Figure 1—source data 1. [file elife-80218-fig1-data1.zip › Round 3 Sorting/31Jan2017 Bac sorting-Batch_Analysis_28022017125550.pdf]
