# Supplementary material for: Biosensor-integrated transposon mutagenesis reveals rv0158 as a coordinator of redox homeostasis in Mycobacterium tuberculosis
Source: eLife. 2023 Aug 29;12:e80218. doi: 10.7554/eLife.80218 (PMC10501769; doi:10.7554/eLife.80218)
Supplement: Figure 1—source data 1. [file elife-80218-fig1-data1.zip › Round 3 Sorting/Sort_Report_06122016162724.pdf]

Experiment : 05EDec2016 Bac sorting	Sort Report	Report Date : 2016.12.06 at 16:21:26
Specimen : 06Dec		Device : 2 Tube
Tube : TN lib		User ID : Administrator
Sort Layout : Sort Layout_003		Cytometer : FACSAriaIII (P65828254001)
Application : FACSDiva Version 8.0.1		

Sort Settings

Sort Setup	70 micron	Precision	Yield
Frequency	88.4	Yield Mask	32
Amplitude	4.3	Purity Mask	0
Phase	0.00	Phase Mask	0
Drop Delay	44.62	Single Cell	Off
Attenuation	Off	Plates Voltage	5,500
Sweet Spot	On	Voltage Centering	6
First Drop	208	Sheath Pressure	70.00
Target Gap	6		

Side Stream Voltage (%)

Far Left	Left	Right	Far Right
0.00	41.00	34.00	0.00

Neighboring Drop Charge (%)

2nd	3rd	4th
18.00	8.00	0.00

Acquisition Counters

Threshold Count	61812829
Processed Events Count(evt)	33526841
Electronic Aborts Count(evt)	586834
Sort Elapsed Time(hh:mm:ss)	01:57:48

Sort Counters

	Left	Right
Sort Rate(evt/s)	235	4
Conflicts Count(evt)	0	0
Conflicts Rate(evt/s)	0	0
Efficiency(%)	100	100

Sort Layout

Left	Right
Ox : 1665294	Red : 35320
