# Supplementary material for: Biosensor-integrated transposon mutagenesis reveals rv0158 as a coordinator of redox homeostasis in Mycobacterium tuberculosis
Source: eLife. 2023 Aug 29;12:e80218. doi: 10.7554/eLife.80218 (PMC10501769; doi:10.7554/eLife.80218)
Supplement: Figure 1—source data 1. [file elife-80218-fig1-data1.zip › Round 3 Sorting/Sort_Report_31012017165601.pdf]

Experiment : Bac sorting	Sort Report	Report Date : 2017.01.31 at 16:49:37
Specimen : SS RVMrx1		Device : 2 Tube
Tube : TN lib ox		User ID : Administrator
Sort Layout : Sort Layout_004		Cytometer : FACSAriaIII (P65828254001)
Application : FACSDiva Version 8.0.1		

Sort Settings

Sort Setup	70 micron	Precision	4-Way Purity
Frequency	88.9	Yield Mask	0
Amplitude	5.3	Purity Mask	32
Phase	0.00	Phase Mask	0
Drop Delay	44.83	Single Cell	Off
Attenuation	Off	Plates Voltage	5,500
Sweet Spot	On	Voltage Centering	6
First Drop	200	Sheath Pressure	70.00
Target Gap	6		

Side Stream Voltage (%)

Far Left	Left	Right	Far Right
0.00	39.00	0.00	0.00

Neighboring Drop Charge (%)

2nd	3rd	4th
18.00	8.00	0.00

Acquisition Counters

Threshold Count	98355662
Processed Events Count(evt)	97649504
Electronic Aborts Count(evt)	2426413
Sort Elapsed Time(hh:mm:ss)	01:32:51

Sort Counters

	Left	Right
Sort Rate(evt/s)	0	NA
Conflicts Count(evt)	1566708	NA
Conflicts Rate(evt/s)	0	NA
Efficiency(%)	0	NA

Sort Layout

Left	Right
Ox : 5526507	
