# Supplementary figures and images for: Biosensor-integrated transposon mutagenesis reveals rv0158 as a coordinator of redox homeostasis in Mycobacterium tuberculosis
Source: eLife. 2023 Aug 29;12:e80218. doi: 10.7554/eLife.80218 (PMC10501769; doi:10.7554/eLife.80218)

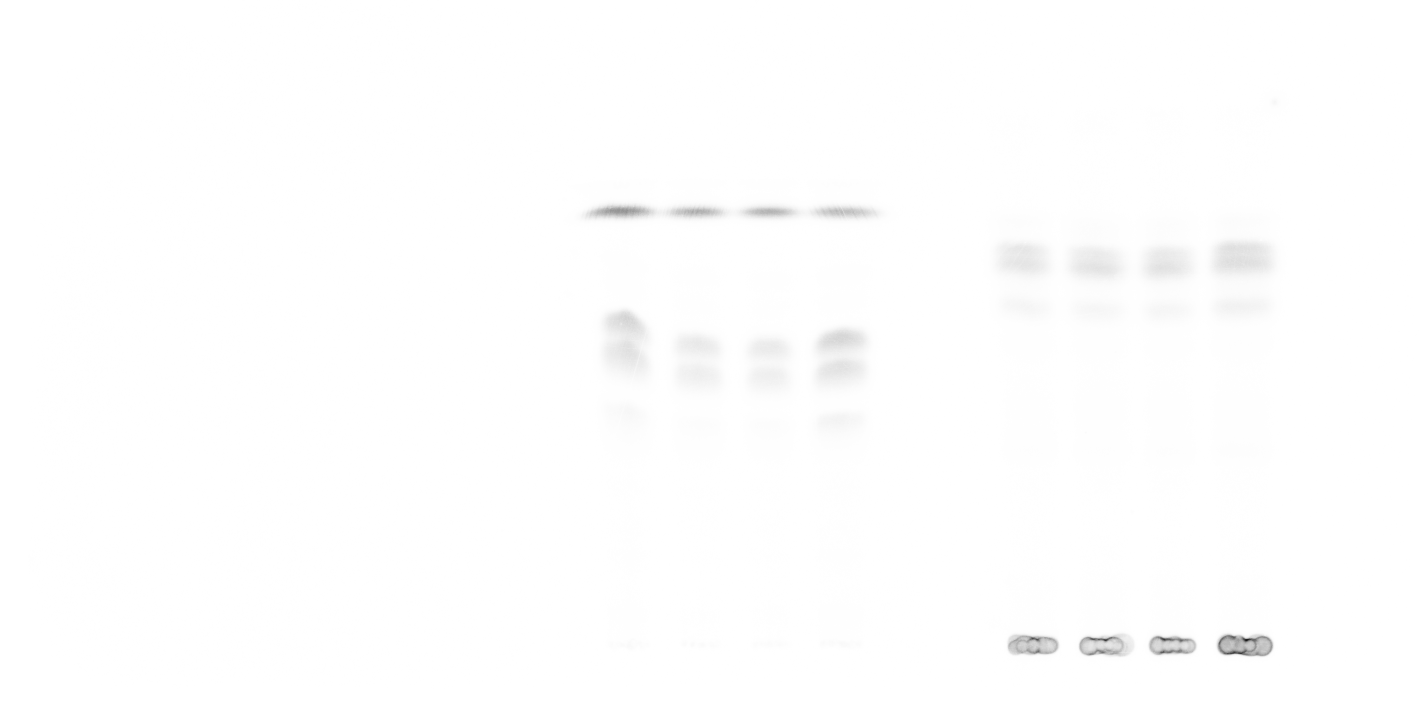


methoxy

MAMES

keto

α

*Rv0158* Comp

*Rv0158* KO

*WT Mtb*

Supplement: Figure 6—source data 1. [file elife-80218-fig6-data1.zip › Figure 6A-Source Data 6A labelled.docx]

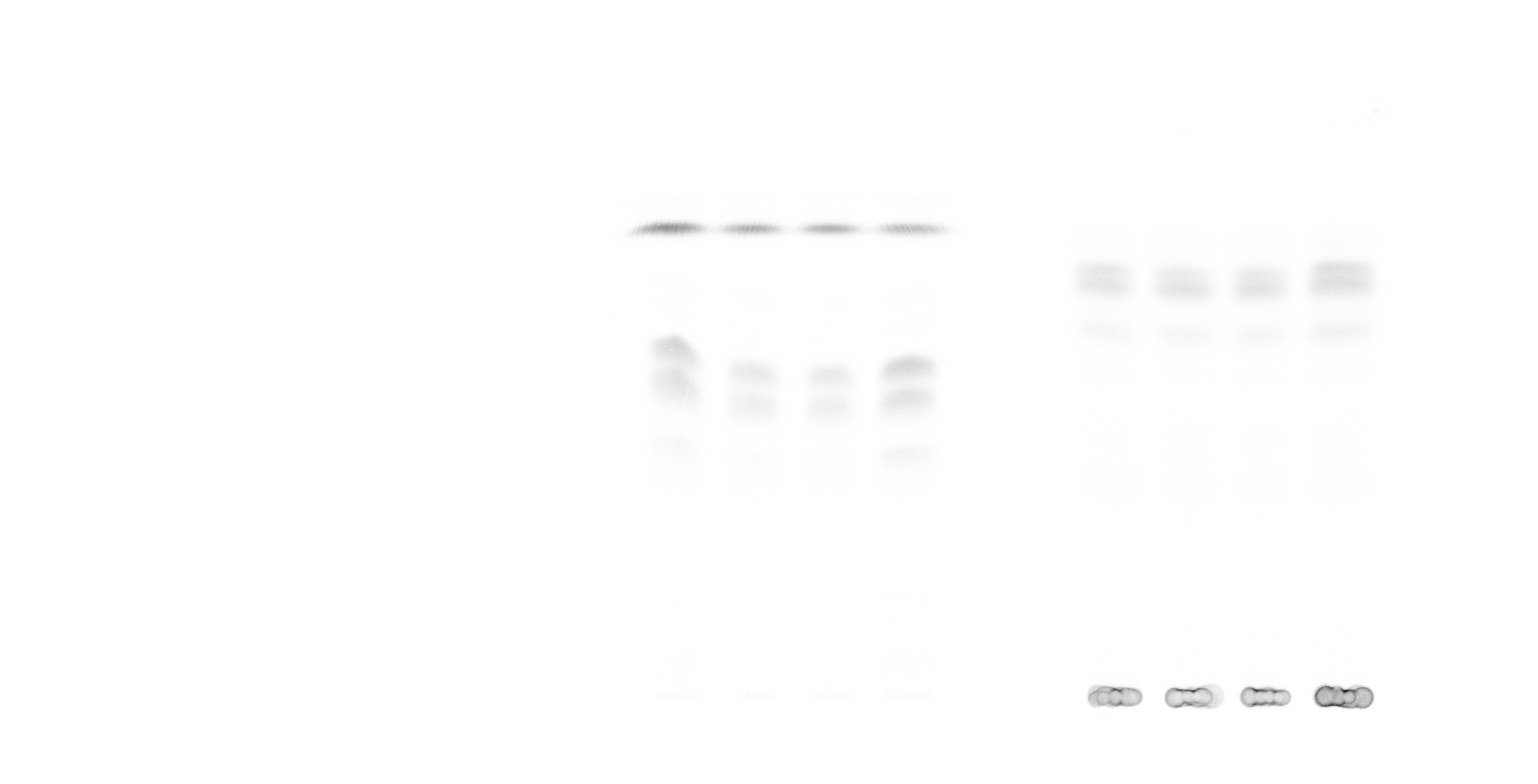

Supplement: Figure 6—source data 1. [file elife-80218-fig6-data1.zip › Figure 6A-Source Data 6A raw unedited.tif]

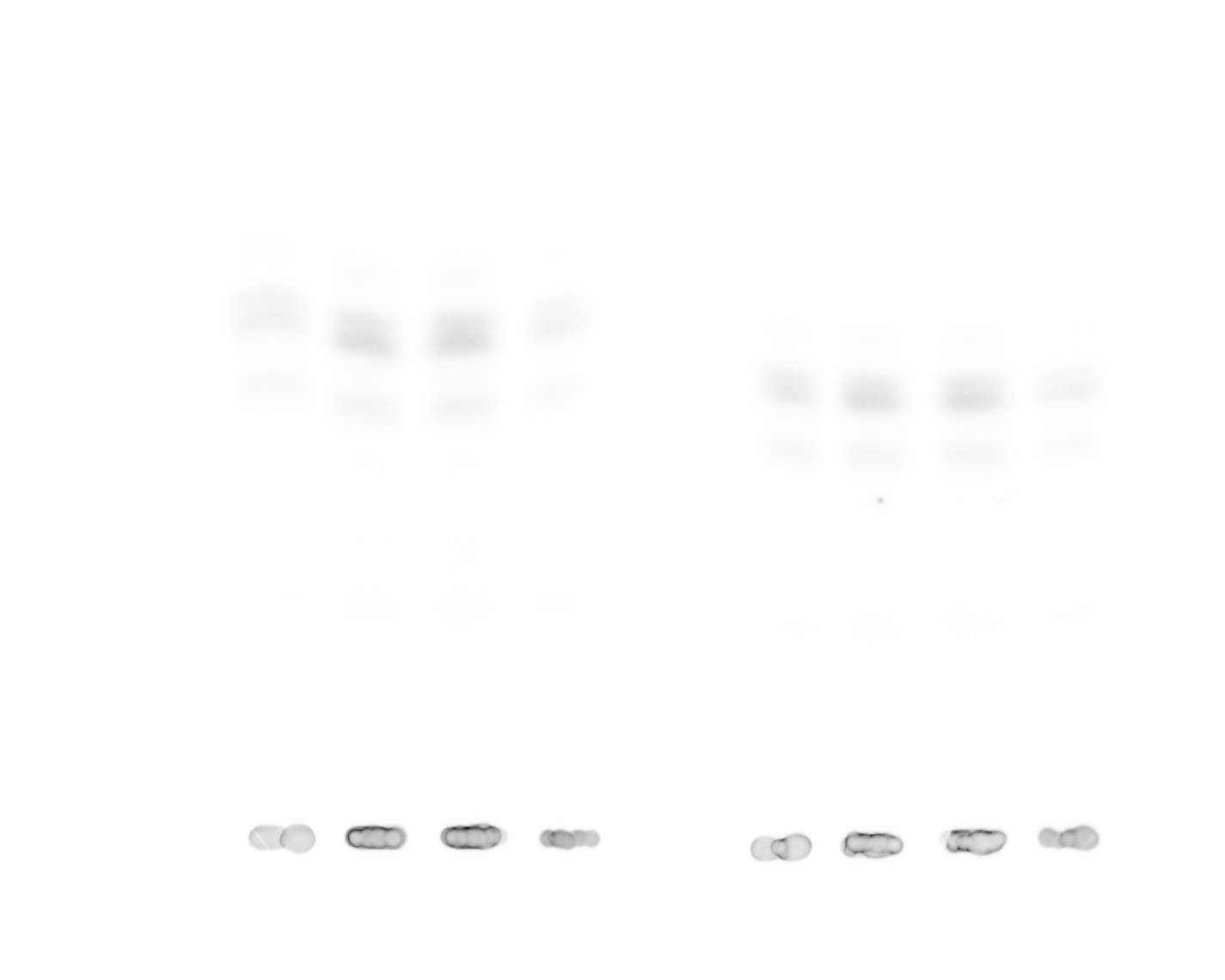


*Rv0158* Comp

*Rv0158* KO

*WT Mtb*

FAMES

Supplement: Figure 6—source data 1. [file elife-80218-fig6-data1.zip › Figure 6B-Source Data 6B labelled.docx]

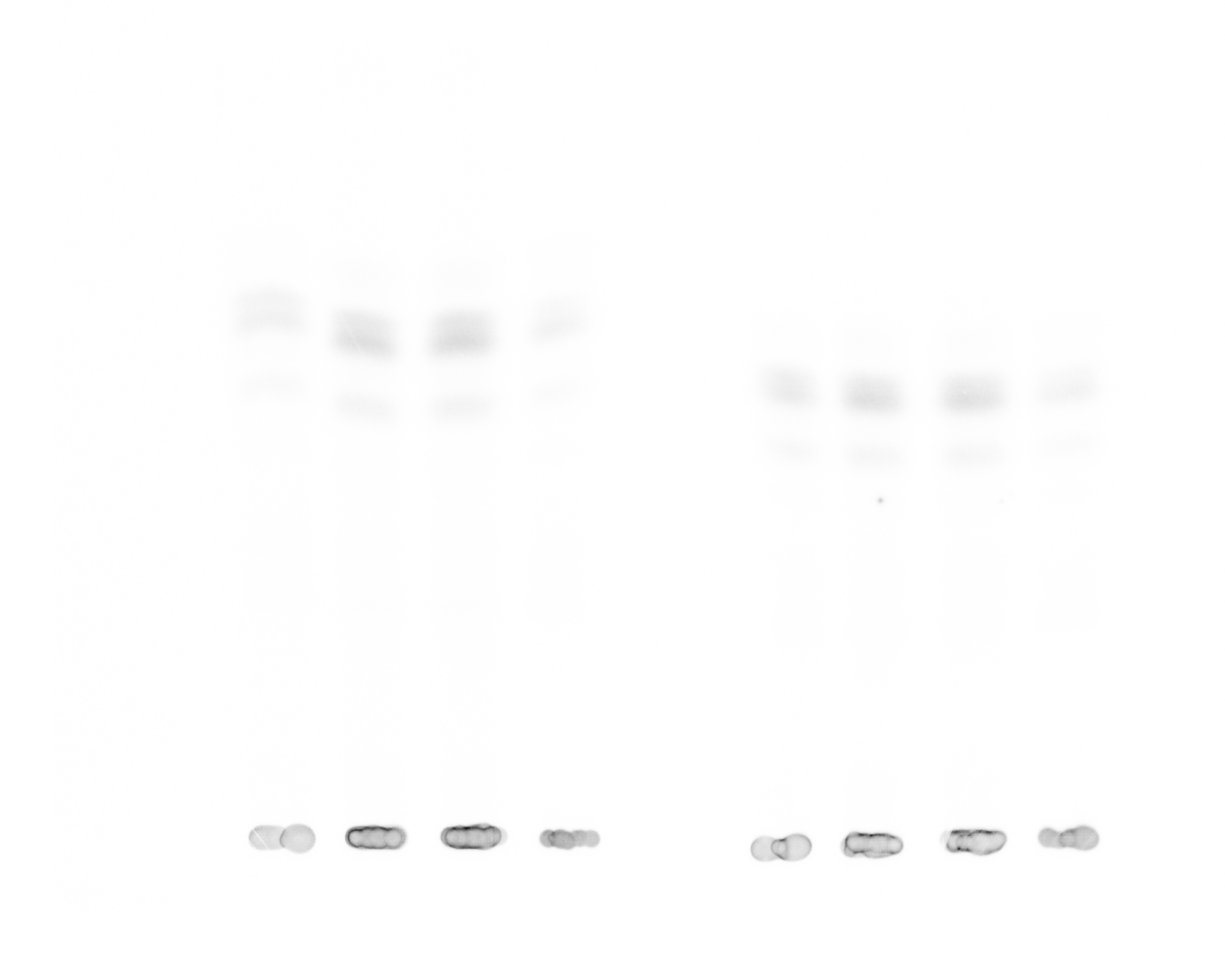

Supplement: Figure 6—source data 1. [file elife-80218-fig6-data1.zip › Figure 6B-Source Data 6B raw unedited.tif]

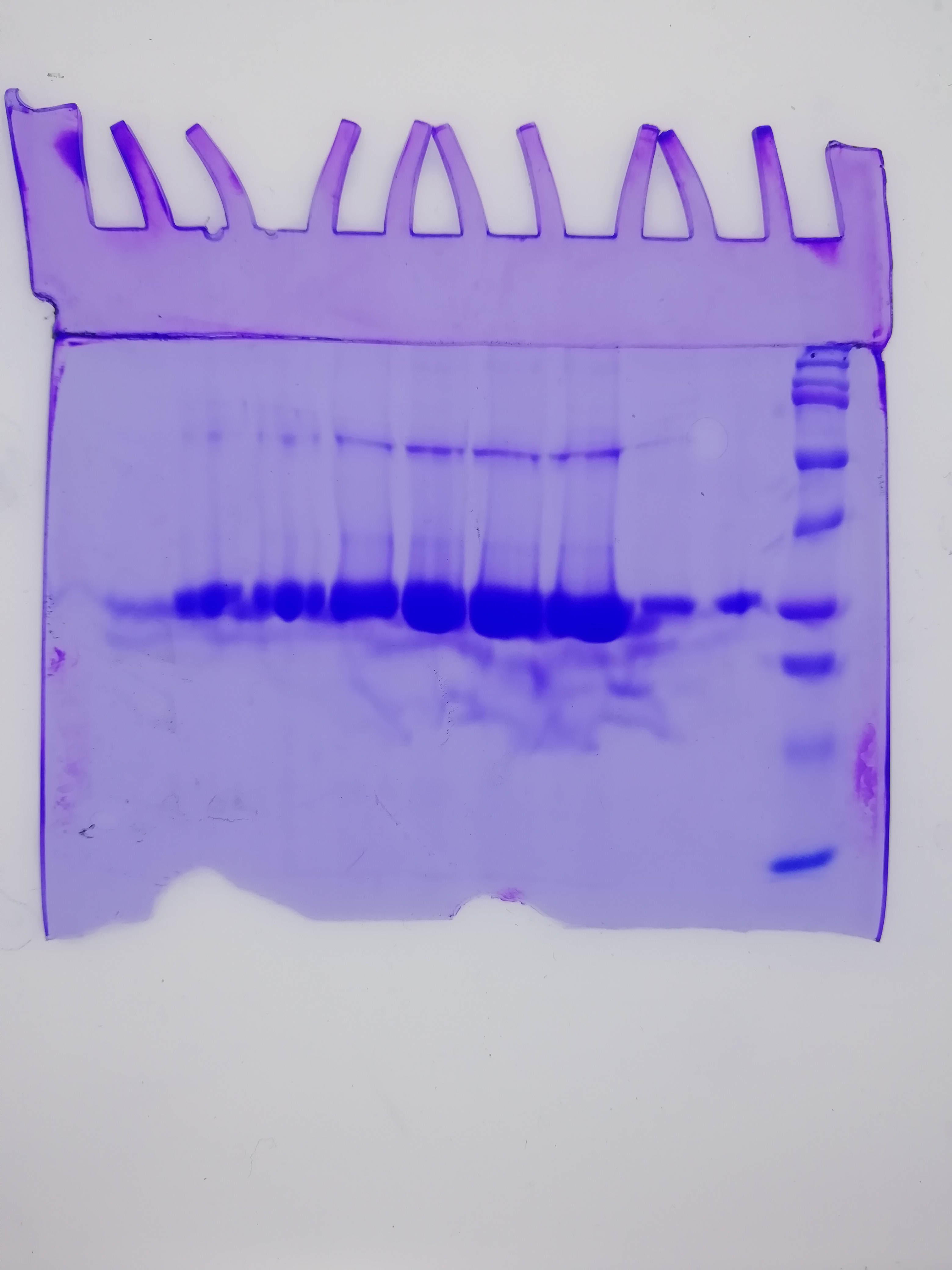

Supplement: Figure 10—figure supplement 1—source data 1. [file elife-80218-fig10-figsupp1-data1.zip › Figure 10-figure supplement 1b- Source Data 1b raw unedited.jpg]

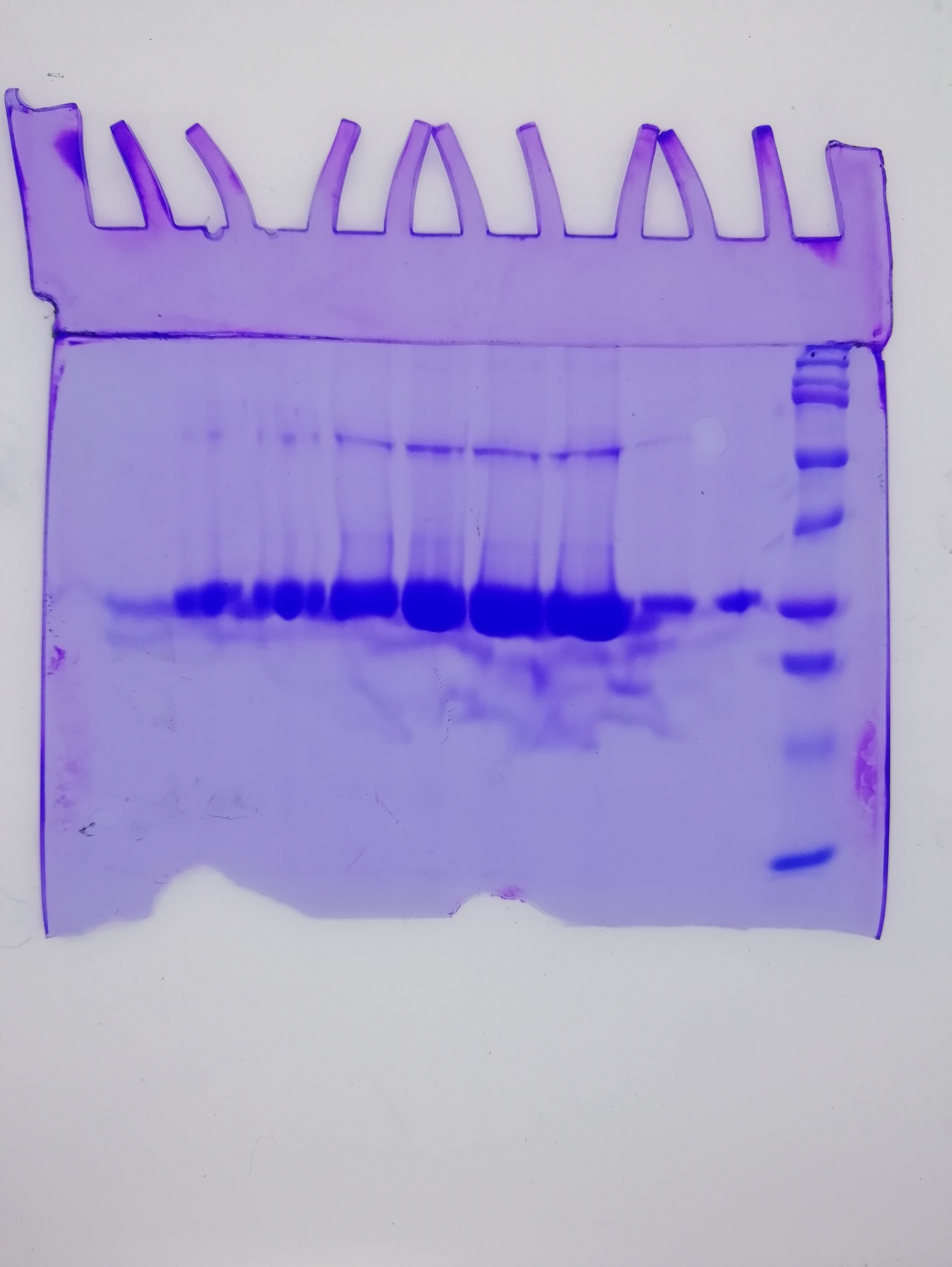


Peak 2 Marker

27 KDa

25 KDa

Supplement: Figure 10—figure supplement 1—source data 1. [file elife-80218-fig10-figsupp1-data1.zip › Figure 10-figure supplement 1b-Source Data 1b labelled.docx]

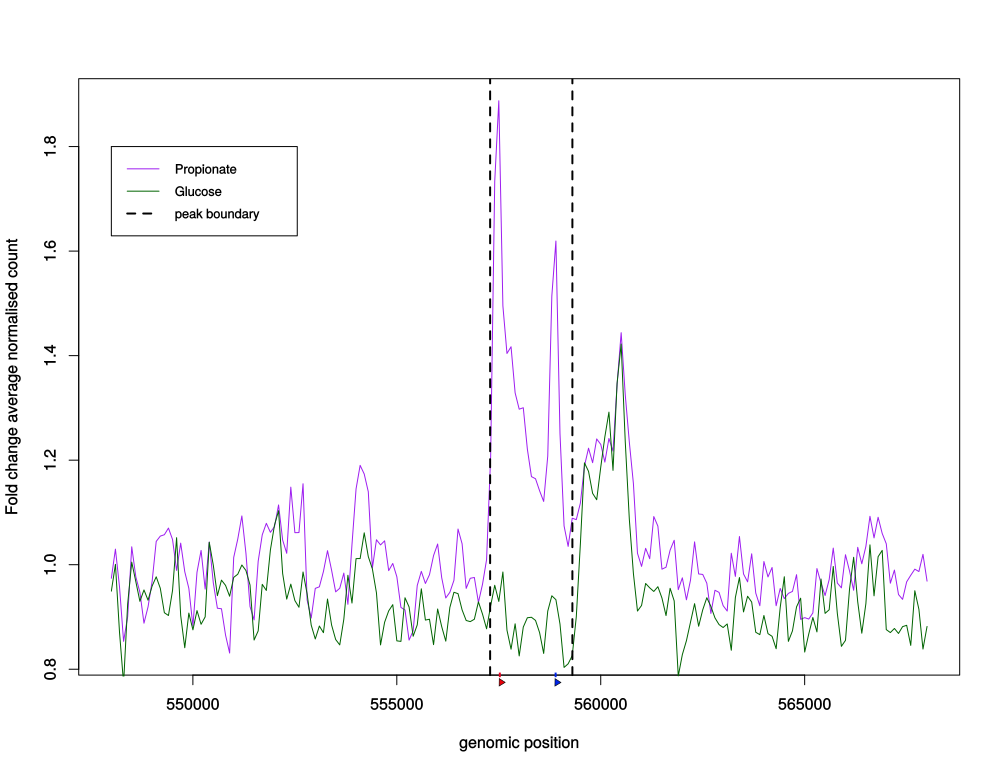

Supplement: Figure 11—source data 1. — Horizontal axis shows genomic positions on the reference genome with the bottommost horizontal panel representing genes with positions obtained from the.gff file. Each horizontal panel represents histogram of mapped reads (bin size of 100 base pairs) in pull down sample which is followed by the input sample (without the pull down) and is followed by the representation of peak regions predicted by the macs2. [file elife-80218-fig11-data1.zip › Figure 11- Source Data File 11_1.png]

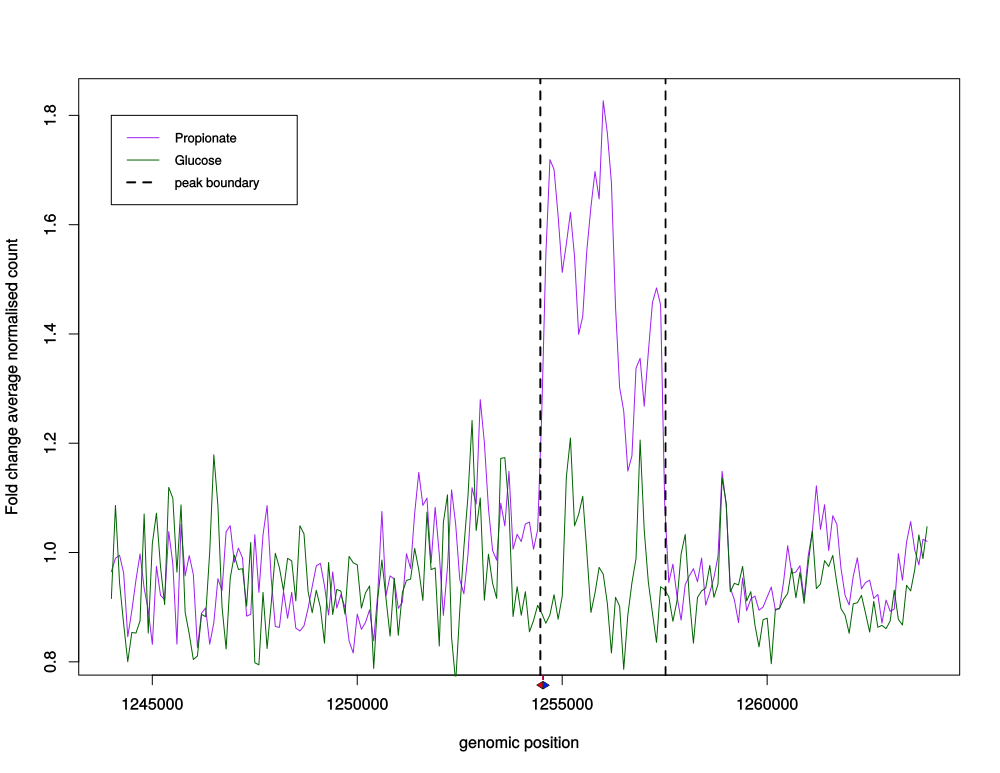

Supplement: Figure 11—source data 1. — Horizontal axis shows genomic positions on the reference genome with the bottommost horizontal panel representing genes with positions obtained from the.gff file. Each horizontal panel represents histogram of mapped reads (bin size of 100 base pairs) in pull down sample which is followed by the input sample (without the pull down) and is followed by the representation of peak regions predicted by the macs2. [file elife-80218-fig11-data1.zip › Figure 11- Source Data File 11_2.png]

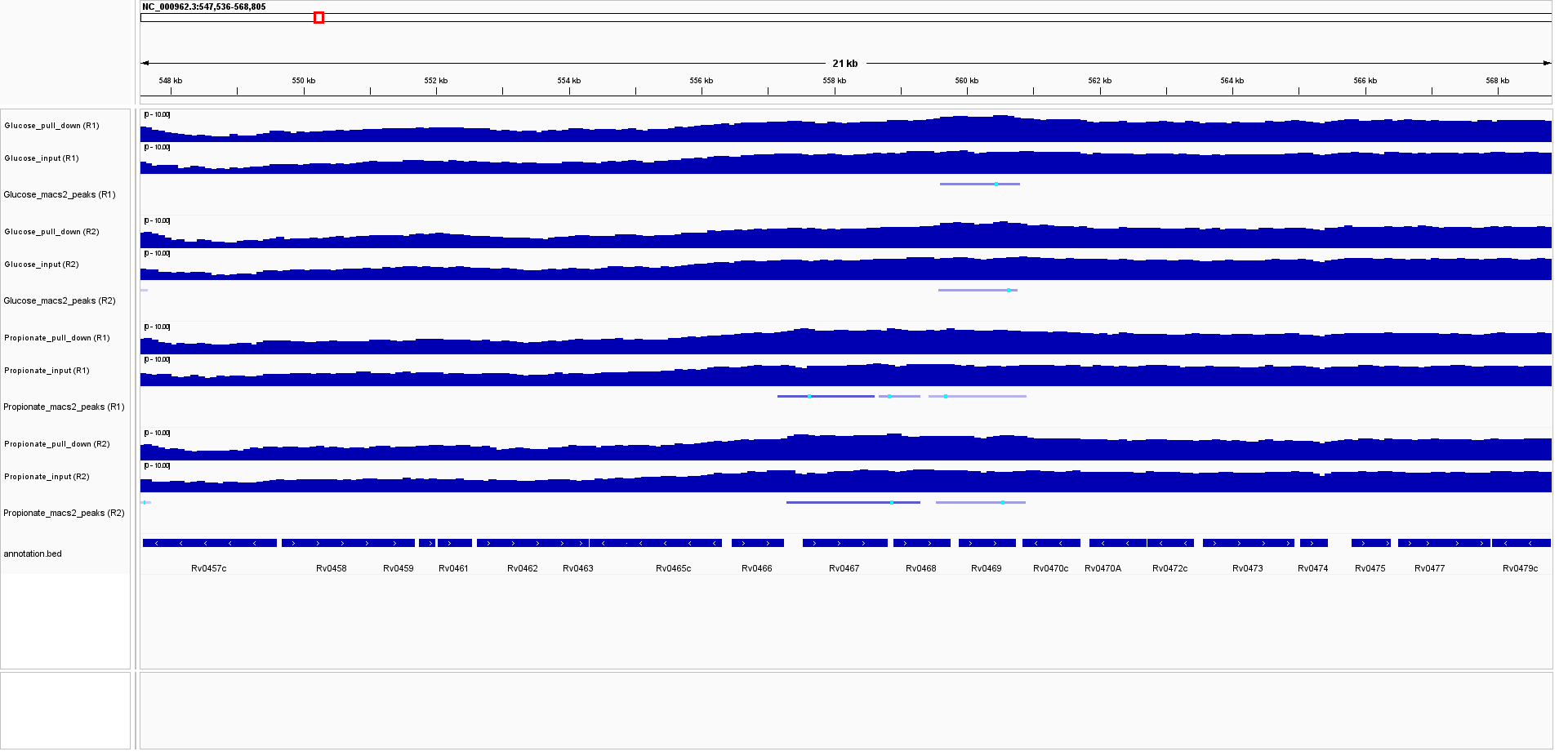

Supplement: Figure 11—source data 1. — Horizontal axis shows genomic positions on the reference genome with the bottommost horizontal panel representing genes with positions obtained from the.gff file. Each horizontal panel represents histogram of mapped reads (bin size of 100 base pairs) in pull down sample which is followed by the input sample (without the pull down) and is followed by the representation of peak regions predicted by the macs2. [file elife-80218-fig11-data1.zip › Figure 11- Source Data File 11_3.png]

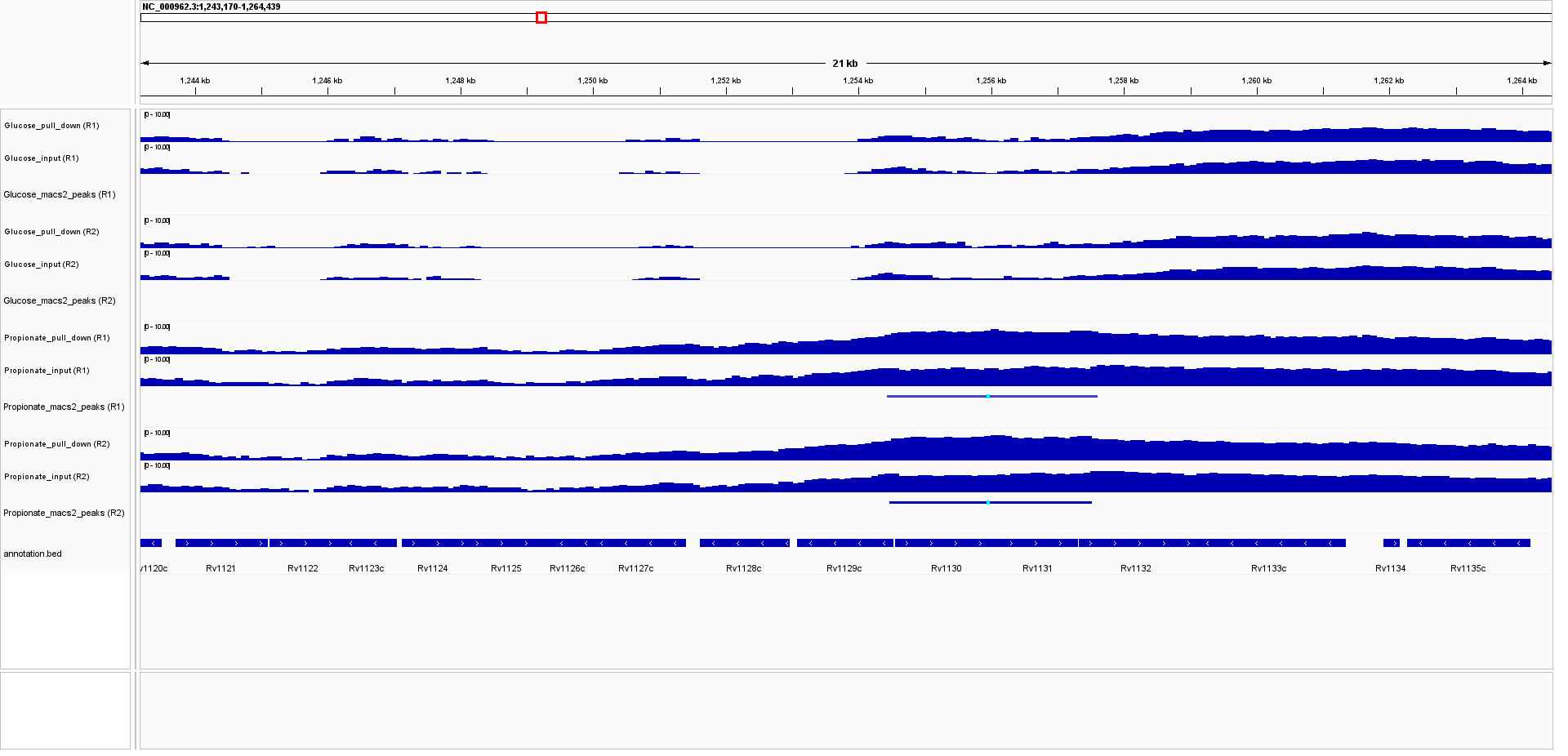

Supplement: Figure 11—source data 1. — Horizontal axis shows genomic positions on the reference genome with the bottommost horizontal panel representing genes with positions obtained from the.gff file. Each horizontal panel represents histogram of mapped reads (bin size of 100 base pairs) in pull down sample which is followed by the input sample (without the pull down) and is followed by the representation of peak regions predicted by the macs2. [file elife-80218-fig11-data1.zip › Figure 11- Source Data File 11_4.png]

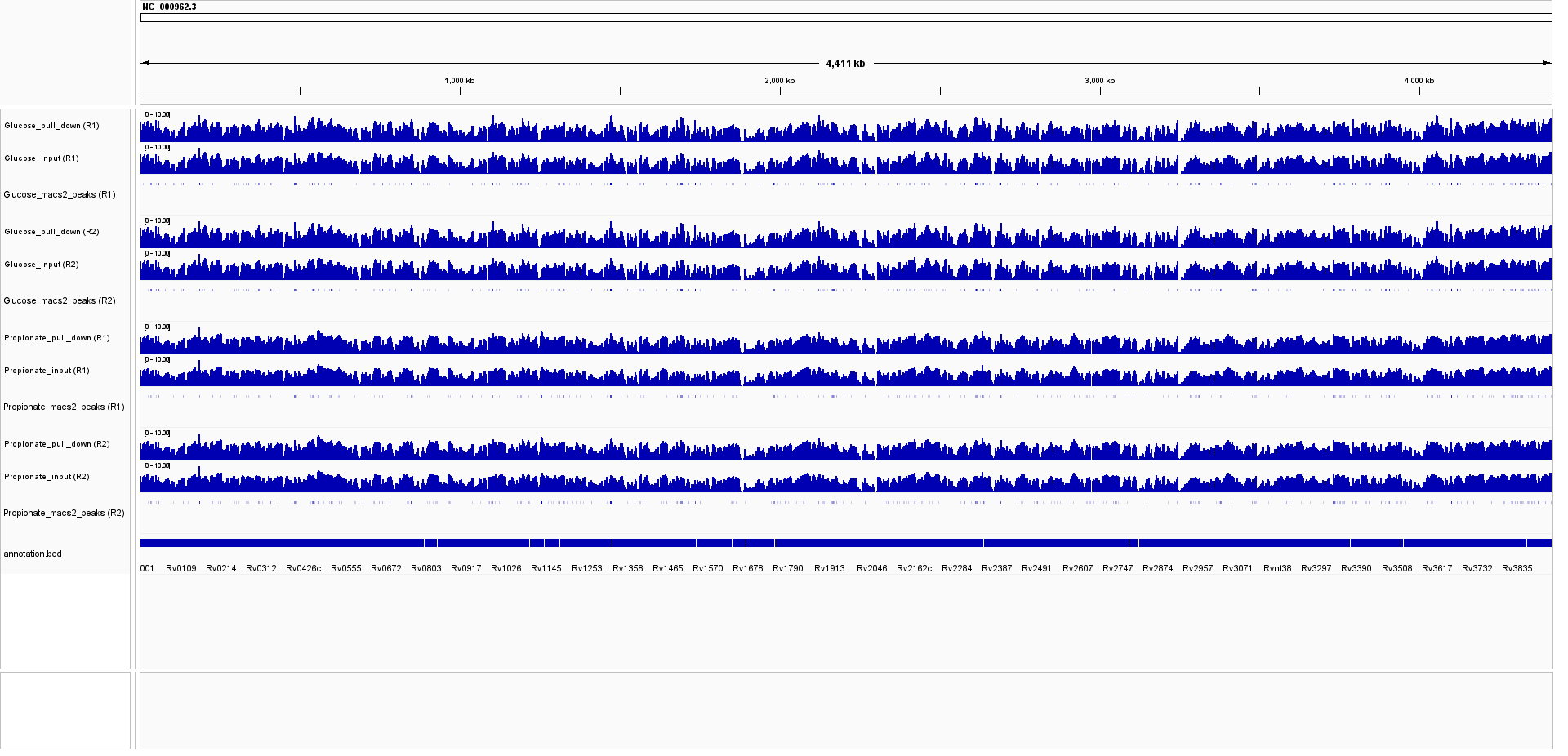

Supplement: Figure 11—source data 1. — Horizontal axis shows genomic positions on the reference genome with the bottommost horizontal panel representing genes with positions obtained from the.gff file. Each horizontal panel represents histogram of mapped reads (bin size of 100 base pairs) in pull down sample which is followed by the input sample (without the pull down) and is followed by the representation of peak regions predicted by the macs2. [file elife-80218-fig11-data1.zip › Figure 11- Source Data File 11_5.png]
